# Evaluation and analysis on suitability of human settlement environment in Qingdao

**DOI:** 10.1371/journal.pone.0256502

**Published:** 2021-09-27

**Authors:** Zhou Jiaxing, Liu Lin, Li Hang, Pei Dongmei

**Affiliations:** Shandong University of Science and Technology, Qingdao, China; Al Mansour University College-Baghdad-Iraq, IRAQ

## Abstract

Human settlement environment is space places closely related to human production and life, and also surface spaces inseparable from human activities. As a coastal city in the east of China, Qingdao has a relatively high level of urbanization. However, it also along with many urban problems at the same time, among which the problem of human settlement environment has attracted more and more general attention from people. According to the characteristics of human settlement environment in Qingdao, the research constructs an index system with 10 index factors from natural factors and humanity factors, and proposes a comprehensive evaluation model. Evaluate and grade suitability of human settlement environment in Qingdao, explore the spatial aggregation and differentiation of the quality of human settlement environment, and reveal the internal connection of spatial evolution. The results indicate that the overall livability of Qingdao is relatively good, showing a multi-center and radial driving development. The distribution of livability is uneven, showing a decreasing spatial distribution law from the coast to the inland, and the quality of human settlement environment in Jiaozhou Bay and the coastal areas is relatively high. Qingdao is mainly based on natural livability, supplemented by humanity livability, compared with natural suitability, the spatio-temporal evolution characteristics of humanity livability have experienced three stages: rising-contradictory rising-harmonious rising. The quality of human settlement environment has obvious spatial correlation and is positively correlated with the degree of agglomeration, and the agglomeration of blocks with a higher quality of human settlement environment is higher than that of blocks with a lower level. The rule of human settlement environment changing over time is that areas with high quality of human settlement environment begin to shift from the city center to the north and the south, transforming into multi-point development, and overall environmental suitability has been improved. According to the results of the comprehensive evaluation, combined with its local development status and policies, the research puts forward developmental suggestions for the construction of human settlement environment in Qingdao, and provides decision-making basis for relevant departments to solve the problem of deterioration of human settlement environment.

## Introduction

In recent years, with the continuous acceleration of urbanization, urban governance issues have become increasingly complex and diversified [1]. The problems of settlements environment in many cities have become increasingly prominent. Human settlement environment refers to the unity of nature, humanity and space, formed under a certain geographical environment background. At present, due to the rapid development of urbanization caused by the change in regional geographical environment, human settlement environment in Qingdao is facing tremendous pressure and challenges. At the same time, the irrational use of natural resources and the destruction of the ecological environment also make human settlement environment deteriorate sharply, threatening the sustainable development of human [2, 3]. How to scientifically and rationally evaluate urban human settlement environment and promote the harmonious coexistence between man and nature has become an urgent issue facing all countries in the world including China in the new century.

Foreign research on human settlement environment is relatively early. In Ancient Rome period, Vitruvius considered the influence of natural conditions and architectural planning and design on suitability of human settlement environment in Ten Books of Architecture [4]. With the continuous development of social science and technology, scholars have also proposed some new research techniques and methods [5]. Achillas et al. (2011) conducted a research on emission reduction in the urban areas of Greece based on Analytic Hierarchy Process, and provided reference for urban energy conservation [6]. Akuraju V et al. (2020) used 11th Sustainable Development Goal (SDG11) index to explore the impact of urban expansion scale on cities and communities with sustainable development [7]. Clark M (2001) believed that the gap between the rich and the poor caused by the market effect leads to the difference of human living environment between cities, and advocated that the government should play the role of macro-control to solve the problem of the gap between the rich and the poor [8]. Herath H et al. (2018) investigated the impact of urban green infrastructure on enhancing microclimate conditions in tropical urban, and the research showed that urban greening strategy design could effectively improve the tropical urban environment and outdoor thermal comfort [9]. Danielaini T T et al. (2018) pointed out the challenge of improving regional sustainability was very significant in the urban fringe transition zone, and defined five basic satisfaction indexes. The research showed that livability satisfaction varied depending upon the level of urbanization in the Cirebon Metropolitan Region (CMR) [10].

Academician Wu Liangyong (2001) first proposed and established "Science of Human settlement environment" in 1993. Therefore, the research of human settlement environment in China has entered a new systematic and scientific stage [11]. Yang Xue et al. (2016) established a comprehensive index of human settlement environment from the perspective of nature and humanity, and analyzed the correlation between human settlement environment and population distribution in the Beijing-Tianjin-Hebei region [12]. Based on Remote Sensing (RS) technology, Xu Hanqiu et al. (2012) proposed Remote Sensing Ecological Index (RSEI) integrating vegetation, humidity, surface temperature and urban buildings etc., which can rapidly monitor and evaluate the ecological environment of the city [13]. Xia Yu et al. (2017) discussed human settlement environment in the Yangtze River Delta from the perspective of humanistic environment and ecological environment suitability, and analyzed its spatial distribution characteristics [14].

At present, research on human settlement environment is mainly based on natural factors such as terrain, climate, hydrology, vegetation, etc. [15]. There are relatively few researches on humanity suitability dominated by humanity factors. Although the comprehensive evaluation of suitability of human settlement environment based on raster data and statistical data combines human environmental factors, the cost of obtaining statistical data is high and largely difficult. During analysis, the units to be evaluated cover a wide area, and cannot be analyzed pixel by pixel, which makes the ability of spatial analysis is not strong. In response to the above problems, the research uses natural and human factors to construct an evaluation index system for suitability of human settlement environment, introduces urban Point of Interest (POI) data, combines RS raster data and meteorological data, explores the evaluation methods of suitability of human settlement environment, and constructs a comprehensive evaluation model. The contribution of this research is not only constructing a comprehensive evaluation model with multi-index to analyze the temporal and spatial distribution of suitability of human settlement environment in Qingdao, but also using methods such as Moran’s I to explore spatial aggregation and differentiation of the quality of human settlements from the perspectives of spatial autocorrelation and cold and hot spots analysis, and revealing spatio-temporal evolution mechanism.

This research will be divided into three phase: first, explain the research area, the type and source of the data and the methods to be used later in this research, including the construction of the index system, the extraction of evaluation index, the index standardization, the calculation of index weight, and comprehensive evaluation of suitability. Then, demonstrate the results of suitability grading of human settlement environment in Qingdao, and analyze and discuss the results from spatio-temporal perspective. Finally, sum up the research, and put forward suggestions for the further development of human settlement environment in Qingdao.

## Material and methods

### Study area

Qingdao is a coastal city located in the southeast of Shandong Peninsula, with a geographical range of 119°30′E~121°00′E, 35°35′N~37°09′N. Qingdao consists of Shinan District, Shibei District, Licang District, Chengyang District, Laoshan District, Jimo District, Huangdao District, Jiaozhou City, Pingdu City, and Laixi City, a total of ten urban units. In terms of terrain, Qingdao is a hilly coastal city with high terrain in the east and low terrain in the west. In terms of climate, Qingdao is located in the north temperate monsoon zone, with obvious characteristics of oceanic climate. In terms of hydrology, the existing rivers in Qingdao are all monsoon rain source rivers, mostly independent mountain rivers.

**Fig 1:**
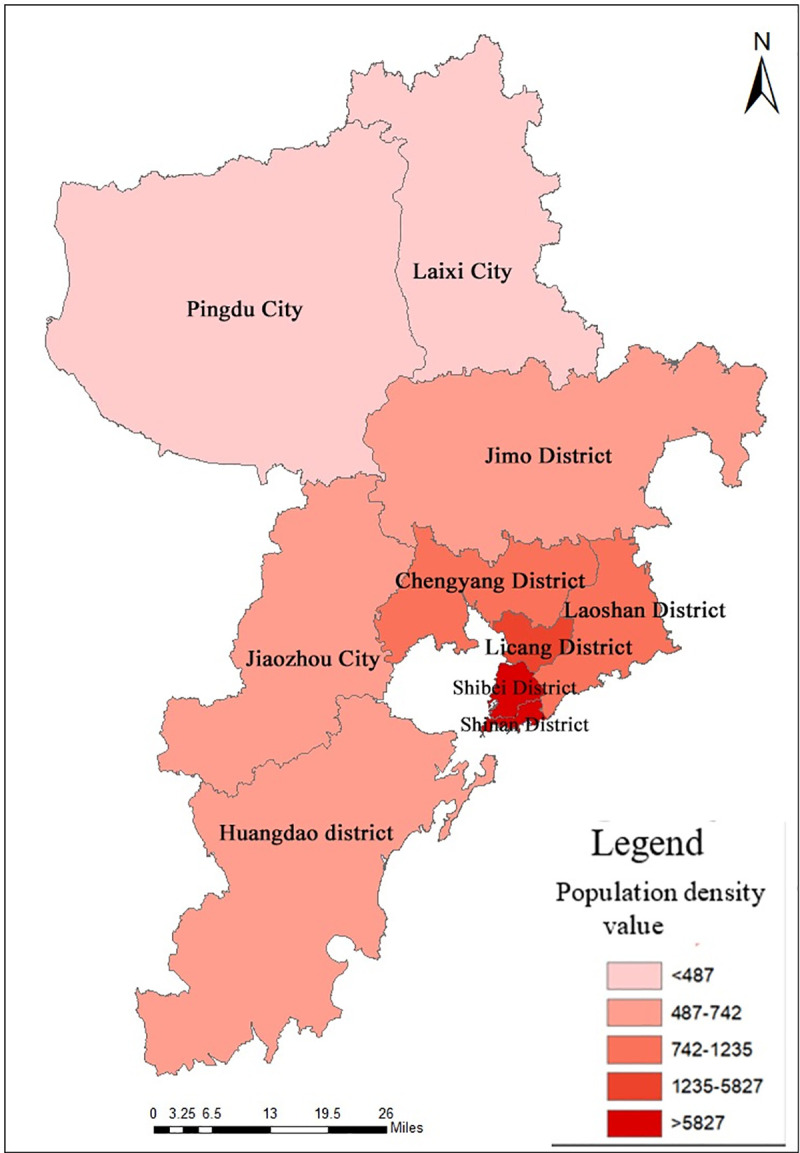
Population density distribution in various urban areas of Qingdao.

Qingdao has achieved a relatively high level of economic development with its superior geographical location. According to Statistical Yearbook of Qingdao issued in 2019, Gross National Product (GNP) of Qingdao is 1200.152 billion yuan. By the end of 2018, the total permanent population of Qingdao was 9394.8 thousand. The population distribution in Qingdao is not uniform, shown as Fig 1, there are large differences between urban areas. From the urban district level, Shinan District has the highest population density with 18,265 people per square kilometer, followed by Shibei District, Licang District, and Chengyang District. In terms of regional context, densely populated areas are mainly concentrated in Jiaozhou Bay, the southern of the research area, and the eastern coastal area of Jiaozhou Bay is the most densely populated. The sparse population density areas are distributed in the mountainous and hilly area in the north of the research area. Among them, Pingdu City has the lowest population density with 435 people per square kilometer.

### Data collection

The data used in the research mainly includes 7 categories: RS image data, terrain data, meteorological data, road network data, traffic facilities data, air quality data, and nighttime light data. The data sources and related characteristics are shown in Table 1.

**Table 1:** Data sources and characteristics.

Data Type	Data Content	Data Source	Data Description
Remote sensing image data	Image data of Qingdao in 2010, 2013, 2016 and 2019.	U.S. Geological Survey https://earthexplorer.usgs.gov/	With 30m spatial resolution, and may mosaic 2 images according to research needs.
Terrain data	DEM in Qingdao	Geospatial Data Cloud http://www.gscloud.cn/	Geo TIF format, With 30m spatial resolution, and UTM/WGS1984 projection.
Meteorological data	Driven dataset of ground meteorological elements in China regional	National Tibetan Plateau Data Center http://www.tpedatabase.cn	Including 7 elements such as near-surface temperature, near-surface wind speed and precipitation rate.
Road network data	Vector dataset of Chinese road	Open Street Map https://www.openstreetmap.org/	Including expressways, main roads, secondary roads and tertiary roads, etc.
Traffic facilities data	POI data of bus stops and parks	BIGEMAP	Including the type of POI data, latitude and longitude coordinates, and the city area, etc.
Air quality data	PM2.5 data of Qingdao in 2010, 2013, 2016 and 2019	Atmospheric Composition Analysis Group http://fizz.phys.dal.ca/~atmos/martin/ China Air Quality Online Monitoring and Analysis Platform https://www.aqistudy.cn/	With 1km spatial resolution, including the PM2.5 value of each pixel point.
Nighttime light data	Nighttime light data of Qingdao in 2010, 2013, 2016 and 2019	Resource and Environment Science and Data Center http://www.resdc.cn/ Earth Observation Group, Payne Institute for Public Policy https://payneinstitute.mines.edu/eog/ [16]	With 1km resolution, including the DN value of each pixel point.

### Data analysis

**Fig 2:**
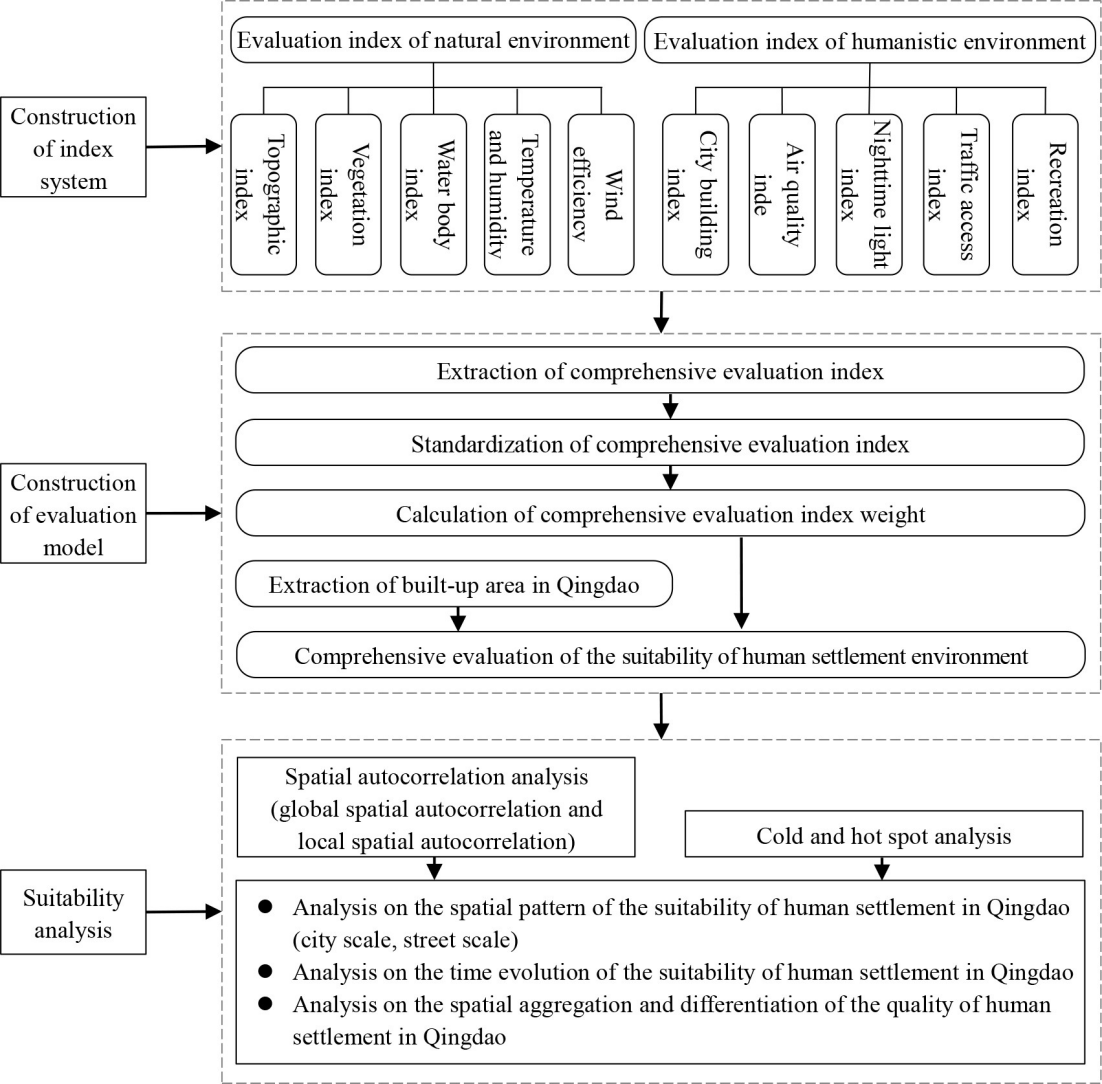
Flowchart of the research.

The data analysis methods and process in this research are shown in the Fig 2.

#### Construction of evaluation index system

According to the common characteristics of nature and humanity in Qingdao, a comprehensive evaluation index system for suitability of human settlement environment in Qingdao has been constructed by comprehensively considering natural and human factors, as shown in Table 2.

**Table 2:** Comprehensive evaluation index system for suitability of human settlement environment.

Evaluation index for suitability of human settlement environment			
Natural factors		Human factors	
Topographic index	RDLS	Air quality index	PM2.5 monitoring index
Vegetation index	NDVI	Nighttime light index	Nighttime Light RS index
Water body index	MNDWI	City building index	NDISI
Temperature and humidity index	THI	Traffic access index	ICSA-Tcost index
WEI	WEI	Recreation index	RI

#### Extraction of evaluation index

The Relief Degree of Land Surface (RDLS) index refers to the height difference between the highest point and the lowest point in a certain area [17, 18]. The selected area should be able to perfectly express the topographical characteristics of the research area. The formula which calculates RDLS based on Digital Elevation Model (DEM) data is:

$$RDLS=\frac{ALT}{1000}+\left\{[Max(H)-Min(H)]\times [1-\frac{P(A)}{A}]\right\}/500$$ (1)

In the formula, *RDLS* is the terrain relief degree, *ALT* is the average altitude in the statistical unit, *Max*(*H*) and *Min*(*H*) are respectively the highest altitude and the lowest altitude in the research area, *P*(*A*) is the flat area in the research area, *A* is the total area of the region, and 500 is the height of the Chinese reference mountain.

**Fig 3:**
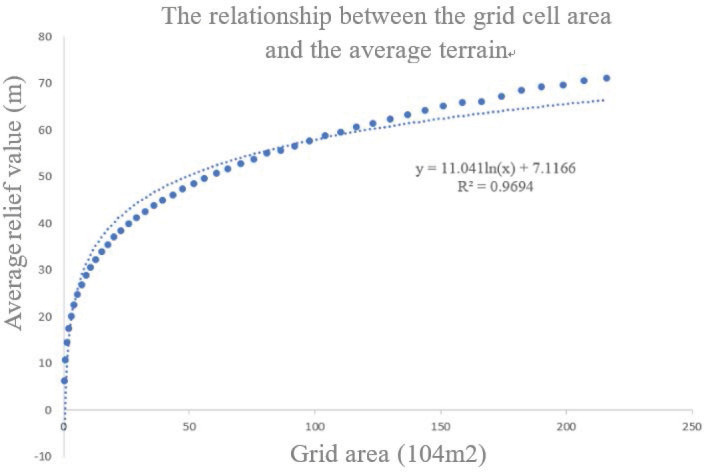
Fitting curve of the correspondence relationship between grid unit area and average relief degree.

The key to the calculation of RDLS is the determination of the best analysis window. The research uses a window analysis method based on raster data. Through the proximity tools of the Geographic Information System (GIS) platform, calculate the height difference between the highest altitude and the lowest altitude under the corresponding unit size from 2×2 pixels to 49×49 pixels, as shown in Table 3. Fit the dependent variable (average RDLS) and independent variable (grid size) of the data in the table, as shown in Fig 3. When R^2^ = 0.9694, the fitting effect is better. It can be seen from the figure that the increasing trend of the average RDLS value gradually flattens with the increase of the grid area, and it can be judged that there must be a turning point from steep to gentle. In order to determine this more accurately, use the mean change-point analysis method to analyze, and the calculation formula is as follows:

$$S_{i}=\sum_{t_{1}=1}^{i-1}{{(x}_{t_{1}}-{\bar{x}}_{i1})}^{2}+\sum_{t_{2}}^{n}{{(x}_{t_{2}}-{\bar{x}}_{i2})}^{2}$$ (2)

$$S=\sum_{i=1}^{n}{\left(x_{i}-\bar{x}\right)}^{2}$$ (3)

In the formula, *x*_*i*_ (i = 1, 2…n) is the change-point, ${\bar{x}}_{i1}、{\bar{x}}_{i2}$ are respectively the arithmetic means of the two sections of samples (samples are divided into two sections with *x*_*i*_ as the boundary), ${\bar{x}}_{i}$ is the sample value, *t*_1_ = 1, 2, 3, …, i-1, *t*_2_ = i, i+1, i+2, …, n are the total number of samples. *S*_*i*_ is group variance, *S* is total variance. The point corresponding to the maximum *S*−*S*_*i*_ value is the change-point.

**Table 3:** Correspondence between grid unit size and average RDLS in the research area.

Grid Size	Grid Area (104m^2^)	Average relief degree of land surface (m)
2×2	0.36	6.143704261
3×3	0.81	10.67251015
…	…	…
7×7	4.41	22.52890186
8×8	5.76	24.75297338
…	…	…
12×12	12.96	32.24400086
13×13	15.21	33.82464112
…	…	…
19×19	32.49	42.37919839
20×20	36	43.70014227
…	…	…
30×30	81	54.91553921
31×31	86.49	55.61916959
…	…	…
48×48	207.36	70.42110508
49×49	216.09	71.04931022

**Fig 4:**
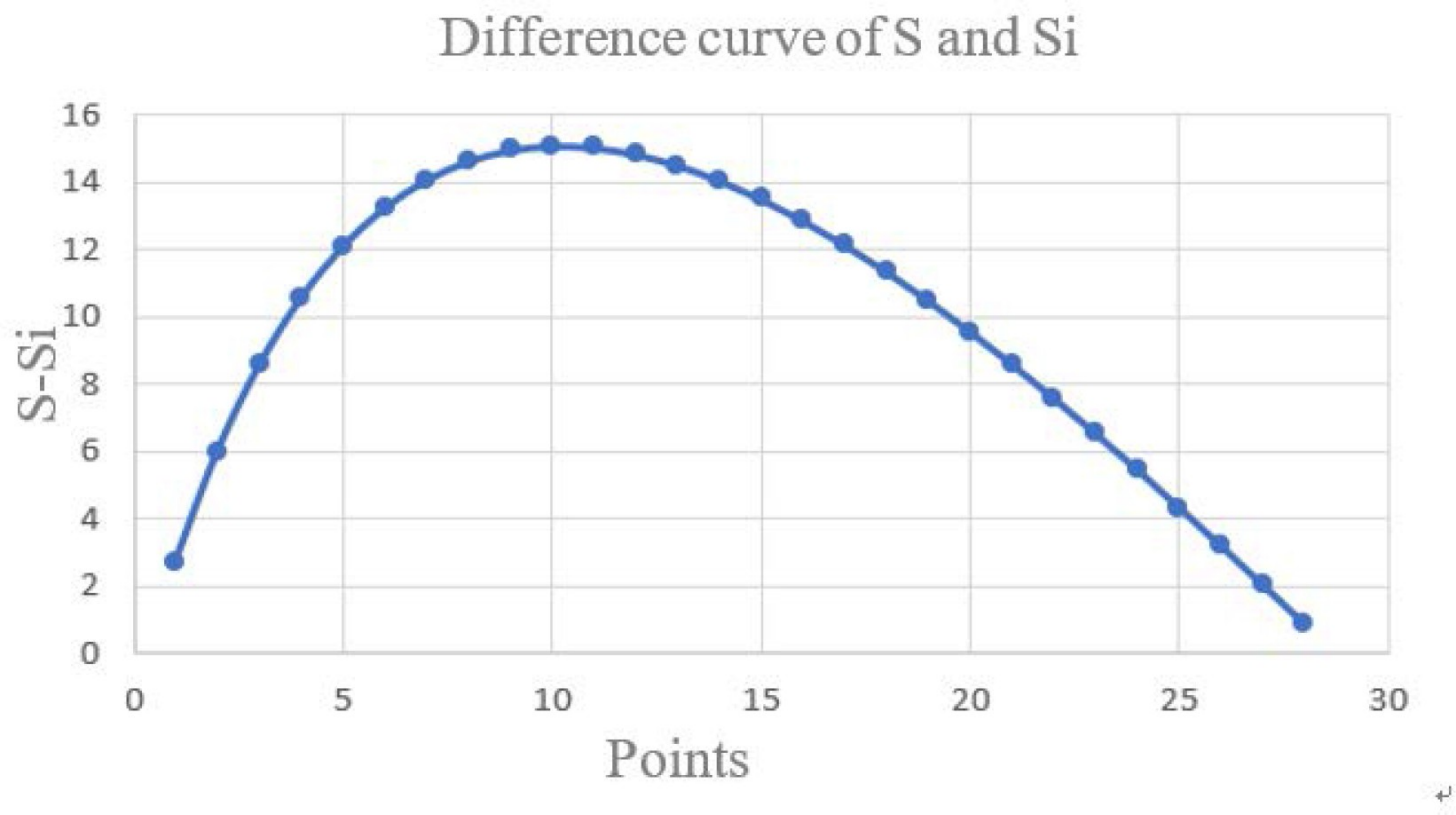
The statistical results of the mean change point method.

**Fig 5:**
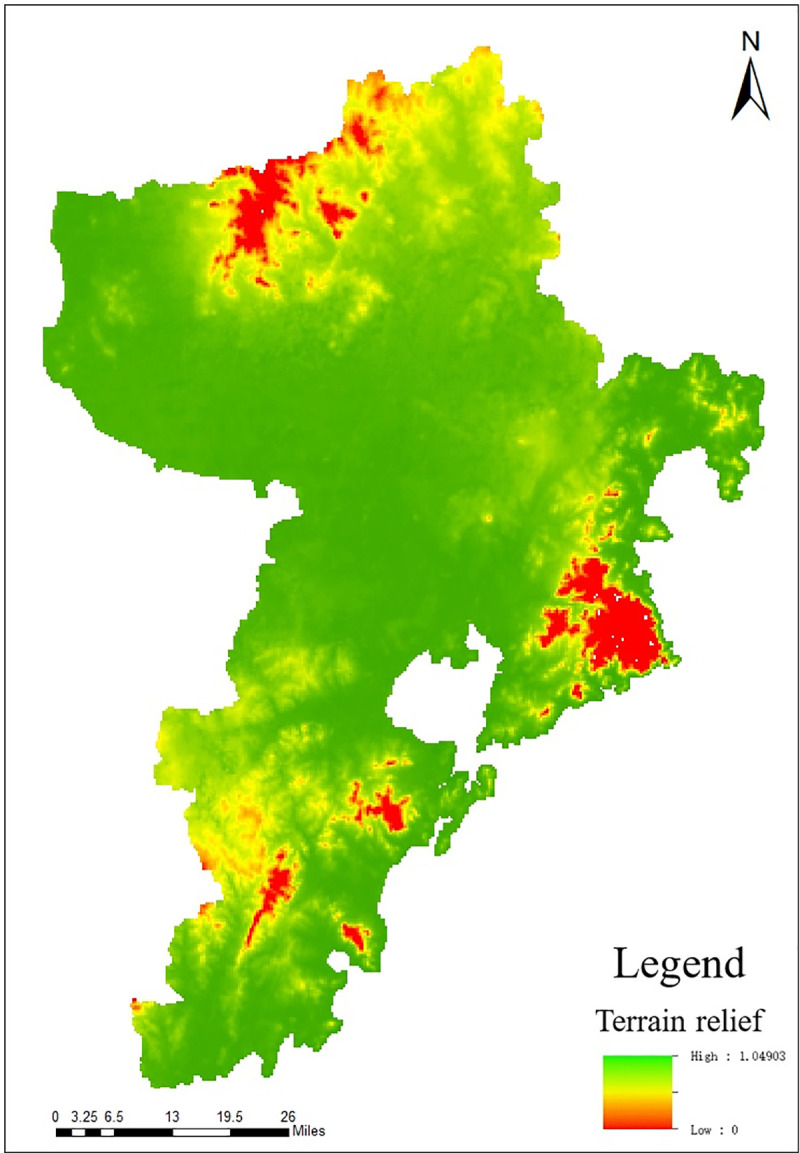
Distribution map of RDLS in Qingdao.

It can be seen from Fig 4 that *S*−*S*_*i*_ is the largest at the 10th point and the grid size corresponding to this point is 13, that is, the best window size for this research is 13×13. Based on the above results, calculate other input data using the proximity tools in ArcGIS, and finally obtain the topographic relief map according to formula (1), as shown in Fig 5.

Normalized Difference Vegetation Index (NDVI) means that according to the spectral characteristics reflected by vegetation, the visible light and near-infrared bands in the multi-spectral RS image are used to calculate the index factor of the land cover index [19, 20]. The research selects NDVI to reflect the vegetation suitability of urban areas, and its calculation formula is:

$$NDVI=\frac{NIR-R}{NIR+R}$$ (4)

**Fig 6:**
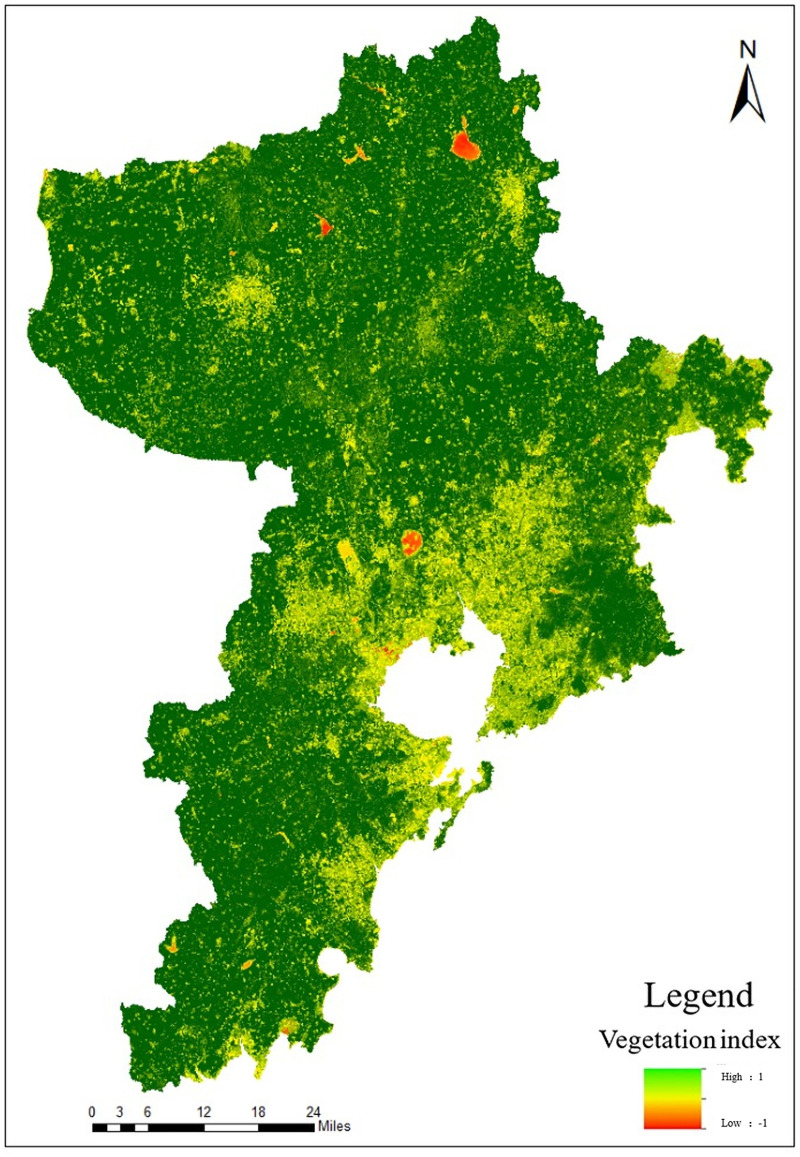
Distribution of NDVI in Qingdao.

In the formula, *NIR* represents near-infrared band, and *R* represents red band. The extraction result is shown in Fig 6.

Modified Normalized Difference Water Index (MNDWI) refers to the band calculation index factor obtained by normalizing the difference between the near-infrared band and the visible light band, which can clearly distinguish the ground object information such as water and vegetation in the image [21, 22]. The calculation formula of MNDWI is:

$$MNDWI=\frac{G-MIR}{G+MIR}$$ (5)

**Fig 7:**
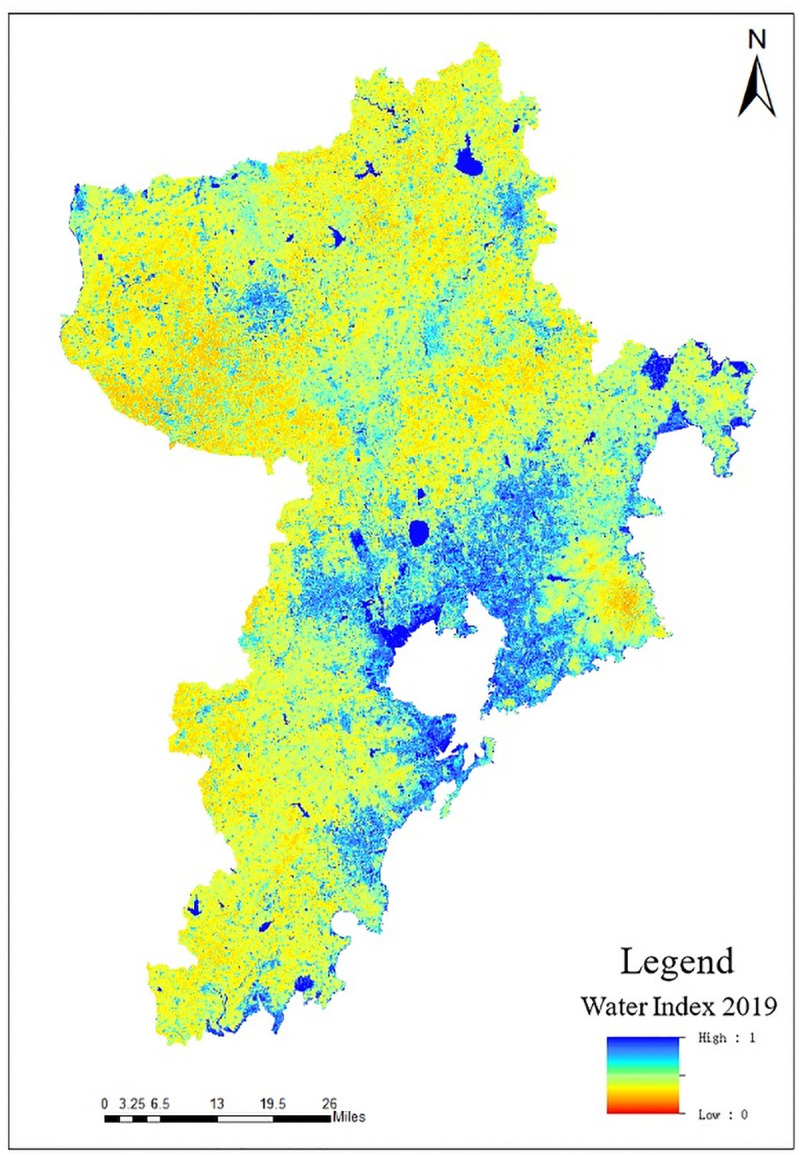
Distribution map of MNDWI in Qingdao.

In the formula, *MIR* represents mid-infrared band, and *G* represents green band. The greater the MNDWI value, the greater the water coverage. The extraction result is shown in Fig 7.

Temperature and Humidity Index (THI), also known as Discomfort Index, can reflect the comfort relationship between the human body and the surrounding environment [23, 24]. THI is a biometeorological index for evaluating human comfort under different climatic conditions. The research selects the average temperature and relative humidity as two factors, and uses THI model proposed by Thom to construct THI. The formula is as follows:

THI=1.8t+32−0.55(1−f)(1.8t−26) (6)

**Fig 8:**
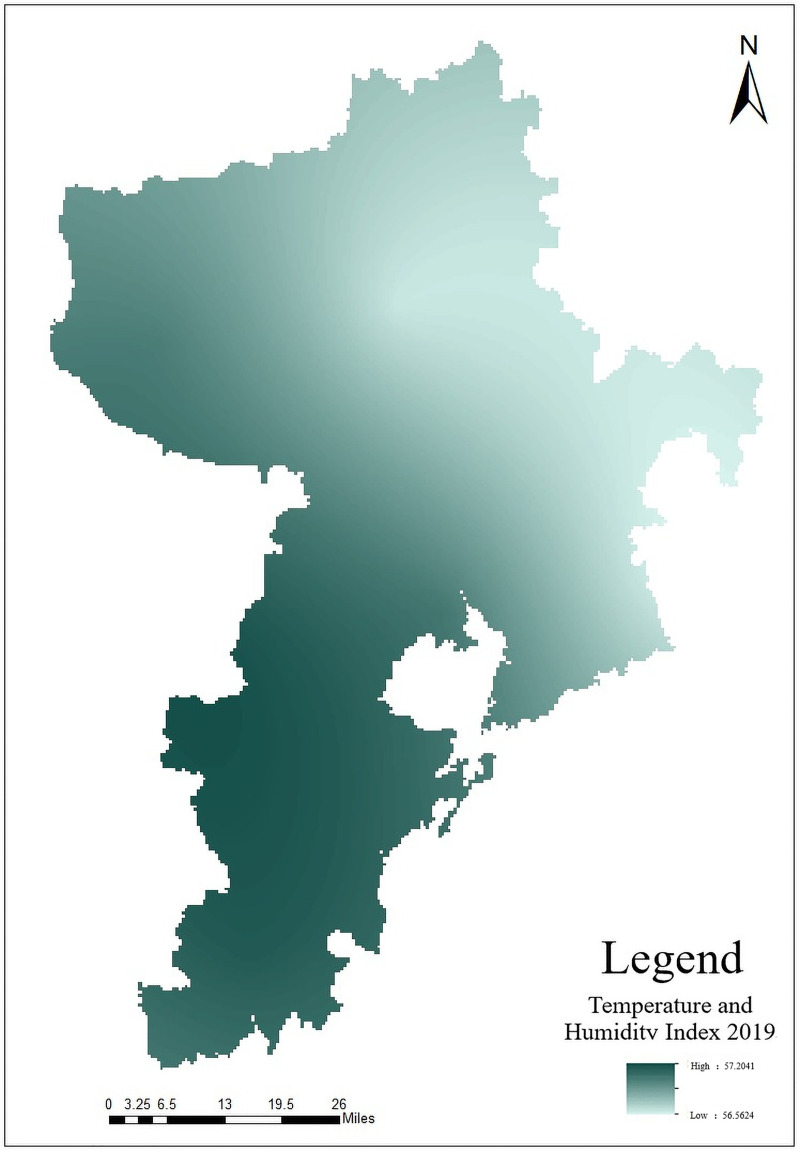
Distribution map of THI in Qingdao.

In the formula, *THI* represents Temperature and Humidity Index, *t* represents the monthly average temperature in units of °C, *f* is the monthly average air relative humidity. The extraction result is shown in Fig 8.

Wind Efficiency Index (WEI) refers to the comfort level of the human body brought by different wind power and temperature [25, 26], which quantitatively reflects the heat exchange between the human body and the surrounding environment, and its calculation formula is as follows:

$$WEI=-(10\times \sqrt{v}+10.45-v)\times (33-T)+8.55\times S$$ (7)

**Fig 9:**
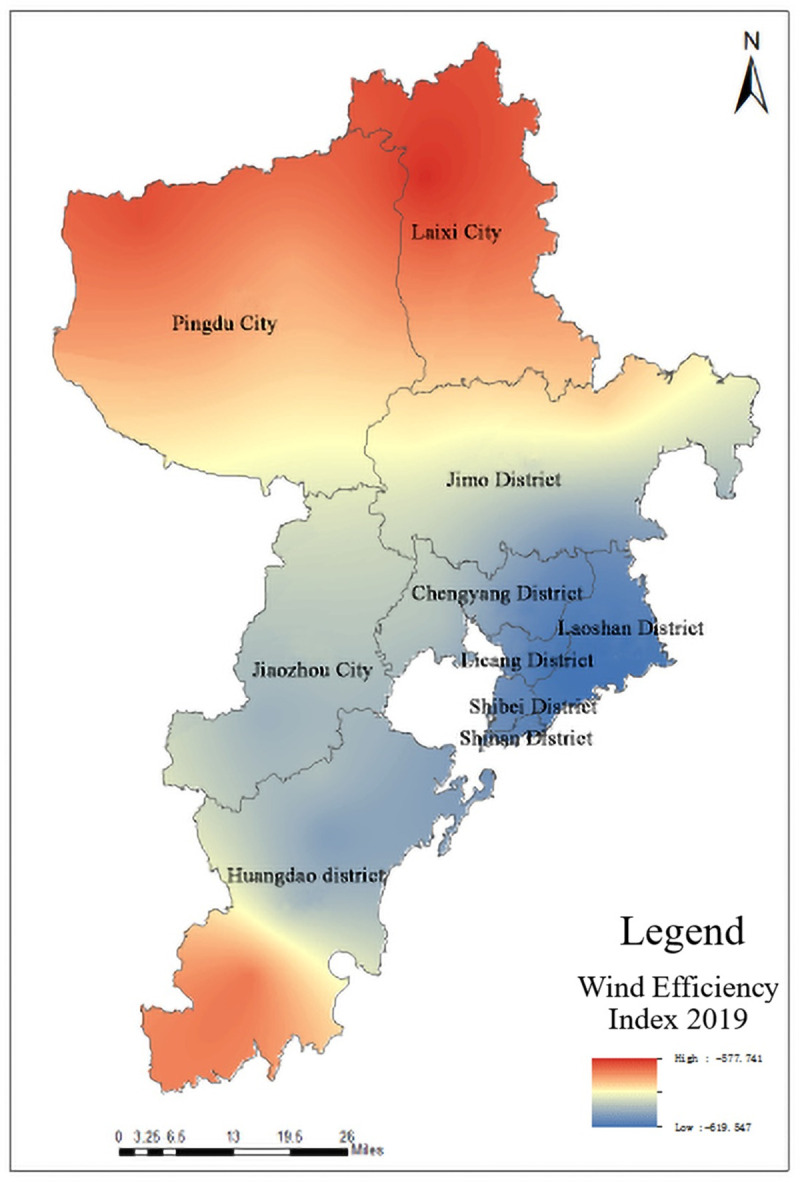
Distribution map of WEI in Qingdao.

In the formula, *v* is wind speed (m/s), *T* is temperature (°C), and *S* is sunshine hour (h/d). The calculated WEI distribution map is shown in Fig 9.

**Fig 10:**
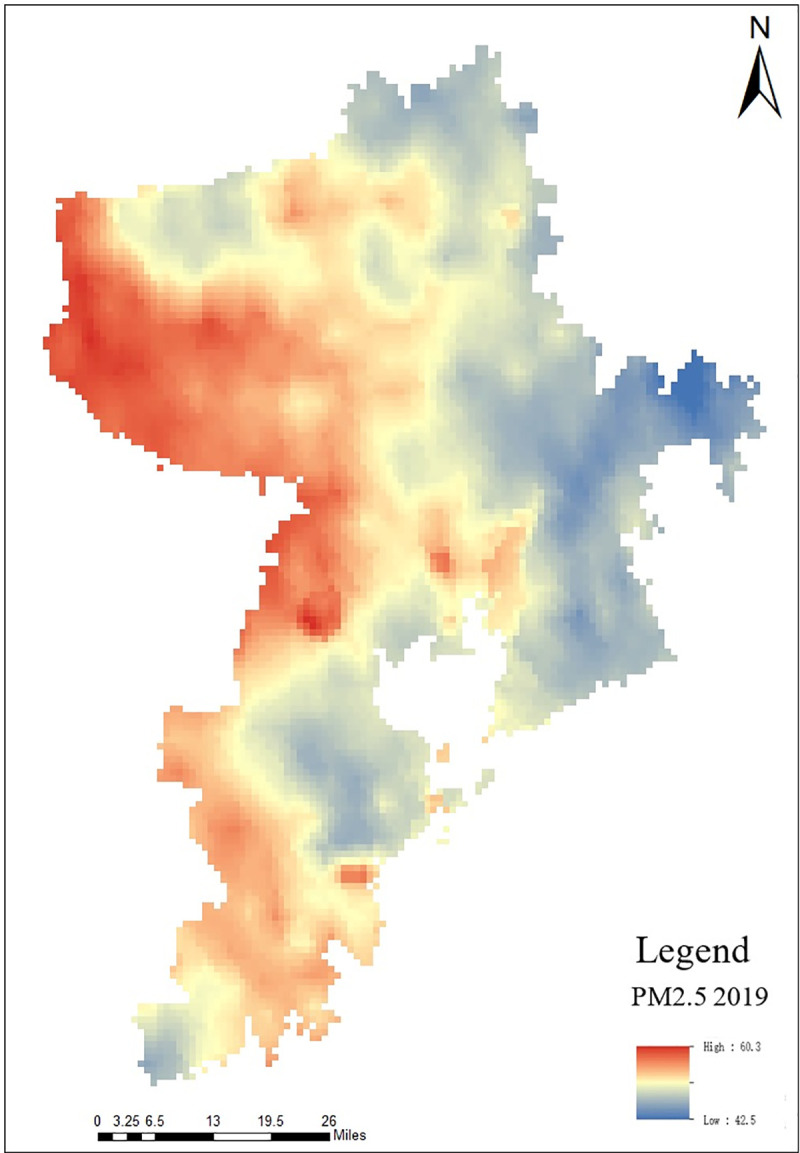
Distribution map of PM2.5 in Qingdao.

PM2.5 is selected as the index of air quality evaluation for suitability of human settlement environment in Qingdao. According to the air quality data of the Atmospheric Composition Analysis Group (http://fizz.phys.dal.ca/~atmos/martin/) and the China Air Quality Testing and Analysis Platform (https://www.aqistudy.cn/), the extraction results are shown in Fig 10.

**Fig 11:**
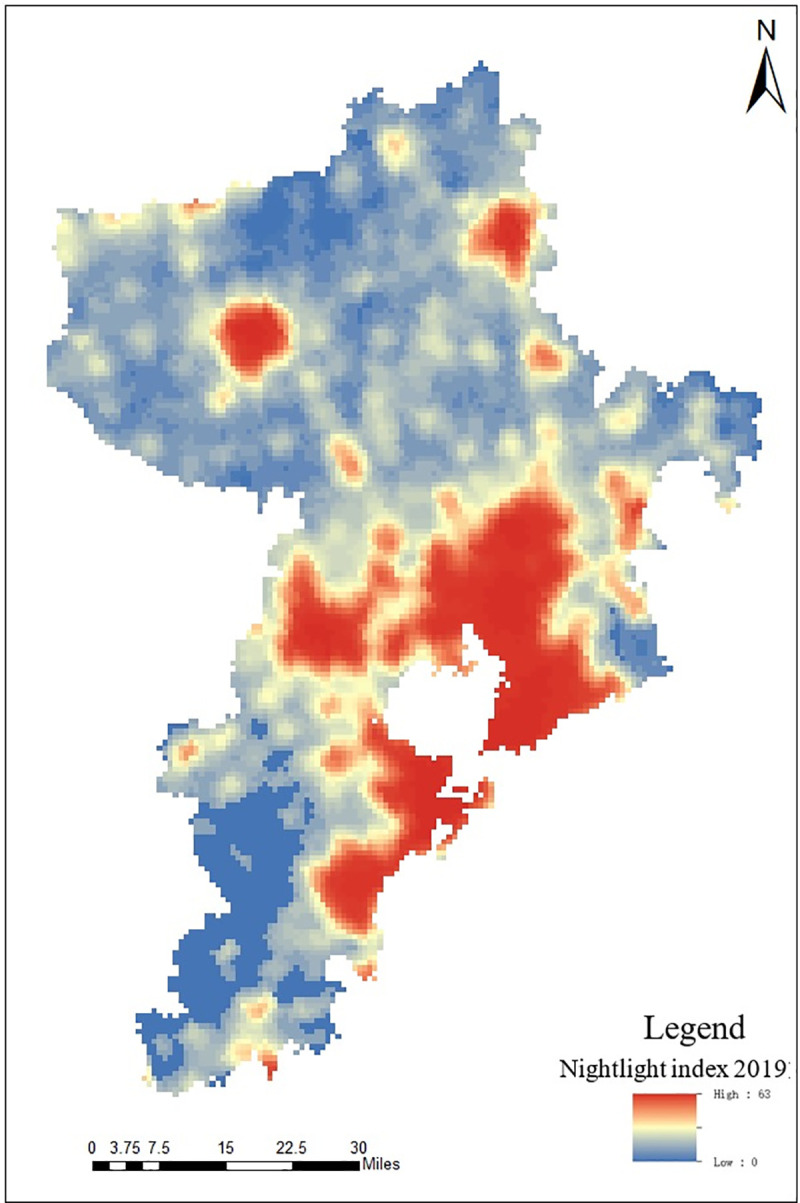
Distribution map of nighttime light index in Qingdao.

As an objective and real-time RS data to record the light intensity of ground buildings and roads, nighttime light RS images [27, 28] have been widely used in the identification and monitoring of social production and life [29–31]. According to the research results of Lu et al. (2008), nighttime light data is selected as the evaluation index of the humanity factors of suitability of human settlement environment in Qingdao [32], denoted as Nighttime Light RS index. According to the nighttime light products of Resource and Environment and Data Center of Chinese Academy of Sciences (http://www.resdc.cn/) and NPP satellite VIIRS sensor released by Earth Observation Group, Payne Institute for Public Policy (https://payneinstitute.mines.edu/eog/), the distribution of nighttime light RS data in Qingdao is shown in Fig 11.

Due to its own material composition, urban buildings are more sensitive to certain bands of RS images, which is reflected in the increased reflectance of near-infrared band and short-infrared band. The research uses Normalized Difference Impervious Surface Index (NDISI) to extract impervious surface information, which can effectively eliminate the interference of soil on impervious surfaces and improve the effectiveness of the information [33, 34]. Its calculation formula is as follows:

$$NDISI=\frac{TIR-[({VIS}_{1}+NIR+{MIR}_{1})/3]}{TIR+[({VIS}_{1}+NIR+{MIR}_{1})/3]}$$ (8)

**Fig 12:**
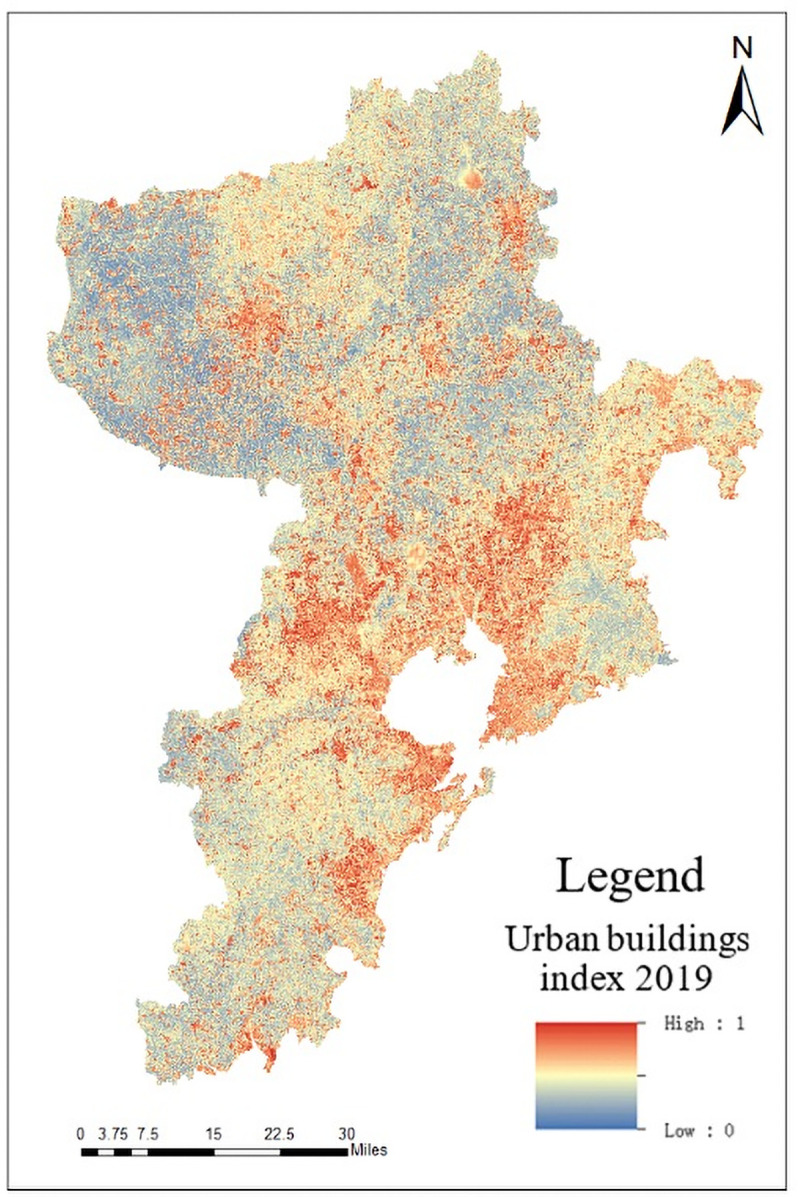
Distribution map of NDISI in Qingdao.

In the formula *NDISI* represents Normalized Difference Impervious Surface Index, *NIR MIR*_1_ and *TIR* respectively represent the near, middle and thermal infrared bands, *VIS*_1_ can be selected from visible bands, such as red, blue and green bands. The red band is selected as the input band. The city building index is calculated and obtained, as shown in Fig 12.

Traffic Accessibility Index (TAI) refers to the accessibility of bus stations in a city. The service range of traffic stations directly affects the convenience of residents travel [35, 36]. The research uses the service area range analysis method in network analysis to construct isochronous circle service area index based on time cost (ICSA-Tcost index), and determines the service range of each traffic station, so as to obtain suitability degree of traffic accessibility for human settlement environment. Set the service range as Manhattan distance *d*_*mh*_, the calculation formula is as follows:

$$cost=\frac{d_{mh}}{V}\times 60min/h$$ (9)

In the formula, *cost* is the time cost, and *V* is the set speed of spatial objects, and the unit is km/h.

**Fig 13:**
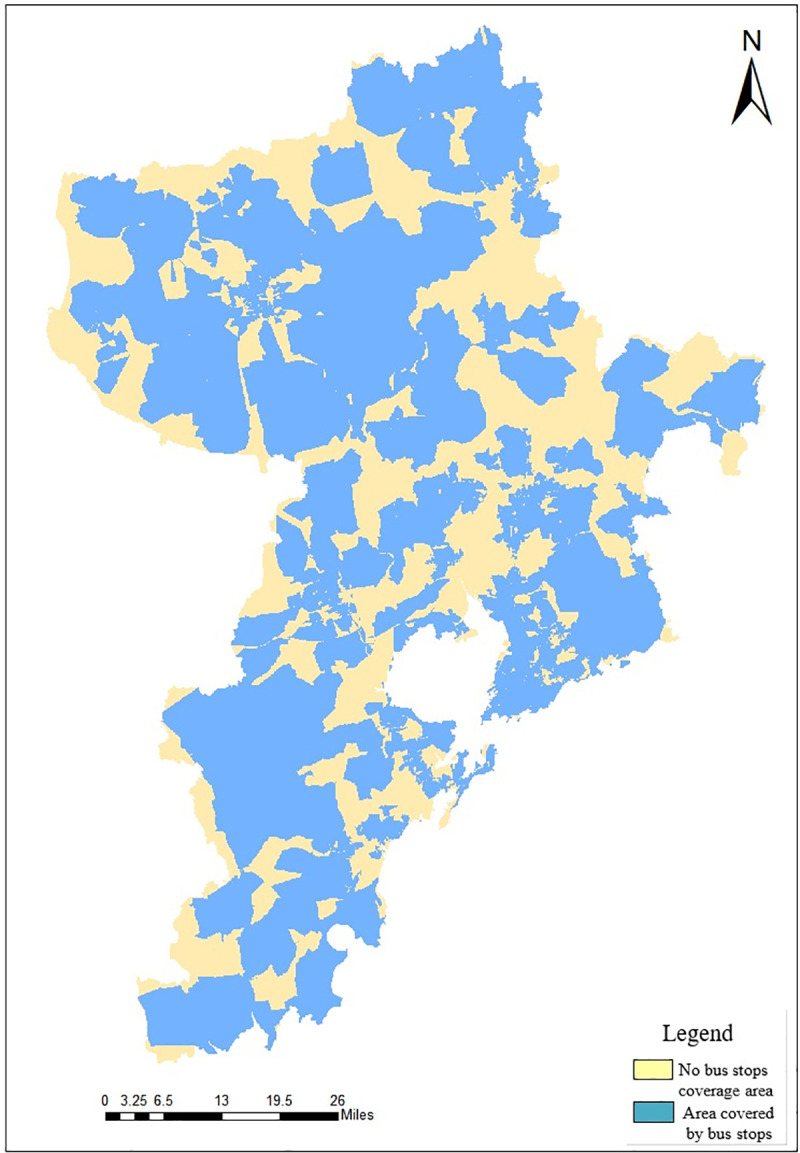
Distribution map of TAI in Qingdao.

According to the traffic conditions of Qingdao, calculate the time cost respectively when *d*_*mh*_ = 2*km*, 5*km*, 8*km*. Calculate the time cost for residents in the city to walk to the nearest station, set the walking speed to 5km/h. According to formula (9), the time cost of 2km service range is 24min. Set different driving speeds according to the road class. The set driving speed of expressways and arterial roads is 80km/h, of secondary roads is 60km/h, and of third-class roads is 45km/h. According to formula (9), calculate the time cost of each road and save the result in the time cost field. Calculate the service area range of the bus stations to get TAI, as shown in Fig 13.

Recreation Index (RI) is based on the distribution of scenic spots and parks in the city to analyze the service convenience degree of parks or spots [37, 38]. The research uses the number of scenic spots and the service range of each scenic spot to measure the recreation suitability of human settlement environment. According to the characteristics of Qingdao, the number of scenic spots and the weight of the service range are respectively 0.4 and 0.6, and the calculation formula is as follows:

$$RI=0.4*{Sum}_{spot}+0.6*\bar{s}$$ (10)

**Fig 14:**
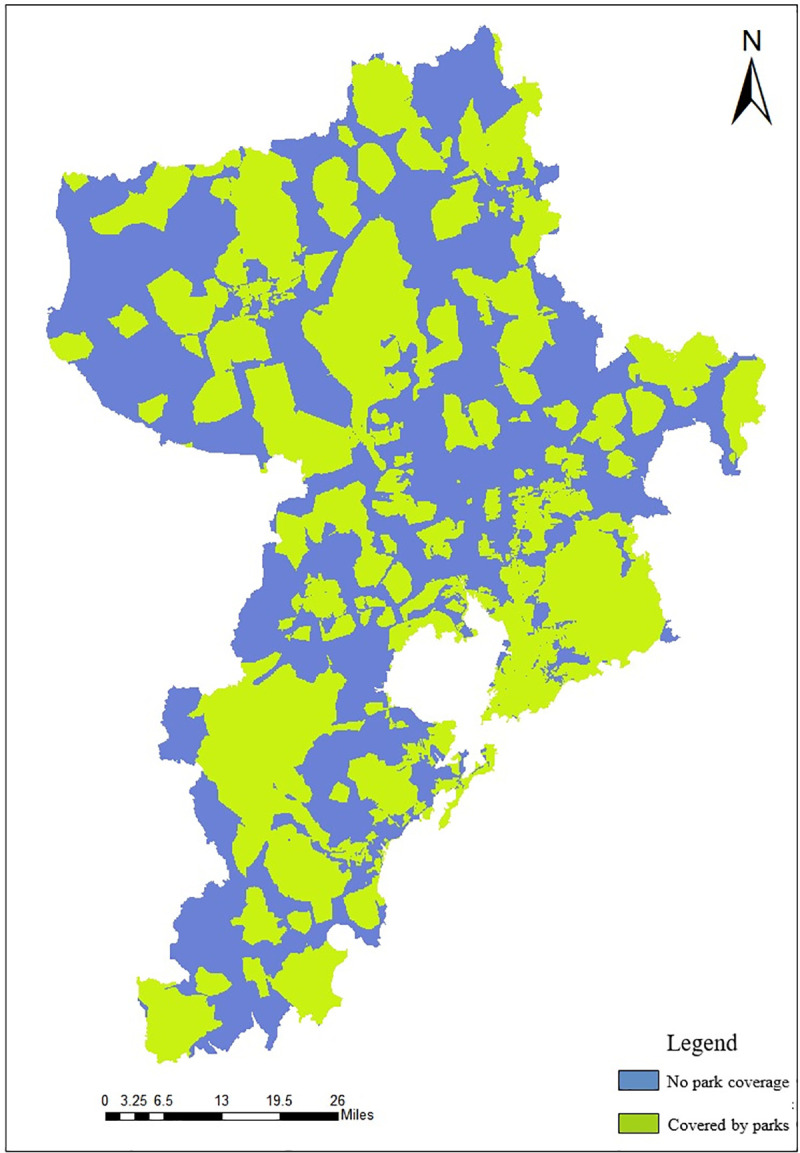
Distribution map of RI in Qingdao.

In the formula, *Sum*_*spot*_ is the normalized index of scenic spots, $\bar{s}$ is the average service range of scenic spots. The time cost can be calculated by formula (9), and then the average service range can be calculated. The calculation structure of the defined RI index is shown in Fig 14.

#### Index standardization

Because of the different sources and formats of each index, it is difficult to make a comprehensive analysis. Thus the de-dimensionalization processing is carried out first. The normalization method is used to standardize the data of 10 indexes. Different standardized formulas are selected for different index types. The minimum effect standardization can be used for the reverse indexes such as terrain, wind efficiency and air quality, while the other indexes are positive indexes, and the maximum effect standardization can be used for them. The calculation formula of maximum effect standardization is as follows:

Iscorei=(Xi−minX)/(maxX−minX) (11)

The calculation formula of minimum effect standardization is as follows:

Iscorei=(maxX−Xi)/(maxX−minX) (12)

In the formula, *Iscorei* represents the normalized value of index i, *maxX* and *minX* respectively represent the maximum and minimum of the initial value of index i, and *Xi* represents the initial value of index i.

#### Index weight calculation

The research uses Principal Component Analysis (PCA) to calculate the weight of human settlement environment index in Qingdao. Merge the bands of a series of index layers after normalization, and then use PCA method to get the eigenvalue of each index, as shown in Table 4.

**Table 4:** PCA Eigenvalues of suitability evaluation indexes.

Principal component	Eigenvalues	Eigenvalue percentage (%)	Cumulative variance contribution rate (%)
1	0.5752	77.61	77.61
2	0.0780	10.52	88.13
3	0.0387	5.21	93.34
4	0.0174	2.35	95.69
5	0.0136	1.84	97.53
6	0.0110	1.48	99.01
7	0.0056	0.74	99.75
8	0.0017	0.23	99.98
9	0.0010	0.01	99.99
10	0.0000	0.01	100.00

Use the linear correlation coefficient and the percentage of principal component eigenvalues to calculate the weight of urban human settlement environment indexes, the results are shown in Table 5.


$$Hj=\sum_{j=1}^{m}\varphi_{jk}^{2}(j=1,2,\ldots ,10;k=1,2,\ldots ,m)$$ (13)

**Table 5:** Weight of evaluation index for suitability of human settlement environment.

Index type	RDLS	NDVI	MNDWI	THI	WEI	AQI	Nighttime light index	NDISI	TAI	RI
Index weight	0.08	0.11	0.07	0.16	0.1	0.12	0.08	0.18	0.03	0.06

#### Extraction of built-up areas

Urban built-up areas mainly refer to the range of urban lands that has been constructed and developed, including urban impervious surface, urban parks, greening areas, and industrial and mining lands, etc. The extraction of built-up areas is mainly determined by extracting the urban impervious surface. This research uses Biophysical Composition Index (BCI) method to extract the urban impervious surface, and its calculation formula is as follows:

$$BCI=\frac{\frac{H+L}{2}-V}{\frac{H+L}{2}+V}$$ (14)

$$H=\frac{TC1-{TC1}_{min}}{{TC1}_{max}-{TC1}_{min}},V=\frac{TC2-{TC2}_{min}}{{TC2}_{max}-{TC2}_{min}},L=\frac{TC3-{TC3}_{min}}{{TC3}_{max}-{TC3}_{min}}$$ (15)

Among them, *TCi* (i = 1, 2, 3) is the first three components obtained by tasseled cap transformation of RS data, and *TCi*_*min*_ and *TCi*_*max*_ (i = 1, 2, 3) are respectively the minimum and maximum values of the i-th component. *H* represents high albedo, *L* represents low albedo and *V* represents vegetation.

**Fig 15:**
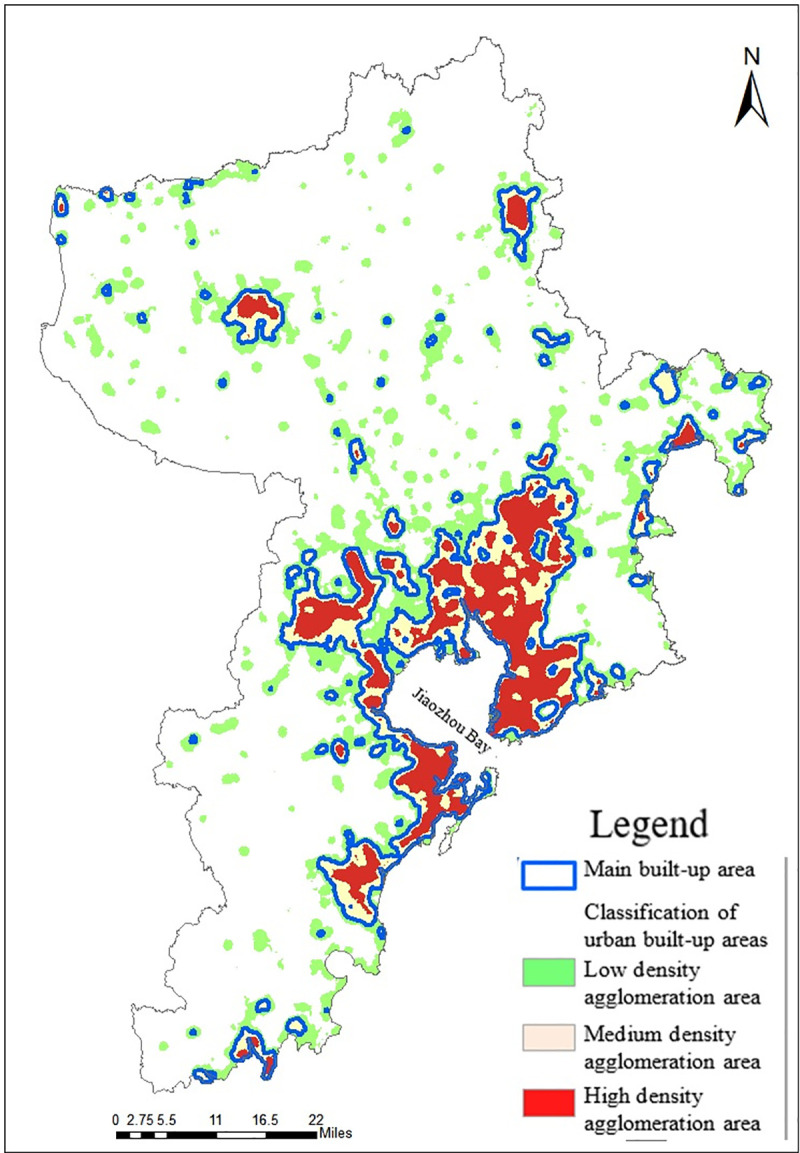
Distribution map of main built-up areas of Qingdao.

This research uses City Clustering Algorithm (CCA) method to extract the range of main urban built-up areas, this method is based on the aggregation density of impervious surface to divide the cluster areas. The aggregation distance is set as 1000m to calculate and obtain the aggregation density of impervious surface, and it is divided into low, medium and high density cluster areas. Its medium and high density cluster areas are selected as the extraction range of main urban built-up areas, as shown in Fig 15.

#### Comprehensive evaluation of suitability

**Fig 16:**
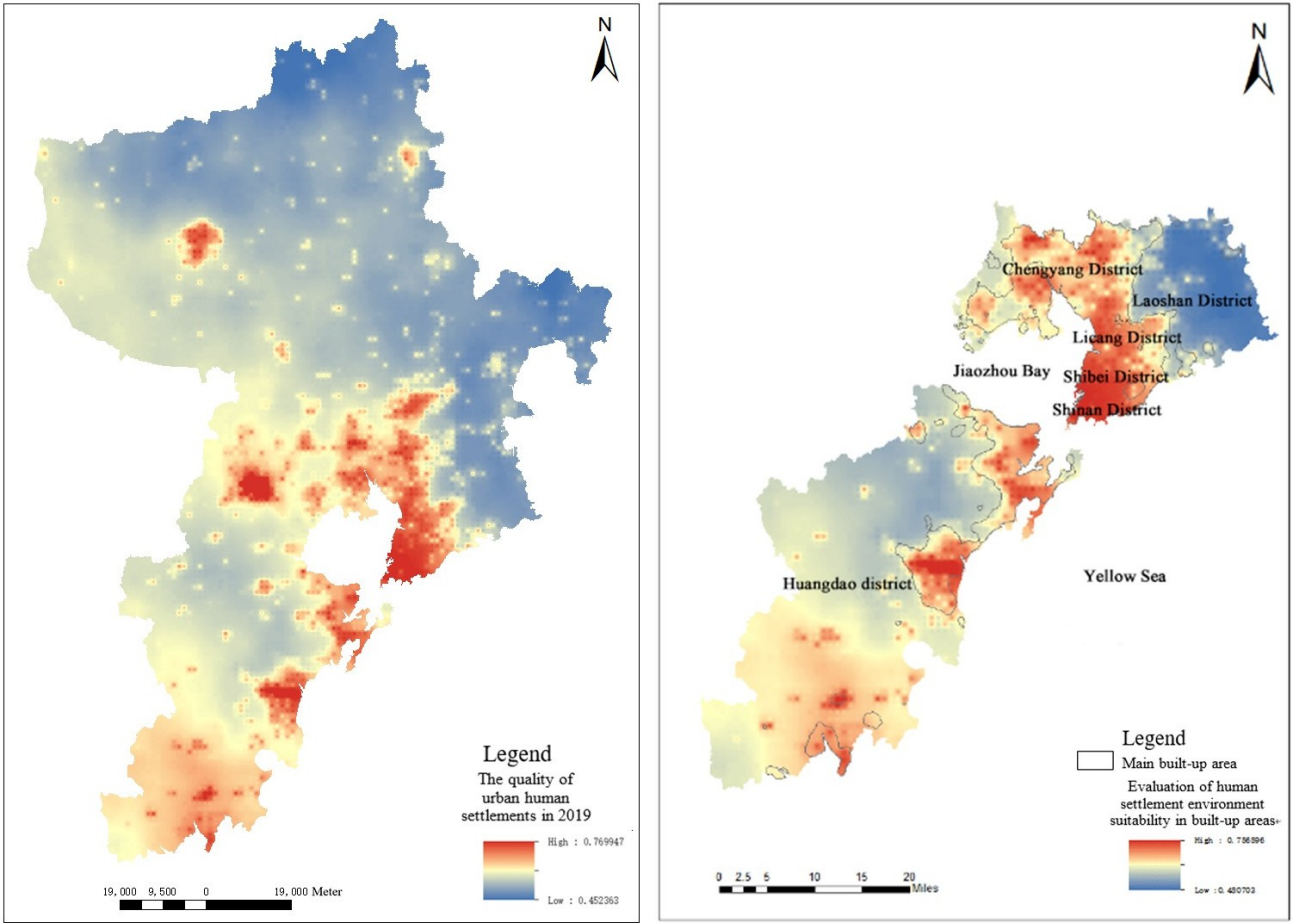
Comprehensive evaluation results of suitability of human settlement environment in Qingdao.

After the weight of each urban human settlement environment index is calculated, the weighted analysis of each index is carried out according to the weight value, and the comprehensive evaluation results are obtained, as shown in the left figure of Fig 16. The quantitative distribution of suitability of urban human settlement environment in regional space is represented by the quality values of human settlement environment. The quality values of human settlement environment in Qingdao are between 0.45 and 0.77. According to the analysis of the evaluation result map, it is concluded that the spatial distribution law of livability in Qingdao in 2019 is as follows: the livability distribution of Qingdao is decreasing from coastal cities to inland cities, and the distribution center inclines to the southwest of the city, while Jiaozhou Bay area has the highest and most significant livability. Taking Pingdu City and Laixi City as an example in the northern area, the phenomenon of mainly aggregation and scattered distribution appears in the area with high suitability of human settlement environment.

The built-up areas of Qingdao are mainly the downtown, Laoshan District, Chengyang District and Huangdao District. The evaluation results of suitability of human settlement environment are shown in the right figure of Fig 16. The quality values of human settlement environment in built-up areas are between 0.48 and 0.76. The quality of human settlement environment along the coast of Jiaozhou Bay and the coastal areas is relatively high. Due to the influence of topography, the quality of human settlement environment in most areas of Laoshan District and the central and western areas of Huangdao District is obviously lower than that of other areas. In addition, the quality of human settlement environment in the periphery of main built-up areas is lower than that inside built-up areas. The main reason is that the internal infrastructure construction in urban built-up areas is relatively mature relative to the urban periphery. Convenient transportation, developed economy, and comfortable coastal environment have a certain impact on the quality of human settlement environment.

#### Spatial autocorrelation analysis

*(1) Global spatial autocorrelation*. Global spatial autocorrelation can be used to describe the degree of spatial correlation and difference in the research area as a whole [39–41]. Moran’s I is used to determine the average correlation and significance for suitability of human settlement environment among each unit [42–45]. Moran index value is between -1 and 1, when the value is positive and closer to 1, the positive correlation is stronger and the average correlation of spatial similarity is stronger. When the value is negative and closer to -1, the negative correlation is stronger and the spatial heterogeneity is stronger. When the value is 0 or close to 0, there is no correlation in the space, or the correlation is smaller [18]. The calculation formulas are as follows:

$$I=\frac{\sum_{i=1}^{n}\sum_{j=1}^{n}\omega_{ij}(x_{i}-\bar{x})(x_{j}-\bar{x})}{S^{2}\sum_{i=1}^{n}\sum_{j=1}^{n}\omega_{ij}}\;(i\neq j)$$ (16)

$$S^{2}=\frac{1}{n}\sum_{i=1}^{n}{\left(x_{i}-\bar{x}\right)}^{2},\;\bar{x}=\frac{1}{n}\sum_{i=1}^{n}x_{i}$$ (17)

$$Z=\frac{I-E(I)}{\sqrt{VAR(I)}}$$ (18)

In the formula, *I* is the Moran index, *n* is the number of street units, *x*_*i*_ is the value of attribute x in the i-th unit, *x*_*j*_ is the value of attribute x in the j-th unit, *ω*_*ij*_ is the value of weight matrix, *E*(*I*) is average value, *VAR*(*I*) is variance, *Z* is standardized result.

*(2) Local Indicators of Spatial Association*. Local Indicators of Spatial Association (LISA) uses the global Moran index to divide block units to determine whether there are similarities or differences and the degree of significance in suitability of settlement environment in neighboring blocks in local areas [21]. The calculation formula is as follows:

$$I_{i}=\frac{n(x_{i}-\bar{x})\sum_{i=1}^{n}\omega_{ij}(x_{j}-\bar{x})}{\sum_{i=1}^{n}{\left(x_{i}-\bar{x}\right)}^{2}}\;(i\neq j),\;\bar{x}=\frac{1}{n}\sum_{i=1}^{n}x_{i}$$ (19)

In the formula, *I*_*i*_ is LISA index, *x*_*i*_ and *x*_*j*_ are the suitability index of human settlement environment in cities i and j, *n* is the number of cities, *ω*_*ij*_ is the spatial weight matrix. If *I*_*i*_ is positive, it indicates that suitability of human settlement environment in city i is close to neighboring cities. If *I*_*i*_ is negative, it indicates that the difference between suitability of human settlement environment in city i and that of neighboring cities is great.

*(3) Cold and hot spot analysis*. Cold and hot spot analysis can express the characteristics of cold and hot spots of spatial clustering in a certain area, which intuitively displays areas of hot spots and cold spots within the research area. The calculation formula is as follows:

$${G_{i}}^{*}=\frac{\sum_{j=1}^{n}w_{ij}x_{j}}{\sum_{j=1}^{n}x_{j}}\;(i\neq j)$$ (20)

In the formula: *x*_*j*_ is the quality of human settlement environment in city j, *w*_*ij*_ is the spatial weight matrix. In order to facilitate comparison and analysis, *G*_i_* is standardized, that is:

$$Z({G_{i}}^{*})=\frac{{G_{i}}^{*}-E({G_{i}}^{*})}{\sqrt{VAR({G_{i}}^{*})}}$$ (21)

In the formula, *Z*(*G*_i_*) represents standardized result of *G*_i_*, *E*(*G*_i_*) represents average value, *VAR*(*G*_i_*) is variance.

If *Z*(*G*_i_*) is positive and significant, it indicates that the suitability index value of human settlement environment in cities around city i is higher, and it belongs to the hot spot areas. On the contrary, if *Z*(*G*_i_*) is negative and significant, it belongs to the cold spot areas.

## Results and discussion

### Suitability grading

**Fig 17:**
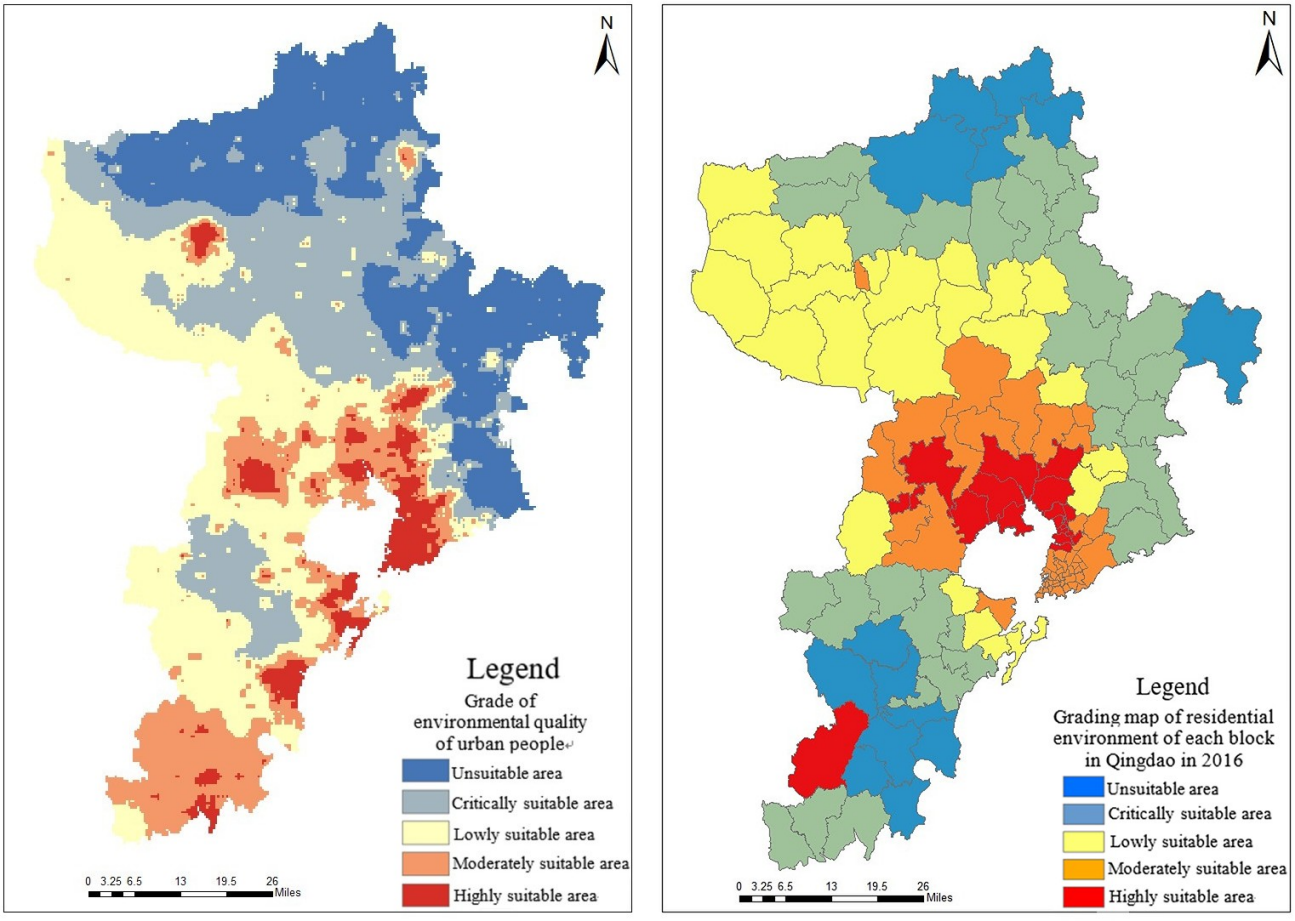
Evaluation grade for suitability of human settlement environment in Qingdao.

The natural discontinuity point method was selected to classify the quality values of human settlement environment in Qingdao, which was divided into 5 grades, as shown in the left figure of Fig 17. It can be seen from the figure that suitability of human settlement environment in Qingdao showed the following spatial distribution characteristics.

The human settlement environment index of the coastal areas in Qingdao was significantly higher than that of the inland areas. Jiaozhou Bay coastal areas had many trade ports and developed maritime trade. The central area in Qingdao had a high degree of urban modernization construction, with a large area of parks and historical sites, and was prosperous in material and culture. The built-up areas were mostly distributed in coastal areas, where the infrastructure construction was relatively perfect and the human livability was high. Therefore, suitability of human settlement environment in the coastal areas in Qingdao was relatively high.

The overall development of suitability of human settlement environment was not balanced. The downtown, west and southwest of Qingdao were overall better than the north and east of Qingdao. This was mainly due to the fact that the terrain in the east and north of Qingdao was more undulating than that in the west and southwest, and it had a certain relationship with the lower traffic accessibility. Affected by the mountainous terrain of Laoshan, the western part of Qingdao had flat terrain, low topographic relief and high suitability of urban human settlement environment.

Table 6 showed the proportion of each grade in the comprehensive evaluation for human settlement environment in Qingdao. The proportion of low suitable areas was the highest at 29.03%. These areas mainly concentrated in surrounding areas such as the junction of Pingdu and Laixi, most areas of Laoshan District, and the eastern part of Jimo District. The terrain in these areas was relatively undulating, the overall suitability of urban human settlement environment was not high, and the natural environment was quite different from the surrounding areas, which caused the areas to lose advantages in natural factors. However, compared with the western area in Qingdao, it also had certain regional advantages such as better air quality, and suitable ecological environment. Compared with urban built-up areas, the humanity livability was poor. Because these areas were located on the periphery of the city, with low traffic accessibility, and low overall level of urbanization, these areas were in the areas of low suitability.

**Table 6:** Proportion of comprehensive evaluation grade of human settlement environment in Qingdao.

Grade	Level 1	Level 2	Level 3	Level 4	Level 5
Rating index	0.45~0.51	0.51~0.55	0.55~0.59	0.59~0.65	0.65~0.77
Proportion of evaluation grades	23.66%	27.4%	29.03%	14.85%	5.06%

The proportion of high suitable areas was the least at 5.06%, mainly concentrated in the central part of Jiaozhou City, the south-eastern coastal areas of Huangdao District, the central part of Qingdao and most areas of Chengyang District. These areas were close to waters, with good accessible transportation, good air quality, and high quality of urban human settlement environment, making them the most suitable for living. The main reason that the highly suitable areas were less on the whole was restricted by natural conditions. Qingdao integrates a variety of urban functions, and there must be a variety of “urban diseases” inside, such as urban heat island effect and urban traffic congestion. The population density was relatively large and urban greening areas were limited, the range of suitable areas was limited to a few areas where urban development and ecological environment were coordinated.Therefore, the overall quality of human settlement environment was at a relatively low level.

Overall, the areas with suitable settlement environment in Qingdao were as high as 76%, which showed that most areas in Qingdao were suitable for long-term living and overall suitability of human settlement environment was relatively high.

The spatial distribution for the quality indexes of human settlement environment in Qingdao was analyzed from the perspective of urban blocks, as shown in the right figure of Fig 17. It can be seen from the figure that unsuitable areas were mainly distributed in Jiudian Town of Pingdu City, Nanshu Town, Rizhuang Town, Malianzhuang Town, Hetoudian Town of Laixi City, Tianheng Town of Jimo District and Liuwang Town, Baoshan Town, Tieshan Street, Zhangjialou Town, Zangnan Town, Langya Town, Binhai Street, Zhuhai Street of Huangdao District, a total of 14 blocks. Highly suitable areas were mainly distributed in Dacun Town of Huangdao District, Jiaodong Street, Fu’an Street, and Zhongyun Street of Jiaozhou City, Hetao Street, Hongdao Street, Chengyang Street, Jihongtan Street, Liuting Street, and Shangma Street of Chengyang District and Zhenhua Road Street, Yongqing Road Street, Yongan Road Street, Xinghua Road Street, Xingcheng Road Street, Licun Street, Xiangtan Road Street, Loushan Street of Licang District, a total of 18 blocks. Among them, the largest number of unsuitable areas was Huangdao District, accounting for 8. The largest number of highly suitable areas was Licang District, also accounting for 8.

### Spatial comparative analysis

**Fig 18:**
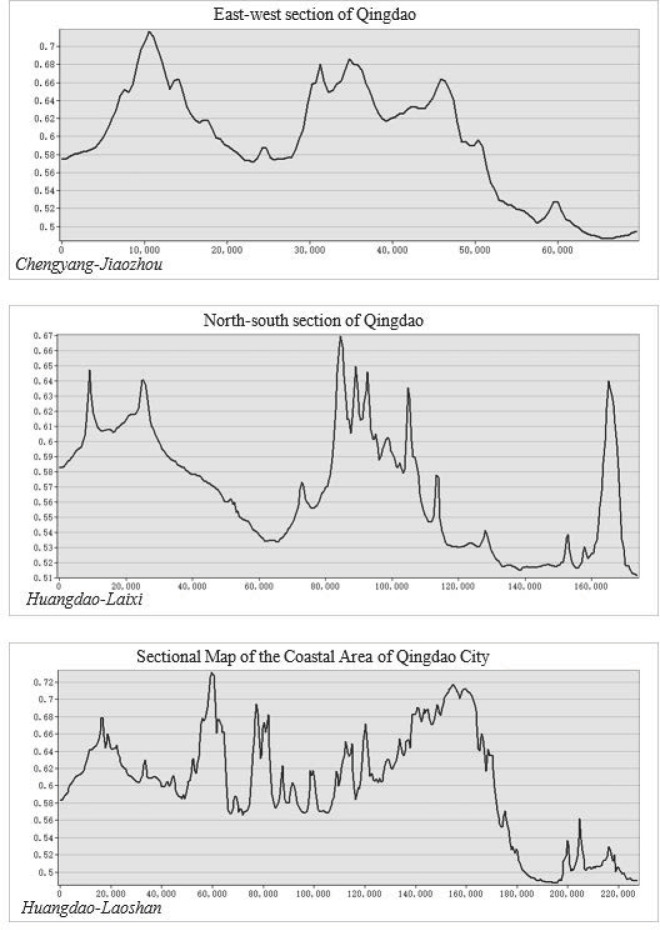
Profiles for quality of human settlement environment in Qingdao.

Taking the livability distribution of Qingdao as the analysis object, along with the general trend of Qingdao, the north-south and east-west extension lines were taken as the main lines of the city, and the coastline of the coastal areas was supplemented to draw the profiles of north-south axis, east-west axis and coastline for the urban environmental indexes. Among them, the axis of Chengyang-Jiaozhou direction was drawn across Jiaozhou City, Chengyang District and Laoshan District as the east-west axis of the city. The axis of Huangdao-Laixi direction was drawn across Huangdao District, Jiaozhou City and Jimo District as the north-south axis of the city. The axis of Huangdao-Laoshan direction was drawn across Huangdao District, Jiaozhou City, Chengyang District, Licang District, Shibei District, Shinan District, Laoshan District and Jimo District as the coastline axis of the city. The grid strip of 20000m*20000m was designated to draw profiles for the quality of urban human settlement environment, as shown in Fig 18.

By analyzing and comparing Fig 18, it can be found that the overall quality of human settlement environment in Qingdao presented a relatively obvious rule, mainly as follows: In the east-west profile, the quality distribution of human settlement environment was higher in the western area and lower in the eastern area. There were two main peak areas, which appeared in the central area of Jiaozhou City and most areas of Chengyang District. From the numerical point of view, Jiaozhou City was obviously higher than Chengyang District, while Laoshan District was decreasing in echelon form. In the north-south profile, the distribution of human settlement environment indexes was higher in the southern area and lower in the northern area. There were three main peak areas, which appeared in the south-west area of Huangdao District, most areas of Jiaozhou City, and the north-east area of Laixi City. From a numerical point of view, Jiaozhou City had the largest value and the suitability indexes of human settlement environment fluctuated greatly. In the coastline profile, the distribution of urban human settlement environment indexes was higher in the south-west area and lower in the north-east area, and the fluctuation degree for suitability of human settlement environment was larger in the southwest area. From the point of view of the value, the areas of Zhuhai Street and Yinzhu Street of Huangdao District had the highest suitability of human settlement environment, followed by the central area of Qingdao. While suitability of human settlement environment in Jimo District was generally low, and only in Hot Spring Street in the southern area of Jimo District, the suitability values of human settlement environment had improved. In summary, gravity center for higher suitability of human settlement environment in Qingdao was inclined to the central and western areas and the south-west area of the city, with unbalanced distribution and obvious local differences.

### Time change characteristics

**Fig 19:**
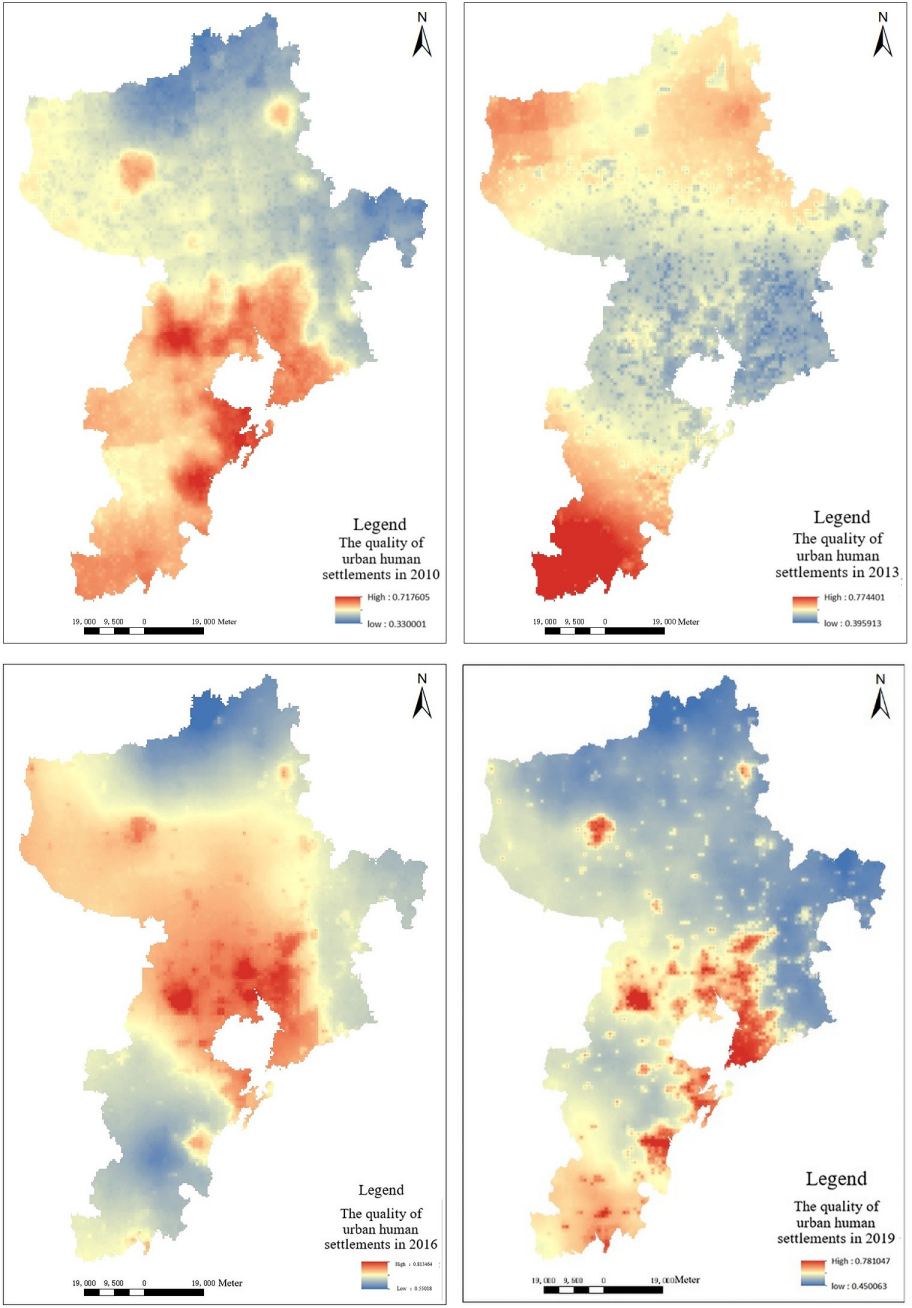
The quality of human settlement environment in Qingdao in 2010, 2013, 2016, 2019.

Use the method mentioned in Section Material and methods to evaluate the livability of human settlement environment in Qingdao in 2010, 2013, 2016 and 2019. The results were shown in Fig 19. It can be seen that: from 2010 to 2013, although the overall livability level of human settlement environment in Qingdao had improved, there were obvious regional differences compared with 2010. From 2013 to 2016, the livability of human settlement environment in Qingdao showed an upward trend, and the overall trend gradually improved. From 2016 to 2019, the changes of human settlement environment in Qingdao tended to be stable, and the overall trend slightly decreased.

From 2010 to 2013, the regional differences for the livability of human settlement environment in Qingdao were great. From the perspective of social production and economic activities, the main reasons were as follows: Qingdao accelerated urban construction, introduced high-quality innovative talents, and promoted the development of high-technology industries. The urban areas in Qingdao and the surrounding areas along Jiaozhou Bay etc. were the most notable. While the land space was being developed and utilized, the urban greening areas were decreased, the humanity livability was improved, and the natural livability was decreased, making the overall livability of human settlement environment in urban construction areas decreased, but relatively improved in the periphery of urban construction areas. Due to the different speed of urbanization between areas, there were obvious regional differences in human settlement environment.

From 2013 to 2016, human settlement environment in Qingdao had been improved, the overall livability of urban environment had rebounded, and showed an upward trend. The main reasons were as follows: the overall construction for main urban areas in Qingdao was basically completed, and the urban functional zoning was more obvious. The impact of urbanization on human settlement environment was gradually weakened, and the ecological environment in the city was gradually recovered. The perfect urban transportation network and supporting medical health education played a positive role in the restoration of human settlement environment, hence, the overall quality of human settlement environment was relatively high during this period.

From 2016 to 2019, the livability of human settlement environment in Qingdao showed a trend of stable development. During this period, urban construction of Qingdao was already at a relatively stable level, and infrastructure construction tended to be improved. Since Shanghai Cooperation Organization (SCO) summit, the greening areas of Qingdao and its surrounding areas had increased, and the vegetation index had improved overall. At the same time, the improvement of people’s consciousness of water landscape and governance and renewal work of urban water pollution had also caused the promotion of urban water landscape index, and urban human settlement environment maintained at a higher level.

**Fig 20:**
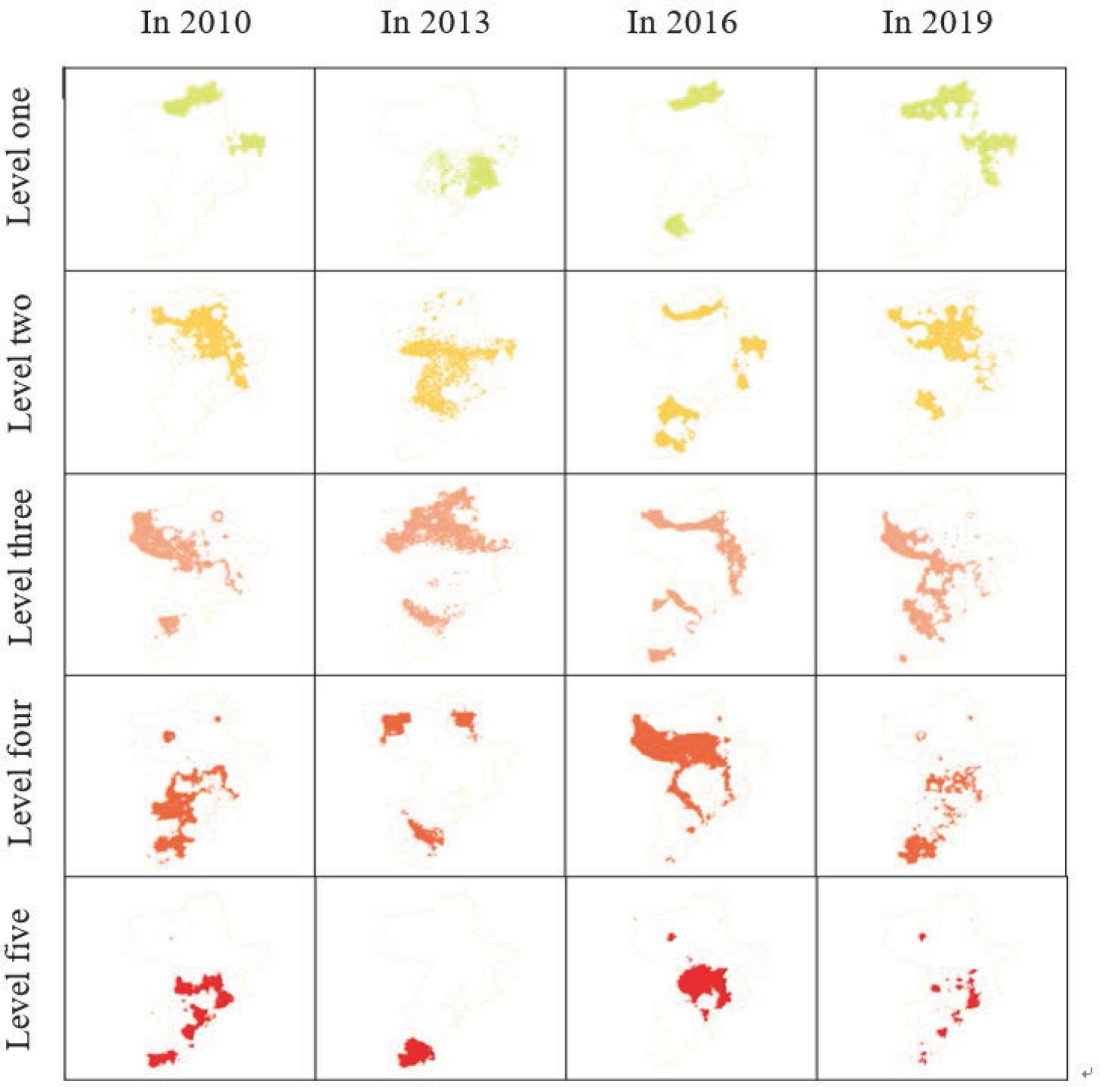
Hierarchical spatiotemporal change map of suitability of human settlement environment in Qingdao.

The evaluation data for suitability of urban human settlement environment in Qingdao from 2010 to 2019 was extracted and processed hierarchically, and hierarchical spatiotemporal change map was obtained for suitability of human settlement environment in Qingdao, as shown in Fig 20. It can more vividly and intuitively explain the spatial distribution trend of the temporal variation of human settlement environment.

### ESDA-GIS spatial analysis

Exploratory Spatial Data Analysis-Geographic Information System (ESDA-GIS) was used as the analysis mode. From the perspective of spatial autocorrelation and cold and hot spots analysis, explored the spatial agglomeration and differentiation of the quality of human settlement environment, and revealed the internal connection of spatial evolution.

#### (1) Global spatial autocorrelation

**Fig 21:**
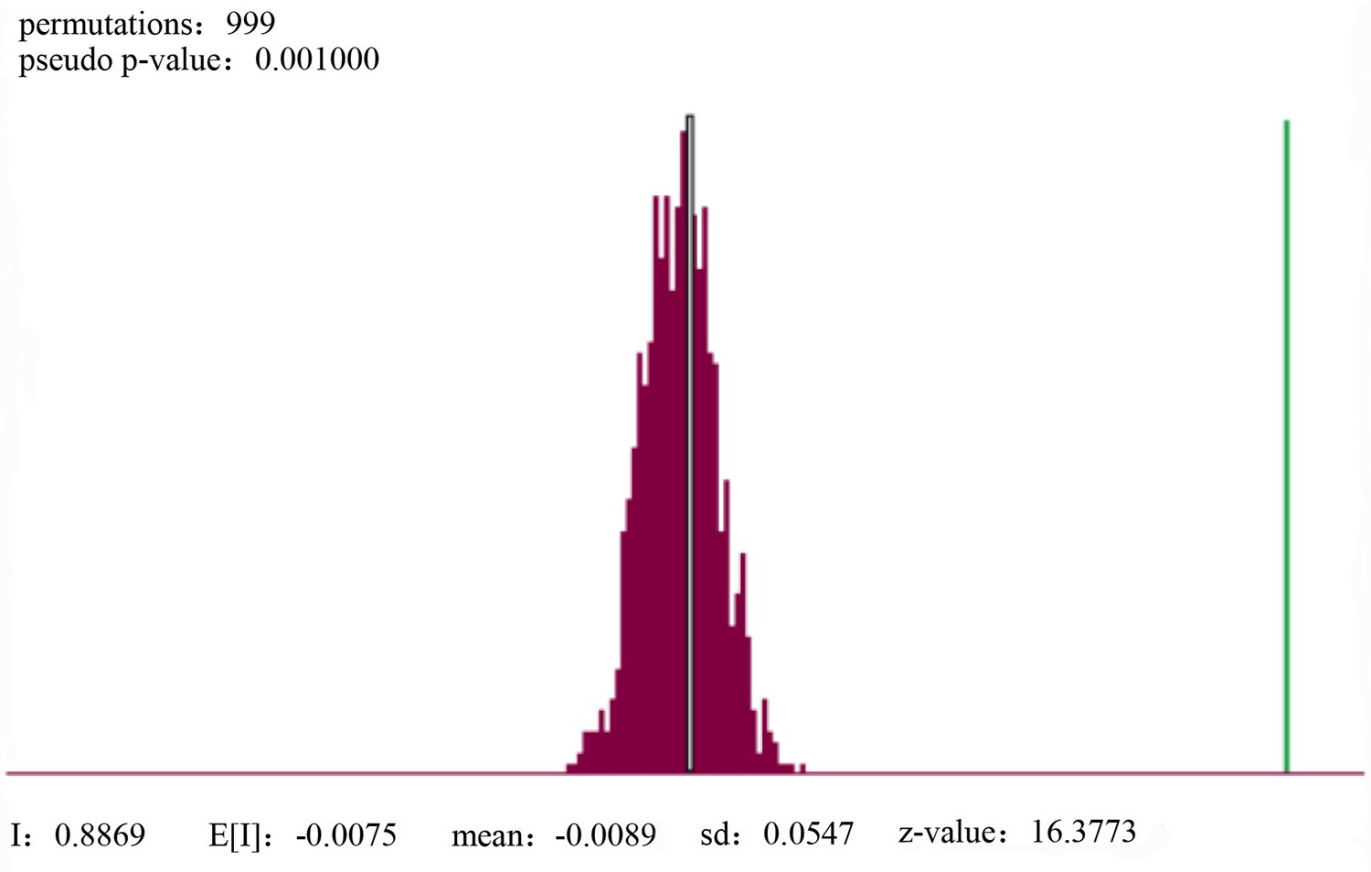
Moran index for quality of human settlement environment in Qingdao.

After the research area was divided according to street units, Moran’s I was calculated, and Fig 21 was obtained. The significance test showed that the value of Moran’s I was 0.8869 and reliability was high. It showed that the spatial distribution of the quality of human settlement environment had obvious correlation, and the quality was proportional to the aggregation degree.

#### (2) Local spatial autocorrelation

**Fig 22:**
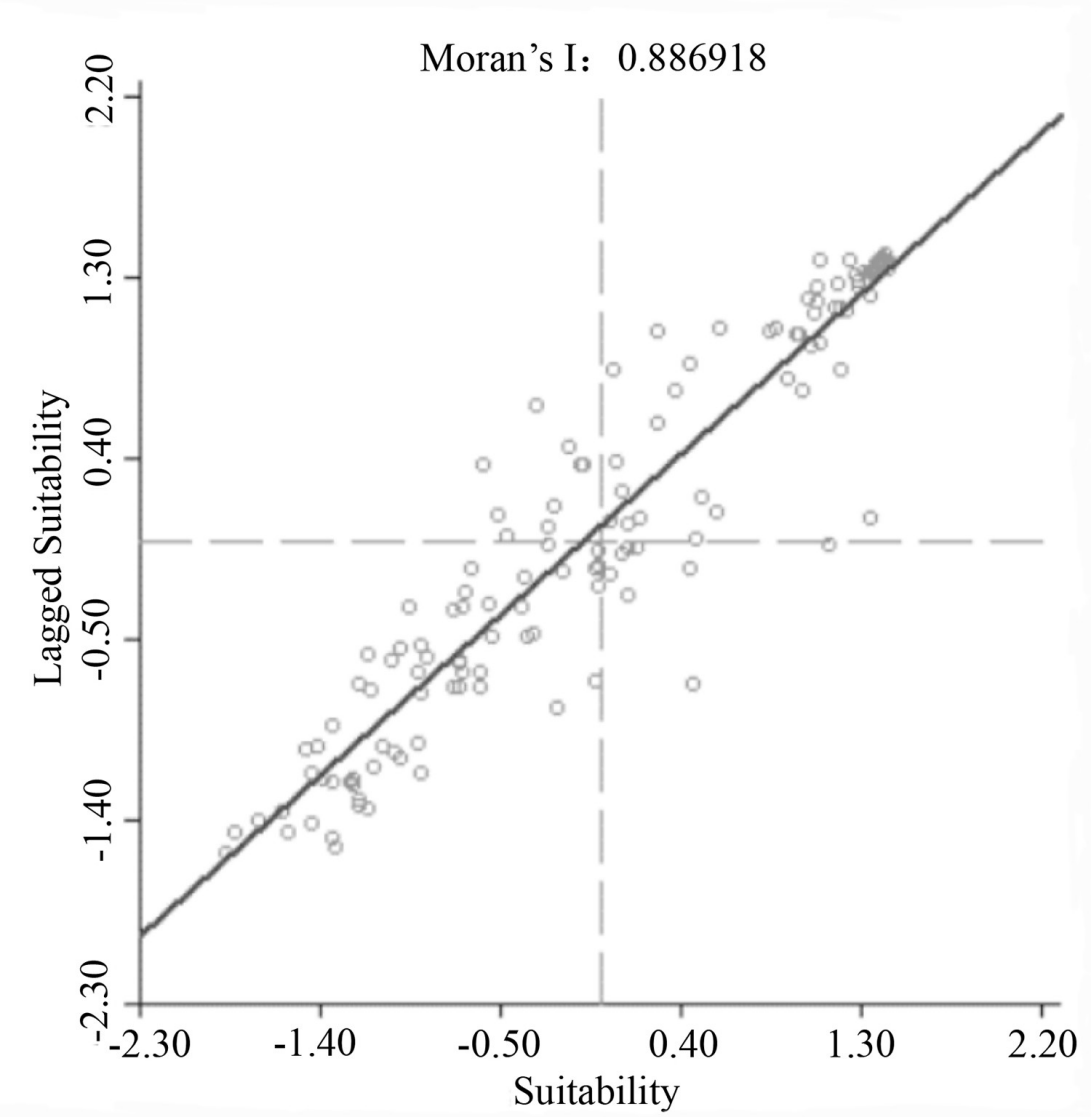
Moran scatterplot for quality of human settlement environment in Qingdao.

Moran scatterplot can reflect the correlation between suitability of urban human settlement environment and urban spatial lag variables, and can intuitively show local spatial correlation pattern [45]. Its abscissa was the suitability index value of urban human settlement environment, and its ordinate was the lagging suitability index value of urban human settlement environment. The four divided quadrants respectively correspond to four different spatial agglomeration states, among them, High-High agglomeration (HH) and Low-Low agglomeration (LL) indicated that the index value for suitability of urban human settlement environment was less different from that of surrounding cities and showed spatial agglomeration, on the contrary, LH and HL showed spatial heterogeneity. The Moran scatterplot for quality of urban human settlement environment in Qingdao is shown in Fig 22, it can be seen that Moran scatterplot in the first quadrant fits the fitted linear equation better than that of the fourth quadrant, which indicates the agglomeration degree of blocks with good quality of human settlement environment is significantly better than that of poor blocks.

Moran scatterplot cannot be used to judge from a statistical point of view whether the local correlation of block units and the aggregation area were meaningful [46–50]. Therefore, LISA were used to determine whether there were similarities or differences and the degree of significance of suitability of settlement environment of neighboring blocks.

**Fig 23:**
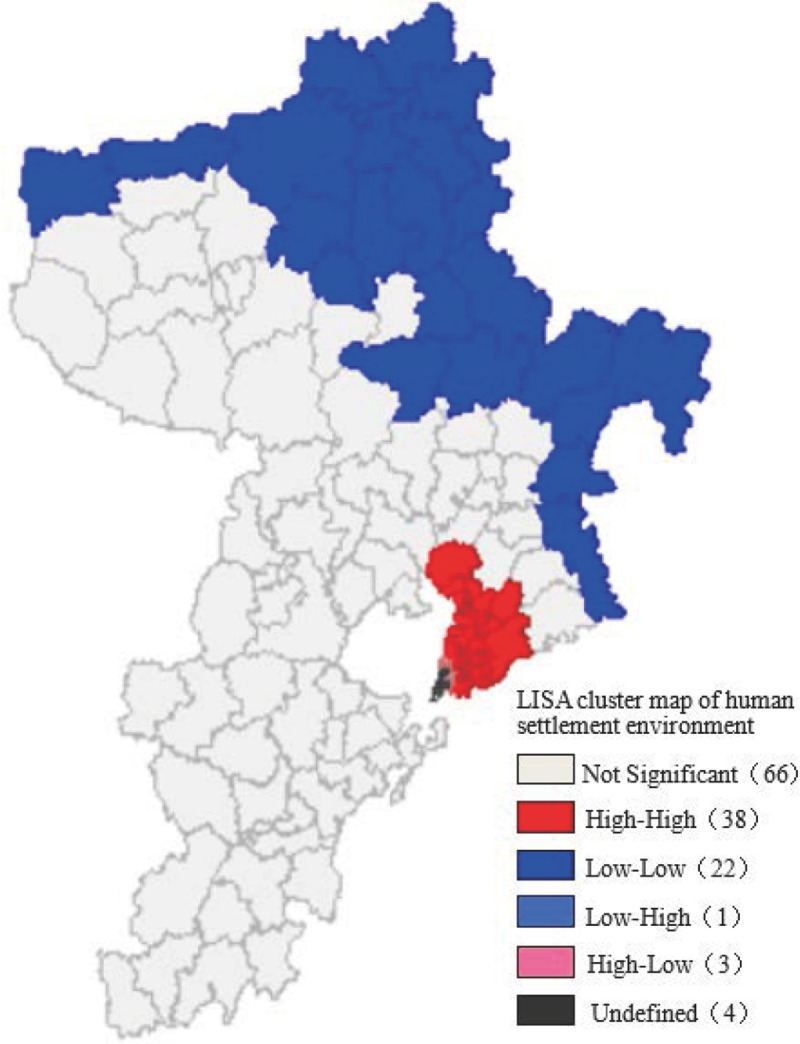
LISA cluster map for quality of human settlement environment in Qingdao.

Based on the spatial distribution data of each street unit in Qingdao, used formula (19) to obtain LISA cluster map of quality of human settlement environment in Qingdao, as shown in Fig 23. From statistical data of view, there were 68 significance areas of spatial agglomeration, accounting for 50.75% of the total, in which HH aggregation areas accounted for 55.88% and LL aggregation areas accounted for 32.35%. From spatial distribution of view, HH aggregation areas were distributed in most of South District, Shibei District, Licang District in Qingdao and Liuting Street of Chengyang District, that is, they were mainly distributed in the eastern coastal areas of Jiaozhou Bay and the near areas for Qingdao City center, LL significance aggregation areas were concentrated in the agglomeration areas formed in the northeastern part of Qingdao, that is, the northern part of Pingdu City, most of Laixi City, the eastern and northern areas of Jimo District, and the eastern areas of Laoshan District. Insignificant aggregation area had a wide range, covering most of 6 urban areas. HH aggregation areas were concentrated in the city center, while LL aggregation areas were distributed in the periphery of the city, showing a patchy distribution.

#### (3) Cold and hot spot analysis

**Fig 24:**
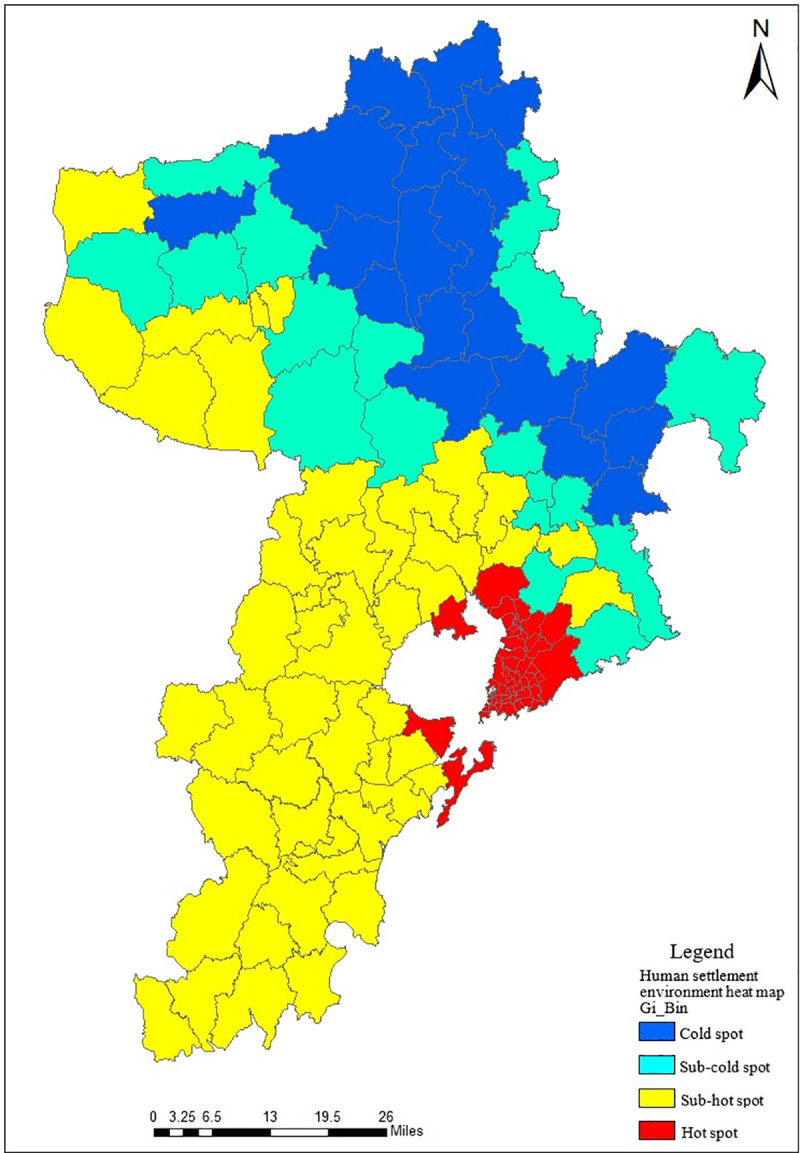
Distribution map of cold and hot spots of human settlement environment in Qingdao.

Using the cold and hot spot analysis method in Section Data analysis, the research analyzed and generated the distribution map of cold and hot spots of human settlement environment in Qingdao, as shown in Fig 24. Hot spot areas of urban human settlement environment were concentrated in the coastal areas of Jiaozhou Bay. The Laoshan area in the east of downtown and most area of Jimo, Laixi and Pingdu were the cold spot areas of human settlement environment. The main reason was that these areas had large topographic relief, low traffic accessibility and low greening degree, which made the level of human activity lower than that of other areas, thus forming cold spot areas.

## Conclusion and suggestion

### Conclusion

The quality evaluation of urban human settlement environment based on raster data has important research and application value. The research selects Qingdao as the research area for suitability evaluation of human settlement environment, used ESDA-GIS analytical mode to analyze and process the evaluation results, evaluated comprehensively human settlement environment in Qingdao, and obtained the following conclusions:

1. Suitability of human settlement environment in Qingdao is well developed, showing the characteristics of multi-center radiation driving development. However, the overall distribution is uneven, decreasing from the coastline to the inland. The southwest and other areas close to the city center are better than the northeast. The development mode of human settlement environment is mainly natural livability, supplemented by humanity livability, and the main driving factors are natural elements.
2. Through exploratory spatial analysis, it is found that the quality of human settlement environment has obvious spatial correlation and is positively correlated with the degree of aggregation. The agglomeration of blocks with a higher level of human settlement environment quality is higher than that of blocks with a lower level. The hot spot areas of human settlement environment are concentrated in Jiaozhou Bay along the coast, close to the city center, while the areas outside built-up areas such as Jimo and Laixi are cold spot areas.
3. The changes of human settlement environment in Qingdao are that: from 2010 to 2013, the areas with high quality of urban human settlement environment began to shift from the city center to the north and the south, the natural livability of built-up areas declined, and the humane livability increased. From 2013 to 2016, the quality of human settlement environment of the central area in Qingdao showed a phenomenon of recovery. The development pattern of the tertiary industry represented by the tourism industry had basically taken shape, and the tourist scenic areas along the coast had formed a high suitability of human settlement environment. From 2016 to 2019, the development mode of human settlement environment in Qingdao had been transformed into multi-point development and the environmental suitability had been improved as a whole.

According to the comprehensive evaluation model of suitability of human settlement environment in Qingdao, the development mode of suitability of human settlement environment in Qingdao takes natural livability as the main, and human livability as the auxiliary. It is mainly manifested in the high level of urban construction and good natural element indicators in coastal landscape areas such as the southern coastal area of Qingdao and the coastal areas of Jiaozhou Bay, which is a representative of the development of human settlement environment in Qingdao. The analysis of its driving factors mainly includes the following points:

1. The first is the development of tourism. As a coastal city, Qingdao is rich in tourism resources, and the development of tourism has attracted many investors from domestic and overseas, policies implementation and the investment in the commercial sector have driven the development of the regional economy, in which the natural environment plays a major role.
2. The second is the construction of university town. In recent years, Qingdao has introduced a number of well-known universities, including provincial key universities and double top universities and institutions etc. The universities stationed in Qingdao give full play to the respective discipline advantages to provide supports for economic construction of Qingdao in terms of personnel training and scientific research cooperation, and make great contributions to urban development.
3. The last is the improvement of the transportation network system. In order to ensure the sustainable development of urban transportation, Qingdao has built a comprehensive transportation system that meets the requirements of urban development. The construction of transportation network and public supporting facilities are in a new stage of rapid development, and suitability of humanity environment is also at a higher level of development.

For other areas in Qingdao, the development of human settlement environment is not optimistic. These areas lack relatively rich tourism resources, and the construction of natural landscapes and urban humanistic environment lags behind built-up areas along the coast, and there is no pillar industry to drive development, resulting in lower overall quality of human settlement environment. These areas are mainly distributed in the inland areas in the north of Qingdao, such as the north-eastern areas of Pingdu City and the western areas of Laixi City.

### Suggestion

In view of the current situation and the comprehensive evaluation results of suitability of human settlement environment in Qingdao, relevant departments should adapt to local conditions in the process of urban planning and construction, and formulate different development routes according to the characteristics of human settlement environment in different regions. Therefore, based on the research conclusions, put forward the following suggestions:

1. Build an ecological city and develop an ecological economy. For cities with a higher degree of greening and a better overall environment, it is necessary to develop an ecological economy while protecting ecological civilization, and make full use of local tourism resources and strive to develop into a natural and livable city. During the process, attentions should be paid to protecting the biodiversity along the western coastal wetlands.
2. Speed up the construction of pillar industries and adjust the industrial structure. For areas with low level of public infrastructure construction and relatively backward overall, it is necessary to dig deeper into pillar industries with local characteristics, and drive the city construction by mineral resources, water resources, agriculture, forestry, animal husbandry, fishery and other natural resources. Development projects around the northern and eastern districts should be suggested despite the topographic defect in order to decongest the stress on other districts.
3. Promote the quality of residence and improve the structure of residence. Combine the function and structure of residence with housing price issues as evaluation standard for healthy development of the real estate industry, ensure the rationality of division of residential functional spaces, and strictly control housing prices. In addition, it is necessary to accelerate the construction of talent housing and provide talent supports for the construction of opening and sharing international metropolis.
4. Improve the rural living environment and implement the rural revitalization strategy. Strengthen the construction of rural relevant infrastructure and guide the formation of a green lifestyle and life philosophy. In the critical period of decisive victory in building a well-off society in an all-round way and poverty alleviation, steadily promote various work of rural human settlement environment to make the countryside more beautiful and livable, and improve people’s overall happiness.

In a word, the research constructed a multi-index model to comprehensively evaluate suitability of human settlement environment in Qingdao, and the spatial and temporal distribution of different levels of livability in Qingdao was obtained. Moreover, the driving mechanism behind it is discovered, and based on this, some suggestions are put forward to improve the quality of human settlement environment in Qingdao. Future research can focus on deeply exploring the driving mechanism of suitability of human settlement environment in Qingdao.

## Supporting information

S1 File (ZIP)
