# Supplementary material for: Evaluation and analysis on suitability of human settlement environment in Qingdao
Source: PLoS One. 2021 Sep 27;16(9):e0256502. doi: 10.1371/journal.pone.0256502 (PMC8476008; doi:10.1371/journal.pone.0256502)
Supplement: S1 File — (ZIP) [file pone.0256502.s001.zip › Accessories for human settlements calculation/Yearbook data/2019─Ω╝°.pdf]

2019
青岛统计年鉴
QINGDAO STATISTICAL YEARBOOK

青岛市统计局
国家统计局青岛调查队 编
QINGDAO MUNICIPAL STATISTICS BUREAU
NBS SURVEY OFFICE IN QINGDAO

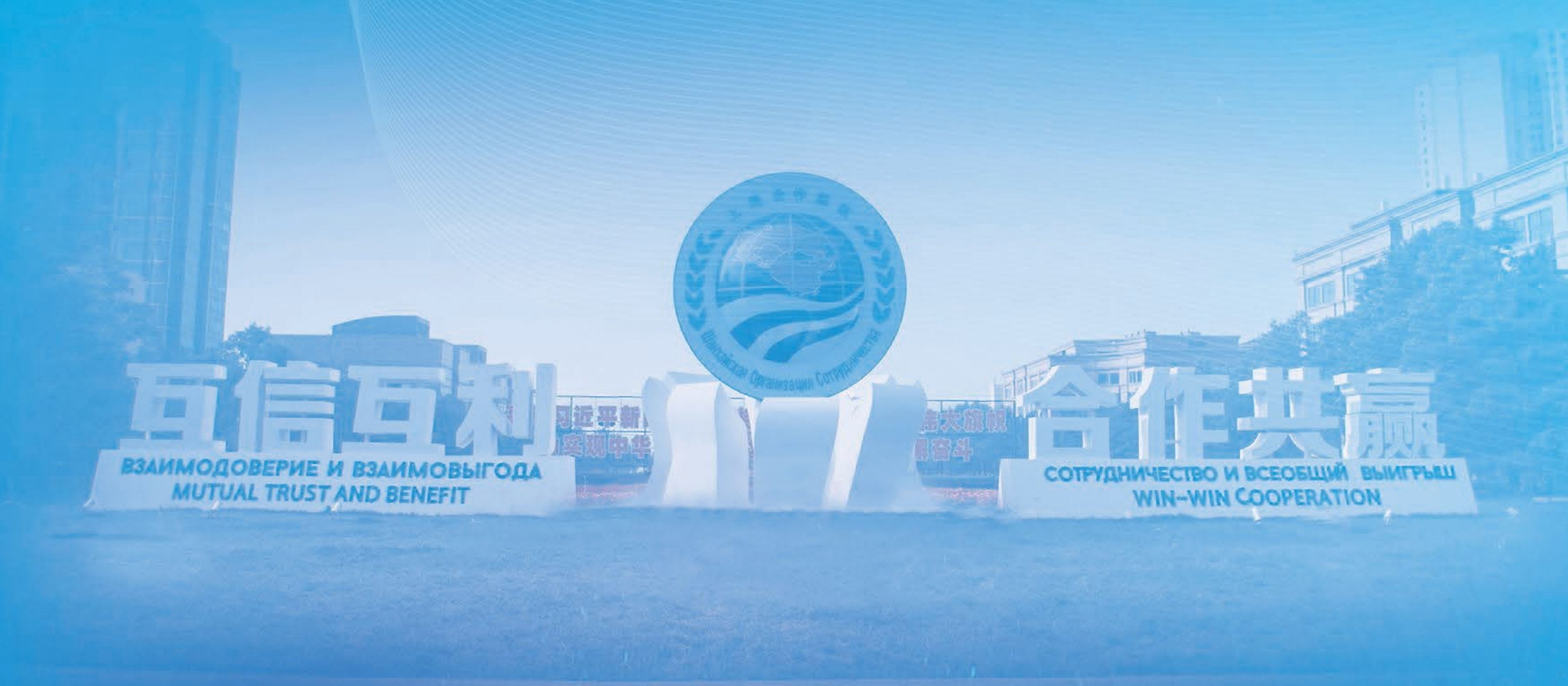

中国统计出版社
China Statistics Press

2019 青岛统计年鉴 QINGDAO STATISTICAL YEARBOOK

中国统计出版社 China Statistics Press

2019 青岛统计年鉴
QINGDAO STATISTICAL YEARBOOK

ISBN 978-7-5037-8827-7
9 787503 788277
定价：280.00元

2019
青岛统计年鉴

QINGDAO STATISTICAL YEARBOOK

（总 第40期 VOL.40）

青 岛 市 统 计 局
国家统计局青岛调查队 编
QINGDAO MUNICIPAL STATISTICS BUREAU
NBS SURVEY OFFICE IN QINGDAO

中国统计出版社
China Statistics Press

© 中国统计出版社有限公司 2019
版权所有。未经许可，本书的任何部分不得以任何方式在世界任何地区以任何文字翻印、拷贝、仿制或转载。

© 2019 China Statistics Press Co.,Ltd.
All rights reserved. No part of the publication may be reproduced or transmitted in any form or by any means, electronic or mechanical, including photocopying, recording, or any information storage and retrieval system, without written permission from the publisher.

图书在版编目（CIP）数据

青岛统计年鉴 . 2019 / 青岛市统计局，国家统计局青岛调查队编 . -- 北京：中国统计出版社，2019.7
ISBN 978-7-5037-8827-7

Ⅰ . ①青… Ⅱ . ①青… ②国… Ⅲ . ①统计资料－青岛－ 2019 －年鉴 Ⅳ . ① C832.523-54

中国版本图书馆 CIP 数据核字 (2019) 第 122109 号

青岛统计年鉴 –2019

作　　者 / 青岛市统计局　国家统计局青岛调查队
责任编辑 / 钟　钰
装帧设计 / 青岛双福文化传播有限公司
出版发行 / 中国统计出版社有限公司
地　　址 / 北京市丰台区西三环南路甲 6 号
邮政编码 / 100073
电　　话 / 邮购（010）63376909　书店（010）68783171
网　　址 / http://csp.stats.gov.cn
印　　刷 / 青岛任缘印刷包装有限公司
经　　销 / 新华书店
开　　本 / 890mm × 1240mm　1/16
字　　数 / 1130 千字
印　　张 / 26.375
版　　别 / 2019 年 7 月第 1 版
版　　次 / 2019 年 7 月第 1 次印刷
定　　价 / 280.00 元

如有印装差错，由本社发行部调换。

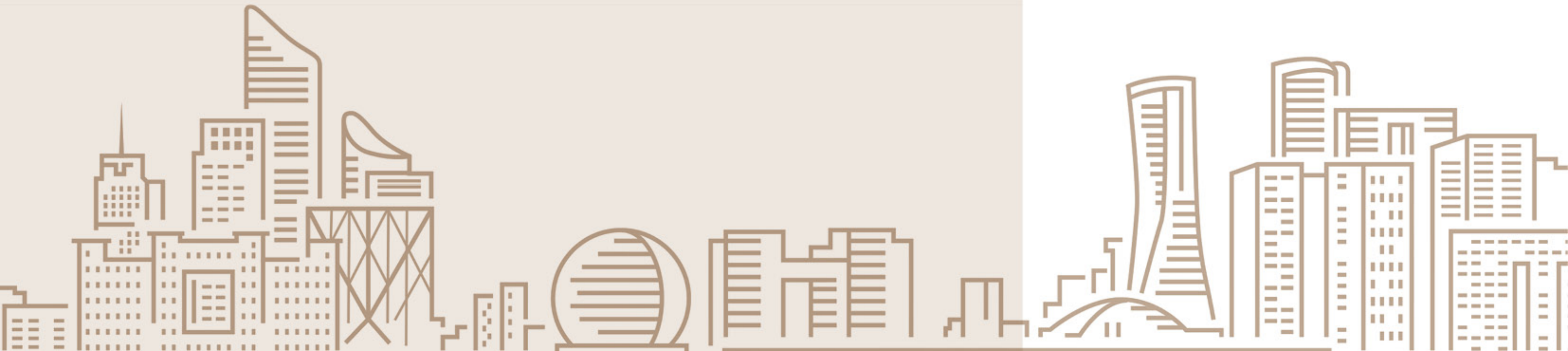

全市生产总值构成（%）

Composition Of Gross Domestic Product（%）

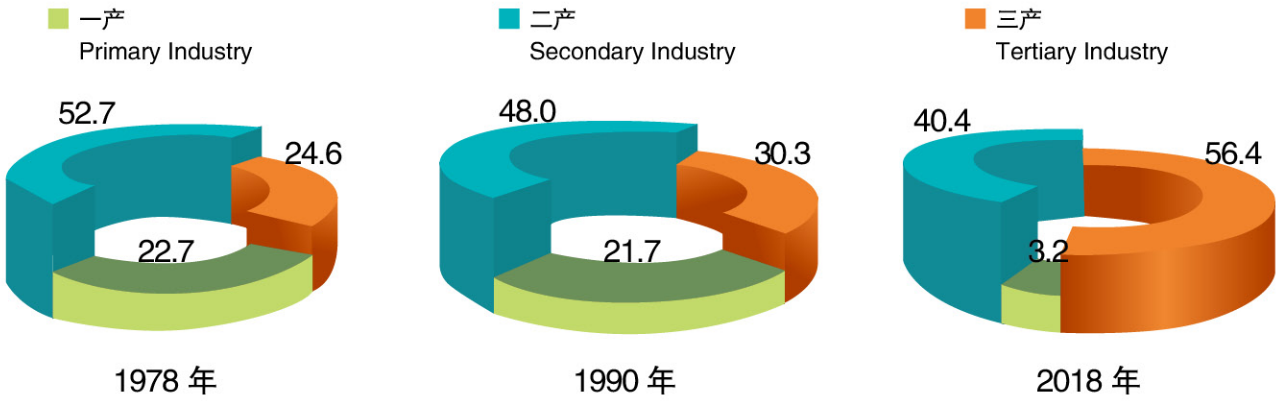

全市生产总值（亿元）

Gross Domestic Product（100 million yuan）

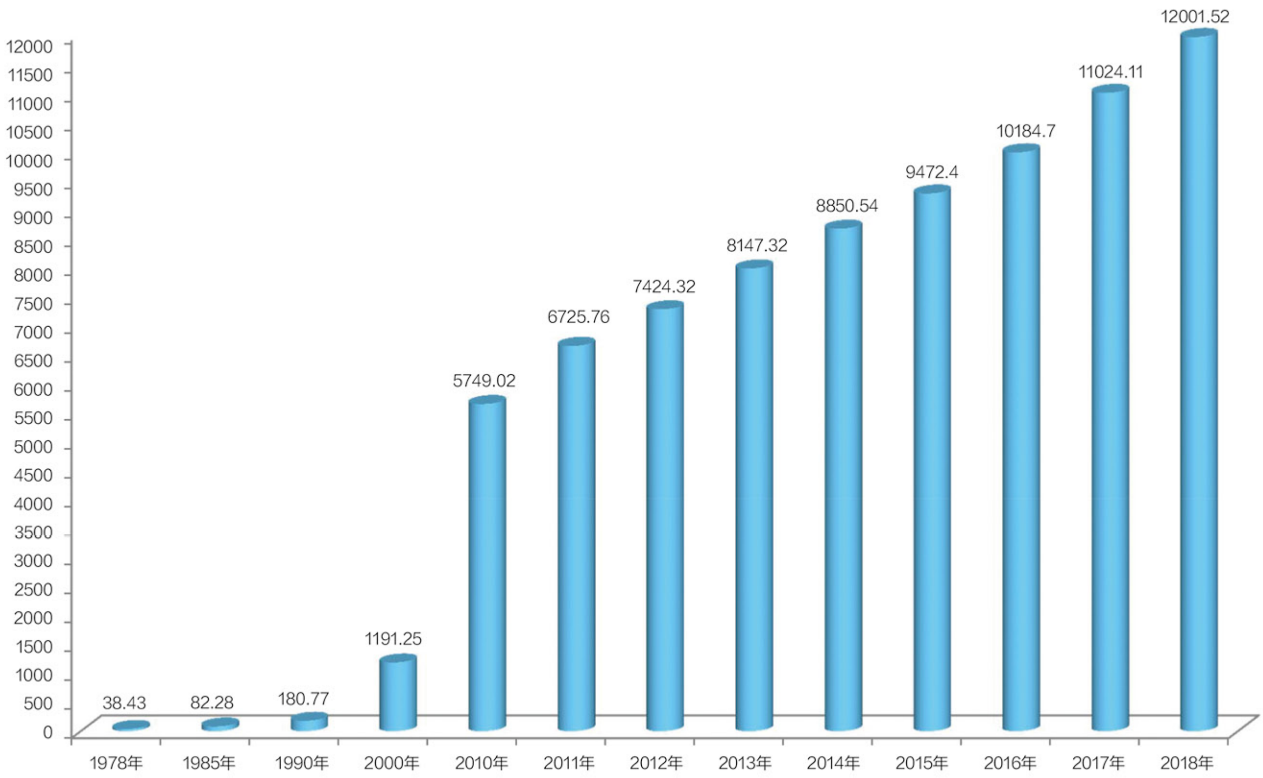

农林牧渔业总产值（亿元）

Gross Output Value Of Agriculture（100 million yuan）

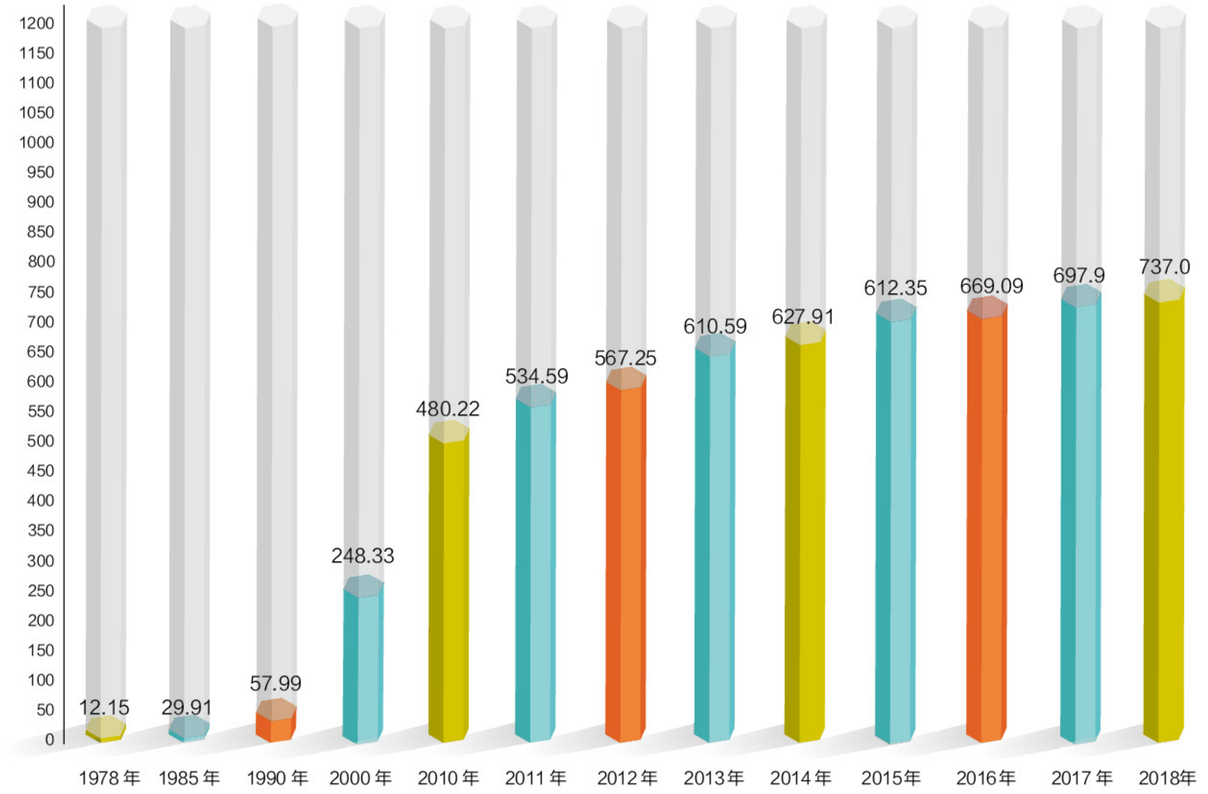

主要农产品产量（万吨）

Output Of Farm Products（10 000 tons）

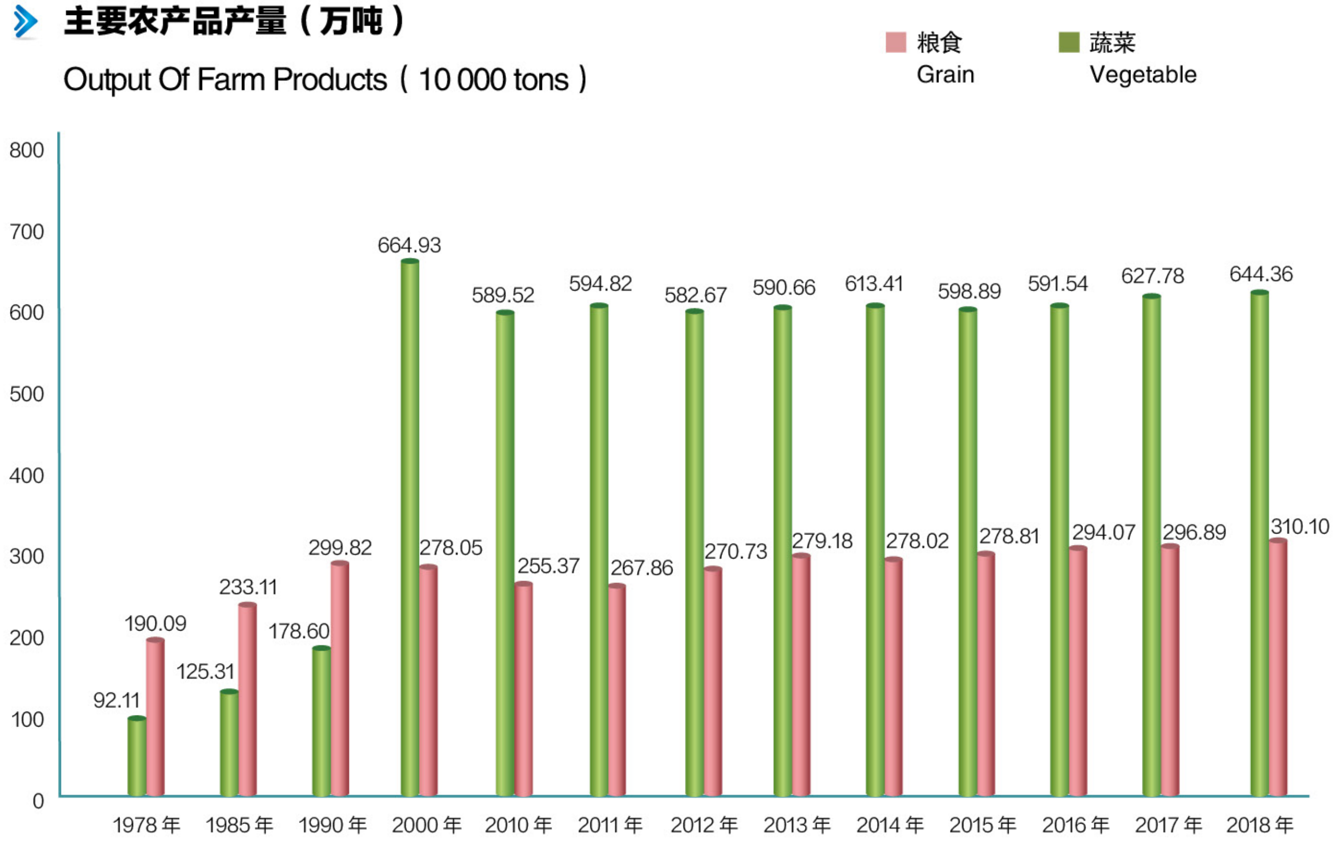

工业总产值（亿元）

Gross Industrial Output Value（100 million yuan）

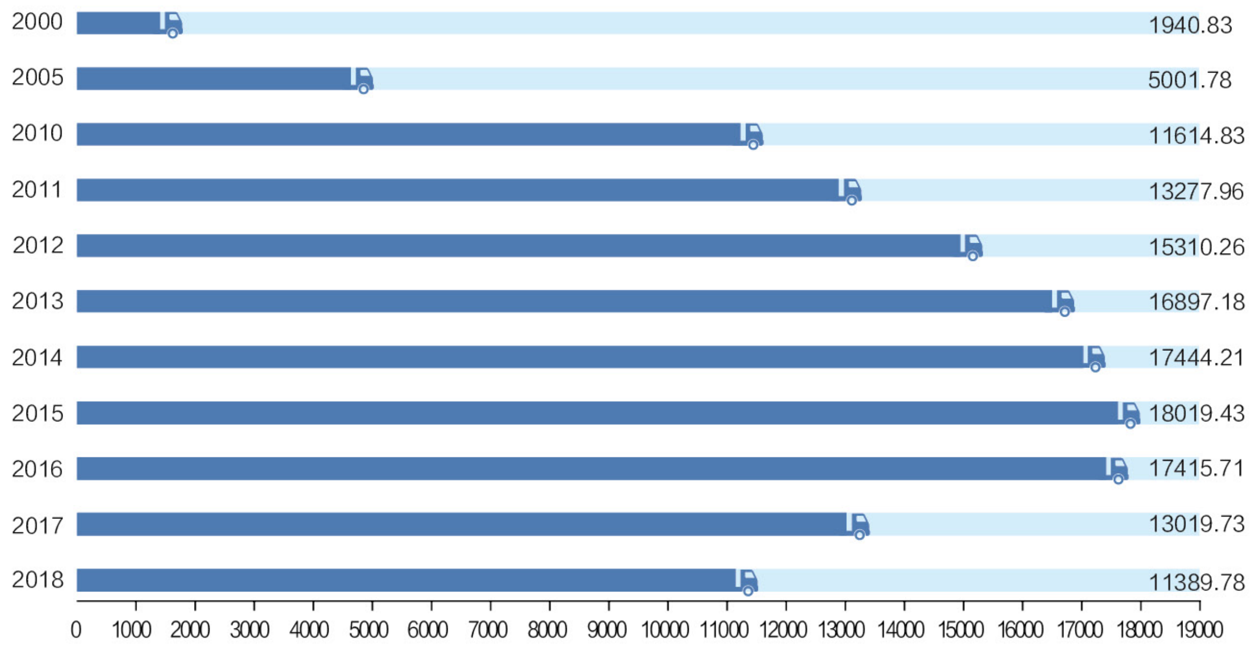

规模以上工业实现利润总额（亿元）

The Total Profits Made By The Industries Above The Designated Size（100 million yuan）

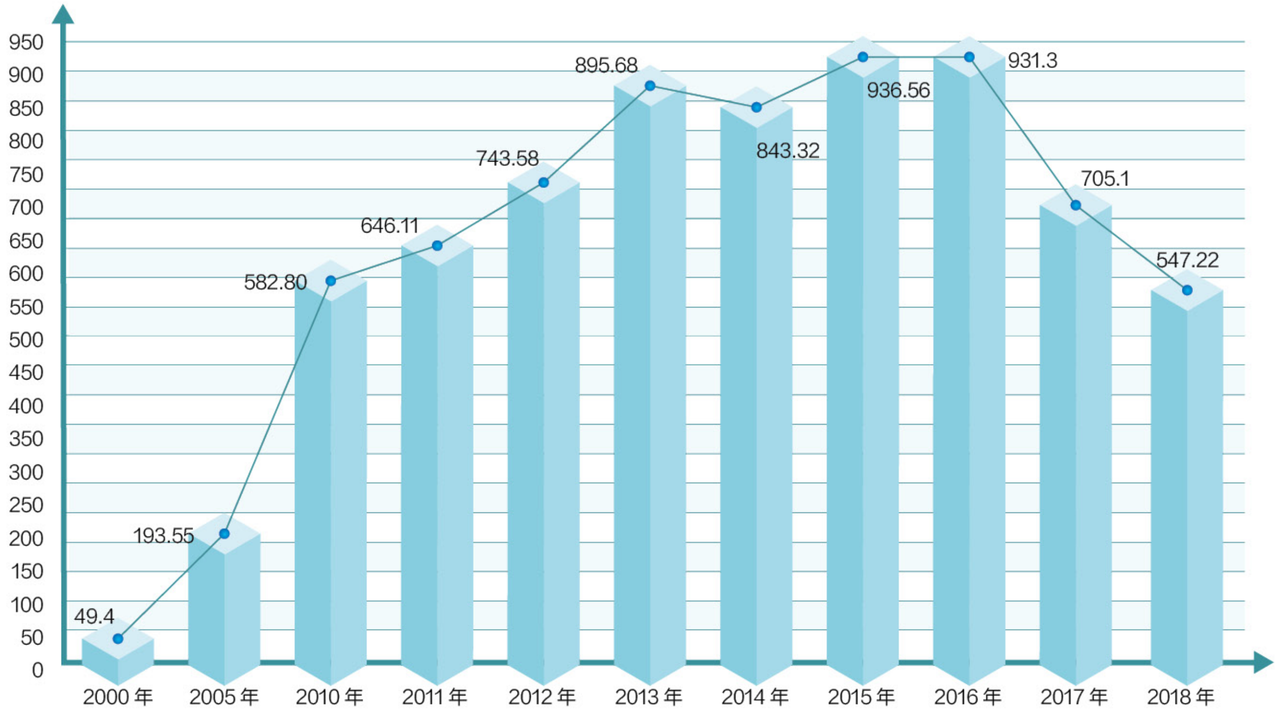

房地产开发投资（亿元）

Investment In Real Estate Development（100 million yuan）

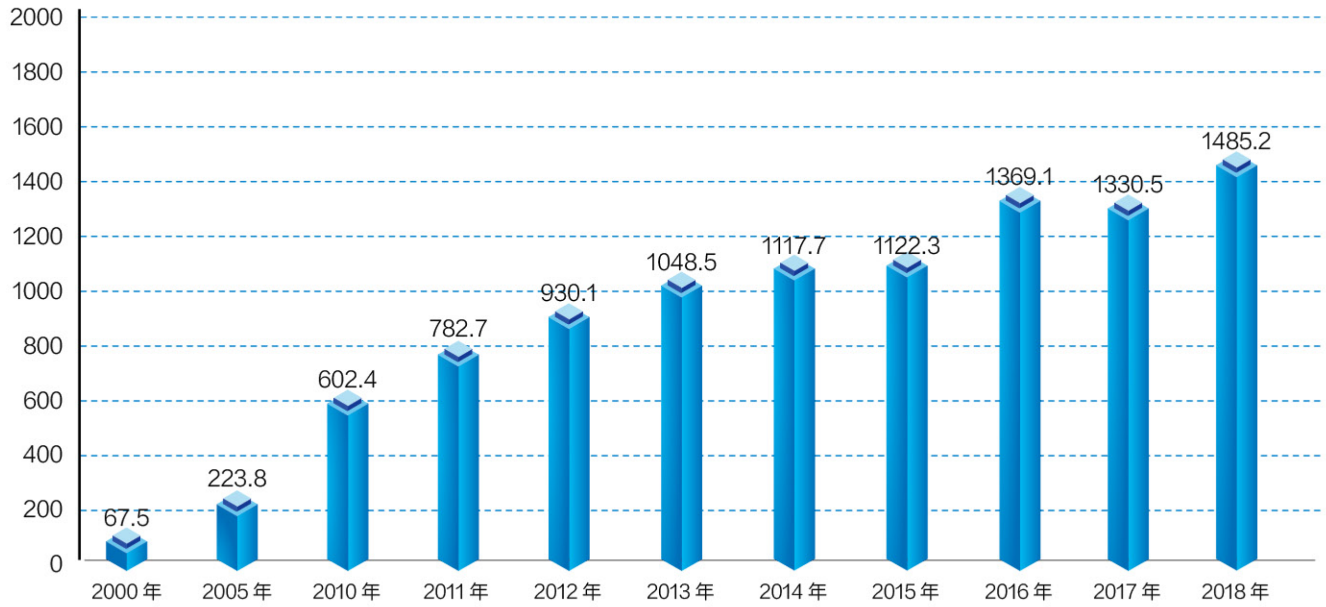

房屋销售面积（万平方米）

Floor Space Of Commercial Buildings Sold（10 000 sq.m）

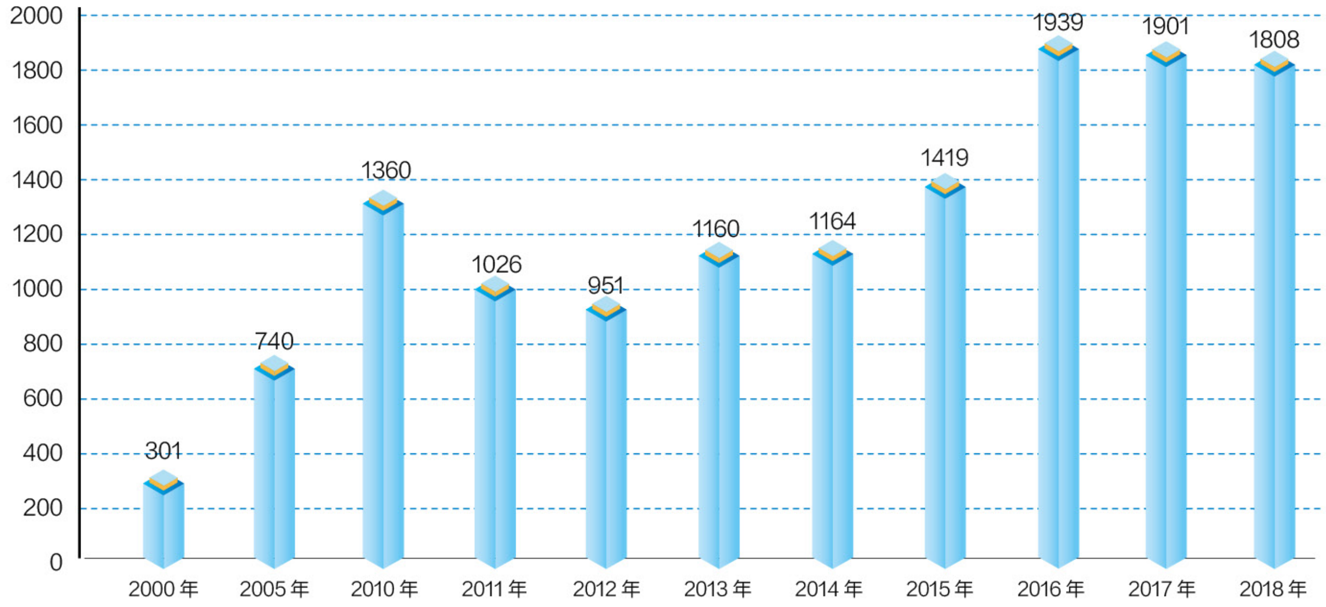

消费构成（%）（2018 年）

2018 Composition Of Consumption（%）

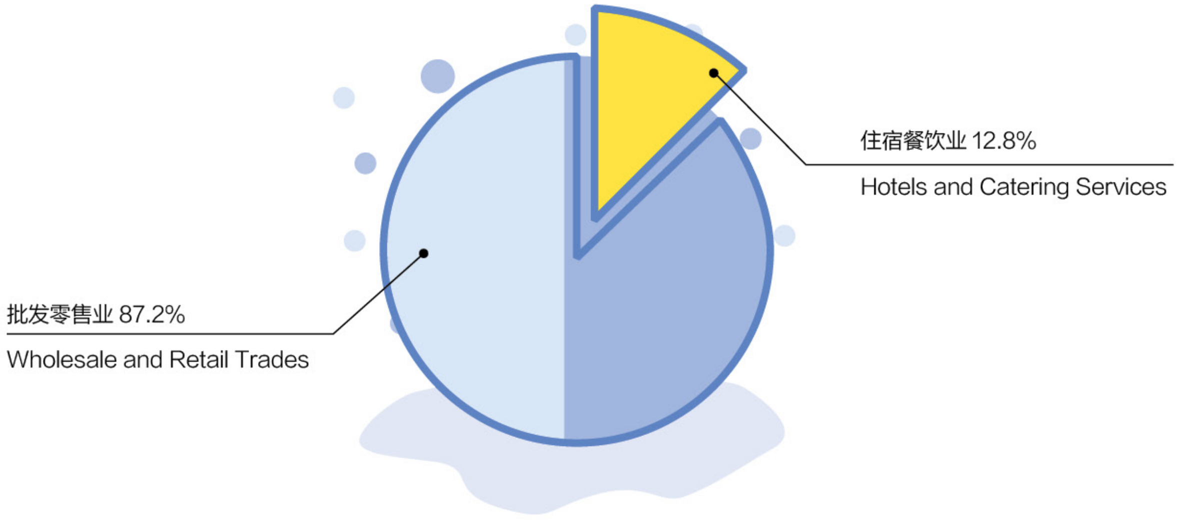

社会消费品零售总额（亿元）

Total Retail Sales Of Consumer Goods（100 million yuan）

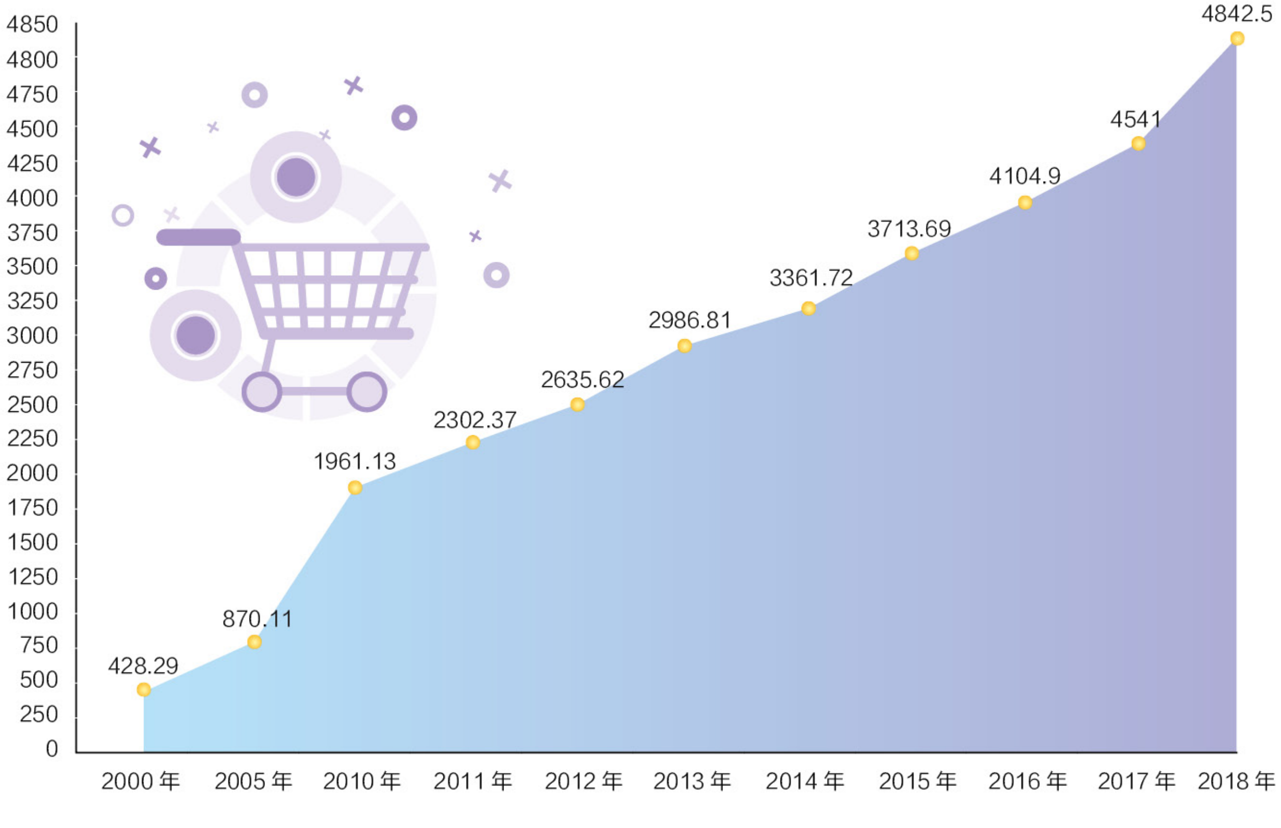

旅游总人数（万人次）

Number Of Tourists（10 000 person–times）

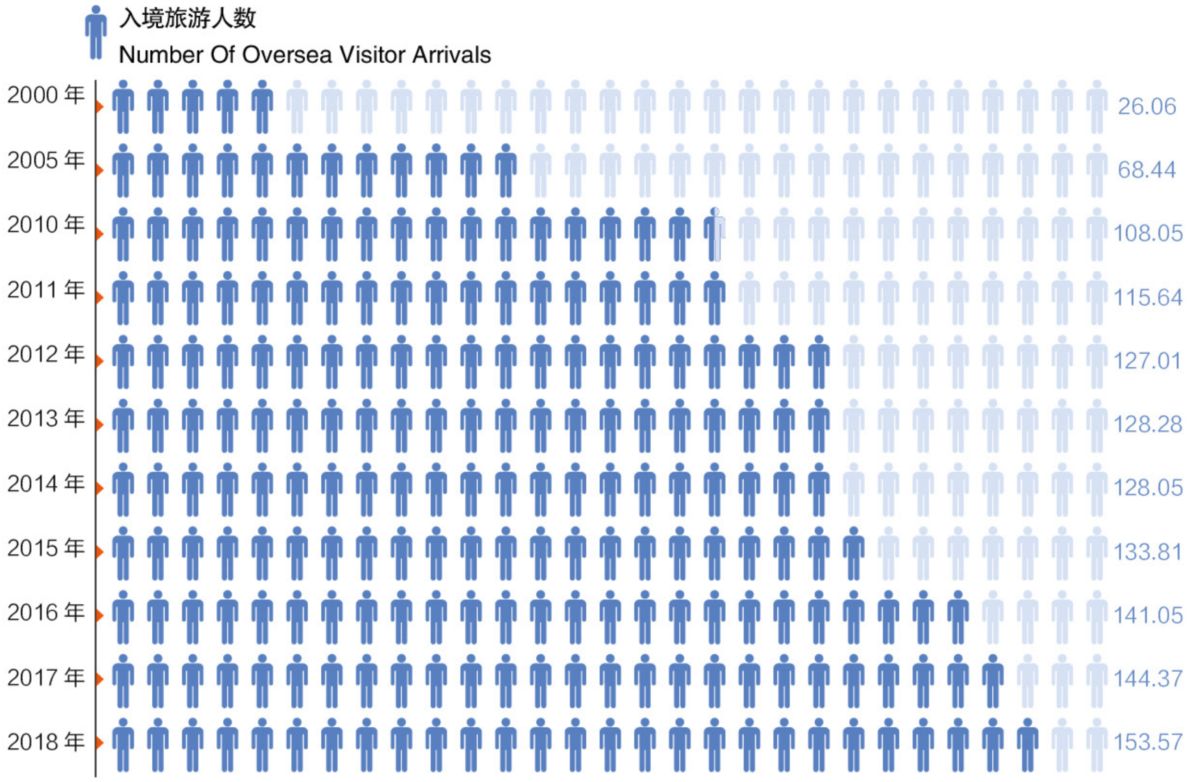

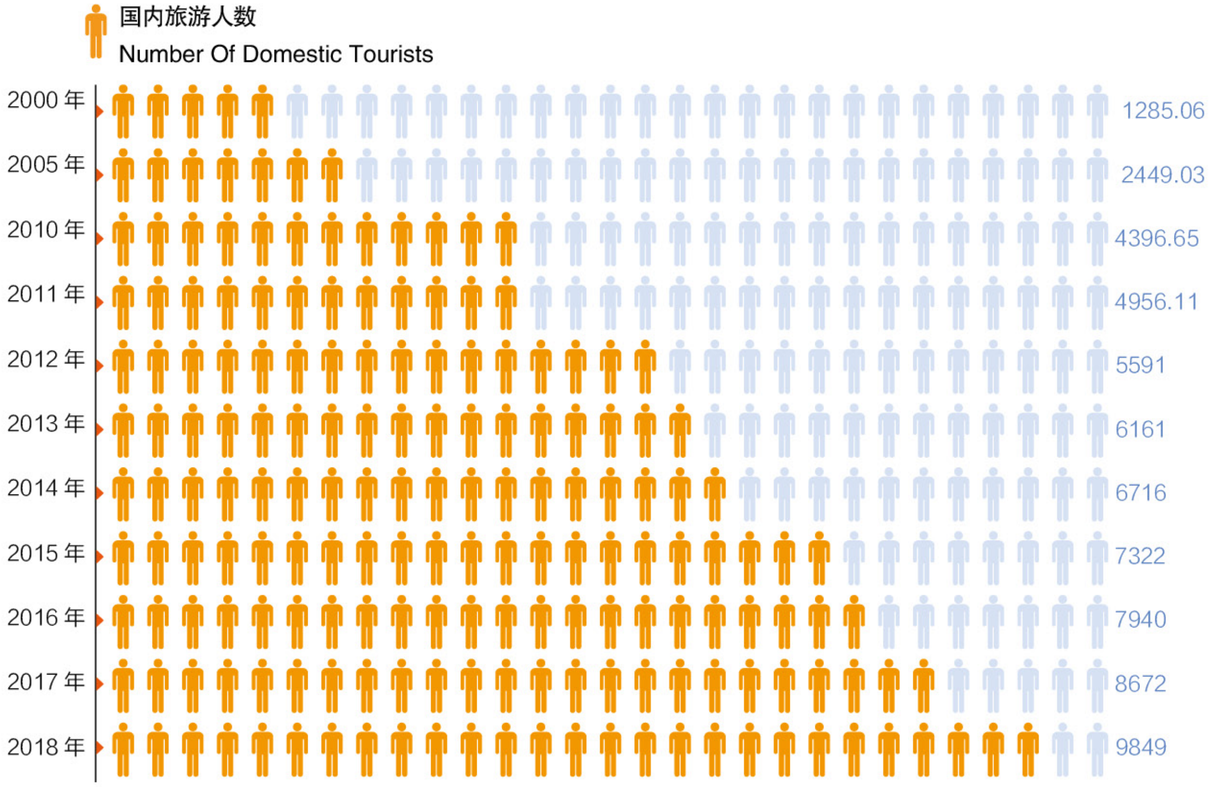

地方财政收入（亿元）

Revenue Of Local Government Finance（100 million yuan）

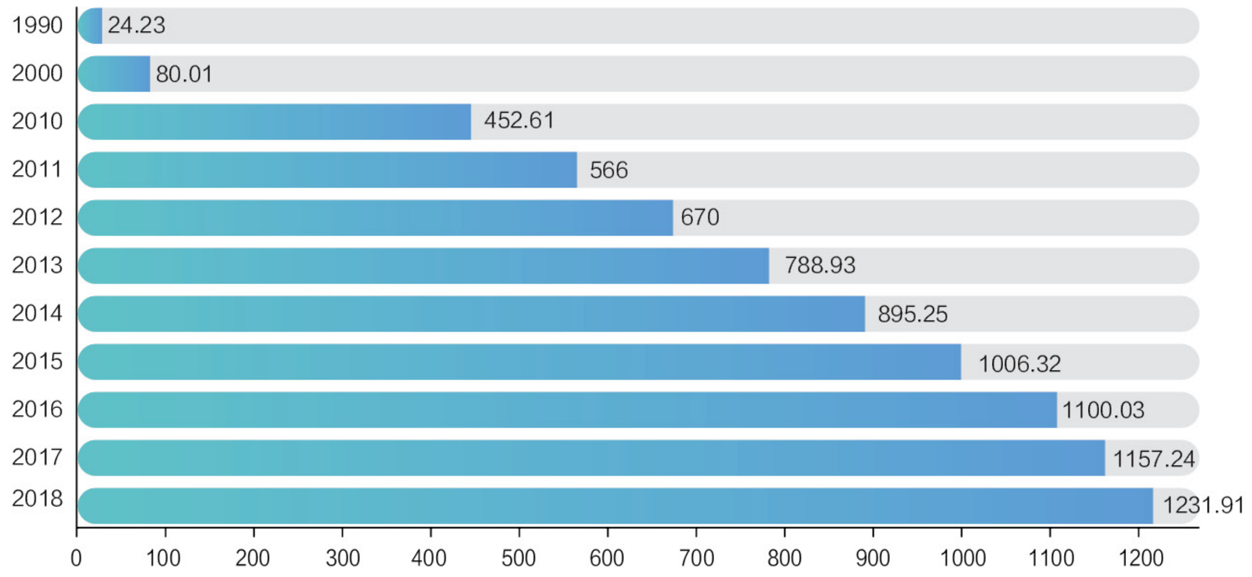

注：2002 年以后财政收入为一般预算收入数。

Note：Since 2002，revenue of government finance refers to general budgetary revenue.

金融机构年末人民币存贷款余额（亿元）

Year-end Savings Deposits And Loans Of Financial Institution（RMB）（100 million yuan）

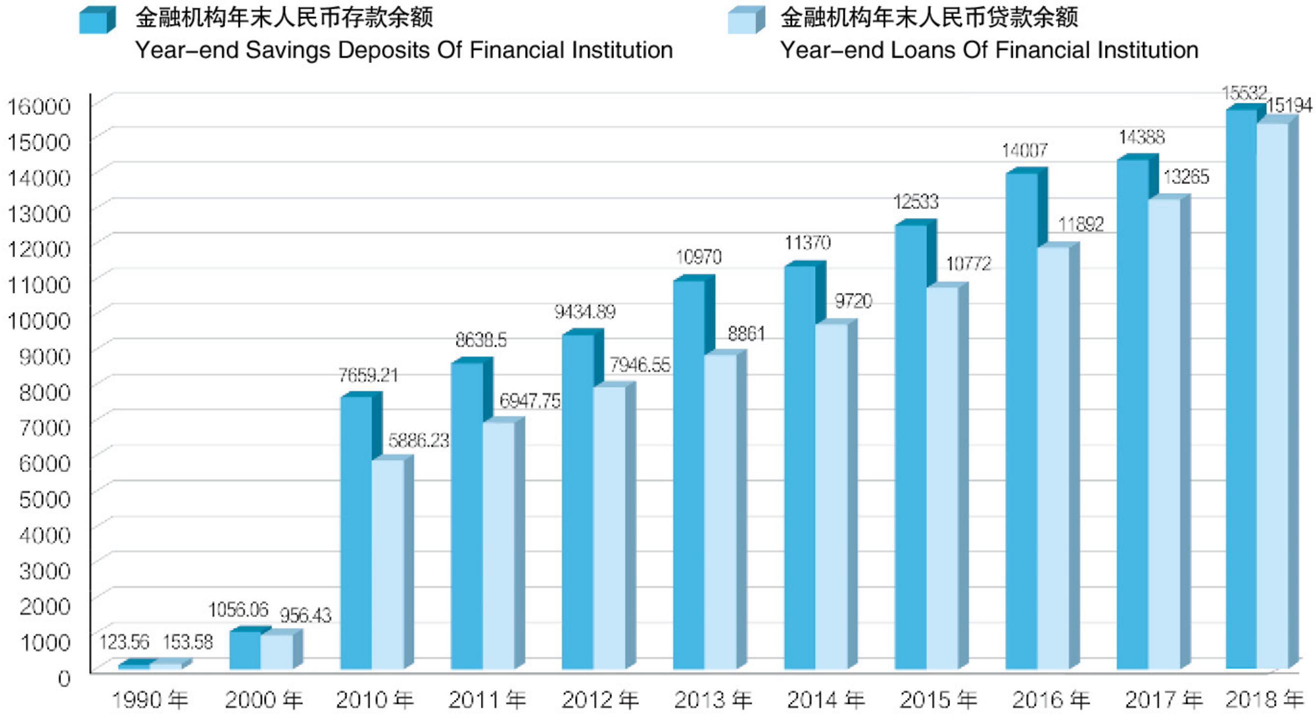

社会从业人数（万人）

Social Employment（10 000 persons）

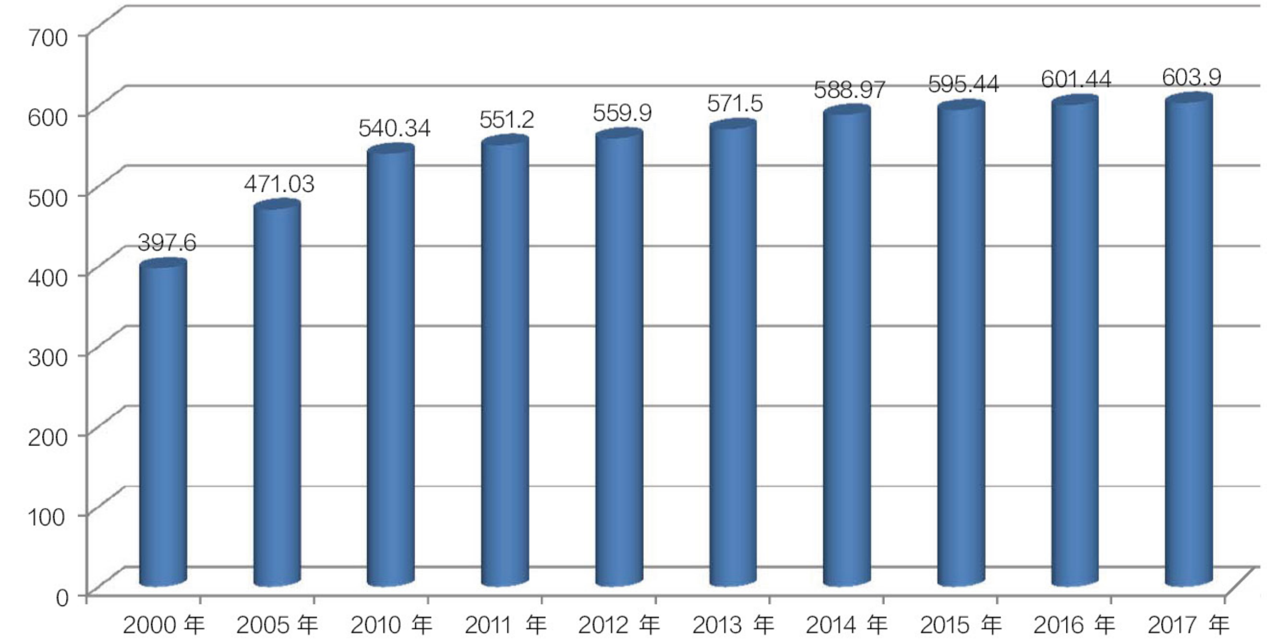

在岗职工平均工资（元）

Average Wage Of Employed Staff And Workers（yuan）

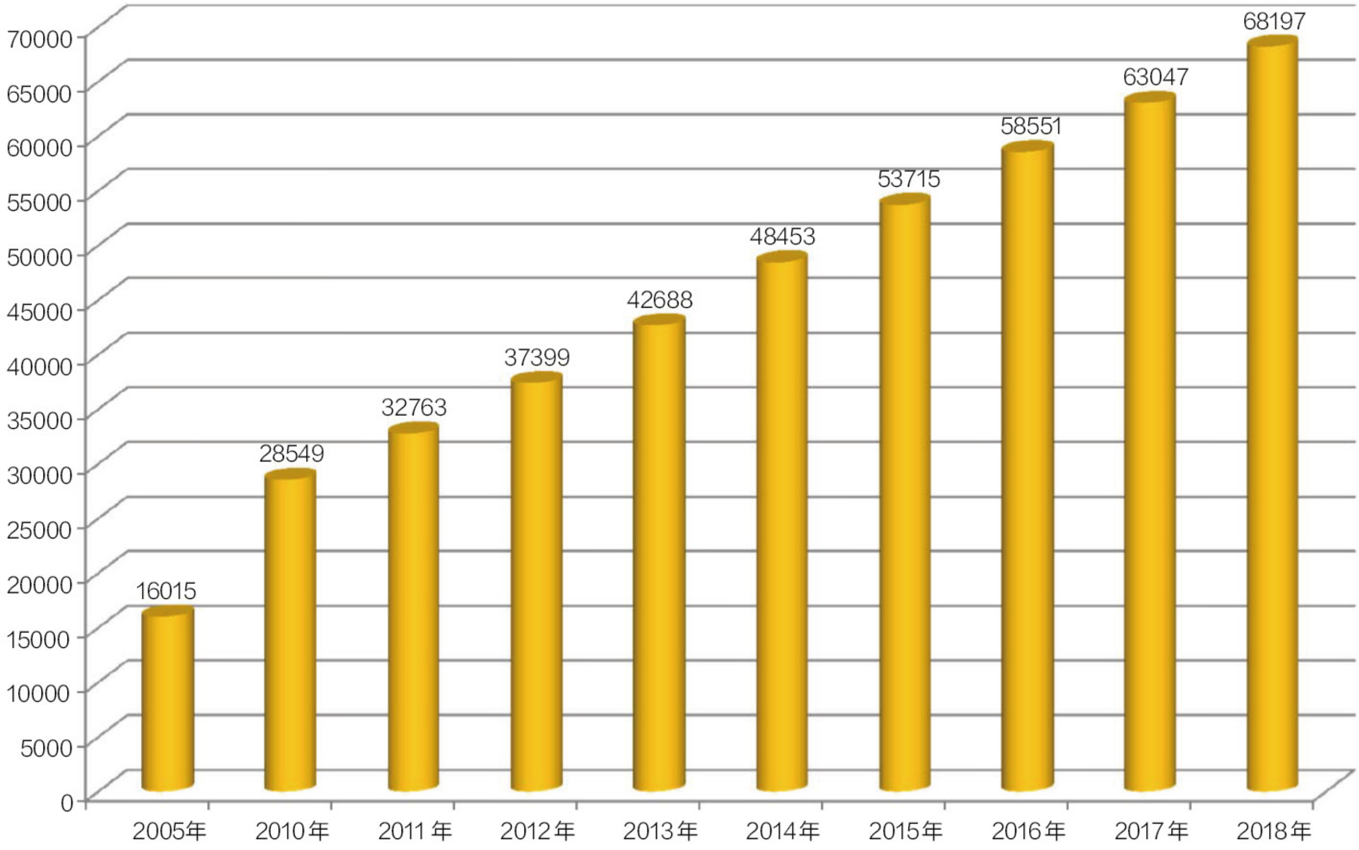

居民消费价格指数（上年＝100）

Consumer Price Indices（preceding year＝100）

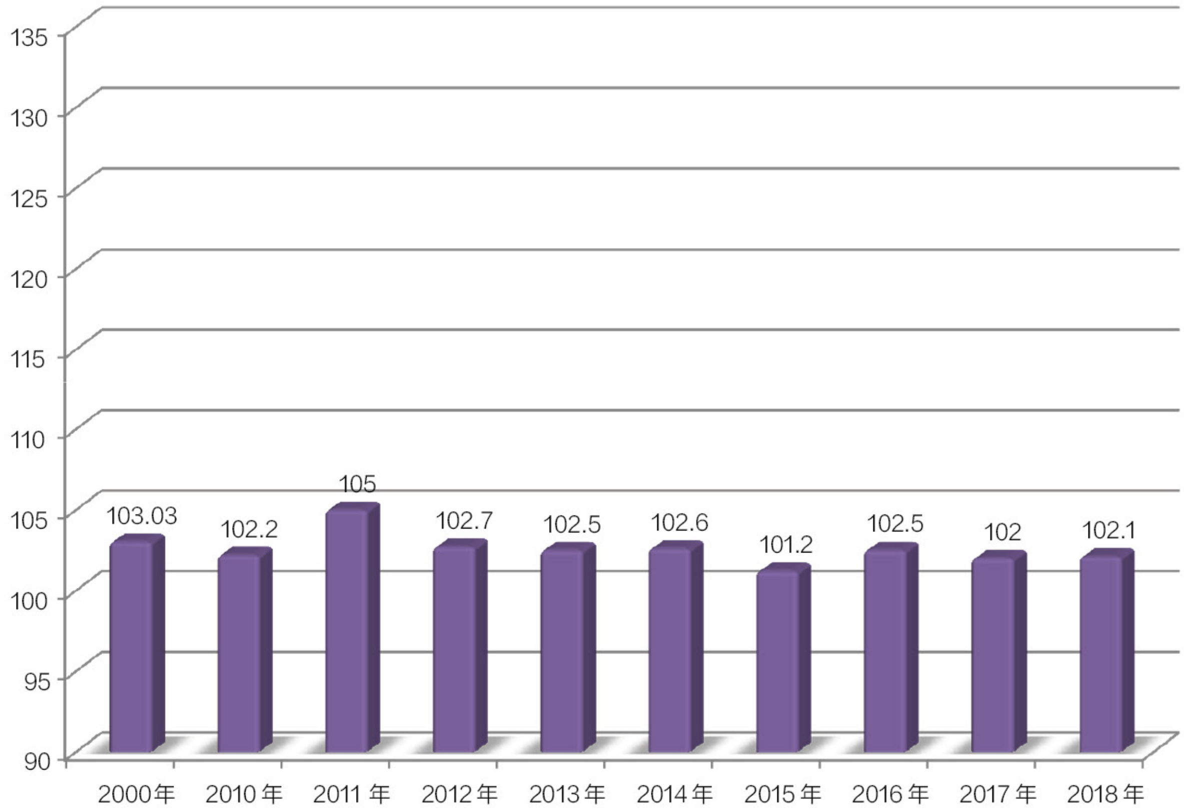

商品零售价格指数（上年＝100）

Retail Price Indices（preceding year＝100）

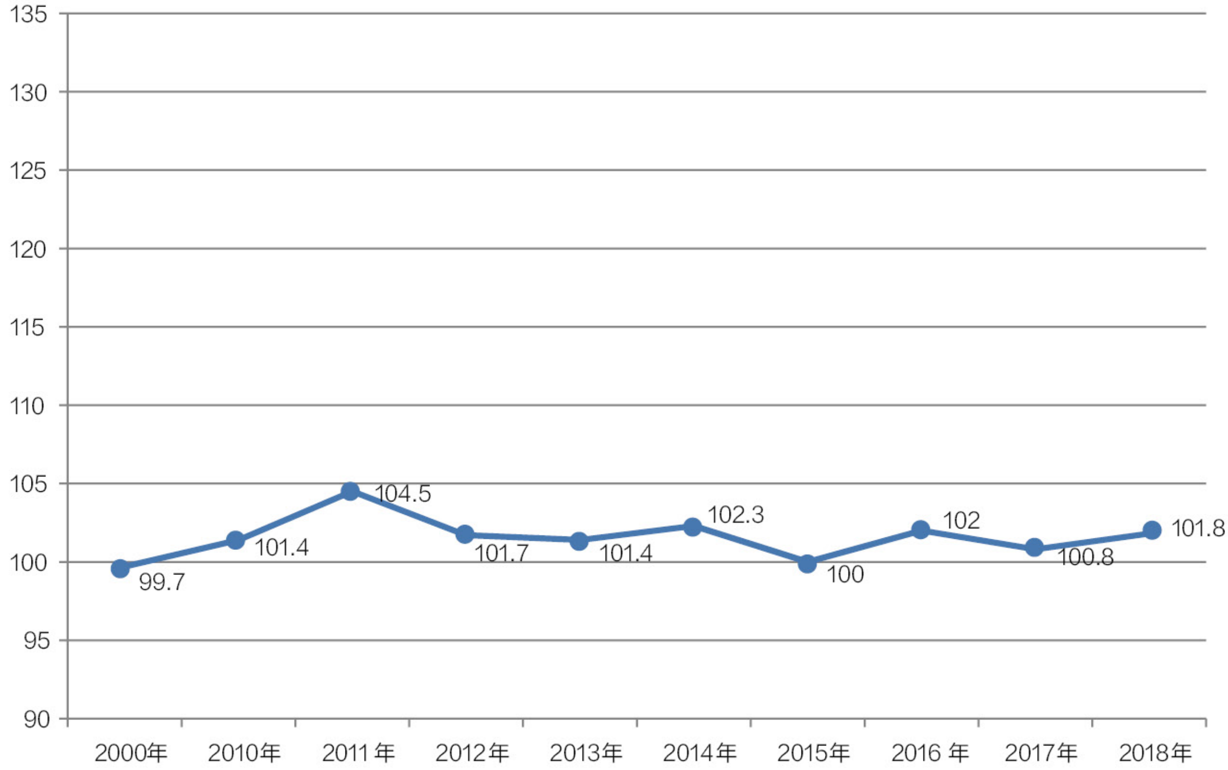

城市建成区面积（平方公里）

Developed Area Of the City（sq.km）

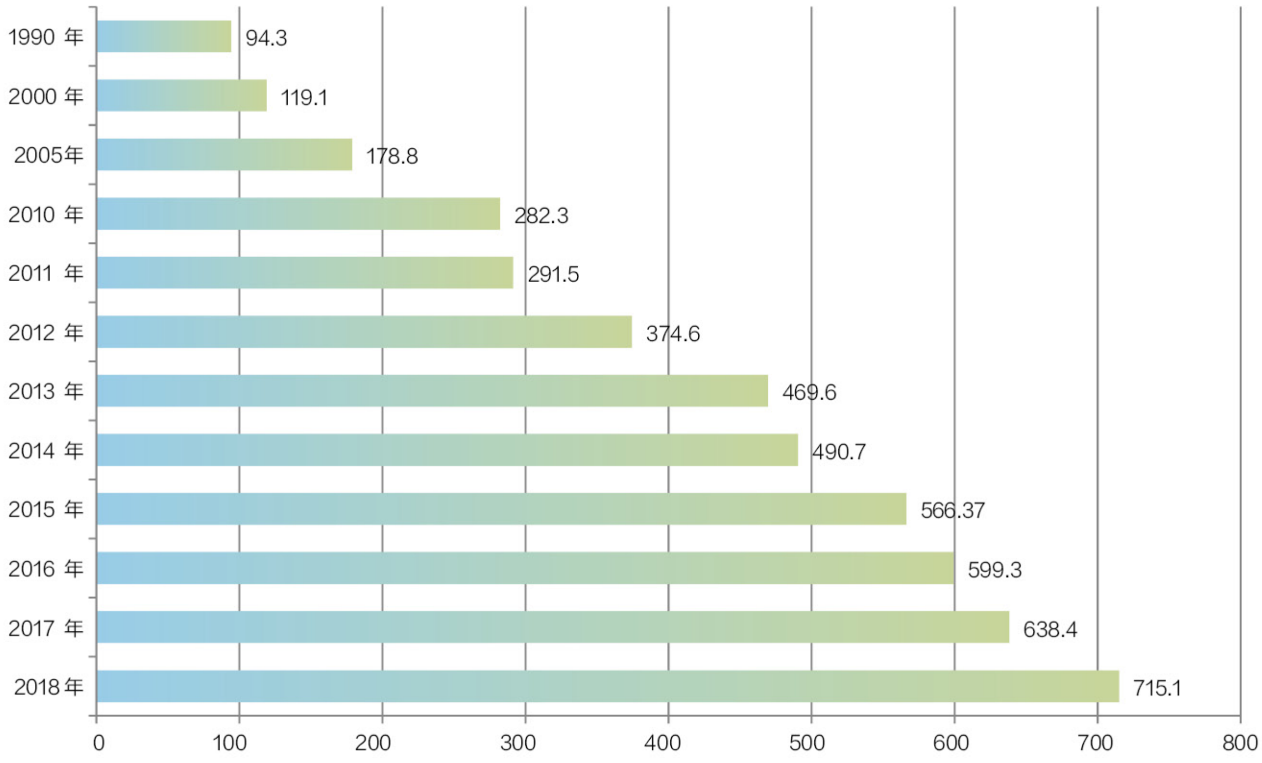

铺装道路面积（万平方米）

Area Of Roads（10 000 sq.m）

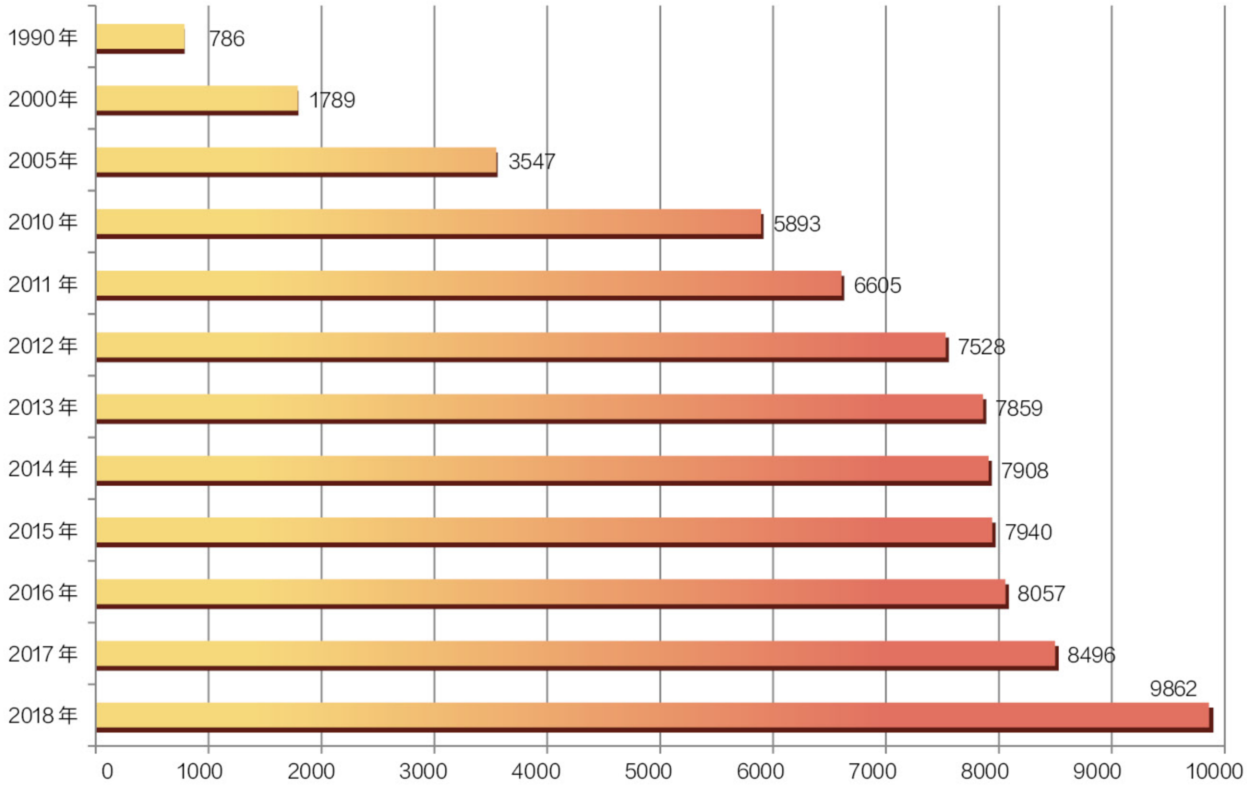

医疗卫生机构（个）

Health Care Institutions（unit）

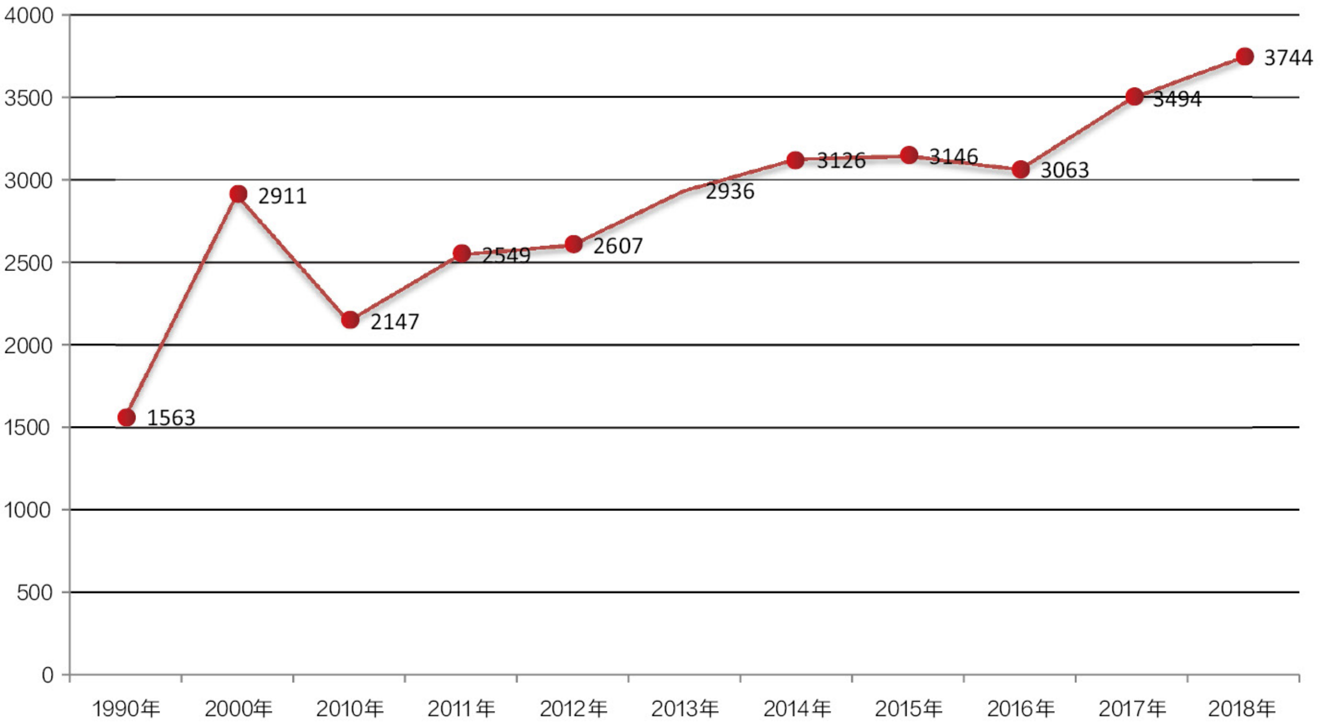

普通高校在校人数（万人）

Students Enrollment Of Regular Institutions Of Higher Education（10 000 persons）

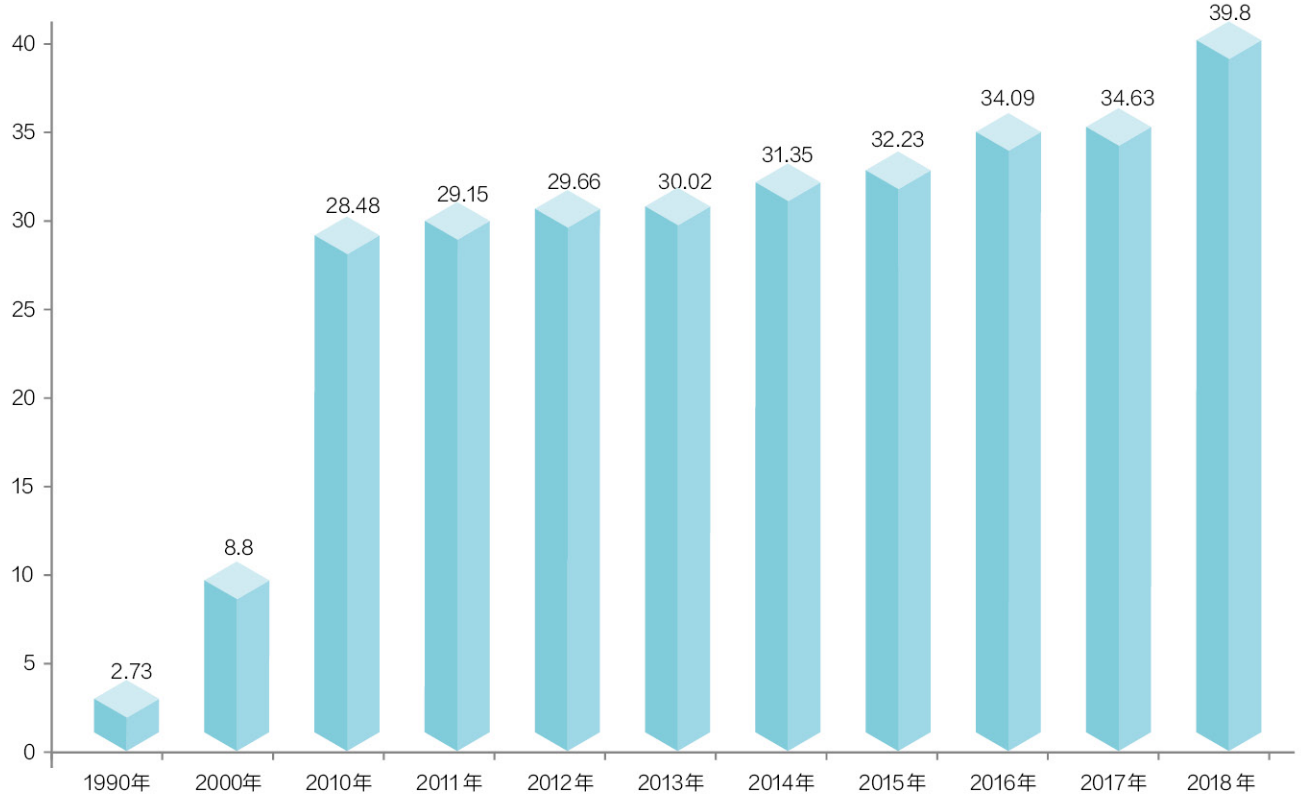

编辑委员会

◆ 主　　任：刘岐涛

◆ 副 主 任：纪中锐　于国庆　于春涛　李坤兴　刘　伟　王志强
张茂海　张德祥　胡宝良　侯　杰　石成伟　毛小美
孙昭信

◆ 委　　员：（以姓氏笔划为序）
于泳洋　马　奇　丰宗义　王　磊　王之钰　王金峰
王维周　王福临　付增梁　李　勇　李连登　宋　波
林玉森　周　巍　郑清波　胡建伟　逄淑英　董　清

◆ 主　　编：刘岐涛

◆ 常务副主编：刘　伟

◆ 副 主 编：王　磊　刘　芹

◆ 责任编辑：钟　钰

◆ 编　　辑：（以姓氏笔划为序）
于世涛　于安生　王　元　王　媛　王明明　仇海燕
尹正德　卢倩倩　冯　健　朱丽华　刘　生　刘　斌
刘　斌　刘连玫　刘俐娜　孙　辉　孙仁太　孙树盛
纪　琳　李凤羽　李守宝　杨雪雅　肖婷婷　宋天戈
张玮华　张砚慧　张翊钧　张瑾璟　陈旭亮　陈　菁
陈　强　武　斌　林乐靖　赵　阳　种玉金　高　爽
唐　东　窦　蕾

EDITORIAL BOARD

Director：Liu Qitao

Deputy Director：

Ji Zhongrui	Yu Guoqing	Yu Chuntao	Li Kunxing	Liu Wei	Wang Zhiqiang
Zhang Maohai	Zhang Dexiang	Hu Baoliang	Hou Jie	Shi Chengwei	Mao Xiaomei
Sun Zhaoxin					

Board：（in order of strokes of Chinese Surname)

Yu Yongyang	Ma Qi	Feng Zongyi	Wang Lei	Wang Zhiyu	Wang Jinfeng
Wang Weizhou	Wang Fulin	Fu Zengliang	Li Yong	Li Liandeng	Song Bo
Lin Yusen	Zhou Wei	Zheng Qingbo	Hu Jianwei	Pang Shuying	Dong Qing

Editor-in-chief：Liu Qitao

Managing Deputy Editor-in-chief：Liu Wei

Deputy Editor-in-chief：Wang Lei Liu Qin

Coordinator：ZhongYu

Editorial Staff：（in order of strokes of Chinese Surname)

Yu Shitao	Yu Ansheng	Wang Yuan	Wang Yuan	Wang Mingming	Qiu Haiyan
Yin Zhengde	Lu Qianqian	Feng Jian	Zhu Lihua	Liu Sheng	Liu Bin
Liu Bin	Liu Lianmei	Liu Lina	Sun Hui	Sun Rentai	Sun Shusheng
Ji Lin	Li Fengyu	Li Shoubao	Yang Xueya	Xiao Tingting	Song Tiange
Zhang Weihua	Zhang Yanhui	Zhang Yijun	Zhang Jinjing	Chen Xuliang	Chen Jing
Chen Qiang	Wu Bin	Lin Lejing	Zhao Yang	Chong Yujin	Gao Shuang
Tang Dong	Dou Lei				

编者说明

一、《青岛统计年鉴 -2019》是一部全面反映青岛市国民经济和社会发展情况、信息高度密集的资料工具书。

二、《青岛统计年鉴 -2019》共包括发展成果图、统计公报、统计表、附录四大部分。

统计表部分收录了 2018 年青岛市经济和社会等各方面的统计数据，以及建国以来重要年份和改革开放以来的主要统计数据，包括：综合，人口，从业人员及职工工资，固定资产投资，对外经济贸易，城市建设、环境保护，能源消耗，财政金融和保险业，价格指数，人民生活，农业，工业，建筑业，运输、邮电，批发和零售业，住宿、餐饮业和旅游，教育、科技和文化，体育、卫生和民政、司法。

为方便读者正确地使用年鉴资料，各篇章前设有《简要说明》，概括介绍各篇主要内容和资料来源；篇末还附有《主要统计指标解释》。

三、本《年鉴》部分历史数据已根据经济普查和农业普查数据进行了调整，在此之前公布的数据凡与本年鉴数字不符的一律以本年鉴为准。

四、本年鉴中的符号说明：年鉴各表中的“空格”表示该项统计指标数据不足本表最小单位数、数据不详或无该项数据；"#" 表示其中的主要项。

五、《青岛统计年鉴》自公开出版以来，受到社会各界的关心和支持，对于年鉴的内容和编辑工作提出了许多宝贵的意见，对此我们深表谢意。限于我们的水平，欢迎读者继续对年鉴的不足之处给予批评和指正，帮助我们进一步改进年鉴编辑工作，以期更好地为广大读者服务。

《青岛统计年鉴》编委会

2019 年 7 月

Editor's Note

Ⅰ. *Qingdao Statistical Yearbook 2019* is a very important reference book, which reflects various aspects of Qingdao's social and economic development and contains High–density information.

Ⅱ.The yearbook contains the following four parts: achievement graphs, statistic gazette, statistics and appendixes.

Statistics contains all kinds of data on Qingdao's social and economic development in 2018, and data in significant year or data since the beginning of reform and opening up, including: General Survey; Population; Employment and Wages; Investment in Fixed Assets; Foreign Trade; City Construction and Environment Protection； Consumption of Energy; Government Finance ,Financial Intermediation and Insurance; Price; People's Living Conditions; Agriculture； Industry； Construction； Transport, Postal and Telecommunication Services； Wholesale and Retail Trades； Hotels, Catering Services and Tourism； Education, Science and Culture; Sports, Public Health and Civil Affairs, Judicial Affairs; Enterprises Survey.

In brief introduction at the beginning of each chapter, main coverage, data sources and statistical coverage are concerned. Meanwhile explanatory notes on major statistical indicators have been attached for readers to use the data correctly.

Ⅲ. All of the data in the book are in accordance with new administrative division.

Some of the data have been adjusted on the basis of statistics of economic census and agriculture census . In any case the data of this book shall be deemed as the authentic ones.

Ⅳ. Marks in this book:

"(blank)" indicates that the figure is not large enough to be measured with the smallest unit in the table, the data is not available, or the data is not available;

"#" indicates the major items of the total.

Ⅴ. Previous editions of Qingdao Statistical Yearbook have won wide acclaim among the readers. In order to excel, we welcome all candid comments and criticism from our readers.

Editorial Board

JULY,2019

目　　录

CONTENTS

2018 年青岛市国民经济和社会发展统计公报 …… 1
Statistics Communique Qindao’s Economic And Social Development In The Year Of 2018

一、综　　合

Chapter 1.　GENERAL SURVEY

简要说明 …… 30
Brief Introduction

1—1　行政区划（2018 年底） …… 31
Administrative Division（End Of 2018）

1—2　气象情况（2018 年） …… 32
Meteorology（2018）

1—3　市区分月气象情况（2018 年） …… 32
Monthly Meteorology Of Urban Area（2018）

1—4　各部门机构数（2018 年底） …… 33
Grass-Roots Untis In Various Sectors（End Of 2018）

1—5　按行业分法人单位数 …… 35
Number Of Corporate Units By Sector

1—6　国民经济主要平均指标 …… 36
Average Indicators On National Economy

1—7　主要年份社会经济主要指标 …… 38
Major Year’s Indicators On Society And Economy

1—8　国民经济主要结构指标 …… 44
Composition Indicators On National Economy

1—9　平均每天主要社会经济活动 …… 46
Selected Indicatirs On Average Dally Soclal And Economic Activities

1—10　主要指标占全国全省比重（2018 年） …… 48
Percentage Of Main Indicators To China And Shandong（2018）

1—11　主要年份全市生产总值（按当年价格计算） …… 50
Major Year’s Gross Domestic Product（At Current Price）

1—12　主要年份全市生产总值构成（以全市生产总值为 100）…… 52
Compostion Of Major Yrar’s Gross Domestic Product（ Gross Domestic Product=100）
1—13　主要年份全市生产总值增长速度（以上年为 100）…… 53
Growth Rate Of Major Year’s Gross Domestic Product（ Preceding Year=100）
1—14　分市、区生产总值（2018 年）…… 55
Gross Domestic Product By Region（2018）
1—15　按支出法计算的全市生产总值（2018 年）…… 56
Griss Domestic Product By Expenditure Approach（2018）
1—16　全市生产总值构成（2018 年）…… 57
Composition Of Gross Domestic Product（2018）
主要统计指标解释 …… 58
Explanatory Notes on Main Statistical Indicators

二、人　口
Chapter 2.　POPULATION

简要说明 …… 64
Brief Introduction
2—1　主要年份全市户籍人口数 …… 65
Major Year’s Total Registred Population
2—2　主要年份全市户数、人口数、人口密度（户籍）…… 66
Major Year’s Houstholds，Population And Density Of Population（With Permanent Residence）
2—3　主要年份全市常住人口数 …… 67
Major Year’s Total Resident Population
2—4　青岛市常住人口 …… 67
Total Resident Population
2—5　主要年份常住人口城镇化率…… 68
The Urbanization Rate Of The Main Year Permanent Residents
2—6　分市、区土地面积（2018 年底）…… 69
Land Area（End Of 2018）
2—7　第一、二、三、四、五、六次人口普查主要数据 …… 70
Main Data From The Six National Population Censuses
2—8　计划生育情况（1978-2018 年）…… 71
Family Planning Situation（1978 – 2018）
2—9　分市、区计划生育情况（2018 年）…… 72
Family Planning Situation By Region（ 2018）

主要统计指标解释 …… 73
Explanatory Notes on Main Statistical Indicators

三、从业人员及职工工资
Chapter 3.　EMPLOYMENT AND WAGES

简要说明 …… 76
Brief Introduction
3—1　社会就业人数（1978–2017 年） …… 77
Social Employment（1978–2017）
3—2　主要年份全市单位就业人员人数 …… 78
Major Year's Number Of Employed Persons In All Units Of The City
3—3　全市单位国民经济各行业就业人员人数（2018 年） …… 79
Number Of Employed Persons In All Units Of The City By Sector（2018）
3—4　全市单位分市、区国民经济各部门就业人员人数（2018 年） …… 80
Number Of Employed Persons In All Untts Of The City By Region And Sector（2018）
3—5　全市单位分市、区全部就业人员人数（2018 年底） …… 84
Number Of Employed Persons In All Units Of The City By Region（End Of 2018）
3—6　全市就业人员工资总额、平均工资（1978–2018 年） …… 85
Total Wages And Average Wage Of Citywide Full–Time Employees（1978–2018）
3—7　全市就业人员工资总额、平均工资指数（1978–2018 年） …… 86
Indexes Of Total Wages And Average Wage Of Citywide Full–Time Employees（1978–2018）
3—8　分行业就业人员平均工资（2018 年） …… 87
Average Wage Of Full–Time Employees By Industry（2018）
3—9　分市、区就业人员工资总额（2018 年） …… 88
Total Wages Of Full–Time Employees By City Or District（2018）
3—10　分市、区就业人员平均工资（2018 年） …… 89
Average Wage Of Full–Time Employees By City Or District（2018）
主要统计指标解释 …… 90
Explanatory Notes on Main Statistical Indicators

四、固定资产投资
Chapter 4.　INVESTMENT IN FIXED ASSETS

简要说明 …… 94
Brief Introduction

4—1 主要年份固定资产投资 …… 95
Major Yeay' s Investment In Fixed Assets
4—2 主要年份固定资产投资构成（以投资总额为 100） …… 97
Composition Of Major Year' s Investment In Fixed Assets（Total Investment=100）
4—3 按三次产业分规模以上固定资产投资增速（2018 年） …… 99
Growth Rate Of Investment In Fixed Assets Above Designated Size By Three Strata Of Industry（2018）
4—4 分市、区固定资产投资额增速（2018 年） …… 99
Growth Rate Of Investment In Fixed Assets By Region（2018）
4—5 规模以上固定资产投资增速（2018 年） …… 100
Investment In Fixed Assets Above Designated Size（2018）
4—6 主要年份房地产开发投资 …… 105
Major Year' s Investment In Real Estate Devealopment
4—7 房地产开发投资情况（2018 年） …… 106
Investment In Real Estate Development（2018）
4—8 房地产施工、竣工面积及竣工价值（2018 年） …… 107
Floor Space Under Construction And Completed And Completed Value Of Real Estate（2018）
4—9 商品房屋销售情况（2018 年） …… 107
Basic Statistics On Sales Of Commercialized Builbings（2018）
主要统计指标解释 …… 108
Explanatory Notes on Main Statistical Indicators

五、对外经济贸易
Chapter 5. FOREIGN TRADE

简要说明 …… 112
Brief Introduction
5—1 青岛口岸进出口总额（1985-2018 年） …… 113
Total Value Of Imports And Exports Of Qingdao Port（1985-2018）
5—2 进出口总额（1988-2018 年） …… 114
Total Value Of Imports And Exports（1988-2018）
5—3 分国别外贸出口总额 …… 115
Total Value Of Exports By Countries Or Regions
5—4 外贸出口商品分类 …… 116
Export Commodities By Category
5—5 外贸进口商品分类 …… 117
Import Commodities By Category

5—6　二十大出口商品出口情况 …… 118
Information On The Exportation Of Top 20 Products
5—7　二十大进口商品进口情况 …… 119
Information On The Importation Of Top 20 Products
5—8　利用外资情况（2000–2018 年） …… 120
Utilization Of Foreign Capotal（2000–2018）
5—9　当年外商直接投资项目数和投资额（2018 年） …… 122
Number Of Projects And Total Amount Of Foreign Direct Investment（2018）
5—10　对外投资与经济合作 …… 123
Outbound Investment And International Economic Cooperation
5—11　对外投资分国别（地区）情况表（2018 年） …… 123
Information On Outbound Investment By Country/Region For（2018）
主要统计指标解释 …… 125
Explanatory Notes on Main Statistical Indicators

六、城市建设、环境保护
Chapter 6.　COTY CONSTRUCTION AND ENVIRONMENT PROTECTION

简要说明 …… 128
Brief Introduction
6—1　主要年份城市建设和公用事业 …… 129
Major Year’s City Construction And Public Utilities
6—2　全年供电（2018 年） …… 133
Annual Electricity Supply（2018）
6—3　分行业用电（2018 年） …… 134
Electrictty Consumption By Sector（2018）
6—4　城市供水 …… 135
Urban Water Supply
6—5　城市公共交通 …… 136
Urban Public Traffic
6—6　城市供气 …… 137
Urban Gas Supply
6—7　城市环境卫生 …… 138
Urban Environmental Sanitation
6—8　城市道路、下水道及绿化 …… 139
Urban Road，Sewage And Green

6—9　环境保护基本情况 …… 140
Basic Conditions Of Environmental Protection
6—10　环境质量状况 …… 140
Environment Condition
6—11　工业“三废”排放情况 …… 141
Discharge Conditions Of Industrial Waste Wster, Waste Gas And Solid Waste
主要统计指标解释 …… 142
Explanatory Notes on Main Statistical Indicators

七、能源消耗
Chapter 7.　CONSUMPTION OF ENERGY

简要说明 …… 146
Brief Introduction
7—1　规模以上工业主要能源消费与库存（2018 年） ……147
Consumption And Stock Of Major Energy Of Industry Above Designated Size（2018）
7—2　规模以上工业主要能源分行业消费量（2018 年） ……148
Major Energy Consumption Of Industry Above Designated Size By Sector（2018）
7—3　重点耗能工业企业能源加工转换（2018 年） …… 149
Energy Conversion Of Major Energy-Consuming Industrial Enterprises（2018）
7—4　规模以上工业主要能源工业消费量（2018 年） …… 150
Major Energy Consumption Of Industry Above Designated Size（2018）
主要统计指标解释 …… 158
Explanatory Notes on Main Statistical Indicators

八、财政、金融和保险业
Chapter 8.　GOVERNMENT FINANCE FINACIAL INTERMEDIATION AND INSURANCE

简要说明 …… 160
Brief Introduction
8—1　主要年份地方财政收支 …… 161
Mafor Year’s Revenue And Expendrrure Of Local Government Finance
8—2　分市、区公共财政预算收入（2018 年） …… 162
Pulbic Finance Budget Revenue By City And Districl（2018）
8—3　分市、区公共财政预算支出（2018 年） …… 164
Public Finance Budget Revenue By City And District（2018）

8—4　主要年份金融系统人民币存贷款（年末余额）…… 166
Major Year’s Deposits And Loans Of Financial Institutions（Year–End Balance）
8—5　金融系统人民币存贷款（年末余额）…… 167
Deposits And Loans Of Financial Institutions（Year–End Balance）
8—6　国内保险业务（2000–2018 年）…… 168
Domestic Insurance Business（2000–2018）
主要统计指标解释 …… 170
Explanatory Notes on Main Statistical Indicators

九、价格指数

Chapter 9.　PRICE INDEXES

简要说明 …… 174
Brief Introduction
9—1　主要年份居民消费和商品零售价格指数 …… 175
Major Year’s Consumer And Retail Price Indexes
9—2　主要年份居民消费和商品零售价格指数（以 1950 年价格为 100）…… 176
Major Year’s Consumer And Retail Price Indexes（1950 =100）
9—3　主要年份生产投资价格指数 …… 177
Major Year’s Price Indexes For Production And Investment
9—4　工业生产者出厂价格指数 …… 178
Producer Price Indexes For Industral Producers
9—5　工业生产者购进价格指数 …… 180
Purchasing Price Indexes For Industrial Producers
9—6　按工业行业分工业生产者出厂价格指数 …… 182
Producer Price Indexes For Industrial Producers By Sector
9—7　固定资产投资价格指数 …… 184
Price Indexes For Investment In Fixed Assets
9—8　住宅销售价格指数（2018 年）…… 184
Sales Price Indexes For Residence（2018）
9—9　居民消费价格分类指数（2018 年）…… 185
Consumer Price Indexes By Category（2018）
9—10　商品零售价格分类指数（2018 年）…… 187
Retail Price Indexes By Category（2018）
主要统计指标解释 …… 189
Explanatory Notes on Main Statistical Indicators

十、人民生活
Chapter 10. PEOPLE SLIVING CONDITIONS

简要说明 …… 192
Brief Introduction

10—1 城市居民收支（1978–2014 年） …… 193
Income And Expenditure Of Urban Residunts（1978–2014）

10—2 农村居民收支（1978–2014 年） …… 194
Income And Expenditure Of Rural Residents（1978–2014）

10—3 城乡居民住房面积（1990–2014 年） …… 195
Housing Area Of Urban And Rural Residents（1990–2014）

10—4 全体居民家庭基本情况（2015 年） …… 196
Basic Information On All Households（2015）

10—5 全体居民家庭基本情况（2016 年） …… 197
Basic Information On All Households（2016）

10—6 全体居民家庭基本情况（2017 年） …… 198
Basic Information On All Households（2017）

10—7 全体居民家庭基本情况（2018 年） …… 199
Basic Information On All Households（2018）

10—8 城镇居民家庭基本情况（2015 年） …… 200
Basic Conditions Of Urban Households（2015）

10—9 城镇居民家庭基本情况（2016 年） …… 201
Basic Conditions Of Urban Households（2016）

10—10 城镇居民家庭基本情况（2017 年） …… 202
Basic Conitions Of Uurban Households（2017）

10—11 城镇居民家庭基本情况（2018 年） …… 203
Basic Conitions Of Uurban Households（2018）

10—12 农村居民家庭基本情况（2015 年） …… 204
Basic Conditions Of Rural Households（2015）

10—13 农村居民家庭基本情况（2016 年） …… 207
Basic Conditions Of Rural Households（2016）

10—14 农村居民家庭基本情况（2017 年） …… 206
Basic Conditions Of Rural Households（2017）

10—15 农村居民家庭基本情况（2018 年） …… 207
Basic Conditions Of Rural Households（2018）

10—16　全体居民家庭消费构成 ………………………………………………………… 208
Household Consumption Structure
10—17　城镇居民家庭消费构成 ………………………………………………………… 209
Composition Of Urban Households Consumption
10—18　农村居民家庭消费构成 ………………………………………………………… 210
Composition Of Rural Households Consumption
10—19　城镇住户每百户家庭主要耐用品拥有量（ 1980-2018 年） ………………………… 211
Ownership Of Major Durable Consumer Goods Per 100 Urban Households（1980-2018）
10—20　农村住户每百户家庭主要耐用品拥有量（1985-2018 年） ………………………… 212
Ownership Of Major Durable Consumer Goods Per 100 Rural Households（1985-2018）
主要统计指标解释 ………………………………………………………………… 213
Explanatory Notes on Main Statistical Indicators

十一、农　业
Chapter 11.　AGRICULTURE

简要说明 ………………………………………………………………………… 216
Brief Introduction
11—1　农村基本情况（2000-2018 年） ………………………………………………… 217
Basic Statistics On Rural Area（2000-2018）
11—2　农村劳动力（1985-2018 年） …………………………………………………… 219
Rurral Labor Firce（1985-2018）
11—3　分市、区乡村户数、人口、劳动力（2018 年） ………………………………… 221
Rural HousehoIda，Population And Labor Force By Region（2018）
11—4　主要年份农、林、牧、渔业总产值（按现价计算） ………………………………… 222
Major Year’s Gross Output Value Of Farming，Forestry，Aninal Husbandry And Fishery（Currentprice）
11—5　分市、区农、林、牧、渔业总产值（2018 年，现价） ……………………………… 223
Gross Output Value Of Farming，Forestry，Animal Husbandry And Fishery By Region（2018，Current Price）
11—6　农、林、牧、渔业总产值、增加值（2018 年） ………………………………… 224
Value-Added Of Farming，Forestry，Animal，Husbandry And Fishery（2018）
11—7　分市、区农、林、牧、渔业增加值（2018 年） ………………………………… 224
Value-Added Of Farming，Forestry，Animal Husbandry Fishery By Region（2018）
11—8　主要年份耕地面积与播种面积 ………………………………………………… 225
Major Year’s Cultivated And Sown Area
11—9　分市、区耕地面积（2018 年） ………………………………………………… 226
Area Of Cultivted Land By Region（2018）

11—10 分市、区农作物播种面积（2018 年）…… 227
Sown Area Of Farm Crops By Region（2018）
11—11 分市、区部分农作物产量（2018 年）…… 228
Output Of Farm Crops By Region（2018）
11—12 分市、区部分农作物播公顷单产量（2018 年）…… 229
Output Of Farm Crops Per Hectare By Region（2018）
11—13 主要年份农作物总产量 …… 230
Major Year's Ouptut Of Farm Crops
11—14 分市、区猪、羊及家禽存养量（2018 年）…… 232
Hogs，Sheep，Goats And Pputltry In Stock By Region（2018）
11—15 分市、区肉、蛋、奶产量（2018 年）…… 232
Output Of Meat，Eggs And Milk By Region（2018）
11—16 分市、区渔业养殖面积 …… 233
Aquaculture Area By Region
11—17 分市、区水产品总产量（2018 年）…… 233
Output Of Aquatic Products By Region（2018）
11—18 分市、区植树及造林面积（2018 年）…… 234
Output Of Aquatic Products By Region（2018）
11—19 分市、区水果产量（2018 年）…… 234
Area Of Forestation And Output Of Fruits By Region（2018）
11—20 分市、区果园、茶园面积和茶叶产量（2018 年）…… 235
Area Of Orchard And Tea Garden And Production Of Tea By Ctty And District（2018）
11—21 主要年份主要农业机械拥有量 …… 236
Major Year's Ownershiip Of Agricutural Machinery
11—22 主要年份农业机械化用电量、化肥施用量 …… 237
Major Year's Mechanization，Electricity And Chemical Fertilizer Consumption
11—23 分市、区农业机械化和电气化（2018 年）…… 238
Mechanization And Electritication In Agriculture By Region（2018）
11—24 分市、区农用化肥施用量（2018 年）…… 238
Consumption Of Chemical Fertilizers By Region（2018）
11—25 分市、区农田水利（2018 年）…… 239
Farmland Water Conservancy By Region（2018）
11—26 分市、区主要农业机械拥有量（2018 年）…… 240
Ownership Ofmajor Agricultural Machinery By Rgion（2018）
主要统计指标解释 …… 241
Explanatory Notes on Main Statistical Indicators

十二、工　业
Chapter 12.　INDUSTRY

简要说明 …… 244
Brief Introduction

12—1　规模以上工业企业单位数 …… 245
Number Of Industrial Enterprises Above Designateo Size

12—2　分市、区规模以上工业企业数（2018 年） …… 251
Number Of All Industrial Enterprises By Region（2018）

12—3　历年全部工业总产值 …… 252
Gross Industrial Output Value Over The Years

12—4　分市、区规模以上工业总产值（2018 年） …… 256
Gross Industrial Output Value Above Designated Sfze By Region（2018）

12—5　规模以上工业企业主要指标（2018 年） …… 260
Man Indicators Ofindustrial Enterprises Above Designated Sixe（2018）

12—6　按行业分国有及国有控股工业企业主要指标（ 2018 年） …… 268
Main ndicators Of State-Owned And State-Holding Industrial Enterprises By Industrial Sector（2018）

12—7　按行业分规模以上外商投资和港澳台商投资工业企业主要指标（ 2018 年） …… 272
Main Indicators Of Foreign Funede Enteprises And Enterprises With Funds From Hond Kong，Macao And Taiwan Above Designated Size By Industrial S ector（2018）

12—8　按行业分大中型工业企业主要指标（ 2018 年） …… 276
Main Indicators Of Large And Medium-Sized Industrial Enterprises By Industrial Sector（2018）

12—9　按行业分规模以上工业企业主要经济效益指标（2018 年） …… 280
Main Indicators On Economic Benefit Of Industria Enterprises Above Designated Size By Indoustrial Ssector（2018）

12—10　按行业分国有控股工业企业主要经济效益指标（2018 年） …… 282
Main Indicators On Economic Benefit Of Stste-Owned And State-Holding Inustrial Enterprises By Industrial Sector（2018）

12—11　按行业分规模以上外商及港澳台商投资工业企业主要经济效益指标（2018 年） …… 284
Main Indicators On Economic Benefit Of Foreign Funded Enterprises And Enterprises With Funds From Hong Kong，Macao And Taiwan Above Designated Size By Industrial Sector（2018）

12—12　按行业分大中型工业企业主要经济效益指标（2018 年） …… 286
Main Indicators On Economic Bemefit Of Large And Medicm-Sized Industrial Enterprises By Industrial Sector（2018）

12—13　主要年份主要工业产品产量 …… 288
Major Year’s Products Output Of Industry Above Designated Size

12—14　规模以上工业主要产品产量 …… 294
Output Of Major Industrial Products Of Industry Above Designated Size
12—15　规模以上工业主要产品生产能力 …… 297
Production Capacity Of Major Products Of Industry Above Designated Size
主要统计指标解释 …… 298
Explanatory Notes on Main Statistical Indicators

十三、建　筑　业
Chapter 13.　CONSTRUCTION

简要说明 …… 300
Brief Introduction
13—1　建筑业企业生产情况（2018 年） …… 301
Production Situation Of Construction Enterprises（2018）
13—2　建筑业企业财务状况（ 2018 年） …… 302
Financial Situation Of Construction Entrprises（2018）
13—3　建筑业企业主要经济效益指标（ 2018 年） …… 304
Main Inaicators On Economic Benefit Of Construction Enterprises（2018）
13—4　建筑业增加值（ 2018 年） …… 305
Value Added Of Construction（2018）
13—5　重点建筑企业一览表（ 2018 年） …… 306
List Of Key Construction Enterprises（2018）
主要统计指标解释 …… 309
Explanatory Notes on Main Statistical Indicators

十四、运输、邮电
Chapter 14.　TRANSPORT，POSTAL AND TELECOMMUNICATION SERVICES

简要说明 …… 312
Brief Introduction
14—1　主要年份客货运输及港口吞吐量 …… 313
Major Year’s Passenger & Freight Traffic And Handling Capacity Of The Ports
14—2　民用车辆拥有量（2018 年底） …… 314
Rossession Of Civil Motor Vehicles（End Of 2018）
14—3　客货运输及港口吞吐量（2018 年） …… 315
Passenger & Freicht Traffic And Handling Capacity Of The Ports（2018）

14—4　规模以上运输邮电单位主要财务指标（2018 年）…… 315
Major Financial Indicators Of Transportation，Post And Telecommunication Units Above Designated Scale（2018）
14—5　邮电通讯基本情况（2018 年）…… 316
Basic Conditions Of Postal And Telecommunication Services（2018）
14—6　邮政行业发展情况 …… 317
Development Of The Postal Services
14—7　快递企业发展情况 …… 318
Express Company Development
14—8　电信行业基本情况 …… 319
Basic Situation Of Telecom Industry
14—9　城市轨道交通发展情况 …… 321
Development Of Urban Rail Transit
主要统计指标解释 …… 322
Explanatory Notes on Main Statistical Indicators

十五、批发和零售业
Chapter 15.　WHOLESALE AND RETAIL TRADES

简要说明 …… 326
Brief Introduction
15—1　社会消费品零售总额（1985–2018 年）…… 327
Total Retail Sales Of Consumer Goods（1985–2018）
15—2　分市、区社会消费品零售总额（2018 年）…… 328
Total Retail Sales Of Consumer Goods By Region（2018）
15—3　限额以上批发和零售业商品购销存总额（2018 年）…… 329
Total Purchases，Sales And Stock Of Enterprises Above Designated Size Of Wholesale And Retail Trades（2018）
15—4　限额以上批发和零售业商品分类销售额（2018 年）…… 330
Sales Value Of Enterprises Above Designated Size Of Wholesale And Retail Trades By Category Of Commodities（2018）
15—5　限额以上批发业财务状况（2018 年）…… 331
Financial Position Of Enterprises Above Designated Size Of Wholesale Trade（2018）
15—6　限额以上零售业财务状况（2018 年）…… 332
Financial Position Of Enterprises Above Designated Size Of Retail Trade（2018）

15—7　分市、区城乡亿元商品交易市场分布情况（2018 年）…… 333
Basic Statistics On Commodity Exchange Markets Of Transaction Vaiue Over 100 Nillion Yuan By Region（2018）
主要统计指标解释 …… 334
Explanatory Notes on Main Statistical Indicators

十六、住宿、餐饮业和旅游
Chapter 16.　HOTELS，CATERING SERVICES AND TOURISM

简要说明 …… 336
Brief Introduction
16—1　限额以上住宿和餐饮业法人企业经营情况（2018 年）…… 337
Business Conditions Of Enterprises Of Hotels And Catering Services Above Designated Size（2018）
16—2　限额以上住宿业财务状况（2018 年）…… 338
Financial Situation Of Hotels Above Designated Size（2018）
16—3　限额以上餐饮业财务状况（2018 年）…… 339
Financial Situation Of Catering Services Above Designated Size（2018）
16—4　入境旅游人数（ 2000－2018 年）…… 340
Number Of Oversea Visitor Arrivals（2000–2018）
16—5　入境旅游收入（2000–2018 年）…… 340
Earnings From International Tourism（2000–2018）
16—6　国内旅游人数及收入（ 2018 年）…… 342
Number Of Dome Sttc Tourism Income（2018）
主要统计指标解释 …… 343
Explanatory Notes on Main Statistical Indicators

十七、教育、科技和文化
Chapter 17.　EDUCATION SCIENCE & TECHNOLOY AND CULTURE

简要说明 …… 346
Brief Introduction
17—1　各级各类学校基本情况（ 2018 年）…… 347
Basic Statistics On Schools By Level And Type Of School（2018）
17—2　主要年份各级各类学校在校学生数 …… 348
Major Year’s Students Enrollment Of Schools By Level And Type Of School

17—3　主要年份普通高等学校基本情况 …… 349
Major Year’s Basic Statistics On Regular Insttutions Of Higher Education
17—4　各类成人教育基本情况（2018 年）…… 350
Basic Statistics On Adult Education（2018）
17—5　分市、区普通中学情况（2018 年）…… 351
Basic Statistics On Regular Secondary Schools By Region（2018）
17—6　分市、区职业中学、小学情况（2018 年）…… 352
Basic Statistics On Vocaional Secondary Schools And Primary School Sby Region（2018）
17—7　分市、区中小学教职工情况（2018 年）…… 353
Basic Statistics On Teachers And Staff In Secondary And Primary Schools By Region（2018）
17—8　分市、区幼儿园基本情况（2018 年）…… 354
Basic Statistics On Kindergartens By Region（2018）
17—9　科研机构基本情况（1978–2018 年）…… 355
Basic Statistics On Scientific Research Institutions（1978–2018）
17—10　独立科学研究机构情况（2018 年）…… 356
Basic Statistics On Independent Inshutions Of Scientitic Research（2018）
17—11　科学技术奖励情况（2018 年）…… 356
Award Statistics On Science And Technology（2018）
17—12　文化、文物事业机构、人员数（2018 年）…… 357
Number Of Institutions And Personnel In Cuuture（2018）
17—13　文化部门艺术剧团情况（2018 年）…… 358
Statistics On Art Troupes Of Cultural Department（2018）
17—14　图书馆、文化馆情况 …… 358
Statistics On Libraries And Cultural Centers
主要统计指标解释 …… 359
Explanatory Notes on Main Statistical Indicators

十八、体育、卫生和民政、司法

Chapter 18.　SPORTS，PUBLIC HEALTH ANDCIVIL AFFAIRS，JUDICIAL AFFAIRS

简要说明 …… 362
Brief Introduction
18—1　体育事业情况（2000–2018 年）…… 363
Statistics On Sports（2000–2018）
18—2　主要年份卫生事业基本情况 …… 365
Major Year’s Basic Statistics On Public Health

18—3　各类卫生机构、床位、人员数（2018 年底）………………………………………… 366
Number Of Health Institutions，Beds And Employed Persons（End Of 2018）
18—4　分市、区各类卫生机构、床位、人员数（2018 年底）………………………………… 368
Number Of Health Institutions，Beds And Employed Persons By Region（End Of 2018）
18—5　收养性社会福利单位情况（2018 年）………………………………………………… 372
Basic Statictics On Social Welfare Institutions（2018）
18—6　社会救济情况（2018 年）……………………………………………………………… 372
Basic Statistics On Soclal Relief（2018）
18—7　分市、区婚姻登记情况（2018 年）…………………………………………………… 374
Basic Statistics On Marriage Registration By Region（2018）
18—8　律师、公证、调解、社会治安基本情况（2000–2018 年）…………………………… 376
Basic Statistics On Law Yers，Notarization，Mediation And Social Order（2000–2018）
18—9　分区、市殡葬服务情况（2018 年）…………………………………………………… 378
Basic Statistics On Funeral Services（2018）
主要统计指标解释 ……………………………………………………………………… 380
Explanatory Notes on Main Statistical Indicators

附　录
APPENDIX

2018 年省内各市主要经济指标对比情况 …………………………………………………… 382
Major Economic Indicators On Ctties Of The Province（2018）
2018 年十五个副省级城市主要经济指标对比表 ………………………………………… 384
Major Economic Indicators On Ctties Under Ppovincial Levels（2018）
2018 年副省级城市之外部分城市主要经济指标情况 …………………………………… 386
The Main Economic Indicators Of Some Other Cities Than The Sub–Provincial City For（2018）
青岛西海岸新区统计指标表………………………………………………………………… 387
Qingdao West Coast New District Statistical Indicator Table
青岛改革开放以来主要指标数据…………………………………………………………… 388
Main Indicator Data Since Qingdao's Reform And Opening Up

2018 年
青岛市国民经济和社会发展
统 计 公 报

青岛市统计局

国家统计局青岛调查队

（2019 年 3 月 19 日）

2018 年，全市坚持以习近平新时代中国特色社会主义思想为指导，深入贯彻习近平总书记视察山东、视察青岛重要讲话、重要指示批示精神，在市委、市政府坚强领导下，坚持稳中求进工作总基调，贯彻新发展理念，落实高质量发展要求，以新旧动能转换重大工程为引领，统筹推进稳增长、促改革、调结构、惠民生、防风险，经济社会保持持续健康发展。

一、综　合

初步核算，2018 年全市生产总值 12001.5 亿元，按可比价格计算，增长 7.4%。其中，第一产业增加值 386.9 亿元，增长 3.5%；第二产业增加值 4850.6 亿元，增长 7.3%；第三产业增加值 6764.0 亿元，增长 7.7%。三次产业比例为 3.2：40.4：56.4。人均 GDP 达到 128459 元。

图 1：2015-2018 年全市生产总值及增速

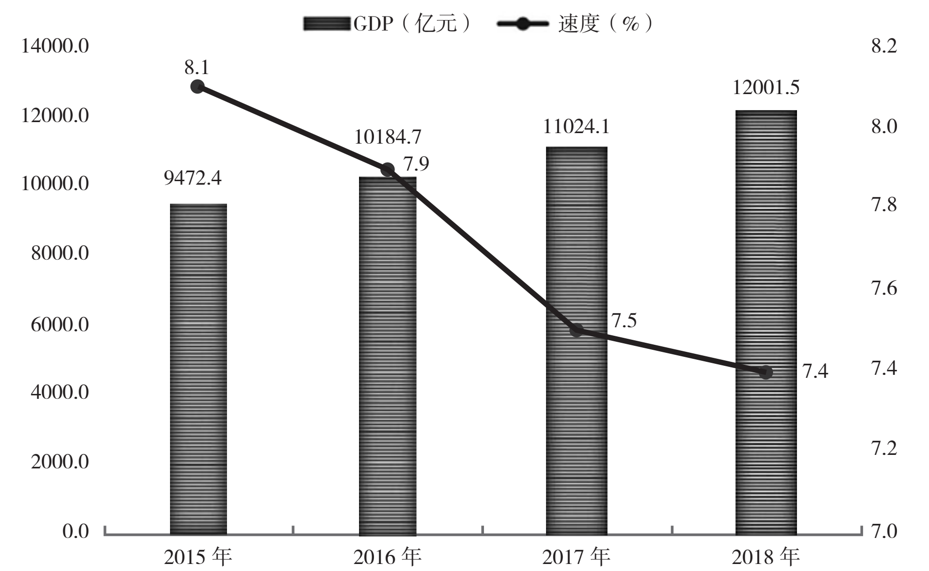

注：2015 年全市生产总值和增速为研发支出计入 GDP 核算修订后数据；2016 年 -2017 年为研发支出计入 GDP 核算并根据第三次农业普查修订后数据。

年末全市常住总人口 939.48 万人，增长 1.1%。其中，市区常住人口 635.25 万人，增长 1.6%。

表 1：2018 年全市及区市常住人口情况

区　市	年末数（万人）
全 市	939.48
市南区	58.83
市北区	110.11
李沧区	57.74
崂山区	44.59
西海岸新区	157.73
城阳区	72.05
即墨区	123.83
胶州市	90.05
平度市	137.89
莱西市	76.29
红岛经济区	10.37

全年财政总收入 3705.5 亿元，增长 15.0%。一般公共预算收入 1231.9 亿元，增长 6.5%。其中，税收收入 905.9 亿元，增长 9.9%；增值税 327.9 亿元，增长 6.1%；企业所得税 152.4 亿元，增长 3.9%；个人所得税 48.8 亿元，增长 15.1%；城市维护建设税 57.7 亿元，增长 9.5%。一般公共预算支出 1561.2 亿元，增长 11.3%。其中，一般公共服务支出 162.7 亿元，增长 6.9%；教育支出 263.0 亿元，增长 3.6%；科学技术支出 45.1 亿元，增长 17.0%；社会保障和就业支出 156.1 亿元，增长 1.8%；城乡社区事务支出 332.0 亿元，增长 3.0%。全年税务系统组织税收收入（含海关代征）2409.0 亿元，增长 6.3%。其中，国内税收 1720.1 亿元，增长 8.0%。

图 2：2015-2018 年全市一般公共预算收入及增速

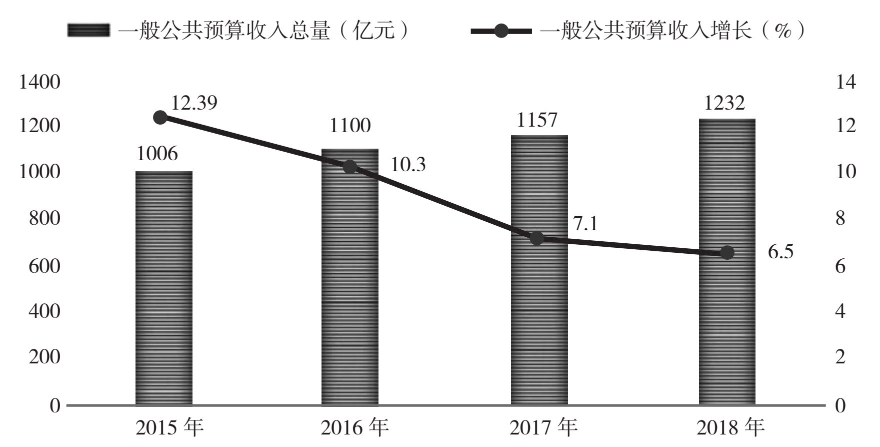

全年居民消费价格指数为 102.1，工业生产者出厂价格指数为 104.22，工业生产者购进价格指数为 105.22。12 月新建商品住宅销售同比价格指数为 113.3，二手住宅同比价格指数为 111.2。

表 2：2018 年全市居民消费价格指数

指标名称	全年（上年同期 =100）
居民消费价格总指数	102.1
非食品价格指数	101.9
服务价格指数	101.6
消费品价格指数	102.4
一、食品烟酒	102.9
食品	103.1
# 粮食	100.5
鲜菜	114.0
猪肉	92.4
鸡蛋	111.7
二、衣着	103.8
三、居住	100.9
四、生活用品及服务	101.3
五、交通和通信	100.8
六、教育文化和娱乐	103.1
七、医疗保健	102.4
八、其他用品和服务	101.3

重点领域改革取得新成效。争取国家、省改革试点任务 59 项。大力推进一流营商环境提升年行动，市级行政审批事项和权力事项分别精简 62.6% 和 55.3%，“一次办好”基本实现全覆盖，市级审批事项网上办理率达到 94%，市行政审批服务大厅全面实行“一窗受理”。户籍制度改革取得新成效，人才落户、居住落户、项目落户、积分落户更加便捷。全部完成 6015 个村集体资产清产核资任务，完成 5550 个村集体产权制度改革。

“三去一降一补”取得新进展。年末商品房待售面积 433.3 万平方米，下降 7.8%；其中，住宅待售面积 162.1 万平方米，下降 22.8%。全市规模以上工业企业每百元主营业务收入中的成本为 81.9 元，与上年持平。全面落实国家减税降费政策，全年为企业新增减负 147 亿元。农业投资增长 15.8%，教育投资增长 14.8%，基础设施投资增长 15.7%。

“三大攻坚战”稳步推进。全市银行不良贷款率降至 1.87%，较年初下降 0.07 个百分点。各级共安排财政扶贫资金

3.3亿元。实施动态调整机制，年末“脱贫享受政策”建档立卡贫困人口为29531人。新建产业扶贫项目111个，累计落地项目达800个，累计资产收益超过6000万元。主要污染物细颗粒物（PM2.5）、二氧化硫、二氧化氮年均值分别为34、10、31微克/立方米，与上年相比，PM2.5改善8.1%，二氧化硫改善28.6%，二氧化氮改善6.1%。

二、新动能

初步核算，全年实现海洋生产总值3327亿元，增长15.6%，海洋生产总值占GDP比重27.7%。其中，海洋第一产业增加值110亿元，增长5.1%；第二产业增加值1766亿元，增长18%；第三产业增加值1451亿元，增长13.7%。其中，滨海旅游业、海洋交通运输业、海洋设备制造业和涉海产品及材料制造业等4个支柱产业共实现增加值2025亿元，增长14.5%，占海洋经济比重60.9%。海洋新兴产业实现增加值366亿元，增长8.6%，占海洋经济比重11%。

“新经济”增加值3065.8亿元，占全市生产总值比重25.5%。“新产业”稳步发展，全年全市高技术产业实现增加值增长6.9%，占GDP比重为4.6%。其中，高技术制造业增加值增长3.3%，占GDP比重为2.0%；高技术服务业增加值增长10.0%，占GDP比重为2.5%。战略性新兴产业增加值增长6.2%，占GDP比重为8.1%。其中，战略性新兴产业工业增加值增长5.0%，占GDP比重为5.4%；战略性新兴产业服务业增加值增长8.6%，占GDP比重为2.7%。“新业态、新模式”显现活力，全市电子商务交易平台实现交易额1.0万亿元，限额以上批发和零售业单位网络零售额增长27.6%。“新技术”发展迅速，全市规模以上高技术服务业企业营业收入占规模以上服务业企业收入比重提升至22.8%。“新产品”产量增速较快，工业机器人2288套，增长59.4%；城市轨道车辆1538辆，增长20.5%；新能源汽车9.2万辆，增长14.4%。新动能投资拉动明显。装备制造业投资增长10.7%，战略新兴产业投资增长32.8%，高技术制造业投资增长59.0%，工业技改投资增长22.6%。

初步统计，全市共获得国家级科技奖励14项。其中，自然科学奖1项，技术发明奖1项，国际科学技术合作奖1项，科技进步奖11项。技术合同交易额155.8亿元，增长23.0%。涉海技术合同成交额18.2亿元，增长21.2%。全年有效发明专利26267件，增长20.5%。每万人有效发明专利拥有量28.5件，PCT国际专利申请1088件。国家知识产权示范企业10家，国家知识产权优势企业60家，国家技术创新示范企业14家。国家企业技术中心39家，高新技术企业总数达到3112家。杰华生物成为全省首家独角兽企业。研发力量加快聚集，国家重点实验室达到9家，省级以上企业技术中心达到193家，工程（技术）研究中心达到292家。

三、农　业

全年农业增加值410.6亿元，增长3.6%。其中，种植业增加值194.0亿元，增长3.0%；林业增加值2.3亿元，增长11.9%；牧业增加值76.1亿元，增长7.9%；渔业增加值114.5亿元，增长2.2%。

全年粮食播种面积48.1万公顷，蔬菜播种面积11.3万公顷，花生播种面积8.0万公顷。粮食总产量310.1万吨，增长4.4%；蔬菜及食用菌总产量644.4万吨，增长2.6%；花生总产量38.5万吨，增长0.3%；水果（含果用瓜）总产量109万吨，下降8.0%。现代农业园区987个，新增51个；认证“三品一标”（无公害农产品、绿色食品、有机农产品和农产品地理标志）产品1056个，其中国家地理标志保护产品51个。

全年新增造林13.5万亩，人工更新造林1.7万亩。

全年肉类总产量52.2万吨，增长3.0%；禽蛋产量18.2万吨，下降13.8%；奶类产量29.8万吨，增长10.3%。

全年水产品产量（不包括远洋捕捞）103.5万吨，增长1.6%。海、淡水养殖面积3.5万公顷，增长0.9%。远洋捕捞量14.5万吨，与上年持平。

农机总动力737.8万千瓦，增加9.9万千瓦。农作物生产综合机械化水平达到87.8%。农田有效灌溉面积33.1万公顷，其中节水灌溉面积18.8万公顷。

表 3：2018 年主要种植业产品产量及增速

指标	单位	产量	增长（%）
粮食	万吨	310.1	4.4
夏粮	万吨	137.7	8.9
秋粮	万吨	172.4	1.1
油料	万吨	38.5	0.3
蔬菜及食用菌	万吨	644.4	2.6
水果	万吨	109	−8.0
园林水果	万吨	71	−5.1

四、工业与建筑业

全市全部工业增加值 4137.1 亿元，增长 6.9%。其中，规模以上工业增加值增长 6.8%。轻工业增加值增长 8.5%，重工业增加值增长 6%。分经济类型看，国有控股企业增加值增长 4.8%，集体企业增加值增长 2.8%，股份制企业增加值增长 7.0%，外商及港澳台商投资企业增加值增长 6.3%。分门类看，采矿业增加值下降 14.5%，制造业增加值增长 6.6%，电力、热力、燃气及水生产和供应业增加值增长 11.8%。全年规模以上工业企业产销率达到 99.9%。规模以上工业企业实现出口交货值增长 8.0%，比上年提高 3.2 个百分点。

表 4：2018 年规模以上工业主要产品产量及增速

产品名称	单位	产量	增长（%）
彩色电视机	万台	1695.2	−0.5
其中：智能电视	万台	1695.2	−0.5
家用电冰箱	万台	886.6	3.3
家用洗衣机	万台	601.7	−0.3
房间空气调节器	万台	1058.1	5.3
移动通信手持机	万台	2010.2	−28.9
橡胶轮胎外胎	万条	6388.8	36.6
平板玻璃	万重量箱	561.5	0.7
粗钢	万吨	309.7	10.2
汽车	万辆	9.2	14.4
金属集装箱	万立方米	1385.5	18.6

规模以上工业利润增长 6%。其中，国有控股企业下降 6.9%，集体企业增长 33.3%，股份制企业增长 4.3%，外商及港澳台商投资企业增长 10.4%。

表 5：2018 年规模以上工业利润增速

指标	增长（%）
规模以上工业利润	6
其中：国有控股	-6.9
集体企业	33.3
股份制企业	4.3
外商及港澳台投资企业	10.4

全年资质内总承包专业承包建筑业企业 711 家，实现增加值 723.4 亿元，增长 10.5%；完成建筑业总产值 2309.3 亿元，增长 22.9%；签订合同额 4777.9 亿元，增长 18.0%；实现利税总额 105.6 亿元，增长 8.9%。

五、服务业

全市服务业增加值占全市生产总值比重为 56.4%，比上年提高 0.9 个百分点；对经济增长的贡献率为 55.6%。

表 6：2018 年全市服务业增加值情况

行 业	增加值（亿元）	增长（%）	比重（%）
农林牧渔服务业	23.65	6.6	0.2
金属制品、机械和设备修理业	9.89	6.9	0.1
批发和零售业	1440.66	7.3	12
交通运输、仓储和邮政业	830.58	2.7	6.9
住宿和餐饮业	268.18	5.7	2.2
金融业	800.4	5.2	6.7
房地产业	672.46	1.2	5.6
其他服务业	2718.2	12.2	22.7
#营利性服务业（主要包括电信、信居租文）	1416.11	19.6	11.8
#非营利性服务业	1302.09	4.8	10.9

全市规模以上服务业总体较快增长，实现营业收入 1957.7 亿元，增长 12.0%。生产性服务业中，商务服务业营业收入增长 29.5%，科技服务业营业收入增长 14.2%。生活性服务业中，影视节目制作营业收入增长 97.7%，电影和广播电视节目发行营业收入增长 12.7 倍，艺术表演场馆营业收入增长 15.8%，体育组织营业收入增长 32.0%。全市现代服务业实现增加值 3773.4 亿元，增长 9.2%，占全市 GDP 比重为 31.4%。

表 7：2018 年规模以上服务业营业收入情况

行 业	营业收入（亿元）	增长（%）
规模以上服务业	1957.7	12.0
交通运输、仓储和邮政业	1006.8	9.0
信息传输、软件和信息技术服务业	306.8	12.2
房地产业（不含房地产开发经营）	31.3	10.8
租赁和商务服务业	306.3	27.4
科学研究和技术服务业	142.9	6.8
水利、环境和公共设施管理业	27.4	−29.3
居民服务业、修理和其他服务业	22.5	−4.2
教育	20.9	14.2
卫生和社会工作	18.6	13.1
文化、体育和娱乐业	74.2	27.1

交通运输、仓储和邮政业平稳发展。全年港口吞吐量 5.4 亿吨，增长 6.1%；外贸吞吐量 3.9 亿吨，增长 6.2%；集装箱吞吐量 1932 万标准箱，增长 5.5 %。年末拥有航线 192 条，增长 3.2%。其中，国内航线 160 条，国际航线 29 条，港澳台地区航线 3 条。全年航空旅客吞吐量达到 2454 万人次，增长 5.7%；航空货邮吞吐量 22 万吨，下降 3.2%。年末全市机动车保有量 282.6 万辆，增长 8.1%；其中，私人汽车 232.1 万辆，增长 9.4 %。全年地铁开行列车 33 万列次，总运营里程 915 万列公里，总客运量 15294 万人次。全年完成邮电业务总量 650.9 亿元，增长 105.2%。其中，邮政业务总量 82.9 亿元，增长 14.4%；电信业务总量 568 亿元，增长 132.1%。快递业务量 3.8 亿件，增长 23%。固定宽带互联网用户 398 万户，增长 7.9%。全市移动电话 1259 万户，其中 3G 和 4G 用户 1064 万户，增长 8.7%。

表 8：2018 年各种运输方式完成的运输量及增速

运输方式	单位	运输量	增长(%)
客运周转量	亿人公里	182.49	6.4
铁路	亿人公里	105.36	8.7
公路	亿人公里	76.97	3.4
水运	亿人公里	0.16	-27.2
货运周转量	亿吨公里	1647.12	9.3
铁路	亿吨公里	190.90	4.3
公路	亿吨公里	532.20	3.2
水运	亿吨公里	924.02	14.4

金融业健康发展。年末金融机构本外币存款余额 16121.3 亿元，比年初增加 992.3 亿元；人民币存款余额 15532.2 亿元，比年初增加 1144.4 亿元，其中，住户存款 5913.7 亿元，比年初增加 518.6 亿元。本外币贷款余额 16098 亿元，比年初增加 1692.9 亿元；人民币贷款余额 15194.2 亿元，比年初增加 1923.8 亿元。保险业实现保费收入 439.4 亿元，增长 10.8%。其中，产险公司实现保费收入 132.8 亿元，增长 20%；寿险公司实现保费收入 306.6 亿元，增长 7.2%。证券经营机构代理成交额 27268.1 亿元，下降 22.7%。年末私募基金机构 236 家，实缴管理基金规模 640.4 亿元，增长 30.9%。

旅游业快速发展。全市共接待国内外游客 1.0 亿人次，增长 15%；实现旅游消费总额 1867.1 亿元，增长 13.8%。接待入境游客 153.6 万人次，增长 6.4%；实现旅游消费 11.6 亿美元，增长 14%。接待国内游客 9848.9 万人次，增长 13.7%；实现旅游消费 1651 亿元，增长 13.8%。年末拥有 A 级旅游景区 122 处，其中，5A 级旅游景区 1 处，4A 级旅游景区 26 处，3A 级旅游景区 74 处。拥有星级酒店 99 个，其中，5 星级酒店 10 个，4 星级酒店 25 个，3 星级酒店 60 个。拥有旅行社 544 个，其中，经营出境旅游业务旅行社 59 个，经营入境和国内旅游业务旅行社 544 个。

六、固定资产投资

全市固定资产投资增长 7.9%。其中，第一产业投资增长 14.3%，第二产业投资增长 9.1%，第三产业投资增长 7.5%。亿元以上新开工项目（含房地产）702 个，较上年增加 1 个，完成投资 1737.5 亿元，下降 12.4%，占全市固定资产投资的比重为 33.2%。

全年房地产开发完成投资 1485.2 亿元，增长 11.6%。其中，住宅投资 1034.8 亿元，增长 11.8%。商品房销售面积 1808 万平方米，下降 4.9%。其中，住宅销售面积 1578.3 万平方米，下降 3.4%。

表 9：2018 年全市房地产开发投资、商品房销售面积及增速

分 类	房地产开发投资（亿元）	增长（%）	商品房销售面积（万平方米）	增长（%）
总计	1485.2	11.6	1808	-4.9
住宅	1034.8	11.8	1578.3	-3.4
办公楼	98.5	-12.5	76.7	-21.6
商业营业用房	153.7	-17.3	119.9	-7.9
其他	198.2	85.7	33.1	-14.6

全年保障性住房基本建成15540套，其中，公共租赁住房11212套，经济适用住房2225套，限价商品住房2103套。截至年末，全市正在享受租赁补贴家庭共7326户，全年发放租赁补贴4367万元。

七、国内贸易

全年实现社会消费品零售额4842.5亿元，增长10.0%。按经营地统计，城镇消费品零售额4026.9亿元，增长9.7%；乡村消费品零售额815.6亿元，增长11.4%。分行业看，批发和零售业实现零售额4221亿元，增长9.1%；住宿和餐饮业实现零售额621.5亿元，增长16.6%。

全年限额以上单位实现消费品零售额1644.2亿元，增长9.1%。其中，粮油、食品类零售额增长17.5%，烟酒类零售额增长27.0%，服装、鞋帽、针纺织品类零售额增长8.6%，化妆品类零售额增长4.8%，金银珠宝类零售额增长11.5%，日用品类零售额增长19.1%，家用电器和音响器材类零售额增长18.5%，中西药品类零售额增长25.1%，文化办公用品类零售额增长10.1%，建筑及装潢材料类零售额增长12.3%，石油及制品类零售额增长9.8%，汽车类零售额比上年下降0.1%。

八、对外经济

全市实现外贸货物进出口总额5321.3亿元，增长5.7%。其中，出口额3172.2亿元，增长4.7%；进口额2149.0亿元，增长7.3%。对“一带一路”沿线国家市场进出口总额2590.4亿元，增长4.2%。其中，出口1630.6亿元，增长1.9%；进口959.8亿元，增长8.4%。

全市服务进出口176.1亿美元，增长9.6%。其中，出口87.5亿美元，增长5.6%；进口88.6亿美元，增长13.8%。全市承接服务外包合同8652份，增长43.9%；合同额56.7亿美元，增长39.7%；执行额44.3亿美元，增长18.3%。其中，离岸服务外包合同7275份，增长53.9%；合同额45.8亿美元，增长43.3%；执行额36.1亿美元，增长18.9%。

青岛口岸对外贸易进出口总额12196.8亿元，增长9.7%。其中，出口额6738.5亿元，增长6.5%；进口额5458.3亿元，增长13.9%。

表10：2018年全市主要商品进出口情况

项目	进出口		出口		进口	
	金额（亿元）	增长（%）	金额（亿元）	增长（%）	金额（亿元）	增长（%）
纺织服装	511.2	6.5	482.0	7.7	29.2	−9.8
农产品	797.7	11.8	387.3	8.4	410.4	15.2
机电产品	1786.8	1.6	1352.2	0.5	434.6	5.2
高新技术产品	525.2	5.1	316.1	5.1	209.1	5.1

表 11：2018 年对主要国家和地区货物进出口额及增速

国别（地区）	出口额（万美元）	增 长（%）	进口额（万美元）	增 长（%）
亚洲	2083063	9.8	1280932	5.3
香港	191991	9.2	11487	19.6
台湾	41522	8.5	118789	15.9
日本	562514	6.6	185504	3.8
韩国	444012	7.6	342617	12.8
东盟	463450	24.0	379222	-14.9
印度尼西亚	83850	39.7	63178	23.0
马来西亚	74747	-0.3	60583	-54.8
新加坡	44554	30.4	47902	14.5
泰国	76848	33.0	134631	-12.8
南亚	161294	16.2	38975	57.3
印度	100542	27.8	34467	60.0
中东	188352	-12.3	135723	82.5
非洲	192717	14.1	260346	44.9
南非	45146	12.9	26943	25.6
欧洲	1019206	-3.3	506724	29.5
欧盟	881451	7.2	290470	19.7
英国	140738	8.4	60435	52.8
德国	151262	3.2	85797	26.0
法国	85567	-0.8	36255	9.5
意大利	84584	10.4	21093	-0.3
独联体及东欧	119678	-44.5	183367	52.4
俄罗斯	89922	-52.9	174582	51.2
南美洲	317302	7.9	531235	-2.4
巴西	55242	30.3	374326	53.2
北美洲	1045878	13.7	316690	3.0
美国	930533	12.3	256098	-0.3
加拿大	109438	20.7	53394	21.7
大洋洲	135684	10.8	353478	13.1
澳大利亚	113291	11.8	298080	9.7

注：本表非全口径。

2018年，全市新批外商投资项目956个，增长47.1%；合同外资98.2亿美元，增长7.8%。全市实际使用外资86.9亿美元，增长10%；按商务部FDI（外商直接投资）统计口径，实际使用外资58.0亿美元，增长4.1%。

全市企业对“一带一路”沿线国家和地区投资项目22个，协议投资额2.0亿美元，增长8.9%；实际投资额6.8亿美元，增长86.9%。

全市对外承包工程业务新承揽项目107个，新签合同额49.3亿美元，增长11.9%；完成营业额43.1亿美元，增长12.8%。对外劳务合作业务派出各类劳务人员11820人。

九、社会事业

年末全市共有各类大专院校（含民办高校）25所，其中，普通高校23所。全年研究生招生1.6万人，在学研究生4.5万人，毕业生1.1万人（不含驻青科研院所研究生）。普通本专科招生10.2万人，在校生35.3万人，毕业生9.4万人。中等职业教育招生2.6万人，在校生8.4万人，毕业生2.8万人。普通高中招生3.8万人，在校生11.7万人，毕业生3.8万人。初中招生8.6万人，在校生26.8万人，毕业生7.2万人。普通小学招生10.4万人，在校生57.0万人，毕业生8.6万人。特殊教育招生0.02万人，在校生0.2万人，毕业生0.02万人。幼儿园在园幼儿26.4万人。

年末全市共有卫生机构（不含村卫生室）3745处，其中，医院、卫生院422处，疾病预防控制中心26处，妇幼保健机构12处，门诊部（所）、诊所、卫生所、医务室2917处。年末各类卫生技术人员8.4万人，其中，医生3.5万人。全市拥有医疗床位5.8万张，其中，医院、卫生院床位5.6万张。

全市运动员在各项比赛中共获得金牌353枚，银牌247枚，铜牌236枚。重点体校8所，学员1370人；业余体校10所，学员1846人。

十、城市建设

年末全市常住人口城镇化率达到73.67%，比上年提高1.1个百分点。城市平均每天供水量129.7万吨，增长2.9%。城市全年实际用水量4.1亿吨，其中，生产用水和生活用水分别为1.6亿吨和2.5亿吨。

城市使用液化气、天然气的总户数达到189.8万户，全年供应液化气总量2.7万吨，供应天然气总量10.7亿立方米。城市气化率达到100%。

全年新增供热面积1060.4万平方米，年末供热面积达到2亿平方米。

年末市区公共汽、电车线路623条，共有营运的公交汽、电车8475辆。市区共有巡游出租汽车10867辆。年末市区共有地铁线路4条，地铁年客运量15294.12万人次，日均客运量41.9万人次。

全市共有公共厕所788座，三类以上的774座，新增202座，改建54座。

年末城市下水道总长度8128.1公里。

十一、能源、环境和安全生产

初步统计，全年全市规模以上工业综合能源消费量1489万吨标准煤，增长4.1%。其中，煤炭消费量1360万吨，下降1.7%；原油消费量1542万吨，增长1.2%；天然气消费量10亿立方米，增长89.3%。全社会用电量432亿千瓦时，增长7.7%。其中，工业用电量238亿千瓦时，增长5.4%；城乡居民生活用电量77亿千瓦时，增长2.2%。

全年全市能源生产量 2834 万吨标准煤，增长 2.1%。其中，原油加工量 1541 万吨，增长 1.2%；汽油生产量 474 万吨，增长 3.9%；柴油生产量 383 万吨，下降 6.2%；发电量 192 亿千瓦时，增长 4.8%。新能源发电量 17 亿千瓦时，增长 13.5%。其中，风力发电量增长 12.6%，生物质发电量增长 25.4%。新能源发电量占全市发电总量比重为 9.1%，提高 0.7 个百分点。

全市年平均气温 13.3℃，平均年降水量为 722.7 毫米。市区空气质量优良天数达 307 天，占全年的 85%。近岸海域水质达到或好于二类海水水质标准的点位数占 90.6%，与上年持平。市区区域环境噪声平均值 56.9 分贝，市区交通干线噪声平均值 68.0 分贝。

全市七个行业（领域）发生各类生产安全事故 63 起，死亡 69 人，分别下降 42.2% 和 38.4%。亿元 GDP 生产安全事故死亡率 0.006，十万工矿商贸企业就业人员生产安全事故死亡率 0.72，道路交通事故万车死亡 1.13 人。

十二、人民生活和社会保障

全年全市居民人均可支配收入 42019 元，增长 8.4%。按常住地分，城镇居民人均可支配收入 50817 元，增长 7.7%；农村居民人均可支配收入 20820 元，增长 7.5%。全市城乡居民收入比为 2.44：1。全市居民人均消费支出 27316 元，增长 8.3%，恩格尔系数为 28.5%。按常住地分，城镇居民人均消费支出 32890 元，增长 7.6%；农村居民人均消费支出 13885 元，增长 7.4%。

表 12：2018 年城乡居民人均可支配收入构成情况

指标名称	全体居民		城镇居民		农村居民	
	绝对值（元）	增长（%）	绝对值（元）	增长（%）	绝对值（元）	增长（%）
可支配收入	42019	8.4	50817	7.7	20820	7.5
工资性收入	25303	7.6	30955	6.8	11684	7.4
经营净收入	8027	7.3	7836	7.4	8487	7.3
财产净收入	3475	12.8	4789	11.4	310	10.8
转移净收入	5214	11.3	7237	9.9	339	17.1

表 13：2018 年城乡居民人均消费支出情况

指标名称	全体居民		城镇居民		农村居民	
	绝对值（元）	增长（%）	绝对值（元）	增长（%）	绝对值（元）	增长（%）
消费支出	27316	8.3	32890	7.6	13885	7.4
食品烟酒	7787	4.0	9318	2.9	4098	5.9
衣着	2538	2.9	3168	1.7	1020	5.1
居住	6365	16.7	7751	17.3	3024	8.0
生活用品及服务	1837	5.1	2207	4.0	945	6.7
交通和通信	4084	8.8	4765	8.3	2445	8.1
教育文化娱乐	2571	9.5	3122	8.6	1245	10.3
医疗保健	1480	9.6	1764	8.6	794	10.6
其他用品和服务	654	4.6	795	3.4	314	6.9

表 14：2018 年每百户居民家庭主要耐用消费品拥有量

指标名称	单位	数 量		
		全体居民	城镇居民	农村居民
家用汽车	辆	56	62	43
摩托车	辆	28	15	61
电冰箱（柜）	台	108	108	109
洗衣机	台	99	100	97
热水器	台	97	101	88
空调	台	112	120	93
彩色电视机	台	107	107	105
计算机	台	70	81	45
移动电话	部	230	228	232

据抽样调查，年末城镇居民人均现住房建筑面积 32.9 平方米，农村居民人均现住房建筑面积 35.9 平方米。

全年城镇新增就业 77.6 万人，增长 6.3%。其中，本市劳动者就业增长 4.8%，外来劳动者来青就业增长 8.1%。服务业吸纳就业 50.7 万人，增长 16.9%。民营经济吸纳就业 59.6 万人，增长 11.3%。

年末全市城镇居民最低生活保障人数达 20457 人，城镇居民最低生活保障资金 19923 万元；农村特困供养人员 4843 人，农村特困供养支出 7624.5 万元。

年末全市城镇职工基本养老保险参保缴费人数为 273.51 万人，参加失业保险人数为 223.69 万人，全年累计领取失业保险金的人数为 7.5 万人。全市城镇登记失业率为 2.94%，降低 0.18 个百分点。

注：

1、公报中统计数据均为初步统计数。

2、全市生产总值（GDP）及各产业增加值绝对数按现价计算，增长速度按可比价计算。根据《三次产业划分规定》（国统字〔2012〕108号），农林牧渔业中的农林牧渔服务业，工业中的开采辅助活动以及金属制品、机械和设备修理业等三个行业划入第三产业。

3、常住人口包括：①住本户、户口在本乡镇街道的人（含户口在本户，外出不满半年的人）；②住本户半年以上，户口在外乡镇街道的人；③住本户不满半年，户口在外乡镇街道，离开户口登记地半年以上的人；④住本户，户口待定的人。

4、PCT（Patent Cooperation Treaty）是《专利合作条约》的英文缩写。PCT国际专利是指我国专利申请人根据PCT规定，可以向中国专利局或向世界知识产权组织提交国际专利申请，是反映地区外向型经济发展水平和地区国际技术竞争能力的重要指标。

5、战略性新兴产业根据国家规划，现阶段主要包括节能环保、新一代信息技术、生物、高端装备制造、新能源、新材料、新能源汽车等七个产业领域。

6、规模以上工业企业为年主营业务收入2000万元及以上企业。限额以上贸易企业为批发业年主营业务收入在2000万元及以上、零售业500万元及以上、住宿和餐饮业200万元及以上的单位。固定资产投资项目统计的起点标准为计划总投资额500万元。规模以上服务业企业，一是指年营业收入1000万元及以上或年末从业人员50人及以上服务业法人单位。包括：交通运输、仓储和邮政业，信息传输、软件和信息技术服务业，租赁和商务服务业，科学研究和技术服务业，水利、环境和公共设施管理业，教育，卫生和社会工作；以及物业管理、房地产中介服务、自有房地产经营活动和其他房地产业等行业。二是指年营业收入500万元及以上或年末从业人员50人及以上服务业法人单位。包括：居民服务、修理和其他服务业，文化、体育和娱乐业。

7、各类生产安全事故起数、死亡人数统计范围包括生产经营性道路交通、水上交通、铁路交通、民航飞行、农业机械、渔业船舶和工矿商贸七个行业（领域）发生的事故，不再包括森林火灾、火灾事故和非生产经营性道路交通。

8、供水、用水、供气、供热、道路、厕所、下水道等有关数据不含即墨区。

9、资料来源：本公报中财政数据来自市财政局，人才、登记失业率、社会保障数据来自市人力资源和社会保障局，银行数据来自人民银行青岛市中心支行，证券数据来自青岛证监局，保险数据来自青岛银保监局，水产品数据来自市海洋发展局，林业数据来自市园林和林业局，农机等数据来自市农业农村局，灌溉面积数据来自市水务管理局，交通运输数据来自市交通运输局，邮政数据来自市邮政管理局，固定电话、宽带、移动电话等数据来自市通信管理局，建成区面积、供水、供气、供热、道路、下水道等数据来自市住房城乡建设局，进出口、外资、对外承包工程、对外劳务合作、跨境电商等数据来自市商务局，旅游数据来自市文化和旅游局，教育数据来自市教育局，科技、专利数据来自市科技局，体育数据来自市体育局，卫生数据来自市卫生健康委，环境数据来自市生态环境局，用电量数据来自青岛供电公司，气象数据来自市气象局，安全生产数据来自市应急局，扶贫数据来自市扶贫开发办，汽车保有量来自市公安局，粮食、居民收支、价格、城乡居民住房面积等数据来自国家统计局青岛调查队，其他数据来自市统计局。

STATISTICS COMMUNIQUE QINGDAO'S ECONOMIC AND SOCIAL DEVELOPMENT IN THE YEAR OF 2018

Qingdao Statistics Bureau

NBS Qingdao Survey Office

(March 19，2019)

In 2018, the city takes Xi Jinping's new era of socialism with Chinese characteristics as the guide, in-depth implementation of General Secretary Xi Jinping's inspection of Shandong, inspection of Qingdao's important speech and important instructions, and under the strong leadership of the municipal government of the Communist Party of China , the overall tone of the work is maintained ， implement the requirements of high-quality development, take the lead in the transformation of new and old kinetic energy projects, and promote the steady growth, promote reform, adjust the structure, benefit the people's livelihood, prevent risks, and maintain sustained and healthy development in the economy and society.

Ⅰ .General

According to the preliminary accounting,annual gross domestic product 1.20015 trill-on yuan in 2018,caleulated at the comparable price,with an increase of 7.4%.Therein,the 38.69 billion yuan of added value of the first industry increased by 3.5 percent.The value-added 485.06 billion yuan of the second industry increased by 7.3.The value-added 676.40 billion yuan of the tertiary industry increased by 7.7%.The proportion of three industries is 3.2:40.4:56.4. GDP per capita reached 128459 yuan.

Figure1：Qingdao GDP and Growth Rate from 2015 to 2018

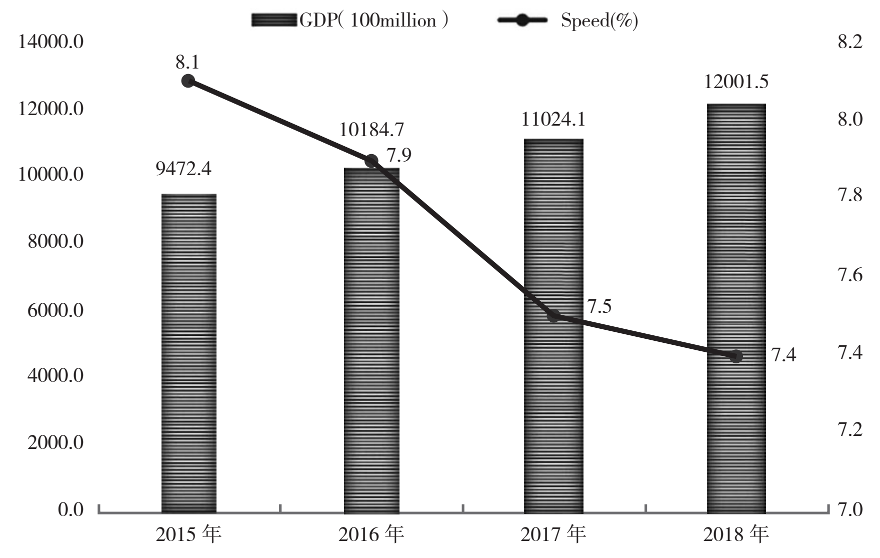

Note: The gross domestic product (GDP) and the growth rate in 2015 are the revised data of DGP input acounting for R & D expenditures

At the end of the year,the total population of the city is 939.48 million,with an inerease of 1.1%.Therein,the urban resident population is 635.25 million,with an increase of 1.6%.

Table 1：City resident population in 2018

Region	Number of end-of-year（10 000 persons）
Total	939.48
Shinan District	58.83
Shibei District	110.11
LIcang District	57.74
Laoshan District	44.59
West Coast New Area	157.73
Chengyang District	72.05
JImo District	123.83
Jiaozhou	90.05
Pingdu Laixi	137.89
Qingdao National High-tech	76.29
Industrial Development Zone	10.37

The total annual financial income is 370.55 billion yuan,inereased by 15.0% .The general publie budget income 123.19 billion yuan rises by 6.5%. Therein the total price is RMB 3.9 billion Yuan,with an increase of 9.9%.The VAT accounting to 32.79 billion yuan increased by 6.1%.The enterprise income tax accounts to 15.24 billion yuan,with an increase of 3.9%.The individual income tax accounts to 4.88 billion yuan,increased by 15.1%.The urban maintenance and construction tax,accounting to 5.77 billion yuan,increased by9.5%.The 156.12 billion yuan of general public budget expenditures increased by 11.3%.Of these,general public service expenditure 16.27 billion yuan rises by 6.9%.Educational expenditure accounts to 26.30 billion yuan, with an increase of 3.6%.The4.51 billion yuan of scientific and technological expenditure inereased by 17.0%.The 15.61 billion yuan of social security and employment expenditure increased by 1.8%.The 33.20 billion yuan of urban and rural community affairs expenditure increased by 3.0%.The annual nation tax system organization revenue, including customs collected as an agency, accounts 240.90 billion yuan, increased by 6.3%。Therein, the domestic tax revenue accounts to 172.01 billion yuan, increased by 8.0%.

Figure 2：General Public Budget Income and Speed-increasing in the City

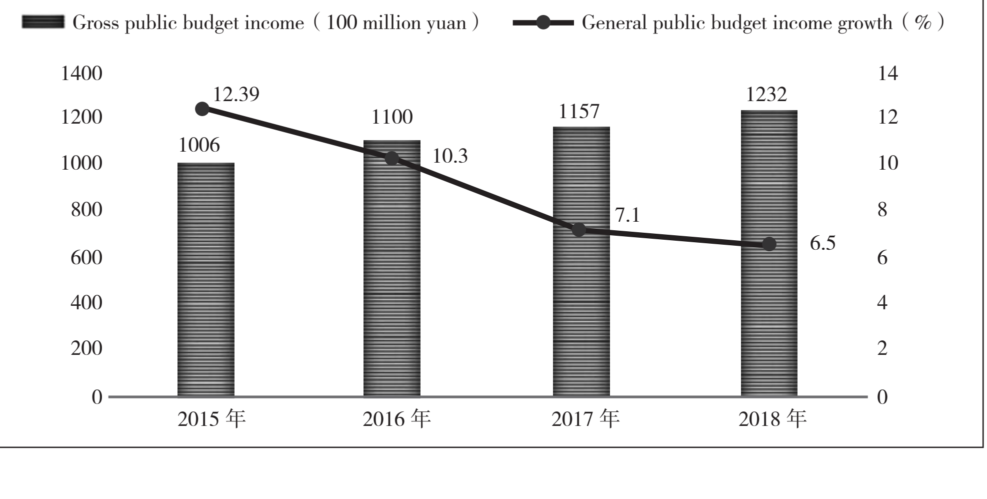

The annual consumer price index was 102.1, the industrial producer ex-factory price index was 104.22, and the industrial producer purchase price index was 105.22. The year-on-year price index for new commercial residential sales in December was 113.3, and the second-hand residential year-on-year price index was 111.2.

Table 2：Consumer Price Index of the City in 2018

Name	Number of end-of-year(10 000 persons)
General Consumer Price Index	102.1
Non-Food Price Index	101.9
Service Price Index	101.6
Consumer Price Index	102.4
1. Food，Tobacco and Liquor	102.9
Food	103.1
#Grain	100.5
Vegetables	114.0
Pork	92.4
Egg	111.7
2. Clothing	103.8
3. Residence	100.9
4. Supplies and Services	101.3
5. Transportation and Communication	100.8
6. Education，Culture and Recreation	103.1
7. Medical Care	102.4
8. Others	101.3

New achievements have been made in reforms in key areas. We will strive for 59 national and provincial reform pilot tasks. Vigorously promote the first-class business environment to improve the annual action, municipal administrative examination and approval matters and power matters were reduced by 62.6% and 55.3%, respectively, "one time to do a good job" to achieve full coverage, the city-level examination and approval matters online processing rate reached 94%, municipal administrative approval The service hall is fully implemented with "one window acceptance". The reform of the household registration system has achieved new results, and it has become more convenient for talents to settle down, settle in residences, settle projects, and settle in points. Completed the task of clearing assets and assets in 6015 village collective assets, and completed the reform of the collective property rights system of 5,550 villages.

"Three to one, one drop and one supplement" made new progress. At the end of the year, the saleable area of commercial housing was 4.333 million square meters, decreased 7.8%. Among them, the residential area for sale was 1.621 million square meters, decreased 22.8%. The cost per 100 yuan of main business income of industrial enterprises above designated size in the city was 81.9 yuan, which was the same as the previous year. Full implementation of the national tax reduction and fee reduction policy, the company's new annual burden reduction of 14.7 billion yuan. Agricultural investment increased by 15.8%, education investment increased by 14.8%, and infrastructure investment increased by 15.7%.

The "three major battles" have steadily advanced. The non-performing loan ratio of the city's banks fell to 1.87%, a decrease of 0.07 percentage points from the beginning of the year. A total of 330 million yuan of financial poverty alleviation funds were arranged at all levels. The implementation of the dynamic adjustment mechanism, the end of the year "Poverty Alleviation Policy" filed the number of poverty-stricken people in the card is 29,531 people. There were 111 new industrial poverty alleviation projects, with a total of 800 projects, and the accumulated asset income exceeded 60 million yuan. The annual average values of fine particulate matter (PM2.5), sulfur dioxide and nitrogen dioxide of main pollutants were 34, 10, 31 μg/m3 respectively. Compared with the previous year, PM2.5 improved by 8.1%, sulfur dioxide improved by 28.6%, and dioxide dioxide. Nitrogen improved by 6.1%.

Ⅱ. New Kinetic Energy

According to the preliminary accounting, the annual gross domestic product（GDP）332.7 billion yuan grew by 15.6, accounting for 27.7% of GDP. Among them, the added value of the marine primary industry was 11 billion yuan, increased by 5.1%.The value-added 176.6 billion yuan of the second industry increased by 18%. The value-added 145.1 billion yuan of the tertiary industry increased by 13.7%.Therein, the four pillar industries of coastal tourism, marine transportation, marine equipment manufacturing and sea-related products and materials manufacturing realized an added value of 202.5 billion yuan, increased by 14.5%, accounting for 60.9% of the marine economy. The marine emerging industry realized an added value of 36.6 billion yuan, increased by 8.6%, accounting for 11% of the marine economy.

The added value of the "new economy" was 306.58 billion yuan, accounting for 25.5% of the city's GDP. "New industry" is developing steadily, In the whole year, the city's high-tech industry realized an increase of 6.9% in value added., accounting for 4.6% of GDP. Therein the added value of high-tech manufacturing increased by 3.3%, accounting for 2.0% of GDP. Strategic emerging industry added value increased by 6.2%, accounting for 8.1% of GDP. Among them, Strategic emerging industry service industry added value increased by 8.6%, accounting for 2.7% of GDP. "New business, new model" shows vitality, The city's e-commerce transaction platform achieved a transaction volume of 1.0 trillion yuan, and the retail sales of wholesale and retail units above designated size increased by 27.6%. The "new technology" has developed rapidly. The operating income of high-tech service enterprises above designated size in the city has increased to 22.8% of the revenue of service enterprises above designated size. The output of "new products" increased rapidly, with 2,288 sets of industrial robots, an increase of 59.4%; 1,538 urban rail vehicles, an increase of 20.5%; and 92,000 new energy vehicles, an increase of 14.4%. New kinetic energy investment is driving. Investment in equipment manufacturing increased by 10.7%, investment in strategic emerging industries increased by 32.8%, investment in high-tech manufacturing increased by 59.0%, and investment in industrial technology reform increased by 22.6%.

According to preliminary statistics, the city has won 14 national science and technology awards. Among them, 1 natural science award, 1 technical invention award, 1 international science and technology cooperation award, and 11 scientific and technological progress awards. The technical contract transaction volume was 15.58 billion yuan, an increase of 23.0%. The turnover of sea-related technology contracts was 1.82 billion yuan, an increase of 21.2%. There were 26,267 effective invention patents in the whole year, an increase of 20.5%. The number of valid invention patents per 10,000 people is 28.5, and the number of PCT international patent applications is 1088. There are 10 national IPR demonstration enterprises, 60 national IPR superiority enterprises and 14 national technology innovation demonstration enterprises. There are 39 national enterprise technology centers, and the total number of high-tech enterprises has reached 3,112. Jiehua Bio became the first unicorn company in the province. The research and development forces accelerated, and the number of national key laboratories reached 9, the number of enterprise technology centers above the provincial level reached 193,and the number of engineering (technical) research centers reached 292.

Ⅲ. Agriculture

The annual agricultural added value was 41.06 billion yuan, an increase of 3.6%.Therein, the added value for the planting industry amounted to 19.40 billion yuan, increased by 3.0%. The added value for forestry amounted to 230 million yuan, increased by

11.9%. The added value for the husbandry amounted to 7.61 billion yuan, increased by 7.9%. The added value for the fishing industry amounted to 11.45 billion, declined by 2.2 %.

The grain sowing area stands at 481,000 acres throughout the year. The vegetables sowing area stands at 113,000 acres. And peanuts sowing area stands at 80,000 acres. The total output of grain stands at 3,101,000 tons, increased by 4.4%. The total output of vegetables and edible fungi stands at 6,444,000 tons, increased by 2.6%. The total output of peanuts stands at 385,000 tons, increased by 0.3%. The total output of fruits (including the fruits-purpose melons) stands at 1,090,000 tons, declined by 8.0%. The modern agricultural parks amount to 987, increased by 51. Those certified with 'three products and one mark(pollution-free agricultural products, green food, organic agricultural products and agricultural products geographical indication) amounts to 1056, including 51 national geographic mark protected products.

Throughout the year, 135,000 mu of new trees are planted. 17,000 mu forests are refreshed.

The total meat output is 522,000 tons in the whole year, increased by 3.0%. The output of poultry eggs weighs 180,000 tons, increased by 13.8%. The output of diary stands at 298,000 tons, increased by 10.3%..

Annual aquatic product yield (excluding far ocean fishing) stands at 1.035 million tons, decreased by 1.6%. The ocean and fresh water cultivation area stands at 35,000 acres, decreased by 0.9%.The ocean fishing volume stands at 145,000 tons, Flat with the previous year.

The total power of agricultural machinery is 7.378 million kW, increasing by 300,000 kW. The mechanization level of crop production reached 87.8%.The effective irrigation area of the farmland stands at 331,000 hectares, of which the water-saving irrigation area stands at 188,000 hectares.

Table 3 : Production and Growth of Major Plantation Products in 2018

Indicators	Unit	Yield	Growth（%）
Grain	10 000 tons	310.1	4.4
Summer grain	10 000 tons	137.7	8.9
Autumn grain	10 000 tons	172.4	1.1
Oilseed	10 000 tons	38.5	0.3
Vegetable and edible fungi	10 000 tons	644.4	2.6
Fruit	10 000 tons	109	−8.0
Garden fruit	10 000 tons	71	−5.1

Ⅳ. Industry and Construction

The city's total industrial added value was 413.71 billion yuan, an increase of 6.9%.

Among them, the added value of industrial enterprises above designated size increased by 6.8%. The added value of light industry increased by 8.5%, and the added value of heavy industry increased by 6%.According to the economic types, state−controlled enterprises grew by 4.8 %. The collective enterprises grew by 2.8%. Shareholding enterprises grew by7.0%. The foreign and Hong Kong , Macao and Taiwan business investment enterprises grew by 6.3%. In terms of categories, the added value of the mining industry fell by 14.5%, the added value of the manufacturing industry increased by 6.6%, and the added value of the electricity, heat, gas and water production and supply industries increased by 11.8%. The production and sales rate of industrial enterprises above designated size reached 99.9%. The export delivery value of industrial enterprises above designated size increased by 8.0%, an increase of 3.2 percentage points over the previous year.

Table 4 ： The Output and Growth Rate of the Main Products of Industries above Designated Size in 2018

Description	Unit	Output	Growth（%）
Color TV	10 000 sets	1695.2	−0.5
Of which: Smart TV	10 000 sets	1695.2	−0.5
Household refrigerators	10 000 sets	886.6	3.3
Household washing machin	10 000 sets	601.7	−0.3
Room air conditioners	10 000 sets	1058.1	5.3
Smart phone Beer	10 000 sets	2010.2	−28.9
Tire casing Sheet glass Raw steel	10 000 kiloliters	6388.8	36.6
Automobile	10 000 pieces	561.5	0.7
Motor train units	10 000 tons	309.7	10.2
urban rail vehicle	10 000 vehicle	9.2	14.4
Metal shipping containers	10 000 m^3	1385.5	18.6

The above-scale industrial profits rises by 6%, therein, the state-owned holding enterprises fell by 6.9%, the collective enterprises increased by 33.3%, the shareholding enterprises increased by 4.3%, the foreign merchants and Hong Kong, Macao and Taiwan merchants decreased by 10.4%.

Table 5：Above-scale industrial profit growth in 2018

Indicators	Growth(%)
Industrial profits above the scale	6
Of which ：State-owned holding	-6.9
Collective businesses	33.3
Shareholder businesses	4.3
Businesses invested by foreigner or by compatriots from Hong Kong ， Macao and Taiwan	10.4

In the whole year, there were 711 contracted construction enterprises in the general contracting industry, achieving an added value of 72.34 billion yuan, an increase of 10.5%; the total output value of the construction industry was 230.93 billion yuan, an increase of 22.9%; the contract value was 477.79 billion yuan, an increase of 18.0%; The total profit and tax was 10.56 billion yuan, an increase of 8.9%.

V .service enterprises

The added value of the city's service enterprises accounted for 56.4% of the city's GDP, an increase of 0.9 percentage points over the previous year; the contribution rate to economic growth was 55.6%.

Table6: Added modern service enterprises rate of the whole city in 2018

Industry Classification	Value Added（100 million yuan）	Growth（%）	proportion（%）
Farming，Forestry，Animal Husbandry and Fishery Industry Construction	23.65	6.6	0.2
Metal products, machinery and equipment repair industry	9.89	6.9	0.1
Wholesale and Retail Sales	1440.66	7.3	12
Transport，Storage and Post	830.58	2.7	6.9
Accommodation and catering	268.18	5.7	2.2
Finance	800.4	5.2	6.7
Real Estate	672.46	1.2	5.6
Other Services	2718.2	12.2	22.7
#For-profit service enterprises (mainly including telecommunications, letter of residence and rent)	1416.11	19.6	11.8
#Non-profit service enterprises	1302.09	4.8	10.9

The service enterprises above designated size in the city has grown rapidly, achieving an operating income of 1,957.7 billion yuan, an increase of 12.0%. In the producer service industry, the business service enterprises revenue increased by 29.5%, and the technology service industry revenue increased by 14.2%. In the life service enterprises, the revenue from film and television program production increased by 97.7%, the revenue from film and radio and television program distribution increased by 12.7 times, the revenue of art performance venues increased by 15.8%, and the income of sports organizations increased by 32.0%. The city's modern service enterprises realized an added value of 377.34 billion yuan, an increase of 9.2%, accounting for 31.4% of the city's GDP.

Table 7: Operating income of service industry above designated size in 2018

Catgory	business revenue	Growth（%）
Catgory	1957.7	12.0
Service enterprises above designated size Transport，storage and postal service	1006.8	9.0
Information transmission，software and information technology service	306.8	12.2
Real estate（Excluding real estate development and management）	31.3	10.8
Leasing and business service	306.3	27.4
Scientific research and technical service	142.9	6.8
Management of water conservancy，environment and public facilities	27.4	-29.3
Households ‘service，repair and other services	22.5	-4.2
Education	20.9	14.2
Health and social work	18.6	13.1
Culture，sports and entertainment	74.2	27.1

The transportation, warehousing and postal industries are developing steadily. The port throughput for the year was 540 million tons, an increase of 6.1%; the foreign trade throughput was 390 million tons, an increase of 6.2%; the container throughput was 19.32 million TEUs, an increase of 5.5%. At the end of the year, there are 192 routes，increased by 3.2%。Therein，At the end of the year, there are 160 domestic airlines, 29 international airlines and 3 airlines from Hong Kong, Macao and Taiwan, with an annual total aviation passenger throughput of 2454.1 million and an increase of 5.7%. The throughput of air cargo is 220000 tons, an decrease of 3.2%. In the whole year, the number of subway trains was 330,000, with a total operating mileage of 9.15 million kilometers and a total passenger volume of 152.94 million. The total 65.09 billion yuan of the total business completed in the whole year increased by 105.2%. Among them, the total postal service 8.29 billion yuan increased by 14.4%. The total 56.8 billion yuan of telecommunication services increased by 132.1%.Express business volume amounted to 380 million, increased by 23%.Fixed broadband Internet users amounted to 3.98 million households, with an increase of 7.9%. The city's mobile phone users amounted to 12.59 million households, of which 3G and 4G user 10.64 million households, increased by8.7%.

Table 8 : Volume and Growth Rate Completed by transportation in 2018

Transportation	Unit	Traffic volume	Growth (%)
Passenger volume	100 billion person-km	182.49	6.4
Railways	100 billion person-km	105.36	8.7
Highways	100 billion person-km	76.97	3.4
Waterways	100 billion person-km	0.16	-27.2
Cargo volumes	100 billion ton-km	1647.12	9.3
Railways	100 billion ton-km	190.90	4.3
Highways	100 billion ton-km	532.20	3.2
Waterways	100 billion ton-km	924.02	14.4

The financial industry is developing healthily. The foreign currency deposit balance of financial institution at year end stands at 1,612.13 billion yuan, increased by 99.23 billion yuan than the beginning of the year. The RMB deposit balance stands at 1,553.22 billion yuan, increased by114.44 billion yuan than the beginning of the year. Therein, the residents savings amounts to 591.37 billion yuan, increased by 51.86billion yuan than the beginning of the year. The foreign currency loan balance stands at 1.6098 trillion yuan, increased by 169.29 billion yuan than the beginning of the year. The RMB loan balance stands at 1.51942 trillion yuan, increased by 1.9238 billion yuan than the beginning of the year. The insurance industry realized the30.66 billion yuan of premium income, increased by 7.2%.Among them, the insurance company realized the 13.28 billion yuan of premium income, increased by 20%. Life insurance company realized the30.66 billion yuan of premium income, with an increase of 7.2%. The securities company' s securities trading volume stands at 2.72681 trillion yuan, decrease by 22.7%. There are 236 private funds collection institutions at the end of the year. The scale of the management fund stands at 60.04 billion yuan, increased by 30.9%.

The tourism industry is developing rapidly. The total tourism consumption stands at186.71 billion yuan, increased by 13.8%. the total tourism consumption reached 186.71 billion yuan, an increase of 13.8%. Among them, 153.6 million inbound visitors are

received, with an increase of 6.4%.; the tourism consumption reached 1.16 billion US dollars, an increase of 14%. It received 98.489 million domestic tourists, an increase of 13.7%; the tourism consumption reached 165.1 billion yuan, an increase of 13.8%. At the end of the year, there are 123 scenic spots at level A, including 1 5A-level tourist scenic spots, 4 4A-level tourist scenic spots, and 3A-level tourist scenic spots. There are 99 star-rated hotels, of which 10 are 5-star hotels, 25 are 4-star hotels, and 60 are 3-star hotels. It has 544 travel agencies, including 59 outbound travel business travel agencies and 544 travel and domestic travel business travel agencies.

Ⅵ .Fixed Investment

The city's fixed asset investment increased by 7.9%. Among them, the investment in the primary industry increased by 14.3%, the investment in the secondary industry increased by 9.1%, and the investment in the tertiary industry increased by 7.5%. More than 702 new projects (including real estate) of more than 100 million yuan were added, an increase of 1 from the previous year. The investment was 173.75 billion yuan, down 12.4%, accounting for 33.2% of the city's fixed asset investment.

An investment of 148.52 billion yuan has been accomplished for the real estate development, increased by 11.6%. Therein, the residential investment stands at 103.48 billion yuan, increased by 11.8%. The sales area of the Commodity House is 18.08 million square meters, decreased by 4.9%. Therein, residential sales area stands at 15.783 million square meters, decreased by 3.4%.

Table 9 : Real Estate Development Investment, Commodity Housing Sales Area and Speed Increase in 2018

Category	Real Estate Development Investment (100 million yuan)	Growth (%)	Sales Area of Commodity House (10 000 m^2)	Growth (%)
Total	1485.2	11.6	1808	-4.9
Residential	1034.8	11.8	1578.3	-3.4
Office building	98.5	-12.5	76.7	-21.6
Commercial operation house	153.7	-17.3	119.9	-7.9
Other	198.2	85.7	33.1	-14.6

Throughout the year, the guaranteed housing basically established stands at 15540 sets, including 11212 sets of public rental housing, 2103 sets of affordable housing and 2225 sets of commodity housing. By the end of the year, 7326 families are enjoying the

rental subsidy, and the rental subsidy 43.67 million yuan is distributed throughout the year.

Ⅶ.Domestic Trade

484.25 billion yuan of retail sales of consumer goods is achieved in the whole year, with an increase of 10.0%. According to the statistics, the retail sales amount of consumer goods in town stands at 4026.9 billion yuan, with an increase of 9.7%. Retail sales of consumer goods in rural areas stands at 81.56 billion yuan, with an increase of 11.4%.According to the industry, retail sales of wholesale and retail sales amounted to 422.1 billion, with an increase of 9.1%.The retail sales of accommodation and catering industry realized 62.15 billion yuan, increased by 16.6%.

The retail sales of consumer goods above designated size reached 164.42 billion yuan, increased by 9.1%. The retail sales of tobacco and alcohol increased by 27.0%, and the retail sales of clothing, shoes, hats and needles increased by 8.6%. The retail sales of cosmetics increased by 4.8%, the retail sales of gold and silver jewellery increased by 11.5%, the retail sales of daily necessities increased by 19.1%, the retail sales of household appliances and audio equipment increased by 18.5%, and the retail sales of Chinese and Western medicines increased by 25.1%. Retail sales increased by 10.1%, retail sales of construction and decoration materials increased by 12.3%, retail sales of petroleum and products increased by 9.8%, and retail sales of automobiles decreased by 0.1% compared with the previous year.

Ⅷ.Foreign Trabe

The total imports and exports sales throughout the city stands at 532.13yuan, increased by 5.7.Therein, the exports valued 317.22 billion yuan, increased by 4.7%. The imports valued 214.90 billion yuan, increased by 7.3%.The total imports and exports to the countries along the 'Belt and Road' stands at 259.04 billion yuan, increased by 4.2%. Therein, the exports stands at 163.06 billion, increased by 1.9%. The imports stands at 95.98 billion, increased by 8.4%.

The total of import and export of foreign trade in Qingdao port stands at 1.21968 trillion yuan, increased by 9.7%. Among them,the exports stands at 673.85 billion yuan, increased by 6.5%. The imports stands at545.83 billion yuan, increased by 13.9%.

Table10 ： Import & Export of Main Commodities to/from Qingdao in 2018

Category	Import & Export		Export		Import	
	Amount (100 million yuan)	GrowthRate (%)	Amount (100 million yuan)	GrowthRate (%)	Amount (100 million yuan)	GrowthRate (%)
Textiles and garments	511.2	6.5	482.0	7.7	29.2	-9.8
Agricultural products	797.7	11.8	387.3	8.4	410.4	15.2
Elextrical and mechanical	1786.8	1.6	1352.2	0.5	434.6	5.2
High-tech products	525.2	5.1	316.1	5.1	209.1	5.1

Table 11 : Import and Export Volume and Growth Ratefrom /to Major Countries and Regions in 2018

Country (Region)	ExportAmount (10 000dollars)	Growth (%)	Import Amount (10 000dollars)	Growth (%)
Asia	2083063	9.8	1280932	5.3
Hong Kong	191991	9.2	11487	19.6
Taiwan	41522	8.5	118789	15.9
	562514	6.6	185504	3.8
Japan	444012	7.6	342617	12.8
Korea	463450	24.0	379222	-14.9
ASEAN	83850	39.7	63178	23.0
South Asia	74747	-0.3	60583	-54.8
Middle East	44554	30.4	47902	14.5
	76848	33.0	134631	-12.8
Africa	161294	16.2	38975	57.3
South Africa	100542	27.8	34467	60.0
Europe	188352	-12.3	135723	82.5
EU	192717	14.1	260346	44.9
U.K.	45146	12.9	26943	25.6
	1019206	-3.3	506724	29.5
Germany France	881451	7.2	290470	19.7
France	140738	8.4	60435	52.8
Italy	151262	3.2	85797	26.0
CIS and Eastern Europe	85567	-0.8	36255	9.5
Russia	84584	10.4	21093	-0.3
	119678	-44.5	183367	52.4
South America	89922	-52.9	174582	51.2
Brazil	317302	7.9	531235	-2.4
North America	55242	30.3	374326	53.2
U.S.A.	1045878	13.7	316690	3.0
	930533	12.3	256098	-0.3
Canada	109438	20.7	53394	21.7
Oceania	135684	10.8	353478	13.1
Australia	113291	11.8	298080	9.7

In 2018, the city approved 956 new foreign investment projects, an increase of 47.1%; contractual foreign investment was US$9.82 billion, an increase of 7.8%. The actual use of foreign capital in the city was 8.69 billion US dollars, an increase of 10%; according to the statistics of the Ministry of Commerce FDI (foreign direct investment), the actual use of foreign capital was 5.80 billion US dollars, an increase of 4.1%.

Throughout the year, there are 22 investment projects to the countries and regions along the 'Belt and Road', with an agreed investment amount of US dollars 2.0 billion, increased by8.9%; The actual investment amounted to 680 million US dollars, an increase of 86.9%.

Of the 107 newly contracted projects in the whole city, the new contract amount is 49.3 billion US dollars, an increase of 11.9%,In the city's foreign labor service cooperation service, 18,294 people are sent,

Ⅸ .Society enterprise

At the end of the year, there are 25 colleges and universities (including private universities) within the city, including 23universities. 16,000 post-graduate student enrollment in the entire year, 45,000 on-duty postgraduates and 110,000 graduates (excluding the p ostgraduates stationed in Qingdao Research Institute)102,000 students enrolled by the General Higher Education Institutes,353,000 students in school and 94,000 graduated.26,000 persons for vocational education enrollment , 84,000 for in-school students, and 28,000 graduated. 38,000 persons for senior high school enrollment , 117,000 for in-school students, and 38,000 graduated.86,000 persons

for junior high school enrollment , 268,000 for in-school students, and 7,2000 graduated.104,000 persons for general primary school enrollment , 570,000 for in-school students, and 86,000 graduated.20 persons for special education enrollment , 200 for in-school students, and 20 graduated. There are 264,000 kids in the kindergartens.

At the end of the year, there are 3745 healthcare institutes (excluding the clinics in villages), 422 hospitals and health centers, 26 disease prevention and control centers, 12 maternal and child health institutions, 2917 clinics and Medical Rooms. At the end of theyear, there are 84,000 various health technicians, among them, the doctors amounted to 35,000 persons. There are 58,000 of medical beds in the city, of which there are 56,000 beds in hospitals and health centers.

The city athletes won 353 gold medals, 247 silver medals and 236 bronze medals in the competitions. There are 2 key sports universities with 1370 students. The amateur sports schools amounted to 10, with 1846 students.

Ⅹ .Urban construction

At the end of the year, the urbanization rate of the city's permanent residents reached 73.67%, increasing by 1.1 percentage points over the previous year. The daily water supply for the city is 129,700 tons, increased by 2.9%.The actual water consumption of the city is 410 million tons in the whole year, in which the production water and living water accounts to 160 million tons and 250 million tons, respectively.

The total household number of liquefied gas and natural gas in cities reaches1.898 million households, and the total amount of liquefied gas is 27000 tons in the whole year, and the total amount of natural gas is 107 million cubic meters. And the gasification rate of the city reaches 100 percent.

In the year, the heating area is 10.604 million square meters, and the heating area at the end of the year reaches 200 million square meters.

At the end of the year, there are 623 bus routes and tram lines. There are 8475 buses running in public. There are 10867 Taxis. At the end of the year, there are 4 metro lines, with 152.9412 million of annual passenger capacity and 419000 of daily average passenger capacity.

There are 788 public toilets in the city, 774 above Category III, 202 newly-added toilets and 54 alterations.

Ⅺ .Energy， Environment and Work Safety

Preliminary statistics show that the total industrial comprehensive energy consumption of the whole city in the whole year is 14.89 million tons of standard coal, increasing by 4.1 percent. Of these, coal consumption is 13.60 million tons, down by 1.7%. The consumption of crude oil is 15.42 million tons, increased by 1.2%. The natural gas consumption is 1000 million cubic meters, with an increase of 89.3%.The total power consumption of the whole society is 43.2 billion KWh , with an increase of 7.7%. Among them, the industrial electrical consumption amounted to 23.8 billion kilowatt-hour, increased by 5.4%. The living consumption of urban and rural residents amounts to 7.7 billion kilowatt-hours, with an increase of 2.2%.

In the year, the city's energy production stands at 28.34 million tons of standard coal, increased by 2.1%. Among them, the processing capacity of crude oil is 15.41 million tons, with an increase of 1.2%. The production of gasoline is 4.74 million tons, with an increase of 3.9%. The production of diesel oil is 3.83 million tons and the drop of diesel oil is 6.2%. The power generating capacity is 19.2 billion kWh, increased by 4.8%.The output of new energy is 1.7 billion, increased by 13.5%. The wind power generation increased by 12.6, and the biomass power generation increased by 25.4%.The power generation of new energy accounts for 9.1% of the total power generation of the city, and increased by 0.7 percentage points.

The annual average temperature of the whole city is 13.3 ℃ , and the average annual precipitation is 722.7 millimeters. The city's air quality is 307 days, accounting for 85% of the year. In the whole city, the water with quality up to or better than that of Category II sea water accounts for 90.6%, which was the same as the previous year. The average value of environmental noise in urban area is 56.9 decibels, and the average noise of urban traffic trunk line is 68.0 decibels.

There are 63 production safety accidents in seven industries (domains) in the city, including 69 deaths, decreased by 42.2% and 38.4% respectively. The death rate of 100 million GDP production safety accident rate is 0.006. The death rate of the production

safety accident of the employment personnel of the trade and commerce enterprise is 0.72. The death toll of the road traffic accidents is 11300.

Ⅻ .Living Conditions and Social Security

In the whole year, the per capita disposable income of the city residents stands at 42019 yuan, increased by 8.4%. Categorizing according to the permanent residential place, the per capita disposable income of urban residents stands at 50817 yuan, increased

by 7.7%. The per capita disposable income of rural residents stands at20820 yuan, increased by 7.5%. The income ratio of urban and rural residents in the city is 2.44:1.

The per capita consumption expenditure, which stands at 27,316 yuan, in the whole city

increased by 8.3 percent, and the Engel coefficient is 28.5 percent. Categorizing according to the permanent residential place, the per capita consumption expenditure of urban residents stands at 32,890 yuan, increased by 7.6%. the per capita consumption expenditure of rural residents stands at 13,885 yuan, increased by 7.4%.

Table 12 ： Per Capita Disposable Income of Urban and Rural Residents in 2018

	Residents		Urban Residents		Rural Residents	
Description of inducators	Absolute Value (yuan)	Growth Rate (%)	Absolute Value (yuan)	Growth Rate (%)	Absolute Value (yuan)	Growth Rate (%)
Disposable income	42019	8.4	50817	7.7	20820	7.5
Salary income	25303	7.6	30955	6.8	11684	7.4
Operating net income	8027	7.3	7836	7.4	8487	7.3
Propetry net income	3475	12.8	4789	11.4	310	10.8
Transfer net income	5214	11.3	7237	9.9	339	17.1

Table 13 : Per Capita Disposable Expense of Urban and Rural Residents in 2018

Description of inducators	Residents		Urban Residents		Rural Residents	
	Absolute Value (yuan)	Growth Rate (%)	Absolute Value (yuan)	Growth Rate (%)	Absolute Value (yuan)	Growth Rate (%)
Consumption expense	27316	8.3	32890	7.6	13885	7.4
tobacco and liquor	7787	4.0	9318	2.9	4098	5.9
Chothing	2538	2.9	3168	1.7	1020	5.1
Housing Living	6365	16.7	7751	17.3	3024	8.0
product and services	1837	5.1	2207	4.0	945	6.7
Transportation andcommunication	4084	8.8	4765	8.3	2445	8.1
Education，culture and entertainment	2571	9.5	3122	8.6	1245	10.3
Medical and health care	1480	9.6	1764	8.6	794	10.6
Other product and services	654	4.6	795	3.4	314	6.9

Table 14：Amount of Major Consumer Durable Owned by every 100 Households in2018

Description of inducators	Unit	Quantity		
		Residents	Urban Residents	Rural Residents
Household automobile	vehicle	56	62	43
Motorcycles	vehicle	28	15	61
Refrigerators	set	108	108	109
Washing machines	set	99	100	97
Water heaters	set	97	101	88
Air-conditioners	set	112	120	93
Color TV	set	107	107	105
Computers	set	70	81	45
Mobile telephones	set	230	228	232

According to the sample survey, the per capita housing construction area of urban residents at the end of the year is 32.9 square meters, and the per capita housing construction area of rural residents is 35.9 square meters.

In the whole year, the newly-increased urban residents employment number stands at 776,000 person, increased by 6.3%.Therein, the employment of workers in the city increased by 4.8%, and the employment of foreign workers increased by 8.1%.The service sector absorbed an employment of 507,000 persons, with an increase of 16.9%.The private economy absorbed an employment of 596,000 persons, with an increase of 11.3%.

At the end of the year, the minimum life guarantee number of urban residents in the city reached 20, 457. The minimum living security fund for urban residents stands at 199.23 million yuan. There are a total of 4, 843 people in the rural five-guarantee support,and the rural five-guarantee support expenditure amounts to 76.245 million yuan.

At the end of the year, the number of insured persons in the basic old-age insurance for workers in the whole city is 2.7351 million.The number of unemployed insurance is 2.2369 million persons. The number of unemployed insurance contributions received in thewhole year is 75,000 persons. The urban registered unemployment rate in the whole city is 2.94%, which decreased by 0.18 percentage points.

Note：

1. Figures reported herein are preliminary statistics.

2. The absolute figures of gross domestic product (GDP) and added value of each industry are calculated according to the current price, and the growth rate is calculated according to the current price. According to "three industry division regulations" (Guo Tong Zi[2012] No. 108), three industries, such as agro-forestry, fishing services, industry, auxiliary activities in industry, metalwork, machinery and equipment repair, are classified into the tertiary industry.

3. The resident population includes: (1) the person who lives in the household and the household registration in the township of this Town (including the person whose household registration is in the household and who has been out for less than half a year). (2) the person who has lived in the household for more than half a year, the household

registration is in the street outside the township. (3) The household registration is in the street of the foreign township, and the person who has left the household registration for more than half a year. (4) who lives in the household and whose household registration is pending.

4. PCT is an English abbreviation for "Patent Cooperation Treaty". The PCT international patent refers to Chinese patent application person can submit international patent application to the China Patent Office or to the World Intellectual Property Organization according to the PCT regulations, and is an important index to reflect the development level of export-oriented economic development in the region and the competitiveness of international technology in the region.

5. The strategic emerging industry, according to the national planning, mainly includes seven industrial fields such as energy conservation, environmental protection, new generation of information technology, biology, high-end equipment manufacturing, new energy, new materials and new energy Automobile.

6. The industrial enterprises above the scale are the annual main business income 20 million yuan and the above enterprises. Trade enterprises above designated size are units with a main business income of 20 million yuan or more in the wholesale industry, 5 million yuan or more in the retail industry, and 2 million yuan and above in the accommodation and catering industry. The starting point standard of the fixed asset investment project statistics is the planned total investment 5 million yuan. For service enterprises above designated size, the first refers to service companies with an annual business income of 10 million yuan or more or 50 or more employees at the end of the year. including transport, storage and postal services, information transmission, software and information technology services, rental and business services, scientific research and technology services, water resources, environmental and public facilities management, education, health and social work. As well as property management, real estate brokerage services, self-owned real estate business activities and other real estate industry. Second, it refers to a service legal entity with an annual operating income of 5 million yuan or more or 50 or more employees at the end of the year. Including: resident services, repairs and other services, cultures, sports and recreation.

7. The statistics range of the number of production safety accidents and the number of deaths include the accidents that occur in the seven industries (areas) of production and business road traffic, water transportation, railway transportation, civil aviation flight, agricultural machinery, fishery ship and maritime trade and commerce, and

no longer includes forest fire, Real-time and non-production operational road traffic.

8. The area of built-up area, water supply, water use, gas supply, heat supply, roads, toilets, sewers and other relevant data excludes Jimo District for now.

9. Source: The financial data in this bulletin comes from the Municipal Finance Bureau. the talents, registered unemployment rate, and social security data come from the Municipal Human Resources and Social Security Bureau. the bank data comes from the Qingdao Central Branch of the People's Bank of China. the securities data comes from

the Qingdao Securities Regulatory Bureau. the insurance data comes from Qingdao Insurance Regulatory Bureau. aquatic product data from the Municipal Ocean and Fisheries Bureau. forestry data from the Municipal Forestry Bureau. “ three products and one standard” , agricultural machinery and other data from the Municipal Agricultural

Commission. irrigated area data from the Municipal Water Resources Bureau. transportation data from the city traffic Transportation Committee. postal data from the Municipal Postal Administration. fixed telephone, broadband, mobile phone and other data from the Municipal Communications Administration. built-up area, water supply, gas supply, heating, roads, sewers and other data from the Municipal Urban and Rural Construction Committee. Export, foreign investment, foreign contracted projects, foreign labor cooperation, cross-border e-commerce and other data from the Municipal Bureau of Commerce. tourism data from the Municipal Tourism Development Committee. education data from the Municipal Education Bureau. technology, patent data from the Municipal Science and Technology Bureau. sports data from Municipal Sports Bureau. health data from the Municipal Health and Family Planning Commission. environmental data From the Municipal Environmental Protection Bureau. meteorological data from the Municipal Meteorological Bureau. safety production data from the Municipal Safety Supervision Bureau. poverty alleviation data from the Municipal Poverty Alleviation Cooperation Office. car ownership from the Municipal Public Security Bureau. household income and expenditure, prices, urban and rural residential housing area and other data from Qingdao Bureau of Investigation, National Bureau of Statistics. other data from the Municipal Bureau of Statistics.

综　合 1

GENERAL SURVEY

简 要 说 明

一、本篇资料的主要内容

本篇资料是对我市行政区划、气象情况、分行业法人单位数、各部门机构数和国民经济、社会发展的综合反映，主要包括行政区划、气象情况、分行业法人单位数、各部门机构数、国民经济主要比例关系、国民经济和社会发展主要指标占全国全省比重、国民经济和社会发展主要指标及其增长速度等资料。

二、本篇资料的来源

1、"行政区划"主要包括2018年底各区（市）街道办事处（乡镇）、社区居委会（村民委员会）区划资料，数据来源于市民政局。

2、"气象情况"包括分区市、市区分月气象资料，数据来源于市气象局。

3、"全市生产总值"来源于国民经济核算统计报表，由市统计局国民经济核算处整理提供。

4、国民经济和社会发展综合部分来源于本年鉴各篇章中的资料，由市统计局国民经济综合统计处加工整理。

Brief Introduction

I. Main Content

Data in this chapter cover the main indicators on divisions of administrative areas, meteorology, corporate units, number of grass-roots units and national economy and social development, including divisions of administrative areas, meteorology, number of corporate units, number of grass-roots units, average daily social and economic activities, ratio, and percentage of main indicators of Qingdao to the whole nation and the whole province and growth rate of main indicators.

II. Source of Data

(1) Data on divisions of administrative areas are mainly including the information on sub-district offices (villages and towns) and community neighborhood committees (villagers residents' committees) at the end of 2018. The data are provided by Qingdao Municipal Bureau of Civil Affairs.

(2) Data on meteorology are provided by Qingdao Municipal Meteorological Bureau.

(3) Data on gross national product are prepared according to the data of national accounts and compiled by the Division of National Accounts of Qingdao Municipal Bureau of Statistics.

(4) Data on general survey of economy and society are based on those of different chapters and compiled by the Division of Comprehensive Statistics of Qingdao Municipal Bureau of Statistics.

1-1 行政区划（2018 年底）
ADMINISTRATIVE DIVISION（END OF 2018）

单位：个（unit）

市区名称	Region	街道办事处 Subdistrict Offices	社区居委会 Community Residents' Committees	镇 Town	村民委员会 Villagers' Committees
**全　市**	**Whole Municipality**	**104**	**1206**	**41**	**5445**
市南区	Shinan District	14	50		
市北区	Shibei District	30	137		
李沧区	Licang District	11	116		
崂山区	Laoshan District	5	162		
黄岛区	Huangdao District	12	267	10	961
城阳区	Chengyang District	8	247		1
即墨区	Jimo District	8	65	7	1028
胶州市	Jiaozhou	8	78	4	811
平度市	Pingdu	5	51	12	1783
莱西市	Laixi	3	33	8	861

1-2　气象情况（2018 年）
METEOROLOGY（2018）

市区名称	Region	平均气温（摄氏度）Average Temperature（℃）	极端气温 Extreme Temperature（centigrade）		降水量（毫米/年）Precipitation（millimeter/year）	日照时数（小时/年）Sunshine Hours（hour/year）	平均气压（百帕）Average Atmospheric Pressure（100 pa）
			最高（摄氏度）Maximum（℃）	最低（摄氏度）Minimum（℃）			
市　区	Urban Area	13.5	35.3	-10.9	686.2	2083	1008.1
崂山区	Laoshan District	13.3	35.1	-11.3	806.8	2370.5	1010.8
黄岛区	Huangdao District	13.6	35.3	-11.8	705.9	2368.5	1016.2
即墨区	Jimo District	13.6	36.1	-13.7	862.3	2403.4	1011.6
胶州市	Jiaozhou	13.5	35	-11.9	741.9	2355.1	1007.2
平度市	Pingdu	13.3	36	-15.3	642.1	2296.8	1009.9
莱西市	Laixi	12.5	36.8	-14.6	613.7	2512.4	1008.2

注：以上为 7 个国家基准站或基本站
Note：These are 7 national reference or base stations.

1-3　市区分月气象情况（2018 年）
MONTHLY METEOROLOGY OF URBAN AREA（2018）

月　份	Month	平均气温（摄氏度）Average Temperature（℃）	降水量（毫米）Precipitation（millimeter）	日照时数（小时）Sunshine Hours（hour）
一月	Jan.	-0.6	5	134.4
二月	Feb.	1.4	0.8	152.6
三月	Mar.	6.7	45.1	177.3
四月	Apr.	11.9	36.9	197.7
五月	May	16.9	95.3	199.1
六月	June	21	116.7	172.4
七月	July	25.3	150.4	134.9
八月	Aug.	27.7	85.2	222.6
九月	Sept.	22.5	90.4	194.5
十月	Oct.	16.1	14.9	240.7
十一月	Nov	10.8	17.8	145.5
十二月	Dec.	2.2	27.7	111.3
全　年	Annual Total	13.5	686.2	2083

1-4 各部门机构数 (2018 年底)
GRASS-ROOTS UNITS IN VARIOUS SECTORS（END OF 2018）

项目	Item	单位	Unit	机构数 Grass-roots Units
**农村基层单位**	**Rural Grass-roots Units**			
镇政府	Town Governments	个	unit	43
乡村户数	Number of Rural Households	万户	10 000 households	131.2
**工业企业单位**	**Industrial Enterprises**	个	**unit**	**34240**
规模以上工业	Above Designated Size	个	unit	3508
# 国有企业	State-owned Enterprises	个	unit	145
集体企业	Collective-owned Enterprises	个	unit	4
外商及港澳台商投资企业	Enterprises with Funds from Hong Kong, Macao，Taiwan and Foreign Conutries	个	unit	938
规模以下工业	Above Designated Size	个	unit	30732
**交通运输业**	**Transportation**			
铁路	Railway	个	unit	
公路	Highway	个	unit	214
水运	Waterway	个	unit	14
**邮电业**	**Rostal and Telecommunication Services**			
邮政局 (所)	Post Offices	处	unit	265
电信营业网点 (自有)	Telecom business outlets	个	unit	458
**建筑业企业**	**Construction Enterprises**	个	**unit**	711
国有企业	State-owned Enterprises	个	unit	19
集体企业	Collective-owned Enterprises	个	unit	8
其他	Others	个	unit	684
**批发和零售业、住宿和餐饮业**	Wholesale & Retail Trades，Hotels and Catering Services			
限额以上批发业	Wholesale Trade above Designated Size	个	unit	1338
限额以上零售业	Retail Trade above Designated Size	个	unit	725
限额以上住宿业	Hotel above Designated Size	个	unit	181
限额以上餐饮业	Catering Service above Designated Size	个	unit	187

1-4 续表
Continued

项目	Item	单位	Unit	机构数 Grass-roots Units
**教育事业**	Education			
普通高等学校	Regular Institutions of Higher Education	所	unit	25
中等专业学校	Specialized Secondary Schools	所	unit	6
普通中学	Regular Secondary Schools	所	unit	319
普通小学	Regular Primary Schools	所	unit	713
幼儿园	Kindergartens	所	unit	2241
**科研机构**	Science and Technology Institutions			
中央属	Central Level	个	unit	22
地方属	Local	个	unit	36
**文化及相关产业**	Culture and Related Industry			
公共图书馆	Public Libraries	个	unit	12
影剧院	Cinemas	个	unit	72
**卫生事业**	Public Health			
# 医院	Hospitals	个	unit	421
**民政行政单位**	Civil Affairs Departments			
# 社会福利事业	Institutions of Social Welfare	个	unit	228

1-5 按行业分法人单位数
NUMBER OF CORPORATE UNITS BY SECTOR

单位：个（unit）

行　业	Sector	2018
总　计	Total	358783
农、林、牧、渔业	Agriculture Forestry Animal Husbandry and Fishery	842
采矿业	Mining	158
制造业	Nanufacturing	60631
电力、热力、燃气及水生产和供应业	Electricity, heat, gas, water production and supply industry	539
建筑业	Construction	27156
批发和零售业	Wholesale and Retail Trades	123864
交通运输、仓储和邮政业	Transport Storage and Post	13726
住宿和餐饮业	Hotels and Catering Services	5934
信息传输、软件和信息技术服务业	Information Transmission Computer Services and Software	14934
金融业	Financial Intermediation	654
房地产业	Real Estate	10066
租赁和商务服务业	Leasing and Business Services	42405
科学研究和技术服务业	Scientific Research and Technical Services	21059
水利、环境和公共设施管理业	Management of Water Conservancy Environment and Public Facilities	2065
居民服务、修理和其他服务业	Services to Households and Other Services	7309
教育	Education	6612
卫生和社会工作	Health Social Security and Social Welfare	3157
文化、体育和娱乐业	Culture Sports and Entertainment	7027
公共管理、社会保障和社会组织	Public Management and Social Organizations	10645

注：国家经普办正在审核查询数据，表中为初步数。
Note：The National Economic Census Office is reviewing the inquiry data. The preliminary figures are in the table.

1-6　国民经济主要平均指标
AVERAGE INDICATORS ON NATIONAL ECONOMY

指　标	Indicator	单位	Unit	2000 年	2005 年
人口密度	DEensity of Population	人 / 平方公里	person/sq.km	664	695
每户年平均人口	Average Household Size	人	person	3.14	3.12
城镇非私营单位在岗职工年平均工资	The Average Annual Wage of Staff and Workers in the Post in Non-Private Units in Towns and Cities	元	yuan	10 072	19086
城市居民人均年可支配收入	Annual Per Capita Net Income of Rural Households	元	yuan	8 016	12920
农民人均年纯收入	Annual Per Capita Net Income of Rural Households	元	yuan	3 637	5806
每一播亩平均粮食产量	Output of Grain Per Mu of Sowing Area	千克	kg	411	421
每亩蔬菜平均产量	Average Output of Vegetables Per Mu	千克	kg	2 735	2985
每亩茶叶平均产量	Average Output of tea Per Mu	千克	kg	8.1	16.5
每亩花生平均产量	Average Output of Peanut Per Mu	千克	kg	298	327
每亩棉花平均产量	Average Output of Cotton Per Mu	千克	kg	83	74
每台拖拉机负担耕地面积	Cultivated Area Ploughed by Per Tractor	亩 / 台	mu/unit	56	39
每亩耕地施用化肥量（折纯）	Chemical Fertilizer Consumption Per Mu（convert to pure amount）	千克	kg	45	52
每人平均消费品零售额	Per Capita Retail Sales of Consumer Goods	元	yuan	6077	11822
城市每天平均生活用水量	Daily Residential Consumption of Water	万吨	10 000 tons	33.33	34.35
城市每人平均公园绿地面积	Per Capita Public Green Areas	平方米	sq.m	8.50	11.82
每万人拥有医疗床位	Number of Hospital Beds Per 10000 Population	张	bed	34.5	41.3
每万人拥有医生数	Number of Doctors per 10000 Population	人	person	21.0	20.3
每万人中高等学校学生数	Students Enrollment of Institutions of Higher Education per 10000 Population	人	person	65	324
每万人中中等学校学生数	Students Enrollment of Secondary Schools per 10000 Population	人	person	704	768
每万人中小学学生数	Students Enrollment of primary Schools per 10000 Population	人	person	757	648

注：1. 自 2015 年开始，全市城乡住户调查统一使用一体化改革后的数据，与原数据相比，城乡居民收支指标的调查范围和口径均存较大变化。原“城市居民人均可支配收入”调整为“城镇居民人均可支配收入”；原“农民人均纯收入”调整为“农村居民人均可支配收入”。
2.2008 年 -2017 年农作物数据为根据第三次农业普查结果核定修正后数据。

Note: 1.Since 2015, the urban and rural households investigation unified the data after the integrated reform, compared with the original data, the investigation scope and the caliber of urban and rural residents' income and expenditure indicators have changed greatly.
The original “ per capita disposable income of urban residents” is adjusted to be a “ per capita disposable income of urban residents”. The original “ per capita net income of farmers” is adjusted to “ per capita disposable income of rural residents”.
2.Crop data from 2008 to 2017 are revised based on the results of the third agricultural census.

2008年	2009年	2010年	2011年	2012年	2013年	2014年	2015年	2016年	2017年	2018年
715	676	677	679	682						
3.13	3.12	3.10	3.09	3.09						
29404	32507	37501	43077	49052	55363	62104	69465	76616	83539	
20464	22368	24998	28567	32145	35227	38294	40370	43598	47176	50817
8509	9249	10550	12370	13990	15731	17461	16730	17969	19364	20820
408	411	399	407	411	407	396	400	404	414	430
3551	3624	3689	3867	3887	3957	3963	3889	3760	3931	3796
39	38	44	47	51	49	52	57	74	75	80
316	315	306	316	326	314	305	287	294	319	321
70	71	62	74	81	81	89	86	82	85	110
36	35	32	31			38	38	37	36	36
50	48	47	47			37	36	36	36	35
19640	22699	25694	30043	34248	38606	43064	47423	51765	56452	59214
30.96	33.56	34.31	27.53	33.21	32.50	40.60	42.10	67.60	50.3	50.6
14.53	14.50	14.58	14.58	14.60	14.60	14.60	14.60	18.60	17.4	16.0
42.5	43.0	47.2	52.2	61.4	58.0	60.3	62.0	63.9	69.4	68.2
21.4	21.9	23.2	23.9	28.1	31.2	32.0	33.5	34.9	38.4	42.3
354	361	373	380	386	388	402	411.0	430.0	431.0	486.6
765	740	701	659	635	626	614	596.0	593.0	611.0	612.5
627	610	606	626	630	642	662	685.0	690.0	685.3	696.9

1-7　主要年份社会经济主要指标

MAJOR YEAR'S INDICATORS ON SOCIETY AND ECONOMY

指　标	Indicator	单位	Unit	2000 年	2005 年
**人口**	**Population**				
总人口（年末）	Population at Year-end	万人	10 000 persons	706.65	740.91
男性人口	Male	万人	10 000 persons	357.74	374.03
女性人口	Female	万人	10 000 persons	348.91	366.87
市区人口	Urban Area	万人	10 000 persons	234.60	265.43
**就业**	**Employment**				
社会从业人员	Employment	万人	10 000 persons	397.6	471.0
#单位从业人员	Employed Persons in Units	万人	10 000 persons	118.3	224.3
**经济总量**	**Gross Economic Amount**				
全市生产总值（当年价）	GDP（at current price）	亿元	100 million yuan	1191.25	2687.46
第一产业增加值	Primary Industry	亿元	100 million yuan	140.85	178.33
第二产业增加值	Secondary Industry	亿元	100 million yuan	555.21	1392.02
第三产业增加值	Tertiary Industry	亿元	100 million yuan	495.19	1117.10
人均生产总值	Per Capita GDP	元	yuan	16009	33085
农林牧渔业总产值（当年价）	Gross Output Value of Farming，Forestry，Animal Husbandry and Fishery（at current price）	亿元	100 million yuan	248.33	320.51
工业总产值（当年价）	Gross Industrial Output Value（at current price）	亿元	100 million yuan	1940.83	5001.78

注：1. 单位从业人员 2004 年以前为职工人数(不含私营企业)。
2. 规模以上固定资产投资数据 2003 年以前为城镇以上统计范围。
3. 2002 年以前地方财政一般预算收支数据为地方财政收支口径。
4. 2008 年 -2017 年农林牧渔业总产值为根据第三次农业普查结果核定修正后数据。

Note: 1.Before 2004，the data of employed persons in units refers to the number of staff and workers（excluding private enterprises）.
2.Before 2003，the data of investment in fixed assets above designated size refers to urban investment.
3.Before 2002，the data of general budgetaty revenue and expenditure of local government finance refers to revenue and expenditure of local government finance.
4.Gross Output Value of Farming，Forestry，Animal Husbandry and Fishery from 2008 to 2017 is revised according to the results of the third agricultural census.

2008 年	2009 年	2010 年	2011 年	2012 年	2013 年	2014 年	2015 年	2016 年	2017 年	2018 年
761.56	762.92	763.64	766.36	769.56						
382.46	382.35	381.92	382.69	383.82						
379.10	380.56	381.72	383.67	385.74						
276.25	275.47	275.50	277.09	279.57						
513.8	525.7	540.3	551.2	559.9	571.5	589.0	595.4	601.4	603.9	
254.1	260.5	269.4	275.9	283.2	293.7	303.0	309.2	315.6	321.4	
4401.56	4853.87	5749.02	6725.76	7424.32	8147.32	8850.54	9472.40	10184.7	11024.11	12001.52
223.40	230.25	276.99	306.38	324.41	340.50	349.62	363.98	359.3	368.85	386.91
2234.83	2420.14	2823.43	3236.92	3496.46	3745.12	4014.99	4168.11	4309.65	4546.21	4850.59
1943.33	2203.48	2648.60	3182.46	3603.45	4061.70	4485.93	4940.31	5515.75	6109.05	6764.02
52266	57251	65827	76804	84063	91376	98283	104418	111302	119215	128459
399.5	407.1	480.2	534.6	567.3	610.6	627.9	612.4	669.1	697.9	737.0
8946.73	10255.62	11614.83	13277.96	15306.32	16897.18	17444.21	18019.43	17416.71	13019.73	11389.78

1-7　续表 1
Contiuned

指　标	Indicator	单位	Unit	2000 年	2005 年
**财政**	**Government Finance**				
公共财政预算收入	Public Budget Revenue	亿元	100 million yuan	80.01	176.34
公共财政预算支出	Public Budget Expenditure	亿元	100 million yuan	87.87	203.06
**规模以上固定资产投资额**	**Investment in Fixed Assets above Designated Size**	**亿元**	**100 million yuan**	**242.68**	**1403.30**
**社会消费品零售额**	**Total Retail Sales of Consumer Goods**	**亿元**	**100 million yuan**	**428.29**	**870.11**
**港口吞吐量**	**Volume of Freight Handled in Ports**	**万吨**	**10 000 tons**	**8661**	**18727**
**集装箱吞吐量**	**Volume of Containers Handled**	**万标箱**	**Ten Thousand TEUs**	**212.0**	**630.7**
**民航货物吞吐量**	**Volume of Freight and Mail Handled in Covil Aviation**	**万吨**	**10 000 tons**	**4.84**	**8.91**
**对外贸易**	**Foreign Trade**				
外贸进出口总额	Imports and Exports（excluding central and provincial companies）	亿美元	100 million USD	108.31	304.55
#出口总额	Exports	亿美元	100 million USD	61.14	175.88
**教育、卫生、文化**	**Education Public Health，Culture**				
小学在校学生数	Students Enrollment in Primaty Schools	万人	10 000 persons	53.49	47.98
普通中学在校学生	Students Enorllment in Regular Secorndary Schools	万人	10 000 persons	39.85	39.11

2008 年	2009 年	2010 年	2011 年	2012 年	2013 年	2014 年	2015 年	2016 年	2017 年	2018 年
342.44	376.99	452.61	566.14	670.18	788.93	895.25	1006.26	1100.03	1157.24	1231.9
369.41	433.58	532.39	658.06	765.98	1014.23	1074.71	1222.87	1352.85	1403.03	1561.23
**2019.01**	**2458.89**	**3022.48**	**3502.54**	**4153.91**	**5027.86**	**5766.03**	**6555.70**	**7454.70**	**7777.09**	
**1492.22**	**1730.22**	**1961.13**	**2302.37**	**2635.62**	**2986.81**	**3361.72**	**3713.69**	**4104.93**	**4541.01**	**4842.5**
**30029**	**31668**	**35012**	**37971**	**41465**	**45782**	**47701**	**49749**	**51463**	**51314**	**54250**
**1037.7**	**1027.7**	**1201**	**1302**	**1450**	**1552**	**1658**	**1743**	**1805**	**1831**	**1932**
**13.05**	**13.54**	**16.37**	**16.65**	**17.19**	**18.62**	**20.44**	**20.80**	**23.07**	**23.00**	**22**
521.59	439.86	561.49	712.63	732.08	779.12	798.88	702.22	4350.67（亿元）	5033.50（亿元）	5321.26（亿元）
314.62	269.22	333.51	400.56	408.20	419.86	457.77	453.50	2821.91（亿元）	3031.81（亿元）	3172.22（亿元）
47.72	46.50	46.27	47.95	49.50	49.63	51.65	53.65	54.72	55.05	56.99
37.39	37.74	38.00	37.32	36.96	36.51	36.26	35.54	35.61	37.22	38.54

1-7 续表 2
Contiuned

指　标	Indicator	单位	Unit	2000 年	2005 年
高等学校在校学生	Students Enrollment in Instutions of Higher Education	万人	10 000 persons	4.61	23.98
小学专任教师	Full-time Teachers in Primary Schools	万人	10 000 persons	3.17	3.17
普通中学专任教师	Full-time Teachers in Regular Secorndary Schools	万人	10 000 persons	2.63	2.97
医生总数	Doctors	万人	10 000 persons	1.49	1.50
医院床位数	Hospital Beds	万张	10 000 beds	2.01	2.86
**人民生活**	People's Living Conditions				
就业人员工资总额	Total wages of Employed Persons	亿元	100 million yuan	120.40	359.5
城市居民人均可支配收入	Per Capita Disposable Income of Urban Households	元	yuan	8 016	12920
农民人均纯收入	Per Capita Net Income of Rural Households	元	yuan	3 637	5806
城乡人民币储蓄存款余额	Savings Deposit of Urban and Rural Households	亿元	100 million yuan	535.32	1343.10
**物价指数**	**Price Indexes**				
商品零售价格指数（以 1950 年价格为 100）	Retail Price Indes（1950=100）	%	%	520.5	495.0
居民消费价格指数（以 1950 年价格为 100）	Consumer Price Index（1950=100）	%	%	711.9	753.1
#食品价格指数（以 1950 年价格为 100）	Food Price Index（1950=100）	%	%	800.1	871.8

注：1. 就业人员工资总额 2005 年前统计范围为全部职工不含私营企业，2015 年及以前为在岗职工工资总额；
2 2015 年"城乡人民币储蓄存款余额"改为"住户存款"。

Note: 1.The total wages of the Full-Time Employees on duty were before 2005. The statistics ranged from all employees to private enterprises, Total wages for staff and workers in 2015 and before.
2. In 2015，"balance of RMB deposits in urban and rural areas" changed to "household deposits."

2008 年	2009 年	2010 年	2011 年	2012 年	2013 年	2014 年	2015 年	2016 年	2017 年	2018 年
26.93	27.52	28.49	29.15	29.66	30.02	31.35	32.23	34.09	34.62	39.8
3.24	3.22	3.2	3.16	3.17	3.28	3.22	3.45	3.42	3.47	3.69
2.99	3.06	3.08	3.14	3.20	3.18	3.2	3.29	3.39	3.47	3.6
1.63	1.67	1.77	1.83	2.16	2.41	2.5	2.63	2.76	3.09	3.46
2.99	3.05	3.34	3.56	4.12	3.97	4.3	4.51	4.73	4.63	5.08
578.7	638.1	738.0	877.5	1044.3	1251.7	1456.0	1644.4	1852.7	2034.7	2251.7
20464	22368	24998	28567	32145	35227	38294	40370	43598	47176	50817
8509	9249	10550	12370	13990	15731	17461	16730	17969	19364	20820
2123.36	2527.88	2912.33	3198.51	3757.60	4141	4436	5024	5326	5394	5914
526.6	519.2	526.5	550.2	559.6	567.4	580.5	580.5	592.1	596.8	607.6
831.4	835.6	854.0	896.7	920.9	943.9	968.4	980.0	1004.5	1024.6	1046.1
1105.3	1123.0	1194.9	1327.5	1384.6	1458.0	1522.2	1548.1	1625.5	1619.0	1669.2

1-8　国民经济主要结构指标
COMPOSITION INDICATORS ON NATIONAL ECONOMY

指　标	Indicator	2000 年	2005 年	2008 年	2009 年
**社会从业人员**	**Employment**				
产业结构	Industrial Composition				
第一产业	Primary Industry	36.4	22.2	19.9	20.1
第二产业	Secondary Industry	33.9	41.8	43.0	41.9
第三产业	Tertiary Industry	29.7	36.0	37.1	38.0
**全市生产总值**	**GDP**				
产业结构	Industrial Composition				
第一产业	Primary Industry	11.8	6.6	5.1	4.7
第二产业	Secondary Industry	46.6	51.8	50.8	49.9
第三产业	Tertiary Industry	41.6	41.6	44.1	45.4
**农业**	**Agriculture**				
农林牧渔业产值结构	Composition of Farming，Forestry，Animal Husbandry and Fishery				
# 农业	Farming	45.0	39.5	43.2	43.5
林业	Forestry	0.8	0.8	0.6	0.5
牧业	Animal Husbandry	27.9	33.4	29.9	27.9
渔业	Fishery	26.4	26.3	22.7	24.1
**工业**	**Industy**				
轻重工业产值结构	Composition of Output Value of Light and Heavy Industry				
轻工业	Light Industry	64.1	49.4	42.3	41.7
重工业	Heavy Industry	35.9	50.6	57.7	58.3
**固定资产投资**	**Investment in Fixed Assets**				
投资结构	Composition of Investment				
生产性	Productive	54.0	65.4	61.6	56.3
非生产性	Non-productive	46.0	34.6	38.4	43.7

注：2008 年 -2017 年农业数据为根据第三次农业普查结果核定修正后数据。
Note: Agricultural data from 2008 to 2017 are revised according to the results of the third agricultural census.

2010年	2011年	2012年	2013年	2014年	2015年	2016年	2017年	2018年
19.5	19.2	18.8	18.8	18.4	18.1	17.8	16.9	
41.4	41.2	41.0	40.6	39.0	38.5	38.0	37.8	
39.1	39.6	40.2	40.6	42.6	43.3	44.2	45.3	
4.8	4.6	4.4	4.2	3.9	3.8	3.5	3.4	3.2
49.1	48.1	47.1	46	45.4	44.0	42.3	41.2	40.4
46.1	47.3	48.5	49.8	50.7	52.2	54.2	55.4	56.4
45.8	40.9	40.0	41.7	42.1	40.3	42.5	43.0	44.7
0.4	0.4	0.4	0.4	0.4	0.4	0.4	0.4	0.5
25.5	28.1	26.6	25.5	24.8	25.0	24.2	23.8	22.1
24.6	26.7	28.8	28.0	27.7	29.6	26.6	26.2	26.1
38.7	38.3	39.5	39.5	38.8	39.5	39.4	37.6	34.3
61.3	61.7	60.5	60.5	61.2	60.5	60.6	62.4	65.7
53.6	53.2	58.4	61.65	57.08	63.16	59.81	53.64	
46.4	46.8	41.6	38.35	42.92	36.84	40.19	46.36	

1-9　平均每天主要社会经济活动

SELECTED INDICATORS ON AVERAGE DAILY SOCLAL AND ECONOMIC ACTIVITIES

指　标	Indicator	单位	Unit	2000 年	2005 年
全市生产总值	GDP	万元	10 000 yuan	32637	73629
工业总产值	Gross Industrial Output Value	万元	10 000 yuan	53174	137035
农林牧渔业总产值	Gross Output Value of Farming，Forestry，Animal Husbandry and Fishery	万元	10 000 yuan	6803	8781
主要工业产品产量	Output of Major Industrial Products				
发电量	Electricity	万千瓦时	10 000 kW・h	2489	2701
钢材	Rolled-steel	吨	ton	2558	9049
纱	Yarn	吨	ton	191	198
布	Cloth	万米	10 000 m	114	141
家用电冰箱	Refrigerator	台	unit	8523	24899
彩色电视机	Color TV set	台	unit	10395	36282
社会消费品零售总额	Total Retail Sales of Consumer Goods	万元	10 000 yuan	11734	23839
固定资产投资	Investment in Fixed Assets above Designated Size	万元	10 000 yuan	6649	38446
外贸进出口总额	Imports and Exports	万美元	10 000 USB	3707	9047
港口吞吐量	Volume of Freight Handled in Ports	万吨	10 000 tons	23.73	51.31

注：1. 固定资产投资包括城镇、农村 500 万元以上投资项目。
2. 一年按照 365 天计算。
3. 2008 年 -2017 年农林牧渔业总产值为根据第三次农业普查结果核定修正后数据。

Note :1. Investment in fixed assets includes construction projects involving an urban or rural investment of 5，000，00 yuan and over.
2. A year is computed according to 365 days.
3. Gross Output Value of Farming，Forestry，Animal Husbandry and Fishery from 2008 to 2017 is revised according to the results of the third agricultural census.

2008 年	2009 年	2010 年	2011 年	2012 年	2013 年	2014 年	2015 年	2016 年	2017 年	2018 年
120591	132983	157507	184267	203406	223214	242481	259518	279033	302030	328809
245116	280976	318215	363780	419351	462936	477924	493683	477143	356705	312048
10945	11153	13157	14646	15541	16728	17203	16777	18331	19120	20192
4509	4730	4984	4759	4787	4935	4885	4754	4805	4981	5271
9593	9579	9136	9101	7525	6312	5830	3853	5243	7996	8764
118	106	97	53	53	92	89	76	92	73	79
133	85	48	120	134	141	122	77	68	65	127
19813	22663	21951	19686	15754	14366	16714	23893	24170	22429	24290
22922	29170	30444	31622	39452	41426	46984	37704	57855	46658	46443
40883	47403	52130	63079	72209	81831	92102	101745	112464	124411	132670
55315	67367	82808	95960	113806	137751	157973	179608	204238	213071	
14695	12288	15633	19768	20057	21346	21887	19239	119196（万元）	137904（万元）	145787（万元）
82.27	86.76	96	104	114	125	131	136	141	141	149

1-10　主要指标占全国全省比重（2018 年）
PERCENTAGE OF MAIN INDICATORS TO CHINA AND SHANDONG（2018）

指　标	Indicator	单位	Unit
一、生产总值	GDP	亿元	100 million yuan
第一产业	Primary Industry	亿元	100 million yuan
第二产业	Secondary Industry	亿元	100 million yuan
第三产业	Tertiary Industry	亿元	100 million yuan
二、年末总人口（常住人口）	The Total Population（Resident Population）at the End of the Year	万人	10 000 persons
三、主要工业产品产量	Output of Major Industrial Products		
发电量	Electricity	亿千瓦小时	100 million kW·h
汽车	Automobile	万辆	10 000 vehicle
房间空气调节器	Air Conditioner	万台	10 000 units
家用电冰箱	Refrigerator	万台	10 000 units
彩色电视机	Color TV Set	万台	10 000 units
钢材	Rolled-steel	万吨	10 000 tons
水泥	Cement	万吨	10 000 tons
四、主要农产品产量	Output of Major Farm Products		
粮食	Grain	万吨	10 000 tons
油料	Oil Plants	万吨	10 000 tons
肉类产量	Meat	万吨	10 000 tons
水产品	Aquatic Products	万吨	10 000 tons
五、房地产开发投资	Investment In Real Estate Development	亿元	100 million yuan
六、货物进出口总额	Imports and Exports	亿元	100 million yuan
其中：出口	Export	亿元	100 million yuan
进口	Import	亿元	100 million yuan
七、高等学校在校学生	Students Enrollment of Institutions of Higher Education	万人	10 000 persons

注：1. 本表全国、全省均为公报数。
2. 青岛市主要工业产品产量数据为本地口径。

Note：1. The number of the country and province in the table are obtained from the statistical report.
2. The output of main industrial products of qingdao are at local calibre.

全国<br>China	全省<br>Shandong	青岛市<br>Qingdao	青岛市占全国比重（%）<br>Qingdao/China	青岛市占全省比重（%)<br>Qingdao/Shandong
900309	76469.7	12001.52	1.33	15.69
64734	4950.5	386.91	0.60	7.82
366001	33641.7	4850.59	1.33	14.41
469575	37877.4	6764.02	1.44	17.86
139538	10047.24	939.48	0.67	9.35
71118	5608.2	192.4	0.27	3.43
2782	136.3	9.24	0.33	6.78
20486	1060.8	1058.10	5.16	99.75
7993	888.4	886.58	11.09	99.80
18835	1695.2	1695.18	9.00	100.00
110552	9427.8	319.90	0.29	3.39
221000	12280.2	284.47	0.13	2.32
65789	5319.5	310.1	0.47	5.83
3439	310.9	38.5	1.12	12.38
8517	849.3	52.2	0.61	6.15
6469	816.6	103.5	1.60	12.67
120264	7553	1485.2	1.23	19.66
305050	19302.5	5321.2	1.74	27.57
164177	10569.6	3172.2	1.93	30.01
140874	8732.9	2149	1.53	24.61
2831.1	204.1	39.8	1.41	19.50

1-11 主要年份全市生产总值（按当年价格计算）

MAJOR YEAR'S GROSS DOMESTIC PRODUCT（AT CURRENT PRICE）

单位：亿元（100 million yuan）

年份 Year	全市生产总值 GDP	第一产业 Primary Industry	第二产业 Secondary Industry	#工 业 Industry	第三产业 Tertiary Industry	人均生产总值（元）Per Capita GDP（yuan）
1949	2.87	1.19	1.10		0.58	71
1952	6.74	1.76	3.25	2.95	1.73	163
1957	10.82	2.00	5.76	5.39	3.06	239
1962	9.69	1.11	4.68	4.27	3.90	205
1965	15.50	1.80	8.96	8.48	4.74	325
1970	23.23	3.15	14.46	13.86	5.62	451
1975	29.07	6.93	15.39	14.67	6.75	522
1978	38.43	8.73	20.25	19.15	9.45	663
1980	48.65	10.19	26.27	24.28	12.19	819
1985	82.28	21.29	37.55	34.03	23.44	1311
1988	142.87	32.72	70.32	63.42	39.83	2199
1990	180.77	39.26	86.76	78.94	54.75	2714
1991	205.65	43.04	98.94	89.64	63.67	3053
1992	261.35	43.92	130.27	117.61	87.16	3856
1993	371.90	62.20	183.35	164.35	126.35	5455
1994	510.81	85.12	244.30	218.87	181.39	7436
1995	631.45	112.53	294.43	263.98	224.49	9089
1996	713.60	133.27	322.33	289.87	258.00	10130
1997	802.59	117.47	378.25	342.80	306.87	11235
1998	901.19	140.66	409.60	369.89	350.93	12443
1999	1018.97	138.19	468.67	424.15	412.11	13884
2000	1191.25	140.85	555.21	504.44	495.19	16009

1-11 续表 1
Continued

单位：亿元（100 million yuan）

年份<br>Year	全市生产总值<br>GDP	第一产业<br>Primary Industry	第二产业<br>Secondary Industry	#工　业<br>Industry	第三产业<br>Tertiary Industry	人均生产总值（元）<br>Per Capita GDP（yuan）
2001	1368.55	144.35	643.44	582.99	580.76	18128
2002	1583.51	147.21	758.33	686.55	677.97	20655
2003	1869.44	148.92	923.76	832.31	796.76	23986
2004	2270.16	163.49	1149.94	1032.50	956.73	28540
2005	2687.46	178.33	1392.02	1259.06	1117.10	33085
2006	3183.18	183.95	1666.96	1517.28	1332.28	38608
2007	3750.16	203.59	1934.52	1766.28	1612.05	44964
2008	4401.56	223.40	2234.83	2034.25	1943.33	52266
2009	4853.87	230.25	2420.14	2174.43	2203.48	57251
2010	5749.02	276.99	2823.43	2519.00	2648.60	65827
2011	6725.76	306.38	3236.92	2880.76	3182.46	76804
2012	7424.32	324.41	3496.46	3135.54	3603.45	84063
2013	8147.32	340.50	3745.12	3356.16	4061.70	91376
2014	8850.54	349.62	4014.99	3559.81	4485.93	98283
2015	9472.40	363.98	4168.11	3689.57	4940.31	104418
2016	10184.70	359.30	4309.65	3802.65	5515.75	111302
2017	11024.11	368.85	4546.21	3952.87	6109.05	119215
2018	12001.52	386.91	4850.59	4137.08	6764.02	128459

注：1. 人均生产总值按常住人口计算。
2. 自 2005 年起三次产业分类采用《国民经济行业分类》（GB/T4754-2002）标准，自 2013 年起，根据《国民经济行业分类》（GB/T4754—2011）和《三次产业划分规定》（国统字［2012］108 号），将农林牧渔业中的农林牧渔服务业以及工业中的开采辅助活动和金属制品、机械和设备修理业归入第三产业。
3. 全国第三次农业普查后，对历年 GDP 数据进行了修订（以下相关表同）。
4. 2018 年 GDP 数据为初步核算数（以下相关表同）。

Note：1. Per capita GDP are calculated at permanent population.
2. Three Industries are grouped by "Classification and Code of the Sectors of the National Economy"（GB/T4754-2002）since 2005，from 2013 on，According to the Industrial Classification for National Economic Activities（GB/4754-2011）and the Rules for Classification of Three Industries（NBS No.［2012］108），the service industries of Agriculture，Forestry，Animal Husbandry and Fishery and mining auxiliary activities and metal products，machinery and equipment repair industries in the Industry are classified into the tertiary industry.
3. According to the the Third Agricultural Census,the date of GDP over the years have been revised (The same applies to tables following) .
4. GDP data for 2018 are preliminary accounting figures(The same applies to tables following).

1-12　主要年份全市生产总值构成（以全市生产总值为 100）

COMPOSITION OF MAJOR YRAR'S GROSS DOMESTIC PRODUCT
（GROSS DOMESTIC PRODUCT=100）

单位：%

年份 Year	全市生产总值 GDP	第一产业 Primary Industry	第二产业 Secondary Industry	#工　业 Industry	第三产业 Tertiary Industry
1952	100	26.1	48.2	43.8	25.7
1957	100	18.5	53.3	49.8	28.2
1962	100	11.4	48.3	44.1	40.3
1965	100	11.6	57.8	54.7	30.6
1970	100	13.6	62.2	59.7	24.2
1975	100	23.9	52.9	50.5	23.2
1978	100	22.7	52.7	49.8	24.6
1980	100	21.0	54.0	49.9	25.1
1985	100	25.9	45.6	41.4	28.5
1988	100	22.9	49.2	44.4	27.9
1990	100	21.7	48.0	43.7	30.3
1991	100	20.9	48.1	43.6	31.0
1992	100	16.8	49.8	45.0	33.4
1993	100	16.7	49.3	44.2	34.0
1994	100	16.7	47.8	42.8	35.5
1995	100	17.8	46.6	41.8	35.6
1996	100	18.7	45.2	40.6	36.2
1997	100	14.6	47.1	42.7	38.2
1998	100	15.6	45.5	41.0	38.9
1999	100	13.6	46.0	41.6	40.4
2000	100	11.8	46.6	42.3	41.6
2001	100	10.5	47.0	42.6	42.4
2002	100	9.3	47.9	43.4	42.8
2003	100	8.0	49.4	44.5	42.6
2004	100	7.2	50.7	45.5	42.1
2005	100	6.6	51.8	46.8	41.6
2006	100	5.8	52.4	47.7	41.8
2007	100	5.4	51.6	47.1	43.0
2008	100	5.1	50.8	46.2	44.1
2009	100	4.7	49.9	44.8	45.4
2010	100	4.8	49.1	43.8	46.1
2011	100	4.6	48.1	42.8	47.3
2012	100	4.4	47.1	42.2	48.5
2013	100	4.2	46.0	41.2	49.8
2014	100	3.9	45.4	40.2	50.7
2015	100	3.8	44.0	39.0	52.2
2016	100	3.5	42.3	37.3	54.2
2017	100	3.4	41.2	35.9	55.4
2018	100	3.2	40.4	34.5	56.4

1-13 主要年份全市生产总值增长速度（以上年为 100）

GROWTH RATE OF MAJOR YEAR'S GROSS DOMESTIC PRODUCT (PRECEDING YEAR=100)

单位：%

年份 Year	全市生产总值 GDP	第一产业 Primary Industry	第二产业 Secondary Industry	第三产业 Tertiary Industry	人均生产总值（元） Per Capita GDP（yuan）
1979	12.0	10.6	12.9	11.4	10.4
1980	10.4	4.3	13.1	10.3	9.4
1981	–2.0	–9.1	–2.4	4.7	–3.2
1982	4.1	5.5	–0.2	11.9	2.6
1983	16.9	42.8	9.3	12.7	15.3
1984	13.0	18.9	11.5	10.3	12.0
1985	9.9	5.4	5.8	21.3	9.3
1986	9.2	2.6	12.1	10.0	8.3
1987	12.0	3.6	16.0	12.0	10.6
1988	13.8	–0.3	21.5	11.2	12.1
1990	9.3	9.8	7.3	12.2	8.0
1991	10.6	9.9	11.0	10.5	9.4
1992	18.1	2.7	23.5	20.7	17.4
1993	22.4	20.5	21.1	25.6	21.7
1994	14.4	3.6	14.7	20.2	13.5
1995	12.0	10.9	12.1	12.4	10.8
1996	7.2	7.7	7.6	6.4	5.7
1997	11.5	–10.6	16.8	14.6	9.9
1998	12.9	17.6	11.3	13.4	11.3
1999	13.9	4.1	15.5	15.3	12.4
2000	15.2	6.5	16.4	16.4	13.6
2001	13.7	2.3	16.3	14.2	12.1
2002	14.5	3.2	16.9	14.6	12.7

1-13　续表 1
Continued

单位：%

年份<br>Year	全市生产总值<br>GDP	第一产业<br>Primary Industry	第二产业<br>Secondary Industry	第三产业<br>Tertiary Industry	人均生产总值（元）<br>Per Capita GDP（yuan）
2003	16.3	2.5	20.1	14.9	14.4
2004	16.7	2.7	20.3	15.2	14.3
2005	16.6	0.4	19.6	15.7	14.1
2006	15.3	0.9	16.8	15.8	13.6
2007	15.5	–2.6	15.3	18.3	14.2
2008	13.2	1.4	11.1	17.1	12.1
2009	12.2	3.0	12.8	12.5	11.4
2010	12.9	1.4	12.6	14.4	11.2
2011	11.8	5.0	11.9	12.5	11.5
2012	10.6	3.2	11.3	10.5	9.6
2013	10.0	2.0	10.1	10.6	9.0
2014	8.0	3.8	8.3	8.1	7.0
2015	8.1	3.2	7.3	9.3	7.3
2016	7.9	2.9	6.7	9.2	6.9
2017	7.5	3.2	6.8	8.4	6.4
2018	7.4	3.5	7.3	7.7	6.3
1978–2010 平均每年增长<br>1978–2010 Average Increase Rate	12.4	5.3	13.1	13.9	11.0
1991–2010 平均每年增长<br>1991–2010 Average Increase Rate	14.2	4.2	15.5	15.3	12.8
1996–2010 平均每年增长<br>1996–2010 Average Increase Rate	13.8	2.5	15.2	14.5	13.0
2001–2010 平均每年增长<br>2001–2010 Average Increase Rate	14.7	1.5	16.1	15.2	13.0
2006–2010 平均每年增长<br>2006–2010 Average Increase Rate	13.8	0.8	13.7	15.6	12.5
2010–2015 平均每年增长<br>2010–2015 Average Increase Rate	9.7	2.8	9.8	10.3	8.9

1-14　分市、区生产总值（2018年）
GROSS DOMESTIC PRODUCT BY REGION（2018）

单位：亿元（100 million yuan）

市、区名称	Region	地区生产总值<br>GDP	第一产业<br>Primary Industry	第二产业<br>Secondary Industry	第三产业<br>Tertiary Industry
市南区	Shinan District	1203.37		115.12	1088.25
市北区	Shibei District	832.20		174.26	657.94
李沧区	Licang District	453.53		132.96	320.57
崂山区	Laoshan District	697.00	8.32	314.58	374.10
黄岛区	Huangdao District	3387.72	73.70	1542.68	1771.34
城阳区	Chengyang District	1079.02	3.28	538.87	536.87
即墨区	Jimo District	1413.43	70.84	761.72	580.87
胶州市	Jiaozhou	1211.38	53.27	603.94	554.17
平度市	Pingdu	895.85	101.36	461.48	333.01
莱西市	Laixi	620.11	61.88	299.50	258.73
红岛经济区	Qingdao National High-tech Industrial Development Zone	107.36	14.27	49.24	43.85
保税港区	Qingdao Free Trade Port Area of Chinat	129.35		35.52	93.83

1-15　按支出法计算的全市生产总值（2018 年）

GROSS DOMESTIC PRODUCT BY EXPENDITURE APPROACH（2018）

单位：亿元（100 million yuan）

项目	Item	2018 年	2018 年为 2017 年 %<br>2018/2017（%）
**支出法计算的全市生产总值**	**Gross Domestic Product by Expenditure Approach**	**12001.52**	**107.4**
（一）最终消费	Final Consumption Expenditures	4570.93	109.1
1. 居民消费	Household Consumption Expenditures	3325.15	109.0
农村居民	Rural Household	554.72	108.6
城镇居民	Urban Household	2770.42	109.1
2. 政府消费	Government Consumption Expenditures	1245.78	109.4
（二）资本形成总额	Gross Capital Formation	6998.65	105.3
1. 固定资产形成总额	Gross Fixed Capital Formation	6659.32	104.6
2. 存货增加	Change in Inventories	339.33	119.6
（三）货物和服务净流出	Net Exports of Goods and Services	431.94	114.9

注：绝对额按当年价格计算，速度按可比价格计算。
Note：The absolute numbers are calculated at current price，their growth are calculated at constant price.

1-16　全市生产总值构成（2018年）
COMPOSITION OF GROSS DOMESTIC PRODUCT（2018）

指标	Indicator	增加值（亿元）<br>Added Value<br>（10 000 million yuan）	2018年为2017年%<br>2018/2017（%）
**地区生产总值**	**GDP**	**12001.52**	**107.4**
农林牧渔业	Farming，Forestry，Animal Husbandry and Fishery	410.56	103.6
#农林牧渔服务业	Agriculture，Forestry，Animal Husbandry and Fishery Service Industry	23.65	106.6
工业	Industry	4137.08	106.9
#开采辅助活动	#Mining Auxiliary Activities		
#金属制品、机械和设备修理业	#Metal Products，Machinery and Equipment Pepair Industries	9.89	106.9
建筑业	Construction	723.40	110.5
批发和零售业	Wholesale and Retail Trade	1440.66	107.3
批发业	Wholesale	940.64	108.1
零售业	Retail Trade	500.02	105.9
交通运输、仓储和邮政业	Transport，Storage and Post	830.58	102.7
住宿和餐饮业	Hotels and Catering Services	268.18	105.7
住宿业	Hotels	44.89	104.4
餐饮业	Catering Services	223.29	106.0
金融业	Financial Intermediation	800.40	105.2
房地产业	Real Extate	672.46	101.2
房地产业（K门类）	Real Extate	443.75	99.2
自有房地产经营活动	Self-Owned Real Estate Business Activities	228.71	105.0
其他服务业	Other Services	2718.20	112.2
营利性服务业	For-Profit Services	1416.11	119.6
非营利性服务业	Non-Profit Services	1302.09	104.8
第一产业	Primary Industry	386.91	103.5
第二产业	Secondary Industry	4850.59	107.3
第三产业	Tertiary Industry	6764.02	107.7

主要统计指标解释

国内生产总值（GDP）　指按市场价格计算的一个国家（或地区）所有常住单位在一定时期内生产活动的最终成果。国内生产总值有三种表现形态，即价值形态、收入形态和产品形态。从价值形态看，它是所有常住单位在一定时期内生产的全部货物和服务价值超过同期中间投入的全部非固定资产货物和服务价值的差额，即所有常住单位的增加值之和；从收入形态看，它是所有常住单位在一定时期内创造并分配给常住单位和非常住单位的初次收入分配之和；从产品形态看，它是所有常住单位在一定时期内最终使用的货物和服务价值与货物和服务净出口价值之和。在实际核算中，国内生产总值有三种计算方法，即生产法、收入法和支出法。三种方法分别从不同的方面反映国内生产总值及其构成。对于一个地区来说，称为地区生产总值或地区 GDP。

人均 GDP　人均 GDP 是一定时期内 GDP 与同期人口平均数的比值。按照国际标准，人口平均数应该是同期平均常住人口。我国在核算制度中也规定，无论是国家还是地区，人口数都采用常住人口。国家统计局规定从 2004 年开始，过去采用户籍人口计算人均 GDP 的地区，作为过渡性措施，可在两年内同时计算两种口径的人均 GDP（数据后面必须注明是什么口径），两年后取消按户籍人口计算的人均 GDP。我市采用常住人口计算人均 GDP。

三次产业　是根据社会生产活动历史发展的顺序对产业结构的划分，产品直接取自自然界的部门称为第一产业，对初级产品进行再加工的部门称为第二产业，为生产和消费提供各种服务的部门称为第三产业。它是世界上通用的产业结构分类，但各国的划分不尽一致。我国的三次产业划分是：

第一产业：是指农业、林业、畜牧业、渔业和农林牧渔服务业。

第二产业：是指采矿业，制造业，电力、燃气及水的生产和供应业；建筑业。

第三产业：除第一、第二产业以外的其他行业。

支出法国内生产总值　是从最终使用角度反映一个国家（或地区）一定时期内生产活动最终成果的一种方法，包括最终消费支出，资本形成总额及货物和服务净出口三部分。对于地区，名称为“支出法地区生产总值”。

最终消费指常住单位为满足物质、文化和精神生活的需要，从本国经济领土和国外购买的货物和服务的支出；不包括非常住单位在本国经济领土内的消费支出。最终消费分为居民消费和政府消费。

居民消费　指常住住户在一定时期内对货物和服务的全部最终消费支出。居民消费支出除了直接以货币形式购买货物和服务的消费之外，还包括以其他方式获得的货物和服务的消费支出，即所谓的虚拟消费支出。居民虚拟消费支出包括以下几种类型：单位以实物报酬及实物转移的形式提供给劳动者的货物和服务；住户生产并由本住户消费了的货物和服务，其中的服务仅指住户的自有住房服务和付酬的家庭雇员提供的家庭和个人服务；金融机构提供的金融媒介服务。

政府消费　指政府部门为全社会提供公共服务的消费支出和免费或以较低价格向居民住户提供的货物和服务的净支出。前者等于政府服务的产出价值减去政府单位所获得的经营收入的价值；后者等于政府部门免费或以较低价格向居民住户提供的货物和服务的市场价值减去向住户收取的价值。

资本形成总额　指常住单位在一定时期内获得的减去处置的固定资产和存货的净额，包括固定资本形成总额和存货增加。

固定资本形成总额　指常住单位在一定时期内获得的固定资产减处置的固定资产的价值总额。固定资产是通过生产活动生产出来的，且使用年限在一年以上、单位价值在规定标准以上的资产，不包括自然资产。分有形固定资产形成总额和无形固定资产形成总额。有形固定资产形成总额包括一定时期内完成的建筑工程、安装工程和设备工器具购置（减处置）价值，以及土地改良、新增役、种、奶、毛、娱乐用牲畜和新增经济林木价值。无形固定资产形成总额包括矿藏的勘探、计算机软件等获得减处置。

存货增加　指常住单位在一定时期内存货实物量变动的市场价值，即期末价值减期初价值的差额，再扣除当期由于价

格变动而产生的持有收益。存货增加可以是正值，也可以是负值；正值表示存货上升，负值表示存货下降。它包括生产单位购进的原材料、燃料和储备物资等存货，以及生产单位生产的产成品、在制品和半成品等存货。

货物和服务净出口 指货物和服务出口减货物和服务进口的差额。出口包括常住单位向非常住单位出售或无偿转让的各种货物和服务的价值；进口包括常住单位从非常住单位购买或无偿得到的各种货物和服务的价值。由于服务活动的提供与使用同时发生，一般把常住单位从国外得到的服务作为进口，非常住单位从本国得到的服务作为出口。货物的出口和进口都按离岸价格计算。

气候 指地球与大气之间长期能量交换与质量交换所形成的一种自然环境状态，它是多种因素综合作用的结果。气候既是人类生活和生产的环境要素之一，又是供给人类生活和生产的重要资源。气温、降水、湿度等气象要素的多年平均值是用来描述一个地区气候状况的主要参数，而各种气象要素某年、某月的平均值（或总量）则可以反映出该时期天气气候状况的重要特征。

气温 指空气的温度，我国一般以摄氏度（℃）为单位表示。气象观测的温度表是放在离地面约 1.5 米处通风良好的百叶箱里测量的，因此，通常说的气温指的是离地面 1.5 米处百叶箱中的温度。其统计计算方法为：月平均气温是将全月各日的平均气温相加，除以该月的天数而得。

年平均气温 是将 12 个月的月平均气温累加后除以 12 而得。

降水量 指从天空降落到地面的液态或固态（经融化后）水，未经蒸发、渗透、流失而在地面上积聚的深度。其统计计算方法为：月降水量是将全月各日的降水量累加而得。年降水量是将 12 个月的月降水量累加而得。

Explanatory Notes on Main Statistical Indicators

Gross Domestic Product（GDP） refers to the final products at market prices producted by all resident units in a country（or a region）during a certain period of time. Gross domestic product is expressed in three different persperctives，namely value，income，and products respectively. GDP in its value perspective refers to the total value of all goods and services produced by all resident units during a certain period of time，minus the total value of input of goods and services of the nature of non-fixed assets；in other words，it is the sum of the value-added of all resident units. GDP from the perspective of products refers to the value of all goods and services for final consumption by all resident units minus the net exports of goods and services during a given period of time. In the practice of national accounting，gross domestic product is calculated from three approaches，namely production approach，income approach and expenditure approach，which reflect gross domestic product and its composition from different angles. For a certain region，it refers region gross product or region GDP.

Per Capita GDP refers to the ratio of GDP in a certain term and average population in the same term. According to the international standard，average population should be average permanent population in the same term. In the account regulation，population of both the country and the region should be permanent population. In the after two yers since 2004，per capita GDP can be calculated at two coverage.i.e. at permanent population and household registered population（the note of coverage should follow the data.）The per capita GDP calculated with household registered population will be abolished after two years. Per capita GDP of Qingdao is calculated at permanent population.

Three Strata of Industries has been classfied according to the historical sequence of development. Primary industry refers to extraction of natural resources；secondary industry involves processing of primary products；and tertiary industry provides services of various kinds for production and consumption. The above classification is universal although it varies to some extent form country to country. In China economic activities are categorized into the following three strata of industry：

Primary industry refers to farming，forestry，animal husbandryand fishery and services in support of these industries.

Secondary industry refers to mining and quarrying, manufacturing, production and supply of electricity, water and gas, and construction.

Tertiary industry refers to all other economic activities not included in primary or secondary industries.

GDP by Expenditure Approach refers to the method of measuring the final results of production activities of a country (region) during a given period from the perspective of final use. It includes final consumption, total capital formation and net export of goods and services. It reflects use and composition of gross domestic product. For a certain region, it refers region gross product by expenditure approach.

Final Consumption refers to the total expenditure of resident units for purchases of goods and services from both the domestic economic territory and abroad to meet the needs of material, cultural and spiritual life. It does not include the expenditure of non-resident units on consumption in the economic territory of the country. The final consumption is broken down into household consumption and government consumption.

Household Consumption refers to the total expenditure of resident households on the final consumption of goods and services. In addition to the consumption of goods and services bought by the households directly with money, the household consumption also includes expenditure on goods and services obtained by the households in other ways, i.e. the so-called imputed consumption, which includes the following: (a) the goods and services provided to households by employers in the form of payment in kind and transfer in kind; (b) goods and services produced and consumed by the households themselves, in which the services refer to the owner-occupied housing and services offered by payed family employees; (c) financial intermediate services provided by financial institution.

Government Consumption refers to the consumption expenditure spent for the provision of public services provided by the government to the whole country and the net expenditure on the goods and services provided by the government to households free of charge or at reduced prices. The former equals to the output value of the government services minus the value of operating income obtained by the government departments. The latter equals to the market value of the goods and services provided by the government free of charge or at reduced prices to the households minus the value received by the government from the households.

Gross Capital Formation refers to the fixed assets acquired less disposal and the net value of inventory, thus including the gross fixed capital formation and charges in inventories.

Gross Fixed Capital Formation refers to the value of acquisitions less those disposals of fixed assets during a given period. Fixed assets are the assets produced through production activities with unit value above a specified amount and which could be used for over one year. Natural assets are not included. Gross fixed capital formation can be categorized into total tangible fixed capital formation and total intangible fixed capital formation. Total tangible fixed capital formation includes the value of the construction projects and installation projects completed and the equipment, apparatus and instruments purchased (less those disposed) as well as the value of land improved, the value of draught animals, breeding stock and animals for milk, for wool and for recreational purposes and the newly increased forest with economic value. Total intangible fixed capital formation includes the prospecting of minerals and the acquisition of computer software minus the disposal of them.

Charges in Inventories refers to the market value of the change in the physical volume of inventory of resident units during a given period, i.e. the difference between the values at the beginning and at the end of the period minus the gains due to the change in prices. The changes in inventories can have a positive or a negative value. A positive value indicates an increase in inventory while a negative value indicates a decrease in inventory. The inventory includes raw materials, fuels and reserve materials purchased by the production units as well as the inventory of finished products, semi-finished products and work-in-progress.

Net Export of Goods and Services refers to the exports of goods and services subtracting the imports of goods and services.

Exports include the value of various goods and services sold or gratuitously transferred by resident units to non-resident units. Imports include the value of various goods and services purchased or gratuitously acquired resident units from non-resident units. Because the provision of services and the use of them happen simultaneously, the acquisition of services by resident units from abroad is usually treated as import while the acquisition of services by non-resident units in this country is usually treated as export. The exports and imports of goods are calculated at FOB.

Climate refers to the natural environmental status formed by the long-term exchange of energy and mass between the earth and the air, and is the results of interaction of many factors. Climate is both one of the environment factors and the important resources for the living and production activities of the human being. The average values across several years of meteorological factors such as temperature, rainfall and humidity are used as important parameters to describe the climate of a region, while the average values (or total values) of a given year or month of meteorological factors reflect the key characteristics of climate for that period of time.

Temperature refers to the air temperature. China uses centigrade as the unit. The thermometry used for weather observation is put in a breezy shutter, which is 1.5 meters high from the ground. Therefore, the commonly used temperature refers to the temperature in the breezy shutter 1.5 meters away from the ground. The calculation method is as follows:

Monthly average temperature is the summation of average daily temperature of one month divided by the actual days of that particular month.

Annual average temperature is the summation of monthly average of a year divided by 12 months.

Volume of Precipitation refers to the deepness of liquid state or solid state (thawed) water falling from the sky to the ground that has not been evaporated, infiltrated or run off. The calculation method is as follows:

Monthly precipitation is the summation of daily precipitation of a month.

Annual precipitation is the summation of 12 months precipitation of a year.

人 口 2

POPULATION

简要说明

一、本篇资料的主要内容

本篇资料主要反映了全市人口方面的基本情况，包括全市主要年份和区（市）的户数、人口数、人口密度、土地面积数据、人口出生率、死亡率、自然增长率、计划生育等数据。另外还对建国以来开展的六次人口普查主要数据进行了比较。

二、本篇资料的来源

本篇资料分别来源于国家开展的人口普查、人口抽样调查和市公安局的户籍登记资料，由市统计局人口和社会科技统计处整理提供。

Brief Introduction

I. Main Content

Data in this chapter show the basic condition of population, such as the basic condition of districts and county-level cities, household, population, density of population, birth rate, death rate, natural growth rate and family planning situation. Furthermore, relevant figures obtained from the six national population censuses have been compared.

II. Source of Data

Data in this chapter are from national population censuses, national sample survey. Some are derived from household registration provided by Qingdao Municipal Bureau of Public Security. The data are compiled by the Division of Population and Science & Technology of Qingdao Municipal Bureau of Statistics.

2-1　主要年份全市户籍人口数
MAJOR YEAR'S TOTAL REGISTERED POPULATION

单位：人（person）

年份 Year	总人口 Total Population	按性别分 Grouped by Sex		平均人口 Average Population
		男 Male	女 Female	
1949	4056550	1997293	2059257	4054431
1952	4233586	2125392	2108194	4203428
1957	4822756	2424729	2398027	4773862
1962	4627432	2319658	2307774	4578313
1965	4901670	2461629	2440041	4859712
1970	5391852	2720633	2671219	5337282
1975	5742206	2904347	2837859	5717456
1978	5853321	2959800	2893521	5841638
1980	5961129	3015357	2945772	5937187
1985	6267223	3185409	3081814	6253157
1988	6516920	3319534	3197386	6474541
1989	6571597	3348454	3223143	6544259
1990	6666482	3392253	3274229	6619040
1991	6709277	3411776	3297501	6687880
1992	6731072	3420886	3310186	6720175
1993	6753497	3431149	3322348	6742285
1994	6785291	3446019	3339272	6769394
1995	6846346	3476300	3370046	6815819
1996	6902677	3502600	3400077	6874512
1997	6954391	3527588	3426803	6928534
1998	6995666	3545403	3450263	6975029
1999	7029707	3561061	3468646	7012687
2000	7066481	3577367	3489114	7048094
2001	7104875	3595652	3509223	7085678
2002	7156537	3619778	3536759	7130706
2003	7206806	3644032	3562774	7181672
2004	7311228	3692991	3618237	7259017
2005	7409052	3740309	3668743	7360140
2006	7493812	3779891	3713921	7451432
2007	7579910	3816620	3763290	7536861
2008	7615647	3824665	3790982	7597779
2009	7629161	3823534	3805627	7622404
2010	7636392	3819240	3817152	7632777
2011	7663612	3826931	3836681	7650002
2012	7695585	3838205	3857380	7679599

注：截止出书前，未获得公安部门青岛市 2013 年 -2018 年户籍人口信息。
Note：Data come from the Qingdao Nunicipal Public Security Bureau，and the Data of 2013-2018 are absent.

2-2 主要年份全市户数、人口数、人口密度（户籍）

MAJOR YEAR'S HOUSEHOLDS，POPULATION AND DENSITY OF POPULATION（WITH PERMANENT RESIDENCE）

年份 Year	总户数（万户） Total Households （10000 households）		总人口（万人） Total Population （10000 persons）		平均每户人口（人） Average Persons Per Household（person）		人口密度（人/平方公里） Density of Population （person/sq.km）	
	全市 Whole Municipality	市区 Urban Area	全市 Whole Municipality	市区 Urban Area	全市 Whole Municipality	市区 Urban Area	全市 Whole Municipality	市区 Urban Area
1949	90.64	21.68	405.66	103.69	4.48	4.78	381	941
1952	93.96	22.70	423.36	107.62	4.51	4.74	397	977
1957	102.56	27.41	482.28	134.76	4.70	4.92	453	1223
1962	106.11	29.29	462.74	137.86	4.36	4.71	434	1251
1965	105.31	29.64	490.17	146.74	4.65	4.95	460	1332
1970	113.07	32.14	539.19	150.81	4.77	4.69	506	1369
1975	123.18	36.56	574.22	161.13	4.66	4.41	539	1462
1978	131.27	40.08	585.33	168.00	4.46	4.19	549	1525
1980	138.44	43.03	596.11	175.27	4.31	4.07	560	1591
1985	157.06	53.43	626.72	190.87	3.99	3.57	588	1732
1988	169.72	58.95	651.69	201.38	3.84	3.42	612	1827
1989	177.47	61.57	657.16	203.63	3.70	3.31	617	1848
1990	186.79	63.94	666.65	205.78	3.57	3.22	626	1867
1991	192.46	67.15	670.93	207.22	3.49	3.09	630	1880
1992	199.08	68.94	673.11	209.28	3.38	3.04	632	1899
1993	201.36	70.13	675.35	212.06	3.35	3.02	634	1924
1994	204.05	71.24	678.53	214.97	3.33	3.02	637	1951
1995	208.13	72.53	684.63	218.38	3.29	3.01	643	1982
1996	212.40	75.11	690.27	223.86	3.25	2.98	648	2032
1997	215.12	76.39	695.44	227.22	3.23	2.97	653	2062
1998	218.59	77.90	699.57	229.58	3.20	2.95	657	2084
1999	222.61	79.27	702.97	231.94	3.16	2.93	660	2105
2000	224.73	80.46	706.65	234.60	3.14	2.92	664	2129
2001	227.43	81.41	710.49	237.60	3.12	2.92	667	2156
2002	229.67	82.85	715.65	241.74	3.12	2.92	672	2194
2003	232.26	84.15	720.68	246.77	3.10	2.93	677	2240
2004	235.29	86.93	731.12	258.40	3.11	2.97	686	2229
2005	237.35	87.99	740.91	265.43	3.12	3.02	695	2290
2006	239.33	89.12	749.38	271	3.13	3.04	703	2338
2007	241.55	90.21	757.99	275.55	3.14	3.05	711	2377
2008	243.18	91.22	761.56	276.25	3.13	3.03	715	2384
2009	244.74	92.15	762.92	275.47	3.12	2.99	676	1873
2010	246.11	93.30	763.64	275.50	3.10	2.95	677	1873
2011	247.94	94.69	766.36	277.09	3.09	2.93	679	1884
2012	249.27	95.87	769.56	279.57	3.09	2.92	682	1901

2-3　主要年份全市常住人口数
MAJOR YESR’S TOTAL RESIDENT POPULATION

单位：万人（10 000 person）

年份<br>Year	总人口<br>Total population
2005	819.55
2006	829.42
2007	838.67
2008	845.61
2009	850.03
2010	871.51
2011	879.51
2012	886.85
2013	896.41
2014	904.62
2015	909.70
2016	920.40
2017	929.05
2018	939.48

2-4　青岛市常住人口
TOTAL RESIDENT POPULATION

单位：万人（10 000 person）

市、区名称	Region	2018 年	2017 年	2018 年比 2017 年 ±<br>2018/2017（±）
**全市**	**Whole Municipality**	**939.48**	**929.05**	**10.43**
市南区	Shinan District	58.83	58.53	0.30
市北区	Shibei District	110.11	109.32	0.79
李沧区	Licang District	57.74	56.12	1.62
崂山区	Laoshan District	44.59	43.87	0.72
黄岛区	Huangdao District	157.73	153.92	3.81
城阳区	Chengyang District	72.05	70.93	1.12
即墨区	Jimo District	123.83	122.45	1.38
胶州市	Jiaozhou	90.05	89.33	0.72
平度市	Pingdu	137.89	138.05	–0.16
莱西市	Laixi	76.29	76.42	–0.13
红岛经济区	Qingdao National High-tech Industrial Development Zone	10.37	10.11	0.26

注：黄岛区含保税港区数据。
Note：The Huangdao District contains the data of the bonded harbor area.

2-5　主要年份常住人口城镇化率
THE URBANIZATION RATE OF THE MAIN YEAR PERMANENT RESIDENTS

单位：%（%）

年份<br>Year	城镇化率<br>Urbanization rate
2005	63.20
2006	63.59
2007	63.90
2008	64.31
2009	64.68
2010	65.81
2011	66.52
2012	67.14
2013	67.72
2014	68.41
2015	69.99
2016	71.53
2017	72.57
2018	73.67

注：城镇化率为城镇区域常住人口数量与全市常住总人口的比值。
Note: The urbanization rate is the ratio of the number of permanent urban residents to the total resident population of the city.

2-6　分市、区土地面积（2018 年底）
LAND AREA（END OF 2018）

市、区名称	Region	土地面积（平方公里）Land Area（aq.km）
**全　市**	**Whole Municipality**	**11293.36**
市南区	Shinan District	32.21
市北区	Shibei District	65.85
李沧区	Licang District	99.10
崂山区	Laoshan District	395.79
黄岛区	Huangdao District	2128.31
城阳区	Chengyang District	583.68
即墨区	Jimo District	1920.90
胶州市	Jiaozhou	1323.65
平度市	Pingdu	3175.65
莱西市	Laixi	1568.22

注：2017 年度土地变更调查结果。
Note：Survey results of land quality change in 2017.

2-7 第一、二、三、四、五、六次人口普查主要数据
MAIN DATA FROM THE SIX NATIONAL POPULATION CENSUSES

项目	Item	第一次人口普查 1st National Population Census	第二次人口普查 2nd National Population Census	第三次人口普查 3rd National Population Census	第四次人口普查 4th National Population Census	第五次人口普查 5th National Population Census	第六次人口普查 6th National Population Census
**一、总人口（万人）**	**Total Population（10000 persons）**	**91.68**	**138.34**	**422.76**	**666.40**	**749.42**	**871.51**
按性别分	By Sex						
男（万人）	Male（10000 persons）	48.44	69.64	214.09	338.86	375.74	439.18
女（万人）	Female（10000 persons）	43.24	68.71	208.67	327.54	373.68	432.33
**二、总户数（万户）**	**Total Household（10000 Household）**	**19.27**	**28.01**	**101.41**	**186.61**	**241.62**	**296.64**
家庭户（万户）	Family Household（10000 Household）			101.09	185.56	232.70	282.43
平均家庭户规模（人）	Average Family Household Size（person）			4.07	3.49	2.97	2.79
**三、民　族**	**Ethnicity**						
民族个数（个）	Number of Fthnic groups（unit）	14	16	25	41	51	53
汉族人口（万人）	Populaiton of Han（10000 persons）	91.33	137.76	422.09	665.58	746.12	863.84
少数民族人口（万人）	Populaiton of Ethnic Minorities（10000 persons）	0.36	0.58	0.67	0.82	3.30	7.67
**四、各种文化程度人口**	**Popution with Various Education Attainments**						
大　学（万人）	College（10000 persons）		1.51	3.62	12.39	41.64	129.54
高　中（万人）	Senior Secondary School（10000 persons）		4.86	34.82	68.42	111.40	150.82
初　中（万人）	Junior Secondary School（10000 persons）		13.72	102.28	195.10	281.91	334.22
小　学（万人）	Premary School（10000 persons）		51.25	144.52	236.34	206.91	170.77

2-8 计划生育情况（1978-2018年）
FAMILY PLANNING SITUATION（1978-2018）

年份<br>Year	已婚有生育能力的人数（人）<br>Married and Fertile Persons（person）	节育人数（人）<br>Persons under Birth Control（person）	节育率（%）<br>Contraceptive Prevalence Rate（%）	四项手术人数（人）<br>Persons Performed Four-operation（person）	#放环人数<br>Persons Set Rings
1978	694292	571077	82.25	125913	68475
**1980**	**763226**	**673069**	**88.19**	**180979**	**70927**
1985	1017649	929284	91.32	146742	67234
**1990**	**1258232**	**1165216**	**92.61**	**149327**	**70964**
1991	1319722	1219795	92.43	153970	77406
1992	1348503	1256271	93.16	90107	56226
1993	1380717	1271884	92.12	76783	45492
1994	1400425	1268378	90.57	73169	41482
1995	1427442	1285264	90.04	84099	52716
1996	1459465	1316516	90.21	84370	57648
1997	1482204	1340399	90.43	76579	55285
1998	1505499	1367648	90.84	74199	50526
1999	1523471	1378131	90.46	73663	44976
**2000**	**1521331**	**1371447**	**90.15**	**74285**	**45999**
2001	1514274	1360543	89.85	70205	43601
2002	1541855	1400145	90.81	50575	45453
2003	1518448	1354917	89.23	57458	47953
2004	1540706	1357703	88.04	52934	42859
2005	1590476	1417755	89.14	58097	49716
2006	1604665	1427785	88.98	51864	42457
2007	1595390	1426787	89.43	50074	41198
2008	1587344	1410426	88.85	120539	93291
2009	1623969	1418178	87.33	111084	100193
**2010**	**1591103**	**1377726**	**86.59**	**78721**	**51794**
2011	1581231	1355808	85.74	70823	48210
2012	1575996	1349572	85.63	62880	43021
2013	1527731	1293810	84.69	53252	30851
2014	1506274	1275142	84.66	34008	12827
2015	1475887	1226439	83.10	34879	9108
2016	1456953	1183221	81.21	36449	4474
2017	1439008	1188307	82.58	20905	4669
2018	1406047	1159775	82.48	17786	5097

2-9　分市、区计划生育情况（2018 年）
FAMILY PLANNING SITUATION BY REGION（2018）

市、区名称	Region	计划生育率（%）Fertility Rate（%）	计划内出生（人）Birth within the Plan（person）		计划外出生（人）Birth without the Plan（person）		多胎（人）Third Birth and Above（person）
			一胎 First Birth	二胎 Second Birth	一胎 First Birth	二胎 Second Birth	
**全市**	**Whole Municipality**	**98.29**	**35981**	**49652**	**194**	**1606**	**2666**
市南区	Shinan District	99.55	2982	2055	7	113	73
市北区	Shibei District	99.76	4420	3662	1	57	90
李沧区	Licang District	99.55	2696	3046	4	42	58
崂山区	Laoshan District	99.87	1505	1638	1	27	33
黄岛区	Huangdao District	97.84	6532	9808	52	381	603
城阳区	Chengyang District	99.37	2777	4023	5	128	130
即墨区	Jimo District	98.43	4291	7509	8	151	394
胶州市	Jiaozhou	97.59	3169	5620	13	167	381
平度市	Pingdu	96.68	4975	8165	66	370	642
莱西市	Laixi	97.75	2634	4126	37	170	262

注：黄岛区含保税港区数据；城阳区含红岛经济区数据。
Note：The Huangdao area contains the data of the bonded harbor area.Chengyang District contains red is land economic zone data.

主要统计指标解释

人口数 指一定时点、一定地区范围内有生命的个人总和。

出生率（又称粗出生率） 指在一定时期内（通常为一年）一定地区的出生人数与同期内平均人数（或期中人数）之比，用千分率表示。本资料中的出生率指年出生率，其计算公式为：

$$出生率=\frac{年出生人数}{年平均人数}\times 1000‰$$

式中：出生人数指活产婴儿，即胎儿脱离母体时（不管怀孕月数），有过呼吸或其他生命现象。

死亡率（又称粗死亡率） 指在一定时期内（通常为一年）一定地区的死亡人数与同期内平均人数（或期中人数）之比，用千分率表示。本资料中的死亡率指年死亡率，其计算公式为：

$$死亡率=\frac{年死亡人数}{年平均人数}\times 1000‰$$

人口自然增长率 指在一定时期内（通常为一年）人口自然增加数（出生人数减死亡人数）与该时期内平均人数（或期中人数）之比，用千分率表示。计算公式为：

$$人口自然增长率=\frac{本年出生人数-本年死亡人数}{年平均人数}\times 1000‰$$

$$=人口出生率-人口死亡率$$

Explanatory Notes on Main Statistical Indicators

Total Population refers to the total number of people alive at a certain point of time within a given area.

Birth Rate（or Crude Birth Rate） refers to the ratio of the number of births to the average population（or mid-period population）during a certain period of time（usually a year）, expressed in ‰. Birth rate in the chapter refers to annual birth rate. The following formula is used：

Birth Rate=（Number of Births/Average Number of Population）× 1000‰

Number of births in the formula refers to live births，i.e. when a baby has breathed or showed any vital phenomena regardless of the length of pregnancy.

Annual average number of population is the average of the number of population at the beginning of the year and that at the end of the year. Sometimes it is substituted by the mid-year population.

Death Rate（or Crude Death Rate） refers to the ratio of the number of deaths to the average population（or mid-period population）during a certain period of time（usually a year）, expressed in ‰. Death rate in the chapter refers to annual death rate. The following formula is used：

Death Rate=（Number of Deaths/Annual Average Number of Population）× 1000‰

Natural Growth Rate of Population refers to the ratio of natural increase in population（number of births minus number of deaths）in a certain period of time（usually a year）to the average population（or mid-period population）of the same period, expressed in ‰. The following formula is applied：

Natural Growth Rate of Population=［（Number of Births-Number of Deaths）/Average Number of Population］× 1000‰

Natural Growth Rate of Population=Birth Rate-Death Rate

从业人员及职工工资 3

EMPLOYMENT AND WAGES

简要说明

一、本篇资料的主要内容

本篇资料主要反映了全市从业人员和职工工资方面的基本情况，主要包括全市社会从业人数及各区（市）单位从业人数、在岗职工工资总额和平均工资等方面的资料。

二、本篇资料的来源

单位从业人员和职工工资来源于劳动统计年报，全市从业人员根据劳动统计年报、农村年报和市工商行政管理局、市人力资源和社会保障局、市交通委相关资料测算。由市统计局人口和社会科技统计处整理提供。

Brief Introduction

I. Main Content

Data in this chapter show the basic conditions of Qingdao' s employment and wages，including the main data on labor statistics of employment，number of employment of the whole city and 12 district and county-level cities，total wage bill and average wage of staff and workers，etc.

II. Source of Data

Data on employment and wages are prepared according to the annual reports of labor statistics.Data on social employment are measured according to the annual reports of labor statistics，countryside statistics and related information from Industry & Commerce Administration Bureau，Human Resources & Social Security Bureau and Transportation Commision.

Data in this chapter are compiled by the Division of Population and Science & Technology of Qingdao Municipal Bureau of Statistics.

3-1 社会就业人数（1978-2017 年）
SOCIAL EMPLOYMENT（1978–2017）

单位：万人（10 000 persons）

年份<br>Year	合计<br>Total	第一产业<br>Primary Industry	第二产业<br>Secondary Industry	第三产业<br>Tertiary Industry
1978	256.70	157.40	65.60	33.70
1979	265.60	156.10	72.20	37.30
1980	270.10	153.00	77.30	39.80
1981	278.90	153.60	82.10	43.20
1982	282.70	151.10	86.20	45.40
1983	289.60	156.40	85.00	48.10
1984	304.20	149.20	97.70	57.40
1985	315.30	149.20	104.50	61.70
1986	326.40	149.60	112.10	64.60
1987	334.80	149.90	120.20	64.70
1988	336.50	147.60	123.20	65.70
1989	344.20	152.50	122.10	69.60
1990	352.80	156.50	123.30	73.10
1991	361.20	161.90	124.30	74.90
1992	370.30	161.70	127.80	80.80
1993	365.70	160.50	128.80	76.40
1994	367.60	157.10	129.40	81.20
1995	374.20	155.40	132.40	86.40
1996	381.50	152.20	137.10	92.20
1997	388.70	155.20	138.10	95.30
1998	393.10	154.80	138.60	99.70
1999	396.10	150.30	142.00	103.80
2000	397.60	144.70	134.91	117.99
2001	400.50	133.90	142.30	124.30
2002	413.31	121.38	153.85	138.08
2003	438.96	119.91	163.93	155.12
2004	458.81	113.98	180.63	164.20
2005	471.03	104.48	196.80	169.75
2006	490.10	102.97	209.83	177.30
2007	505.80	102.30	217.80	185.70
2008	513.80	102.40	220.80	190.60
2009	525.71	105.65	220.33	199.73
2010	540.34	105.16	223.85	211.33
2011	551.18	106.02	227.08	218.08
2012	559.88	104.95	229.79	225.14
2013	571.47	107.59	232.17	231.71
2014	588.97	108.45	229.91	250.61
2015	595.44	107.92	229.51	258.01
2016	601.44	107.30	228.44	265.70
2017	603.90	101.86	228.14	273.90

3-2 主要年份全市单位就业人员人数

MAJOR YEAR' S NUMBER OF EMPLOYED PERSONS IN ALL UNITS OF THE CITY

单位：万人（10 000 persons）

年份 Year	合计 Total	# 市区 Urban Area	# 国有单位 State-owned Units	# 集体单位 Collective-owned Units	其他所有制单位 Other Ownership Units
1949	14.8	13.4	9.3	5.5	
1952	23.4	20.1	18.3	5.1	
1957	29.7	24.5	24.1	5.6	
1962	32.3	26.8	24.5	7.8	
1965	36.7	30.6	26.8	9.9	
1970	45.6	37.4	32.2	13.4	
1975	53.4	43.5	38.0	15.4	
1978	70.7	54.5	49.2	21.5	
1980	79.2	61.5	55.0	24.2	
1985	88.8	66.7	59.8	28.9	0.1
1988	98.2	70.0	66.7	31.2	0.3
1990	103.2	70.6	69.5	32.9	0.8
1991	105.4	78.4	71.5	32.5	1.4
1992	108.2	80.4	73.6	32.8	1.8
1993	109.4	80.8	75.3	29.5	4.6
1994	110.1	80.2	72.4	28.3	9.4
1995	111.6	80.2	72.4	27.5	11.7
1996	118.4	81.1	71.6	26.0	20.8
1997	117.1	78.1	69.0	22.6	25.5
1998	116.2	75.6	64.2	19.0	33.0
1999	119.0	78.3	60.4	16.6	42.0
2000	118.3	76.6	57.6	14.2	46.5
2001	117.5	75.1	53.2	12.2	52.1
2002	119.1	75.2	49.5	10.3	59.3
2003	118.0	74.3	47.2	8.9	61.9
2004	207.1	111.1	43.7	18.0	145.4
2005	224.3	122.3	43.9	18.2	162.2
2006	243.2	133.7	41.7	16.2	185.3
2007	249.8	137.3	45.1	17.9	186.8
2008	254.1	140.9	44.4	15.4	194.3
2009	260.5	145.6	44.4	14.6	201.5
2010	269.4	151.1	44.6	15.1	209.7
2011	275.9	156.5	44.3	14.7	216.9
2012	283.2	162.1	44.0	13.8	225.4
2013	293.7	184.1	37.8	5.4	250.5
2014	303.0	191.4	36.7	4.9	261.4
2015	309.2	192.4	35.8	4.6	268.8
2016	315.6	196.4	35.5	4.7	275.4
2017	321.4	249.1	35.1	4.6	281.7
2018	330.4	258.3	34.7	2.0	293.7

注：1.2004 年以前为城镇单位职工人数 .
　　2.2013 年统计口径变化，国有单位、集体单位数据与上年不可比。

Note：1. Before 2004，the data refer to number of staff and workers in urban units.
　　2. Because of the adjustment about the statistics system，the data of State-owned Units and Collective-owned Units since 2013are not fully comparable with historical statistics.

3-3　全市单位国民经济各行业就业人员人数（2018 年）

NUMBER OF EMPLOYED PERSONS IN ALL UNITS OF THE CITY BY SECTOR（2018）

单位：万人（10 000 persons）

		2018 年		2017 年	
		合计 Total	女性 Female	合计 Total	女性 Female
总计	**Total**	330.4	125.2	321.4	125.7
1. 农、林、牧、渔业	Farming，Forestry，Animal Husbandry and Fishery	1.2	0.4	1.2	0.5
2. 采 矿 业	Mining	0.1		0.1	
3. 制 造 业	Manufacturing	133.4	48.4	139.4	52.9
4. 电力、热力、燃气及水生产和供应业	The Electricity，Heat，Gas and Water Production and Supply Industry	2.5	0.6	2.6	0.6
5. 建筑业	Construction	32.7	5.2	29.4	4.7
6. 批发和零售业	Wholesale and Retail Trade	53.0	24.4	48.8	23.7
7. 交通运输、仓储和邮政业	Transport，Storage and Post	15.5	4.9	12.8	4.1
8. 住宿和餐饮业	Hotels and Catering Services	7.0	3.6	6.9	3.6
9. 信息传输、软件和信息技术服务业	Information Transmission Computer Services and Software	4.7	1.8	4.2	1.6
10. 金融业	Financial Intermediation	6.4	3.6	6.4	3.5
11. 房地产业	Real Estate	8.8	3.4	8.1	3.0
12. 租赁和商务服务业	Leasing and Business Services	16.0	6.0	14.7	5.7
13. 科学研究和技术服务业	Scientific Research and Technical Services	7.5	2.5	6.7	2.1
14. 水利、环境和公共设施管理业	Management of Water Conservancy，Environmentand Public Facilities	3.7	1.2	3.3	1.0
15. 居民服务、修理和其他服务业	Residents Service，RePair and Other Services	2.7	1.3	2.6	1.0
16. 教育	Education	14.1	8.3	14.4	8.4
17. 卫生和社会工作	Health and Social Welfare	8.0	5.5	7.7	5.3
18. 文化、体育和娱乐业	Culture，Sports and Entertainment	1.8	0.9	1.9	0.8
19. 公共管理、社会保障和社会组织	Public Management，Social Security and SocialOrganization	11.3	3.2	10.2	3.2

3-4 全市单位分市、区国民经济各部门就业人员人数（2018年）

NUMBER OF EMPLOYED PERSONS IN ALL UNTTS OF THE CITY BY REGION AND SECTOR（2018）

市、区名称	Region	合计 Total	农林牧渔业 Farming，Forestry，Animal Husbandryand Fishery	采矿业 Mining	制造业 Manufacturing	电力热力燃气及水生产和供应业 The Electricity，Heat，Gas and Water Production and Supply Industry
**全市**	**Whole Municipality**	**330.4**	**1.2**	**0.1**	**133.4**	**2.5**
市南区	Shinan District	39.9			3.1	0.3
市北区	Shibei District	27.2			2.7	0.5
李沧区	Licang District	16.4			3.9	0.1
崂山区	Laoshan District	24.1	0.1		6.1	0.1
黄岛区	Huangdao District	72.5	0.3		28.3	0.4
城阳区	Chengyang District	38.6	0.1		25.8	0.1
即墨区	Jimo District	32.7	0.1		20.5	0.4
胶州市	Jiaozhou	34.2	0.1		18.5	0.4
平度市	Pingdu	16.1	0.2	0.1	9.4	0.1
莱西市	Laixi	21.8	0.3		11.7	0.1
红岛经济区	Qingdao National High-tech Industrial Development Zone	3.7			1.7	
保税港区	Qingdao Free Trade Port Area of China	3.2			1.7	

单位：万人（10 000 persons）

建筑业 Construction	批发和零售业 Wholesale and Retail Trade	交通运输仓储和邮政业 Transport，Storage and Post	住宿和餐饮业 Hotels and Catering Services	信息传输软件和信息技术服务业 Information Transmission Computer Services and Software
**32.7**	**53.0**	**15.5**	**7.0**	**4.7**
2.5	9.1	2.6	2.0	1.9
3.1	7.3	2.9	0.4	0.4
0.8	3.8	0.6	1.0	0.1
4.1	2.4	1.4	0.6	1.6
9.7	14.3	4.5	1.5	0.4
2.1	2.9	1.3	0.8	0.1
2.2	3.9	0.3	0.3	
2.6	5.9	0.8	0.1	0.1
0.8	0.9	0.1	0.2	
4.5	1.6	0.1		
0.3	0.3	0.1	0.1	0.1
	0.6	0.8		

3-4 续表
Continued

市、区名称	Region	金融业 Financial Intermediation	房地产业 Real Estate	租赁和商务服务业 Leasing and Business Services	科学研究和技术服务业 Scientific Research and Technical Services	水利环境和公共设施管理业 Management of Water Conservancy, Environment and Public Facilities
**全市**	**Whole Municipality**	**6.4**	**8.8**	**16.0**	**7.5**	**3.7**
市南区	Shinan District	5.7	1.4	3.5	1.5	0.5
市北区	Shibei District		1.2	2.4	0.8	0.3
李沧区	Licang District		0.5	2.1	0.5	0.5
崂山区	Laoshan District	0.7	0.8	0.8	2.4	0.5
黄岛区	Huangdao District		1.8	3.3	1.1	0.6
城阳区	Chengyang District		0.7	1.4	0.5	0.5
即墨区	Jimo District		0.6	0.3	0.1	0.3
胶州市	Jiaozhou		0.8	1.3	0.2	0.1
平度市	Pingdu		0.7	0.1		0.2
莱西市	Laixi		0.2	0.5		0.2
红岛经济区	Qingdao National High-tech Industrial Development Zone		0.1	0.3	0.4	
保税港区	Qingdao Free Trade Port Area of China					

单位：万人（10 000 persons）

居民服务修理和其他服务业 Services to Households and Other Services	教育 Education	卫生和社会工作 Health and Social Welfare	文化体育和娱乐业 Culture，Sports and Entertainment	公共管理社会保障和社会组织 Public Management，Social Security and Social Organization	国际组织 International Organization
**2.7**	**14.1**	**8.0**	**1.8**	**11.3**	
0.4	1.7	1.5	0.4	1.8	
0.5	1.7	1.7	0.4	0.9	
0.3	1.0	0.6	0.2	0.4	
0.1	1.0	0.3	0.4	0.7	
0.4	2.8	0.9	0.3	1.9	
0.2	1.0	0.4		0.7	
0.2	1.3	0.9	0.1	1.2	
0.1	1.2	0.6		1.4	
0.2	1.5	0.5		1.1	
0.2	0.8	0.6		1.0	
0.1	0.1			0.1	
				0.1	

3-5　全市单位分市、区全部就业人员人数（2018 年底）

NUMBER OF EMPLOYED PERSONS IN ALL UNITS OF THE CITY BY REGION（END OF 2018）

单位：万人（10 000 persons）

市、区名称	Region	年末人数 Year-end Population	国有单位 State-owned Units	集体单位 Collective-owned Units	其他经济类型单位 Units in Other Types of Economy
**全市**	**Whole Municipality**	**330.4**	**34.7**	**2.0**	**293.7**
市南区	Shinan District	39.9	7.2	0.3	32.4
市北区	Shibei District	27.2	4.4	0.2	22.6
李沧区	Licang District	16.4	1.8	0.1	14.5
崂山区	Laoshan District	24.1	2.2	0.1	21.8
黄岛区	Huangdao District	72.5	5.6	0.1	66.8
城阳区	Chengyang District	38.6	2.0	0.1	36.5
即墨区	Jimo District	32.7	2.6	0.6	29.5
胶州市	Jiaozhou	34.2	3.4	0.2	30.6
平度市	Pingdu	16.1	2.9	0.2	13.0
莱西市	Laixi	21.8	2.3	0.1	19.4
红岛经济区	Qingdao National High-tech Industrial Development Zone	3.7	0.2		3.5
保税港区	Qingdao Free Trade Port Area of China	3.2	0.1		3.1

3-6 全市就业人员工资总额、平均工资(1978-2018年)

TOTAL WAGES AND AVERAGE WAGE OF CITYWIDE FULL-TIME EMPLOYEES (1978-2018)

年份 Year	工资总额(亿元) Total Wage Bill (100 million yuan)		平均工资(元) Avergae Wage (yuan)	
	全社会单位 All Units	其中:非私营单位 of which : Non-private Units	全社会单位 All Units	其中:非私营单位 of which : Non-private Units
1978		4.0		584
1979		4.8		662
1980		6.0		782
1981		6.3		778
1982		6.6		785
1983		6.9		809
1984		9.0		1041
1985		9.6		1103
1986		11.8		1311
1987		14.2		1519
1988		18.0		1862
1989		20.7		2120
1990		24.4		2400
1991		26.4		2553
1992		31.8		2970
1993		40.4		3694
1994		59.5		5455
1995		68.3		6164
1996		78.2		6640
1997		82.5		7030
1998		87.2		7518
1999		100.1		8405
2000		120.4		10072
2001		135.8		11426
2002		153.8		12839
2003	225.6	160.3	12597	15335
2004	280.5	193.4	13932	17190
2005	359.5	242.8	16015	20022
2006	445.6	292.0	18574	23457
2007	518.1	338.6	21419	27083
2008	578.7	371.9	23296	30233
2009	638.1	393.2	25396	33258
2010	738.0	446.8	28549	37805
2011	877.5	522.0	32763	43077
2012	1044.3	613.1	37399	49052
2013	1251.7	785.7	42688	55363
2014	1456.0	890.5	48453	62104
2015	1644.4	993.0	53715	69465
2016	1852.7	1088.5	58551	75803
2017	2034.7	1193.6	63047	82177
2018	2251.7	1290.8	68197	89525

注:1.2003年以前为职工工资总额、平均工资。
2.2015年以前为在岗职工工资总额、平均工资。

Note : 1. The data before 2003 refer to the total wage bill and average wage of stall and workers.
2. It was the total salary and average salart of the on-spot staff before 2015.

3-7 全市就业人员工资总额、平均工资指数（1978-2018 年）
INDEXES OF TOTAL WAGES AND AVERAGE WAGE OF CITYWIDE FULL-TIME EMPLOYEES（1978-2018）

年份 Year	工资总额指数 Total Wage Bill Indexed		平均工资指数 Avergae Wage Indexes	
	全社会单位 All Units	其中：非私营单位 of which：Non-private Units	全社会单位 All Units	其中：非私营单位 of which：Non-private Units
1978		120.80		104.66
1979		119.53		113.36
1980		123.95		118.13
1981		106.71		99.49
1982		103.80		100.90
1983		104.14		103.06
1984		130.64		128.68
1985		107.11		105.96
1986		123.22		118.86
1987		120.10		115.87
1988		126.48		122.58
1989		115.29		113.86
1990		117.66		113.21
1991		108.28		106.38
1992		120.26		116.33
1993		127.09		124.38
1994		147.46		147.67
1995		114.76		113.00
1996		114.54		107.72
1997		105.50		105.87
1998		105.64		106.94
1999		114.81		111.80
2000		120.29		119.83
2001		112.77		113.44
2002		113.31		112.37
2003		108.84		110.30
2004	124.34	120.65	110.60	112.10
2005	128.16	125.54	114.95	116.47
2006	123.95	120.26	115.98	117.16
2007	116.27	115.96	115.32	115.46
2008	111.70	109.83	108.76	111.63
2009	110.26	105.73	109.01	110.01
2010	115.66	113.63	112.42	113.67
2011	118.90	116.83	114.76	113.95
2012	119.01	117.45	114.15	113.87
2013	119.86	128.15	114.14	112.87
2014	116.32	113.34	113.50	112.18
2015	112.94	111.51	110.86	111.85
2016	110.25	106.49	109.71	110.33
2017	109.82	109.66	107.68	108.41
2018	110.66	108.14	108.17	108.94

注：1.2004 年以前为职工工资总额、平均工资指数。
2.2015 年以前为在岗职工工资总额、平均工资。

Note：1. The data before 2004 refer to the indexes of total wage of stall and workers.
2. It was the total salary and average salart of the on-spot staff before 2015.

3-8　分行业就业人员平均工资（2018 年）

AVERAGE WAGE OF FULL-TIME EMPLOYEES BY INDUSTRY（2018）

行业	Sector	全部单位（元）All Units（yuan）	非私营单位（元）Non-private Units（yuan）	私营单位（元）Private Units（yuan）
**总计**	**Total**	**68197**	**89525**	**51976**
（一）农、林、牧、渔业	Farming，Forestry，Animal Husbandry	40922	62087	39405
（二）采矿业	Mining	44739	47046	37115
（三）制造业	Manufacturing	58455	72284	49538
（四）电力、热力、燃气及水生产和供应业	The Electricity，Heat，Gas and Water Production and Supply Industry	77673	86461	54059
（五）建筑业	Construction	62364	69447	55402
（六）批发和零售业	Wholesale and Retail Trade	54037	67985	51360
（七）交通运输、仓储和邮政业	Transport，Storage and Post	78289	89768	58625
（八）住宿和餐饮业	Hotels and Catering Services	48320	57303	43217
（九）信息传输、软件和信息技术服务业	Information Transmission Computer Services and Software	101179	118509	89056
（十）金融业	Financial Intermediation	136656	145388	59844
（十一）房地产业	Real Estate	73370	94910	51253
（十二）租赁和商务服务业	Leasing and Business Services	58715	74057	55104
（十三）科学研究和技术服务业	Scientific Research and Technical Services	90648	121088	64298
（十四）水利、环境和公共设施管理业	Management of Water Conservancy，Environment and Public Facilities	55114	63390	45520
（十五）居民服务、修理和其他服务业	Residents Service，RePair and Other Services	50081	80554	45581
（十六）教育	Education	122615	125322	64242
（十七）卫生和社会工作	Health and Social Welfare	111966	123463	50340
（十八）文化、体育和娱乐业	Culture，Sports and Entertainment	87377	95094	71331
（十九）公共管理、社会保障和社会组织	Public Management，Social Security and Social Organization	123726	123726	

3-9 分市、区就业人员工资总额（2018 年）

TOTAL WAGES OF FULL-TIME EMPLOYEES BY CITY OR DISTRICT (2018)

单位：亿元（100 million yuan）

市、区名称	Region	合计 Total	国有单位 State-owned Units	集体单位 Collective-owned Units	其他所有制单位 Other Owership Units
**全市**	**Whole Municipality**	**2251.7**	**428.4**	**17.6**	**1805.7**
市南区	Shinan District	362.1	104.7	2.8	254.6
市北区	Shibei District	205.7	59.6	0.9	145.2
李沧区	Licang District	104.2	20.9	0.7	82.6
崂山区	Laoshan District	210.3	28.9	0.4	181.0
黄岛区	Huangdao District	462.2	67.4	0.9	393.9
城阳区	Chengyang District	260.1	29.5	1.0	229.6
即墨区	Jimo District	189.4	27.9	7.4	154.1
胶州市	Jiaozhou	194.0	33.2	1.1	159.7
平度市	Pingdu	91.4	30.6	1.2	59.6
莱西市	Laixi	124.9	23.1	1.2	100.6
红岛经济区	Qingdao National High-tech Industrial Development Zone	22.9	1.5		21.4
保税港区	Qingdao Free Trade Port Area of China	24.5	1.1		23.4

3-10 分市、区就业人员平均工资(2018年)

AVERAGE WAGE OF FULL-TIME EMPLOYEES BY CITY OR DISTRICT (2018)

单位：元/年(yuan/year)

市、区名称	Region	合计 Total	国有单位 State-owned Units	集体单位 Collective-owned Units	其他所有制单位 Other Owership Units
**全市**	**Whole Municipality**	**68197**	**124324**	**89742**	**61704**
市南区	Shinan District	91683	144682	92312	79670
市北区	Shibei District	75753	135006	52142	64335
李沧区	Licang District	63018	117940	59766	56380
崂山区	Laoshan District	87257	133996	66615	82713
黄岛区	Huangdao District	65009	121635	75620	60193
城阳区	Chengyang District	66960	145961	91191	62543
即墨区	Jimo District	57891	109670	116028	52175
胶州市	Jiaozhou	57052	99649	63991	52362
平度市	Pingdu	57422	105454	73840	46388
莱西市	Laixi	56595	101123	114543	51132
红岛经济区	Qingdao National High-tech Industrial Development Zone	60800	80292	96611	59744
保税港区	Qingdao Free Trade Port Area of China	73302	164597		71493

主要统计指标解释

单位从业人员 指报告期末最后一日24时在本单位中工作，并取得工资或其他形式劳动报酬的人员数。该指标为时点指标，不包括最后一日当天及以前已经与单位解除劳动合同关系的人员，是在岗职工、劳务派遣人员及其他就业人员之和。就业人员不包括：

（1）离开本单位仍保留劳动关系，并定期领取生活费的人员；

（2）利用课余时间打工的学生及在本单位实习的各类在校学生；

（3）本单位因劳务外包而使用的人员。

在岗职工 指在本单位工作且与本单位签订劳动合同，并由单位支付各项工资和社会保险、住房公积金的人员，以及上述人员中由于学习、病伤、产假等原因暂未工作仍由单位支付工资的人员。在岗职工还包括：

（1）应订立劳动合同而未订立劳动合同人员（如使用的农村户籍人员）；

（2）处于试用期人员；

（3）编制外招用的人员；

（4）派往外单位工作，但工资仍由本单位发放的人员（如挂职锻炼、外派工作等情况）。

工资总额 指根据《关于工资总额组成的规定》（1990年1月1日国家统计局发布的一号令）进行修订，在报告期内（季度或年度）直接支付给本单位全部就业人员的劳动报酬总额。包括计时工资、计件工资、奖金、津贴和补贴、加班加点工资、特殊情况下支付的工资，是在岗职工工资总额、劳务派遣人员工资总额和其他就业人员工资总额之和。

工资总额是税前工资，包括单位从个人工资中直接为其代扣或代缴的房费、水费、电费、住房公积金和社会保险基金个人缴纳部分等。

工资总额不论是计入成本的还是不计入成本的，不论是以货币形式支付的还是以实物形式支付的，均应列入工资总额的计算范围。

平均工资 指单位就业人员在一定时期内平均每人所得的工资额。它表明一定时期工资收入的高低程度，是反映就业人员工资水平的主要指标。

Explanatory Notes on Main Statistical Indicators

Jobholders refers to the number of employees who works in the institution or company and gets salary or other form of labor reward at 24：00 of the last day of the report period. This is a time-point indicator which is the total of on-spot staff，labor dispatched personnel and other employees，excluding persons that have released the employment contract with the institution or company on the last day or bedore. The following are not deemed as Jobholders：

（1）Persons that keep the employment relations and get regular living expenst after leaving the institution or company；

（2）Various students who work part time or as internship；

（3）Personnel that uses by the institution or company in labour outsourcing.

On-spot staff refers to the employees that works for and signs the employment contract with the institution or company，and relevant salary，social insurance and house funding are paid by the institution or company，and the persons of the previous employees that are not working but still gets salary from the institution or company due to the reason of learning，injury，maternity leave，etc. It also includes the following：

（1）Employees who shall sign the employment contract with institution or company but not signed（for example，employees of rural household registration）.

（2）Employees that are still in probation period；

（3）Employees that are recruited outside the authorized size；

（4）Employees that are sent to other institution or company but the salaries are still paid by the institution or company（such as onsite training and labour dispatching）.

Total salary refers to total payment for labour made directly to all jobholders of the institution or company within the report period（quarterly or annually）revised according to Regulations on Constitution of Total Salary，Order No. 1 released by National Bureau of Statistics on January 1，1990，including hourly wage，piece wage，bonus，allowance and subsidy，overtime wage，and wages paid under special situations. It is also the total volume of on-spot staff，labour dispatching staff and other employment.

Total salary is the pre-tax salary，including the expenses of housing，water，power and the personal part of house funding and social insurance funds，which has been withheld and paid by the institution or company from personal salaries.

No matter it is credited into cost or not，no matter it is paid in currency or in kind，all total salary shall be categorized as total salary.

Average salary refers to the total salary per capita of employees of the institution or company within the certain period，which is the major indicator of the salary level of employees and the level of salary income of the certain salary.

固定资产投资 4

INVESTMENT IN FIXED ASSETS

简要说明

一、本篇资料的主要内容

本篇资料主要反映了全市固定资产投资及房地产开发方面的情况，主要包括固定资产投资的规模、结构、资金来源和房地产开发投资、施竣工及销售情况等方面的资料。

二、本篇资料的来源

本篇资料来源于固定资产投资及房地产开发投资统计年报，由市统计局固定资产投资统计处整理提供。

Brief Introduction

I. Main Content

Data in this chapter show the basic conditions of investment in fixed assets and real estate development of the whole city，mainly including the total investment in fixed assets，the structure of investment，the resources of investment and investment in real estate development，construction，completion，sales，etc.

II. Source of Data

Data in this chapter are based on the annual report on investment in fixed assets and real estate development，and provided by the Division of Investment and Construction Statistics of Qingdao Municipal Bureau of Statistics.

4-1 主要年份固定资产投资
MAJOR YEAR'S INVESTMENT IN FIXED ASSETS

单位：万元（10 000 yuan）

年份 Year	规模以上固定资产投资总额 Investment in Fixed Assets above Designated Size	按用途分 Crouped by Use		
		生产性投资 Productive Investment	非生产性投资 Non-Productive Investment	# 住宅投资 Investment in Residential Buildings
1949	16	12	4	3
1952	3097	1368	1729	1007
1957	5923	3947	1976	1151
1962	2747	2049	698	192
1965	5481	4155	1326	375
1970	6100	5813	287	154
1975	15839	13548	2381	1064
1978	29660	23499	6161	3203
1980	47499	33865	13634	9335
1985	107421	60643	73424	19058
1990	284576	210453	74123	38898
1991	349031	256633	92398	51898
1992	583068	420748	162320	83844
1993	953274	511684	441590	162419
1994	1329716	728532	601184	324094
1995	1648281	925194	723087	390206
1996	1610562	972398	638164	285412
1997	1611902	932207	679695	278951
1998	1909711	1125821	783890	316999
1999	2205891	1246328	959563	421151
2000	2426820	1311538	1115282	470963
2001	2934728	1487762	1446966	729181
2002	3683623	1962588	1721035	765030
2003	5475526	3023930	2451596	1038304
2004	9845646	6336279	3509367	1608738
2005	14032960	9172206	4860754	1841644
2006	14856894	9214578	5642316	2222733
2007	16353636	10231673	6121963	2720970
2008	20190098	12443179	7746919	3315538
2009	24588889	13852653	10736236	3831976
2010	30224785	16207885	14016900	5609189
2011	35025382	18622819	16402563	5707625
2012	41539146	24246803	17292343	6689114
2013	50278649	30996768	19281881	7220432
2014	57660308	32913906	24746402	7311090
2015	65556685	41402714	24153971	7569123
2016	74547006	44588200	29958806	9561782
2017	77770902	41719893	36051009	9255095

注：1. 规模以上固定资产投资数据 2003 年以前为城镇以上统计范围。
2. 因 2004 年以来数据有调整，故表中数据不可比。

Note：1.The data of investment above designated size refer to investment above city and town level before 2003.
2.The data since 2004 are not comparable with other data because of the adjustment.

4-1 续表
Continued

单位：万元（10 000 yuan）

年份 Year	按构成分 Gouped by Composition of Funds			按资金来源分 Grouped by Sources of Funds			
	建筑安装工程 Constrection and Installation	设备工器具购置 Purchase of Equipment and Instruments	其他费用 Others	国家投资 State Investment	国内贷款 Domestic Loans	利用外资 Foreign Investment	自筹及其他 Self-raising Fund and Others
1949	11	3	2	16			
1952	2146	870	81	1512			1585
1957	2853	2773	297	3816			2107
1962	1631	984	132	2205			542
1965	3346	1859	276	4451			1030
1970	3136	2835	129	2721			3379
1975	7489	8133	217	9378	1362		5099
1978	19968	9124	568	16828	1601		11231
1980	30993	15541	965	10371	12925	1420	22783
1985	64665	35100	7656	17377	35888	1136	53020
1990	167731	91305	25540	24687	91895	23688	144306
1991	196212	108929	43890	22615	131676	38325	156415
1992	304060	208384	70624	22660	213217	89130	258061
1993	600011	225555	127708	23736	208672	94469	626397
1994	842809	272126	214781	26062	318334	221265	764055
1995	1020484	385934	241863	25683	379198	328124	915276
1996	929735	458284	222543	35639	444443	310722	819758
1997	875057	485122	251723	27928	297089	363789	923096
1998	1034500	574852	300359	67762	491812	170709	1179428
1999	1456473	459596	289822	128603	584120	145589	1347579
2000	1476397	652640	297783	75978	489697	164175	1806238
2001	1781235	744549	408944	93491	620562	217526	2157787
2002	2328492	796685	558446	78775	719677	411704	2831288
2003	3544790	1133333	797403	127861	989178	722644	4237643
2004	6413046	2312139	1120461	85549	1100297	1016145	8245272
2005	8949231	3141732	1941997	136632	1250343	1850921	11540604
2006	8818614	3668191	2370089	391315	2126523	1662897	11856506
2007	9981666	4171878	2200092	215649	2427031	1788056	13109824
2008	11795435	5907195	2487468	322484	3309471	1951468	15855082
2009	14911874	5869214	3807801	778090	4217265	1593287	21555685
2010	18725922	6362880	5135983	1305737	5881886	2135736	27898294
2011	22865421	7521441	4638520	1640702	5871709	1766278	33579182
2012	26080239	8601282	6857625	1324159	5484459	1561597	37356847
2013	33237368	9969777	7071504	1959340	8154829	1528585	49552883
2014	39006113	11640126	7014069	1404667	7452983	1181541	55024776
2015	42893643	15972153	6690889	2067396	7249574	1098178	62458544
2016	45915827	16425993	8789790	1788103	14203650	903773	66069945
2017	55647641	10977494	11145767	2257927	14569701	641216	65055089

4-2 主要年份固定资产投资构成（以投资总额为 100）

COMPOSITION OF MAJOR YEAR'S INVESTMENT IN FIXED ASSETS（TOTAL INVESTMENT=100）

年份 Year	按用途分 Crouped by Use		
	生产性投资 Productive Investment	非生产性投资 Non-Productive Investment	#住宅投资占非生产性比重 Percentage of Investment in Residential Buildings to Non-Productive Investment
1949	75.00	25.00	75.00
1952	44.17	55.83	58.24
1957	66.64	33.36	58.25
1962	74.59	25.41	27.51
1965	75.81	24.19	28.28
1970	95.30	4.70	53.66
1975	84.97	15.03	44.69
1978	79.23	20.77	51.99
1980	71.30	28.70	68.47
1985	56.45	43.55	40.74
1990	73.95	26.05	52.50
1991	73.53	26.47	56.17
1992	72.16	27.84	51.65
1993	53.68	46.32	36.78
1994	54.79	45.21	53.91
1995	56.10	43.90	54.00
1996	60.40	39.60	44.70
1997	57.80	42.20	41.00
1998	59.00	41.00	16.60
1999	56.50	43.50	19.10
2000	54.00	46.00	19.40
2001	50.70	49.30	50.40
2002	53.30	46.70	44.50
2003	55.20	44.80	42.40
2004	64.40	35.60	45.80
2005	65.40	34.60	37.90
2006	62.00	38.00	39.40
2007	62.60	37.40	44.40
2008	61.60	38.40	42.80
2009	56.30	43.70	35.70
2010	53.62	46.38	40.02
2011	53.17	46.83	34.80
2012	58.37	41.63	38.68
2013	61.65	38.35	37.45
2014	57.08	42.92	29.54
2015	63.16	36.84	31.34
2016	59.81	40.19	31.92
2017	53.64	46.36	25.67

4-2 续表
Continued

年份 Year	按构成分 Gouped by Composition of Funds			按资金来源分 Grouped by Sources of Funds			
	建筑安装工程 Constrection and Installation	设备工器具购置 Purchase of Equipment and Instruments	其他费用 Others	国家投资 State Investment	国内贷款 Domestic Loans	利用外资 Foreign Investment	自筹及其他 Self-raising Fund and Others
1949	68.75	18.75	12.5	100			
1952	69.29	28.09	2.62	48.82			51.18
1957	48.17	46.82	5.01	64.43			35.57
1962	59.37	35.82	4.81	80.27			19.73
1965	61.05	33.92	5.03	81.21			18.79
1970	51.41	46.48	2.11	44.61			55.39
1975	47.28	51.35	1.37	59.21	8.60		32.19
1978	67.32	30.76	1.92	56.74	5.40		37.86
1980	65.25	32.72	2.03	21.83	27.21	2.99	47.97
1985	60.2	32.68	7.12	16.18	33.41	1.06	49.35
1990	58.94	32.08	8.98	8.68	32.29	8.32	50.71
1991	56.22	31.21	12.57	6.48	37.73	10.98	44.81
1992	52.15	35.74	12.11	3.89	36.56	15.29	44.26
1993	62.94	23.66	13.4	2.49	21.89	9.91	65.71
1994	63.38	20.46	16.16	1.96	23.94	16.64	57.46
1995	61.9	23.4	14.7	1.6	23.0	19.9	55.5
1996	57.7	28.5	13.8	2.2	27.6	19.3	50.9
1997	54.3	30.1	15.6	1.7	18.4	22.6	57.3
1998	54.2	20.8	15.7	3.5	25.8	8.9	61.8
1999	66.0	20.8	13.2	5.8	26.5	6.6	61.1
2000	60.8	26.9	12.3	3.1	20.2	6.8	74.4
2001	60.7	25.4	13.9	3.0	20.1	7.0	69.9
2002	63.2	21.6	15.2	1.9	17.8	10.2	70.1
2003	64.7	20.7	14.6	2.1	16.3	11.9	69.7
2004	65.1	23.5	11.4	0.8	10.6	9.7	78.9
2005	63.8	22.4	13.8	0.9	8.5	12.5	78.1
2006	59.4	24.7	15.9	2.4	13.3	10.4	73.9
2007	61.0	25.5	13.5	1.2	13.8	10.2	74.8
2008	58.4	29.3	12.3	1.5	15.4	9.1	74.0
2009	60.6	23.9	15.5	2.8	15.0	5.6	76.6
2010	62.0	21.0	17.0	3.5	15.8	5.7	75.0
2011	65.3	21.5	13.3	3.8	13.7	4.1	78.4
2012	62.8	20.7	16.5	2.9	12.0	3.4	81.7
2013	66.1	19.8	14.1	3.2	13.3	2.5	81.0
2014	67.6	20.2	12.2	2.2	11.5	1.8	84.6
2015	65.4	24.4	10.2	2.8	10.0	1.5	85.7
2016	61.6	22.0	11.8	2.2	17.1	1.1	79.6
2017	71.6	14.1	14.3	2.7	17.7	0.8	78.8
2018	68.8	7.8	23.4	2.9	15.2	0.4	81.5

4-3 按三次产业分规模以上固定资产投资增速（2018 年）
GROWTH RATE OF INVESTMENT IN FIXED ASSETS ABOVE DESIGNATED SIZE BY THREE STRATA OF INDUSTRY（2018）

单位：%（%）

指标	Indicator	2018 年
**总　计**	**Total**	**7.9**
第一产业	Primary Industry	14.3
第二产业	Secondary Industry	9.1
# 工业	Industry	7.4
第三产业	Tertiary Industry	7.5

4-4 分市、区固定资产投资额增速（2018 年）
GROWTH RATE OF INVESTMENT IN FIXED ASSETS BY REGION（2018）

单位：%（%）

市、区名称	Region	规模以上固定资产投资额 Investment in Fixed Assets above Designated Size	# 房地产开发 Investment in Real Estate Development
**全市**	**Whole Municipality**	**7.9**	**11.6**
市南区	Shinan District	16.0	-63.8
市北区	Shibei District	3.6	-13.6
李沧区	Licang District	15.8	-38.3
崂山区	Laoshan District	15.6	20.6
黄岛区	Huangdao District	13.1	2.7
城阳区	Chengyang District	3.5	49.5
即墨区	Jimo District	9.9	5.8
胶州市	Jiaozhou	9.9	160.3
平度市	Pingdu	3.1	40.7
莱西市	Laixi	3.0	17.9
红岛经济区	Qingdao National High-tech Industrial Development Zone	15.9	90.7
保税港区	Qingdao Free Trade Port Area of China	10.4	17.6

4-5　规模以上固定资产投资增速（2018 年）
INVESTMENT IN FIXED ASSETS ABOVE DESIGNATED SIZE（2018）

单位：%（%）

项目	Item	合计 Total	# 房地产开发 Investment in Real Estate Development
**一、本年施工项目（个）**	**Projects Under Consruction This Year（unit）**	**2434**	
# 本年新开工（个）	of which：Newly Started（unit）	1409	
**二、本年建成投产项目（个）**	Projects Completed and Put into Use This Year（unit）	1395	
**三、本年完成投资（%）**	**Investment Completed This Year（%）**	**7.9**	**11.6**
1. 按构成分	**Grouped by Composition**		
# 建筑安装工程	Construction installation project	25.1	-7.5
设备工器具购置	Purchase of Equipment and Instruments	-67.1	-18.4
2. 按建设性质分	Grouped by Type of Construction		
# 新建	New Construction	8.5	
扩建	Expansion	-39.9	
改建和技术改造	Reconstruction	112.7	
3. 按国民经济行业分	Grouped by Economic Sector		
（1）农、林、牧、渔业	Farming，Forestry，Animal Husbandry and Fishery	15.8	
农业	Farming	25.1	
林业	Forestry	146.6	
畜牧业	Animal Husbandry	-24.4	
渔业	Fishery	325.6	
农、林、牧、渔服务业	Farming，Forestry，Animal Husbandry and Fishery Services	38.6	
（2）采矿业	Mining	1123.7	
煤炭开采和洗选业	Mining and Washing of Coal		
石油和天然气开采业	Extraction of Petroleum and Natural Gas		
黑色金属矿采选业	Mining of Ferrous Metal Ores		
有色金属矿采选业	Mining of Non-ferrous Metal Ores	776.2	
非金属矿采选业	Mining and Processing of Nonmetal Ores		
开采辅助活动	Auxiliary Activities of Mining		
其他采矿业	Mining of Other Ores		

4-5 续表 1
Continued

单位：%（%）

项目	Item	合计 Total	# 房地产开发 Investment in Real Estate Development
（3）制造业	Manufacturing	9.9	
农副食品加工业	Processing of Food from Agricultural Products	-8.5	
食品制造业	Manufacture of Foods	13.2	
酒、饮料和精制茶制造业	Manufacture of Liquor，Beverage and Refind Tea	275.5	
烟草制品业	Manufacture of Tobacco		
纺织业	Manufacture of Textile	-13.1	
纺织服装、服饰业	Manufacture of Textile Wearing Apparel	-46.6	
皮革、毛皮、羽毛及其制品和制鞋业	Manufacture of Leather，Fur，Feather & Its Products Footwear	-44.1	
木材加工和木、竹、藤、棕、草制品业	Processing of Timbers，Manufacture of Wood，Bamboo，Rattan，Palm and Straw Products	133.8	
家具制造业	Manudacture of Furniture	84.8	
造纸和纸制品业	Manufacture of Paper and Paper Products	-3.9	
印刷和记录媒介复制业	Printing，Reproduction of Recording Media	-28.8	
文教、工美、体育和娱乐用品制造业	Manufacture of Articles for Culture，Arts & Crafts，Sports and Entertainment	6.2	
石油加工、炼焦和核燃料加工业	Processing of Petroleum，Coking，Processing of Nucleus Fuel	-96.0	
化学原料和化学制品制造业	Manufacture of Chemical Raw Material and Chemical Products	266.2	
医药制造业	Manufacture of Medicines	-25.3	
化学纤维制造业	Manufacture of Chemical Fiber	63.2	
橡胶和塑料制品业	Manufacture of Rubber and Plastic	-18.2	
非金属矿物制品业	Manufacture of Non-metallic Mineral Products	57.2	
黑色金属冶炼和压延加工业	Smelting and Pressing of Ferrous Metals	-86.1	
有色金属冶炼和压延加工业	Smelting and Pressing of Non-ferrous Metals	-18.6	
金属制品业	Manufacture of Metal Products	42.9	
通用设备制造业	Manufacture of General Purpose Machinery	38.1	
专用设备制造业	Manufacture of Special Purpose Machinery	-11.7	
汽车制造业	Manufacture of Vehicle	-14.5	
铁路、船舶、航空航天和其他运输设备制造业	Manufacture of Transport Equipement for Railway，Shipping，Aerospace and other uses	7.8	
电气机械和器材制造业	Manufacture of Electrical Machinery & Equipment	38.4	
计算机、通信和其他电子设备制造业	Namufacture of Computer，Communication Equipment and Other Electronic Equipment	30.7	
仪器仪表制造业	Manufacture of Measuring Instrument	-40.7	
其他制造业	Manufacture of Other Products	-77.4	
废弃资源综合利用业	Recycling and Disposal of Waste Resources	180.6	
金属制品、机械和设备修理业	Maintenance of Metal Products，Machinery and Equipment	238.4	

4-5 续表 2
Continued

单位：%（%）

项目	Item	合计 Total	# 房地产开发 Investment in Real Estate Development
（4）电力、燃气及水的生产和供应业	Production and Supply of Electricity Gas and Water	-27.0	
电力、热力的生产和供应业	Production and Supply of Electric Power and Heat Power	-34.3	
燃气生产和供应业	Production and Supply of Gas	-61.1	
水的生产和供应业	Production and Supply of Water	8.8	
（5）建筑业	Construction	474.1	
（6）交通运输、仓储及邮政业	Transport Storage and Post	-18.7	
铁路运输业	Railway Transport	-12.3	
道路运输业	Road Transport	-11.3	
水上运输业	Water Transport	-21.4	
航空运输业	Air Transport	-66.8	
管道运输业	Pipeline Transport	-13.7	
装卸搬运和运输代理业	Handing，Corrying and Transportation Agent	-74.8	
仓储业	Ware houses and Storage	90.4	
邮政业	Post		
（7）信息传输、软件和信息技术服务业	Information Transmission Computer Services and Software	-27.3	
（8）批发和零售业	Wholesale and Retail Trade	-62.3	
（9）住宿和餐饮业	Hotels and Catering Services	-21.7	
（10）金融业	Financial Intermediation	1241.4	
（11）房地产业	Financial Intermediation	-12.2	11.6

4-5 续表 3
Continued

单位：%（%）

项目	Item	合计 Total	# 房地产开发 Investment in Real Estate Development
（12）租赁和商务服务业	Leasing and Business Services	107.9	
（13）科学研究和技术服务业	Scientific Research，Technical Service and Geologic Prospecting	-5.8	
（14）水利、环境和公共设施管理业	Management of Water Conservancy，Environment and Pubic Facilities	105.0	
水利管理业	Management of Water Conservancy	39.7	
环境管理业	Environmental Management	223.0	
公共设施管理业	Management of Public Facilities	110.2	
（15）居民服务和其他服务业	Services to Households and Other Services	-29.8	
居民服务业	Serviced to Households	-48.9	
机动车、电子产品和日用产品修理业	Maintenance of Vehicle，Electronic Products and Daily Articles	-26.3	
其他服务业	Other Services	-17.0	
（16）教育	Educatilon	14.3	
（17）卫生和社会工作	Health and Social Work	-28.0	
卫生	Health	-27.1	
（18）文化、体育和娱乐业	Culture，Sports and Entertainment	-69.1	
新闻出版业	Journalism and Publishing Activities	134.8	
广播、电视、体育和音像业	Broadcasting，Movies，Televisions and Audiovisual Activities	-96.7	
文化艺术业	Cultural and Art Activities	-19.3	
体育	Sports Activities	-82.7	
娱乐业	Entertainment	-14.0	
（19）公共管理、社会保障和社会组织	Public Management，Social Security and Social Organzation	0.5	
中国共产党机关	Communist Party of China	-89.1	
国家机构	Govenment Agencies	1.9	
人民政协、民主党派	People’s Political Consultatioe Conference，Democratic Party		
社会保障	Social Security		
群众团体、社会团体和其他成员组织	Non-Governmental Institutions，Social Organizations and Other Organizations		
基层群众自治组织	Local People Self-government Organization	-0.9	
（20）国际组织	International Organization		

4-5 续表 4
Continued

单位：%（%）

项目	Item	合计 Total	# 房地产开发 Investment in Real Estate Development
**四、本年资金来源合计**	**Total Funds This Year**	**5.6**	**12.6**
上年末结余资金	Balance of Last Year	30.6	15.6
本年资金来源小计	Sub-total Funds This Year	1.6	11.5
国家预算内资金	State Budget	-16.6	
国内贷款	Domestic Loans	-19.2	-13.9
债券	State Treasury Bond		
利用外资	Foreign Investment	-41.6	-80.7
自筹资金	Self-raising Funds	1.8	-9.5
其他资金来源	Others	21.1	33.6

4-6 主要年份房地产开发投资
MAJOR YEAR'S INVESTMENT IN REAL ESTATE DEVELOPMENT

单位：万元、万平方米（10 000 yuan，10 000 sq.m）

年份 Year	房地产开发投资额 Investment in Real Estate Development	房屋施工面积 Floor Space Under Construction	房屋竣工面积 Floor Space Completed	房屋实际销售面积 Floor Space of Buildings Sold	房屋实际销售额 Sale of Buildings
1995	562861	1008	240	103	185384
1996	455765	748	203	80	125150
1997	426570	675	237	113	216311
1998	438059	742	259	178	351805
1999	629717	900	474	240	430123
2000	675142	1047	352	301	550401
2001	925153	1374	532	406	802364
2002	1036476	1417	537	427	933031
2003	1277969	1765	552	469	1121885
2004	1626965	2101	635	516	1530964
2005	2238370	2363	811	740	2666738
2006	2683631	2751	654	719	3054907
2007	3223547	3224	641	833	4333566
2008	3805652	3773	672	770	3924175
2009	4594829	4310	814	1262	7036744
2010	6024387	5058	1020	1360	8952942
2011	7827193	5693	925	1026	7702379
2012	9301099	6474	1212	951	7660684
2013	10485229	7073	957	1160	9786176
2014	11177297	8171	1136	1164	9708984
2015	11223458	8971	1523	1419	12628568
2016	13691425	9101	1426	1939	17899532
2017	13305432	9533	1487	1901	19992012
2018	14852089	10390	1625	1808	22824618

4-7 房地产开发投资情况（2018 年）
INVESTMENT IN REAL ESTATE DEVELOPMENT（2018）

单位：万元（10 000 yuan）

指标名称	Indicator	合计 Total	按经济类型分 Grouped by Economic Types		
			国有经济 State-owned	集体经济 Collective-owned	其他经济 Others
**本年完成投资额**	**Investment Completed This Year**	**14852089**	**705310**	**37535**	**14109244**
1. 住宅	Residential Buildings	10348412	418191	24493	9905728
2. 办公楼	Office Buildings	984710	94846		889864
3. 商业营业用房	Houses for Business Use	1536861	94621	824	1441416
4. 其他	Others	1982106	97652	12218	1872236

4-7 续表
Continued

单位：万元（10 000 yuan）

指标名称	Indicator	按资质等级分 Grouped by Qualification Criteria					
		一级 First Grade	二级 Second Grade	三级 Third Grade	四级 Fourth Grade	暂定 Provistonal	其他 Others
**本年完成投资额**	**Investment Completed This Year**	**351255**	**767378**	**338783**	**237751**	**10000875**	**3156047**
1. 住宅	Residential Buildings	136358	595640	260740	149377	7150803	2055494
2. 办公楼	Office Buildings	57923	3731	10507	19525	595499	297525
3. 商业营业用房	Houses for Business Use	73991	24776	48632	51378	1075779	262305
4. 其他	Others	82983	143231	18904	17471	1178794	540723

4-8 房地产施工、竣工面积及竣工价值（2018 年）
FLOOR SPACE UNDER CONSTRUCTION AND COMPLETED AND COMPLETED VALUE OF REAL ESTATE（2018）

单位：万平方米（10 000 sq.m）

施工、竣工房屋面积及竣工价值	Indicator	施工面积 Floor Space Under Construction	# 新开工 Newly Started	竣工房屋 Floor Space Completed	竣工房屋价值（万元）Completed Value（10 000 yuan）
**房屋建筑面积总计**	**Floor Space of Buildings**	**10390**	**2803**	**1625**	**4609219**
住宅	Residential Buildings	6832	1848	1079	2861438
办公楼	Office Buildings	670	131	126	432764
商业营业用房	Houses for Business Use	1046	272	170	560814
其他	Others	1842	552	250	754103

4-9 商品房屋销售情况（2018 年）
BASIC STATISTICS ON SALES OF COMMERCIALIZED BUILBINGS（2018）

单位：万平方米（10 000 sq.m）

商品房屋销售情况	Sales and Rental of Commercialized Buildings	实际销售 Actual Sales	实际销售额（万元）Actual Sales Volume（10 000 yuan）	待售面积 Area for Sale
**房屋面积总计**	**Floor Space of Buildings**	**1808**	**22824618**	**433**
住宅	Residential Buildings	1578	19529067	162
办公楼	Office Buildings	77	1123363	91
商业营业用房	Houses for Business Use	120	1781244	152
其他	Others	33	390944	28

主要统计指标解释

固定资产投资 是以货币形式表现的在一定时期内建造和购置固定资产的工作量以及与此有关的费用的总称。

房地产开发投资 指各种登记注册类型的房地产开发法人单位统一开发的住宅、厂房、仓库、饭店、宾馆、度假村、写字楼、办公楼等房屋建筑物，配套的服务设施，土地开发工程（如道路、给水、排水、供电、供热、通讯、平整场地等基础设施工程）和土地购置的投资；不包括单纯的土地开发和交易活动。

固定资产投资的资金来源 根据固定资产投资的资金来源不同，分为国家预算资金、国内贷款、利用外资、自筹资金和其他资金。

固定资产投资 按建设性质分建设项目的性质一般分为新建、扩建、改建和技术改造、单纯建造生活设施、迁建、恢复、单纯购置。

固定资产投资 按构成分固定资产投资活动按其工作内容和实现方式分为建筑安装工程，设备、工具、器具购置，其他费用三个部分。

新增生产能力（或工程效益） 指建成投产项目或工程新增生产能力（或工程效益）的名称。

房屋施工面积 是指报告期内施工的全部房屋建筑面积。包括本期新开工的面积和上期开工跨入本期继续施工的房屋面积，以及上期已停缓建在本期恢复施工的房屋面积。本期竣工和本期施工后又停缓建的房屋，其建筑面积仍计入本期房屋施工面积中。

房屋竣工面积 是指在报告期内房屋建筑按照设计要求全部完工，达到住人和使用条件，经验收鉴定合格（或达到竣工验收标准），正式移交使用单位的各栋房屋建筑面积的总和。

本年新增固定资产 指在报告期已经完成建造和购置过程，并已交付生产或使用单位的固定资产的价值，包括已经建成投入生产或交付使用的工程投资和达到固定资产标准的设备、工具、器具的投资及有关应摊入的费用。

Explanatory Notes on Main Statistical Indicators

Fixed investment is a generic term of quantity of work from building and acquiring of fixed assets over a period of time and any relevant expenses incurred thereby in the form of currency.

Real estate development investment means investments in all kinds of registered housing & building，including residences，plants，warehouses，restaurants，hotels，resorts，office buildings and administrative buildings which are developed uniformly by real estate development corporation，supporting sevice facilities and land development projects（such as road，water supply & drainage，power supply，heat supply，telecommunication and ground leveling and other infrastructure projects）and land acquisition；excludes simple land development and transaction activities.

Source of funds for fixed-asset investment can be divided into national budget funds，domestic loans，utilization of foreign capital，self-raised funds and other funds in accordance with the different sources.

Construction items of fixed-asset investment can be divided into new construction, extension, reconstruction, technical reform, simply-constructed living facilities, relocation, restoration and simple acquisition by property of construction.

Fixed-asset investment activities can be divided into three parts, namely construction and installation project, acquisition of\ equipement, tools and appliances and other expenses by working content and method of achieving.

New productive capacity (or project benefit) means the completed projects or new productive capacity (or projevt benefit) of projects.

Construction area neans total areas of all buildings which are under construction in the ruport period, including the area which is newly constructed in this period and the areas of buildings which commence in the last period and carry forward to this period for continuing construction. and the areas of buildings which stop constructing in the last period and resume to be constructed in this period. The areas of the buildings which are completed in this period and stop constructing after this period' s construction are still reckoned in floor space under construction in this period.

Completed ares means the wum total of areas of housings & buildings which are completed according to design requirements in the report period, reach conditions of living and using and are transferred to building users formally after checked and accepted (or reach the standards of completion and acceptance).

New fixed assets in this year means the value of fixed assets which are completed and acquired in the report period and delivered for production or use, including investment in constructions which are put into production or service, investment in equipment, tools and appliances which reach the standards of fixed asset, and the expenses which should be added into fixed assets.

对外经济贸易 5

FOREIGN TRADE

简 要 说 明

一、本篇资料的主要内容

本篇资料主要反映了全市外经外贸和外资企业的基本情况，主要包括外贸进出口、利用外资、对外投资与经济合作等方面的资料。

二、本篇资料的来源

1、口岸进出口数据来源于青岛市海关。

2、进出口、利用外资等资料、对外投资与经济合作来源于市商务局。

本篇资料由市统计局外经贸易统计处整理提供。

Brief Introduction

I. Main Content

Data in this chapter show the basic conditions of foreign trade and foreign-funded enterprises, mainly including imports and exports, utilization of foreign capitals, production and operation condition of foreign-funded enterprises, etc.

II. Source of Data

1.Data on imports and exports of Qingdao port are provided by Office of Qingdao Port Administration.

2.Data on imports & exports and utilization of foreign capitals are provided by Qingdao Municipal Commerce Bureau.

3.Data on production and operation condition of foreign-funded enterprises are based on the National Union Annual Inspection of foreign-funded enterprises.

Data in this chapter are prepared and compiled by the Division of Trade and External Economic Relations Statistics of Qingdao Municipal Bureau of Statistics.

5-1 青岛口岸进出口总额（1985-2018年）

TOTAL VALUE OF IMPORTS AND EXPORTS OF QINGDAO PORT（1985-2018）

单位：万美元（10 000 USD）、万元（10 000 yuan）

年份 Year	青岛口岸进出口总额 Total Value of Imports and Exports Oing dao Port	出口 Exports	进口 Imports
1985	414448	234652	179796
1986	382840	191926	190914
1987	445090	259458	185632
1988	474516	259176	215340
1989	506152	268397	237755
1990	477117	304011	173106
1991	519273	332949	186324
1992	581495	350163	231332
1993	678013	360297	317716
1994	888574	510760	377814
1995	1307508	705245	602263
1996	1391323	760566	629757
1997	1542284	936142	609142
1998	1463718	933392	530326
1999	1696514	1063768	632746
2000	2520420	1423266	1097154
2001	2797085	1636888	1160197
2002	3128048	1889612	1238436
2003	4088096	2366046	1722050
2004	5677570	3197697	2479873
2005	6932944	3959947	2972997
2006	8019198	4495731	3523467
2007	9257099	5263539	3993560
2008	11558079	5953501	5604578
2009	9049699	4694832	4354867
2010	11912702	5988254	5924448
2011	15095088	7294226	7800862
2012	14888716	7120813	7767903
2013	15662732	7696141	7966591
2014	16489966	8736358	7753608
2015	14448349	8776286	5672063
2016	94927180	56513317	38413863
2017	111024375	63298062	47726314
2018	121968146	67385279	54582867

注：2016年起，统计单位为人民币万元。
Note：Statistic unit is RMB ten thousand Yuan as from 2016.

5-2 进出口总额（1988-2018 年）
TOTAL VALUE OF IMPORTS AND EXPORTS（1988-2018）

单位：万美元（10 000 USD）、万元（10 000 yuan）

年份 Year	进出口总额（含中央、省驻青公司）Total Value of Imports and Exports（including central and provincial companies）	出口 Exports	进口 Imports	进出口总额（不含中央、省驻青公司）Total Value of Imports and Exports（including central and provincial companies）	出口 Exports	进口 Imports
1988				28097	21731	6366
1989				39327	27797	11530
1990				41649	33529	8120
1991				54624	44746	9878
1992				88600	66291	22309
1993	517643	346288	171355	139048	100201	38847
1994	633213	434102	199111	234978	164135	70843
1995	858560	536386	322174	376372	245072	131300
1996	863521	516317	347204	461950	287123	174827
1997	916269	579261	377008	521967	338535	183432
1998	882042	573020	309022	595826	382657	213169
1999	1009885	631072	378813	775565	446260	329305
2000	1353222	826891	526331	1083133	611426	471707
2001	1541564	950160	591404	1235781	712024	523757
2002	1692567	1057141	635426	1409598	850420	559178
2003	2065912	1239199	826713	1784556	1035528	749028
2004	2698781	1578167	1120614	2433188	1391171	1042017
2005	3302230	1942242	1359988	3045542	1758834	1286708
2006	3911543	2346552	1564991	3655737	2164541	1491196
2007	4572534	2831004	1741530	4360499	2677596	1682903
2008	5363659	3262476	2101183	5215886	3146246	2069640
2009	4485115	2729865	1755250	4398639	2692197	1706442
2010	5705963	3391560	2314403	5614928	3335141	2279787
2011	7215217	4061309	3153908	7126310	4005572	3120738
2012				7320781	4081968	3238813
2013				7791217	4198605	3592612
2014				7988833	4577696	3411137
2015				7022243	4535015	2487228
2016				43506659	28219059	15287600
2017				50334973	30318103	20016870
2018				53212551	31722209	21490342

注：1、2012 年起，中央、省驻青公司外贸统计划归青岛，取消含中央、省驻青公司统计口径；
2、2016 年起，统计单位为人民币万元。

Note：1. Since 2012，the statistical calibre including central and provincoal companies has been cancled.
2. Statistic unit is RMB ten thousand Yuan as from 2016.

5-3 分国别外贸出口总额
TOTAL VALUE OF EXPORTS BY COUNTRIES OR REGIONS

单位：万美元（10 000 USD）

国别（地区）	Country（Region）	2018 年	2018 年比 2017 年 ±%<br>2018/2017（±%）	占比 %
亚洲	Aioa	2083063	9.8	43.5
香港	Hong Kong	191991	9.2	4.0
日本	Japan	562514	6.6	11.7
韩国	Korea	444012	7.6	9.3
东盟	ASEAN	463450	24.0	9.7
非洲	Africa	192717	14.1	4.0
南非	South Africa	45146	12.9	0.9
欧洲	Europe	1019206	-3.3	21.3
欧盟	EU	881451	7.2	18.4
南美洲	South America	317302	7.9	6.6
北美洲	North America	1045878	13.7	21.8
美国	United States	930533	12.3	19.4
大洋洲	Oceanica	135684	10.8	2.8
澳大利亚	Australia	113291	11.8	2.4

5-4 外贸出口商品分类
EXPORT COMMODITIES BY CATEGORY

单位：万元（10 000 yuan）

项目	Item	2018 年	2018 年比 2017 ± %<br>2018/2017（± %）	占比 %
**合计**	**Total**	**31722209**	**4.7**	**100.0**
按企业性质划分	By Enterprises Nature			
国有企业	State-owned Enterprises	2794782	-25.3	8.8
外商投资企业	Foreign Funded Enterprises	10116044	2.0	31.9
其他企业	Other Enerprises	18811383	13.0	59.3
# 集体企业	#Collective-owned Enterprises	897020	-9.1	2.8
私营企业	Private Enterprises	17903042	14.4	56.5
按贸易方式划分	By Customs Regime			
一般贸易	Ordinary Trade	21953506	13.2	69.2
加工贸易	Processing Trade	8927337	-8.4	28.2
其他贸易	Others	841366	-28.1	2.6
按大类商品划分	By Category of Commodities			
纺织服装	Textile Garments	4820050	7.7	15.2
农产品	Agricultural Products	3872661	8.4	12.2
机电产品	Mechanical and Electrical Products	13522213	0.5	42.7
高新技术产品	High-tech Products	3160605	5.1	10.0

5-5 外贸进口商品分类
IMPORT COMMODITIES BY CATEGORY

单位：万元（10 000 yuan）

项目	Item	2018 年	2018 年比 2017 ± %<br>2018/2017（ ± %）	占比 %
**合计**	**Total**	**21490342**	**7.3**	**100.0**
按企业性质划分	By Enterprises Nature			
国有企业	State-owned Enterprises	4959960	7.5	23.1
外商投资企业	Foreign Funded Enterprises	6199653	12.0	28.8
其他企业	Other Enerprises	10330728	4.6	48.1
# 集体企业	#Collective-owned Enterprises	215374	21.8	1.0
私营企业	Private Enterprises	10043400	4.3	46.7
按贸易方式划分	By Customs Regime			
一般贸易	Ordinary Trade	11282609	3.8	52.5
加工贸易	Processing Trade	3205228	1.8	14.9
其他贸易	Others	7002505	16.4	32.6
按大类商品划分	By Category of Commodities			
纺织服装	Textile Garments	292168	-9.8	1.4
农产品	Agricultural Products	4104093	15.2	19.1
机电产品	Mechanical and Electrical Products	4345845	5.2	20.2
高新技术产品	High-tech Products	2091472	5.1	9.7

5-6 二十大出口商品出口情况
INFORMATION ON THE EXPORTATION OF TOP 20 PRODUCTS

单位：万美元（10 000 USD）

商品名称	Name	2018 年	2018 年比 2017 年 ±%<br>2018/2017（±%）	占比 %
**合计**	**Total**	**4176922**	**6.6**	**100.0**
机械设备	Machinery and Equipment	661917	-9.0	15.8
电器及电子类产品	Electric Appliance and Electronic Products	571322	4.6	13.7
服装	Garments	463888	7.9	11.1
计算机与通信技术	Computer and Communication Technology	377063	4.8	9.0
运输工具	Transport Tools	434924	21.6	10.4
纺织品	Textile Products	266174	15.4	6.4
金属制品	Metal Products	240227	10.5	5.8
轮胎	Tyre	156194	11.0	3.7
电话机	Telephone Sets	160903	23.0	3.9
水海产品	Aquatic and Seawater Products	131601	4.7	3.2
蔬菜	Vegetables	120818	4.3	2.9
家具及其零件	Furniture	103243	2.4	2.5
钢材	Rolled Steel	134897	35.1	3.2
鞋类	Shoes	97556	10.0	2.3
塑料制品	Plastic Articles	82790	10.7	2.0
箱包	Luggage and Bags	59857	-13.5	1.4
汽车零件	Parts of Motor Vehicles	72722	26.7	1.7
空调	Air Conditioner	40728	-13.6	1.0
游戏机	Game Console	30	3.8	
铁合金	Ferroalloy	71		

5-7 二十大进口商品进口情况
INFORMATION ON THE IMPORTATION OF TOP 20 PRODUCTS

单位：万美元（10 000 USD）

商品名称	Name	2018 年	2018 年比 2017 年 ±%<br>2018/2017（±%）	占比 %
**合计**	**Total**	**1902558**	**3.2**	**100.0**
铁矿砂	Machinery and Equipment	321376	–4.8	16.9
电器及电子产品	Electric Appliance and Electronic Products	240801	–4.6	12.7
机械设备	Machinery and Equipment	198619	15.2	10.4
粮食	Grain	137134	–10.9	7.2
合成橡胶	Synthetic Rubber	112878	–13.3	5.9
天然橡胶	Natural Rubber	79625	–37.8	4.2
仪器仪表	Instruments and Meters	136277	19.5	7.2
集成电路	Integrated Circuits	102051	–2.6	5.4
塑料原料	Plastic Raw Materials	121952	25.7	6.4
冻鱼	Frozen Fish	110891	21.9	5.8
液晶显示板	Liquid Crystal DIsplay	61774	17.7	3.2
棉花	Cotton	80907	62.8	4.3
纺织品	Textile Products	40863	–8.1	2.1
运输工具	Means of Conveyance	52784	25.7	2.8
钢材	Rolled Steel	41070	6.4	2.2
塑料制品	Plastic Articles	10644	0.9	0.6
铝锭及铝材	Aluminum Ingots and Aluminum Products	8621	–2.7	0.5
成品油	Refined Petroleum Products	37449	377.1	2.0
氧化铝	Alumina			
钻石	Diamonds	3960	24.0	0.2

5-8 利用外资情况（2000-2018年）
UTILIZATION OF FOREIGN CAPITAL（2000-2018）

项目	Item	单位	Unit	2000年	2005年
**批准企业（项目）个数**	**Number of Enterprises（Projects）Approved**	**个**	**unit**	**1132**	**2530**
一、外商直接投资	Foreign Direct Investments	个	unit	1128	2530
中外合资企业	Sino-foreign Joint Ventures	个	unit	303	274
中外合作企业	Sino-foreign Cooperative Enterprises	个	unit	57	18
外商独资企业	Foreign-owned Enterprises	个	unit	76	2236
其它	Others	个	unit	1	2
二、外商其他投资	Other Foreign Investments	个	unit	4	
**合同外资金额**	**Total Amount of Contracted Foreign Investment**	**万美元**	**10000 USD**	**269081**	**954486**
一、外商直接投资	Foreign Direct Investments	万美元	10000 USD	266221	954486
中外合资企业	Sino-foreign Joint Ventures	万美元	10000 USD	69889	112777
中外合作企业	Sino-foreign Cooperative Enterprises	万美元	10000 USD	26835	17588
外商独资企业	Foreign-owned Enterprises	万美元	10000 USD	169370	813485
其它	Others	万美元	10000 USD	127	10636
二、外商其他投资	Other Foreign Investments	万美元	10000 USD	2860	
**实际利用外资金额**	**Total Amount of Foreign Investment Actually Utilized**	**万美元**	**10000 USD**	**128171**	**365625**
一、外商直接投资	Foreign Direct Investments	万美元	10000 USD	126132	365625
中外合资企业	Sino-foreign Joint Ventures	万美元	10000 USD	39224	77636
中外合作企业	Sino-foreign Cooperative Enterprises	万美元	10000 USD	3886	5654
外商独资企业	Foreign-owned Enterprises	万美元	10000 USD	82992	280130
其它	Others	万美元	10000 USD	30	2205
二、外商其他投资	Other Foreign Investments	万美元	10000 USD	2039	

单位：万美元（10 000 USD）

2008 年	2009 年	2010 年	2011 年	2012 年	2013 年	2014 年	2015 年	2016 年	2017 年	2018 年
**640**	**647**	**731**	**707**	**553**	**645**	**619**	**763**	**680**	**650**	**956**
640	647	731	707	553	645	619	763	680	650	956
99	99	153	158	113	165	140	162	154	188	294
4	2	4	4	2	2	1	2		1	8
537	545	574	544	438	478	475	597	523	457	653
	1		1			3	2	3	4	1
**304505**	**271504**	**475723**	**528501**	**600231**	**758063**	**638637**	**827357**	**769101**	**909629**	**980231**
304505	271504	475723	528501	600231	758063	638637	827357	769101	909629	980231
81796	61734	124148	142535	102252	191172	126878	116013	207933	246280	195336
367	4531	9179	15173	122	4765	–849	1731	28	327	34333
221759	204187	341977	369425	490764	562208	506271	706172	554073	655377	756426
583	1052	419	1368	7093	–82	6337	3441	7067	7645	–5864
**264295**	**186397**	**284281**	**363350**	**460027**	**552227**	**608100**	**669062**	**700273**	**773500**	**869257**
264295	186397	284281	363350	460027	552227	608100	669062	700273	773500	869257
43810	46856	84920	100699	169469	159424	172180	135671	171051	195189	191040
2945	3971	3567	2759	6262	2355	128	1570	251	220	19032
217540	135026	194500	255763	275579	390210	435102	531544	520052	569461	658368
	544	1294	4129	8717	238	690	277	8918	8325	817

5-9 当年外商直接投资项目数和投资额（2018 年）

NUMBER OF PROJECTS AND TOTAL AMOUNT OF FOREIGN DIRECT INVESTMENT（2018）

单位：万美元（10 000 USD）

项目	Item	批准企业项目数（个）Number of Projects Approved（unit）	合同外资金额（万美元）Total Amount of Contracted Foreign Investment（10000 USD）	实际利用外资金额（万美元）Total Amount of Foreign Investment Aetually Utilized（10000 USD）
**外商直接投资**	**Foreign Direct Investments**			
**一、按投资方式分**	**Grouped by Investment Form**			
# 中外合资企业	Sino-foreign Joint Ventures	294	195336	191040
中外合作企业	Sino-foreign Cooperative Enterprises	8	34333	19032
外商独资企业	Foreign-owned Enterprises	653	756426	658368
**二、按主要行业分**	**Grouped by Sector**			
# 农、林、牧、渔业	Farming，Foresty，Animal Hus-bandry and Fishery	12	724	5499
制造业	Manufacturing	248	302982	318301
电力、燃气及水的生产和供应业	Production and Supply of Electricity，Gas and Water		3446	5385
建筑业	Construction	12	16405	13278
信息传输、计算机服务和软件业	Information transmission，computer services and software industry	15	21870	20393
批发和零售业	Wholesale and Retail Trades	307	83170	130478
房地产业	Real estate industry	39	147112	237697
租赁和商务服务业	Leasing and Business Services	75	80238	54226
科学研究、技术服务和地质勘查业	Scientific Research，Technical Service and Geological Survey	60	108114	23167
**三、按主要国别（地区）分**	**Grouped by Countries（Regions）**			
# 香港	Hong Kong	254	701315	529004
韩国	Korea	356	115816	103323
美国	United States	53	19396	20035
日本	Japan	36	7615	26664
德国	Germany	27	22266	26593
澳大利亚	Australia	13	9020	13028
新加坡	Singapore	22	6947	26142

5-10 对外投资与经济合作
OUTBOUND INVESTMENT AND INTERNATIONAL ECONOMIC COOPERATION

名称	Name	单位	Unit	2018 年	2018 年比 2017 年 ± %<br>2018/2017（ ± %）
对外投资项目数	Number of Outbound Investment Projects	个	unit	95	-39.1
对外投资中方投资额	The Amount of Investment by Chinese Sides in Outbound Investment	万美元	10000 USD	504818.28	-21.5
对外承包工程新签合同额	The Amount of New Contracts for Foreign Contracted Projects	万美元	10000 USD	493108	11.9
对外承包工程完成营业额	The Completed Turnover of Foreign Contracted Projects	万美元	10000 USD	431244	12.8
对外劳务合作	Number of Persons Sent Overseas for International Labor Srevice Cooperation	人	Person	11820	-35.4

5-11 对外投资分国别（地区）情况表（2018 年）
INFORMATION ON OUTBOUND INVESTMENT BY COUNTRY/REGION FOR（2018）

国别（地区）	Country（Region）	项目数量（个）<br>Number of Projects（unit）	2018 年比 2017 年 ± %<br>2018/2017（ ± %）	中方投资额（万美元）<br>Amount of Investment by Chinese Sides（10000 USD）	2018 年比 2017 年 ± %<br>2018/2017（ ± %）
**总计**	**Total**	95	-39.1	504818	-21.5
亚洲	Asia	48	-44.8	201888	-8.8
韩国	Korea	13		71562	382.3
中国香港	Hong Kong，China	12	-52	117693	497
越南	Victnam	8	-38.5	4852	-74.93
乌兹别克斯坦	Uzbekistan	3		145	
泰国	Thailand	2		1011	-85.1
柬埔寨	Cambodia	2	-50	1160	-91.2
巴基斯坦	Pakistan	1		20000	16428.9
老挝	Laos	1		40	-99.7
马来西亚	Malaysia	1	-80	-7250	-109.8
日本	Japan	1	-88.9	-6379	-151.6
缅甸	Myanmar	1		950	90

5-11 对外投资分国别（地区）情况表（2018 年）

INFORMATION ON OUTBOUND INVESTMENT BY COUNTRY/REGION FOR（2018）

国别（地区）	Country（Region）	项目数量（个）<br>Number of Projects<br>（unit）	2018 年比 2017 年<br>± %<br>2018/2017（ ± %）	中方投资额（万美元）<br>Amount of Investment<br>by Chinese Sides<br>（10000 USD）	2018 年比 2017 年<br>± %<br>2018/2017（ ± %）
哈萨克斯坦	Kazakhstan	1		26	
新加坡	Singapore	1	-83.3	896	-83.4
印度	India	1	-50	30	-99.2
欧洲	Europe	7	-30	63603	313.9
英国	U.K.	2	-33.3	150	-97.7
荷兰	Holland	2		1512	7460.4
德国	Germany	1	-80	12	-99.6
葡萄牙	Portugal	1		351	
意大利	Italy	1		61139	
俄罗斯联邦	Russian	增资	-100	440	-92.9
拉丁美洲	Latin America	6		13465	-95.5
英属维尔京群岛	British Virgin Islands	2		12750	99.1
墨西哥	Mexico	2		540	
阿根廷	Argentina	1		23	
玻利维亚	Bolivia	1		3	
巴拿马	Panama	增资		150	
北美洲	North America	18	-51.3	202549	176.1
美国	United States	14	-57.6	92335	106.4
加拿大	Canada	4		100378	250.6
百慕大群岛	Bermuda	增资		9837	
大洋洲	Oceanica	7	16.7	29455	1478.7
澳大利亚	Australia	7	40	28911	2087.3
新西兰	New Zealand	增资	-100	544	

主要统计指标解释

批准企业（项目）个数　是指外商直接投资中批准设立的外商投资企业个数、批准的合作开发项目个数。

合同外资金额是指批准外商投资企业的合同、章程中规定的外国投资者认缴的出资额和企业投资总额内的应由外方投资者以自己的境外自有资金直接向企业提供的贷款。包括新批准企业合同外资和原有企业的增资减资，增资减资不对企业（项目）个数进行调整。

实际使用外资金额　是指合同外资金额的实际执行金额。

外商直接投资是指外国企业和经济组织或个人（包括华侨、港澳台胞以及我国在境外注册的企业）按我国有关政策、法规，用现汇、实物、技术等在我国境内开办外商独资企业、与我国境内的企业或经济组织共同举办中外合资经营企业、合作经营企业或合作开发资源的投资（包括外商投资收益的再投资）以及经政府有关部门批准的项目投资总额内，企业从境外借入的资金。

外商其它投资　是指除外商直接投资以外其他方式吸收的外资。

进出口总额　指实际进出我国国境的货物总金额。包括对外贸易实际进出口货物，来料加工装配进出口货物，国家间、联合国及国际组织无偿援助物资和赠送品，华侨、港澳台同胞和外籍华人捐赠品，租赁期满归承租人所有的租赁货物，进料加工进出口货物，边境地方贸易及边境地区小额贸易进出口货物（边民互市贸易除外），中外合资企业、中外合作经营企业、外商独资经营企业进出口货物和公用物品，到、离岸价格在规定限额以上的进出口货样和广告品（无商业价值、无使用价值和免费提供出口的除外），从保税仓库提取在中国境内销售的进口货物，以及其他进出口货物。

Explanatory Notes on Main Statistical Indicators

Enterprises（Projects）Permitted　refers to number of foreign invested enterprises and developed projects under cooperation through the permission.

Contracted Foreign Investment　refers to expenditure promised by the foreign investor and loan stemed from broad innate fund of the foreign investor，according to the contract and regulation approved of enterprise invested by foreigner.

Actual Utilization of Foreign Investmentrefers to actual usage of contracted foreign investment，including cash，investment in kind and incorporeal agreed by the both sides as part of the investment，such as services and technology.

Foreign Direct Investmen　trefers to the investments by foreign enterprises and economic organizations or individuals（including overseas Chinese，compatriots from Hong dong，Macao and Taiwan，and Chinese enterprises registered abroad），following the relevant policies and laws of China，for the establishment of ventures and cooperative enterprises or cooperative exploration of resources with enterprises or economic organizations in China，lt includes the reinvestment of the foreign entrepreneurs with the profits gained from the investment and the funds that enterprises borrow from aboard in the total investment of projects which are approved by the relevant department of the government.

Other Investment by Foreign Entrepreneurs　refers to all forms of utilization of foreign capitals other than foreign direct investment.

Total Imports and Exports refers to the real value of commodities imported into and exported from the boundary of China. They include the actual imports and exports through foreign trade, imported and exported goods under the processing and assembling trades and materials, supplies and gifts as aid given gratis between governments and by the United Nations and other international organizations, and contributions donated by overseas Chinese, compatriots in Hong Kong and Macao and Chinese with foreign citizenship, leasing commodities owned by tenant at the expiration of leasing period, the imported and exported commodities processed with imported materials, commodities trading in border areas (excluding mutual exchange goods), the imported and exported commodities and articles for public use of the Sino-foreign joint ventures, cooperative enterprises and ventures exclusively with foreign own investment. Also included are import or export of samples and advertising goods for whose CIF or FOB value are beyond the permitted ceiling (excluding goods of no trading or use value and free commodities for export), imported goods sold in China from bonded warehouses and other imported or exported goods.

城市建设、环境保护 6

COTY CONSTRUCTION AND
ENVIRONMENT PROTECTION

简 要 说 明

一、本篇资料的主要内容

本篇资料主要反映了全市城市基础设施基本情况，包括市政设施、供水、供电、公共交通、园林绿化、燃气供热、城市环卫、环境保护及工业“三废”排放情况等方面的资料。

二、本篇资料的来源

1、本篇资料中市政设施、供水、公共交通、园林绿化、燃气供热、城市环卫相关资料来源于市建设委员会的城市建设统计年报，由市统计局固定资产投资统计处整理提供。

2、本篇资料中供电资料来源于青岛供电公司，由市统计局能源统计处整理提供。

3、本篇资料中环境保护、环境质量及工业“三废”排放情况来源于市环境保护局，由市统计局能源统计处整理提供。

Brief Introduction

I. Main Content

Data in this chapter show the basic conditions of public facilities of the whole city, including urban construction and infrastructure, water supply, electricity supply, public communications, urban greenery, gas and heating, urban sanitation, environmental protection and discharge conditions of industrial waste water, waste gas and solid waste, etc.

II. Source of Data

(1) Data on conditions of urban construction and infrastructure, water supply, public communications, urban greenery, gas and heating, urban sanitation are based on the annual report of city construction provided by Qingdao Municipal Construction Commission, and compiled by the Division of Investment and Construction Statistics of Qingdao Municipal Bureau of Statistics.

(2) Data on electricity supply are provided by Qingdao Power Corporation, and compiled by the Division of Energy Statistics of Qingdao Municipal Bureau of Statistics.

(3) Data on environmental protection, environmental conditions and discharge conditions of industrial waste water, waste gas and solid waste are provided by Qingdao Municipal Bureau of Environmental Protection, and compiled by the Division of Energy Statistics of Qingdao Municipal Bureau of Statistics.

6-1 主要年份城市建设和公用事业
MAJOR YEAR'S CITY CONSTRUCTION AND PUBLIC UTILITIES

年份<br>Year	建成区面积<br>（平方公里）<br>Developed Areas<br>（sq.km）	年末道路长度<br>（公里）<br>Length of Roads at Year-end（km）	年末道路面积<br>（万平方米）<br>Area of Roads at Year-end（10 000 sq.m）	公共汽车、电车线路网长度<br>（公里）<br>Network Length of Bus and Trolley Bus（km）
1949	27	243	206	26.4
1952	29	245	213	124.4
1957	36	248	217	232.3
1962	55	266	235	252.8
1965	57	266	235	308.1
1970	59	374	346	358.5
1975	63	392	355	389.3
1978	66	408	368	422.1
1980	72	408	377	474.1
1985	79	462	464	611.3
1990	94.3	667	786	777
1991	94.7	673	826	922
1992	95.4	683	840	988
1993	99.9	764	944	1094
1994	103.9	811	985	1372
1995	106	936	1177	1101
1996	110	991	1339	1211
1997	112	1047	1436	1492
1998	114	1106	1436	1859
1999	116	1171	1698	2339
2000	119.1	1192	1789	2711
2001	123	1248	2169	2521
2002	133	1426	2594	896
2003	145.9	1595	2816	973
2004	154.8	1755	3254	1185
2005	178.8	1862	3547	1159
2006	227.5	3160	5218	1362
2007	250.7	3288	5411	1480
2008	267.1	3318	5596	1470
2009	272.9	3402	5763	1216
2010	282.3	3409	5893	1383
2011	291.5	3705	6605	1719
2012	374.6	4281	7528	1978
2013	469.6	4334	7859	2002
2014	490.7	4393	7908	2023
2015	566.4	4375	7940	2106
2016	599.3	4484	8057	2116
2017	638.4	4865	8496	2220
2018	715.1	5770	9862	2400

6-1 续表 1
Continued

年份<br>Year	自来水供水管道长度（公里）<br>Length of Tap Water Pipelines（km）	全年供水总量（万立方米）<br>Annual Volume of Tap Water Supply（10 000 cu.m）	#全年售水量（万立方米）<br>Annual Volume of Tap Water Sale（10 000 cu.m）	#居民家庭用水<br>of which：Consumption for Residential Use	用水普及率（%）<br>Coverage Rate of Population with Access to Tap Water（%）	排水管道长度（公里）<br>Length of Sewage Pipes（km）	全年用电量（亿千瓦时）<br>Annual Volume of Electricity Supply（100 million kW·h）	#居民生活用电<br>of which：Consumption for Residential Use
1949	308	721	456	331	89	200	0.88	0.14
1952	341	778	612	310	90	227	1.85	0.14
1957	412	1392	1296	606	93	273	2.61	0.26
1962	483	2700	2512	937	98	290	3.77	0.57
1965	500	3220	2979	929	98	295	5.92	0.68
1970	521	4503	4106	1085	99	350	9.83	1.02
1975	564	6726	6302	1950	99	361	12.41	1.43
1978	572	5334	4975	1314	99	382	16.34	1.62
1980	588	8224	7874	2577	99	394	21.48	2.78
1985	685	7557	7038	2628	99.9	505	26.39	2.87
1990	873	11896	10211	4270	96.9	645	41.08	4.32
1991	908	13865	11760	4990	96.6	649	44.51	5.34
1992	1035	16385	13070	5931	96.5	672	51.78	6.84
1993	1123	16137	14027	6124	99.1	734	55.02	7.66
1994	1124	17367	14716	6405	100	811	60.74	8.78
1995	1046	22513	15810	7050	100	990	67.23	10.16
1996	1182	23277	16566	7731	100	1051	72.87	12.23
1997	1266	24130	17215	8787	100	1167	77.82	13.73
1998	1390	23845	17271	9115	100	1210	79.65	14.28
1999	1471	23849	18558	10032	100	1301	88.12	15.31
2000	1524	25414	20642	11405	100	1460	106.80	16
2001	1665	22225	18556	10555	100	1539	116.10	17.62
2002	1781	27662	21684	10129	100	1641	131.44	18.31
2003	2060	27594	21483	10142	100	1884	147.68	19.88
2004	2566	30877	23864	11695	100	2002	167.95	23.63
2005	2876	33265	25874	12536	100	2309	193.81	33.76
2006	3984	30524	25572	10682	100	3994	215.35	34.87
2007	4187	31533	26454	11408	100	4079	236.42	37.40
2008	4642	32675	27396	11301	100	4229	250.07	40.31
2009	4767	33490	28274	12250	100	4555	259.42	43.29
2010	4926	34909	29617	12524	100	4708	292.97	49.50
2011	5121	34109	28837	10049	100	5187	313.44	50.13
2012	5243	38945	33039	12121	100	6814	318.36	51.64
2013	5522	38331	32418	11851	100	6536	339.27	57.52
2014	6177	46649	40612	14834	100	6840	337.82	59.95
2015	6142	46203	40202	15381	100	6993	342.29	64.71
2016	6132	43033	36748	14430	100	7146	367.26	69.52
2017	6498	45991	38963	17439	100	7367	401.06	74.84
2018	7531	53235	43298	18473	100	8854	431.82	77.39

6-1 续表 2
Continued

年份 Year	公共汽车、电车营运车辆（辆）Number of Bus and Trolley Bus under Operation（unit）	全年客运量（万人次）Annual Passenger Traffic（10 000 person-times）	出租汽车（辆）Number of Taxi（unit）	使用液化气、煤气、天然气人数（万人）Population with Access to Gas（10 000 persons）				液化气供气量（吨）Volume of Liquefied Petroleum Supply（ton）
					液化气 Liquefied Petroleum	煤气 Coal Gas	天然气 Natural Gas	
1949	32	298						
1952	76	932						
1957	112	4110						
1962	127	4747						
1965	173	6092						
1970	224	13002						
1975	324	14426		2	2			184.9
1978	409	27351		17	17			3246
1980	485	38981	48	24	24			4345
1985	621	48852	219	42.1	42.1			10619
1990	801	63495	832	75.0	57.4	17.6		17512
1991	1372	67552	1070	76.2	57.4	18.8		17963
1992	1470	73529	1828	82.8	62.0	20.8		19565
1993	1875	72427	4874	88.4	64.6	23.8		22224
1994	1850	78231	5806	103.6	77.4	26.2		24684
1995	1891	71867	5887	116.2	82.9	33.3		32443
1996	2012	49077	6660	126.0	88.0	38.0		4065
1997	2148	55642	6861	133.6	92.9	40.7		38717
1998	2184	51042	7469	146.8	97.8	49.0		45381
1999	2470	55414	7839	154.9	98.3	56.6		40537
2000	3141	59879	7933	164.1	93.3	70.8		46951
2001	3453	60167	8110	168.8	87.5	81.3		45295
2002	3681	61713	8376	224.2	133.8	90.4		60123
2003	3648	58792	8109	246.7	138.3	93.1	15.3	67803
2004	3848	66463	8144	258.4	140.6	78.8	39.0	58548
2005	4039	68776	8121	265.0	132.6	45.7	86.7	78000
2006	4167	73726	8146	271.0	112.5	49.5	109.0	97343
2007	4524	78702	8221	275.6	104.1	12.1	159.4	79715
2008	4701	82460	9241	276.3	91.9	10.5	173.9	81987
2009	4288	81768	9316	276.1	59.8	12.3	204.0	96721
2010	4664	85251	9539	276.3	36.7	12.6	227.0	80073
2011	5419	89614	9683	277.1	25.8	12.6	238.7	79812
2012	5640	97922	9693	313.7	36.7	13.1	263.9	54341
2013	6179	101108	9826	318.9	33.0	0.0	285.9	41302
2014	6515	105592	9720	325.4	33.0	0.0	292.4	39255
2015	6748	102402	10033	338.3	28.9	0.0	309.4	35255
2016	7210	99596	10048	441.6	29.0	0.0	412.6	32291
2017	7420	104898	10875	445.8	26.2	0.0	419.6	28309
2018	7795	104782	10867	512.7	29.9	0.0	482.8	30427

注：1990 年以前公共营运车辆不包括系统外及个体。
Note：Vehicles not belonging to system and individual vehicles are not contained in public operating vehicles before 1990.

6-1　续表 3
Continued

年份 Year	煤气供气量（万立方米）Volume of Coal Gas Supply（10 000 cu.m）	天然气供气量（万立方米）Volume of Natural Gas Supply（10 000 cu.m）	燃气普及率(%) Coverage Rate of Population with Access to Gas（%）	绿地面积（公顷）Area of Green Areas（Hectate）	公园绿地面积（公顷）Park Green Areas（hectare）	人均公园绿地面积(平方米) Per Capita Park Green Areas（sq.m）	建成区绿化覆盖率(%) Green Coverage Rate of Developed Areas（%）	公园(个) Number of Parks and Zoos（unit）	公园面积（公顷）Area of Parks and zoos（hectare）
1949				133	42	0.7	4.9	3	43
1952				134	43	0.7	4.4	4	45
1957				245	132	1.7	6.9	10	122
1962				771	184	2.2	14.0	12	174
1965				777	184	2.1	13.7	12	174
1970				772	184	2.1	13.0	6	117
1975			2.3	465	162	1.8	7.6	5	134
1978			18.6	469	162	1.7	7.6	6	165
1980			24.5	625	206	2.1	10.4	6	152
1985			36.3	1072	255	2.3	17.3	7	153
1990	3073		56.2	2120	492	3.7	22.8	26	404
1991	3439		56.5	2318	500	3.7	24.4	28	443
1992	3824		60.0	2471	515	3.7	25.9	28	417
1993	4215		63.1	2488	543	3.9	26.5	31	453
1994	4116		63.1	2866	572	4.0	27.9	31	463
1995	4734		79.2	4547	771	5.3	30.1	33	675
1996	5447		83.9	4679	836	5.6	30.4	33	675
1997	5920		87.3	4717	924	6.0	31.3	34	730
1998	5959		92.5	6812	1047	6.6	35.0	35	775
1999	7319		96.0	7007	1186	7.4	35.9	36	791
2000	9567		98.0	7439	1423	8.5	37.0	37	790
2001	11814		99.0	7688	1588	9.3	37.5	39	832
2002	13500		99.5	7967	1791	8.1	36.4	43	926
2003	14826	1181	100	8829	2305	9.3	37.5	45	1034
2004	15503	2710	100	10047	2842	11.0	38.0	48	1421
2005	16573	7456	100	11137	3132	11.8	38.8	47	1110
2006	9821	13876	100	11756	3198	11.8	39.2	47	1188
2007	9516	18103	100	15369	3661	13.3	37.8	73	1268
2008	8691	21780	100	15630	4014	14.5	41.5	77	1815
2009	7523	26948	100	16003	4003	14.5	43.4	71	1897
2010	9153	35681	100	16619	4027	14.6	43.38	72	1917
2011	9137	46147	100	18013	4041	14.6	44.69	74	1931
2012	8148	68397	100	21471	4573	14.6	44.7	78	2112
2013	0	70918	100	28007	4649	14.6	44.7	87	2698
2014	0	74823	100	28805	4741	14.6	44.7	91	2988
2015	0	70311	100	29117	4802	14.6	44.7	87	3163
2016	0	70586	100	34851	8194	18.6	38.6	151	4460
2017	0	91572	100	36209	7763	17.4	39.1	175	4727
2018	0	120089	100	39229	8587	16	39	191	5767

注：1. 自 2002 年起人均公共绿地面积按辖区内全部人口计算。
2. 2005 年以前所用园林绿地面积；公共绿地面积；人均公共绿地面积；公园、动物园个数；公园、动物园面积指标分别改为现在的绿地面积；公共绿地面积；人均公园绿地面积；公园个数；公园面积。
3. 由于城阳区绿化指标进行了调整，部分绿地属性发生变化。导致 2017 年公园绿地面积及人均公园绿地面积的数据小于 2016 年。

Note：1. Per capita public green areas is calculated at total population in the area under jurisdiction since 2002.
2. Before 2005，the corresponding indicators of greenbelt atea，public greenbelt area，public greenbelt area，ber capita public greenbelt area，coverage rate of green，number of parks and zoos，area of parks and zoos.
3. As the green index of Chengyang District has been adjusted, some green space properties have changed. As a result, the data of park green area and per capita park green area in 2017 are smaller than 2016.

6-2 全年供电（2018 年）
ANNUAL ELECTRICITY SUPPLY（2018）

全年供电项目	Item	单位	Unit	2018 年
发电设备总容量	Total Capacity of Generation Equipment	万千瓦	10 000 kW	376.30
#青岛电厂	Qingdao Power Plant	万千瓦	10 000 kW	127.00
黄岛电厂	Huangdao Power Plant	万千瓦	10 000 kW	156.50
全年发电量	Annual Electricity Generation	亿千瓦时	100 million kw·h	181.70
#青岛电厂	Qingdao Power Plant	亿千瓦时	100 million kw·h	65.03
黄岛电厂	Huangdao Power Plant	亿千瓦时	100 million kw·h	81.24
全年实际用电量	Annual Electricity Consumption	亿千瓦时	100 million kw·h	431.82
#工业	Industrial Consumption	亿千瓦时	100 million kw·h	237.79
农业	Agricultural Consumption	亿千瓦时	100 million kw·h	8.32
生活	Residential Consumption	亿千瓦时	100 million kw·h	77.39
平均每日用电量	Average Daily Consumption	万千瓦时	100 million kw·h	11831

6-3 分行业用电（2018 年）
ELECTRICTTY CONSUMPTION BY SECTOR（2018）

单位：万千瓦时（10 000 kW · h）

行业	Sector	2018 年	2018 年比 2017 年增长（%）Growth Rate in 2018 over 2017（%）
**全社会用电总计**	**Total Electricity Consumption**	**4318172**	**7.67**
一、农、林、牧、渔业	Tarming，Forestry，Animal Husbandryand Fishery	83162	0.84
二、工业	Industry	2377894	5.39
三、建筑业	Construction	87095	9.82
四、交通运输、仓储和邮政业	Transport，Storage and Post	161244	15.71
五、信息传输、计算机服务和软件业	Information Transmission，Computer Services and Software	60337	11.44
六、商业、住宿和餐饮业	Trade，Hotels and Catering Services	234794	8.33
七、金融、房地产、商务及居民服务业	Financial Intermediation，Real Estate and Business Services	223995	31.02
八、公共事业及管理组织	Public Management and Social Organization	315778	24.28
九、城乡居民生活用电	Household Consumption	773874	2.21
城镇居民	Urban Area	484712	-1.65
乡村居民	Rural Area	289161	9.40

6-4 城市供水
ANNUAL ELECTRICITY SUPPLY

项目	Item	单位	Unit	2018 年
自来水供水管道长度	Length of Tap Water Pipelines	公里	km	7462.96
综合生产能力	Synthesis Producton Capacity	万立方米 / 日	10 000 cu.m/day	217.29
全年供水总量	Annual Volume of Tap Water Supply	万立方米	10 000 cu.m	50113.64
# 售水量	of which：Volume of Tap Water Supply	万立方米	10 000 cu.m	43297.84
# 生产运营用水	of which：Consumption for Production Use	万立方米	10 000 cu.m	15544.6
公共服务用水	Consumption for Public Services Use	万立方米	10 000 cu.m	9123.89
居民家庭用水	Consumption for Residential Use	万立方米	10 000 cu.m	18472.66
其他用水	Consumption for Other Uses	万立方米	10 000 cu.m	156.69
平均每日供水量	Daily Volume of Tap Water Supply	万立方米	10 000 cu.m	137.3
# 生产用	of which：Consumption for Production Use	万立方米	10 000 cu.m	42.6
生活用	Consumption for Residential Use	万立方米	10 000 cu.m	50.6
用水户数	Households with Access to Water	户	household	1811462
用水人口	Population with Access to water	万人	10 000 persons	510.82
工业用水量重复利用率	Recycle Rate of water for Industrial Use	%	%	89.99

6-5 城市公共交通
URBAN PUBLIC TRAFFIC

项目	Itme	单位	Unit	2018 年
公共汽车、电车线路网长度	Network Length of Bus and Trolley Bus	公里	km	2400
公共汽车、电车营运车辆	Number of Bus and Trolley Bus under Operation	辆	unit	7795
# 汽车	Bus	辆	unit	5192
电车	Trolley Bus	辆	unit	2603
公共汽车、电车全年客运量	Annual Passenger Traffic of Bus and Trolley Bus	万人次	10 000 person-times	104782
公共汽车、电车平均每日客运量	Daily Passenger Traffic of Bus and Trolley Bus	万人	10 000 persons	287
出租汽车数	Number of Taxi	辆	unit	10867
轮渡运营船数	Number of Ferry Boat under Operation	艘	ship	3
轮渡客运总量	Passenger Traffic of Ferry Boat	万人次	10 000 person-times	19.2
轮渡平均每日客运量	Daily Passenger Traffic of Ferry Boat	万人	10 000 persons	0.05

6-6 城市供气
URBAN GAS SUPPLY

项目	Itme	单位	Unit	2018 年
煤气供应总量	Volume of Coal Gas Supply	万立方米	10 000 cu.m	0
#家庭用量	Household Consumption	万立方米	10 000 cu.m	0
煤气用气户数	Households with Access to Coal Gas	户	household	0
#家庭用户	Household Users	户	household	0
液化石油气供应总量	Volume of Liquefied Petroleum Supply	吨	ton	30421
#家庭用量	Household Consumption	吨	ton	17199
液化石油气用气户数	Households with Access to Liquefied Petroleum	户	household	129217
#家庭用户	Household Users	户	household	125783
天然气供应总量	Volume of Natural Gas Supply	万立方米	10 000 cu.m	120089
#家庭用量	Household Consumption	万立方米	10 000 cu.m	28343
天然气用气户数	Households with Access to Natural Gas	户	household	1974645
#家庭用户	Household Users	户	household	1961323
燃气普及率	Coverage Rate of Population with Access to Gas	%	%	100

6-7 城市环境卫生
URBAN ENVIRONMENTAL SANITATION

项目	Itme	单位	Unit	2018 年
市容环卫专用车辆总数	Number of Special Vehicles for Environmental Sanitation	辆	unit	6194
生活垃圾清运量	Volume of Garbage Disposal	万吨	10 000 ton	268
生活垃圾无害化处理厂（场）数	Number of Bio-safety Disposal Plant of Garbage	座	unit	5
生活垃圾无害化处理能力	Bio-safety Disposal Capacity of Garbage	吨 / 日	ton/dan	6800
生活垃圾无害化处理量	Bio-safety Disposal Volume of Garbage	万吨	10 000 ton	268
道路清扫保洁面积	Area under Cleaning Program	万平方米	10 000 sq.m	8146.7
# 机械化	of which：Mechanization	万平方米	10 000 sq.m	4525
公共厕所	Public Toilets	个	unit	849

6-8 城市道路、下水道及绿化
URBAN ROAD，SEWAGE AND GREEN

项目	Itme	单位	Unit	2018 年
**道路**	**Road**			
年末道路长度	Length of Roads at Year-end	公里	km	5770
年末道路面积	Area of Roads at Year-end	万平方米	10 000 sq.m	9862
#人行道面积	of which： Area of Pavements	万平方米	10 000 sq.m	2230
排水管道长度	Length of Sewage Pipes	公里	km	8854
**园林绿化**	**Greening**			
绿化覆盖面积	Area of Green Coverage Areas	公顷	hectare	41914.27
绿地面积	Area of Green Areas	公顷	hectare	39228.68
公园绿地面积	Area of Park Green Areas	公顷	hectare	8587.16
公园数	Number of Parks and Zoos	个	unit	191
公园面积	Area of Parks and Zoos	公顷	hectare	5766.75
城市每人平均公园绿地面积	Per Capita Park Green Area	平方米	sq.m	16.3
建成区绿化覆盖率	Green Coverage Rate of Developed Areas	%	%	39.2

6-9 环境保护基本情况
BASIC CONDITIONS OF ENVIRONMENTAL PROTECTION

项目	Itme	单位	Unit	2018 年	2017 年	2018 年比 2017 年增（+）减（-）% 2018 Compared to 2017（+/-）
二氧化硫排放总量	Sulphur Dioxide Emission	吨	ton	14631.39	15540.54	-5.85
氮氧化物排放总量	Discharge Amount of Nitrogenoxides	吨	ton	16095.77	16673.86	-3.47
烟（粉）尘排放总量	Soot（Dust）Emission	吨	ton	12773.77	15404.52	-17.08
工业固体废物排放总量	Industrial Solid Wastes Discharged	吨	ton	0	0	0.00
化学需氧量排放总量	Discharge Amount of Chemical Oxygen Demand	吨	ton	25889.04	27615.18	-6.25
氨氮排放总量	Discharge Amount of Ammonia and Nitrogen	吨	ton	2468.11	2796.91	-11.76
废水排放总量	Waste Water Discharged	万吨	10 000 tons	58722.15	53421.38	9.92

6-10 环境质量状况
ENVIRONMENT CONDITION

项目	Itme	单位	Unit	2018 年	2017 年	2018 年比 2017 年增（+）减（-）% 2018 Compared to 2017（+/-）
一、二类海水水质点位比例	Point Location Proportion of Sea Water Quality of Category 2	%	%	90.6	90.6	0.00
市区区域环境噪声平均等效声级	The Average Equivalent Sound Level of the Urban Regional Environmental Noise	分贝（A）	db（A）	56.9	57.3	-0.70
市区道路交通噪声平均等效声级	The Average Equivalent Sound Level of the Urban Road Traffic Noise	分贝（A）	db（A）	68	68.7	-1.02

6-11 工业“三废”排放情况
DISCHARGE CONDITIONS OF INDUSTRIAL WASTE WSTER，WASTE GAS AND SOLID WASTE

项目	Itme	单位	Unit	2018 年	2017 年	2018 年比 2017 年增（+）减（-）% 2018 Compared to 2017（+/-）
工业废水排放总量	Industrial Waste Water Discharged	万吨	10 000 tons	5527	5613.22	-1.54
废水治理设施数	Number of Facilities for Treatment of Wsate water	套	set	365	391	-6.65
废水治理设施处理能力	Capacity of Facilities for Treatment of Waste Water	万吨 / 日	10 000 tons/day	38.08	40.02	-4.85
进入城市污水处理厂量	Volume Handled by Sewage Treatment Plant	万吨	10 000 tons	5030.47	5195.84	-3.18
化学需氧量排放量	Discharge Amount of Chemical Oxygen Demand	吨	ton	1620.28	2184.05	-25.81
氨氮排放量	Discharge Amount of Ammonia and Nitrogen	吨	ton	75.99	115.17	-34.02
工业废气排放总量	Industrial Waste Air Discharged	万标立方米	10 000 cu.m	35367746	28818474	22.73
废气治理设施数	Number of Facilities for Treatment of Waste Air	套	set	2071	1704	21.54
其中：脱硫设施数	of which： Desulphurization Facilities	套	set	268	270	-0.74
废气治理设施处理能力	Capacity of Facilities for Treatment of Waste Air	万标立方米 / 时	10 000 cu.m/hr	11986	10377.1	15.50
二氧化硫去除量	Sulphur Dioxide Removed	吨	ton	215570.67	225677.97	-4.48
二氧化硫排放量	Sulphur Dioxide Emission	吨	ton	4227.39	5136.54	-17.70
烟（粉）尘排放量	Soot（Dust）Emission	吨	ton	4613.88	7244.52	-36.31
工业固体废物产生量	Industrial Solid Wastes Producted	万吨	10 000 tons	793.36	769.55	3.09
其中：危险废物	of which： Hazardous Wastes	吨	ton	147026	114589	28.31
工业固体废物综合利用量	Industrial Solid Wastes Comprehensive Utilized	万吨	10 000 tons	734.36	707.17	3.84
工业固体废物排放量	Industrial Solid Wastes Discharged	万吨	10 000 tons	0	0	0.00
其中：危险废物	of whick： Hazardous Wastes	吨	ton	0	0	0.00
污染治理项目完成投资	Investment in Treatment Projects of Pollution	万元	10 000 yuan	41191.94	61194.7	-32.69
污染治理项目数	Number of Treatment Projects of Pollution	个	item	31	62	-50.00
当年竣工治理项目数	Number of Completed Treatment Projects in the Year	个	item	25	48	-47.92

主要统计指标解释

供水综合生产能力　指按供水设施取水、净化、送水、出厂输水干管等环节设计能力计算的综合生产能力。

年末供水管道长度　指从送水泵至用户水表之间所有管道的长度。不包括新安装尚未使用的管道。

用水普及率指城市用水人口数与城市人口总数的比率。计算公式：

$$用水普及率 = \frac{城市用水人口数}{城市人口总数} \times 100\%$$

全年供水总量　指报告期供水企业（单位）供出的全部水量。包括有效供水量和漏损水量。

全年供气总量　指全年燃气企业（单位）向用户供应的燃气数量。包括销售量和损失量。

用气普及率　指报告期末使用燃气的城市人口数与城市人口总数的比率。计算公式为：

$$用气普及率 = \frac{城市用气人口数}{城市人口总数} \times 100\%$$

年末道路长度　指年末道路长度和与道路相通的广场、桥梁、隧道的长度，按车行道中心线计算。在统计时只统计路面宽度在 3.5 米（含 3.5 米）以上的各种铺装道路，包括开放型工业区和住宅区道路在内。

工业废气排放量　指报告期内企业厂区内燃料燃烧和生产工艺过程中产生的各种排入大气的含有污染物的气体的总量，以标准状态（273K，101325Pa）计算。

工业废水排放量　指经过企业厂区所有排放口排到企业外部的工业废水量。包括生产废水、外排的直接冷却水、超标排放的矿井地下水和与工业废水混排的厂区生活污水，不包括外排的间接冷却水（清污不分流的间接冷却水应计算在内）。

工业废水排放达标量　指各项指标都达到国家或地方排放标准的外排工业废水量，包括未经处理外排达标的和经过处理后外排达标的和两部分。国家排放标准见 GB8978-88。

工业固体废物产生量　指企业在生产过程中产生的固体状、半固体状和高浓度液体状废弃物的总量，包括危险废物、冶炼废渣、粉煤灰、炉渣、煤矸渣、尾矿、放射性废物和其他废物等；不包括矿山开采的剥离废石和掘进废石（煤矸石和呈酸性或碱性的废石除外）酸性或碱性废石是指采掘的废石其流经水、雨淋水的 pH 值小于 4 或 pH 值大于 10.5 者。

工业粉尘排放量　指企业在生产工艺过程中排放的能在空气中悬浮一定时间的固体颗粒物排放量。如钢铁企业的耐火材料粉尘、焦化企业的筛焦系统粉尘、烧结机的粉尘、石灰窑的粉尘、建材企业的水泥粉尘等。不包括电厂排入大气的烟尘。

化学需氧量（COD）　测量有机和无机物质化学分解所消耗氧的质量浓度的水污染指数。

Explanatory Notes on Main Statistical Indicators

Production Capacity of Water Supply　refers to the designed comprehensive production capacity of water facilities，covering the 4 links of water collection，purification，conveyance，and outflow through trunk pipelines.

Length of Water Supply Pipelines at the Year-end　refers to the total length of all the pipelines between the water pumps and the user water meters，excluding pipelines newly installed but not used yet.

Coverage Rate of Urban Population with Access to Tap Water refers to the ratio of the urban population with access to tap water to the total urban population. The formula is:

Coverage rate of urban population with access to tap water=（Urban population with access to tap water）/（Urban population）×100%

Annual Volume of Water Supply refers to the total volume of water supplied by water-works（units）during the reference period, including both the effective water supply and loss during the water supply.

Annual Volume of Gas Supply refers to the total volume of gas provided to users by gas-producing enterprises（units）in a year, including the volume sold and the volume lost.

Coverage Rate of Urban Population with Access to Gas refers to the ratio of the urban population with access to gas to the total urban population at the end of the reference period.The formula is:

Coverage rate of urban population with access to gas=（Urban population with access to gas/Urban population）×100%

Length of Paved Roads at the Year-end refers to the length of roads with paved surface including squares bridges and tunnels connected with roads by the end of the year.Length of the roads is measured by the central lines for vehicles for paved roads with a width of 3.5 meters and over, including roads in open-ended factory compounds and residential quarters.

Industrial Waste Air Emission refers to discharge into atmosphere of waste air containing pollutants generated from fuel burning and production process in enterprises within a given period of time.It is calculated at standard status（273K, 101325Pa）.

Waste Water Discharged by Industry refers to the volume of waste water discharged by industrial enterprises through all their outlets, including waste water from production process, directly cooled water, groundwater from mining wells which does not meet discharge standards and sewage from households mixed with waste water produced by industrial activities, but excluding indirectly cooled water discharged（It should be included if the discharge is not separated with waste water）.

Industrial Waste Water Meeting Discharge Standards refers to volume of industrial waste water discharge which, with or without treatment, reaches national or local standards with regard to all pollutants.National Discharge standards see GB8978-88.

Industrial Solid Wastes Produced refers to total volume of solid, semi-solid and high concentration liquid residues produced by industrial enterprises from production process in a given period of time, including hazardous wastes, slag, coal ash, gangue, tailings, radioactive residues and other wastes, but excluding stones stripped or dug out in mining（gangue and acid or alkaline stones not included）.A stone is acid or alkaline depending on the pH value of the water below 4 or above 10.5 when the stone is in, or soaked by, the water.

Industrial Dust Emission refers to volume of dust emitted by production process of enterprises and suspended in the air for a given period of time, including dust from refractory material of iron and steel works, dust from coke-screening systems and sintering machines of coke plants, dust from lime kilns and dust from cement production in building material enterprises, but excluding soot and dust emitted from power plants.

Chemical Oxygen Demand（COD） refers to index of water pollution measuring the mass concentration of oxygen consumed by the chemical breakdown of organic and inorganic matter.

能源消耗 7

CONSUMPTION OF ENERGY

简要说明

一、本篇资料的主要内容

本篇资料主要反映了全市规模以上工业主要能源消费与库存情况，主要包括规模以上工业主要能源消费与库存、规模以上工业主要能源分行业消费量、重点耗能工业企业能源加工转换、规模以上工业主要能源工业消费量等方面的资料。

二、本篇资料的来源

本篇资料来源于规模以上工业能源统计年报，由市统计局能源统计处整理提供。

Brief Introduction

I. Main Content

Data in this chapter show the consumption and stock of major energy of industry above designated size，including consumption and stock of major energy of industry above designated size，major energy consumption of industry above designated size grouped by sector，energy conversion of major energy-consuming industrial enterprises and major energy consumption of industry above designated size，etc.

II. Source of Data

Data in this chapter are based on the annual report of energy consumed by industrial enterprises above designated size. The data are provided by the Division of Energy Statistics of Qingdao Municipal Bureau of Statistics.

7-1　规模以上工业主要能源消费与库存（2018 年）
CONSUMPTION AND STOCK OF MAJOR ENERGY OF INDUSTRY ABOVE DESIGNATED SIZE（2018）

名称	Name	计算单位	Unit	年初库存 Stock at Year-beginning	本年消费 Consumption in the Year	#工业 Industry	非工业 Non-industry	年末库存 Stock at Year-end
原　煤	Raw Coal	吨	ton	1604467.69	11437971.31	11401665.54	36305.77	1683068.85
焦　炭	Coke	吨	ton	295.00	1491996.00	1491996.00		76180.00
焦炉煤气	Coking Gas	万立方米	10 000 cu.m		36141.20	36141.20		
高炉煤气	Blast Furnace Gas	万立方米	10 000 cu.m		158161.70	158161.70		
原　油	Grude Oil	吨	ton	391907.36	15422450.59	15422419.39	31.20	418902.36
汽　油	Petrol	吨	ton	184.35	35328.55	21858.80	13469.75	59.41
煤　油	Kerosene	吨	ton		638.92	480.94	157.98	
柴　油	Diesel Oil	吨	ton	1660.73	62561.97	47021.70	15540.27	2654.71
燃料油	Fuel Oil	吨	ton	8458.97	89822.04	89651.16	170.88	9224.79
液化石油气	Liquefied Petroler Gas	吨	ton	44.75	99389.59	99362.94	26.65	
炼厂干气	Refinery Dry Gas	吨	ton		446466.00	446466.00		
热　力	Heat	百万千焦	million kJ		18097873.17	17615671.95	482201.22	
电　力	Electricity	万千瓦时	10 000kW·h		1819017.44	1800302.87	18714.57	

补充资料：2018 年综合能源消费量 1492.19 万吨标准煤。
Note：In 2018，comprehensive energy consumption is 1492.19 million rons SCE.

7-2 规模以上工业主要能源分行业消费量（2018 年）
MAJOR ENERGY CONSUMPTION OF INDUSTRY ABOVE DESIGNATED SIZE BY SECTOR（2018）

名称	Name	原煤（吨）Raw Coal（ton）	焦炭（吨）Coke（ton）	焦炉煤气（万立方米）Coking Gas（10 000 cu.m）	原油（吨）Crude Oil（ton）	汽油（吨）Petrol（ton）
**总　计**	**Total**	**11437971**	**1491996**	**36141**	**15422451**	**35329**
采 掘 业	Mining	20				1
制 造 业	Manufacturing	1435674	1491996	4949	15422451	34167
电力煤气及水生产供应业	Production and Supply of Electricity，Gas and Water	10002277		31192		1160

7-2 续表
Continued

名称	Name	煤油（吨）Kerosene（ton）	柴油（吨）Diesel Oil（ton）	燃料油（吨）Fuel Oil（ton）	液化石油气（吨）Liquefied Petroleum Gsa（ton）	热力（百万千焦）Heat（millon kJ）	电力（万千瓦时）Electricity（10 000kW·h）
**总　计**	**Total**	**639**	**62562**	**89822**	**99390**	**18097873**	**1819017**
采 掘 业	Mining		272				1660
制 造 业	Manufacturing	639	60235	88777	99389	17343697	1607106
电力煤气及水生产供应业	Production and Supply of Electricity，Gas and Water		2055	1045	1	754176	210252

7-3 重点耗能工业企业能源加工转换（2018 年）

ENERGY CONVERSION OF MAJOR ENERGY-CONSUMING INDUSTRIAL ENTERPRISES（2018）

名称	Name	单位	Unit	能源消费合计 Total Energy Constumption	# 加工转换投入 Conversion Input	火电 Thermal Power	供热 Leating	炼油 Petrolume Refining	制气 Gas Production	能源加工转换产出 Conversion Output of Energy
原 煤	Raw Coal	吨	ton	10680529.47	10128200.95	5937193.23	4191007.72			
洗精煤	Dressing Coal	吨	ton	2129966.00	2129966.00					
煤制品	Coal Products	吨	ton	13512.37	13512.37		13512.37			
焦 炭	Coke	吨	ton	1490948.00						1532645.00
其他焦化产品	Other Coking Products	吨	ton	51407.97						77013.00
焦炉煤气	Coking Gas	万立方米	10 000 cn.m	31192.20	31192.20	29745.00	1447.20			36675.40
高炉煤气	Blast Furnace GAS	万立方米	10 000 cn.m	148855.40	148855.40	141583.90	7271.50			
原 油	Raw Oil	吨	ton	15422347.01	15407527.01			15407527.01		
汽 油	Petrol	吨	ton	61.55						4739129.00
煤 油	Kerosene	吨	ton							1885917.00
柴 油	Diesel Oil	吨	ton	1644.32	180.00	180.00				3828611.46
燃料油	Fule Oil	吨	ton	30344.00	30259.00	777.00	103.00	29379.00		217949.00
液化石油气	Liquefied Petroleum Gas	吨	ton							1124441.00
炼厂干气	Refinery Dry Gas	吨	ton	446466.00	8260.00	826.00	7434.00			446466.00
其他石油制品	Other Petroleum Products	吨	ton	773305.44	332657.00			332657.00		1235872.50
热 力	Heat	百万千焦	million kJ	1372551.00						77971831.92
电 力	Electricity	万千瓦时	10 000kW·h	499415.20						1817014.92
其他燃料	Other Fuel	吨标准煤	ton SCE							
**能源合计**	**Total**	**吨标准煤**	**ton SCE**	**37219514.30**	**33364047.39**	**5482675.02**	**3442332.14**	**22415572.53**		**28336630.86**

7-4 规模以上工业主要能源工业消费量（2018 年）

MAJOR ENERGY CONSUMPTION OF INDUSTRY ABOVE DESIGNATED SIZE（2018）

行业	Sector
**总　　计**	**Total**
煤炭开采和洗选业	Mining and Washing of Coal
石油和天然气开采业	Extraction of Petroleum and Natural Gas
黑色金属矿采选业	Mining of Ferrous Metal Ores
有色金属矿采选业	Mining of Non-ferrous Metal Ores
非金属矿采选业	Mining and Processing of Nonmetal Ores
开采辅助活动	Auxiliary Activities of Mining
其他采矿业	Mining of Other Ores
农副食品加工业	Processing of Food from Agricultural Products
食品制造业	Manufacture of Foods
酒、饮料和精制茶制造业	Manufacture of Liquor，Beverage and Refind Tea
烟草制品业	Manufacture of Tobacco
纺织业	Manufacture of Textile
纺织服装、服饰业	Manufacture of Textil Wearing Apparel
皮革、毛皮、羽毛及其制品和制鞋业	Manufacture of Leather，Fur，Feathre&Its Products Footwear
木材加工和木、竹、藤、棕、草制品业	Processing of Timbers，Manufacture of Wood，Bamboo，Rattan，Palm and Straw Products
家具制造业	Manufacture of Furniture
造纸和纸制品业	Manufacture of Paper and Paper Products
印刷和记录媒介复制业	Printing，Reproduction of Recording Media
文教、工美、体育和娱乐用品制造业	Manufacture of Articles for Culture，Arts& Crafts，Sports and Entertainment
石油加工、炼焦和核燃料加工业	Processing of Petroleum，Coking，Processing of Nucleus Fuel
化学原料和化学制品制造业	Manufacture of Chemical Raw Material and Chemical Products
医药制造业	Manufacture of Medicines
化学纤维制造业	Manufacture of Chemical Fiber
橡胶和塑料制品业	Manufacture of Rubber and Plastic
非金属矿物制品业	Manufacture of Non-metallic Mineral Products
黑色金属冶炼和压延加工业	Smelting and Pressing of Ferrous Metals
有色金属冶炼和压延加工业	Smelting and Pressing of Non-ferrous Metals
金属制品业	Manufacture of Metal Products
通用设备制造业	Manufacture of General Purpose Machinery
专用设备制造业	Manufacture of Special Purpose Machinery
汽车制造业	Manufacture of Vehicle
铁路、船舶、航空航天和其他运输设备制造业	Manufacture of Transport Equipment for Railway，Shipping，Aerospace and other uses
电气机械和器材制造业	Manufacture of Electrical Machinery &Equipment
计算机、通信和其他电子设备制造业	Manufacture of Computer，Communication Equipment and Other Electronic Equipmen
仪器仪表制造业	Manufacture of Measuring Imstrument
其他制造业	Manufacture of Other Products
废弃资源综合利用业	Recycling and Disposal of Waste Resources
金属制品、机械和设备修理业	Manufacture of Metal Products，Machinery and Equipment
电力、热力生产和供应业	Production and Supply of Electric Power and Heat Power
燃气生产和供应业	Production and Supply of Gas
水的生产和供应业	Production and Supply of Water

原煤<br>（吨）<br>Raw Coal<br>（ton）	洗精煤<br>（吨）<br>Dressing Coal<br>（ton）	其他洗煤<br>（吨）<br>Other Dressing Coal<br>（ton）	煤制品<br>（吨）<br>Coal Products<br>（ton）	焦炭<br>（吨）<br>Coke<br>（ton）
**11437971**	**2129966**	**40361**	**14811**	**1491996**
20				
33217		278	302	
1392				
15589				
8865			6	
5785		1006		
5203				
247			19	
75459			379	
3789				
6976				
10688				
383030		39077		
50			562	
113054				
204099				
551328	2129966			1490948
1140				
4481				747
3943				230
2030			14	
1078				71
972				
1642			16	
1616				
9999992			13512	
2285				

7-4 续表 1
Continued

行业	Sector
**总　计**	**Total**
煤炭开采和洗选业	Mining and Washing of Coal
石油和天然气开采业	Extraction of Petroleum and Natural Gas
黑色金属矿采选业	Mining of Ferrous Metal Ores
有色金属矿采选业	Mining of Non-ferrous Metal Ores
非金属矿采选业	Mining and Processing of Nonmetal Ores
开采辅助活动	Auxiliary Activities of Mining
其他采矿业	Mining of Other Ores
农副食品加工业	Processing of Food from Agricultural Products
食品制造业	Manufacture of Foods
酒、饮料和精制茶制造业	Manufacture of Liquor，Beverage and Refind Tea
烟草制品业	Manufacture of Tobacco
纺织业	Manufacture of Textile
纺织服装、服饰业	Manufacture of Textil Wearing Apparel
皮革、毛皮、羽毛及其制品和制鞋业	Manufacture of Leather，Fur，Feathre&Its Products Footwear
木材加工和木、竹、藤、棕、草制品业	Processing of Timbers，Manufacture of Wood，Bamboo，Rattan，Palm and Straw Products
家具制造业	Manufacture of Furniture
造纸和纸制品业	Manufacture of Paper and Paper Products
印刷和记录媒介复制业	Printing，Reproduction of Recording Media
文教、工美、体育和娱乐用品制造业	Manufacture of Articles for Culture，Arts& Crafts，Sports and Entertainment
石油加工、炼焦和核燃料加工业	Processing of Petroleum，Coking，Processing of Nucleus Fuel
化学原料和化学制品制造业	Manufacture of Chemical Raw Material and Chemical Products
医药制造业	Manufacture of Medicines
化学纤维制造业	Manufacture of Chemical Fiber
橡胶和塑料制品业	Manufacture of Rubber and Plastic
非金属矿物制品业	Manufacture of Non-metallic Mineral Products
黑色金属冶炼和压延加工业	Smelting and Pressing of Ferrous Metals
有色金属冶炼和压延加工业	Smelting and Pressing of Non-ferrous Metals
金属制品业	Manufacture of Metal Products
通用设备制造业	Manufacture of General Purpose Machinery
专用设备制造业	Manufacture of Special Purpose Machinery
汽车制造业	Manufacture of Vehicle
铁路、船舶、航空航天和其他运输设备制造业	Manufacture of Transport Equipment for Railway，Shipping，Aerospace and other uses
电气机械和器材制造业	Manufacture of Electrical Machinery &Equipment
计算机、通信和其他电子设备制造业	Manufacture of Computer，Communication Equipment and Other Electronic Equipmen
仪器仪表制造业	Manufacture of Measuring Imstrument
其他制造业	Manufacture of Other Products
废弃资源综合利用业	Recycling and Disposal of Waste Resources
金属制品、机械和设备修理业	Manufacture of Metal Products，Machinery and Equipment
电力、热力生产和供应业	Production and Supply of Electric Power and Heat Power
燃气生产和供应业	Production and Supply of Gas
水的生产和供应业	Production and Supply of Water

焦炉煤气<br>（万立方米）<br>Coking Gas<br>（10 000 cu.m）	高炉煤气<br>（万立方米）<br>Blast Furnace GAS<br>（10 000 cu.m）	原油<br>（吨）<br>Crude Oil<br>（ton）	汽油<br>（吨）<br>Petrol<br>（ton）	煤油<br>（吨）<br>Kerosene<br>（ton）
**36141**	**158162**	**15422451**	**35329**	**639**
			1	
			3411	316
			669	
			270	23
			493	
			1575	
			823	
			54	
			153	
			354	
			1304	25
			1579	23
		15422347	151	
			1452	
			815	
		1	2472	
4949	9306	61	1710	
			99	
			111	
		15	2311	8
			3517	70
			2141	
		19	4285	51
			982	123
			1890	
			1038	
			362	
			104	
			41	
31192	148855		278	
			321	
			562	

7-4 续表 2
Continued

行业	Sector
**总　计**	**Total**
煤炭开采和洗选业	Mining and Washing of Coal
石油和天然气开采业	Extraction of Petroleum and Natural Gas
黑色金属矿采选业	Mining of Ferrous Metal Ores
有色金属矿采选业	Mining of Non-ferrous Metal Ores
非金属矿采选业	Mining and Processing of Nonmetal Ores
开采辅助活动	Auxiliary Activities of Mining
其他采矿业	Mining of Other Ores
农副食品加工业	Processing of Food from Agricultural Products
食品制造业	Manufacture of Foods
酒、饮料和精制茶制造业	Manufacture of Liquor，Beverage and Refind Tea
烟草制品业	Manufacture of Tobacco
纺织业	Manufacture of Textile
纺织服装、服饰业	Manufacture of Textil Wearing Apparel
皮革、毛皮、羽毛及其制品和制鞋业	Manufacture of Leather，Fur，Feathre&Its Products Footwear
木材加工和木、竹、藤、棕、草制品业	Processing of Timbers，Manufacture of Wood，Bamboo，Rattan，Palm and Straw Products
家具制造业	Manufacture of Furniture
造纸和纸制品业	Manufacture of Paper and Paper Products
印刷和记录媒介复制业	Printing，Reproduction of Recording Media
文教、工美、体育和娱乐用品制造业	Manufacture of Articles for Culture，Arts& Crafts，Sports and Entertainment
石油加工、炼焦和核燃料加工业	Processing of Petroleum，Coking，Processing of Nucleus Fuel
化学原料和化学制品制造业	Manufacture of Chemical Raw Material and Chemical Products
医药制造业	Manufacture of Medicines
化学纤维制造业	Manufacture of Chemical Fiber
橡胶和塑料制品业	Manufacture of Rubber and Plastic
非金属矿物制品业	Manufacture of Non-metallic Mineral Products
黑色金属冶炼和压延加工业	Smelting and Pressing of Ferrous Metals
有色金属冶炼和压延加工业	Smelting and Pressing of Non-ferrous Metals
金属制品业	Manufacture of Metal Products
通用设备制造业	Manufacture of General Purpose Machinery
专用设备制造业	Manufacture of Special Purpose Machinery
汽车制造业	Manufacture of Vehicle
铁路、船舶、航空航天和其他运输设备制造业	Manufacture of Transport Equipment for Railway，Shipping，Aerospace and other uses
电气机械和器材制造业	Manufacture of Electrical Machinery &Equipment
计算机、通信和其他电子设备制造业	Manufacture of Computer，Communication Equipment and Other Electronic Equipmen
仪器仪表制造业	Manufacture of Measuring Imstrument
其他制造业	Manufacture of Other Products
废弃资源综合利用业	Recycling and Disposal of Waste Resources
金属制品、机械和设备修理业	Manufacture of Metal Products，Machinery and Equipment
电力、热力生产和供应业	Production and Supply of Electric Power and Heat Power
燃气生产和供应业	Production and Supply of Gas
水的生产和供应业	Production and Supply of Water

柴油<br>（吨）<br>Diesel Oil<br>（ton）	燃料油<br>（吨）<br>Fule Oil<br>（ton）	液化石油气<br>（吨）<br>Liquefied Petroleum Gas<br>（ton）	炼厂干气<br>（吨）<br>Refinery Dry Gas<br>（ton）
**62562**	**89822**	**99390**	**446466**
272			
3793	788	343	
848			
856		252	
298		9	
2159			
358	136		
44			
389			
1099			
1220	231		
934	12		
119	29379		446466
2338	703	97090	
432			
2083	1620	988	
23618	55434	19	
859		22	
511			
1972	39	371	
3833	7	37	
1416			
1911		47	
6193	430	41	
1230		152	
1154		19	
423			
118			
27			
1658	1045	1	
27			
370			

7-4 续表 3
Continued

行业	Sector
**总　　计**	**Total**
煤炭开采和洗选业	Mining and Washing of Coal
石油和天然气开采业	Extraction of Petroleum and Natural Gas
黑色金属矿采选业	Mining of Ferrous Metal Ores
有色金属矿采选业	Mining of Non-ferrous Metal Ores
非金属矿采选业	Mining and Processing of Nonmetal Ores
开采辅助活动	Auxiliary Activities of Mining
其他采矿业	Mining of Other Ores
农副食品加工业	Processing of Food from Agricultural Products
食品制造业	Manufacture of Foods
酒、饮料和精制茶制造业	Manufacture of Liquor，Beverage and Refind Tea
烟草制品业	Manufacture of Tobacco
纺织业	Manufacture of Textile
纺织服装、服饰业	Manufacture of Textil Wearing Apparel
皮革、毛皮、羽毛及其制品和制鞋业	Manufacture of Leather，Fur，Feathre&Its Products Footwear
木材加工和木、竹、藤、棕、草制品业	Processing of Timbers，Manufacture of Wood，Bamboo，Rattan，Palm and Straw Products
家具制造业	Manufacture of Furniture
造纸和纸制品业	Manufacture of Paper and Paper Products
印刷和记录媒介复制业	Printing，Reproduction of Recording Media
文教、工美、体育和娱乐用品制造业	Manufacture of Articles for Culture，Arts& Crafts，Sports and Entertainment
石油加工、炼焦和核燃料加工业	Processing of Petroleum，Coking，Processing of Nucleus Fuel
化学原料和化学制品制造业	Manufacture of Chemical Raw Material and Chemical Products
医药制造业	Manufacture of Medicines
化学纤维制造业	Manufacture of Chemical Fiber
橡胶和塑料制品业	Manufacture of Rubber and Plastic
非金属矿物制品业	Manufacture of Non-metallic Mineral Products
黑色金属冶炼和压延加工业	Smelting and Pressing of Ferrous Metals
有色金属冶炼和压延加工业	Smelting and Pressing of Non-ferrous Metals
金属制品业	Manufacture of Metal Products
通用设备制造业	Manufacture of General Purpose Machinery
专用设备制造业	Manufacture of Special Purpose Machinery
汽车制造业	Manufacture of Vehicle
铁路、船舶、航空航天和其他运输设备制造业	Manufacture of Transport Equipment for Railway，Shipping，Aerospace and other uses
电气机械和器材制造业	Manufacture of Electrical Machinery &Equipment
计算机、通信和其他电子设备制造业	Manufacture of Computer，Communication Equipment and Other Electronic Equipmen
仪器仪表制造业	Manufacture of Measuring Imstrument
其他制造业	Manufacture of Other Products
废弃资源综合利用业	Recycling and Disposal of Waste Resources
金属制品、机械和设备修理业	Manufacture of Metal Products，Machinery and Equipment
电力、热力生产和供应业	Production and Supply of Electric Power and Heat Power
燃气生产和供应业	Production and Supply of Gas
水的生产和供应业	Production and Supply of Water

其他油制品<br>（吨）<br>Other Petroleum Products<br>（ton）	热力<br>（百万千焦）<br>Heat<br>（million kJ）	电力<br>（万千瓦时）<br>Electricity<br>（10 000kW·h）	其他燃料<br>（吨标准煤）<br>Other Fuel<br>（ton SCE）
**774635**	**18097873**	**1819017**	**3805**
		1660	
2	1917701	93999	621
	266708	23973	75
	2203876	20587	
	1292481	20040	327
	1813260	28245	
	98705	11605	210
		2962	
	1337	6742	
	881000	14926	
	14464	14185	
	43358	28333	964
715249	5241	130045	
59257	3787193	203155	
	517661	14493	
	4656	3760	
	1276995	147137	
	39459	96326	
	343085	213760	
	50276	20713	
12	59123	83085	
	68569	71300	442
	17331	32152	181
	711015	110959	
115	957547	70771	839
	606240	78008	146
	366415	56969	
		4807	
		1946	
		166	
		1956	
	754176	176282	
		9540	
		24430	

主要统计指标解释

工业企业能源消费量 工业企业能源消费包括工业企业在生产过程中作为燃料、动力、原料、辅助材料使用的能源以及工艺用能、非生产用能；作为能源加工转换企业，还要包括能源加工转换的投入量。工业企业能源消费量具体包括：

（1）用于本企业产品生产、工业性作业和其他生产性活动的能源。

（2）用于技术更新改造措施、新技术研究和新产品试制以及科学试验等方面的能源。

（3）用于经营维修、建筑及设备大修理、机电设备和交通运输工具等方面的能源。

（4）用于劳动保护的能源。

（5）其他非生产消费的能源。

不包括：

（1）由仓库发到车间，但在报告期最后一天没有消费的能源。这部分能源应在办理假退料手续后计入库存量。

（2）拨到外单位，委托外单位加工用的能源。

（3）调出本单位或借给外单位的能源。

工业生产能源消费 是指工业企业为进行工业生产活动所使用的能源。主要包括：

1. 用于本企业产品生产、工业性作业的能源，包括用作原料、材料、燃料、动力：作为能源加工转换企业，还包括用作加工转换的能源。

2. 产品生产过程中作为辅助材料使用的能源。

3. 生产工艺过程使用的能源。

4. 新技术研究、新产品试制、科学试验使用的能源。

5. 为了工业生产活动而在进行的各种修理过程中使用的能源。

6. 生产区内的劳动保护用能等。

Explanatory Notes on Main Statistical Indicators

Energy Consumption of Industrial Enterprises include energy in the production process as fuel，power，raw materials，supplementary materials，and for use of technology and non-production. As energy processing and conversion enterprises，also include energy processing and conversion of inputs. These specifically include：（1）Energy for the enterprise product，industrial production operations and other activities.（2）Energy for technical upgrading measures and new technology research and new product production and scientific experiments.（3）Energy for operation maintenance，construction and equipment overhaul，electrical and mechanical equipment and transport，and other aspects.（4）Energy for the protection of labor.（5）Energy for Other non-production and consumption. And these exclude：（1）Energy from the warehouse to the workshop，but out of consumption on the last day of the reporting period. This part of the energy should leave retreat materials handling procedures included stock.（2）Energy transferred to other units，entrusted with the processing.（3）Energy transferred out of the unit or loans to other units.

Energy Consumption for Industrial Production refers to energy for industrial enterprises in industrial production activities. These mainly include：1.Energy for the enterprise products，industrial operations，including energy as raw materials，materials，fuels，and power. As energy processing and conversion enterprises，also include energy for processing and conversion. 2. Energy as supplementary material in product process. 3. Energy used in production process. 4. Energy for new technologies，new product production，and scientific experiment. 5. Energy in process of repairing for industrial production activities. 6. Energy for labor protection in production areas and so on.

财政、金融和保险业 8

GOVERNMENT FINANCE FINACIAL INTERMEDIATION AND INSURANCE

简 要 说 明

一、本篇资料的主要内容

本篇资料主要反映了全市财政收支、金融和保险方面的情况，主要包括财政收入、财政支出、金融机构存贷款、保险业务开展等方面的资料。

二、本篇资料的来源

1、财政部分的资料来源于市财政局。

2、金融方面的资料来源于中国人民银行青岛市中心支行。

3、保险方面的资料来源于中国保险监督管理委员会青岛监管局。

本篇资料由市统计局国民经济核算处整理提供。

Brief Introduction

I. Main Content

Data in this chapter show the conditions of local government budgetary finance, banking and insurance, and securities, including government revenue and expenditure, deposits and loans of financial institutions and statistics on insurance companies.

II. Source of Data

(1) Data on local government finance are provided by Qingdao Municipal Finance Bureau.

(2) Data on banking are provided by Qingdao Branch of the People' s Bank of China.

(3) Data on insurance are provide by China Insurance Regulatory Commission of Qingdao Bureau.

Data in this chapter are prepared and compiled by the Division of National Accounts of Qingdao Municipal Bureau of Statistics.

8-1 主要年份地方财政收支
MAJOR YEAR'S REVENUE AND EXPENDRRURE OF LOCAL GOVERNMENT FINANCE

单位：万元（10 000 yuan）

年份 Year	财政收入 Revenue of Government Finance	财政支出 Expenditure of Government Finance
1949	1527	306
1952	19809	2226
1957	29875	3556
1962	32869	4327
1965	47682	6280
1970	95778	7825
1975	90220	11966
1978	130749	19427
1980	124835	20064
1985	165155	41996
1988	201206	93931
1990	242303	133875
1991	259377	137716
1992	275589	155696
1993	182348	211938
1994	227490	277331
1995	294771	376982
1996	379674	469569
1997	476804	564787
1998	580434	678575
1999	680089	740937
2000	800120	878702
2001	987080	1097848
2002	1006616	1243880
2003	1201398	1471747
2004	1305136	1646214
2005	1763412	2030622
2006	2257663	2367875
2007	2925798	3211777
2008	3424359	3694111
2009	3769896	4335754
2010	4526138	5323888
2011	5661400	6580605
2012	6701820	7659801
2013	7889313	10142273
2014	8952450	10747138
2015	10063220	12228664
2016	11000303	13528516
2017	11572389	14030252
2018	12319138	15597764

注：1. 1993 年以后实行新制度，财政收入数与历年不可比。
2. 2002 年以后，财政收入、支出为一般预算数。

Note：1. Since 1993，new regulations have been adopted in calculating revenue of government finance，the figutrs are not comparable with those over the years.
2. Since 2002，revenue of government finance and expenditure of government finance refer to general budgetary revenue and expenditure.

8-2 分市、区公共财政预算收入（2018年）

PULBIC FINANCE BUDGET REVENUE BY CITY AND DISTRICL（2018）

单位：万元（10 000 yuan）

市、区名称	Region	一般公共预算收入 Public Finance Bodget Revenue	#增值税 Value-added Tax	#营业税 Business Tax	#企业所得税 Enterprise Income Tax
**全　市**	**Whole Municipality**	**12319138**	**3278946**	**3713**	**1523764**
市南区	Shiman District	921789	312727	1117	165931
市北区	Shiben District	1101239	320692	986	149746
李沧区	Licang District	1001717	191628	2077	65718
崂山区	Laoshan District	1532191	398735	-4376	294115
黄岛区	Huangdao District	2498092	759811	287	367296
保税港区	Qingdao Free Trade Port Area of Chinat	128520	43889	6	42778
城阳区	Chengyang District	1125417	437273	26	140757
即墨区	Jimo District	1111918	270077	749	72135
胶州市	Jiaozhou	1003788	241928	1327	60461
平度市	Pingdu	560180	133525	869	31456
莱西市	Laixi	493777	81315	608	26529
红岛经济区	Qingdao National High-tech Industrial Development Zone	318152	87346	37	39375
市本级	Municipal Level	522358			67467

8-2 续表
Continued

单位：万元（10 000 yuan）

市、区名称	Region	# 个人所得税 Personal Income Tax	# 城市维护建设税 Urban Maintenance and Development Tax	# 基金预算收入 Funds Budgetary Revenue
**全 市**	**Whole Municipality**	**487635**	**576986**	**8855214**
市南区	Shiman District	118780	43490	
市北区	Shiben District	50378	59369	
李沧区	Licang District	19962	38829	
崂山区	Laoshan District	90020	102369	743328
黄岛区	Huangdao District	84842	137234	1720353
保税港区	Qingdao Free Trade Port Area of Chinat	5380	6338	4849
城阳区	Chengyang District	37812	61782	925418
即墨区	Jimo District	22005	39316	828113
胶州市	Jiaozhou	18903	38907	989641
平度市	Pingdu	14125	20904	282606
莱西市	Laixi	13911	13853	29216
红岛经济区	Qingdao National High-tech Industrial Development Zone	11517	14595	1131694
市本级	Municipal Level			2199996

8-3 分市、区公共财政预算支出（2018年）
PUBLIC FINANCE BUDGET REVENUE BY CITY AND DISTRICT（2018）

单位：万元（10 000 yuan）

市、区名称	Region	一般公共预算支出 Pubic Finance Budget Revenue	# 一般公共服务支出 General Public Services	# 公共安全 Public Securty	# 教育 Education	# 科学技术 Science and Technology	# 文化体育与传媒 Culture，Sport and Media
**全　市**	**Whole Municipality**	**15597764**	**1626828**	**1083753**	**2630041**	**451472**	**240026**
市南区	Shiman District	614559	74049	34241	108140	5793	2461
市北区	Shiben District	753358	127340	30049	189206	17016	6533
李沧区	Licang District	869924	46414	20776	142081	131791	7904
崂山区	Laoshan District	987755	82625	45375	128072	99244	6877
黄岛区	Huangdao District	2149197	307976	102626	507568	23724	17769
保税港区	Qingdao Free Trade Port Area of Chinat	67940	19746	1202	104	4874	72
城阳区	Chengyang District	736420	85269	35665	216158	22233	3841
即墨区	Jimo District	1687264	140956	46749	294528	14409	21976
胶州市	Jiaozhou	1169575	138150	66503	257362	19761	12861
平度市	Pingdu	995209	98809	39746	284225	6003	8249
莱西市	Laixi	767102	82346	26486	106439	1249	4652
红岛经济区	Qingdao National High-tech Industrial Development Zone	286695	25795	4750	40376	35128	10569
市本级	Municipal Level	4512766	397353	629585	355782	70247	136262

8-3 续表
Continued

单位：万元（10 000 yuan）

市、区名称	Region	#社会保障和就业 Social Security and Empooyment	#医疗卫生 Health Care	#节能环保 Environment Protection	#城乡社区事务 Urban and Rural Community Affairs	#农林水事务 Affairs of Agiculture Forest and Irrigatior	#基金预算支出 Funds Budgetary Expenditure
**全　市**	**Whole Municipality**	**1561217**	**1023535**	**248344**	**3320256**	**686776**	**9255764**
市南区	Shiman District	71534	33614	6006	209040	108	38572
市北区	Shiben District	96793	48452	5934	198480	46	212935
李沧区	Licang District	79010	31910	69362	313492	2945	158026
崂山区	Laoshan District	39791	54547	31661	326577	63928	519549
黄岛区	Huangdao District	222410	151131	37973	382317	109079	1847380
保税港区	Qingdao Free Trade Port Area of Chinat	2942	83	1	27560		9805
城阳区	Chengyang District	42436	49074	5144	223874	22211	1058540
即墨区	Jimo District	135735	103633	15785	344622	84837	1010711
胶州市	Jiaozhou	97946	89141	28996	257308	106962	1085727
平度市	Pingdu	175529	125398	12834	72631	123910	256028
莱西市	Laixi	147533	74350	12526	62688	76407	32778
红岛经济区	Qingdao National High-tech Industrial Development Zone	16803	9370	835	37135	3215	978863
市本级	Municipal Level	432755	252832	21287	864532	93128	2046850

8-4　主要年份金融系统人民币存贷款（年末余额）

MAJOR YEAR'S DEPOSITS AND LOANS OF FINANCIAL INSTITUTIONS（YEAR-END BALANCE）

单位：万元（10 000 yuan）

年份<br>Year	存款合计<br>Total Deposits	#企业存款<br>Deposits by Enterprises	#储蓄存款<br>Savings Deposits	贷款合计<br>Total Loans
1949	693	239	28	278
1952	9093	4308	1838	3322
1957	10372	3359	4037	30690
1962	20040	8579	3279	73601
1965	27331	11830	6347	64555
1970	38264	13024	7576	126382
1975	60622	22568	15637	207006
1978	66523	21004	21455	297184
1980	120644	38105	38609	379666
1985	344760	99736	155604	452142
1990	1235562	321542	655895	1535751
1991	1553564	411417	824428	1871462
1992	2076667	612868	1044165	2352449
1993	2747294	835617	1372239	2940658
1994	3769019	1399110	1918734	3610564
1995	5324026	2030382	2687888	4777864
1996	6991289	2630958	3499219	5941281
1997	7385092	2885632	4060259	6508776
1998	8183471	3018063	4591012	7239231
1999	9160001	3454570	4978045	8945730
2000	10560624	4386316	5353215	9564293
2001	12323942	4955336	6187409	10798871
2002	15225733	5819655	7449408	13044792
2003	18920868	7027227	9084693	16783917
2004	22462592	7924690	10894941	18472613
2005	26975372	8226661	13431016	20394792
2006	32453583	10144296	15676197	25779442
2007	38915882	13213434	17020383	30970553
2008	47353803	14641948	21233637	37483209
2009	63019764	21001185	25278658	48735326
2010	76592065	27271963	29123256	58862263
2011	86384994	49189415	31985099	69477500
2012	94348924	50978985	37576007	79465532
2013	109695588	60367185	41405946	88607439
2014	113703085	60791933	44358964	97200532
2015	125330268	45526577	50235905	107718528
2016	140071344	50912759	53263262	118916821
2017	143877550	53835435	53941843	132647913
2018	155321863	57270448	59137089	151941934

注：本表数据来源于人民银行，自 2015 年起，人民银行报表指标变化，企业存款指标暂用非金融企业存款，储蓄存款指标暂用住户存款，口径与以前年度不一致。

Note：The data in this table comes from the People's Bank of China. Since 2015, the report index of the People' s Bank of China has changed, the enterprise deposit index is temporarily used as the deposit of the enterprise， the savings deposit index is temporarily used for household deposit，The caliber is not consistent with the previous year.

8-5 金融系统人民币存贷款（年末余额）
DEPOSITS AND LOANS OF FINANCIAL INSTITUTIONS（YEAR-END BALANCE）

单位：万元（10 000 yuan）

项目	Item	2018 年	比年初增减数 Incremental/Reductions Compared to the Beginning of the Year
一、存款总计	Total Deposits	155321863	11444315
住户存款	Household Deposits	59137089	5186155
非金融企业存款	Non-Financial Corporate Deposits	57270448	3444106
广义政府存款	General Government Deposits	29329183	4414777
非银行业金融机构存款	Non-Banking Financial Institutions' s Deposits	9130588	-1637887
二、贷款总计	Total Loans	151941934	19238288
住户贷款	Household Loans	47898973	7331502
#短期贷款	Short-Term Loans	4421237	561679
中长期贷款	Medium And Long-Term Loans	43477736	6769823
非金融企业及机关团体贷款	Non-Financial Enterprises And Institutions/ Organizations' Loans	103704752	11922783
#短期贷款	Short-Term Loans	34346742	2197999
中长期贷款	Medium&Long-term Loans	62846188	6646810
票据融资	Notes Financing	5525190	2690625
非银行业金融机构贷款	Non-Banking Financial Institution' s Loans	20112	-193821

注：自 2015 年起，人民银行报表中指标发生变化，本表数据按照新指标提供。

Note：Since 2015, the indexes in the People' s Bank of China' s statements have changed, the data in the talbe are provided according to the new indexes.

8-6 国内保险业务（2000-2018年）

DOMESTIC INSURANCE BUSINESS（2000-2018）

项目	Item	2000年	2005年	2006年	2008年	2009年
**风险保障金额**	**Total domestic insurance Value**	**38357485**	**91691581**	**119396881**	**276989344**	**293519697**
**国内业务收入**	**Domestic Business Income**	**220780**	**495701**	**605143**	**1027258**	**1153101**
1. 保费收入	Premium Income	218714	495701	605143	1027258	1153101
#财产险	Propetry Insurance	85161	159746	199213	280008	339314
农业险	Agiculture Insurance	18	12	19	3135	4066
人身险	Personal Insurance	133535	335943	405911	744115	809721
**国内业务支出**	**Domestic Business Expenditure**	94877	214148	252477	423743	476377
1 . 赔款支出	Cham Expenditure	62275	111385	131321	196318	219546
#财产险	Property Insurance	58361	92700	115214	164415	188723
农业险	Agriculture Insurance	5	11	3	907	3452
人身险	Personal Insurance	3909	18674	16104	30996	27371
2 . 给付支出	Mature Payment	27955	32739	63144	132621	123056
3 . 退保	Surrended	4647	70024	58012	94804	133775

注：本表由青岛保监局提供，风险保障金额2012年之前为国内保险总值。

Note：This table was provided by the Qingdao Insurance Regulatory Bureau. The amount of risk protection before 2012 was the total value of domestic insurance.

单位：万元（10 000 yuan）

2010 年	2011 年	2012 年	2013 年	2014 年	2015 年	2016 年	2017 年	2018 年
**309244255**	**380829634**	**447476690**	**491953749**	**611632799**	**836499657**	**1687559828**	**2232390824**	**2337365406**
**1538526**	**1457269**	**1602881**	**1789854**	**2031421**	**2441172**	**3359013**	**3967170**	**4393970**
1538526	1457269	1602881	1789854	2031421	2441172	3359013	3967170	4393970
492190	556257	644269	744470	872805	920229	1046908	1062116	1269489
3261	3587	5046	6824	8395	12100	12734	15670	18043
1043075	897425	953566	1038560	1150220	1508843	2299371	2889384	3106438
497832	463751	592167	757404	1031415	1236281	1522375	1704218	1919252
279019	307564	360085	432494	492693	539320	681211	733493	917964
250966	286214	328877	392860	442168	492573	526586	554513	756987
2808	1087	1833	3820	5519	9724	16337	18232	10985
25245	20263	29375	35814	45006	37023	138288	160749	149992
109318	156188	154922	194592	274790	340786	455300	470266	493511
109496	69275	77159	130318	263932	356175	376777	500459	507777

主要统计指标解释

财政收入　指国家财政参与社会产品分配所取得的收入，是实现国家职能的财力保证。财政收入所包括的内容几经变化，目前主要包括：

（1）税收收入：包括增值税、营业税、企业所得税、个人所得税、资源税、城市维护建设税、房产税、印花税、城镇土地使用税、土地增值税、车船税、耕地占用税、契税、烟叶税、其他税收收入。

（2）非税收入：包括专项收入、行政事业性收费收入、罚没收入、国有资本经营收入、国有资源有偿使用收入、其他收入。

财政支出　国家财政将筹集起来的资金进行分配使用，以满足经济建设和各项事业的需要，主要包括：

（1）一般公共服务支出：反映政府提供一般公共服务的支出。

（2）公共安全：反映政府维护社会公共安全方面的支出，有关事务包括武装警察、公安、国家安全、检察、法院、司法行政、监狱、劳教、国家保密、缉私警察等。

（3）教育支出：反映政府教育事务支出。有关具体教育事务包括教育行政管理、学前教育、小学教育、初中教育、普通高中教育、普通高等教育、初等职业教育、中专教育、技校教育、职业高中教育、高等职业教育、广播电视教育、留学生教育、特殊教育、干部继续教育、教育机关服务等。

（4）科学技术：反映政府用于科学技术方面的支出。

（5）文化体育与传媒：反映政府在文化、文物、体育、广播电视、新闻出版等方面的支出。

（6）社会保障和就业：反映政府在社会保障与就业方面的支出。有关事项包括社会保障与就业管理事务、民政管理事务、财政对社会保险基金的补助、补充全国社会保障基金、行政事业单位离退休、企业改革补助、就业补助、抚恤、退役安置、社会福利、残疾人事业、城市居民最低生活保障、其他城镇社会救济、农村社会救济、自然灾害生活补助、红十字事务等。

（7）医疗卫生支出：反映政府医疗卫生方面的支出。具体包括医疗卫生管理事务支出、医疗服务支出、医疗保障支出、疾病预防控制支出、卫生监督支出、妇幼保健支出、农村卫生支出等。

（8）环境保护：反映政府环境保护支出。具体包括：环境保护管理事务支出、环境监测与监察支出、污染治理支出、自然生态保护支出、天然林保护工程支出、退耕还林支出、风沙荒漠治理支出、退牧还草支出、已垦草原退耕还草支出。

（9）城乡社区事务：反映政府城乡社区事务支出。具体包括：城乡社区管理事务支出、城乡社区规划与管理支出、城乡社区公共设施支出、城乡社区住宅支出、城乡社区环境卫生支出、建设市场管理与监督支出等。

（10）农林水事务：反映政府农林水事务方面的支出。具体包括农业、林业、水利、扶贫支出、农业综合开发支出等。

存款　指企业、机关、团体或居民根据资金必须收回的原则，把货币资金存入银行或其他信贷机构保管并取得一定利息的一种信用活动形式。根据存款对象或性质的不同可划分为企业存款、财政存款、机关团体存款、基本建设存款、储蓄存款、农村存款、委托存款、其他存款等科目。它是银行信贷资金的主要来源。

贷款　指银行或其他信贷机构根据资金必须归还的原则，按一定利率，为企业、个人等提供资金的一种信用活动形式。我国银行贷款分为短期贷款、中期流动资金贷款、中长期贷款、信托贷款、融资租赁、委托贷款、票据融资、各项垫款等。

承保额　又叫保险金额，指保险人承担赔偿或者给付保险金责任的最高限额。

保费　指投保人为取得保险人在约定范围内所承担赔偿责任而支付给保险人的费用。

Explanatory Notes on Main Statistical Indicators

Government Revenue　refers to the revenue of the government finance by means of participating in the distribution of the social products，which is the financial resources for ensuring the government to function. The contents of government revenue have been changed several times. Now it includes the following main items：

（1）Various tax revenues，including value added tax，business tax，enterprise income tax，personal income tax，resources tax，fixed assets investment direction regulating tax，tax on city maintenance and construction，real estate tax，stamp tax，tax on use of urban land，land value added tax，vehicle and vessel tax，tax on occupancy of cultivated land，property tax，tobacco leaf tax，and other tax revenues.

（2）Non-tax Revenues including special revenues，revenues from Administrative and institutional fees，penalty and confiscatory revenues，revenues from state-owned capital operationg，revenues from paid use of state-owned resources，and other revenues.

Government Expenditure refers to the distribution and use of the funds the government finance has raised，so as to meet the needs of economic construction and various causes. It includes the following main items：

（1）Expenditure for general public services：It reflects the expenditure from the government for general public services.

（2）Expenditure on public security：It reflects the expenditure from the government towards safeguarding the public security，including the related affairs of armed police，public security，state security，procuratorial administration，law court，judicial administration，jail，reeducation through labor，state confidentiality，anti-smuggling Patrol，etc.

（2）Expenditure on education：It reflects the expenditure from the government on education，including the related affairs of educational administration management，preschool education，primary education，junior secondary educate，regular senior secondary educate，regular higher education，primary vocational education，specialized secondary educate，technical educate，vocational senior secondary educate，vocational higher education，radio and television education，foreign student educate，special education，cadre continuing education，education institution services，etc.

（4）Expenditure on science and technology：It reflects the expenditure from the government on science and technology.

（5）Expenditure on culture，sport and media：It reflects the expenditure from the government on culture，cultural relics，sport，radio and television，publication，etc.

（6）Expenditure on social security and employment：It reflects the expenditure from the government on social security and employment，including the related affairs of management of social security and employment，civil administration，subsidies to social insurance funds，supplement to national social security funds，retirees of government agencies and institutions，subsidies to enterprises reform，subsidies to employment，pension，settling down demobilized servicemen，social security，disabled person administration，minimum living allowance in urban area，other social relief in urban area，social relief in rural area，subsidies to natural disaster，Red Cross business，etc.

（7）Expenditure on health care：It reflects the expenditure from the government on health care，including expenditure on management of health care，medical services，medical security，disease control and prevention，public health supervision，rural health care，etc.

（8）Expenditure on environment protection：It reflects the expenditure from the government on environment protection，including expenditure on management of environment protection，environment monitoring and supervisory，pollution government，natural ecological protection，project of natural forest protection，returning farmland to forest，sandstorm and wilderness government，returning grazing land to grassland，returning cultivated grassland to grassland，etc.

（9）Expenditure on urban and rural community affairs：It reflects the expenditure from the government on urban and rural community affairs，including expenditure on management of urban and rural community affairs，plan and management of urban and rural community，public utility of urban and rural community，residential buildings of urban and rural community，environmental sanitation of urban and rural community，management and supervision of markets construction，etc.

（10）Expenditure on agriculture，forest and irrigation：It reflects the expenditure from the government on agriculture，forest and irrigation，including expenditure on agriculture，forest，irrigation，poverty alleviation，comprehensive development of agriculture，etc.

Deposit is a form of credit by which enterprises，institutions，organizations or households can put money into banks and other credit institutions for safekeeping and interest earning under the principle of free withdrawal. According to different depositors，deposits are divided into enterprise deposits，treasury deposits，deposits of government agencies and organizations，capital construction deposits，savings deposits，rural saving deposits，entrusted deposits and other deposits. Deposits are major sources of the credit funds

of banks.

Loan is a form of credit by which banks and other credit institutions provide funds at certain interest rate to enterprises and individuals in the light of the principle of unconditional repayment. Loans from Chinese banks include circulating capital loans，fixed assets loans，loans to urban and rural individuals engaged in industrial and commercial business and agricultural loans.

Amount Covered which also is known as amount Insured， refers to the maximum that the insurant will get for the claim of the case insured.

Premium is the fee paid by the insurant to the insurer to obtain the obligation of compensation from the insurance within the agreed terms.

价格指数 9

PRICE INDEXES

简 要 说 明

一、本篇资料的主要内容

本篇资料主要包括工业生产者出厂、工业生产者购进、固定资产投资、房地产、居民消费、商品零售等价格指数。

二、本篇资料的来源

1、工业生产者出厂、工业生产者购进、固定资产投资、房地产价格指数分别来源于生产、房地产价格统计调查年报，由国家统计局青岛调查队生产生产价格调查处整理提供。

2、居民消费、商品零售价格指数来源于消费价格统计调查年报，由国家统计局青岛调查队消费价格调查处整理提供。

Brief Introduction

I. Main Content

Data in this chapter mainly include producer price indices for manufactured goods, purchasing price indices for industrial producers, price indices of investment in fixed assets, real estate price indices, consumer price indices and retail price indices.

II. Source of Data

（1）Data on producer price indices for manufactured goods, purchasing price indices for industrial producers, price indices of investment in fixed assets, real estate price indices are based on annual report of price survey on production, investment, real estate, and provided by the Division of Production Price Survey of Survey Office of the National Bureau of Statistics in Qingdao.

（2）Data on consumer price indices and retail price indices are based on annual report of consumer price survey, and provided by the Division of Consumer Price Survey of Survey Office of the National Bureau of Statistics in Qingdao.

9-1 主要年份居民消费和商品零售价格指数
MAJOR YEAR'S CONSUMER AND RETAIL PRICE INDEXES

（上年价格 =100） （perceding year=100）

年份 Year	居民消费价格指数 Consumer Price Index	# 食品类 Food	# 衣着类 Clothing	# 服务项目价格指数 Services Price Index	商品零售价格指数 Retail Price Index
1951	110.5	105.2	112.4		110.5
1952	99.8	99.6	98.7		99.8
1957	101.4	101.3	101.4	99.3	101.4
1962	102.6	102.0	100.2	99.9	102.6
1965	101.2	101.3	98.8	96.9	101.2
1970	98.9	99.7	100.0	99.5	98.9
1975	100.3	100.1	100.0	100.0	100.3
1978	100.5	100.2	99.8	100.0	100.5
1980	105.2	108.5	100.3	100.6	105.5
1985	110.4	115.2	102.3	102.7	110.9
1988	120.7	125.2	117.3	118.3	120.9
1990	104.5	103.4	103.4	118.8	103.6
1991	107.0	107.0	112.1	109.6	106.8
1992	111.4	115.1	108.6	119.9	110.8
1993	123.9	120.9	119.0	158.5	119.8
1994	126.9	135.2	130.1	117.9	122.8
1995	116.2	118.1	122.8	123.2	114.2
1996	112.6	110.1	104.3	130.4	106.0
1997	104.0	101.2	102.3	120.8	100.2
1998	100.0	93.7	94.5	136.2	94.9
1999	100.2	96.7	97.1	115.6	96.6
2000	103.3	100.0	108.3	112.0	99.7
2001	101.0	100.6	98.0	108.7	98.9
2002	98.9	97.8	100.2	101.0	99.5
2003	101.4	104.3	98.4	101.7	98.3
2004	102.1	104.0	103.6	104.7	99.0
2005	102.3	102.1	108.3	101.4	99.3
2006	100.9	101.7	99.4	100.8	99.7
2007	104.5	111.6	99.4	102.4	102.7
2008	104.7	111.7	103.9	99.8	103.9
2009	100.5	101.6	101.4	100.6	98.6
2010	102.2	106.4	101.0	100.4	101.4
2011	105.0	111.1	108.2	103.1	104.5
2012	102.7	104.3	106.3	102.1	101.7
2013	102.5	105.3	104.9	100.7	101.4
2014	102.6	104.4	103.2	101.9	102.3
2015	101.2	101.7	102.8	101.3	100.0
2016	102.5	105.0	100.3	101.8	102.0
2017	102.0	99.6	100.5	103.4	100.8
2018	102.1	103.1	103.8	101.6	101.8

注：1.2016 年起，食品类统计口径有变化，不包括“茶及饮料”和“在外餐饮”两项内容。
2.2016 年起，统计分类“服务项目价格指数”改为“服务价格指数”，增加“养老服务”和“金融服务”两项内容。

Note：1. Statistical caliber of food category has changed since 2016.The two sections of Tea and Drinks and Catering Service are not included.
2. The statistical category of “Price Index of Service Item” has been revised to “Price Index of Service” as of 2016 with two additional sections of “Elderiy Care Service” and “Financial Service”.

9-2 主要年份居民消费和商品零售价格指数（以 1950 年价格为 100）

MAJOR YEAR'S CONSUMER AND RETAIL PRICE INDEXES（1950=100）

年份<br>Year	居民消费价格指数<br>Consumer Price Index	#食品类<br>Food	#衣着类<br>Clothing	#服务项目价格指数<br>Services Price Index	商品零售价格指数<br>Retail Price Index
1951	110.5	105.2	112.4		110.5
1952	110.3	104.8	110.9		110.3
1957	119.5	120.7	115.3	91.6	119.5
1962	127.4	128.8	115.1	94.2	127.4
1965	133.3	138.7	108.4	81.5	133.3
1970	129.0	137.6	110.3	76.8	129.0
1975	128.8	138.2	110.0	75.7	128.8
1978	129.1	138.7	109.8	75.7	129.1
1980	137.6	154.1	109.3	76.1	138.1
1985	160.8	196.1	103.8	80.5	162.5
1988	223.8	295.2	133.2	100.8	227.6
1990	269.7	333.3	166.8	155.9	269.7
1991	288.6	356.7	187.0	170.9	288.1
1992	321.5	410.5	203.1	204.9	319.2
1993	398.3	496.3	241.7	324.8	382.4
1994	505.4	671.1	314.4	382.9	469.5
1995	587.3	792.5	386.1	471.8	536.2
1996	661.3	872.6	402.7	615.2	568.4
1997	687.8	883.0	412.0	743.1	569.5
1998	687.8	827.4	389.3	1012.1	540.5
1999	689.2	800.1	378.0	1170.0	522.1
2000	711.9	800.1	409.4	1310.4	520.5
2001	719.0	804.9	401.2	1424.4	514.8
2002	711.1	787.2	402.0	1438.7	512.2
2003	721.1	821.0	395.6	1463.1	503.5
2004	736.2	853.9	409.8	1531.9	498.5
2005	753.1	871.8	443.8	1553.4	495.0
2006	759.9	886.6	441.2	1565.8	493.5
2007	794.1	989.5	438.5	1603.4	506.8
2008	831.4	1105.3	455.6	1600.2	526.6
2009	835.6	1123.0	462.0	1609.8	519.2
2010	854.0	1194.9	466.6	1616.2	526.5
2011	896.7	1327.5	504.9	1666.3	550.2
2012	920.9	1384.6	536.7	1701.3	559.6
2013	943.9	1458.0	563.0	1713.2	567.4
2014	968.4	1522.2	581.0	1745.8	580.5
2015	980.0	1548.1	597.3	1768.5	580.5
2016	1004.5	1625.5	599.1	1800.3	592.1
2017	1024.6	1619.0	602.1	1861.5	596.8
2018	1046.1	1669.2	625.0	1891.3	607.6

注：1.2016 年起，食品类统计口径有变化，不包括“茶及饮料”和“在外餐饮”两项内容。
2.2016 年起，统计分类“服务项目价格指数”改为“服务价格指数”，增加“养老服务”和“金融服务”两项内容。

Note：1. Statistical caliber of food category has changed since 2016.The two sections of Tea and Drinks and Catering Service are not included.
2. The statistical category of “Price Index of Service Item” has been revised to “Price Index of Service” as of 2016 with two additional sections of “Elderiy Care Service” and “Financial Service”.

9-3 主要年份生产投资价格指数

MAJOR YEAR'S PRICE INDEXES FOR PRODUCTION AND INVESTMENT

（上年价格 =100） （perceding year=100）

年份 Year	工业生产者出厂价格指数 Producer Price Indexes for Industrial Producers	工业生产者购进价格指数 Purchasung Price Indexes for Industrial Producers	房屋销售价格指数 Sales Price Indexes of Houses	房屋租赁价格指数 Renting Price Indexes of Houses	物业管理价格指数 Property Managerment Price Indexes	固定资产投资价格指数 Price Indexes for Investment in Fixed Assets
1990	104.33	108.64				
1991	101.68	108.10				
1992	105.46	107.13				
1993	115.49	126.91				
1994	121.62	120.30				
1995	112.70	114.25				
1996	103.81	103.25				
1997	100.22	101.93				
1998	94.96	92.77	100.1	94.0		
1999	96.93	95.88	103.7	104.3		
2000	101.89	106.81	102.3	95.8		
2001	98.00	97.81	104.1	107.0		
2002	97.37	96.78	107.6	94.4		
2003	100.97	106.51	114.6	99.4		
2004	102.92	113.62	115.2	98.6		105.8
2005	101.39	106.52	110.9	103.3	100.2	103.1
2006	101.08	105.80	106.9	110.1	99.8	102.6
2007	101.50	106.69	106.5	108.3	100.4	104.1
2008	105.33	115.91	105.1	106.5	102.1	111.0
2009	95.78	90.35	100.3	103.5	100.4	94.6
2010	103.75	112.21		103.0	100.5	104.9
2011	104.90	109.39				107.0
2012	98.61	97.00				100.0
2013	98.82	96.53				100.1
2014	99.20	97.40				99.9
2015	97.04	94.24				97.1
2016	98.77	97.28				99.8
2017	104.40	110.73				107.3
2018	104.22	105.22				106.6

注：2010 年之前工业生产者出厂价格为工业品出厂价格；工业生产者购进价格为主要原材料、燃料、动力购进价格。

Note：Producer 's Prices at works were the prices of industrial products at words before 2010；Producer 's purchasing price refers to the purchasing price of main raw materials，fuels and power.

9-4　工业生产者出厂价格指数
PRODICER PRICE INDEXES FOR INDUSTRIAL PRODUCERS

（上年价格 =100）

类别	Item	2000 年	2005 年	2008 年	2009 年
**总指数**	**General Index**	**101.89**	**101.39**	**105.33**	**95.78**
其中：轻工业	Light Industry	98.50	99.78	102.93	97.21
重工业	Heavy Industry	105.62	104.32	109.17	93.45
**其中：生产资料**	**Means of Production**	**105.03**	**103.24**	**108.76**	**94.25**
采掘	Excavation	95.67	114.74	115.24	93.44
原料	Raw Materials	113.82	106.91	108.81	90.67
加工	Processing	97.58	101.45	108.59	95.34
**生活资料**	**Means of Livelihood**	**98.25**	**99.13**	**101.72**	**97.45**
食品	Food	96.59	98.80	108.42	98.21
衣着	Clothing	99.37	101.48	103.36	102.03
一般日用品	Articles for Daily Use	104.52	101.97	103.03	99.39
耐用消费品	Articles for Daily Use	97.70	97.43	97.13	94.52

（perceding year=100）

2010 年	2011 年	2012 年	2013 年	2014 年	2015 年	2016 年	2017 年	2018 年
**103.75**	**104.90**	**98.61**	**98.82**	**99.20**	**97.04**	**98.77**	**104.40**	**104.22**
100.80	103.46	99.70	99.11	99.62	98.74	99.70	101.13	101.27
107.57	105.89	97.87	98.62	98.91	98.79	97.87	106.46	106.01
**106.43**	**105.54**	**97.55**	**98.58**	**98.87**	**95.23**	**98.60**	**106.34**	**105.86**
130.94	132.07	93.35	91.72	100.65	95.12			
115.24	111.52	99.05	99.26	98.87	88.59	93.92	112.92	114.50
103.50	103.49	97.20	98.50	98.84	97.06	99.60	104.96	103.95
**99.61**	**103.82**	**100.42**	**99.21**	**99.76**	**100.07**	**99.12**	**100.69**	**100.95**
105.06	109.71	99.91	95.34	98.34	102.53	102.59	100.55	100.55
101.56	107.44	102.03	102.74	100.34	100.39	100.93	101.54	100.14
99.96	108.02	101.64	101.42	101.01	99.59	100.12	101.63	101.54
93.23	94.34	98.79	98.41	99.65	98.26	94.58	99.78	101.32

9-5 工业生产者购进价格指数

PURCHASING PRICE INDEXES FOR INDUSTRIAL PRODUCERS

（上年价格 =100）

类别	Item	2000 年	2005 年	2008 年	2009 年
**总指数**	**General Index**	**106.81**	**106.52**	**115.91**	**90.35**
燃料、动力类	fuel ang Power	113.82	117.34	126.19	90.53
黑色金属材料类	Ferrous Metals	102.66	107.84	122.19	82.38
#钢材	Rolled-steel	105.44	106.57	119.10	85.25
其他	Others	101.1	112.88	132.40	72.73
有色金属材料及电线类	Nonferrous Metals and Electric Wire	106.75	108.06	93.41	86.24
化工原料类	Raw Chemical Materials	106.9	112.47	123.00	87.66
木材及纸浆类	Timber and Paper Pulp	108.98	103.8	103.58	89.16
建筑材料及非金属矿类	Building Materials and Nonmetals	99.5	115.54	124.52	100.98
其他工业原材料及半成品	Other Industrial Raw Materials and Semi-fin-ished Goods	105.32	97.87	109.65	92.70
农副产品	Agricultural Products	92.73	101.55	117.68	88.60
纺织原料类	Textile Materials	104.03	98.15	101.44	98.69

(perceding year=100)

2010年	2011年	2012年	2013年	2014年	2015年	2016年	2017年	2018年
**112.21**	**109.39**	**97.00**	**96.53**	**97.40**	**94.24**	**97.28**	**110.73**	**105.22**
115.43	111.29	100.64	95.18	97.65	88.51	89.94	112.91	112.55
111.97	113.08	93.20	93.76	95.81	87.26	97.23	117.41	104.02
108.67	107.95	92.33	92.87	96.87	89.99	96.89	119.10	108.75
122.64	129.01	94.64	96.57	92.49	78.70	98.41	110.30	86.82
122.65	108.45	93.64	96.23	96.24	92.34	96.29	114.82	103.94
111.04	113.79	96.72	96.47	99.51	96.44	101.00	107.63	101.55
117.06	103.78	93.12	98.79	102.42	98.33	99.93	125.23	111.21
107.61	116.71	102.48	100.13	97.36	95.52	100.91	115.06	115.97
106.41	103.31	98.05	96.65	96.25	96.95	97.50	106.42	102.41
121.30	115.04	98.21	98.54	97.62	99.75	101.53	105.92	99.70
112.90	111.11	95.61	99.60	97.38	95.86	99.81	110.09	105.80

9-6 按工业行业分工业生产者出厂价格指数
PRODUCER PRICE INDEXES FOR INDUSTRIAL PRODUCERS BY SECTOR

（上年价格 =100）

行业	Sector
**工业生产者出厂价格指数**	**Producer Price Indexes for Manufactured Manufactured Goods**
黑色金属矿采选业	Mining of Ferrous Metal Ores
有色金属矿采选业	ining of Non-ferrous Metal Ores
非金属矿采选业	Mining and Processing of Nonmetal Ores
农副食品加工业	Processing of Food from Agricultural Products
食品制造业	Manufacture of Foods
酒、饮料和精制茶制造业	Manufacturing Industry of Alcohol，Beverage and Refined Tea
纺织业	Manufacture of Textile
纺织服装、服饰业	Industry of Textile and Garment，and Apparel
皮革、毛皮、羽毛及其制品和制鞋业	Industry of Leather，Furs，Down and Related Products，and Shoes Making
木材加工和木、竹、藤、棕、草制品业	Processing of Timbers，Manufacture of Wood，Bamboo，Rattan，Palm，and Staw Products
家具制造业	Manufacture of Furniture
造纸和纸制品业	Manufacture of Paper and Paper Products
印刷和记录媒介复制业	Printing，Reproduction of Recording Media
文教、工美、体育和娱乐用品制造业	Industry of Culture and Education，Arts and Crafts，and Entertainment Products Manufacturing
石油加工、炼焦和核燃料加工业	Processing of Petroleum，Coking，Processing of Nucleus Fuel
化学原料和化学制品制造业	Manufacture of Chemical Raw Material and Chemical Products
医药制造业	Manufacture of Medicines
化学纤维制造业	Manufacture of Chemical Fiber
橡胶和塑料制品业	Industry of Rubber and Plastic Products
非金属矿物制品业	Manufacture of Non-metallic Mineral Products
黑色金属冶炼和压延加工业	Smelting and Pressing of Ferrous Metals
有色金属冶炼和压延加工业	Smelting and Pressing of Non-ferrous Metals
金属制品业	Manufacture of Metal Products
通用设备制造业	Manufacture of General Purpose Machinery
专用设备制造业	Manufacture of Special Purpose Machinery
汽车制造业	Vehicle Manufacturing Industry
铁路、船舶、航空航天和其他运输设备制造业	aufacturing Industry of Railroads. Vessels，Aerospace and Other Transporation Equipment
电气机械和器材制造业	Manufacture of Electrical Machinery&Equipment
计算机、通信和其他电子设备制造业	Computer. Communications and Other Electronic Equipment Manufacturing
仪器仪表制造业	Instruments Manufacturing Industry
其他制造业	Other Manufacturing Industry
金属制品、机械和设备修理业	Industry of Metal Products，and Repair of Machinery and Equipment
电力、热力生产和供应业	Production and Supply of Electric Power and Heat Power
燃气生产和供应业	Production and Supply of Gas
水的生产和供应业	Production and Supply of Water

注：2012 年国民经济行业分类调整，本表中 2011 年— 2018 年数据采用新国民经济行业分类 GB/T4754-2011。

Note：Classification of national economic industries was adjusted in 2012. The new natinoal economic industries classification by GB/T4754-2011 applies to data from 2011 to 2018 in this table.

（perceding year=100）

2011 年	2012 年	2013 年	2014 年	2015 年	2016 年	2017 年	2018 年
**104.90**	**98.61**	**98.82**	**99.20**	**97.04**	**98.77**	**104.40**	**104.22**
104.33	99.93	100.00	99.86	100.00			
111.39	102.48	98.72	95.57	89.94			
145.13	89.33	87.06	102.14	94.85			
111.34	98.69	93.46	97.58	102.47	102.80	101.12	101.41
112.52	103.11	103.08	101.78	100.66	100.30	101.40	98.67
104.14	105.89	100.48	98.76	99.01	99.97	99.54	100.85
105.09	96.15	100.18	100.27	99.41	97.60	103.84	103.58
105.43	101.66	104.17	101.74	100.84	100.83	102.17	100.28
104.79	102.59	101.83	98.56	99.72	99.81	100.15	99.82
108.13	103.21	99.67	100.96	100.35			
101.11	102.00	100.02	100.00	99.91	98.46	98.72	100.48
105.71	95.59	96.13	101.96	99.78	99.58	115.83	104.86
110.06	107.10	108.65	96.97	95.96	98.45	101.11	102.77
100.61	101.47	100.47	100.00	99.96	99.88	101.90	100.54
114.95	104.26	99.49	97.38	76.03	91.60	114.48	121.44
114.60	89.61	98.04	99.70	96.53	95.20	119.17	113.72
107.33	95.05	105.08	127.56	100.73	104.83	101.06	100.95
123.72	92.56	95.65	96.69	90.31	89.56	117.03	98.53
114.68	99.67	95.40	95.50	94.93	97.60	100.62	100.10
108.19	98.39	99.82	100.91	101.62	99.08	114.14	120.19
111.00	86.47	93.52	94.45	83.44	99.13	117.16	108.23
108.26	89.14	93.87	94.43	87.95	95.29	125.17	102.35
99.79	99.82	104.14	96.62	96.59	103.64	106.15	105.34
101.39	100.07	103.87	101.02	100.37	98.78	103.45	104.80
101.69	101.26	99.99	99.81	99.72	98.36	100.00	101.23
99.27	98.97	98.59	102.49	101.04	101.46	100.93	99.83
99.17	99.66	97.65	100.96	100.03	99.29	100.16	99.79
103.10	98.40	98.82	99.24	98.61	98.96	102.82	102.36
84.65	95.43	94.31	98.88	96.31	90.16	102.17	100.06
102.01	98.72	98.01	98.43	97.05	100.00	100.09	100.15
95.16	98.52	99.94	100.12	99.89	100.87	104.20	105.84
102.17	98.43	88.75	91.69	90.21			
101.49	104.36	99.81	99.15	98.10	97.59	97.05	97.83
107.34	103.32	102.54	109.38	98.88	101.26	96.32	108.74
100.15	100.25	100.05	100.34	103.46	108.78	100.00	113.81

9-7 固定资产投资价格指数
PRICE INDEXES FOP INVESTMENT IN FIXED ASSETS

（上年价格 =100） （perceding year=100）

项目名称	Item	2005	2008	2009	2010	2011	2012	2013	2014	2015	2016	2017	2018
**总计**	**Total**	**103.1**	**111.0**	**94.6**	**104.9**	**107.0**	**100.0**	**100.1**	**99.9**	**97.1**	**99.8**	**107.3**	**106.6**
建筑安装、装饰工程	Construction and Installation Project	103.7	115.5	92.1	107.5	110.1	99.8	100.0	99.6	95.5	99.6	110.7	109.5
其中：人工费	Of which： Labor Cost	112.7	112.7	104.9	112.0	115.9	110.4	107.8	104.8	103.1	101.6	103.0	104.9
材料费	Materials Expenses	101.0	120.5	87.5	107.0	109.6	96.7	97.6	97.7	92	98.8	114.9	112.4
机械使用费	Expenses on Machinery Use	104.2	106.9	100.0	104.3	105.0	102.3	101.0	100.8	100.9	100.1	101.2	102.0
设备、工器具购置	Purchase of Equipment，Tools and Instruments	99.7	102.5	98.0	100.2	101.8	99.2	99.3	99.9	99.2	98.7	100.6	101.5
其他费用	Others	105.5	105.9	98.5	102.2	103.0	102.0	101.4	101.4	101.2	102.2	102.0	99.9

9-8 住宅销售价格指数（2018 年）
SALES PRICE INDEXES FOR RESIDENCE（2018）

（上年价格 =100） （perceding year=100）

项目	Item	2018
**新建住宅销售价格指数**	**Sales Price Indexes for Newly Constucted Residence**	
一月	January	103.6
二月	February	103.9
三月	March	103.3
四月	April	103.1
五月	May	104.8
六月	June	107.0
七月	July	108.8
八月	August	110.1
九月	September	110.4
十月	October	110.7
十一月	November	112.2
十二月	December	113.3
**二手住宅销售价格指数**	**Sales Price Indexes for Secong-hand Residence**	
一月	January	110.6
二月	February	108.0
三月	March	107.7
四月	April	107.7
五月	May	107.5
六月	June	107.2
七月	July	108.8
八月	August	110.4
九月	September	110.9
十月	October	111.4
十一月	November	111.9
十二月	December	111.2

9-9 居民消费价格分类指数（2018 年）
CONSUMER PRICE INDEXES BY CATEGORY（2018）

（上年价格 =100） （perceding year=100）

项目	Item	指数<br>Indexes
**居民消费价格总指数**	**Consumer Price Index**	**102.1**
非食品烟酒价格指数	Price index of non-food cigarettes and alcohol	101.8
服务价格指数	Price index of services	101.6
鲜活食品价格指数	Price index of fresh food	105.3
消费品价格指数	Consumer Goods Price Index	102.4
扣除鲜菜鲜果价格指数	Price Index Without Fersh Vegetables and Fresh Fruits	101.8
**一、食品烟酒**	**Food，cigarettes and liquor**	**102.9**
1. 食品	Food	103.1
（1）粮食	Grain	100.5
（2）薯类	Potatoes	120.9
（3）豆类	Beans	101.0
（4）食用油	Cooking Oil	99.7
（5）菜	Vegetables	112.9
（6）畜肉类	Livestock Products	97.3
（7）禽肉类	Poultry Products	106.5
（8）水产品	Aquatic Products	104.5
（9）蛋类	Eggs	111.4
（10）奶类	Dairy Products	100.7
（11）干鲜瓜果类	Dried and Fresh Melons and Fruits	102.1
（12）糖果糕点类	Candy and Pastry	99.4
（13）调味品	Flavoring	101.3
（14）其他食品类	Other foods	100.3
2. 茶及饮料	Tea and Beverages	98.9
3. 烟酒	Tobacco Liquor	100.8
4. 在外餐饮	Catering service	103.5

注：2016 年起，居民消费价格调查目录进行了调整，新调查目录与国际标准更为接近。

Note：Since 2016，the consumption price investigation directory has been adjusted，and the new investigation directory is basically consistent with the household consumption expenditure catalogue.

9-9 续表
Continued

项目	Item	指数<br>Indexes
**二、衣着**	**Clothing**	**103.8**
1. 服装	Garments	105.9
2. 服装材料	Clothing material	100.0
3. 其他衣着及配件	Other clothing an accessories	100.5
4. 衣着加工服务费	Service charge of clothing processing	100.4
5. 鞋类	Shoes	98.7
**三、居住**	**Residence**	**100.9**
1. 租赁房房租	Rents	100.6
2. 住房保养维修及管理	Maintenance and management of house	101.6
3. 水电燃料	Water，Electricity and Fuels	101.1
4. 自有住房	Private Housing	100.7
**四、生活用品及服务**	**Article of daily use and service**	**101.3**
1. 家具及室内装饰品	Furniture and interior decoration	103.2
2. 家用器具	Home appliances	99.4
3. 家用纺织品	Home textile	103.8
4. 家庭日用杂品	Daiy Use Household Articles	100.6
5. 个人护理用品	Personal-care supply	100.1
6. 家庭服务	Household Services	105.0
**五、交通和通信**	**Transportation and Communication**	**100.8**
1. 交通	Transportation	103.4
2. 通信	Communication	95.5
**六、教育文化和娱乐**	**Education，culture and entertainment**	**103.1**
1. 教育	Education	104.0
2. 文化娱乐	Cultural	102.3
**七、医疗保健**	**Health Care**	**102.4**
1. 药品及医疗器具	Medicine and medical apparatus	104.6
2. 医疗服务	Medical service	100.0
**八、其他用品和服务**	**Other articles and services**	**101.3**
1. 其他用品类	Other articles	97.3
2. 其他服务类	Other services	104.1

9-10 商品零售价格分类指数（2018 年）
RETAIL PRICE INDEXES BY CATEGORY（2018）

（上年 =100） （perceding year=100）

项目	Item	指数 Indexes
**商品零售价格总指数**	**Retail Price Indes**	**101.8**
**一、食品**	**Food**	**103.2**
1. 粮食	Grain	100.5
2. 薯类	Potatos	120.9
3. 豆类	Beans	101.0
4. 食用油	Edible oil	99.7
5. 菜	vegetables	112.9
6. 畜肉类	Stock rneat	97.3
7. 禽肉类	Poultiy meat	106.5
8. 水产品	Aquatic Products	104.5
9. 蛋类	Eggs	111.4
10. 奶类	Dairy	100.7
11. 干鲜瓜果类	Dried and Fresh Melon，and Fruits	102.1
12. 糖果糕点类	Sweets and cakes	99.4
13. 调味品	Flavoring	101.3
14. 其他食品类	Other food	100.3
15. 在外餐饮	Catering service	103.5
**二、饮料、烟酒**	**Beverages，Tobacco and Liquor**	**100.2**
1. 茶及饮料	Tea and Beverages	98.9
2. 烟草	Tobacco	100.0
3. 酒类	Liquor	101.6
**三、服装、鞋帽**	**Clothing**	**104.0**
1. 服装	Garments	106.0
2. 鞋帽袜	Footgear and Hats	98.8
3. 其他衣着配件	Other clothing and accessories	100.6
**四、纺织品**	**Textiles**	**104.3**
1. 服装材料	Clothing material	100.0
2. 床上用品	Blend Cloth	104.9
**五、家用电器及音像器材**	**Household Appliances，Music and Video Equipment**	**98.8**
1. 家庭设备	Household Facilities	99.4
2. 文娱用耐用消费品	Durable Consumer Goods for Cultural and Recreational Use and Services	97.3
3. 专业音像器材	Professional Audio and Video Equipment	98.7

注：2016 年起，商品零售价格调查目录进行了微调。
Note：Contents of survey of retailing price of commodities have been finely adjusted as of 2017.

9-10 续表
Continued

项目	Item	指数 Indexes
**六、文化办公用品**	**Culture and office Articles**	**100.7**
**七、日用品**	**Articles for Daily Use**	**100.4**
1. 日用百货	General Merchandise for Daily Use	101.7
2. 厨具餐具茶具	Kitchen ware，tableware，tea set	99.4
3. 清洗用品	Cleaning Products	100.0
4. 其他日用品	Other Articles for Daily Use	99.8
**八、体育娱乐用品**	**Sports and Recreation Articles**	**99.9**
1. 体育户外用品	Sports and outdoors	99.7
2. 娱乐用品	Recreation Articles	100.0
**九、交通、通信用品**	**Transportation and Communication Appliances**	**97.3**
1. 交通运输机械	Transportation Machines	99.0
2. 通信器材	Communication Equipment	88.7
**十、家具**	**Furniture**	**103.1**
**十一、化妆品**	**Cosmetics**	**100.2**
**十二、金银饰品**	**Gold，Silver and Jewelry**	**96.0**
**十三、中西药品及医疗保健用品**	**Traditional Chinese and Western Medicals and Health Care Articles**	**105.1**
1. 医疗卫生器具	Medical apparatus	100.1
2. 中药	Herbal medicine	109.0
3. 西药	Western Medicines	104.7
4. 保健器具及用品	Health Care Articles	101.8
**十四、书报杂志及电子出版物**	**Books，Newspapers，Magazines and Electronic Publications**	**102.5**
1. 教材及参考书	Teaching Material and Reference Books	102.3
2. 书报杂志	Books，Mewspapers and Magazines	101.9
3. 计算机办公软件	Computer office software	106.2
**十五、燃料**	**Fuels**	**109.7**
1. 煤炭及制品	Coal and Its Products	107.6
2. 石油及制品	Pertoleum and Its Products	109.8
**十六、建筑材料及五金电料**	**Building Materials and Hardware**	**100.7**
1. 建筑装璜材料	Building Decoration Materials	99.4
2. 五金水暖	Hardware	104.2

主要统计指标解释

居民消费价格指数　是反映一定时期内城乡居民所购买的生活消费品价格和服务项目价格变动趋势和程度的相对数，是对城市居民消费价格指数和农村居民消费价格指数进行综合汇总计算的结果。该指数可以观察和分析消费品的零售价格和服务价格变动对城乡居民实际生活费支出的影响程度。

商品零售价格指数　是反映一定时期内城乡商品零售价格变动趋势和程度的相对数。商品零售价格的变动直接影响到城乡居民的生活支出和国家的财政收入，影响居民购买力和市场供需的平衡，影响到消费与积累的比例关系。因此，该指数可以从一个侧面对上述经济活动进行观察和分析。

工业生产者出厂价格指数　是反映一定时期内全部工业产品出厂价格总水平的变动趋势和程度的相对数，包括工业企业售给本企业以外所有单位的各种产品和直接售给居民用于生活消费的产品。该指数可以观察出厂价格变动对工业总产值及增加值的影响。

工业生产者购进价格指数　是反映工业企业作为生产投入，而从物资交易市场和能源、原材料生产企业购买原材料、燃料和动力产品时，所支付的价格水平变动趋势和程度的统计指标，是扣除工业企业物质消耗成本中的价格变动影响的重要依据。

房地产价格指数　是反映一定时期内房地产价格变动趋势和程度的相对数，包括新建住宅销售价格指数、二手住宅销售价格指数。

Explanatory Notes on Main Statistical Indicators

Consumer Price Indices　reflect the trend and degree of changes in prices of consumer goods and services purchased by urban and rural households during a given period. They are obtained by combining the Urban Consumer Price Indices and the Rural Consumer Price Indices. The Indices enable the observation and analysis of the degree of impact of the changes in the prices of retailed goods and services on the actual living expenses of urban and rural residents.

Retail Price Indices　reflect the trend and degree of change in retail prices of commodities during a given period. The change in retail prices of commodities directly affect the living expenditure of urban and rural residents，government revenue，purchasing power of residents and the equilibrium of market supply and demand，and the ratio of consumption to accumulation. Therefore，the retail price indices are useful to analyze the changes of the above economic activities.

Producer Price Indices for Industrial Producers　reflect the trend and degree of changes in general ex-factory prices of all industrial products during a given period，including sales of industrial products by an industrial enterprise to all units outside the enterprise，as well as sales of consumer goods to residents. It can be used to analyze the impact of ex-factory prices on gross output value and value-added of the industrial sector.

Purchasing Price Indices for Industrial Producers　reflect changes in the level and degree of prices paid by industrial enterprises when they purchase production input such as raw materials，fuels and power from the market or from other energy or raw

materials producing enterprises. These indices provide important basis for measuring the material consumption of industrial enterprises after removing influence of price changes.

Price Indices for Real Estate reflect the trend and degree of changes in prices of real estate during a given period，including sales price indices for newly constructed residential buildings and secondhand residential buildings.

人民生活 10

PEOPLE'S LIVING CONDITIONS

简要说明

一、本篇资料的主要内容

本篇资料主要反映了全市居民的家庭收支、居住、家庭消费构成、耐用消费品拥有量等方面的情况。

二、本篇资料的来源

本篇资料中相关数据来源于居民收支调查年报，由国家统计局青岛调查队住户调查处整理提供。

Brief Introduction

I. Main Content

This section mainly reflects the residents' household income and expenditure, housing, household consumption structure, quantity of durable consumer goods possessed and other information.

II. Source of Data

The relevant data in this material comes from the annual report of the residents' income and expenditure survey, which is provided by the Investigation Team of the Investigation Team of the National Bureau of Statistics.

10-1 城市居民收支（1978-2014 年）
INCOME AND EXPENDITURE DF URBAN RESIDENTS（1978-2014）

年份<br>Year	城市居民人均可支配收入（元）<br>Aunual Per Capita Disposable Income of Urban Households（yuan）	城市居民人均消费性支出（元）<br>Per Capita Consumption Expenditure of Urban Households（yuan）	城市恩格尔系数（%）<br>Engel coefficient（%）
1978	336	321	
1979	380	360	
1980	537	495	
1981	500	490	57.8
1982	528	484	61.6
1983	549	510	63.7
1984	665	580	62.8
1985	733	681	58.6
1986	882	806	59.2
1987	1089	996	58.1
1988	1225	1278	55.9
1989	1455	1439	57.8
1990	1624	1507	58.4
1991	1856	1767	57.4
1992	2138	1956	57.0
1993	2668	2445	51.8
1994	3880	3479	53.2
1995	5357	4606	52.5
1996	5602	5079	51.8
1997	6222	5525	48.2
1998	6554	5565	46.4
1999	7282	5981	44.5
2000	8016	6677	42.5
2001	8731	6849	41.9
2002	8721	7344	40.5
2003	10075	8056	39.3
2004	11089	9002	38.0
2005	12920	9883	37.6
2006	15328	11945	36.4
2007	17856	13376	37.0
2008	20464	14999	37.4
2009	22368	16080	38.5
2010	24998	17531	37.4
2011	28567	19297	37.5
2012	32145	20391	36.5
2013	35227	22060	36.5
2014	38294	24016	35.7

注：2002 年开始城市居民可支配收入为新计算口径。

Note：From 2002 on，disposable income of urban residents are counted by new gauges.

10-2 农村居民收支（1978-2014 年）
INCOME AND EXPENDITURE DF RURAL RESIDENTS（1978-2014）

年份 Year	农民人均纯收入（元） Aunual Per Capita Net Income of Rural Households（yuan）	农村居民人均生活消费支出（元） Per Capita Consumption Expenditure of Rural Households（yuan）	农村恩格尔系数（%） Engel coefficient（%）
1978	146	109	59.3
1979	153	129	58.4
1980	209	142	54.9
1981	218	163	44.3
1982	273	199	52.9
1983	399	277	52.8
1984	452	304	50.0
1985	567	376	47.8
1986	647	474	46.4
1987	730	462	42.9
1988	820	439	38.8
1989	876	510	38.9
1990	952	494	41.5
1991	1021	546	41.3
1992	1029	572	41.7
1993	1255	743	42.7
1994	1694	1384	54.5
1995	2225	1641	55.7
1996	2625	2173	50.5
1997	2599	2150	52.9
1998	3177	2119	49.5
1999	3415	2150	47.3
2000	3637	2380	43.2
2001	3901	2618	41.2
2002	4195	2820	38.9
2003	4530	2988	38.0
2004	5080	3353	38.7
2005	5806	3737	37.5
2006	6546	4203	36.6
2007	7477	4736	37.2
2008	8509	5303	37.7
2009	9249	5832	36.4
2010	10550	6662	35.5
2011	12370	7661	36.6
2012	13990	8653	36.2
2013	15731	9786	34.9
2014	17461	10808	33.8

10-3 城乡居民住房面积（1990-2014 年）
HOUSING AREA OF URBAN AND RUREL RESIDENTS（1990-2014）

年份<br>Year	城市居民人均现住房建筑面积（平方米）<br>Per Capita Home Floor Area of Urban Residents（square meter）	农村居民人均居住面积（平方米）<br>Per Capita Housing Area of Rural Residents（square meter）
1990	14.16	19.60
1995	17.12	23.03
2000	20.76	25.88
2001	21.86	26.59
2002	22.76	27.00
2003	23.73	27.00
2004	24.22	27.99
2005	22.96	29.54
2006	23.73	30.82
2007	23.72	30.95
2008	26.83	30.73
2009	26.71	31.39
2010	27.42	30.97
2011	27.67	31.73
2012	27.86	32.32
2013	29.12	33.50
2014	29.80	34.20

10-4 全体居民家庭基本情况（2015年）
BASIC INFORMATION ON ALL HOUSEHOLDS（2015）

指标名称	Name	单位	Unit	2015	2014	2015年比2014年增长（±%）<br>2015/2014（±%）
**人均可支配收入**	**Per Capita Disposable Income**	**元**	**yuan**	**32885**	**30273**	**8.6**
（一）工资性收入	Income from Wages and Salaries	元	yuan	20068	18348	9.4
（二）经营净收入	Net Income from Household Operations	元	yuan	6480	6035	7.4
（三）财产净收入	Income from Properties	元	yuan	2522	2396	5.2
（四）转移净收入	Income from Transfers	元	yuan	3815	3494	9.2
**人均消费支出**	**Per Capita Consumption Expenditure**	**元**	**yuan**	**21326**	**19653**	**8.5**
（一）食品烟酒	Food，Tobacco and Iquor	元	yuan	6459	5970	8.2
（二）衣着	Cloth	元	yuan	2096	1944	7.8
（三）居住	Residence	元	yuan	4584	4316	6.2
（四）生活用品及服务	Household Facilities，Articles and Services	元	yuan	1485	1374	8.1
（五）交通通信	Transportation and Communication	元	yuan	3226	2905	11.0
（六）教育文化娱乐	Education，Cultural and Recreation Services	元	yuan	1830	1654	10.6
（七）医疗保健	Health Care and Medical Services	元	yuan	1115	1002	11.2
（八）其他用品和服务	Other Goods and Services	元	yuan	531	488	8.8
**城镇居民人均现住房建筑面积**	**Per Capita Building Space in Urban Areas**	**平方米**	**sq.m**	**31.5**		
**农村居民人均现住房建筑面积**	**Per Capita Living Space in Rural Areas**	**平方米**	**sq.m**	**32.4**		

注：自2015年开始，全市城乡住户调查统一使用一体化改革后的数据，与原数据相比，城乡居民收支指标的调查范围和口径均存较大变化。原“城市居民人均可支配收入”调整为“城镇居民人均可支配收入”；原“农民人均纯收入”调整为“农村居民人均可支配收入”。2014年数据也为新口径，与2015年年鉴数据不一致。2014年农村居民住房为拥有面积，2015年为现住房建筑面积。

Note：From 2015 on，the data used are statistics of the integrated survey of residents in urban and rural areas. Compared with previous data，there is a significant change in statistic coverage and gauge for indicators of urban and rural residents’ income and expenditure. The ‘per capita disposable income of residents in cities was changed to’ per capita disposable income of urban residents’；the ‘pe capita net income of farmers’ was changed to ‘per capita disposable income of rural residents. Data in 2014 are counted by new gauge and they are different from the data in last yrar’s yearbook. As for the statistics of rural resident homes，the owned house area was taken in 2014，but in 2015，the construction area was counted.

10-5 全体居民家庭基本情况（2016 年）
BASIC INFORMATION ON ALL HOUSEHOLDS（2016）

指标名称	Name	单位	Unit	2016	2015	2016 年比 2015 年增长（±%）2016/2015（±%）
**人均可支配收入**	**Per Capita Disposable Income**	**元**	**yuan**	**35680**	**32885**	**8.5**
（一）工资性收入	Income from Wages and Salaries	元	yuan	21700	20068	8.1
（二）经营净收入	Net Income from Household Operations	元	yuan	7011	6480	8.2
（三）财产净收入	Income from Properties	元	yuan	2781	2522	10.3
（四）转移净收入	Income from Transfers	元	yuan	4188	3815	9.8
**人均消费支出**	**Per Capita Consumption Expenditure**	**元**	**yuan**	**23256**	**21326**	**9.0**
（一）食品烟酒	Food，Tobacco and Iquor	元	yuan	6983	6459	8.1
（二）衣着	Cloth	元	yuan	2271	2096	8.4
（三）居住	Residence	元	yuan	5018	4584	9.5
（四）生活用品及服务	Household Facilities，Articles and Services	元	yuan	1607	1485	8.2
（五）交通通信	Transportation and Communication	元	yuan	3479	3226	7.8
（六）教育文化娱乐	Education，Cultural and Recreation Services	元	yuan	2075	1830	13.4
（七）医疗保健	Health Care and Medical Services	元	yuan	1233	1115	10.6
（八）其他用品和服务	Other Goods and Services	元	yuan	590	531	11.2
**城镇居民人均现住房建筑面积**	**Per Capita Building Space in Urban Areas**	**平方米**	**sq.m**	**31.7**	**31.5**	**0.6**
**农村居民人均现住房建筑面积**	**Per Capita Living Space in Rural Areas**	**平方米**	**sq.m**	**33.2**	**32.4**	**2.5**

10-6 全体居民家庭基本情况（2017 年）
BASIC INFORMATION ON ALL HOUSEHOLDS（2017）

指标名称	Name	单位	Unit	2017	2016	2017 年比 2016 年增长（±%）2017/2016（±%）
**人均可支配收入**	**Per Capita Disposable Income**	**元**	**yuan**	**38763**	**35680**	**8.6**
（一）工资性收入	Income from Wages and Salaries	元	yuan	23514	21700	8.4
（二）经营净收入	Net Income from Household Operations	元	yuan	7485	7011	6.8
（三）财产净收入	Income from Properties	元	yuan	3082	2781	10.8
（四）转移净收入	Income from Transfers	元	yuan	4682	4188	11.8
**人均消费支出**	**Per Capita Consumption Expenditure**	**元**	**yuan**	**25232**	**23256**	**8.5**
（一）食品烟酒	Food，Tobacco and Iquor	元	yuan	7489	6983	7.2
（二）衣着	Cloth	元	yuan	2467	2271	8.6
（三）居住	Residence	元	yuan	5454	5018	8.7
（四）生活用品及服务	Household Facilities，Articles and Services	元	yuan	1747	1607	8.7
（五）交通通信	Transportation and Communication	元	yuan	3753	3479	7.9
（六）教育文化娱乐	Education，Cultural and Recreation Services	元	yuan	2347	2075	13.1
（七）医疗保健	Health Care and Medical Services	元	yuan	1350	1233	9.5
（八）其他用品和服务	Other Goods and Services	元	yuan	625	590	6.0
**城镇居民人均现住房建筑面积**	**Per Capita Building Space in Urban Areas**	**平方米**	**sq.m**	**32.7**	**31.7**	**3.2**
**农村居民人均现住房建筑面积**	**Per Capita Living Space in Rural Areas**	**平方米**	**sq.m**	**34.8**	**33.2**	**4.8**

10-7 全体居民家庭基本情况（2018 年）
BASIC INFORMATION ON ALL HOUSEHOLDS（2018）

指标名称	Name	单位	Unit	2018	2017	2018 年比 2017 年增长（±%）<br>2018/2017（±%）
**人均可支配收入**	**Per Capita Disposable Income**	**元**	**yuan**	**42019**	**38763**	**8.4**
（一）工资性收入	Income from Wages and Salaries	元	yuan	25303	23514	7.6
（二）经营净收入	Net Income from Household Operations	元	yuan	8027	7485	7.3
（三）财产净收入	Income from Properties	元	yuan	3475	3082	12.8
（四）转移净收入	Income from Transfers	元	yuan	5214	4682	11.3
**人均消费支出**	**Per Capita Consumption Expenditure**	**元**	**yuan**	**27316**	**25232**	**8.3**
（一）食品烟酒	Food，Tobacco and Iquor	元	yuan	7787	7489	4.0
（二）衣着	Cloth	元	yuan	2538	2467	2.9
（三）居住	Residence	元	yuan	6365	5454	16.7
（四）生活用品及服务	Household Facilities，Articles and Services	元	yuan	1837	1747	5.1
（五）交通通信	Transportation and Communication	元	yuan	4084	3753	8.8
（六）教育文化娱乐	Education，Cultural and Recreation Services	元	yuan	2571	2347	9.5
（七）医疗保健	Health Care and Medical Services	元	yuan	1480	1350	9.6
（八）其他用品和服务	Other Goods and Services	元	yuan	654	625	4.6
**城镇居民人均现住房建筑面积**	**Per Capita Building Space in Urban Areas**	**平方米**	**sq.m**	**32.9**	**32.7**	**0.8**
**农村居民人均现住房建筑面积**	**Per Capita Living Space in Rural Areas**	**平方米**	**sq.m**	**35.9**	**34.8**	**3.1**

10-8 城镇居民家庭基本情况（2015 年）
BASIC CONDITIONS OF URBAN HOUSEHOLDS（2015）

指标名称	Name	单位	Unit	2015	2014	2015 年比 2014 年增长（±%）<br>2015/2014（±%）
**人均可支配收入**	**Per Capita Disposable Income**	**元**	**yuan**	**40370**	**37346**	**8.1**
（一）工资性收入	Income from Wages and Salaries	元	yuan	25040	23034	8.7
（二）经营净收入	Net Income from Household Operations	元	yuan	6277	5818	7.9
（三）财产净收入	Income from Properties	元	yuan	3580	3436	4.2
（四）转移净收入	Income from Transfers	元	yuan	5473	5058	8.2
**人均消费支出**	**Per Capita Consumption Expenditure**	**元**	**yuan**	**26052**	**24122**	**8.0**
（一）食品烟酒	Food，Tobacco and Iquor	元	yuan	7856	7295	7.7
（二）衣着	Cloth	元	yuan	2685	2497	7.5
（三）居住	Residence	元	yuan	5607	5302	5.8
（四）生活用品及服务	Household Facilities，Articles and Services	元	yuan	1819	1686	7.9
（五）交通通信	Transportation and Communication	元	yuan	3810	3455	10.3
（六）教育文化娱乐	Education，Cultural and Recreation Services	元	yuan	2264	2057	10.0
（七）医疗保健	Health Care and Medical Services	元	yuan	1352	1227	10.2
（八）其他用品和服务	Other Goods and Services	元	yuan	659	603	9.3

注：本表数据为新口径，2014 年数据与 2015 年年鉴数据不一致。
Note：The data in this sheet is in line with new statistic caliber，which leads to the inconsistency between the data of 2014 and of 2015.

10-9 城镇居民家庭基本情况（2016 年）
BASIC CONDITIONS OF URBAN HOUSEHOLDS（2016）

指标名称	Name	单位	Unit	2016	2015	2016 年比 2015 年增长（±%）<br>2016/2015（±%）
**人均可支配收入**	**Per Capita Disposable Income**	**元**	**yuan**	**43598**	**40370**	**8.0**
（一）工资性收入	Income from Wages and Salaries	元	yuan	26898	25040	7.4
（二）经营净收入	Net Income from Household Operations	元	yuan	6846	6277	9.1
（三）财产净收入	Income from Properties	元	yuan	3909	3580	9.2
（四）转移净收入	Income from Transfers	元	yuan	5945	5473	8.6
**人均消费支出**	**Per Capita Consumption Expenditure**	**元**	**yuan**	**28285**	**26052**	**8.6**
（一）食品烟酒	Food，Tobacco and Iquor	元	yuan	8473	7856	7.9
（二）衣着	Cloth	元	yuan	2882	2685	7.4
（三）居住	Residence	元	yuan	6105	5607	8.9
（四）生活用品及服务	Household Facilities，Articles and Services	元	yuan	1962	1819	7.8
（五）交通通信	Transportation and Communication	元	yuan	4089	3810	7.3
（六）教育文化娱乐	Education，Cultural and Recreation Services	元	yuan	2553	2264	12.8
（七）医疗保健	Health Care and Medical Services	元	yuan	1490	1352	10.2
（八）其他用品和服务	Other Goods and Services	元	yuan	731	659	10.8

10-10 城镇居民家庭基本情况（2017 年）
BASIC CONDITIONS OF URBAN HOUSEHOLDS（2017）

指标名称	Name	单位	Unit	2017	2016	2017 年比 2016 年增长（±%）<br>2017/2016（±%）
**人均可支配收入**	**Per Capita Disposable Income**	**元**	**yuan**	**47176**	**43598**	**8.2**
（一）工资性收入	Income from Wages and Salaries	元	yuan	28992	26898	7.8
（二）经营净收入	Net Income from Household Operations	元	yuan	7299	6846	6.6
（三）财产净收入	Income from Properties	元	yuan	4297	3909	9.9
（四）转移净收入	Income from Transfers	元	yuan	6588	5945	10.8
**人均消费支出**	**Per Capita Consumption Expenditure**	**元**	**yuan**	**30569**	**28285**	**8.1**
（一）食品烟酒	Food，Tobacco and Iquor	元	yuan	9059	8473	6.9
（二）衣着	Cloth	元	yuan	3117	2882	8.1
（三）居住	Residence	元	yuan	6605	6105	8.2
（四）生活用品及服务	Household Facilities，Articles and Services	元	yuan	2121	1962	8.1
（五）交通通信	Transportation and Communication	元	yuan	4399	4089	7.6
（六）教育文化娱乐	Education，Cultural and Recreation Services	元	yuan	2875	2553	12.6
（七）医疗保健	Health Care and Medical Services	元	yuan	1624	1490	9.0
（八）其他用品和服务	Other Goods and Services	元	yuan	769	731	5.2

10-11 城镇居民家庭基本情况（2018 年）
BASIC CONDITIONS OF URBAN HOUSEHOLDS（2018）

指标名称	Name	单位	Unit	2018	2017	2018 年比 2017 年增长（±%）<br>2018/2017（±%）
**人均可支配收入**	**Per Capita Disposable Income**	**元**	**yuan**	**50817**	**47176**	**7.7**
（一）工资性收入	Income from Wages and Salaries	元	yuan	30955	28992	6.8
（二）经营净收入	Net Income from Household Operations	元	yuan	7836	7299	7.4
（三）财产净收入	Income from Properties	元	yuan	4789	4297	11.4
（四）转移净收入	Income from Transfers	元	yuan	7237	6588	9.9
**人均消费支出**	**Per Capita Consumption Expenditure**	**元**	**yuan**	**32890**	**30569**	**7.6**
（一）食品烟酒	Food，Tobacco and Iquor	元	yuan	9318	9059	2.9
（二）衣着	Cloth	元	yuan	3168	3117	1.7
（三）居住	Residence	元	yuan	7751	6605	17.3
（四）生活用品及服务	Household Facilities，Articles and Services	元	yuan	2207	2121	4.0
（五）交通通信	Transportation and Communication	元	yuan	4765	4399	8.3
（六）教育文化娱乐	Education，Cultural and Recreation Services	元	yuan	3122	2875	8.6
（七）医疗保健	Health Care and Medical Services	元	yuan	1764	1624	8.6
（八）其他用品和服务	Other Goods and Services	元	yuan	795	769	3.4

10-12 农村居民家庭基本情况（2015 年）
BASIC CONDITIONS OF RURAL HOUSEHOLDS（2015）

指标名称	Name	单位	Unit	2015	2014	2015 年比 2014 年增长（±%）2015/2014（±%）
**人均可支配收入**	**Per Capita Disposable Income**	**元**	**yuan**	**16730**	**15434**	**8.4**
（一）工资性收入	Income from Wages and Salaries	元	yuan	9337	8517	9.6
（二）经营净收入	Net Income from Household Operations	元	yuan	6919	6489	6.6
（三）财产净收入	Income from Properties	元	yuan	236	216	9.3
（四）转移净收入	Income from Transfers	元	yuan	238	212	12.4
**人均消费支出**	**Per Capita Consumption Expenditure**	**元**	**yuan**	**11127**	**10277**	**8.3**
（一）食品烟酒	Food，Tobacco and Iquor	元	yuan	3442	3188	8.0
（二）衣着	Cloth	元	yuan	826	784	5.3
（三）居住	Residence	元	yuan	2377	2248	5.8
（四）生活用品及服务	Household Facilities，Articles and Services	元	yuan	765	720	6.2
（五）交通通信	Transportation and Communication	元	yuan	1965	1751	12.2
（六）教育文化娱乐	Education，Cultural and Recreation Services	元	yuan	895	809	10.6
（七）医疗保健	Health Care and Medical Services	元	yuan	604	532	13.5
（八）其他用品和服务	Other Goods and Services	元	yuan	253	245	3.3

注：本表数据为新口径，2014 年与去年年鉴数据不一致。

Note：The data in this sheet is in line with new statistic caliber，which leads to the inconsistency between the data of 2014 and of 2015.

10-13 农村居民家庭基本情况（2016 年）
BASIC CONDITIONS OF RURAL HOUSEHOLDS（2015）

指标名称	Name	单位	Unit	2016	2015	2016 年比 2015 年增长（±%）<br>2016/2015（±%）
**人均可支配收入**	**Per Capita Disposable Income**	**元**	**yuan**	**17969**	**16730**	**7.4**
（一）工资性收入	Income from Wages and Salaries	元	yuan	10074	9337	7.9
（二）经营净收入	Net Income from Household Operations	元	yuan	7381	6919	6.7
（三）财产净收入	Income from Properties	元	yuan	256	236	8.6
（四）转移净收入	Income from Transfers	元	yuan	258	238	8.2
**人均消费支出**	**Per Capita Consumption Expenditure**	**元**	**yuan**	**12006**	**11127**	**7.9**
（一）食品烟酒	Food，Tobacco and Iquor	元	yuan	3651	3442	6.1
（二）衣着	Cloth	元	yuan	905	826	9.5
（三）居住	Residence	元	yuan	2586	2377	8.8
（四）生活用品及服务	Household Facilities，Articles and Services	元	yuan	814	765	6.4
（五）交通通信	Transportation and Communication	元	yuan	2113	1965	7.5
（六）教育文化娱乐	Education，Cultural and Recreation Services	元	yuan	1004	895	12.2
（七）医疗保健	Health Care and Medical Services	元	yuan	658	604	9.0
（八）其他用品和服务	Other Goods and Services	元	yuan	275	253	8.8

10-14 农村居民家庭基本情况（2017 年）
BASIC CONDITIONS OF RURAL HOUSEHOLDS（2017）

指标名称	Name	单位	Unit	2017	2016	2017 年比 2016 年增长（±%）2017/2016（±%）
**人均可支配收入**	**Per Capita Disposable Income**	**元**	**yuan**	**19364**	**17969**	**7.8**
（一）工资性收入	Income from Wages and Salaries	元	yuan	10882	10074	8.0
（二）经营净收入	Net Income from Household Operations	元	yuan	7913	7381	7.2
（三）财产净收入	Income from Properties	元	yuan	280	256	9.0
（四）转移净收入	Income from Transfers	元	yuan	289	258	12.2
**人均消费支出**	**Per Capita Consumption Expenditure**	**元**	**yuan**	**12928**	**12006**	**7.7**
（一）食品烟酒	Food，Tobacco and Iquor	元	yuan	3869	3651	6.0
（二）衣着	Cloth	元	yuan	971	905	7.3
（三）居住	Residence	元	yuan	2801	2586	8.3
（四）生活用品及服务	Household Facilities，Articles and Services	元	yuan	885	814	8.8
（五）交通通信	Transportation and Communication	元	yuan	2262	2113	7.0
（六）教育文化娱乐	Education，Cultural and Recreation Services	元	yuan	1129	1004	12.4
（七）医疗保健	Health Care and Medical Services	元	yuan	717	658	9.0
（八）其他用品和服务	Other Goods and Services	元	yuan	294	275	6.8

10-15 农村居民家庭基本情况（2018 年）
BASIC CONDITIONS OF RURAL HOUSEHOLDS（2018）

指标名称	Name	单位	Unit	2018	2017	2018 年比 2017 年增长（±%）<br>2018/2017（±%）
**人均可支配收入**	**Per Capita Disposable Income**	**元**	**yuan**	**20820**	**19364**	**7.5**
（一）工资性收入	Income from Wages and Salaries	元	yuan	11684	10882	7.4
（二）经营净收入	Net Income from Household Operations	元	yuan	8487	7913	7.3
（三）财产净收入	Income from Properties	元	yuan	310	280	10.8
（四）转移净收入	Income from Transfers	元	yuan	339	289	17.1
**人均消费支出**	**Per Capita Consumption Expenditure**	**元**	**yuan**	**13885**	**12928**	**7.4**
（一）食品烟酒	Food，Tobacco and Iquor	元	yuan	4098	3869	5.9
（二）衣着	Cloth	元	yuan	1020	971	5.1
（三）居住	Residence	元	yuan	3024	2801	8.0
（四）生活用品及服务	Household Facilities，Articles and Services	元	yuan	945	885	6.7
（五）交通通信	Transportation and Communication	元	yuan	2445	2262	8.1
（六）教育文化娱乐	Education，Cultural and Recreation Services	元	yuan	1245	1129	10.3
（七）医疗保健	Health Care and Medical Services	元	yuan	794	717	10.6
（八）其他用品和服务	Other Goods and Services	元	yuan	314	294	6.9

10-16 全体居民家庭消费构成
HOUSEHOLDS CONSUMPTION STRUCTURE

项目	Item	2014 年		2015 年		2016 年		2017 年		2018 年	
		年人均支出金额（元）Per Capita Annual Expenditure（yuan）	占消费支出的比重（%）Percentage to Consumption Expenditure（%）	年人均支出金额（元）Per Capita Annual Expenditure（yuan）	占消费支出的比重（%）Percentage to Consumption Expenditure（%）	年人均支出金额（元）Per Capita Annual Expenditure（yuan）	占消费支出的比重（%）Percentage to Consumption Expenditure（%）	年人均支出金额（元）Per Capita Annual Expenditure（yuan）	占消费支出的比重（%）Percentage to Consumption Expenditure（%）	年人均支出金额（元）Per Capita Annual Expenditure（yuan）	占消费支出的比重（%）Percentage to Consumption Expenditure（%）
**消费支出**	**Consumption Expenditure**	**19653**	**100**	**21326**	**100**	**23256**	**100**	**25232**	**100**	**27316**	**100.0**
（一）食品烟酒	Food，Tobacco and Liquor	5970	30.4	6459	30.3	6983	30.0	7489	29.7	7787	28.5
（二）衣着	Clothing	1944	9.9	2096	9.8	2271	9.8	2467	9.8	2538	9.3
（三）居住	Residence a.r	4316	21.9	4584	21.5	5018	21.6	5454	21.6	6365	23.3
（四）生活用品及服务	Household Facilities，Articles and Services	1374	7.0	1485	7.0	1607	6.9	1747	6.9	1837	6.7
（五）交通通信	Transportation and Communication	2905	14.8	3226	15.1	3479	15.0	3753	14.9	4084	15.0
（六）教育文化娱乐	Education. Cultural and Recreation Services	1654	8.4	1830	8.6	2075	8.9	2347	9.3	2571	9.4
（七）医疗保健	Health Care and Medical Services	1002	5.1	1115	5.2	1233	5.3	1350	5.4	1480	5.4
（八）其他用品和服务	Other Goods and Services	488	2.5	531	**2.5**	**590**	**2.5**	625	2.4	654	2.4

10-17 城镇居民家庭消费构成
COMPOSITION OF URBAN HOUSEHOLDS CONSUMPTION

项目	Item	2014年		2015年		2016年		2017年		2018年	
		年人均支出金额（元）Per Capita Annual Expenditure（yuan）	占消费支出的比重（%）Percentage to Consumption Expenditure（%）	年人均支出金额（元）Per Capita Annual Expenditure（yuan）	占消费支出的比重（%）Percentage to Consumption Expenditure（%）	年人均支出金额（元）Per Capita Annual Expenditure（yuan）	占消费支出的比重（%）Percentage to Consumption Expenditure（%）	年人均支出金额（元）Per Capita Annual Expenditure（yuan）	占消费支出的比重（%）Percentage to Consumption Expenditure（%）	年人均支出金额（元）Per Capita Annual Expenditure（yuan）	占消费支出的比重（%）Percentage to Consumption Expenditure（%）
**消费支出**	**Consumption Expenditure**	**24122**	**100**	**26052**	**100**	**28285**	**100**	**30569**	**100**	**32890**	**100.0**
（一）食品烟酒	Food，Tobacco and Liquor	7295	30.2	7856	30.2	8473	30.0	9059	29.6	9318	28.3
（二）衣着	Clothing	2497	10.4	2685	10.3	2882	10.2	3117	10.2	3168	9.6
（三）居住	Residence a.r	5302	22.0	5607	21.5	6105	21.6	6605	21.6	7751	23.6
（四）生活用品及服务	Household Facilities, Articles and Services	1686	7.0	1819	7.0	1962	6.9	2121	6.9	2207	6.7
（五）交通通信	Transportation and Communication	3455	14.3	3810	14.6	4089	14.4	4399	14.4	4765	14.5
（六）教育文化娱乐	Education. Cultural and Recreation Services	2057	8.5	2264	8.7	2553	9.0	2875	9.4	3122	9.5
（七）医疗保健	Health Care and Medical Services	1227	5.1	1352	5.2	1490	5.3	1624	5.3	1764	5.4
（八）其他用品和服务	Other Goods and Services	603	2.5	659	2.5	731	2.6	769	2.6	795	2.4

10-18 农村居民家庭消费构成
COMPOSITION OF RURAL HOUSEHOLDS CONSUMPTION

项目	Item	2014年		2015年		2016年		2017年		2018年	
		年人均支出金额（元） Per Capita Annual Expenditure (yuan)	占消费支出的比重（%） Percentage to Consumption Expenditure (%)	年人均支出金额（元） Per Capita Annual Expenditure (yuan)	占消费支出的比重（%） Percentage to Consumption Expenditure (%)	年人均支出金额（元） Per Capita Annual Expenditure (yuan)	占消费支出的比重（%） Percentage to Consumption Expenditure (%)	年人均支出金额（元） Per Capita Annual Expenditure (yuan)	占消费支出的比重（%） Percentage to Consumption Expenditure (%)	年人均支出金额（元） Per Capita Annual Expenditure (yuan)	占消费支出的比重（%） Percentage to Consumption Expenditure (%)
**消费支出**	**Consumption Expenditure**	**10277**	**100**	**11127**	**100**	**12006**	**100**	**12928**	**100**	**13885**	**100.0**
（一）食品烟酒	Food，Tobacco and Liquor	3188	31.0	3442	30.9	3651	30.4	3869	29.9	4098	29.5
（二）衣着	Clothing	784	7.6	826	7.4	905	7.5	971	7.5	1020	7.3
（三）居住	Residence a.r	2248	21.9	2377	21.4	2586	21.5	2801	21.7	3024	21.8
（四）生活用品及服务	Household Facilities, Articles and Services	720	7.0	765	6.9	814	6.8	885	6.8	945	6.8
（五）交通通信	Transportation and Communication	1751	17.0	1965	17.7	2113	17.6	2262	17.5	2445	17.6
（六）教育文化娱乐	Education. Cultural and Recreation Services	809	7.9	895	8.0	1004	8.4	1129	8.7	1245	9.0
（七）医疗保健	Health Care and Medical Services	532	5.2	604	5.4	658	5.5	717	5.5	794	5.7
（八）其他用品和服务	Other Goods and Services	245	2.4	253	2.3	275	2.3	294	2.4	314	2.3

10-19 城镇住户每百户家庭主要耐用品拥有量（1980-2018 年）

OWNERSHIP OF MAJOR DURABLE CONSUMER GOODS PER 100 URBAN HOUSEHOLDS（1980-2018）

年份 Year	家用电脑（台） Compuer （set）	钢琴（架） Piano （set）	空调器（台） Air Conditioner （set）	热水器（台） Water Heater （unit）	洗衣机（台） Washing Machine （set）	电冰箱（台） Household Refrigerator （set）	家用汽车（辆） Automobile （unit）	彩色电视机（台） Color TV （set）	移动电话（部） Call Phone （unit）	照相机（架） Camera （set）
1980										2
1981					12			1		2
1982					22			2		4
1983					27	1		6		6
1984					30	2		8		8
1985					36	4		18		14
1986					40	5		25		15
1987					52	18		34		18
1988					61	33		47		21
1989					67	50		59		22
1990					66	62		68		26
1991					70	76		82		33
1992		1	0	13	73	83		81		39
1993		2	1	18	83	86		88		50
1994		4	1	17	81	89		92		51
1995		4	3	34	82	89		100		61
1996		5	7	40	83	92		105		63
1997	1	5	9	47	83	93		111	3	63
1998	8	5	17	53	83	92		111	7	69
1999	11	4	22	61	83	94		117	15	69
2000	19	2	25	66	83	94		120	30	70
2001	20	2	24	66	84	93		128	36	69
2002	32	2	38	75	89	93	1	123	80	73
2003	41	3	48	76	91	93	0	125	106	70
2004	46	3	55	79	93	93	2	124	128	68
2005	55	4	69	84	92	95	4	118	153	72
2006	71	8	89	88	97	99	6	125	187	82
2007	75	7	98	93	100	107	8	122	194	83
2008	75	5	94	90	95	103	13	116	191	67
2009	82	7	98	91	96	105	18	118	199	71
2010	86	8	99	91	97	107	20	119	208	71
2011	95	8	103	92	98	108	22	117	219	77
2012	101	9	104	93	98	108	29	115	231	79
2013	105	10	104	93	98	107	32	118	232	79
2014	105	10	104	98	99	105	34	109	235	79
2015	80	9	91	91	91	94	50	101	215	57
2016	86		98	93	90	96	58	100	212	63
2017	80		101	94	91	95	60	100	205	
2018	81		120	101	100	108	62	107	228	

10-20 农村住户每百户家庭主要耐用品拥有量（1985-2018 年）

OWNERSHIP OF MAJOR DURABLE CONSUMER GOODS PER 100 RURAL HOUSEHOLDS（1985-2018）

年份<br>Year	家用电脑（台）<br>Compuer（set）	空调器（台）<br>Air Conditioner（set）	热水器（台）<br>Water Heater（unit）	自行车（辆）<br>Bicycle（unit）	洗衣机（台）<br>Washing Machine（set）	电冰箱（台）<br>Household Refrigerator（set）	彩色电视机（台）<br>Color TV（set）	移动电话（部）<br>Call Phone（unit）	照相机（架）<br>Camera（set）	家用汽车（辆）<br>Automobile（unit）
1985				147	0.3		3		1	
1986				165	1.1	0.3	4.5		1	
1987				148	2.4	0.5	7		2	
1988				154	2.4	1	9		2	
1989				170	3	1	12		2	
1990				164	4	2	13		2	
1991				173	5	4	16		2	
1992				178	5	7	20		2	
1993				203	7	14	26		2	
1994				204	9	21	35		7	
1995				203	16	28	43		6	
1996				198	13	33	46		8	
1997				191	15	36	56		13	
1998				184	17	39	63		15	
1999				185	20	42	74		10	
2000				165	28	47	86		13	
2001				153	28	53	92		13	
2002				152	36	58	98		16	
2003	6	5	33	137	39	60	100	45	16	2
2004	8	8	39	142	48	68	105	68	21	2
2005	14	14	53	138	57	77	109	91	21	5
2006	17	15	54	116	63	87	112	114	14	7
2007	19	19	63	118	70	94	113	132	17	8
2008	25	23	68	124	76	97	114	148	19	9
2009	28	25	71	127	79	99	115	160	21	11
2010	34	30	75	128	82	102	117	170	21	12
2011	44	32	76	114	85	100	112	202	18	15
2012	55	37	85	119	91	102	114	216	21	16
2013	61	45	88	111	93	105	116	221	26	23
2014	61	49	85		93	102	106	225	24	26
2015	46	54	86		92	105	110	219	21	41
2016	56	70	89		96	107	113	226	21	45
2017	52	78	90		98	106	110	223		48
2018	45	93	88		97	109	105	232		43

主要统计指标解释

常住人员 指住户成员中，经常在家居住、或者调查期内居住时间超过一半的人员，以及本住户供养的学生。

可支配收入 指调查户在调查期内获得的、可用于最终消费支出和储蓄的总和，即调查户可以用来自由支配的收入。可支配收入既包括现金，也包括实物收入。按照收入的来源，可支配收入包含四项，分别为：工资性收入、经营净收入、财产净收入和转移净收入。计算公式为：

可支配收入 = 工资性收入 + 经营净收入 + 财产净收入 + 转移净收入

居民人均可支配收入是按居民家庭常住人口计算的平均每人可支配收入

消费支出 指住户用于满足家庭日常生活消费需要的全部支出。根据用途不同，消费支出可划分为食品烟酒、衣着、居住、生活用品及服务、交通通信、教育文化娱乐、医疗保健、其他用品及服务八大类。根据来源不同，消费支出可划分为现金消费支出、实物消费支出（含自产自用、来自单位、来自政府和其他社会组织）。

Explanatory Notes on Main Statistical Indicators

Permanent resident refers to residents that live usually in home or more than half of the survey period and students that are supported by them.

Disposable income refers to sum of all income earned within the survey period that can be used for deposit and expense on final consumption, which means the income that can be disposed freely by residents. The disposable income includes not only cash, but also income in kinds, which shall be categorized into four kinds according to source of incomes--salary income, net operating income, net property income, net transfer income. Computing formula is as follows:

Disposable income = salary income + net operating income + net property income + net transfer income

Disposable income per capita of residents is the average disposable income per person calculated based on the number of permanent residents of the family.

Consumer spending refers to all spending of residents to cover daily life consumption demands. According to different usages, the consumer spending shall be classified into eight categories: food, cigarettes and alcohol; clothing; housing; daily use articles and services; transportation & communication; education, culture & entertainment; medical and health care; other articles and services. According to sources, the consumer spending shall be classified as cash consumer spending, consumer spending in kinds (including self-produced, from the unit, from government and other social organization).

农　业 11

AGRICULTURE

简要说明

一、本篇资料的主要内容

本篇资料主要反映了全市农业生产和农村经济的基本情况，主要包括农林牧渔业总产值、中间消耗、增加值、农村劳动力、耕地、主要农产品产量、农业机械年末拥有量、农业机械化和电气化以及农田水利建设等方面的资料。

二、本篇资料的来源

1、本篇资料中耕地面积资料来源于市自然资源和规划局。

2、水产品产量等相关资料来源于市海洋发展局。

3、农业机械化等相关资料来源于市农村农业局。

4、农田水利灌溉等相关资料来源于市水务管理局。

5、植树造林等相关资料来源于市园林和林业局。

三、说明

本篇按照国家统计局规定，根据 2016 年第三次全国农业普查结果对 2007 年至 2017 年历史数据进行了核定和修正。

本篇资料由市统计局农村统计处、国家统计局青岛调查队农村调查处整理提供。

Brief Introduction

I. Main Content

Data in this chapter show the basic conditions of agricultural production and rural economy, mainly including agricultural output, intermediate consumption, value added, rural labor force, cultivated land, output of main agricultural produces, agricultural machinery and electrification in rural areas and basic construction on irrigation and drainage.

II. Source of Data

(1) Data on area of cultivated land are provided by Qingdao Natural Resources and Planning Bureau.

(2) Data on output of aquatic products are provided by Qingdao Marine Development Bureau.

(3) Data on Agricultural machinery are provided by Qingdao Rural Agriculture Bureau.

(4) Data on farmland water conservancy are provided by Qingdao Water Administration Bureau.

(5) Data on forest planting are provided by Qingdao Landscape and Forestry Bureau.

III. Explain

This chapter is in accordance with the provisions of the State Statistical Bureau. According to the results of the third national agricultural census in 2016, the historical data from 2007 to 2017 were verified and revised.

Data in this chapter are compiled by the Division of Countryside Statistics of Qingdao Municipal Bureau of Statistics and Division of Rural Household Survey of Survey Office of the National Bureau of Statistics in Qingdao.

11-1 农村基本情况（2000-2018 年）
BASIC STATISTICS ON RURAL AREA（2000-2018）

项目	Item	单位	Unit	2000	2005	2008	2009	2010	2011	2012
**一、乡村户数人口、劳动力**	**Rural Households Population and Labor Force**									
乡村户数	Rural Households	万户	10 000 households	148	151	152	152	155	155	156
乡村人口	Rural Population	万人	10 000 persons	483	479	480	485	487	489	491
乡村劳动力	Rural Labor Force	万人	10 000 persons	255	258	271	275	276	277	277
男劳动力	Male	万人	10 000 persons	137	138	144	146	147	147	146
女劳动力	Female	万人	10 000 persons	118	120	126	129	129	131	130
**二、年末实有耕地面积**	**Year-end Area of Cultivated Land**	**万公顷**	**10 000 hectares**	**48**	**42**	**42**	**42**			**53**
**三、农用机械总动力**	**Total Agricultural Machinery Power**	**万千瓦**	**10 000 kW**	**459**	**620**	**697**	**719**	**764**	**784**	**798**
拖拉机	Tractors	台	unit	126981	163032	176156	180171	200208	202022	206290
**四、农村用电量**	**Electricity Consumed in Rural Areas**	**万千瓦时**	**10 000 kW·h**	**189651**	**372645**	**424292**	**426306**	**426819**	**431646**	**426815**
**五、农用化肥施用量（折纯）**	**Consumption of Chemical Fertilizer（convert to pure amount）**	**万吨**	**10 000 tons**	**33**	**33**	**31**	**30**	**30**	**29**	**29**
**六、有效灌溉面积**	**Irrigated Area**	**万公顷**	**10 000 hectares**	**30**	**30**	**31**	**32**	**33**	**33**	**33**

11-1 续表
Continued

项目	Item	单位	Unit	2013	2014	2015	2016	2017	2018
**一、乡村户数人口、劳动力**	**Rural Households Population and Labor Force**								
乡村户数	Rural Households	万户	10 000 households	155	157	155	155	140	131
乡村人口	Rural Population	万人	10 000 persons	491	494	495	497	453	419
乡村劳动力	Rural Labor Force	万人	10 000 persons	277	277	275	273	246	224
男劳动力	Male	万人	10 000 persons	147	147	145	145	131	119
女劳动力	Female	万人	10 000 persons	131	130	130	128	115	105
**二、年末实有耕地面积**	**Year-end Area of Cultivated Land**	**万公顷**	**10 000 hectares**	**53**	**52**	**52**	**52**	**52**	**52**
**三、农用机械总动力**	**Total Agricultural Machinery Power**	**万千瓦**	**10 000 kW**	**809**	**827**	**854**	**698**	**728**	**738**
拖拉机	Tractors	台	unit	206300	205065	207809	211689	214799	215668
**四、农村用电量**	**Electricity Consumed in Rural Areas**	**万千瓦时**	**10 000 kW·h**	**359139**	**404380**	**401122**	**385434**	**382437**	**377858**
**五、农用化肥施用量（折纯）**	**Consumption of Chemical Fertilizer（convert to pure amount）**	**万吨**	**10 000 tons**	**29**	**29**	**28**	**28**	**28**	**27**
**六、有效灌溉面积**	**Irrigated Area**	**万公顷**		**30**	**32**	**32**	**33**	**33**	**33**

注：1. 农机机械总动力 2016 年为新统计口径，扣除农用运输汽车数据。
2. 乡村劳动力 2018 年为新统计口径。

Note：1.Data of total power of agricultural machinery of 2016 is based on the new statistic caliber，deducted by the data of agricultural vehicle.
2.Rural Labor Force of 2018 is based on the new statistic caliber.

11-2　农村劳动力（1985-2018 年）
RURAL LABOR FORCE（1985-2018）

单位：万人（10 000 persons）

年份<br>Year	合计<br>Total	农林牧渔业<br>Farming，Forestry，Animal Husbandry and Fishing	工业<br>Industry	建筑业<br>Construction
1985	225.09	147.89	32.92	15.62
1988	238.93	146.00	44.46	17.51
1989	242.70	151.16	44.65	16.46
1990	247.29	155.20	44.48	16.17
1991	253.01	160.31	45.49	15.87
1992	258.23	160.04	47.00	17.25
1993	259.18	159.22	44.04	20.20
1994	258.34	155.74	43.70	21.52
1995	258.66	154.08	44.06	22.33
1996	256.34	151.10	43.08	21.80
1997	258.60	153.07	41.66	22.02
1998	258.50	152.79	40.79	21.68
1999	256.82	149.11	42.18	22.32
2000	254.62	143.59	42.76	22.99
2001	253.98	132.46	47.76	24.66
2002	254.08	119.98	53.66	26.52
2003	257.67	118.27	55.65	26.65
2004	258.91	112.05	60.32	28.36
2005	257.87	103.03	65.59	29.03
2006	264.70	102.28	71.46	28.16
2007	268.31	100.63	74.70	30.97
2008	270.71	100.90	74.82	30.87
2009	274.74	102.64	75.42	31.23
2010	276.04	102.45	76.32	32.10
2011	277.24	102.73	76.87	32.57
2012	276.97	102.24	76.82	32.36
2013	277.32	104.48	76.42	31.15
2014	276.90			
2015	274.77			
2016	273.43			
2017	245.92			
2018	223.54			

注：乡村劳动力 2018 年为新统计口径。
Note：Rural Labor Force of 2018 is based on the new statistic caliber.

11-2 续表
Continued

单位：万人（10 000persons）

年份<br>Year	交通运输仓储和邮电业<br>Transport，Postal and Telecommunication Services	批发零售贸易、餐饮业<br>Wholesale and Retail Trades and Catering Services	金融保险业<br>Financial and Insurance	其他劳动力<br>Other Labour Force
1985	5.23	0.06	19.76	3.61
1988	5.89	0.09	20.40	4.58
1989	6.00	0.12	19.95	4.36
1990	6.11	0.12	20.71	4.50
1991	6.09	0.13	20.52	4.60
1992	7.02	0.16	21.74	5.02
1993	8.93	0.16	20.65	5.98
1994	6.48	10.10		20.80
1995	7.11	10.71		20.37
1996	7.33	11.78		21.25
1997	7.62	12.40		21.83
1998	8.02	13.32		21.90
1999	8.19	14.03		20.99
2000	8.39	16.10		20.79
2001	9.02	17.80		22.28
2002	10.00	20.07		23.85
2003	9.96	20.60		26.54
2004	10.20	24.08		22.96
2005	10.70	25.88		22.34
2006	11.66	25.31		24.37
2007	11.61	27.61		21.16
2008	11.06	28.76		22.09
2009	11.40	29.03		22.75
2010	11.78	28.85		22.01
2011	11.89	29.34		21.02
2012	11.96	29.56		21.11
2013	11.92	29.48		20.33
2014				
2015				
2016				
2017				
2018				

11-3 分市、区乡村户数、人口、劳动力（2018 年）
RURAL HOUSEHOLDS, POPULATION AND LABOR FORCE BY REGION（2018）

市、区名称	Region	乡村户数（万户） Rural Households (10 000 households)	乡村人口（万人） Rural Population (10 000 persons)	乡村劳动力（万人） Labour Force of Village (10 000 persons)	男劳动力 Male Laborer	女劳动力 Female Laborer	#种植业 Planting
**全市**	**Whole Municipality**	**131.23**	**419.07**	**223.54**	**118.53**	**105.02**	**74.27**
崂山区	Laoshan District	4.51	13.37	7.57	4.02	3.55	1.85
黄岛区	Huangdao District	19.55	62.38	30.65	16.44	14.21	7.72
城阳区	Chengyang District	11.80	32.30	18.06	9.51	8.55	0.93
即墨区	Jimo District	23.70	78.42	41.51	22.08	19.44	13.81
胶州市	Jiaozhou	16.93	54.79	28.63	14.73	13.90	8.93
平度市	Pingdu	35.64	116.80	63.32	33.65	29.67	27.27
莱西市	Laixi	19.10	61.00	33.79	18.10	15.70	13.76

注：1. 城阳区含红岛经济区数据。
2. 乡村劳动力 2018 年为新统计口径。

Note：1.Chengyang District includes Hongdao Economic Zone.
2.Rural Labor Force of 2018 is based on the new statistic caliber.

11-4 主要年份农、林、牧、渔业总产值（按现价计算）
MAJOR YEAR'S GROSS OUTPUT VALUE OF FARMING, FORESTRY, ANINAL HUSBANDRY AND FISHERY (CURRENT PRICE)

单位：万元（10 000 yuan）

年份 Year	农、林、牧、渔业总产值 Gross Output Value of Farming, Forestry, Animal Husbandry and Fishery	农业产值 Farming	林业产值 Forestry	牧业产值 Animal Husbandry	渔业产值 Fishery
1949	16511	14755	325	1200	231
1952	24428	21353	479	1888	708
1957	26189	22717	644	2218	610
1962	15356	13257	230	1243	626
1965	25123	21488	854	2110	671
1970	44078	36889	1851	3817	1521
1975	93213	79208	2144	7923	3938
1978	121497	94397	3112	17293	6695
1980	149436	117547	2953	22215	6721
1985	299139	215729	8986	53894	20530
1987	378981	263066	5949	73271	36695
1989	463952	303679	19134	91885	49254
1990	579877	381287	13801	119208	65581
1991	664779	417111	12875	144909	89884
1992	703433	400471	15252	150942	136768
1993	989763	509601	20845	232143	227174
1994	1424074	763366	23101	356734	280873
1995	1923771	1021413	26765	503999	371594
1996	2317434	1145399	29100	692362	450573
1997	2047712	939922	24052	578443	505295
1998	2386303	1198043	16760	633735	537765
1999	2382320	1169596	17122	618020	577582
2000	2483256	1116397	19102	692782	654975
2001	2615228	1135816	20433	758313	700666
2002	2688532	1110671	23157	796007	758697
2003	2759692	1062576	36183	859915	801018
2004	2967389	1181009	32831	937494	816055
2005	3205057	1265695	25246	1070831	843285
2006	3396096	1360755	23546	1076254	855131
2007	3665342	1460800	20801	1143644	916648
2008	3995027	1723855	24356	1195857	907793
2009	4070777	1771982	22133	1133732	980995
2010	4802184	2197935	19090	1225222	1181911
2011	5345885	2188072	19049	1500469	1426786
2012	5672486	2270097	20221	1510234	1633469
2013	6105875	2547805	21875	1553621	1711549
2014	6279086	2644851	22266	1559709	1739234
2015	6123485	2469656	21789	1529587	1814625
2016	6690910	2844023	27741	1621935	1777293
2017	6978980	3003367	30891	1663278	1830324
2018	7369968	3290651	36575	1631422	1920519

注：2007 年 -2017 年数据为根据第三次农业普查结果核定修正后数据。
Note: The 2007-2017 data are the approved revised data based on the results of the third agricultural census.

11-5 分市、区农、林、牧、渔业总产值(2018年,现价)

GROSS OUTPUT VALUE OF FARMING, FORESTRY, ANIMAL HUSBANDYRY AND FISHERY BY REGION (2018, CURRENT PRICE)

单位:万元(10 000 yuan)

市、区名称	Region	农、林、牧、渔业总产值 Gross Output Value of Farming, Forestry, Animal Husbandry and Fishery	农业 Farming	林业 Forestry	牧业 Animal Husbandry	渔业 Fishery	服务业 Services
**全市**	**Whole Municipality**	**7369968**	**3290651**	**36575**	**1631422**	**1920519**	**490802**
崂山区	Laoshan District	163756	11635	782	0	130876	20463
黄岛区	Huangdao District	1386082	485918	5474	124343	681100	89247
城阳区	Chengyang District	63200	23542	997	17952	18534	2175
即墨区	Jimo District	1371472	440457	5753	233416	570913	120933
胶州市	Jiaozhou	1019778	435255	6430	232281	256085	89728
平度市	Pingdu	1886702	1239994	8135	538832	13178	86563
莱西市	Laixi	1232117	653337	8920	480315	11401	78143
红岛经济区	Qingdao National High-tech Industrial Development Zone	246861	513	85	4282	238432	3549

11-6 农、林、牧、渔业总产值、增加值（2018年）
VALUE-ADDED OF FARMING, FORESTRY, ANIMAL, HUSBANDRY AND FISHERY (2018)

单位：万元（10 000 yuan）

		总计 Total	农业 Farming	林业 Forestry	牧业 Animal Husbandry	渔业 Fishery	服务业 Services
**总产值**	**Gross Output Value**	**7369969**	**3290650**	**36575**	**1631422**	**1920520**	**490801**
**中间消耗**	**Intermediate Expenditure**	3264395	1351102	13734	870157	775086	254316
中间物质消耗	Intermediate Expenditure of Matter	2258032	965710	8948	660739	495110	127525
对非农生产部门的劳动支出	Expenditure of Labour Services to Nonagricultural Production Development	1006363	385392	4786	209418	279976	126791

11-7 分市、区农、林、牧、渔业增加值（2018年）
VALUE-ADDED OF FARMING，FORESTRY，ANIMAL HUSBANDRY FISHERY BY REGION（2018）

单位：万元（10 000 yuan）

市、区名称	Region	增加值 Added Value	农业 Farming	林业 Forestry	牧业 Animal Husbandry	渔业 Fishery	服务业 Services
**全市**	**Whole Municipality**	**4105574**	**1939549**	**22841**	**761265**	**1145434**	**236485**
崂山区	Laoshan District	92828	7133	464		75560	9672
黄岛区	Huangdao District	779542	267985	3522	60646	404897	42492
城阳区	Chengyang District	33707	12712	608	8487	10953	947
即墨区	Jimo District	767281	256183	3701	107611	340855	58931
胶州市	Jiaozhou	576299	260697	4199	109367	158407	43629
平度市	Pingdu	1055902	751700	4780	249316	7787	42319
莱西市	Laixi	655725	382834	5517	223858	6631	36886
红岛经济区	Qingdao National High-tech Industrial Development Zone	144290	306	50	1981	140344	1609

11-8 主要年份耕地面积与播种面积
MAJOR YEAR'S CULTIVATED AND SOWN AREA

单位：万公顷（10 000 hectares）

年份<br>Year	年末实有耕地面积<br>Year-end Area of Cultivated Land	农作物播种面积<br>Sown Area of Farm Crops	# 粮食作物<br>Grain Crops	# 经济作物<br>Economic Crops
1949		93.47	84.00	7.20
1952		97.53	85.07	10.80
1957		97.87	87.07	10.53
1962		81.47	72.53	5.80
1965		82.80	71.80	8.33
1970		80.73	68.27	10.07
1975		79.82	65.84	11.01
1978		77.60	63.60	10.40
1980		75.87	60.33	12.00
1985		78.27	57.33	15.73
1987		76.67	55.87	15.93
1989		74.78	54.28	16.06
1990		76.85	56.99	15.53
1991		76.98	56.89	15.69
1992		76.96	56.37	15.64
1993	56.90	77.75	56.22	15.71
1994		76.61	54.08	16.20
1995		76.54	53.71	15.65
1996	55.01	77.26	54.81	14.90
1997		75.04	52.32	14.40
1998		77.18	53.05	14.54
1999		77.56	50.61	13.24
2000		76.47	45.07	13.98
2001		73.69	41.91	14.54
2002		72.32	40.83	13.95
2003		67.82	36.05	14.50
2004		71.33	41.40	14.11
2005	42.10	75.02	49.93	11.89
2006	41.29	74.86	49.79	11.75
2007	41.31	64.13	39.16	24.98
2008	41.79	65.29	42.56	22.73
2009	41.87	63.72	42.01	21.72
2010		64.10	42.65	21.45
2011		64.53	43.84	20.70
2012	52.81	64.36	43.95	20.40
2013	52.55	66.16	45.69	20.48
2014	52.42	66.70	46.75	19.95
2015	52.22	67.10	47.93	19.17
2016	52	68.19	48.49	19.70
2017	51.86	67.32	47.79	19.54
2018	51.86	68.37	48.11	20.26

注：1.2009 年前本表数据由统计局提供。
2.2010 年起，耕地面积由国土部门提供，粮食作物面积由青岛调查队提供，经济作物面积由统计局提供。
3.2007 年 -2017 年农作物播种面积为根据第三次农业普查结果核定修正后数据。
4. 耕地面积数据由国土部门错年提供。

Note：1.Those of before 2009 were provided by the National Bureau of Statistics.
2. From 2010,data on area of cultivated land are provided by Ministry of Land and Resources, area of grain crops are provided by Nation Bureau of Statistics in Qingdao ,area of economic crops are provided by Nation Bureau of Statistics.
3. Sown Area of Farm Crops from 2007 to 2017 is revised according to the results of the third agricultural census.
4.Arable land data provided by the land department next year.

11-9 分市、区耕地面积（2018 年）
AREA CULTIVATED LAND BY REGION（2018）

单位：公顷（hectares）

市、区名称	Region	年末耕地面积 Year-end Area of Cultivated Land	# 旱田 Dry Farmland	当年增加的耕地 Area of Culitivated Land Increased in the Year	# 新开荒地 Wasteland Newly Opened up	当年减少的耕地 Area of Culitivated Land Decreased in the Year
**全市**	**Whole Municipality**	518620	273413	366		943
崂山区	Laoshan District	898	866	0		2
黄岛区	Huangdao District	74150	71820	1		211
城阳区	Chengyang District	6682	5226	85		89
即墨区	Jimo District	99463	86312	1		182
胶州市	Jiaozhou	63764	23516	5		268
平度市	Pingdu	184250	12616	153		129
莱西市	Laixi	89378	73023	121		63

注：城阳区含红岛经济区数据。

Note：Chengyang District includes Hongdao Economic Zone.

11-10 分市、区农作物播种面积（2018 年）

SOWN AREA OF FARM CROPS BY REGION（2018）

单位：万公顷（10 000 hectares）

市、区名称	Region	总播种面积 Total Sown Area	# 粮食 Grain	# 棉花 Cotton	# 花生 Peanut	# 烟叶 Tobacco	# 蔬菜 Vegetable
**全市**	**Whole Municipality**	**68.37**	**48.11**	**0.06**	**8.00**	**0.03**	**11.32**
崂山区	Laoshan District	0.05	0.01		0.01		0.03
黄岛区	Huangdao District	7.78	4.95		1.93	0.02	0.81
城阳区	Chengyang District	0.31	0.19		0.00		0.11
即墨区	Jimo District	10.31	7.92		1.37		0.96
胶州市	Jiaozhou	9.07	6.29		0.66	0.00	2.09
平度市	Pingdu	28.08	20.03	0.06	2.41	0.01	5.23
莱西市	Laixi	12.76	8.72		1.63		2.08

注：城阳区含红岛经济区数据。

Note：Chengyang District includes Hongdao Economic Zone.

11-11 分市、区部分农作物产量（2018 年）
OUTPUT OF FARM CROPS BY REGION（2018）

单位：吨（ton）

市、区名称	Region	粮食 Grain	棉花 Cotton	花生 Peanut	烟叶 Tobacco	蔬菜 Vegetable
**全市**	**Whole Municipality**	**3100964**	**977**	**384918**	**707**	**6443615**
崂山区	Laoshan District	829		262		10845
黄岛区	Huangdao District	263545		99290	569	601531
城阳区	Chengyang District	9629		127		42497
即墨区	Jimo District	448809		59094		564332
胶州市	Jiaozhou	368571		28140	11	1071284
平度市	Pingdu	1450710	977	120207	126	2921519
莱西市	Laixi	558872		77799		1231606

注：城阳区含红岛经济区数据。
Note：Chengyang District includes Hongdao Economic Zone.

11-12 分市、区部分农作物播公顷单产量（2018年）
OUTPUT OF FARM CROPS PER HECTARE BY REGION（2018）

单位：千克（kg）

市、区名称	Region	粮食 Grain	棉花 Cotton	花生 Peanut	烟叶 Tobacco	蔬菜 Vegetable
**全市**	**Whole Municipality**	**6446**	**1649**	**4809**	**2609**	**56942**
崂山区	Laoshan District	7327		3087		32627
黄岛区	Huangdao District	5321		5147	2688	74097
城阳区	Chengyang District	5139		3289		37769
即墨区	Jimo District	5668		4326		58883
胶州市	Jiaozhou	5862		4272	3014	51303
平度市	Pingdu	7244	1649	4986	2277	55838
莱西市	Laixi	6406		4785		59214

注：城阳区含红岛经济区数据。

Note：Chengyang District includes Hongdao Economic Zone.

11-13 主要年份农作物总产量
MAJOR YEAR'S OUPTUT OF FARM CROPS

单位：吨（ton）

年份 Year	粮食 Grain	#小麦 Wheat	#玉米 Corn	棉花 Cotton	花生 Peanut	水果 Fruit	蔬菜 Vegetable
1949	723190	158815	25810	1280	71080	9955	356015
1952	967640	200380	58130	6025	115585	13468	284050
1957	921920	210690	141540	3520	90445	18869	625220
1962	628930	104340	53010	1280	35470	8366	524455
1965	917635	180610	135595	6090	88390	16034	465850
1970	1175985	243945	244530	16490	96280	33907	541145
1975	1899540	430135	420095	15390	98495	59206	693970
1978	1900850	514570	510635	8720	114370	82819	921140
1980	2164415	520045	819930	19630	160670	80840	996565
1985	2331060	961055	739455	19335	475270	200515	1253075
1987	2626029	996165	1030926	13819	394296	249655	1489250
1989	2654334	993406	1084441	14638	391782	288757	1664238
1990	2998246	1265157	1210687	17490	413636	292366	1786041
1991	3180893	1421143	1285043	25965	446880	294349	1837265
1992	2675641	1235755	981490	8160	321145	341415	2030327
1993	3164812	1463759	1248780	15344	419109	383368	2705349
1994	3106562	1392977	1252952	8326	494827	423024	3002318
1995	3291587	1480385	1365167	8177	510662	478685	3221507
1996	3390033	1513479	1466668	4001	486574	505711	3405056
1997	2523064	1584478	743199	2000	308233	438398	3333181
1998	3469821	1627261	1484182	4159	546786	529592	4036992
1999	3331124	1501182	1499775	3668	572941	606800	5459140
2000	2780481	1353445	1169649	2077	534174	651591	6649322
2001	2539257	1143679	1178851	3409	579441	675244	6250361
2002	2383802	1085936	1094731	3755	557714	579703	6847570
2003	2221697	931787	1117704	5324	568798	677361	7305618
2004	2650952	1301791	1202063	7216	580290	748516	6720886
2005	3150203	1535471	1478616	4042	503941	737300	5792110
2006	3039266	1548928	1372166	3954	471853	827755	6051239
2007	2319354	1118044	1106106	3546	495458	1366106	6180515
2008	2602592	1234264	1279002	3163	470424	1276385	6164760
2009	2590287	1276029	1228485	3213	467937	1294706	5743437
2010	2553744	1206593	1272970	2411	439872	1267226	5895190
2011	2678626	1213908	1386365	2375	441237	1262896	5948249
2012	2707295	1242299	1396704	2334	456372	1201836	5826702
2013	2791826	1295227	1432355	2057	440426	1217969	5906625
2014	2780244	1302282	1418469	1392	394461	1188690	6134061
2015	2878062	1332125	1497121	1279	342034	1189312	5988945
2016	2940668	1345273	1551200	408	365207	1207498	5915380
2017	2968896	1264073	1670338	420	383668	1187041	6277779
2018	3100964	1376646	1690817	977	384918	1092176	6443615

注：2007 年 -2017 年数据为根据第三次农业普查结果核定修正后数据。
Note：Agricultural data from 2007 to 2017 are revised according to the results of the third agricultural census.

11-13 续表 1
Continued

单位：吨（ton）

市、区名称	Region	蔬菜 Vegetable	#大白菜 Cabbage	#芹菜 Celery	#胡萝卜 Carrot	#马铃薯 Potato
**全市**	**Whole Municipality**	**6443615**	**1355475**	**172931**	**542514**	**1187282**
崂山区	Laoshan District	10845	5276	45	74	2245
黄岛区	Huangdao District	601531	296779	24395	411	84415
城阳区	Chengyang District	42497	9710	1024		2650
即墨区	Jimo District	564332	115802	14385	61384	80952
胶州市	Jiaozhou	1071284	260143	55922	5579	388271
平度市	Pingdu	2921519	463705	47768	236247	578943
莱西市	Laixi	1231606	204060	29392	238818	49806

注：1. 城阳区含红岛经济区数据。
　　2. 马铃薯作为蔬菜统计。
Note：1.Chengyang District includes Hongdao Economic Zone.
　　2. Statistics of potatoes as vegetables.

11-13 续表 2
Continued

单位：吨（ton）

市、区名称	Region	#黄瓜 Cucumer	西红柿 Tomato	#大葱 Green onion	#蒜头 Carlic	#食用菌 Edible fungi
**全市**	**Whole Municipality**	**408646**	**339822**	**745935**	**178025**	**82870**
崂山区	Laoshan District	412	122	141	43	
黄岛区	Huangdao District	9220	39107	4172	1104	64714
城阳区	Chengyang District	8242	7535	780		938
即墨区	Jimo District	51030	25547	55908	4850	13660
胶州市	Jiaozhou	22266	13408	53788	420	427
平度市	Pingdu	45760	78211	618353	162708	1602
莱西市	Laixi	271715	175893	12793	8900	1530

注：城阳区含红岛经济区数据。
Note：Chengyang District includes Hongdao Economic Zone.

11-14 分市、区猪、羊及家禽存养量（2018年）
HOGS、SHEEP、GOATS AND POULTRY IN STOCK BY REGION（2018）

单位：万头、万只（10 000 head）

市、区名称	Region	年末生猪存养量 Hoga in Stock (year-end)	#母猪 Sow	牛 Cattle and Buffaloes	#奶牛 Cows	年末羊存养量 Sheep and Goats in Stock (year-end)	年末家禽存栏 Poultry stock at the eng of the year	#蛋鸡 Egg
**全市**	**Whole Municipality**	**170.1**	**18.3**	**16.2**	**8.8**	**21.1**	**5310.2**	**1289.2**
崂山区	Laoshan District							
黄岛区	Huangdao District	30.7	2.9	1.0	0.1	4.2	530.0	178.4
城阳区	Chengyang District	0.8	0.1	0.2	0.2		10.4	10.2
即墨区	Jimo District	24.0	2.6	1.9	1.2	1.8	811.5	350.3
胶州市	Jiaozhou	24.7	2.4	1.2	0.3	4.6	358.6	236.0
平度市	Pingdu	56.9	6.7	4.0	0.7	6.7	1604.1	240.4
莱西市	Laixi	32.5	3.7	7.9	6.3	3.7	1968.0	254.2
红岛经济区	Qingdao National High-tech Industrial Development Zone	0.5	0.0	0.0	0.0	0.0	27.6	19.6

11-15 分市、区肉、蛋、奶产量（2018年）
OUTPUT OF MEAT，EGGS AND MILK BY REGION（2018）

单位：万吨（10 000 tons）

市、区名称	Region	肉类产量 Output of Meat	#猪肉 Port	#禽肉 Poultry Meat	禽蛋 Poultry Meat	牛羊奶 Milk
**全市**	**Whole Municipality**	**52.2**	**22.5**	**27.8**	**18.2**	**29.8**
崂山区	Laoshan District					
黄岛区	Huangdao District	7.2	4.0	2.5	2.5	1.1
城阳区	Chengyang District	0.1	0.1	0.0	0.1	0.5
即墨区	Jimo District	6.5	3.2	3.2	4.9	3.9
胶州市	Jiaozhou	4.7	3.5	0.9	3.3	1.7
平度市	Pingdu	16.8	7.4	8.9	3.5	2.6
莱西市	Laixi	16.8	4.2	12.2	3.6	19.9
红岛经济区	Qingdao National High-tech Industrial Development Zone	0.1	0.1	0.0	0.3	0.1

11-16 分市、区渔业养殖面积
AQUACULTURE AREA BY REGION

单位：公顷（hectare）

市、区名称	Region	2018年 养殖面积 Aquaculture Area	# 海水 Seawater	# 淡水 Fresh Water	2017年 养殖面积 Aquaculture Area	# 海水 Seawater	# 淡水 Fresh Water
**全市**	**Whole Municipality**	**35259**	**32344**	**2915**	**34941**	**32403**	**2538**
崂山区	Laoshan District	1600	1600		1600	1600	
黄岛区	Huangdao District	12076	11650	426	12124	11698	426
城阳区	Chengyang District	6728	6465	263	6871	6465	406
即墨区	Jimo District	11146	10878	268	10957	10889	68
胶州市	Jiaozhou	2276	1751	525	2059	1751	308
平度市	Pingdu	463		463	365		365
莱西市	Laixi	970		970	966		966

注：城阳区含红岛经济区数据。
Note：Chengyang District includes Hongdao Economic Zone.

11-17 分市、区水产品总产量（2018年）
OUTPUT OF AQUATIC PRODUCTS BY REGION（2018）

单位：吨（ton）

市、区名称	Region	水产品总产量 Output of Aquatic Products	# 养殖产量 Aquaculture Output	# 捕捞产量 Catch	# 鱼类 Fish	# 甲壳类 Crust	# 贝类 Shellfish	# 藻类 Algae
**全市**	**Whole Municipality**	**1035135**	**833977**	**201158**	**151451**	**56083**	**748590**	**3630**
崂山区	Laoshan District	60002	21102	38900	13200	6826	19922	1230
黄岛区	Huangdao District	350234	287655	62579	86584	17090	215045	2400
城阳区	Chengyang District	237444	214384	23060	8838	3152	213152	
即墨区	Jimo District	285057	234281	50776	12951	24407	233963	
胶州市	Jiaozhou	98492	72649	25843	26031	4552	66508	
平度市	Pingdu	1719	1719		1668	48		
莱西市	Laixi	2187	2187		2179	8		

注：城阳区含红岛经济区数据。
Note：Chengyang District includes Hongdao Economic Zone.

11-18 分市、区植树及造林面积（2018 年）
OUTPUT OF AQUATIC PRODUCTS BY REGION（2018）

市、区名称	Region	当年造林面积（公顷）Area of Afforestation in the Year(hectare)	育苗面积（公顷）Seeding Area（hectare）	森林抚育作业面积（公顷）Forest Tending Area（hectare）	零星（四旁）植树（万株）Surrounding Tree Planting（10 000trees）
**全市**	**Whole Municipality**	**9018**	**11135**	**6714**	**536.2**
崂山区	Laoshan District	80	120	1713	4.2
黄岛区	Huangdao District	1513	1862	667	
城阳区	Chengyang District	191	554	667	30.0
即墨区	Jimo District	1365	3480	1000	90.0
胶州市	Jiaozhou	1260	1790	667	17.0
平度市	Pingdu	1025	2539	1667	124.0
莱西市	Laixi	3584	790	333	271.0

注：城阳区含红岛经济区数据。
Note：Chengyang District includes Hongdao Economic Zone.

11-19 分市、区水果产量（2018 年）
AREA OF FORESTATION AND OUTPUT OF FRUITS BY REGION（2018）

单位：吨（ton）

市、区名称	Region	水果总产量 Output of Fruits（ton）	#园林水果 Garden Fruits	#瓜果类水果 Melon Fruits
**全市**	**Whole Municipality**	**1092176**	**712473**	**379703**
崂山区	Laoshan District	7099	6983	116
黄岛区	Huangdao District	112683	86006	26676
城阳区	Chengyang District	17362	15677	1684
即墨区	Jimo District	40056	16627	23429
胶州市	Jiaozhou	56681	38418	18264
平度市	Pingdu	428202	260967	167235
莱西市	Laixi	430093	287794	142299

注：城阳区含红岛经济区数据。
Note：Chengyang District includes Hongdao Economic Zone.

11-19 续表
Continued

单位：吨（ton）

市、区名称	Region	#苹果 Apple	#梨 Pear	#桃 Peach	#葡萄 Grape	#杏 Apricot	#山楂 Hawthorn	#西瓜 Watermelon	#甜瓜 Muskmelon
**全市**	**Whole Municipality**	**284595**	**96778**	**111206**	**146065**	**10422**	**2858**	**206979**	**117107**
崂山区	Laoshan District	66	95	2172	8	1578	5		11
黄岛区	Huangdao District	33686	4066	17932	4178	369	41	17030	1139
城阳区	Chengyang District	635	126	6109	5112	2888			
即墨区	Jimo District	4557	554	5930	3207	76	44	11356	5389
胶州市	Jiaozhou	14618	3639	9326	4320	5398	60	17091	214
平度市	Pingdu	96407	24198	43048	69095	113	814	140426	4702
莱西市	Laixi	134626	64102	26690	60146	1	1895	21075	105652

注：城阳区含红岛经济区数据。
Note：Chengyang District includes Hongdao Economic Zone.

11-20 分市、区果园、茶园面积和茶叶产量（2018年）
AREA OF ORCHARD AND TEA GARDEN AND PRODUCTION OF TES BY CTTY AND DISTRICT（2018）

单位：公顷 （hectare），吨（ton）

市、区名称	Region	果园面积 Area of Orchards	#苹果园 Apple Orchard	#梨园 Pear Orchard	#葡萄园 Vineyand	#桃园 Peach Orchard	茶园面积 Area of Tea Plantations	茶叶产量 Cocoon yield
**全市**	**Whole Municipality**	**28184**	**8724**	**3072**	**4527**	**4836**	**3919**	**4430**
崂山区	Laoshan District	644	3	4	1	80	1126	1261
黄岛区	Huangdao District	5489	1083	135	218	1333	2323	2577
城阳区	Chengyang District	837	32	6	170	247	65	99
即墨区	Jimo District	986	248	43	143	424	358	482
胶州市	Jiaozhou	1362	416	94	109	401	24	5
平度市	Pingdu	9972	3031	866	2079	1358	22	7
莱西市	Laixi	8895	3912	1924	1807	993	0	0

注：城阳区含红岛经济区数据。
Note：Chengyang District includes Hongdao Economic Zone.

11-21 主要年份主要农业机械拥有量
MAJOR YEAR'S OWNERSHIP OF AGRICULTURAL MACHINERY

年份 Year	农业机械总动力（万千瓦）Total Agricultural Machinery Power (10 000kW)	农用拖拉机 Agricultural Tractors 混合台 set	农用拖拉机 Agricultural Tractors 千瓦 kW	大、中型机引农具（万台）Large and Medium Towong Farm Machinery (10 000 sets)	排灌机械 Draingage and Irrigation Machinery 台 set	排灌机械 Draingage and Irrigation Machinery 万千瓦 10 000kW
1949						
1952						
1957	0.22	59	2.2	0.01		
1962	2.47	254	11.47	0.06		
1965	3.49	296	13.2	0.08		
1970	10.40	602	7963	0.07		
1975	43.94	6447	103476	0.48		
1978	75.45	15576	309287	1.02		
1980	110.44	24278	502731	1.72		
1985	174.76	33097	506217	1.71	67794	54.92
1990	253.52	55363	680903	2.03	105574	74.63
1991	250.58	57404	691332	2.20	134900	73.77
1992	253.94	57182	684182	2.28	124800	82.68
1993	261.24	56335	678161	2.34	144460	78.96
1994	278.41	58698	688886	2.39	147259	80.81
1995	289.23	56391	657025	2.28	138625	73.98
1996	303.57	59698	680447	2.32	148313	81.53
1997	326.72	66841	744424	2.42	155629	88.09
1998	364.21	87226	894985	2	156565	86.68
1999	411.44	105743	1075907	3	170875	96.20
2000	459.21	126981	1253210	3	182646	108.26
2001	496.27	141409	1394114	4	178437	104.18
2002	534.92	152920	1537544	5	182799	107.35
2003	560.94	160676	1666185	5	192930	109.44
2004	599	168061	1818276	5	192144	110
2005	620	163032	1757177	6	188689	112
2006	651	171155	1890402	6	192010	116
2007	680	173326	1958047	7	195050	118
2008	697	176156	2034453	8	208711	134
2009	719	180171	2266697	9	207648	134
2010	764	200208	2331867	10	190770	123
2011	784	202022	2649283	10	189834	127
2012	798	206290	2759004	10	190134	127
2013	809	206309	2968332	10	190433	127
2014	827	205065	2960438	10	190584	128
2015	854	207809	3176870	10	191152	128
2016	698	211689	3372681	10	191283	128
2017	728	214799	3550005	11	191417	128
2018	738	215668	3668505	0.8	232943	

注：大、中型机引农具 2018 年为新统计口径，只统计功率 58.8 千瓦以上机引农具。

Note：Large and medium type agricultural tools are the new statistical caliber in 2018，and only the statistical power is 58.8 kilowatts or more.

11-22 主要年份农业机械化用电量、化肥施用量
MAJOR YEAR'S MECHANIZATION，ELECTRICITY AND CHEMICAL FERTILIZER CONSUMPTION

年份 Year	有效灌溉面积（公顷）Irrigated Area（hectare）	配套机电井（眼）Number of Electromechanical Well（well）	机耕面积（公顷）Area of Motorized Culitivation（hectare）	机播面积（公顷）Area of Motorized Planting（hectare）	机收面积（公顷）Area of Motorized Harvesing（hectare）	农村用电量（万千瓦时）Electricity Consumption in Rural Area（10 000kW·h）	化肥施用量（折纯万吨）Chemical Fertilizer Consumption（convert to 10 000tons）
1949	8340						
1952	14980						
1957	59353		12800	13			0.19
1962	55213	191	65680	927	927	55	0.10
1965	80000	125	11433	573	247	128	0.55
1970	147613	3947	143700	2527	133	1208	1.76
1975	233980	12820	248867	54507	953	2522	2.77
1978	276660	21017	329213	178533	15060	13975	3.64
1980	296606	22472	444800	187980	55333	20492	12.06
1985	302647	42925	366786	148433	56447	41524	11.66
1990	274680	56821	426680	220593	143133	67616	20.47
1991	281500	58987	431293	254266	192946	81200	22.53
1992	281610	62100	438533	270453	207780	88024	22.60
1993	284080	63954	439300	260287	225807	102501	28.04
1994	286750	64877	436820	262846	238693	121307	28.73
1995	287020	64651	435213	271707	250267	134284	31.45
1996	289320	65237	438947	280480	235933	143740	31.36
1997	292130	66783	442213	308267	266147	150971	28.99
1998	294120	64109	442920	359447	285513	153113	32.0
1999	294790	64734	440967	396180	286367	169337	32.6
2000	295190	65179	439980	346480	292787	189651	32.5
2001	296980	65547	436576	382870	265730	228069	31.7
2002	294310	65588	433660	364680	267690	256453	31.1
2003	292410	65406	415790	404980	256600	299425	31.9
2004	292520	65501	415220	454800	314680	330609	32.5
2005	296450	65303	409610	493240	367740	372644	33.0
2006	303070	65981	403980	493750	398700	413762	32.6
2007	305760	66400	400480	499590	429630	425715	33.9
2008	313920	66828	404080	517540	455120	424292	31.1
2009	322710	67027	404080	538220	481176	426306	30.2
2010	328960	67242	578726	558611	499248	426819	29.9
2011	331690	62104	600451	567947	547108	431646	29.4
2012	333850	62112	610382	627701	566653	426815	29.1
2013	302790	170008	410601	627701	566653	359138	29.1
2014	321670	169491	411355	590798	545360	404380	28.9
2015	323400	155074	417991	640022	600696	401122	28.5
2016	327600	135057	362912	561022	511000	385434	28.37
2017	329620	139342	360085	566264	516763	382437	27.83
2018	331240	136785	359238	578082	531733	377858	27.05

11-23 分市、区农业机械化和电气化（2018 年）
MECHANIZATION AND ELECTRIFICATION IN AGRICULTURE BY REGION（2018）

市、区名称	Region	农村用电量（万千瓦小时）Electricity Consumed in Rural Areas（10 000kW·h）	机耕作业面积 Area of Motorized Cultivation(hectare）	机播地面积 Area of Motorized Planting（hectare）	机收面积 Area of Motorized Harvesting（hectare）
			面积 Area（公顷）（hectare）		
**全市**	**Whole Municipality**	**377858**	**359238**	**578082**	**531733**
崂山区	Laoshan District	8773			
黄岛区	Huangdao District	56526	41291	66390	60497
城阳区	Chengyang District	45776	1929	2284	1915
即墨区	Jimo District	60459	48795	87811	83478
胶州市	Jiaozhou	99951	45638	74359	71289
平度市	Pingdu	71387	153445	238820	213569
莱西市	Laixi	34984	68139	108418	100985

注：城阳区含红岛经济区数据。
Note：Chengyang District includes Hongdao Economic Zone.

11-24 分市、区农用化肥施用量（2018 年）
CONSUMPTION OF CHEMICAL FERTILIZERS BY REGION（2018）

单位：折纯吨（ton converted to pure amount）

市、区名称	Region	农用化肥施用量 Consumption of Chemical Fertilizers	氮肥 Nitrogenous Fertilizer	磷肥 Phosphate Fertilizer	钾肥 Potash Fertilizer	复合肥 Compound Fertilizer	附：平均每公顷耕地施用化肥量（折纯千克）Annotation：Consumption of Chemical Fertilizer Per Hectare（convert to pure volume）
**全市**	**Whole Municipality**	**270505**	**39773**	**10420**	**12197**	**208115**	**522**
崂山区	Laoshan District	330	44	9	6	270	367
黄岛区	Huangdao District	30705	5494	2242	3541	19429	414
城阳区	Chengyang District	931	173	71	73	613	139
即墨区	Jimo District	36234	13216	2276	1099	19644	364
胶州市	Jiaozhou	35225	5737	847	3926	24715	552
平度市	Pingdu	118163	5162	1305	3077	108619	641
莱西市	Laixi	48918	9948	3670	476	34825	547

注：城阳区含红岛经济区数据。
Note：Chengyang District includes Hongdao Economic Zone.

11-25 分市、区农田水利（2018 年）
FARMLAND WATER CONSERVANCY BY REGION（2018）

市、区名称	Region	有效灌溉面积（公顷）Irrigated Area（hectare）	有效灌溉面积占耕地面积比重（%）Percentage of Irrigated Area to Area of Cultivated Land（%）	机电井数（眼）Number of Electromechanical Well（well）
**全市**	**Whole Municipality**	**331240**	**63.9**	**136785**
崂山区	Laoshan District	520	57.9	1307
黄岛区	Huangdao District	44990	60.7	10229
城阳区	Chengyang District	1990	29.8	1027
即墨区	Jimo District	59560	59.9	34297
胶州市	Jiaozhou	39710	62.3	17726
平度市	Pingdu	130590	70.9	45121
莱西市	Laixi	53880	60.3	27078

注：城阳区含红岛经济区数据。
Note：Chengyang District includes Hongdao Economic Zone.

11-26　分市、区主要农业机械拥有量（2018 年）
OWNERSHIP OFMAJOR AGRICULTURAL MACHINERY BY REGION（2018）

市、区名称	Region	农业机械总动力（万千瓦）Agricultural Machinery Power（10 000kW）	平均每公顷耕地拥有量（千瓦）Power Per Hectare of Cultivated Land（kW）	大中型拖拉机 Large and Medium Tractors		小型拖拉机 Small Tractors	
				台 set	千瓦 kW	台 set	千瓦 kW
**全市**	**Whole Municipality**	**738**	**14.2**	**43457**	**2172767**	**172211**	**1495738**
崂山区	Laoshan District						
黄岛区	Huangdao District	81	10.9	2658	126473	44740	282911
城阳区	Chengyang District	31	46.4	444	21893	1547	10193
即墨区	Jimo District	111	11.2	4223	231974	35273	325210
胶州市	Jiaozhou	108	16.9	4687	316822	20649	301865
平度市	Pingdu	274	14.9	24396	1151765	33723	320365
莱西市	Laixi	133	14.9	7049	323840	36279	255194

注：城阳区含红岛经济区数据。
Note：Chengyang District includes Hongdao Economic Zone.

11-26　续表
Continued

市、区名称	Region	种植机械（台）Planting Machinery（unit）	机引犁（部）Towing Ploughs（unit）	机引耙（部）Towing Rakes（unit）	粮食加工机械（台）Grain Processing Machinery（unit）	油料加工机械（部）Oill Processing Machinery（unit）	棉花加工机械（部）Cotton Processing Machinery（unit）	饲草料加工机械（台套）Forage Processing Machinery（unit）
**全市**	**Whole Municipality**	**966513**	**160392**	**32240**	**25683**	**9144**	**132**	**23093**
崂山区	Laoshan District							
黄岛区	Huangdao District	71599	32920	109	1117	256		3544
城阳区	Chengyang District	9639	1200	207	1605	520	17	338
即墨区	Jimo District	387432	19958	1680	3259	1472		1481
胶州市	Jiaozhou	67138	15126	5242	3050	2125		3589
平度市	Pingdu	255413	21850	16500	4372	1421	6	7538
莱西市	Laixi	175292	69338	8502	12280	3350	109	6603

注：城阳区含红岛经济区数据。
Note：Chengyang District includes Hongdao Economic Zone.

主要统计指标解释

农业总产值　是以货币表现的农、林、牧、渔业全部产品的总量，它反映一定时期内农业生产的总规模和总成果。

农、林、牧、渔业的统计范围是：

（1）农业包括农作物种植业和其他农业。

农作物种植业包括谷物、豆类、薯类、棉、油料、糖料、麻类、烟叶、蔬菜、药材、瓜类和其他农作物的种植，以及茶园、桑园、果园的生产经营。其他农业包括采集野生植物的果实、纤维、树胶、树脂、油料以及柴草、野生药材、菌类等及农民家庭兼营的商品性工业。

（2）林业包括林木的栽培（不包括茶园、桑园和果园的栽培、管理和收获等活动）、林产品的采集和村及村以下合作经济组织和农户的竹木采伐。

（3）牧业包括除渔业养殖以外的一切动物饲养和放牧，以及野生动物的捕猎和饲养。

（4）渔业包括水生动物和海藻类植物的养殖和捕捞。

粮食产量　指稻谷、小麦、玉米、高粱等谷物及薯类和豆类的全社会产量。包括国有经济经营的、集体统一经营的和农民家庭经营的粮食产量，还包括工矿企业办的农场和其他生产单位的产量。其产量计算方法，豆类按去豆荚后的干豆计算；薯类（不包括芋头、木薯和马铃薯）按5公斤鲜薯折1公斤粮食计算；马铃薯作为蔬菜统计；其他粮食一律按脱粒后的原粮计算。

水产品产量　指人工养殖的水产品和天然生长的水产品的捕捞量．包括海水的鱼类、虾蟹类、贝类和藻类以及内陆水域的鱼类、虾蟹类和贝类，不包括淡水生植物。

猪、牛、羊肉产量　指当年出栏并已屠宰、除去头蹄下水后带骨头肉（即胴体重）的重量。其统计范围为全社会。

农用化肥施用量　指本年内实际用于农业生产的化肥数量，包括氮肥、磷肥、钾肥和复合肥。化肥施用量要求按折纯量计算数量。折纯量是指把氮肥、磷肥、钾肥分别按含氮、含五氧化二磷、含氧化钾的百分之百成分进行折算后的数量。复合肥按其所含主要成分折算。

公式：折纯量＝实物量 × 某种化肥有效成分含量的百分比

农业机械总动力　指用于农、林、牧、渔业生产的各种动力机械的动力总和。动力机械包括耕作、排灌、种植、植物保护、收获、农产品加工、运输、畜牧、渔业、农田水利等各种机械。不包括专门用于乡办工业、基本建设、非农业运输、科学试验和教学等非农业生产方面用的动力机械与作业机械的数量。

Explanatory Notes on Main Statistical Indicators

Gross Output Value of Agriculture　refers to the total volume of products of farming，forestry，animal husbandry，and fishery expressed in the monetary terms.It reflects the overall scale and achievements of agricultural production during a given period of time.

The scope of statistics on farming，forestry，animal husbandry，and fishery are as follows：

Farming includes cultivation of farm crops and other agricultural activities.

Cultivation of farm crops include cultivation of grain crops，legume crops，tuber-crops，cotton，oil-bearing crops，sugar crops，and cultivation and management of tea plantations，mulberry fields and orchards.

Other agricultural activities include harvesting wild fruits，fiber，tree gum，resin，oil-bearing plants，firewood，wild medicinal herbs，fungus，and rural-household commodity industries.

Forestry refers to planting trees of various kinds（excluding tea plantations，mulberry fields and orchards），collection of forestry products and cutting and felling of bamboo and trees by villages and other cooperative organizations under village level.

Animal husbandry refers to raising and grazing of all kinds of farm animals except fishing and aquatic cultivating, and hunting and rising of wild animals.

Fishery refers to cultivation and catching of fish and other aquatic products and cultivation and collection of seaweed and other aquatic plants.

Grain Output refers to the total output of rice, wheat, corn, sorghum, millet and other miscellaneous grains as well as tubers and bean in the whole country including grains produced by states farms, collective unit, industrial enterprises and mines, output of beans refers to dry beans without pods, the output of tubers(excluding taros,cassava and potatoes)was converted into that of grain at the ratio 5: 1, i. e. 5 kilograms of fresh tubers was equivalent to 1 kilogram of grain. Statistics of potatoes as vegetables. Output of all other grains refers to husked grain.

Output of Aquatic Products refers to catches of both artificially cultured and naturally grown aquatic products, including fish, shrimps, crabs and shellfish in sea and inland water as well as seaweed. Freshwater plants are not included.

Output of Pork, Beef, and Muttonrefers to the meat of slaughtered hogs, cattle, sheep and goats with head, feet, and offal taken away. Data refers to the production of the whole country.

Consumption of Chemical Fertilizers in Agriculture refers to the quantity of chemical fertilizers applied in agriculture in the year, including nitrogenous fertilizer, phosphate fertilizer, potash fertilizer, and compound fertilizer. The consumption of chemical fertilizers is required in calculation to convert the gross weight into weight containing 100% effective component (e.g. 100% nitrogen content in nitrogenous fertilizer, 100% phosphorous pent oxide contents in phosphate fertilizer, 100% potassium oxide contents in potash fertilizer) . Compound fertilizer is converted with its major component. The formula is:

Volume of effective component= physical quantity x effective component of certain chemical fertilizer (%)

Total Power of Agriculture Machinery Refers to the total mechanical power of machinery used in farming forestry animal husbandry and fishery, including machines used for ploughing, irrigation and drainage, crop growing, plant protection, harvesting, farm product processing, transport, stock breeding, fishery and water conservancy, Machinery employed for non agricultural purposes such as township industry, capital construction, non-agricultural transport, scientific experiments and for teaching is excluded.

工　业 12

INDUSTRY

简要说明

一、本篇资料的主要内容

本篇资料主要反映了全市工业生产和基本效益情况，主要包括工业企业单位数、历年工业总产值、规模以上工业、国有控股工业、国有工业、集体工业、外商投资和港澳台投资工业、大中型工业企业的主要经济指标、相关的财务分析指标和主要工业产品产量等方面的内容。

二、本篇资料的来源

1、本篇中规模以上工业资料来源于工业统计年报，由市统计局工业统计处整理提供。

2、规模以下工业资料产值与单位数均来源于抽样调查推算，工业单位数除普查年度外无分市（区）数据，由国家统计局青岛调查队工业与投资建筑业调查处整理提供。

Brief Introduction

I. Main Content

Data in this chapter show the basic condition of industrial production and benefit in Qingdao, mainly including the number of industrial enterprises, gross industrial output value and indices, the output of major industrial products and major economic and relevant financial indicators of industrial enterprises. Industrial enterprises include enterprises above designated size, state share-holding enterprises, state owned enterprises, collective owned enterprises, foreign funded enterprises, enterprises with funds from Hong Kong, Macao and Taiwan, large and medium sized enterprises.

II. Source of Data

(1) Data on industry above designated size are based on the annual report of industrial statistics, and prepared by the Division of Industry Statistics of Qingdao Municipal Bureau of Statistics.

(2) Data on output value and unit number of industry below designated size are based on the sample survey, and there is no data on number of enterprises by region except census year. The Data are complied by the Division of Industry, Investment and Construction Survey of Survey Office of the National Bureau of Statistics in Qingdao.

12-1 规模以上工业企业单位数

NUMBER OF INDUSTRIAL ENTERPRISES ABOVE DESIGNATEO SIZE

单位：个（unit）

项　目	Item	1985 年	1990 年	1995 年	2000 年	2005 年	2006 年
**总　计**	**Total**	**2411**	**2997**	**4987**	**1504**	**3697**	**4567**
**一、按隶属关系分**	**Grouped by Subordination**						
中央工业	Central Indiustry	24	16	102	18	26	17
地方工业	Local Industry	2361	2959	4821	1479	3634	4517
**二、按经济类型分**	**Gronped by Economic Types**						
国有企业	State-owned	502	568	1084	179	76	74
集体企业	Gollective-owned	1907	2386	2731	226	87	72
其他经济类型企业	Other Economic Types	2	43	1172	1099	3534	4421
# 外商及港澳台商投资企业	Foreign Funded Enterprises and Enterprises with Funds from Hong Kong，Macao and Taiwan		35	1027	593	1658	1952
**三、按轻重工业分**	**Grouped by Light and Heavy Industries**						
轻工业	Light Industry	1398	1730	2858	891	1942	2313
重工业	Heavy Industry	1013	1267	2129	613	1755	2254
**四、按企业规模分**	**Grouped by Size of Enterprises**						
大型企业	Large	26	42	89	202	51	51
中型企业	Medium	80	168	216	229	441	469
小型企业	Small	2305	2787	4480	1073	3205	4047
**五、按行业分**	**Grouped by sectors**						
黑色金属矿采选业	Miring of Non-ferrous Metal Ores		4	6	1	2	5
有色金属矿采选业	Mining of Non-ferrous Metal Ores	2	7	9	7	5	5
非金属矿采选业	Mining and Processing of Nometal Ores	43	70	87	29	34	31
食品制造业	Manufacture of Foods	244	324	437	170	121	137
# 粮食及饲料加工	Processing of Gren and Feed	62	57	60	27		
饮料制造业	Manufacture of Beverage	31	44	74	23	29	28
# 饮料酒	Beverage Liquor	20	28	27	19	20	25

注：1. 规模以上工业企业为年主营业务收入 2000 万元及以上的工业法人企业。
2. 2010 年以前为国有及年主营业务收入 500 万元以上的非国有工业企业。
3. 本表 1998 年以前为乡及乡以上工业企业单位数。
4. 2012 年行业分类按 2011 年发布的《国民经济行业分类》（GB/T4754-2011）重新划分。

Note：1. Industrial enterprises above designated siza refers to industrial enterprises with revenue from pricipal business over 20 millinon yuan.
2. Before 2010，industrial enterprises above designated size refered to all the state-owned enterprises and non-state enterprises with revennie from principal business over 5 million yuan.
3. Before 1998，the number of industrial enterprises refer to those at and above county level.
4.The seetors are grouped by " Classification Code of the Sectors of the National Economy" （GB/T4754-2011）in 2012.

12-1　续表 1
Continued

单位：个（unit）

项　目	Item	2007 年	2008 年	2009 年	2010 年	2011 年
**总　计**	**Total**	**5032**	**5628**	**5895**	**5674**	**4727**
**一、按隶属关系分**	**Grouped by Subordination**					
中央工业	Central Indiustry	9	40	44	31	31
地方工业	Local Industry	4989	5560	5826	5618	4679
**二、按经济类型分**	**Gronped by Economic Types**					
国有企业	State-owned	63	65	163	61	147
集体企业	Gollective-owned	72	61	59	53	31
其他经济类型企业	Other Economic Types	4897	5502	5673	5560	4549
# 外商及港澳台商投资企业	Foreign Funded Enterprises and Enterprises with Funds from Hong Kong，Macao and Taiwan	2091	2217	2244	2037	1618
**三、按轻重工业分**	**Grouped by Light and Heavy Industries**					
轻工业	Light Industry	2489	2704	2805	2635	2131
重工业	Heavy Industry	2543	2924	3090	3039	2596
**四、按企业规模分**	**Grouped by Size of Enterprises**					
大型企业	Large	53	48	50	52	50
中型企业	Medium	489	519	502	525	516
小型企业	Small	4490	5061	5343	5097	4161
**五、按行业分**	**Grouped by sectors**					
黑色金属矿采选业	Miring of Non-ferrous Metal Ores	7	23	22	12	12
有色金属矿采选业	Mining of Non-ferrous Metal Ores	5	6	6	4	
非金属矿采选业	Mining and Processing of Nometal Ores	33	30	26	26	23
食品制造业	Manufacture of Foods	156	158	174	167	127
# 粮食及饲料加工	Processing of Gren and Feed					
饮料制造业	Manufacture of Beverage	33	40	42	42	36
# 饮料酒	Beverage Liquor					

12-1　续表 2
Continued

单位：个（unit）

项　目	Item	1985 年	1990 年	1995 年	2000 年	2005 年	2006 年
烟草制品业	Manufacture of Tobacco	2	2	5	3	2	2
纺织业	Manufacture of Textile	169	225	330	132	300	327
纺织服装、鞋、帽制造业	Manfacture of Testilee Wearing Apparel. Footware and Caps	171	194	322	95	272	339
皮革、毛皮、羽毛(绒)及其制品业	Manufacture of Leather，Fur，Feather and Related Products	56	64	161	64	167	194
木材加工制品业	Processing of Woon and Wood Prodncts	71	89	116	19	40	53
家具制造业	Manufacture of Furniture	61	72	88	10	72	103
造纸及纸制品业	Manufacture of Paper and Paper Producls	66	74	110	56	102	128
印刷业和记录媒介的复制	Printing，Reproduction of Recording Media	61	81	215	23	44	59
文教体育用品制造业	Manufacture of Articles For Culture，Education and Sport Activities	31	27	93	48	89	91
石油、煤炭及其他燃料加工业	Oil, coal and other fuel processing industries	2	3	13	5	10	6
化学原料及制品	Manufacture of Raw Chemical Materials and Chemical Products	106	156	278	81	178	233
医药制造业	Manufacture of Medicines	16	14	39	20	30	46
化学纤维制造业	Manufacure of Chemical Fibers	2	4	26	15	14	13
橡胶制品业	Manufacture of Rubber	53	57	143	48	116	170
塑料制品业	Manufacture of Plastics	103	131	237	64	153	179
非金属矿物制品业	Manufacture of Non-mettallic Mineral Products	230	245	275	62	161	218
黑色金属压延加工业	Smelting and Pressing of Ferrous Metals	11	13	31	15	41	45
有色金属压延加工业	Smelting and Pressing of Non-ferrous Metals	6	8	19	9	28	40
金属制品业	Manufacture of Metal Products	161	186	372	88	186	250
通用设备制造业	Manufacture of General Purpose Machinery	320	398	320	94	299	397
交通运输制造业	Manufacture of Electrical Machinery and Equipment	104	114	358	52	151	194
电气机械及器材制造业	Manufacture of Electrication Machinery and Equipment	86	96	211	79	214	249
通信设备、计算机及其他电子设备制造业	Manufacture of Communication Equipment，Computers and Other Eleetronic Equipment	32	31	79	34	123	140
仪器仪表及文化、办公用机械制造业	Manufacture of Measuring Instruments and Machinery for Cultural Activity and Office Work	28	33	70	20	40	44
水的生产和供应业	Production and Supply of Water	8	12	12	8	10	10
电力、热力的生产和供应业	Production and Supply of Electric Power and Heat Power	10	16	20	17	27	32
燃气生产和供应业	Production and Supply of Gas	1	4	3	2	5	6
其他工业	Other Industrial Sectors	46	48	209	63	166	534

12-1 续表 3
Continued

单位：个（unit）

项 目	Item	2007 年	2008 年	2009 年	2010 年	2011 年
烟草制品业	Manufacture of Tobacco	2	2	2	1	1
纺织业	Manufacture of Textile	340	330	322	287	229
纺织服装、鞋、帽制造业	Manfacture of Testilee Wearing Apparel. Footware and Caps	362	418	434	392	295
皮革、毛皮、羽毛(绒)及其制品业	Manufacture of Leather，Fur，Feather and Related Products	197	209	210	211	160
木材加工制品业	Processing of Woon and Wood Prodncts	64	66	68	68	49
家具制造业	Manufacture of Furniture	119	130	133	117	97
造纸及纸制品业	Manufacture of Paper and Paper Producls	126	133	133	138	101
印刷业和记录媒介的复制	Printing，Reproduction of Recording Media	63	74	79	92	58
文教体育用品制造业	Manufacture of Articles For Culture，Education and Sport Activities	97	96	99	90	75
石油、煤炭及其他燃料加工业	Oil, coal and other fuel processing industries	7	7	9	8	7
化学原料及制品	Manufacture of Raw Chemical Materials and Chemical Products	261	276	275	262	231
医药制造业	Manufacture of Medicines	48	49	56	52	44
化学纤维制造业	Manufacure of Chemical Fibers	12	13	14	12	10
橡胶制品业	Manufacture of Rubber	183	211	214	196	187
塑料制品业	Manufacture of Plastics	199	227	234	233	169
非金属矿物制品业	Manufacture of Non-mettallic Mineral Products	259	311	320	327	303
黑色金属压延加工业	Smelting and Pressing of Ferrous Metals	39	24	27	31	29
有色金属压延加工业	Smelting and Pressing of Non-ferrous Metals	40	41	40	35	34
金属制品业	Manufacture of Metal Products	271	347	376	361	294
通用设备制造业	Manufacture of General Purpose Machinery	473	535	592	597	521
交通运输制造业	Manufacture of Electrical Machinery and Equipment	217	264	290	296	263
电气机械及器材制造业	Manufacture of Electrication Machinery and Equipment	274	303	300	281	230
通信设备、计算机及其他电子设备制造业	Manufacture of Communication Equipment，Computers and Other Eleetronic Equipment	152	163	168	167	124
仪器仪表及文化、办公用机械制造业	Manufacture of Measuring Instruments and Machinery for Cultural Activity and Office Work	47	47	55	54	39
水的生产和供应业	Production and Supply of Water	10	13	14	14	11
电力、热力的生产和供应业	Production and Supply of Electric Power and Heat Power	34	36	39	38	36
燃气生产和供应业	Production and Supply of Gas	7	13	13	14	11
其他工业	Other Industrial Sectors	895	1033	1109	1049	921

12-1 续表 4
Continued

单位：个（unit）

项 目	Item	2012 年	2013 年	2014 年	2015 年	2016 年	2017 年	2018 年
**总计**	**Total**	**4817**	**4917**	**4790**	**4876**	**4431**	**3569**	**3508**
**一、按隶属关系分**	**Grouped by Subordination Relation**							
中央工业	Central Indiustry	15	30	28	33	27	32	43
地方工业	Local Industry	4784	4871	4747	4831	4398	3532	3465
**二、按经济类型分**	**Gronped by Economic Types**							
国有企业	State-owned	144	140	132	141	128	131	145
集体企业	Gollective-owned	28	24	19	15	11	5	4
其他经济类型企业	Other Economic Types	4645	4753	4639	4720	4292	3433	3359
# 外商及港澳台商投资企业	Foreign Funded Enterprises and Enterprises with Funds from Hong Kong，Macao and Taiwan	1599	1551	1445	1361	1205	1037	938
**三、按轻重工业分**	**Grouped by Light and Heavy Industries**							
轻工业	Light Industry	2153	2190	2080	2095	1875	1522	1448
重工业	Heavy Industry	2664	2727	2710	2781	2556	2047	2060
**四、按企业规模分**	**Grouped by Size of Enterprises**							
大型企业	Large	98	97	91	89	85	78	76
中型企业	Medium	596	599	568	566	516	459	383
小型企业	Small	4123	4221	4131	4221	3830	3032	3049
**五、按行业分**	**Grouped by sectors**							
黑色金属采选业	Mining of ferrous metal Ores	10	11	8	7	1		1
有色金属采选业	Mining of non-ferrous Metal Ores							1
非金属矿采选业	Mining and processing of Nometal Ores	14	13	7	6	5	2	1
农副食品加工业	Processing of Food from Agricultural Products	454	468	444	458	419	338	331
食品制造业	Manufacture of Foods	129	123	109	116	115	111	115
酒、饮料和精制茶制造业	Manufacture of Liquor，Beverage and Refind Tea	34	35	34	38	33	18	17

注：国家统计局根据《国民经济行业分类》(GB/T 4754-2017)，对《三次产业划分规定 (2012)》中行业类别进行了对应调整，其中“石油加工、炼焦和核燃料加工业”更名为“石油、煤炭及其他燃料加工业”，并将 2011 版《国民经济行业分类》4500 中部分内容和 4120 全部内容调到此类。

Note：According to the national economic industry classification (GB/ T 4754-2017), the National Bureau of Statistics carries out a corresponding adjustment to the three industries according to the classification of the national economy (GB/ T 4754-2017), in which the petroleum processing, coking and nuclear fuel processing industry is changed to petroleum, coal and other fuel processing industries, and the content of part of the content and 4120 of the 2011 edition of the national economy industry category 4500 are transferred to this category.

12-1 续表 5
Continued

单位：个（unit）

项 目	Item	2012 年	2013 年	2014 年	2015 年	2016 年	2017 年	2018 年
烟草制品业	Manufacture of tobacco	1	1	1	1	0	0	0
纺织业	Manufacture of Textile	181	175	146	133	111	91	93
纺织服装、服饰业	Manufacture of Texitile Wearing Apparel	312	283	249	244	206	152	144
皮革、毛皮、羽毛及其制品和制鞋业	Manufacture of Leather，Fur，Feather & Its Products Footwear	177	148	124	116	89	58	47
木材加工和木、竹、藤、棕、草制品业	Processing of Timbers，Manufacture of Wood，Bamboo，Rattan，Palm and Straw Producls	34	40	32	34	31	27	22
家具制造业	Manufacture of Furniture	103	107	96	98	88	65	64
造纸和纸制品业	Manufacture of Paper and Paper Producls	100	71	54	52	50	40	40
印刷和记录媒介复制业	Printing，Reproduction of Recording Media	80	121	150	156	137	110	101
文教、工美、体育和娱乐用品制造业	Manufacture of Ariticles for Culture，Ars & Crafts，Sports and Entertaiment	263	335	348	345	310	222	179
石油、煤炭及其他燃料加工业	Oil, coal and other fuel processing industries	8	8	9	9	9	9	8
化学原料和化学制品制造业	Manufacture of Chemical Raw Material and Chemical Products	224	240	246	249	223	176	171
医药制造业	Manufacture of Medicines	45	49	50	54	47	35	34
化学纤维制造业	Manufacure of Chemical Fibers	9	8	8	8	6	7	8
橡胶和塑料制品业	Manufacture of Rubber and Plastic	350	345	331	310	281	250	252
非金属矿物制品业	Manufacture of Non-mettallic Mineral Products	325	333	330	338	315	228	218
黑色金属冶炼和压延加工业	Smelting and Pressing of Ferrous Metals	105	93	87	83	73	27	30
有色金属冶炼和压延加工业	Smelting and Pressing of Non-ferrous Metals	37	36	38	39	30	23	24
金属制品业	Manufacture of Metal Products	399	389	380	388	348	289	278
通用设备制造业	Manufacture of General Purpose Machinery	362	382	395	402	383	301	316
专用设备制造业	Manufacture of Special Purpose Machinery	312	322	314	336	311	233	245
汽车制造业	Manufacture of Vehicle	136	141	145	158	154	156	168
铁路、船舶、航空航天和其他运输设备制造业	Manufacture of Trarisport Epquipment for Railway，Shipping，Aerospace and other uses	145	146	152	168	156	150	155
电气机械和器材制造业	Manufacture of Electrical Machinery & Equipment	202	212	217	226	211	184	186
计算机、通信和其他电子设备制造业	Manufactuter of Computer，Communication Equipment and Other Electronic Equpment	137	136	141	147	140	128	114
仪器仪表制造业	Manufacture of Measuring Instument	41	49	52	58	50	46	49
其他制造业	Manufacture of Other Products	18	17	13	15	16	10	11
废弃资源综合利用业	Reeycling and Disposal of Waste Resources	4	6	6	5	6	3	3
金属制品、机械和设备修理业	Maintenance of Metal Products，Machinery and Equipment	7	7	7	6	5	6	6
电力、热力生产和供应业	Production and Supply of Electric Power and Heat Power	36	39	40	43	42	43	44
燃气生产和供应业	Production and Supply of Gas	12	13	12	14	14	14	15
水的生产和供应业	Production and Supply of water	11	15	15	16	16	17	18

12-2 分市、区规模以上工业企业数（2018 年）
NUMBER OF ALL INDUSTRIAL ENTERPRISES BY REGION（2018）

单位：个（unit）

市、区名称	Region	规模以上 Above Designated	按轻重工业分 Grouped by Light and Heavy Ind		按企业规模分 Grouped by Size of Enterprises		
			轻工业 Light Industry	重工业 Heavy Industry	大型企业 Large	中型企业 Medium	小型企业 Small
**全 市**	**Whole Municipality**	**3508**	**1448**	**2060**	**76**	**383**	**3049**
市内三区	Three Districts in Urban Area	151	45	106	8	25	118
崂山区	Laoshan District	82	36	46	6	11	65
黄岛区	Huangdao District	731	214	517	20	97	614
城阳区	Chengyang District	474	191	283	11	52	411
即墨区	Jimo District	517	252	265	10	59	448
胶州市	Jiaozhou	737	334	403	5	59	673
平度市	Pingdu	381	169	212	4	33	344
莱西市	Laixi	279	160	119	6	23	250
红岛经济区	Qingdao National High-tech Industrial Development Zone	115	34	81	1	16	98
保税港区	Qingdao Free Trade Port Area of China	41	13	28	5	8	28

12-2 续表
Contiued

单位：个（unit）

市、区名称	Region	按经济类型分 Grouped by Economic Types			
		国有企业 State-owned	集体企业 Gollective-wned	其他经济 Others	# 外资及港澳台商企业 Foreign Funded Entreprises and Enterprises with Funds from Kong Kong，Macao and Taiwan
**全 市**	**Whole Municipality**	**145**	**4**	**3359**	**938**
市内三区	Three Districts in Urban Area	28	1	122	35
崂山区	Laoshan District	6		76	23
黄岛区	Huangdao District	42		689	179
城阳区	Chengyang District	13	1	460	169
即墨区	Jimo District	11		506	138
胶州市	Jiaozhou	9		728	161
平度市	Pingdu	19	2	360	91
莱西市	Laixi	5		274	80
红岛经济区	Qingdao National High-tech Industrial Development Zone	12		103	41
保税港区	Qingdao Free Trade Port Area of China			41	21

12-3 历年全部工业总产值
GROSS INDUSTRIAL OUTPUT VALUE OVER THE YEARS

单位：万元（10 000 yuan）

年 份<br>Year	工业总产值<br>Gross Industrial Output Value	轻工业产值<br>Light Industry	重工业产值<br>Heavy Industry
1949	21605	18494	3111
1950	34010	28720	5290
1951	53538	44600	8938
1952	84295	69258	15037
1953	115222	74716	40506
1954	126064	80604	45460
1955	118935	86956	31979
1956	144833	93809	51024
1957	142920	101202	41718
1958	217648	92407	125241
1959	282610	84376	198234
1960	291451	77043	214408
1962	120738	64233	56505
1963	139542	83630	55912
1964	177273	108884	68389
1965	215536	141765	73771
1966	252896	152304	100592
1967	287739	163627	124112
1968	299814	175791	123489
1969	296814	188860	107954
1970	345212	202903	142309

注：本表按当年价格计算，1995 年开始工业总产值按新规定计算。
Note：The data in this form are calculated at current price. Since 1995，new regulations have been adopted in calcnlating gross industrial output value.

12-3 续表 1
Continued

单位：万元（10 000 yuan）

年　份 Year	工业总产值 Gross Industrial Output Value	轻工业产值 Light Industry	重工业产值 Heavy Industry
1971	365770	216172	149598
1972	378266	230309	147957
1973	377943	245371	132572
1974	237219	154054	83165
1975	402541	261418	141123
1976	439793	278514	161279
1977	490669	296728	193941
1978	565503	336817	228686
1979	625227	379276	245951
1980	677690	442078	235612
1981	707434	479954	227480
1982	732705	474767	257938
1983	816793	513759	303034
1984	918364	569080	349284
1985	1113891	699524	414367
1986	1316230	833864	482366
1987	1703830	1065873	637957
1988	2481594	1451215	1030379
1989	3191643	1857019	1334624
1990	3571845	2057968	1513878

12-3 续表 2
Continued

单位：万元（10 000 yuan）

年　份	Year	工业总产值 Gross Industrial Output Value	轻工业产值 Light Industry	重工业产值 Heavy Industry
1991	1991	3986427	2318257	1668170
1992	1992	4826834	2835728	1991106
1993	1993	5929236	3422107	2507129
1994	1994	8456974	4931262	3525712
1995	1995	9412635	5579637	3832998
1996	1996	11173672	6793007	4380665
1997	1997	13704735	8620710	5084025
1998	1998	15794106	10153634	5640472
1999	1999	16822436	10918827	5903609
2000	2000	19408338	12433214	6975124
2001	2001	22398464	14368582	8029882
2002	2002	25794324	14811106	10983218
2003	2003	31195595	17306539	13889056
2004	2004	39590783	21473202	18117581
2005	2005	50017829	24697792	25320037
2006	2006	59188120	28686826	30501294
2007	2007	74306364	33463071	40843293
2008	2008	89467300	37871227	51596073
2009	2009	102556155	42715781	59840374
2010	2010	116148345	44954630	71193715
2011	2011	132779629	50854938	81924691

12-3 续表 3
Continued

单位：万元（10 000 yuan）

年 份	Year	工业总产值 Gross Industrial Output Value	轻工业产值 Light Industry	重工业产值 Heavy Industry
2012	2012	153102565	60516341	92546833
2013	2013	168971805	66799317	102172488
2014	2014	174442125	67683545	106758580
2015	2015	180194299	71176748	109017551
2016	2016	174157133	68552071	105605062
2017	2017	130197343	48954201	81243142
2018	2018	113897787	39054621	74843166
“一五”时期合计	“First Five-Year Plan” Peiod	647974	437287	210687
“二五”时期合计	“Second Five-Year Plan” Peiod	1077075	388406	688669
1963-1965 年合计	1963-1965	532351	334279	198072
“三五”时期合计	“Third Five-Year Plan” Peiod	1481941	883485	598456
“四五”时期合计	“Fourth Five-Year Plan” Peiod	1761739	1107324	654415
“五五”时期合计	“Fifth Five-Year Plan” Peiod	2798882	1733413	1065469
“六五”时期合计	“Sixth Five-Year Plan” Peiod	4289187	2737084	1552103
“七五”时期合计	“Seventh Five-Year Plan” Peiod	12265142	7265939	4999204
“八五”时期合计	“Eighth Five-Year Plan” Peiod	32612106	19087323	13524783
“九五”时期合计	“Ninth Five-Year Plan” Peiod	76903287	48919392	27983895
“十五”时期合计	“Tenth Five-Year Plan” Peiod	168996995	92657221	76339774
“十一五”时期合计	“Eleventh Five-Year Plan” Peiod	175336465	73641456	101695009
“十二五”时期合计	“Twelveth Five-Year Plan” Peiod	809490422	317030889	492420143

12-4　分市、区规模以上工业总产值（2018 年）

GROSS INDUSTRIAL OUTPUT VALUE ABOVE DESIGNATED SFZE BY REGION（2018）

指标	Indicator	总　计<br>Total	市内三区<br>Three Districts in Urban Area	崂山区<br>Laoshan District	黄岛区<br>Huangdao District
**总　　计**	**Total**	**96048610**	**6893905**	**5277439**	**38932632**
**按隶属关系分**	**Groped by Subordination**				
中央	Centrality	18295613	2106056	162509	7961919
县级及以下	At and below the county level	13037485	1412443	181488	6979832
其它	Others	64715512	3375406	4933441	23990881
**按登记注册类型分**	**Grouped by Status of Registration**				
国有经济	State-owned	32973045	3516434	355601	17966417
集体经济	Collecive-owned	78599	2305		
其他经济	Others	62996966	3375167	4921838	20966215
# 外资与港澳台企业	Foreign Funded Enterprises and Enterprises with Funds fron Hong Kong，Macao and Taiwan	24092865	1750726	763842	9339786
**按轻重工业分**	**Grouped by Light and Heavy Industries**				
轻工业	Light Industry	32934284	2850483	3572441	12104305
重工业	Heavy Industry	63114325	4043422	1704998	26828327
**按企业规模分**	**Grouped by Size of Enterprises**				
大型企业	Large	43857362	3121930	3938487	19313541
中型企业	Medium	25358975	2308370	581485	13075708
小型企业	Small	26832272	1463606	757467	6543383

单位：万元（10 000 yuan）

城阳区 Chengyang District	即墨区 Jimo District	胶州市 Jiaozhou	平度市 Pingdu	莱西市 Laixi	红岛经济区 Qingdao National High-tech Industrial Development Zone	保税港区 Qingdao Free Trade Port Area China
**12726134**	**10054580**	**7589934**	**6172057**	**4800416**	**2470580**	**1130934**
4918626	2811187	6088	77632		251596	
622979	1645990	31908	1733622	68767	360457	
7184529	5597404	7551938	4360803	4731649	1858527	1130934
5461724	2900694	132297	1836391	63114	740373	
52333			23962			
7212076	7153886	7457638	4311705	4737302	1730207	1130934
3074188	1675191	2440089	1570745	1593051	993867	891380
2281945	3614440	2668145	2330813	2871417	456086	184210
10444189	6440140	4921789	3841244	1928998	2014494	946724
6385829	4961923	1080572	1987532	2232149	241153	594247
2109033	1845278	2313001	1063409	862899	891632	308160
4231272	3247379	4196360	3121116	1705368	1337795	228527

12-4 续表
Continued

指标	Indicator	总　计 Total	市内三区 Three Districts in Urban Area
**按行业分**	**Grouped by Industrial Sector**	**96048610**	**6893905**
煤炭开采和洗选业	Mining and Washing of Coal		
石油和天然气开采业	Extraction of Petroleum and Natural Gas		
黑色金属矿采选业	Mining of Ferrous Metal Ores		
有色金属矿采选业	Mining of Non-ferrous Metal Ores	12298	
非金属矿采选业	Mining and Processing of Nometal Ores	4395	
开采辅助活动	Auxiliary Activities of Mining		
其他采矿业	Mining of Other Ores		
农副食品加工业	Processing of Food from Agricultural Products	6763720	848287
食品制造业	Manufacture of Foods	2393024	57153
酒、饮料和精制茶制造业	Manufacture of Liquor，Beverage and Refind Tea	1619606	619041
烟草制品业	Manufacture of Tobacco		
纺织业	Manufacture of Textile	874484	25467
纺织服装、服饰业	Manufacture of Textile Wearing Apparel	2300334	6609
皮革、毛皮、羽毛及其制品和制鞋业	Processing of Leather，Fur，Feather & Its Products Footwear	662706	9381
木材加工和木、竹、藤、棕、草制品业	Processing of Timbers，Manufacture of Wood，Bamboo，Rattan，Palm and Straw Products	118525	
家具制造业	Manufacture of Furniture	414226	18300
造纸和纸制品业	Manufacture of Paper and Paper Products	460542	5641
印刷和记录媒介复制业	Printing，Reproduction of Recording Media	859851	106617
文教、工美、体育和娱乐用品制造业	Manufacture of Articles for Culture，Arts & Crafts，Sports and Entertainment	1176298	44975
石油、煤炭及其他燃料加工业	Oil, coal and other fuel processing industries	7406847	1412239
化学原料和化学制品制造业	Manufacture of Chemical Raw Material and Chemical Products	4789079	130356
医药制造业	Manufacture of Medicines	1047219	23317
化学纤维制造业	Manufacture of Chemical Fibers	50643	
橡胶和塑料制品业	Manufacture of Rubber and Plastic	3990200	69274
非金属矿物制品业	Manufacture of Non-mettallic Mineral Products	2652062	191078
黑色金属冶炼和压延加工业	Smelting and Pressing of Ferrous Metals	2824713	
有色金属冶炼和压延加工业	Smelting and Pressing of Nonferrous Metals	606704	
金属制品业	Manufacture of Metal Products	3618624	25057
通用设备制造业	Manufacture of General Purpose Machinery	4391881	227471
专用设备制造业	Manufacture of Special Purpose Machinery	2796824	142341
汽车制造业	Manufacture of Vehicle	10008249	122242
铁路、船舶、航空航天和其他运输设备制造业	Manufacture of Transport Equipment for Railway，Shipping，Aerospace and other uses	7824563	528848
电气机械和器材制造业	Manufacture of Electrical Machinery & Equipment	15075296	1007553
计算机、通信和其他电子设备制造业	Manufacture of Computer，Communication Equipment and Other Electronic Equipment	8169914	409309
仪器仪表制造业	Manufacture of Measuring Instrument	838520	25417
其他制造业	Manufacture of Other Products	130145	18892
废弃资源综合利用业	Recycling and Disposal of Waste Resources	10287	
金属制品、机械和设备修理业	Manufacture of Metal Products，Machinery and Equipment	19641	4542
电力、热力生产和供应业	Production and Supply of Electric Power and Heat Power	1245197	503507
燃气生产和供应业	Production and Supply of Gas	651732	199774
水的生产和供应业	Production and Supply of Water	240264	111218

单位：万元（10 000 yuan）

崂山区 Laoshan District	黄岛区 Huangdao District	城阳区 Chengyang District	即墨区 Jimo District	胶州市 Jiaozhou	平度市 Pingdu	莱西市 Laixi	红岛经济区 Qingdao National High-tech Industrial Development Zone	保税港区 Qingdao Free Trade Port Area China
5277439	38932632	12726134	10054580	7589934	6172057	4800416	2470580	1130934
						12298		
					4395			
40233	1301805	434971	400745	720598	1271723	1605519	124212	15628
70582	1077315	148621	134144	361484	68144	437913	37669	
263876	382282	1400	27196	2509	320736	2564		
23961	54852	236835	100283	85896	115933	185020	10052	36187
12594	119009	168023	1678440	141878	86186	59382	28213	
7460	49925	17773	50730	210114	1401	315923		
	51266	31327	11895	20643			3394	
	13250	106226	34277	185267	48238	2340	6330	
14700	147635	139409	9700	113011	28975	1473		
8778	101766	164121	339331	63331	11647	14860	45450	3951
3945	120026	193988	206514	392933	133210	55522	3526	21659
	5530218	456668	7092	629				
19538	2470153	466132	152725	424804	634350	230188	255047	5785
218203	468435	103792	33528	13411	20954	34174	131405	
	7736	19162	2926	5595		15224		
61702	1577577	443277	656996	280605	331036	385477	146009	38246
35457	1002749	255639	264056	281176	358741	210929	32148	20089
	2484440	157784	130479	6402	10956	34653		
	88149	25989	25922	73762	355987		36896	
15650	859050	621751	678110	1085345	166934	80651	45406	40672
25352	1036774	279756	421653	1608493	259675	74664	264492	193551
74886	1234781	597994	156325	274712	51671	39391	146210	78513
34940	4207526	787849	3317382	181353	226001	771824	251297	107837
192921	869575	5350110	80497	236292	116829	8263	441229	
3693994	8393802	410047	840153	488643	77051	60309	37547	66196
172090	4440514	888940	160583	62070	1286584	30779	249471	469574
250414	87931	79136	27223	158147	7625	21765	147814	33048
4465		30859		5175	70754			
				1751	5592	2943		
	10553		4546					
27515	461990	38502	57707	17171	67673	56134	14997	
	205932	59681	35279	81043	23239	41735	5050	
4184	75618	10372	8143	5692	9820	8500	6717	

12-5　规模以上工业企业主要指标（2018 年）

MAN INDICATORS OFINDUSTRIAL ENTERPRISES ABOVE DESIGNATED SIXE（2018）

项目	Indicator	企业单位数<br>（个）<br>Number of Enterprises<br>（Unit）
**总　计**	**Total**	**3508**
**按注册登记类型分**	**Grouped by Status of Registration**	
国有及国有控股企业	State-owned and State-holdinig Enterprises	145
集体企业	Gollective-owned Enterprises	4
股份有限公司	Share-holding Corporations Ltd	103
外商投资企业	Fereign Funded Enterprises	751
港澳台商投资企业	Invested by HongKong，Macao and Taiwan	187
**按轻重工业分**	**Grouped by Light and Heavy Industries**	
轻工业	Light Industry	1448
重工业	Heavy Industry	2060
**按企业规模分**	**Grouped by Size of Enterprises**	
大型企业	Large	76
中型企业	Medium	383
小型企业	Small	3049
**按所在地分**	**Grouped by Location**	
市内三区	Three Districts in Urban Area	151
崂山区	Laoshan District	82
黄岛区	Huangdao District	731
城阳区	Chengyang District	474
即墨区	Jimo District	517
胶州市	Jiaozhou	737
平度市	Pingdu	381
莱西市	Laixi	279
红岛经济区	Qingdao National High-tech Industrial Development Zone	115
保税港区	Qingdao Free Trade Port Area of China	41
**按行业分**	**Grouped by sector**	
煤炭开采和洗选业	Mining and Washing of Coal	
石油和天然气开采业	Extraction of Petroleum and Natural Gas	
黑色金属矿采选业	Mining of Ferrous Metal Ores	
有色金属矿采选业	Mining of Non-ferrous Metoul Ores	1
非金属矿采选业	Mining and Processing of Nometal Ores	1
开采辅助活动	Auxiliary Activities of Mining	
其他采矿业	Mining of Other Ores	

单位：万元（10 000 yuan）

工业总产值<br>Gross Industrial Output Value	资产合计<br>Total Assets	流动资产年合计<br>Total Current Assets	固定资产净值<br>Net Value of Fixed Assets
**96048610**	**128639538**	**78607024**	**23334538**
32973045	42028433	27281061	8800214
78599	59050	38424	11735
20896522	46012470	27234709	3904589
19935021	23316927	16458795	4296015
4157844	4325408	2741677	1146110
32934284	50083387	27733925	6874143
63114325	78556151	50873099	16460396
43857362	72492349	45967165	8623051
25358975	24659089	14635748	6383878
26832272	31488100	18004111	8327609
6893905	10008662	5084233	2308825
5277439	11889495	6934242	1045610
38932632	52169793	32374618	8686548
12726134	15338963	11124619	2061443
10054580	11700272	7360097	2280735
7589934	10995580	5866366	3173744
6172057	6919282	3680384	1557441
4800416	4726725	3111261	1206571
2470580	4059158	2551313	781924
1130934	831610	519891	231699
12298	67057	2451	50918
4395	10223	6909	1361

12-5 续表 1
Continued

项目	Indicator	营业收入<br>Revenue from Principal Business
**总 计**	**Total**	**103439056**
**按注册登记类型分**	**Grouped by Status of Registration**	
国有及国有控股企业	State-owned and State-holdinig Enterprises	35976282
集体企业	Gollective-owned Enterprises	85895
股份有限公司	Share-holding Corporations Ltd	23440589
外商投资企业	Fereign Funded Enterprises	21245635
港澳台商投资企业	Invested by HongKong，Macao and Taiwan	4736585
**按轻重工业分**	**Grouped by Light and Heavy Industries**	
轻工业	Light Industry	37245414
重工业	Heavy Industry	66193642
**按企业规模分**	**Grouped by Size of Enterprises**	0
大型企业	Large	49966203
中型企业	Medium	25504239
小型企业	Small	27968614
**按所在地分**	**Grouped by Location**	
市内三区	Three Districts in Urban Area	8718600
崂山区	Laoshan District	6272071
黄岛区	Huangdao District	41477166
城阳区	Chengyang District	13210425
即墨区	Jimo District	10588052
胶州市	Jiaozhou	7615308
平度市	Pingdu	6858600
莱西市	Laixi	4954742
红岛经济区	Qingdao National High-tech Industrial Development Zone	2574639
保税港区	Qingdao Free Trade Port Area of China	1169454
**按行业分**	**Grouped by sector**	
煤炭开采和洗选业	Mining and Washing of Coal	
石油和天然气开采业	Extraction of Petroleum and Natural Gas	
黑色金属矿采选业	Mining of Ferrous Metal Ores	
有色金属矿采选业	Mining of Non-ferrous Metoul Ores	12810
非金属矿采选业	Mining and Processing of Nometal Ores	5363
开采辅助活动	Auxiliary Activities of Mining	
其他采矿业	Mining of Other Ores	

单位：万元（10 000 yuan）

营业成本<br>Cost of Prinipal Business	营业税金及附加<br>Taxes and Extra Charges on Principal Business	利润总额<br>Total Profits	全部从业人员年平均人数（人）<br>Annual Average Employed Persons（person）
**84897026**	**2014374**	**5472206**	**727998**
29900096	1581325	1957865	115832
76721	525	3554	964
16757194	174020	1984935	84978
18033302	210958	1143635	193567
4068953	27422	188633	40668
29187541	288865	1810789	310516
55709485	1725509	3661417	417482
39938250	420207	3207973	242502
20854990	1414279	1154465	204079
24103786	179887	1109768	281417
7094501	435634	300250	36174
4226482	53420	568667	29665
33305141	1153114	2387984	185523
11106717	152662	738352	108231
9256939	59212	437134	112804
6537980	62337	386535	106205
5947041	53607	190415	60620
4395061	20866	217948	50136
2033892	19031	166053	22401
993273	4489	78867	16239
8209	647	327	219
3614	180	236	138

12-5 续表 2
Continued

项目	Indicator	企业单位数（个）<br>Number of Enterprises<br>（Unit）
农副食品加工业	Processing of Food from Agricultural Products	331
食品制造业	Manufacture of Foods	115
酒、饮料和精制茶制造业	Manufacture of Liquor，Beverage and Refind Tea	17
烟草制品业	Manufacture of Tobacco	
纺织业	Manufacture of Textile	93
纺织服装、服饰业	Manufacture of Texitile Wearing Apparel	144
皮革、毛皮、羽毛及其制品和制鞋业	Processing of Leather，Fur，Feather & Its Products Footwear	47
木材加工和木、竹、藤、棕、草制品业	Processing of Timbers，Manufacture of Wood，Bamboo，Rattan，Palm and Straw Products	22
家具制造业	Manufacture of Furniture	64
造纸和纸制品业	Manufacture of Paper and Paper Products	40
印刷和记录媒介复制业	Printing，Reproduction of Recording Media	101
文教、工美、体育和娱乐用品制造业	Manufacture of Articles for Culture，Arts & Crafts，Sports and Entertainment	179
石油、煤炭及其他燃料加工业	Oil, coal and other fuel processing industries	8
化学原料和化学制品制造业	Manufacture of Chemical Raw Material and Chemical Products	171
医药制造业	Manufacture of Medicines	34
化学纤维制造业	Manufacture of Chemical Fibers	8
橡胶和塑料制品业	Manufacture of Rubber and Plastic	252
非金属矿物制品业	Manufacture of Non-mettallic Mineral Products	218
黑色金属冶炼和压延加工业	Smelting and Pressing of Ferrous Metals	30
有色金属冶炼和压延加工业	Smelting and Pressing of Nonferrous Metals	24
金属制品业	Manufacture of Metal Products	278
通用设备制造业	Manufacture of General Purpose Machinery	316
专用设备制造业	Manufacture of Special Purpose Machinery	245
汽车制造业	Manufacture of Vehicle	168
铁路、船舶、航空航天和其他运输设备制造业	Manufacture of Transport Equipment for Railway，Shipping，Aerospace and other uses	155
电气机械和器材制造业	Manufacture of Electrical Machinery & Equipment	186
计算机、通信和其他电子设备制造业	Manufacture of Computer，Communication Equipment and Other Electronic Equipment	114
仪器仪表制造业	Manufacture of Measuring Instrument	49
其他制造业	Manufacture of Other Products	11
废弃资源综合利用业	Recycling and Disposal of Waste Resources	3
金属制品、机械和设备修理业	Manufacture of Metal Products，Machinery and Equipment	6
电力、热力生产和供应业	Production and Supply of Electric Power and Heat Power	44
燃气生产和供应业	Production and Supply of Gas	15
水的生产和供应业	Production and Supply of Water	18

单位：万元（10 000 yuan）

工业总产值<br>Gross Industrial Output Value	资产合计<br>Total Assets	流动资产合计<br>Total Current Assets	固定资产净值<br>Net Value of Fixed Assets
6763720	4240506	2761683	1015009
2393024	3156057	1848791	718607
1619606	2807099	1212427	362699
874484	705756	488720	165930
2300334	1923312	1032906	467933
662706	521843	247719	222816
118525	127740	73870	36167
414226	457452	251217	137395
460542	371427	233008	105567
859851	779983	449480	250686
1176298	1153624	627029	349423
7406847	2977829	1933772	841356
4789079	6193540	3081663	1631150
1047219	1283683	740212	297820
50643	116562	36809	56780
3990200	5308413	2490199	1488952
2652062	3195185	2217490	698298
2824713	2509719	1233477	1184519
606704	535730	164834	240061
3618624	4171674	2428688	809639
4391881	6663964	4210520	1190500
2796824	4071620	2694739	683278
10008249	12909494	11087583	1255367
7824563	12164553	8722924	1869515
15075296	34724824	19485598	2642117
8169914	7413430	5750161	747246
838520	1274076	1004603	121535
130145	241088	200630	24794
10287	24217	9251	10997
19641	26521	8190	12078
1245197	3871872	1185855	2188398
651732	1304253	236884	917424
240264	1335214	446732	538206

12-5 续表 3
Continued

项目	Indicator	营业收入<br>Revenue from Principal Business
农副食品加工业	Processing of Food from Agricultural Products	7674656
食品制造业	Manufacture of Foods	2139842
酒、饮料和精制茶制造业	Manufacture of Liquor，Beverage and Refind Tea	2371302
烟草制品业	Manufacture of Tobacco	
纺织业	Manufacture of Textile	928562
纺织服装、服饰业	Manufacture of Texitile Wearing Apparel	2367809
皮革、毛皮、羽毛及其制品和制鞋业	Processing of Leather，Fur，Feather & Its Products Footwear	627305
木材加工和木、竹、藤、棕、草制品业	Processing of Timbers，Manufacture of Wood，Bamboo，Rattan，Palm and Straw Products	114446
家具制造业	Manufacture of Furniture	415958
造纸和纸制品业	Manufacture of Paper and Paper Products	434191
印刷和记录媒介复制业	Printing，Reproduction of Recording Media	908442
文教、工美、体育和娱乐用品制造业	Manufacture of Articles for Culture，Arts & Crafts，Sports and Entertainment	1168179
石油、煤炭及其他燃料加工业	Oil, coal and other fuel processing industries	7624113
化学原料和化学制品制造业	Manufacture of Chemical Raw Material and Chemical Products	5032020
医药制造业	Manufacture of Medicines	976336
化学纤维制造业	Manufacture of Chemical Fibers	50942
橡胶和塑料制品业	Manufacture of Rubber and Plastic	4069518
非金属矿物制品业	Manufacture of Non-mettallic Mineral Products	2445202
黑色金属冶炼和压延加工业	Smelting and Pressing of Ferrous Metals	3192791
有色金属冶炼和压延加工业	Smelting and Pressing of Nonferrous Metals	621703
金属制品业	Manufacture of Metal Products	3700194
通用设备制造业	Manufacture of General Purpose Machinery	4470508
专用设备制造业	Manufacture of Special Purpose Machinery	2752042
汽车制造业	Manufacture of Vehicle	10234396
铁路、船舶、航空航天和其他运输设备制造业	Manufacture of Transport Equipment for Railway，Shipping，Aerospace and other uses	7925571
电气机械和器材制造业	Manufacture of Electrical Machinery & Equipment	17956053
计算机、通信和其他电子设备制造业	Manufacture of Computer，Communication Equipment and Other Electronic Equipment	9838220
仪器仪表制造业	Manufacture of Measuring Instrument	872255
其他制造业	Manufacture of Other Products	148463
废弃资源综合利用业	Recycling and Disposal of Waste Resources	10119
金属制品、机械和设备修理业	Manufacture of Metal Products，Machinery and Equipment	19420
电力、热力生产和供应业	Production and Supply of Electric Power and Heat Power	1315545
燃气生产和供应业	Production and Supply of Gas	725965
水的生产和供应业	Production and Supply of Water	288820

单位：万元（10 000 yuan）

营业成本<br>Cost of Prinipal Business	营业税金及附加<br>Taxes and Extra Charges on Principal Business	利润总额<br>Total Profits	全部从业人员年平均人数（人）<br>Annual Average Employed Persons（person）
7175212	18717	61212	56912
1756042	15706	23005	17367
1887689	61834	106273	10216
844417	3865	–3952	11691
2080163	21553	54808	57594
559385	5026	5244	21193
102969	792	6679	2446
355129	3727	11259	9950
393858	2850	8805	4404
776375	5489	32966	11405
1026088	9015	18006	25873
5694811	1297296	508530	2850
4300613	24693	241915	24265
483951	13756	171903	9551
41592	509	–1818	586
3458551	24995	149307	43191
2063384	17993	149931	22864
2913693	13386	69835	8419
579032	3559	5851	2990
3280323	28639	255637	40343
3757249	28955	170922	50108
2218938	20100	130301	34150
9073265	91790	581911	49792
6559426	77753	559887	55025
12402164	129033	1432615	68028
8266807	55789	521150	54803
589442	7701	101193	9414
126757	1209	8215	3422
9564	156	–468	189
15024	175	1408	439
1296033	11666	–65923	11350
500633	3547	160150	2476
296625	12272	–5113	4335

12-6 按行业分国有及国有控股工业企业主要指标(2018年)

MAIN INDICATORS OF STATE-OWNED AND STATE-HOLDING INDUSTRIAL ENTERPRISES BY INDUSTRIAL SECTOR (2018)

项目	Indicator	企业单位数(个) Number of Enterprises (Unit)
**总计**	**Total**	**145**
煤炭开采和洗选业	Mining and Washing of Coal	
石油和天然气开采业	Extraction of Petroleum and Natural Gas	
黑色金属矿采选业	Mining of Ferrous Metal Ores	
有色金属矿采选业	Mining of Non-ferrous Metal Ores	1
非金属矿采选业	Mining and Processing of Nometal Ores	1
开采辅助活动	Auxiliary Activities of Mining	
其他采矿业	Mining of Other Ores	
农副食品加工业	Processing of Food from Agricultural Products	1
食品制造业	Manufacture of Foods	3
酒、饮料和精制茶制造业	Manufacture of Liquor, Beverage and Refind Tea	2
烟草制品业	Manufacture of Tobacco	
纺织业	Manufacture of Textile	
纺织服装、服饰业	Manufacture of Textile Wearing Apparel	
皮革、毛皮、羽毛及其制品和制鞋业	Manufacture of Leather, Fur, Feather & Its Products Footwear	1
木材加工和木、竹、藤、棕、草制品业	Processing of Timbers, Manufacture of Wood, Bamboo, Rattan, Palm and Straw Products	1
家具制造业	Manufacture of Furniture	
造纸和纸制品业	Manufacture of Paper and Paper Products	1
印刷和记录媒介复制业	Printing, Reproduction of Recording Media	1
文教、工美、体育和娱乐用品制造业	Manufacture of Articles for Culture, Arts & Crafts, Sports and Entertainment	
石油、煤炭及其他燃料加工业	Oil, coal and other fuel processing industries	3
化学原料和化学制品制造业	Manufacture of Chemical Raw Material and Chemical Products	10
医药制造业	Manufacture of Medicines	2
化学纤维制造业	Manufacture of Chemical Fibers	
橡胶和塑料制品业	Manufacture of Rubber and Plastic	6
非金属矿物制品业	Manufacture of Non-mettallic Mineral Products	10
黑色金属冶炼和压延加工业	Smelting and Pressing of Ferrous Metals	4
有色金属冶炼和压延加工业	Smelting and Pressing of Non-ferrous Metals	5
金属制品业	Manufacture of Metal Products	4
通用设备制造业	Manufacture of General Purpose Machinery	8
专用设备制造业	Manufacture of Special Purpose Machinery	5
汽车制造业	Manufacture of Vehicle	6
铁路、船舶、航空航天和其他运输设备制造业	Manufacture of Transport Equipment for Railway, Shipping, Aerospace and other uses	17
电气机械和器材制造业	Manufacture of Electrical Machinery & Equipment	5
计算机、通信和其他电子设备制造业	Manufacture of Computer, Communication Equipment and Other Electronic Equipment	3
仪器仪表制造业	Manufacture of Measuring Instrument	2
其他制造业	Manufacture of Other Products	1
废弃资源综合利用业	Recycling and Disposal of Waste Resources	
金属制品、机械和设备修理业	Manufacture of Metal Products, Machinery and Equipment	1
电力、热力生产和供应业	Production and Supply of Electric Power and Heat Power	23
燃气生产和供应业	Production and Supply of Gas	6
水的生产和供应业	Production and Supply of Water	12

单位：万元（10 000 yuan）

工业总产值<br>Gross Industrial Output Value	资产合计<br>Total Assets	流动资产合计<br>Total Current Assets	固定资产净值<br>Net Value of Fixed Assets
**32973045**	**32973045**	**27281061**	**8800214**
12298	67057	2451	50918
4395	10223	6909	1361
6086	6350	3238	2606
63008	87514	71264	3067
1038283	2293510	877507	223940
9381	14919	12083	1582
10911	22834	20748	1556
124288	62276	34758	15064
4960	11137	4891	5614
7232378	2847158	1870482	784804
1109007	1964108	605272	722959
265415	233159	138196	56415
546504	1549348	778706	394322
521436	359366	273148	49006
1891137	1800778	752939	1007910
119879	339538	47988	185767
19464	87068	33891	43162
225497	329635	223630	91716
168068	339521	174737	116118
5712097	8489024	7862306	405408
6317587	9877053	7127821	1461991
712176	1057699	709251	59362
5257778	5348439	4301947	319572
12850	17406	15588	502
14440	42870	36523	2882
4542	2747	2699	46
1036493	2728776	816685	1638534
341023	868103	121012	656541
191663	1170820	354393	497491

12-6 续表
Continued

项目	Indicator	营业收入<br>Revenue from Principal Business
**总计**	**Total**	**35976282**
煤炭开采和洗选业	Mining and Washing of Coal	
石油和天然气开采业	Extraction of Petroleum and Natural Gas	
黑色金属矿采选业	Mining of Ferrous Metal Ores	
有色金属矿采选业	Mining of Non-ferrous Metal Ores	12810
非金属矿采选业	Mining and Processing of Nometal Ores	5363
开采辅助活动	Auxiliary Activities of Mining	
其他采矿业	Mining of Other Ores	
农副食品加工业	Processing of Food from Agricultural Products	7798
食品制造业	Manufacture of Foods	65301
酒、饮料和精制茶制造业	Manufacture of Liquor, Beverage and Refind Tea	1922370
烟草制品业	Manufacture of Tobacco	
纺织业	Manufacture of Textile	
纺织服装、服饰业	Manufacture of Textile Wearing Apparel	
皮革、毛皮、羽毛及其制品和制鞋业	Manufacture of Leather, Fur, Feather & Its Products Footwear	8634
木材加工和木、竹、藤、棕、草制品业	Processing of Timbers, Manufacture of Wood, Bamboo, Rattan, Palm and Straw Products	10091
家具制造业	Manufacture of Furniture	
造纸和纸制品业	Manufacture of Paper and Paper Products	103098
印刷和记录媒介复制业	Printing, Reproduction of Recording Media	4351
文教、工美、体育和娱乐用品制造业	Manufacture of Articles for Culture, Arts & Crafts, Sports and Entertainment	
石油、煤炭及其他燃料加工业	Oil, coal and other fuel processing industries	7433339
化学原料和化学制品制造业	Manufacture of Chemical Raw Material and Chemical Products	1073477
医药制造业	Manufacture of Medicines	238740
化学纤维制造业	Manufacture of Chemical Fibers	
橡胶和塑料制品业	Manufacture of Rubber and Plastic	542980
非金属矿物制品业	Manufacture of Non-mettallic Mineral Products	235788
黑色金属冶炼和压延加工业	Smelting and Pressing of Ferrous Metals	2033037
有色金属冶炼和压延加工业	Smelting and Pressing of Non-ferrous Metals	120983
金属制品业	Manufacture of Metal Products	19584
通用设备制造业	Manufacture of General Purpose Machinery	187513
专用设备制造业	Manufacture of Special Purpose Machinery	148195
汽车制造业	Manufacture of Vehicle	5927742
铁路、船舶、航空航天和其他运输设备制造业	Manufacture of Transport Equipment for Railway, Shipping, Aerospace and other uses	6249719
电气机械和器材制造业	Manufacture of Electrical Machinery & Equipment	1101055
计算机、通信和其他电子设备制造业	Manufacture of Computer, Communication Equipment and Other Electronic Equipment	6779254
仪器仪表制造业	Manufacture of Measuring Instrument	14557
其他制造业	Manufacture of Other Products	31559
废弃资源综合利用业	Recycling and Disposal of Waste Resources	
金属制品、机械和设备修理业	Manufacture of Metal Products, Machinery and Equipment	4542
电力、热力生产和供应业	Production and Supply of Electric Power and Heat Power	1076889
燃气生产和供应业	Production and Supply of Gas	377026
水的生产和供应业	Production and Supply of Water	240487

单位：万元（10 000 yuan）

营业成本<br>Cost of Prinipal Business	营业税金及附加<br>Taxes and Extra Charges on Principal Business	利润总额<br>Total Profits	全部从业人员年平均人数（人）<br>Annual Average Employed Persons（person）
**29900096**	**1581325**	**1957865**	**115832**
8209	647	327	219
3614	180	236	138
7604	39	-144	57
47807	669	9554	756
1538507	53685	96327	6135
5451	148	287	165
9278	34	494	457
99333	540	74	950
3293	121	11	145
5524932	1295281	501076	2382
909460	4805	91423	3406
108176	2565	59931	1500
485298	3661	-8507	6627
202689	1226	15569	1260
1839441	10742	39459	4230
98526	2522	2758	1272
16691	435	12	400
177424	1387	-13722	2240
130026	783	16	1058
5267881	70259	343773	8652
5220340	59810	434379	30908
979320	5676	8153	6346
5586546	42325	342533	22346
11002	140	1091	166
29388	88	649	112
3904	23	178	42
1110837	8753	-91863	8895
207780	3123	139346	1054
267343	11662	-15555	3914

12-7　按行业分规模以上外商投资和港澳台商投资工业企业主要指标（2018 年）

MAIN INDICATORS OF FOREIGN FUNEDE ENTERPRISES AND ENTERPRISES WITH FUNDS FROM HONG HONG，MACAO AND TAIWAN ABOVE DESIGNATED SIZE BY INDUSTRIAL SECTOR（2018）

项目	Indicator	企业单位数（个）Number of Enterprises（Unit）
**总计**	**Total**	**938**
煤炭开采和洗选业	Mining and Washing of Coal	
石油和天然气开采业	Extraction of Petroleum and Natural Gas	
黑色金属矿采选业	Mining of Ferrous Metal Ores	
有色金属矿采选业	Mining of Non-ferrous Metal Ores	
非金属矿采选业	Mining and Processing of Nometal Ores	
开采辅助活动	Auxiliary Activities of Mining	
其他采矿业	Mining of Other Ores	
农副食品加工业	Processing of Food from Agricultural Products	98
食品制造业	Manufacture of Foods	39
酒、饮料和精制茶制造业	Manufacture of Liquor，Beverage and Refind Tea	8
烟草制品业	Manufacture of Tobacco	
纺织业	Manufacture of Textile	35
纺织服装、服饰业	Manufacture of Textile Wearing Apparel	62
皮革、毛皮、羽毛及其制品和制鞋业	Manufacture of Leather，Fur，Feather & Its Products Footwear	27
木材加工和木、竹、藤、棕、草制品业	Processing of Timbers，Manufacture of Wood，Bamboo，Rattan，Palm and Straw Products	5
家具制造业	Manufacture of Furniture	21
造纸和纸制品业	Manufacture of Paper and Paper Products	13
印刷和记录媒介复制业	Printing，Reproduction of Recording Media	26
文教、工美、体育和娱乐用品制造业	Manufacture of Articles for Culture，Arts & Crafts，Sports and Entertainment	57
石油、煤炭及其他燃料加工业	Oil, coal and other fuel processing industries	1
化学原料和化学制品制造业	Manufacture of Chemical Raw Material and Chemical Products	56
医药制造业	Manufacture of Medicines	6
化学纤维制造业	Manufacture of Chemical Fibers	3
橡胶和塑料制品业	Manufacture of Rubber and Plastic	54
非金属矿物制品业	Manufacture of Non-mettallic Mineral Products	36
黑色金属冶炼和压延加工业	Smelting and Pressing of Ferrous Metals	8
有色金属冶炼和压延加工业	Smelting and Pressing of Non-ferrous Metals	2
金属制品业	Manufacture of Metal Products	59
通用设备制造业	Manufacture of General Purpose Machinery	62
专用设备制造业	Manufacture of Special Purpose Machinery	43
汽车制造业	Manufacture of Vehicle	57
铁路、船舶、航空航天和其他运输设备制造业	Manufacture of Transport Equipment for Railway，Shipping，Aerospace and other uses	30
电气机械和器材制造业	Manufacture of Electrical Machinery & Equipment	43
计算机、通信和其他电子设备制造业	Manufacture of Computer，Communication Equipment and Other Electronic Equipment	54
仪器仪表制造业	Manufacture of Measuring Instrument	13
其他制造业	Manufacture of Other Products	5
废弃资源综合利用业	Recycling and Disposal of Waste Resources	
金属制品、机械和设备修理业	Manufacture of Metal Products，Machinery and Equipment	1
电力、热力生产和供应业	Production and Supply of Electric Power and Heat Power	4
燃气生产和供应业	Production and Supply of Gas	7
水的生产和供应业	Production and Supply of Water	3

单位：万元（10 000 yuan）

工业总产值<br>Gross Industrial Output Value	资产合计<br>Total Assets	流动资产合计<br>Total Current Assets	固定资产净值<br>Net Value of Fixed Assets
**24092865**	**27642336**	**19200472**	**5442126**
2176535	1707105	1292512	308419
1570361	1919797	1250215	431368
1184541	2446758	969369	278217
478805	364861	247938	99505
650510	582914	375573	137911
473560	232037	157718	60297
28558	35157	26534	7889
183671	196138	130569	53259
175872	160779	115408	38165
396696	414713	251096	124406
447802	470222	291349	137027
68377	30327	17959	7814
2114387	1889180	1071410	644777
176859	139820	80232	12238
31448	48768	28412	21759
935560	989811	495929	421041
475963	611717	362857	213079
389602	255770	185131	55707
50032	50440	35296	15299
1651556	1384669	941731	346924
1496323	1405849	924210	342102
634297	931859	637110	208854
4022309	7197708	6507805	545045
636189	955287	709909	163421
875948	686965	472688	158209
1942521	1313371	941643	272639
332494	341567	305802	19573
87398	173690	143583	19131
6290	2682	2612	50
34188	143303	43254	82225
334202	423121	145614	175917
30013	135956	39004	39862

12-7 续表
Continued

项目	Indicator	营业收入<br>Revenue from Principal Business
**总计**	**Total**	**25982220**
煤炭开采和洗选业	Mining and Washing of Coal	
石油和天然气开采业	Extraction of Petroleum and Natural Gas	
黑色金属矿采选业	Mining of Ferrous Metal Ores	
有色金属矿采选业	Mining of Non-ferrous Metal Ores	
非金属矿采选业	Mining and Processing of Nometal Ores	
开采辅助活动	Auxiliary Activities of Mining	
其他采矿业	Mining of Other Ores	
农副食品加工业	Processing of Food from Agricultural Products	2660511
食品制造业	Manufacture of Foods	1399184
酒、饮料和精制茶制造业	Manufacture of Liquor，Beverage and Refind Tea	2140361
烟草制品业	Manufacture of Tobacco	
纺织业	Manufacture of Textile	516162
纺织服装、服饰业	Manufacture of Textile Wearing Apparel	668251
皮革、毛皮、羽毛及其制品和制鞋业	Manufacture of Leather，Fur，Feather & Its Products Footwear	471941
木材加工和木、竹、藤、棕、草制品业	Processing of Timbers，Manufacture of Wood，Bamboo，Rattan，Palm and Straw Products	26808
家具制造业	Manufacture of Furniture	176432
造纸和纸制品业	Manufacture of Paper and Paper Products	173936
印刷和记录媒介复制业	Printing，Reproduction of Recording Media	443396
文教、工美、体育和娱乐用品制造业	Manufacture of Articles for Culture，Arts & Crafts，Sports and Entertainment	452730
石油、煤炭及其他燃料加工业	Oil, coal and other fuel processing industries	68737
化学原料和化学制品制造业	Manufacture of Chemical Raw Material and Chemical Products	2300470
医药制造业	Manufacture of Medicines	163309
化学纤维制造业	Manufacture of Chemical Fibers	31157
橡胶和塑料制品业	Manufacture of Rubber and Plastic	947801
非金属矿物制品业	Manufacture of Non-mettallic Mineral Products	498319
黑色金属冶炼和压延加工业	Smelting and Pressing of Ferrous Metals	392840
有色金属冶炼和压延加工业	Smelting and Pressing of Non-ferrous Metals	50266
金属制品业	Manufacture of Metal Products	1674628
通用设备制造业	Manufacture of General Purpose Machinery	1555635
专用设备制造业	Manufacture of Special Purpose Machinery	639414
汽车制造业	Manufacture of Vehicle	4050052
铁路、船舶、航空航天和其他运输设备制造业	Manufacture of Transport Equipment for Railway，Shipping，Aerospace and other uses	710419
电气机械和器材制造业	Manufacture of Electrical Machinery & Equipment	828590
计算机、通信和其他电子设备制造业	Manufacture of Computer，Communication Equipment and Other Electronic Equipment	2085001
仪器仪表制造业	Manufacture of Measuring Instrument	342157
其他制造业	Manufacture of Other Products	88590
废弃资源综合利用业	Recycling and Disposal of Waste Resources	
金属制品、机械和设备修理业	Manufacture of Metal Products，Machinery and Equipment	6290
电力、热力生产和供应业	Production and Supply of Electric Power and Heat Power	35001
燃气生产和供应业	Production and Supply of Gas	353859
水的生产和供应业	Production and Supply of Water	29974

单位：万元（10 000 yuan）

营业成本<br>Cost of Prinipal Business	营业税金及附加<br>Taxes and Extra Charges on Principal Business	利润总额<br>Total Profits	全部从业人员年平均人数（人）<br>Annual Average Employed Persons（person）
**22102255**	**238380**	**1332268**	**234235**
2483635	6246	19141	20682
1154959	10601	–33209	8702
1700456	55520	99489	7536
459422	2039	4860	5428
594004	5452	5189	18792
428875	3902	863	15337
23369	118	1069	740
145553	1704	7570	4651
156254	983	5570	1551
364615	3175	23082	5200
389672	3849	11088	11973
62327	254	1364	136
1994210	10315	62045	9062
94055	2709	22367	1175
24830	217	–3261	268
783653	6069	50297	11646
408813	4559	34636	4651
374210	805	10699	1269
47690	163	–1269	145
1509460	11077	178195	15546
1286915	8286	86751	16575
516801	3520	23009	6940
3363251	70865	351157	20472
556281	7866	60794	7154
709667	3421	44780	10462
1812082	9011	158029	20567
216707	3110	71400	2874
75649	888	3585	2793
3706	63	1019	127
22693	585	7099	312
319202	492	19458	1160
19242	519	5403	309

12-8　按行业分大中型工业企业主要指标 (2018 年)

MAIN INDICATORS OF LARGE AND MEDIUM-SIZED INDUSTRIAL ENTERPRISES BY INDUSTRIAL SECTOR（2019）

项目	Indicator	企业单位数 (个) Number of Enterprises (Unit)
**总计**	**Total**	**459**
煤炭开采和洗选业	Mining and Washing of Coal	
石油和天然气开采业	Extraction of Petroleum and Natural Gas	
黑色金属矿采选业	Mining of Ferrous Metal Ores	
有色金属矿采选业	Mining of Non-ferrous Metal Ores	
非金属矿采选业	Mining and Processing of Nometal Ores	
开采辅助活动	Auxiliary Activities of Mining	
其他采矿业	Mining of Other Ores	
农副食品加工业	Processing of Food from Agricultural Products	32
食品制造业	Manufacture of Foods	19
酒、饮料和精制茶制造业	Manufacture of Liquor，Beverage and Refind Tea	4
烟草制品业	Manufacture of Tobacco	
纺织业	Manufacture of Textile	7
纺织服装、服饰业	Manufacture of Textile Wearing Apparel	32
皮革、毛皮、羽毛及其制品和制鞋业	Manufacture of Leather，Fur，Feather & Its Products Footwear	13
木材加工和木、竹、藤、棕、草制品业	Processing of Timbers，Manufacture of Wood，Bamboo，Rattan，Palm and Straw Products	2
家具制造业	Manufacture of Furniture	8
造纸和纸制品业	Manufacture of Paper and Paper Products	1
印刷和记录媒介复制业	Printing，Reproduction of Recording Media	7
文教、工美、体育和娱乐用品制造业	Manufacture of Articles for Culture，Arts & Crafts，Sports and Entertainment	17
石油、煤炭及其他燃料加工业	Oil, coal and other fuel processing industries	3
化学原料和化学制品制造业	Manufacture of Chemical Raw Material and Chemical Products	18
医药制造业	Manufacture of Medicines	10
化学纤维制造业	Manufacture of Chemical Fibers	
橡胶和塑料制品业	Manufacture of Rubber and Plastic	25
非金属矿物制品业	Manufacture of Non-mettallic Mineral Products	16
黑色金属冶炼和压延加工业	Smelting and Pressing of Ferrous Metals	5
有色金属冶炼和压延加工业	Smelting and Pressing of Non-ferrous Metals	2
金属制品业	Manufacture of Metal Products	29
通用设备制造业	Manufacture of General Purpose Machinery	37
专用设备制造业	Manufacture of Special Purpose Machinery	23
汽车制造业	Manufacture of Vehicle	37
铁路、船舶、航空航天和其他运输设备制造业	Manufacture of Transport Equipment for Railway，Shipping，Aerospace and other uses	32
电气机械和器材制造业	Manufacture of Electrical Machinery & Equipment	31
计算机、通信和其他电子设备制造业	Manufacture of Computer，Communication Equipment and Other Electronic Equipment	26
仪器仪表制造业	Manufacture of Measuring Instrument	6
其他制造业	Manufacture of Other Products	1
废弃资源综合利用业	Recycling and Disposal of Waste Resources	
金属制品、机械和设备修理业	Manufacture of Metal Products，Machinery and Equipment	
电力、热力生产和供应业	Production and Supply of Electric Power and Heat Power	11
燃气生产和供应业	Production and Supply of Gas	2
水的生产和供应业	Production and Supply of Water	3

单位：万元（10 000 yuan）

工业总产值<br>Gross Industrial Output Value	资产合计<br>Total Assets	流动资产合计<br>Total Current Assets	固定资产净值<br>Net Value of Fixed Assets
**69216338**	**97151438**	**60602913**	**15006929**
2953132	1910761	1241876	468867
1545762	2234917	1361631	408545
1498188	2594251	1098266	287897
352424	213195	174224	27847
1759197	1354481	682090	331538
526353	255786	169261	63319
18187	29087	22159	2181
158046	171264	117299	45646
124288	62276	34758	15064
196071	157251	113289	40002
363909	344654	199503	95787
7232378	2847158	1870482	784804
2969413	3858657	1846949	1117444
883218	994248	595531	192583
2249375	3857330	1599260	1179538
878149	965338	688049	175618
1922189	2112626	963013	1083157
42659	221258	8239	133406
1774292	1493647	1026625	322447
2522728	4005134	2531412	552268
1326014	1410420	1096445	240432
8786414	11772188	10342009	983280
6913738	10794895	7764214	1611676
13616022	32572128	18160737	2224061
6847403	6348404	4983082	541088
485101	790823	668528	56768
70754	88028	59384	18191
895282	2400093	828978	1385985
199774	455110	109155	267667
105880	836032	246466	349825

12-8 续表
Continued

项目	Indicator	营业收入<br>Revenue from Principal Business
**总计**	**Total**	**75470442**
煤炭开采和洗选业	Mining and Washing of Coal	
石油和天然气开采业	Extraction of Petroleum and Natural Gas	
黑色金属矿采选业	Mining of Ferrous Metal Ores	
有色金属矿采选业	Mining of Non-ferrous Metal Ores	
非金属矿采选业	Mining and Processing of Nometal Ores	
开采辅助活动	Auxiliary Activities of Mining	
其他采矿业	Mining of Other Ores	
农副食品加工业	Processing of Food from Agricultural Products	3603765
食品制造业	Manufacture of Foods	1329906
酒、饮料和精制茶制造业	Manufacture of Liquor，Beverage and Refind Tea	2216301
烟草制品业	Manufacture of Tobacco	
纺织业	Manufacture of Textile	336037
纺织服装、服饰业	Manufacture of Textile Wearing Apparel	1803238
皮革、毛皮、羽毛及其制品和制鞋业	Manufacture of Leather，Fur，Feather & Its Products Footwear	495194
木材加工和木、竹、藤、棕、草制品业	Processing of Timbers，Manufacture of Wood，Bamboo，Rattan，Palm and Straw Products	17498
家具制造业	Manufacture of Furniture	157752
造纸和纸制品业	Manufacture of Paper and Paper Products	103098
印刷和记录媒介复制业	Printing，Reproduction of Recording Media	201446
文教、工美、体育和娱乐用品制造业	Manufacture of Articles for Culture，Arts & Crafts，Sports and Entertainment	376233
石油、煤炭及其他燃料加工业	Oil, coal and other fuel processing industries	7433339
化学原料和化学制品制造业	Manufacture of Chemical Raw Material and Chemical Products	3069429
医药制造业	Manufacture of Medicines	811344
化学纤维制造业	Manufacture of Chemical Fibers	
橡胶和塑料制品业	Manufacture of Rubber and Plastic	2240952
非金属矿物制品业	Manufacture of Non-mettallic Mineral Products	628978
黑色金属冶炼和压延加工业	Smelting and Pressing of Ferrous Metals	2091672
有色金属冶炼和压延加工业	Smelting and Pressing of Non-ferrous Metals	43477
金属制品业	Manufacture of Metal Products	1793764
通用设备制造业	Manufacture of General Purpose Machinery	2575389
专用设备制造业	Manufacture of Special Purpose Machinery	1346653
汽车制造业	Manufacture of Vehicle	9018577
铁路、船舶、航空航天和其他运输设备制造业	Manufacture of Transport Equipment for Railway，Shipping，Aerospace and other uses	6909697
电气机械和器材制造业	Manufacture of Electrical Machinery & Equipment	16533556
计算机、通信和其他电子设备制造业	Manufacture of Computer，Communication Equipment and Other Electronic Equipment	8449405
仪器仪表制造业	Manufacture of Measuring Instrument	482968
其他制造业	Manufacture of Other Products	71114
废弃资源综合利用业	Recycling and Disposal of Waste Resources	
金属制品、机械和设备修理业	Manufacture of Metal Products，Machinery and Equipment	
电力、热力生产和供应业	Production and Supply of Electric Power and Heat Power	945232
燃气生产和供应业	Production and Supply of Gas	239876
水的生产和供应业	Production and Supply of Water	144555

单位：万元（10 000 yuan）

营业成本<br>Cost of Prinipal Business	营业税金及附加<br>Taxes and Extra Charges on Principal Business	利润总额<br>Total Profits	全部从业人员年平均人数（人）<br>Annual Average Employed Persons（person）
**60793240**	**1834487**	**4362438**	**44658**
3398260	6593	25180	3448
1083843	8690	–19421	939
1777589	58437	102239	857
304711	707	–763	345
1574265	17878	53071	4541
444225	4013	5044	1684
16447	117	506	101
128351	1666	7000	415
99333	540	74	95
159385	1606	13805	322
323216	3022	8338	869
5524932	1295281	501076	238
2658087	10874	156540	1129
379719	11966	160323	674
1839384	13521	106992	2362
518213	4354	45277	806
1854606	11621	56443	634
27665	1408	2288	120
1578695	14199	208502	1920
2196752	15276	94029	2291
1078770	7712	77820	1537
8031771	82673	544114	3500
5735813	67933	477315	4403
11226499	120859	1361529	5260
7089309	49834	414364	4284
332109	4475	52088	421
61963	653	1210	240
975648	8282	–84253	823
191015	370	6027	116
182668	9928	–14320	287

12-9　按行业分规模以上工业企业主要经济效益指标（2018 年）

MAIN INDICATORS ON ECONOMIC BENEFIT OF INDUSTRIA ENTERPRISES ABOVE DESIGNATED SIZE BY INDOUSTRIAL SSECTOR（2018）

项目	Indicator	总资产贡献率(%)<br>Ratio of Total Assets to Industrial Output Value<br>（%）
**总计**	**Total**	**8.11**
煤炭开采和洗选业	Mining and Washing of Coal	
石油和天然气开采业	Extraction of Petroleum and Natural Gas	
黑色金属矿采选业	Mining of Ferrous Metal Ores	
有色金属矿采选业	Mining of Non-ferrous Metal Ores	2.9
非金属矿采选业	Mining and Processing of Nometal Ores	8.49
开采辅助活动	Auxiliary Activities of Mining	
其他采矿业	Mining of Other Ores	
农副食品加工业	Processing of Food from Agricultural Products	4.02
食品制造业	Manufacture of Foods	3.07
酒、饮料和精制茶制造业	Manufacture of Liquor，Beverage and Refind Tea	8.98
烟草制品业	Manufacture of Tobacco	
纺织业	Manufacture of Textile	2.47
纺织服装、服饰业	Manufacture of Textile Wearing Apparel	8.39
皮革、毛皮、羽毛及其制品和制鞋业	Manufacture of Leather，Fur，Feather & Its Products Footwear	3.81
木材加工和木、竹、藤、棕、草制品业	Processing of Timbers，Manufacture of Wood，Bamboo，Rattan，Palm and Straw Products	6.79
家具制造业	Manufacture of Furniture	5.59
造纸和纸制品业	Manufacture of Paper and Paper Products	6.17
印刷和记录媒介复制业	Printing，Reproduction of Recording Media	8.26
文教、工美、体育和娱乐用品制造业	Manufacture of Articles for Culture，Arts & Crafts，Sports and Entertainment	4.05
石油、煤炭及其他燃料加工业	Oil, coal and other fuel processing industries	65.3
化学原料和化学制品制造业	Manufacture of Chemical Raw Material and Chemical Products	6.42
医药制造业	Manufacture of Medicines	20.43
化学纤维制造业	Manufacture of Chemical Fibers	-0.07
橡胶和塑料制品业	Manufacture of Rubber and Plastic	5.41
非金属矿物制品业	Manufacture of Non-mettallic Mineral Products	7.94
黑色金属冶炼和压延加工业	Smelting and Pressing of Ferrous Metals	5.18
有色金属冶炼和压延加工业	Smelting and Pressing of Non-ferrous Metals	3.53
金属制品业	Manufacture of Metal Products	8.95
通用设备制造业	Manufacture of General Purpose Machinery	4.43
专用设备制造业	Manufacture of Special Purpose Machinery	5.74
汽车制造业	Manufacture of Vehicle	6.6
铁路、船舶、航空航天和其他运输设备制造业	Manufacture of Transport Equipment for Railway，Shipping，Aerospace and other uses	7.8
电气机械和器材制造业	Manufacture of Electrical Machinery & Equipment	6.34
计算机、通信和其他电子设备制造业	Manufacture of Computer，Communication Equipment and Other Electronic Equipment	12.83
仪器仪表制造业	Manufacture of Measuring Instrument	11.46
其他制造业	Manufacture of Other Products	7.14
废弃资源综合利用业	Recycling and Disposal of Waste Resources	-0.1
金属制品、机械和设备修理业	Manufacture of Metal Products，Machinery and Equipment	9.22
电力、热力生产和供应业	Production and Supply of Electric Power and Heat Power	-0.09
燃气生产和供应业	Production and Supply of Gas	15.15
水的生产和供应业	Production and Supply of Water	2.17

资产负债率 (%) Assets–Liability Ratio (%)	流动资产周转次数(次/年) Number of Times of Annual of Turnover Current Assets (time/year)	工业成本费用利润率 (%) Ratio of Profits to Industrial Costs (%)	产品销售率 (%) Ratio of Sales to Output (%)
**61.53**	**1.32**	**5.62**	**100.18**
50.97	5.23	2.75	100
80.22	0.78	4.89	88.39
65.3	2.78	0.81	98.86
62.68	1.16	1.12	95.86
41.89	1.96	4.58	161.07
73.27	1.9	–0.43	99.03
52.53	2.29	2.38	99.24
60.87	2.53	0.85	94.57
50.6	1.55	6.03	99.97
61.09	1.66	2.8	98.18
61.79	1.86	2.07	98.01
58.7	2.02	3.78	98.42
51.92	1.86	1.58	99.83
55.13	3.94	8.74	99.62
52	1.63	5.05	98.35
32.81	1.32	21.33	96.46
56.05	1.38	–3.62	98.63
59.26	1.63	3.8	99.34
60.56	1.1	6.53	99.33
71.72	2.59	2.24	99.95
69.19	3.77	0.95	100.16
48.33	1.52	7.11	99.72
53.47	1.06	3.97	98.21
54.89	1.02	4.99	97.99
66.57	0.92	6.03	98.97
62.99	0.91	7.45	101.6
68.98	0.92	8.53	96.54
51.34	1.71	5.52	103.73
60.1	0.87	13.39	101.73
61.19	0.74	5.86	99.73
48.37	1.09	–4.49	98.88
24.65	2.37	7.89	98.77
75.03	1.11	–4.64	97.76
43.27	3.06	27.57	99.86
65.91	0.65	–1.49	99.34

12-10 按行业分国有控股工业企业主要经济效益指标（2018 年）
MAIN INDICATORS ON ECONOMIC BENEFIT OF STSTE-OWNED AND STATE-HOLDING INUSTRIAL ENTERPRISES BY INDUSTRIAL SECTOR（2018）

行业	Indicator	总资产贡献率(%) Ratio of Total Assets to Industrial Output Value (%)
**总计**	**Total**	**11.06**
煤炭开采和洗选业	Mining and Washing of Coal	
石油和天然气开采业	Extraction of Petroleum and Natural Gas	
黑色金属矿采选业	Mining of Ferrous Metal Ores	
有色金属矿采选业	Mining of Non-ferrous Metal Ores	2.90
非金属矿采选业	Mining and Processing of Nometal Ores	8.49
开采辅助活动	Auxiliary Activities of Mining	0.00
其他采矿业	Mining of Other Ores	0.00
农副食品加工业	Processing of Food from Agricultural Products	-1.38
食品制造业	Manufacture of Foods	14.83
酒、饮料和精制茶制造业	Manufacture of Liquor，Beverage and Refind Tea	9.31
烟草制品业	Manufacture of Tobacco	
纺织业	Manufacture of Textile	
纺织服装、服饰业	Manufacture of Textile Wearing Apparel	
皮革、毛皮、羽毛及其制品和制鞋业	Manufacture of Leather，Fur，Feather & Its Products Footwear	
木材加工和木、竹、藤、棕、草制品业	Processing of Timbers，Manufacture of Wood，Bamboo，Rattan，Palm and Straw Products	5.74
家具制造业	Manufacture of Furniture	0.84
造纸和纸制品业	Manufacture of Paper and Paper Products	3.17
印刷和记录媒介复制业	Printing，Reproduction of Recording Media	4.10
文教、工美、体育和娱乐用品制造业	Manufacture of Articles for Culture，Arts & Crafts，Sports and Entertainment	
石油、煤炭及其他燃料加工业	Oil, coal and other fuel processing industries	67.91
化学原料和化学制品制造业	Manufacture of Chemical Raw Material and Chemical Products	5.63
医药制造业	Manufacture of Medicines	32.80
化学纤维制造业	Manufacture of Chemical Fibers	
橡胶和塑料制品业	Manufacture of Rubber and Plastic	1.44
非金属矿物制品业	Manufacture of Non-mettallic Mineral Products	7.46
黑色金属冶炼和压延加工业	Smelting and Pressing of Ferrous Metals	4.90
有色金属冶炼和压延加工业	Smelting and Pressing of Non-ferrous Metals	2.94
金属制品业	Manufacture of Metal Products	2.40
通用设备制造业	Manufacture of General Purpose Machinery	-2.23
专用设备制造业	Manufacture of Special Purpose Machinery	1.69
汽车制造业	Manufacture of Vehicle	5.98
铁路、船舶、航空航天和其他运输设备制造业	Manufacture of Transport Equipment for Railway，Shipping，Aerospace and other uses	7.45
电气机械和器材制造业	Manufacture of Electrical Machinery & Equipment	3.64
计算机、通信和其他电子设备制造业	Manufacture of Computer，Communication Equipment and Other Electronic Equipment	13.56
仪器仪表制造业	Manufacture of Measuring Instrument	9.49
其他制造业	Manufacture of Other Products	4.06
废弃资源综合利用业	Recycling and Disposal of Waste Resources	
金属制品、机械和设备修理业	Manufacture of Metal Products，Machinery and Equipment	12.66
电力、热力生产和供应业	Production and Supply of Electric Power and Heat Power	-1.78
燃气生产和供应业	Production and Supply of Gas	19.69
水的生产和供应业	Production and Supply of Water	1.09

资产负债率 (%)<br>Assets–Liability Ratio<br>（%）	流动资产周转次数(次/年)<br>Number of Times of Annual of<br>Turnover Current Assets（time/year）	工业成本费用利润率 (%)<br>Ratio of Profits to Industrial<br>Costs（%）	产品销售率 (%)<br>Ratio of Sales to Output<br>（%）
**62.46**	**1.32**	**5.91**	**103.55**
50.97	5.23	2.75	100
80.22	0.78	4.89	88.39
98.54	2.41	–1.83	100
31.94	0.92	17.31	93.23
35.02	2.19	5.09	194.21
57.72	0.71	3.50	100.11
87.26	0.49	5.16	100
61.91	2.97	0.07	96
10.38	0.89	0.26	87.72
55.37	3.97	8.89	99.62
56.51	1.77	9.24	93.76
24.09	1.73	31.97	101.49
78.96	0.70	–1.49	99.9
71.92	0.86	7.07	100.17
80.22	2.70	1.99	99.02
74.15	2.52	2.37	95.44
72.28	0.58	0.06	100.29
56.64	0.84	–7.05	79.67
79.28	0.85	0.01	85.73
67.11	0.75	6.23	100.85
65.04	0.88	7.26	101.29
65.60	1.55	0.75	101.35
51.16	1.58	5.22	105.23
58.42	0.93	8.27	100.83
75.43	0.86	2.09	100
81.50	1.68	4.10	100
81.15	1.32	–7.66	97.48
30.58	3.12	57.36	99.9
67.40	0.68	–5.08	99.74

12-11 按行业分规模以上外商及港澳台商投资工业企业主要经济效益指标（2018 年）

MAIN INDICATORS ON ECONOMIC BENEFIT OF FOREIGN FUNDED ENTERPRISES AND ENTERPRISES WITH FUNDS FROM HONG KONG，MACAO AND TAIWAN ABOVE DESIGNATED SIZE BY INDUSTRIAL SECTOR（2018）

行业	Indicator	总资产贡献率（%）<br>Ratio of Total Assets to Industrial Output Value<br>（%）
**总计**	**Total**	**7.77**
煤炭开采和洗选业	Mining and Washing of Coal	
石油和天然气开采业	Extraction of Petroleum and Natural Gas	
黑色金属矿采选业	Mining of Ferrous Metal Ores	
有色金属矿采选业	Mining of Non-ferrous Metal Ores	
非金属矿采选业	Mining and Processing of Nometal Ores	
开采辅助活动	Auxiliary Activities of Mining	
其他采矿业	Mining of Other Ores	
农副食品加工业	Processing of Food from Agricultural Products	3.32
食品制造业	Manufacture of Foods	0.88
酒、饮料和精制茶制造业	Manufacture of Liquor，Beverage and Refind Tea	9.38
烟草制品业	Manufacture of Tobacco	
纺织业	Manufacture of Textile	4.23
纺织服装、服饰业	Manufacture of Textile Wearing Apparel	3.03
皮革、毛皮、羽毛及其制品和制鞋业	Manufacture of Leather，Fur，Feather & Its Products Footwear	4.40
木材加工和木、竹、藤、棕、草制品业	Processing of Timbers，Manufacture of Wood，Bamboo，Rattan，Palm and Straw Products	2.64
家具制造业	Manufacture of Furniture	7.68
造纸和纸制品业	Manufacture of Paper and Paper Products	6.75
印刷和记录媒介复制业	Printing，Reproduction of Recording Media	9.50
文教、工美、体育和娱乐用品制造业	Manufacture of Articles for Culture，Arts & Crafts，Sports and Entertainment	4.35
石油、煤炭及其他燃料加工业	Oil, coal and other fuel processing industries	6.75
化学原料和化学制品制造业	Manufacture of Chemical Raw Material and Chemical Products	7.23
医药制造业	Manufacture of Medicines	29.55
化学纤维制造业	Manufacture of Chemical Fibers	-4.39
橡胶和塑料制品业	Manufacture of Rubber and Plastic	7.71
非金属矿物制品业	Manufacture of Non-mettallic Mineral Products	9.09
黑色金属冶炼和压延加工业	Smelting and Pressing of Ferrous Metals	5.82
有色金属冶炼和压延加工业	Smelting and Pressing of Non-ferrous Metals	-0.34
金属制品业	Manufacture of Metal Products	14.89
通用设备制造业	Manufacture of General Purpose Machinery	8.04
专用设备制造业	Manufacture of Special Purpose Machinery	4.62
汽车制造业	Manufacture of Vehicle	7.41
铁路、船舶、航空航天和其他运输设备制造业	Manufacture of Transport Equipment for Railway，Shipping，Aerospace and other uses	10.48
电气机械和器材制造业	Manufacture of Electrical Machinery & Equipment	9.08
计算机、通信和其他电子设备制造业	Manufacture of Computer，Communication Equipment and Other Electronic Equipment	13.65
仪器仪表制造业	Manufacture of Measuring Instrument	27.56
其他制造业	Manufacture of Other Products	5.59
废弃资源综合利用业	Recycling and Disposal of Waste Resources	
金属制品、机械和设备修理业	Manufacture of Metal Products，Machinery and Equipment	54.84
电力、热力生产和供应业	Production and Supply of Electric Power and Heat Power	8.32
燃气生产和供应业	Production and Supply of Gas	6.28
水的生产和供应业	Production and Supply of Water	6.74

资产负债率 (%) Assets-Liability Ratio (%)	流动资产周转次数(次/年) Number of Times of Annual of Turnover Current Assets (time/year)	工业成本费用利润率(%) Ratio of Profits to Industrial Costs (%)	产品销售率(%) Ratio of Sales to Output (%)
**53.35**	**1.35**	**5.41**	**103.51**
66.06	2.06	0.73	97.74
68.31	1.12	–2.43	94.34
36.91	2.21	4.73	184.17
61.60	2.08	0.96	100.08
48.22	1.78	0.79	98.8
59.27	2.99	0.19	99.58
66.23	1.01	4.19	100
57.96	1.35	4.54	99.47
57.21	1.51	3.29	98.35
56.36	1.77	5.54	96.87
45.70	1.55	2.53	98.87
63.60	3.83	2.04	100
54.03	2.15	2.79	101.02
39.10	2.04	16.30	91.01
76.72	1.10	–10.16	99.07
42.00	1.91	5.60	100.7
37.87	1.37	7.49	98.75
41.71	2.12	2.77	98.94
50.87	1.42	–2.50	100
52.72	1.78	11.04	99.78
46.83	1.68	5.87	99.1
53.74	1.00	3.76	101.02
57.40	0.62	9.69	100.06
55.03	1.00	9.47	101.88
50.06	1.75	5.73	96.71
45.24	2.21	8.21	101.64
47.74	1.12	26.64	102.53
59.68	0.62	4.22	99.69
49.51	2.41	19.55	100
63.62	0.81	24.86	100
57.37	2.43	5.54	99.82
64.12	0.77	21.78	100

12-12 按行业分大中型工业企业主要经济效益指标（2018 年）
MAIN INDICATORS ON ECONOMIC BENEFIT OF LARGE AND MEDICM-SIZED INDUSTRIAL ENTERPRISES BY INDUSTRIAL SECTOR（2018）

行业	Indicator	总资产贡献率(%)<br>Ratio of Total Assets to Industrial Output Value<br>(%)
**总计**	**Total**	**8.67**
煤炭开采和洗选业	Mining and Washing of Coal	
石油和天然气开采业	Extraction of Petroleum and Natural Gas	
黑色金属矿采选业	Mining of Ferrous Metal Ores	
有色金属矿采选业	Mining of Non-ferrous Metal Ores	
非金属矿采选业	Mining and Processing of Nometal Ores	
开采辅助活动	Auxiliary Activities of Mining	
其他采矿业	Mining of Other Ores	
农副食品加工业	Processing of Food from Agricultural Products	3.91
食品制造业	Manufacture of Foods	1.31
酒、饮料和精制茶制造业	Manufacture of Liquor，Beverage and Refind Tea	9.03
烟草制品业	Manufacture of Tobacco	
纺织业	Manufacture of Textile	2.05
纺织服装、服饰业	Manufacture of Textile Wearing Apparel	10.85
皮革、毛皮、羽毛及其制品和制鞋业	Manufacture of Leather，Fur，Feather & Its Products Footwear	5.97
木材加工和木、竹、藤、棕、草制品业	Processing of Timbers，Manufacture of Wood，Bamboo，Rattan，Palm and Straw Products	2.98
家具制造业	Manufacture of Furniture	8.97
造纸和纸制品业	Manufacture of Paper and Paper Products	3.17
印刷和记录媒介复制业	Printing，Reproduction of Recording Media	14.29
文教、工美、体育和娱乐用品制造业	Manufacture of Articles for Culture，Arts & Crafts，Sports and Entertainment	4.60
石油、煤炭及其他燃料加工业	Oil, coal and other fuel processing industries	67.91
化学原料和化学制品制造业	Manufacture of Chemical Raw Material and Chemical Products	6.46
医药制造业	Manufacture of Medicines	24.15
化学纤维制造业	Manufacture of Chemical Fibers	
橡胶和塑料制品业	Manufacture of Rubber and Plastic	4.98
非金属矿物制品业	Manufacture of Non-mettallic Mineral Products	7.09
黑色金属冶炼和压延加工业	Smelting and Pressing of Ferrous Metals	5.08
有色金属冶炼和压延加工业	Smelting and Pressing of Non-ferrous Metals	3.17
金属制品业	Manufacture of Metal Products	17.17
通用设备制造业	Manufacture of General Purpose Machinery	3.88
专用设备制造业	Manufacture of Special Purpose Machinery	8.94
汽车制造业	Manufacture of Vehicle	6.52
铁路、船舶、航空航天和其他运输设备制造业	Manufacture of Transport Equipment for Railway，Shipping，Aerospace and other uses	7.58
电气机械和器材制造业	Manufacture of Electrical Machinery & Equipment	6.36
计算机、通信和其他电子设备制造业	Manufacture of Computer，Communication Equipment and Other Electronic Equipment	12.93
仪器仪表制造业	Manufacture of Measuring Instrument	9.50
其他制造业	Manufacture of Other Products	3.74
废弃资源综合利用业	Recycling and Disposal of Waste Resources	
金属制品、机械和设备修理业	Manufacture of Metal Products，Machinery and Equipment	
电力、热力生产和供应业	Production and Supply of Electric Power and Heat Power	-1.75
燃气生产和供应业	Production and Supply of Gas	3.37
水的生产和供应业	Production and Supply of Water	0.02

资产负债率 (%) Assets–Liability Ratio (%)	流动资产周转次数(次/年) Number of Times of Annual of Turnover Current Assets (time/year)	工业成本费用利润率 (%) Ratio of Profits to Industrial Costs (%)	产品销售率 (%) Ratio of Sales to Output (%)
**63.74**	**1.25**	**6.18**	**100.65**
62.09	2.90	0.70	99.22
68.57	0.98	–1.51	93.88
41.02	2.02	4.70	165.46
77.16	1.93	–0.23	98.77
51.37	2.64	3.05	99.55
65.12	2.93	1.04	93.31
80.26	0.79	2.99	100
70.57	1.34	4.69	99.9
61.91	2.97	0.07	96
54.66	1.78	7.42	100.32
39.91	1.89	2.30	101.48
55.37	3.97	8.89	99.62
52.78	1.66	5.35	98.31
28.93	1.36	24.57	95.72
60.27	1.40	4.99	99.09
64.32	0.91	7.82	99.4
74.24	2.17	2.78	100.06
60.20	5.28	5.54	100
49.55	1.75	12.24	101.51
54.28	1.02	3.77	97.37
68.48	1.23	6.15	97.12
67.07	0.87	6.41	99
64.59	0.89	7.24	101.69
70.17	0.91	8.81	96.65
50.30	1.70	5.08	104.48
72.68	0.72	12.48	100
82.71	1.20	1.74	99.62
80.78	1.14	–7.97	97.36
63.68	2.20	2.49	100
58.50	0.59	–7.01	99.53

12-13 主要年份主要工业产品产量
MAJOR YEAR 'S PRODUCTS OUTPUT OF INDUSTRY ABOVE DESIGNATED SIZE

年份 Year	原盐 (万吨) Salt (10 000 tons)	发电量 (亿千瓦小时) Electricity (100 million kW·h)	饮料酒 (万吨) Beverage Liquour (10 000 tons)	#啤酒 Beer	卷烟 (万箱) Cigarettes (10 000 cases)	罐头 (吨) Canned Food (ton)	化学纤维 (吨) Chemical Fiber (ton)	纱 (吨) Yarn (ton)
1949	15.16	1.21	0.18	0.12	2.36			27447
1952	35.08	2.11	0.37	0.18	12.27			59674
1957	44.34	2.92	0.96	0.58	23.41			50699
1962	38.60	3.86	1.58	0.79	16.59		28	17939
1965	41.34	6.80	2.04	1.34	31.34		205	68082
1970	29.96	10.57	3.39	2.55	42.16		621	85591
1975	34.52	10.98	5.30	2.98	36.00		357	79516
1978	60.68	13.63	6.25	3.75	44.00	4743	1413	85233
1980	41.19	12.50	8.49	4.84	48.79	3521	2116	83389
1985	47.34	26.97	13.76	10.05	51.81	6400	1747	91839
1988	53.28	29.35	26.61	15.58	57.01	27200	3393	107720
1990	35.70	40.22	29.18	19.61	61.94		2900	100381
1991	31.39	54.60	36.75	27.38	56.19	130510	5200	96271
1992	46.24	58.63	44.59	37.01	59.37	3200	6000	99043
1993	48.88	59.39	54.74	45.92	63.46	1900	27100	91351
1994	53.02	59.62	60.00	50.92	69.20	2300	21400	86744
1995	50.84	64.69	60.85	54.16	70.96	4609	20973	79492
1996	41.39	83.02	61.79	54.40	73.04	1719	22945	66832
1997	34.11	95.12	66.64	56.06	88.32	554	26300	70402
1998	19.08	79.65	47.33	42.79	77.10		23858	66504
1999	30.30	82.00	126.21	120.10	149.00	379	29967	56431
2000	41.83	90.84	211.47	206.50	148.10	225	65362	69561
2001	31.40	85.73	279.37	274.28	142.95	99	89989	72933
2002	42.50	89.70	323.20	320.60	138.10	11690	91394	70888
2003	36.85	87.69	353.90	351.80	148.00		101752	65919
2004	34.36	89.01	393.30	390.60	147.10	10889	115187	71931
2005	10.00	98.63	439.70	435.20	150.70	10097	95887	72140
2006	15.30	124.54	101.60（本地）	95.60（本地）	95.16（本地）	1668	72381	56128
2007	19.23	155.30	118.10（本地）	112.50（本地）	100.08（本地）	1850	68746	47850
2008	15.90	164.58	132.07（本地）	124.75（本地）	100.02（本地）	1510	51751	43102
2009	5.90	172.66	137.21（本地）	127.12（本地）	100.08（本地）	4706	59852	38566
2010	11.25	181.93	144.66（本地）	141.65（本地）	102.55（本地）	1092	68654	35278
2011		173.69	164.04（本地）	156.50（本地）	104.51（本地）	41204	38640	18556
2012		174.72	174.03（本地）	163.95（本地）	106.03（本地）	35026	32371	19404
2013	1.28	180.11	194.22（本地）	185.17（本地）	108.36（本地）	30203	23647	33687
2014		178.29	169.85（本地）	164.59（本地）	112.06（本地）	23970	22053	32336
2015		173.53	162.66（本地）	157.83（本地）	111.48（本地）	36179	17419	27721
2016		175.40	168.82（本地）	164.27（本地）		53139	14839	33416
2017		181.80	163.41（本地）	159.97（本地）		85942	11565	26493
2018		192.40	165.81（本地）	162.44（本地）		43893	5545	28948

注：1998 年以前为乡及乡以上工业。
Note：Before 1998，the data refer to those at and above county level.

12-13 续表 1
Continued

年份<br>Year	布<br>（万米）<br>Cloth<br>（10 000 m）	印染布<br>（万米）<br>Printed Fabric<br>（10 000 m）	机制纸及纸板（万吨）<br>Machine-made Paper and Paperboards<br>（10 000 tons）	硫酸<br>（万吨）<br>Sulfuric Acid<br>（10 000 tons）	烧碱<br>（万吨）<br>Caustic Soda<br>（10 000 tons）	纯碱<br>（万吨）<br>Soda Ash<br>（10 000 tons）
1949	10608	2947	0.98		0.01	
1952	30069	13658	0.44		0.17	
1957	27806	16053	1.54		0.74	
1962	7444	7375	1.45	0.06	0.89	
1965	29538	13670	2.22		1.66	5.32
1970	33660	21994	3.14	0.04	2.14	12.01
1975	31242	22828	3.10	0.53	2.36	9.15
1978	36877	25797	4.55	1.32	3.61	14.04
1980	34937	22900	5.25	4.11	4.33	18.18
1985	34998	17679	6.20	3.34	5.52	26.03
1988	35890	21213	7.42	4.88	6.62	31.12
1990	34066	21446	6.02	4.86	6.73	30.48
1991	34076	19999	5.99	5.62	5.99	29.50
1992	33682	21534	5.95	6.70	5.62	34.60
1993	35912	16606	8.84	6.96	6.07	40.43
1994	43241	16387	12.36	8.33	6.48	43.36
1995	41170	17864	11.06	9.69	7.90	43.20
1996	39023	16881	11.31	10.12	7.30	46.91
1997	43615	15722	7.78	10.16	8.45	50.55
1998	40440	9864	6.91	10.33	9.25	52.80
1999	39576	4340	7.77	12.56	8.96	53.70
2000	41783	3526	10.96	12.96	10.24	56.20
2001	40640	3402	23.05	13.03	11.44	58.47
2002	47609	5592	29.92	5.00	13.80	60.70
2003	35922	10131	31.76	4.50	13.53	62.50
2004	50111	7718	36.38	14.70	14.86	60.80
2005	51307	16027	31.95	14.80	16.10	62.50
2006	55093	20867	15.50	7.00	15.90	63.95
2007	44251	18708	24.04	9.22	14.91	71.02
2008	48399	58907	22.41	13.02	12.78	72.03
2009	31048	27998	34.59	1.40	12.24	68.21
2010	17457	29631	35.43	4.07	13.87	69.35
2011	49617	28449	32.28	5.06	13.20	70.07
2012	48987	32478	22.27	7.22	13.71	63.35
2013	51646	34381	17.95	4.32	11.06	61.66
2014	44593	33653	18.26			67.85
2015	28040	25458	18.29			68.95
2016	24973	28661	18.17		11.33	
2017	23550	21530	17.39		33.89	
2018	46737	22647	14.58		35.36	

12-13 续表 2
Continued

年份<br>Year	合成氨<br>（万吨）<br>Synthetic Ammonia<br>（10 000 tons）	化学肥料<br>（万吨）<br>Chemical Fertilizer<br>（10 000 tons）	化学农药<br>（吨）<br>Chemical Peticides<br>（ton）	油漆<br>（吨）<br>Paint<br>（ton）	染料<br>（吨）<br>Dye<br>（ton）
1949				30	599
1952				480	2855
1957			1226	1247	4307
1962		0.45	213	910	3560
1965		1.42	2	4727	8667
1970	0.98	5.89	2313	6380	9347
1975	3.46	12.46	4439	7016	5967
1978	7.17	23.66	9714	8786	5877
1980	7.24	7.82	8333	14556	2159
1985	6.31	5.16	2000	26055	4448
1988	8.72	9.41	2400	28943	5527
1990	7.56	7.55	2500	29075	5119
1991	8.52	8.04	2800	33548	5263
1992	10.45	7.85	3400	35058	4821
1993	10.70	8.02	3600	33054	4550
1994	13.04	12.39	3600	29437	5097
1995	14.98	12.62	4934	37772	7185
1996	18.67	12.62	4976	26762	7668
1997	16.94	12.52	4600	19743	11189
1998	22.36	15.30	6755	17594	8274
1999	26.09	20.19	4480	3730	7866
2000	24.64	20.04	3758	693	9306
2001	35.53	28.07	3915	1349	9757
2002	20.30	9.80	4020	1701	9826
2003	21.55	9.70	2039	2106	10734
2004	30.30	19.60	5061	1955	3961
2005	33.80	22.10	6524	1687	13979
2006	33.30	21.30	12544		26544
2007	28.22	25.70	16433	2957	28269
2008	17.93	21.51	12848	12105	18263
2009	17.71	21.23	49684		24893
2010	16.84	14.10	16450		41383
2011	10.78	9.26	23286		
2012	7.71	6.98	28801		
2013	6.48	5.10	27715		
2014			27160		
2015			36853		
2016		14.33	34993		
2017		7.38	18911		
2018		8.08	8507		

12-13 续表 3
Continued

年份 Year	化学原料药（吨） Chemical Medicines（ton）	水泥（万吨） Cement（10 000 tons）	平板玻璃（万重量箱） Plate Glass（10 000 weight cases）	耐火材料（万吨） Refractory Material（10 000 tons）	粗钢（万吨） Crude Steel（10 000 tons）	钢材（万吨） Rolled-steel（10 000 tons）
1949						0.06
1952				0.78	0.36	0.41
1957				3.39	1.77	4.30
1962		2.09		2.41	4.59	2.94
1965		6.59		2.91	11.72	8.43
1970		7.35	16.31	3.23	19.38	14.56
1975	55	20.93	19.44	4.88	21.25	20.01
1978	185	35.40	8.86	5.93	31.06	27.61
1980	123	44.66	70.13	5.38	37.14	32.82
1984	7228	70.27	91.25	7.98	37.81	32.43
1985	4222	82.70	77.00	4.35	37.51	32.68
1988	7655	141.00	108.81	4.56	50.17	38.26
1990	6913	139.48	83.13	3.91	54.55	38.04
1991	4754	165.66	96.90	3.71	56.96	39.02
1992	5743	191.50	120.00	3.86	63.75	50.09
1993	6566	221.30	148.71	3.55	61.75	44.88
1994	4238	246.50	145.12	3.06	60.82	46.95
1995	1993	213.82	426.40	3.88	67.27	49.60
1996	5505	181.45	423.60	3.54	72.02	50.60
1997	1693	208.04	570.85	2.45	75.71	51.06
1998	910	131.51	509.38	0.99	84.02	64.93
1999	1691	149.41	576.50	0.59	108.10	86.58
2000	2131	137.52	556.58	0.62	101.17	93.35
2001	2534	148.67	421.51	0.51	124.45	119.77
2002	2712	140.30	405.40	0.45	145.90	142.20
2003	3288	154.90	361.40	0.47	204.80	204.30
2004	4394	181.19	163.30	0.69	225.70	230.97
2005	4430	210.80	297.50	1.04	309.70	330.30
2006	32232	232.72	418.74	1.21	325.82	375.52
2007	14139	425.53	397.79	2.38	327.00	365.55
2008	17910	363.57	599.87	3.65	300.19	350.14
2009	19086	397.66	681.02	18.46	307.89	349.63
2010	26170	442.66	662.04	8.17	300.04	333.46
2011	29337	588.08	510.05	20.02	318.27	325.89
2012	49290	556.57	494.54	33.44	252.78	274.67
2013	49081	575.66	610.21	42.34	235.53	230.39
2014	50713	597.35	612.40	39.37	214.43	212.79
2015	45923	532.36	534.99	41.13	154.56	140.63
2016	2248	600.56	576.16	108.76	188.04	191.37
2017	2775	525.42	557.44	109.84	280.97	291.85
2018	1809	284.47	561.51	27.97	309.70	319.90

12-13　续表 4
Continued

年份<br>Year	金属切削机床（台）<br>Metal-cutting Machine Tools<br>（unit）	锻压机械（台）<br>Metal Forming Machinery<br>（unit）	汽车（辆）<br>Motor Vehicles<br>（set）	交流电动机（万千瓦）<br>AC Motors<br>（10 000 kW）
1949				0.02
1952	105			0.50
1957	76			3.61
1962	167	77		3.85
1965	526	111	13	6.48
1970	2353	531	120	14.53
1975	3213	788	490	15.56
1978	2558	379	22	21.72
1980	595	444	1644	20.11
1985	900	607	4575	26.08
1988	1336	737	3722	39.60
1990	1049	440	2317	34.28
1991	869	764	1514	44.17
1992	1018	982	3287	61.08
1993	1532	919	4828	64.13
1994	1505	860	2793	37.71
1995	972	2563	5210	39.18
1996	1084	584	12247	22.65
1997	449	416	13020	20.74
1998	280		15475	6.43
1999	251		20038	11.10
2000	384		30053	38.70
2001	472	2477	40658	42.72
2002	588		70079	68.25
2003	990		65612	95.63
2004	1287		62572	96.13
2005	1347		31055	63.50
2006	3636		50278	702.02
2007	3392	1869	61016	119.23
2008	8381	1230	70161	96.89
2009	668		458900	728.03
2010	1120		566211	101.10
2011	1194		488330	74.08
2012	1014		574729	104.48
2013	2689		708966	126.82
2014	2563		770245	114.46
2015	618		861250	104.30
2016	588		816839	98.70
2017	610		741455	202.79
2018	204		92280	230.86

12-13 续表 5
Continued

年份<br>Year	钢芯铝绞线(吨)<br>Steel-cored Aluminum Stranded Wire (ton)	家用电冰箱(万台)<br>Home Refrigerators (10 000 sets)	家用洗衣机(万台)<br>Home Washing Machines (10 000 sets)	彩色电视机(万部)<br>Color TV Sets (10 000 sets)
1949				
1952				
1957				
1962				
1965	250			
1970	34			
1975	188			
1978	684			
1980	1232			
1985	2327	1.66	0.48	6.60
1988	1571	15.22	42.38	17.00
1990	1880	27.41	45.86	18.50
1991	2463	31.51	45.70	22.27
1992	2248	54.84	46.31	28.07
1993	1144	50.48	56.59	36.08
1994	287	62.50	71.34	60.15
1995	1659	107.91	64.34	60.53
1996	2265	193.80	100.94	56.68
1997	2616	257.10	171.95	124.86
1998	1725	219.23	183.67	96.83
1999	2805	259.60	256.30	285.20
2000	4202	311.10	318.50	379.40
2001	4498	396.30	367.89	455.40
2002	5825	518.20	408.6	593.20
2003	4940	598.30	471.5	671.70
2004	6608	815.03	575.06	957.90
2005	116592	908.80	614.9	1324.30
2006	46974	1290.00	649.4	1205.60
2007	22564	1421.40	769.4	1243.10
2008	29507	723.19	407.54	836.66
2009		827.21	488.72	1064.70
2010		801.22	580.87	1111.19
2011		718.54	604.55	1154.20
2012		575.01	583.6	1439.98
2013		524.35	606.65	1512.05
2014		610.07	590.95	1714.93
2015		872.09	595.11	1736.20
2016		882.22	606.74	2111.71
2017		818.65	603.4	1703.01
2018		886.58	601.72	1695.18

12-14　规模以上工业主要产品产量

OUTPUT OF MAJOR INDUSTRIAL PRODUCTS OF INDUSTRY ABOVE DESIGNATED SIZE

产品名称	Name	计算单位	Unit	2018 年	2017 年	2018 年比 2017 年 (± %) 2018/2017 (± %)
**冶金工业产品**	**Products of Metallurgical Industry**					
粗钢	Crude Steel	万吨	10 000 tons	309.70	280.96	10.23
钢材	Rolled-steel	万吨	10 000 tons	319.90	294.28	8.71
耐火材料制品	Refractory Material	万吨	10 000 tons	27.97	27.59	1.39
**电力工业产品**	**Products of Electricity Industry**					
发电量	Electricity	亿千瓦小时	100 million kW·h	192.4	183.6	4.79
**化学工业产品**	**Products of Chemical Industry**					
原油加工量	Processing Amount of Crude Oill	万吨	10 000 tons	1540.75	1522.87	1.17
汽油	Petrol	万吨	10 000 tons	473.91	456.07	3.91
柴油	Diesel Oill	万吨	10 000 tons	382.86	408.08	-6.18
燃料油	Fuel Oil	万吨	10 000 tons	21.79	12.08	80.36
化学农药	Chemical Pesticides	万吨	10 000 tons	0.85	2.11	-59.59
塑料制品	Plastic	万吨	10 000 tons	23.68	23.04	2.80
化学药品(原料)	Chemical Medicines (Paw Materials)	万吨	10 000 tons	0.18	0.15	22.99

12-14　续表 1
Continued

产品名称	Name	计算单位	Unit	2018 年	2017 年	2018 年比 2017 年 (±%) 2018/2017 (±%)
橡胶轮胎外胎	Tires	万条	10 000 tires	6388.78	4676.98	36.60
**机械工业产品**	**Products of Machinery Industry**					
工业锅炉	Industrial Boiler	蒸发量吨	ton（evaporation amunt）	10208.12	13772.67	-25.88
交流电动机	AC Motors	万千瓦	10 000 kW	230.86	202.79	13.84
金属切削机床	Metal-cutting Machine	台	set	204	188	8.51
汽车	Motor Vehicles	辆	set	92280	80669	14.39
改装汽车	Refitted Motor Vehicles	辆	set	20714	20982	-1.28
民用钢质船舶	Civil Steel Ships	载重吨	syn-ton	88923	103207	-13.84
**电子产品**	**Electronic Products**					
彩色电视机	Color TV sets	万台	10 000 sets	1695.18	1703.01	-0.46
电子元件	Electronice	万只	10 000 Units	300433.49	345523.85	-13.05
**建材工业产品**	**Products of Construction Industry**					
水泥	Cement	万吨	10 000 tons	284.47	381.98	-25.53
平板玻璃	Plate Glass	万重量箱	10 000 weight cases	561.51	557.44	0.73

12-14 续表 2
Continued

产品名称	Name	计算单位	Unit	2018 年	2017 年	2018 年比 2017 年 (±%) 2018/2017 (±%)
**纺织工业产品**	**Products of Textile Industry**					
纱	Yarn	吨	ton	28947.6	29403.88	-1.55
布	Cloth	万米	10 000 m	46737.31	49563.20	-5.70
印染布	Printed Fabric	万米	10 000 m	22646.85	19022.00	19.06
化学纤维	Chemical Fibers	吨	ton	5545.28	11617.76	-52.27
**轻工产品**	**Products of Light Industry**					
机制纸及纸板	Machine-made Paper and Paperboards	万吨	10 000 tons	14.58	17.32	-15.85
家用洗衣机	Home Washing Machines	万台	10 000 sets	601.72	603.40	-0.28
糖果	Sugar	吨	ton	6550	11545	-43.27
食用植物油	Vegetable Oil	万吨	10 000 tons	45.94	38.42	19.55
饮料酒	Beverage Liquor	万千升	10 000 KL	163.85	161.01	1.76
# 白酒	Wine	万千升	10 000 KL	2.09	1.97	6.13
# 啤酒	Beer	万千升	10 000 KL	160.52	158.07	1.55
塑料制品	Plastic Products	万吨	10 000 tons	23.68	23.04	2.80
家具	Furniture	万件	10 000 articles	257.66	422.32	-38.99
家用电冰箱	Home Refrigerators	万台	10 000 sets	886.58	857.89	3.34
皮革鞋靴	Leather Shoes	万双	10 000 pairs	2006.43	2086.62	-3.84
服装	Chothing	万件	10 000 articles	48141.93	49412.83	-2.57

12-15　规模以上工业主要产品生产能力
OUTPUT OF MAJOR INDUSTRIAL PRODUCTS OF INDUSTRY ABOVE DESIGNATED SIZE

产品名称	Name	计算单位	Unit	2018 年 生产能力 Production Capacity of 2018	2017 年 生产能力 Production Capacity of 2017
发电设备容量总计 / 发电量	Total Capacity of Generation Equipment	万千瓦 / 万千瓦小时	10 000 kW/ 10 000 kW·h	388	389
化学纤维	Chemical Fiber	吨	ton	15500	15450
棉纺锭 / 纺纱量	Knitting Spindle	锭 / 吨	spindle/ton	205016	205016
棉布织机 / 布	Cotton Cloth Loom	台 / 万米	set/10 000 m	2399	2401
焦炭	Coke	吨	ton	1600000	1600000
农用氮、磷、钾化学肥料总计 (折纯)	Chemical Fertilizer	吨	ton	80000	80000
水泥	Cement	吨	ton	6800000	6800000
平板玻璃	Plate Glass	重量箱	weight cases	6200000	6200000
生铁	Pig Iron	吨	ton	3227000	3227000
粗钢	Crude Steel	吨	ton	3125000	3125000
钢材	Rolled-steel	吨	ton	5146435	5125445
金属切削机床	Metal-cutting Machine	台	set	2995	2928
汽车	Motor Vehicles	辆	set	70000	68000
家用电冰箱	Home Refrigerators	台	set	10749793	11981493
房间空气调节器	Air Conditioner	台	set	14525970	10900000
移动通信手持机 (手机)	Cell Phone	台	set	25859000	33500000
彩色电视机	Color TV Sets	台	set	20700000	20700000

主要统计指标解释

工业总产值　是以货币表现的工业企业在一定时期内生产的已出售或可供出售工业产品总量，它是反映一定时间内工业生产的总规模和总水平。

资产总计　指企业拥有或控制的能以货币计量的经济资源，包括各种财产、债权和其他权利。资产按其流动性（即资产的变现能力和支付能力）划分为：流动资产、长期投资、固定资产、无形资产、递延资产和其他资产。

负债合计　指企业所承担的能以货币计量，将以资产或劳务偿付的债务，偿还形式包括货币、资产或提供劳务。负债一般按偿还期长短分为流动负债和长期负债。

利润总额　指企业生产经营活动的最终成果，是企业在一定时期内实现的盈亏相抵后的利润总额（亏损以“-”号表示），它等于营业利润加上补贴收入加上投资收益加上营业外净收入再加上以前年度损益调整。

Explanatory Notes on Main Statistical Indicators

Gross Industrial Output Value　is the total volume of final industrial products produced and industrial services provided during a given period. It reflects the total achievements and overall scale of industrial production during a given period.

Total Assets　refer to all economic resources，in monetary terms，that is owned or controlled by enterprises，including properties，creditors equity and other economic rights of all forms. Classified by the degree of equitability，total assets include circulating assets，long-term investment，fixed assets，intangible assets and deferred assets，and other assets.

Total Liabilities　refer to payable liabilities of enterprises that have to repay in terms of money，assets or labour services. In terms of payment，it can be divided into liquid liabilities and long-term liabilities.

Total Profits　refer to the final achievements of production and operation of the enterprises，represented by the total profits after deducting losses（loss is expressed by the negative figure）. It is the sum of profits from operation，income from subsidies，investment earnings，net income from activities other than operation，and adjustment of profits and losses of previous years.

建筑业 13

CONSTRUCTION

简 要 说 明

一、本篇资料的主要内容

本篇资料主要反映了全市建筑业基本情况，主要包括建筑业总产值、增加值、建筑企业生产指标、财务指标、重点建筑企业名单等方面的内容。

二、本篇资料的来源

本篇资料来源于建筑业统计年报，由市统计局固定资产投资统计处整理提供。

Brief Introduction

I. Main Content

Data in this chapter show the basic conditions of construction of the whole city，mainly including the gross output value of construction，value added，major production and financial indicators and list of key enterprises of construction，etc.

II. Source of Data

Data in this chapter are based on the annual report of construction industry，and complied by the Division of Investment and Construction Statistics of Qingdao Municipal Bureau of Statistics.

13-1 建筑业企业生产情况（2018 年）
PRODUCTION SITUATION OF CONSTRUCTION ENTERPRISES（2018）

项目	Item	单位	Unit	合计 Total	#中央 of which：Central Enterprises	国有企业 State-owned Enterprise	集体企业 Collectiveowend Enterprise	其他所有制企业 Other Ownership Enterprises
施工企业单位数	Number of Construction Enterpises	个	unit	711	12	19	8	684
建筑业总产值	Gross Output Value of Construction	万元	1000yuan	22617411	4758279	196626	7314	22413471
#建筑工程	of which：Construction	万元	1000yuan	20360384	4113263	155258	7277	20197849
安装工程	Installation	万元	1000yuan	1814993	617342	17641	38	1797315
建筑业增加值	Value Added of Construction	万元	1000yuan	7234000				
全年竣工产值	Output Value of Buildings Completed in the Year	万元	1000yuan	9037628	1100700	130671	26466	8880491
施工房屋面积	Floor Spece of Buildings under Construction	万平方米	1000sp.m	15136	2907	12	20	15104
#新开工	of which：Newly Operating	万平方米	1000sp.m	5547	827	12	8	5527
竣工房屋面积	Floor Space of Buildings Completed	万平方米	1000sp.m	3033	162	10	12	3011
#住宅	of which：Residential Buildings	万平方米	1000sp.m	1917	96	0	12	1905
计算建筑业劳动生产率平均人数	Average Number of Persons for Caleulating the Labor Productivity	人	person	576372	78991	3405	371	572596

13-2 建筑业企业财务状况（2018年）
FINANCIAL SITUATION OF CONSTRUCTION ENTERPRISES（2018）

项目	Item	单位	Unit	合计 Total
流动资产	Circulating Funds	万元	10000yuan	21564526
#存货	of which：Inventory	万元	10000yuan	3412837
年末固定资产原价	Original Value of Fixed Assets（year-end）	万元	10000yuan	2055214
本年固定资产折旧	Depreciation of Fixed Assets in the Year	万元	10000yuan	126477
年末资产合计	Total Assets（year-end）	万元	10000yuan	26069266
年末负债合计	Total Liabilities（year-end）	万元	10000yuan	20142778
所有者权益合计	Total Owners' Equity	万元	10000yuan	5926488
#实收资本	Paid-in Capitals	万元	10000yuan	5341314
主营业务收入	Revenue from Principal Business	万元	10000yuan	22361816
主营业务成本	Cost of Principal Business	万元	10000yuan	20768095
主营业务税金及附加	Taxes and Extra Charges on Principal Business	万元	10000yuan	130363
管理费用	Management Expenses	万元	10000yuan	684809
利润总额	Total Profits	万元	10000yuan	552843
利税总额	Total Pre-tax Profits	万元	10000yuan	1069277
年末应收工程款	Account Receivable（year-end）	万元	10000yuan	7510128

#中央<br>of which：Central Enterprises	国有企业<br>State-owned Enterprise	集体企业<br>Collectiveowend Enterprise	其他所有制企业<br>Other Ownership Enterprises
5848975	317799	5414	21241313
912009	8236	1769	3402832
760716	55042	4318	1995854
59570	1247	280	124950
7078410	374166	12932	25682168
6042307	268299	7592	19866888
1036103	105867	5341	5815280
519880	68404	5773	5267137
6300947	214932	19892	22126993
5897886	191485	17844	20558766
10314	1283	269	128811
204404	12278	1002	671529
134064	8198	510	544134
204095	15097	1391	1052789
1476694	174634	1891	7333604

13-3 建筑业企业主要经济效益指标（2018 年）
MAIN INDICATORS ON ECONOMIC BENEFIT OF CONSTRUCTION ENTERPRISES (2018)

项目	Item	单位	Unit	合计 Total	#中央 of which: Central Enterprises	国有企业 State-owned Enterprise	集体企业 Collectiveowend Enterprise	其他所有制企业 Other Ownership Enterprises
**建筑业劳动生产率**	**Labor Productivity of Construction**							
按总产值计算	In Terms of gross Output Value	元/人	yuan/person	392170	502382	477463	297148	321194
**竣工率**	**Rate of Buildings completd**							
按产值计算	In Terms of Gross output Value	%	%	40.9	43.1	61.5	59.8	39.6
产值工资率	Ratio of Wages to Gross Output Value	%	%	12.8	11.1	15.7	24.9	12.8
资金利润率	Ratio of Profit to Funds	%	%	2.4	1.5	2.1	2.6	2.5
产值利润率	Ratio of Profit to Gross Output Value	%	%	2.8	3.2	4.5	5.3	2.7
流动比率	Current Ratio	%	%	117.9	104.8	113.1	101.2	117.3
资产负债率	Assets-Liability Ratio	%	%	77.2	85.3	71.7	58.7	75.1

13-4 建筑业增加值（2018 年）
VALUE ADDED OF CONSTRUCTION（2018）

单位：亿元（100 million yuan）

市、区名称	Region	增加值 Vaiue Added
**全　市**	**Whole Municipality**	**723.4**
市南区	Shinan District	65.6
市北区	Shibei District	88.1
李沧区	Licang District	16.8
崂山区	Laoshan District	104.8
黄岛区	Huangdao District	186.7
城阳区	Chengyang District	19.1
即墨区	Jimo District	84.4
胶州市	Jiaozhou	79.8
平度市	Pingdu	18.1
莱西市	Laixi	52.4
红岛经济区	Qingdao National High-tech Industrial Development Zone	7.6

注：黄岛区含保税港区数据。
Note：The Huangdao District contains the data of the bonded harbor area.

13-5 重点建筑企业一览表（2018 年）
LIST OF KEY CONSTRUCTION ENTERORISES（2018）

序号 Precedence	企业单位名称	Name	所在地区 Location	资质等级 Qualification Croteria
1	青建集团股份公司	Qingjian Group Co.，Ltd.	市北区 Shibei District	A001
2	中青建安建设集团有限公司	Zhongqing Jianan Construction Group Co.，Ltd.	市南区 Shinan District	A001
3	荣华建设集团有限公司	Ronghua Construction Group Co.，Ltd.	莱西市 Laixi	A001
4	中建八局第四建设有限公司	Fourth Construction of China Construction Eight Bureau Co.，Ltd.	市南区 Shinan District	A001
5	中启胶建集团有限公司	Zhongqi Rubber Construction Group Co.，Ltd.	胶州市 Jiaozhou	A001
6	中铁十七局集团第一工程有限公司	China Railway No.17 Bureau Group First Engineering Co.,Ltd	黄岛区 Huangdao District	A003
7	中铁二十局集团第四工程有限公司	Fourth Engineering of China Railway twenty Bureau Group Co.，Ltd.	崂山区 Laoshan District	A103
8	中铁建工集团山东有限公司	China Railway Construction Engineering Group Shandong Co.，Ltd.	崂山区 Laoshan District	A301
9	东亚装饰股份有限公司	East Asia Decoration Limited by Share Ltd.	市南区 Shinan District	B103
10	青岛一建集团有限公司	Qingdao Yi Jian Group Co.，Ltd.	市北区 Shibei District	A001
11	青岛中建联合建设工程有限公司	Qingdao Zhongjian United Construction Engineering Co.，Ltd.	崂山区 Laoshan District	A101
12	中建筑港集团有限公司	Zhongjian Zhu Gang Group Co.，Ltd.	市北区 Shibei District	A104
13	青岛博海建设集团有限公司	Qingdao Bohai Construction Group Co.，Ltd.	市南区 Shinan District	A001
14	青岛海川建设集团有限公司	Qingdao Hai Chuan Construction Group Co.，Ltd.	市北区 Shibei District	A001
15	山东兴华建设集团有限公司	Shandong Xinghua Construction Group Co.，Ltd.	黄岛区 Huangdao District	A001
16	德才装饰股份有限公司	Decai Decoration Limited by Share Ltd.	崂山区 Laoshan District	B103
17	青岛海尔家居集成股份有限公司	Qingdao Haier Integrated Limited by Share Ltd.	胶州市 Jiaozhou	B103
18	青岛中嘉建设集团有限公司	Qingdao Zhongjia Construction Group Co.，Ltd.	市北区 Shibei District	A101
19	中铁二十五局集团第五工程有限公司	Fifth Engineering Co.，Ltd. of China Railway twenty-five Bureau Group	崂山区 Laoshan District	A110
20	山东莱钢建设有限公司	Shandong Laiwu Iron and Steel Construction Co.，Ltd.	市南区 Shinan District	A101
21	青岛亿联集团股份有限公司	Qingdao Yilian Group Limited by Share Ltd.	黄岛区 Huangdao District	A001
22	青建国际集团有限公司	Qingjian International Group Limited	市北区 Shibei District	A101
23	中铁十局集团青岛工程有限公司	China Railway Ten Bureau Group Qingdao Engineering Co.，Ltd.	市北区 Shibei District	A110
24	中交一航局第二工程有限公司	Second Engineering Company Co.，Ltd. of China Communications Central Airlines	市南区 Shinan District	A104
25	青岛即建建设集团有限公司	Qingdao JiJian Construction Group Co.，Ltd.	即墨区 Jimo District	A101
26	山东电力建设第三工程有限公司	Third Engineering Co.，Ltd. of Shandong Electric Power Construction	崂山区 Laoshan District	A006
27	青岛新华友建工集团股份有限公司	Qingdao Xinhuayou Construction Engineering Group Limited by Share Ltd.	崂山区 Laoshan District	A101
28	青岛温泉建设集团有限公司	Qingdao Hot Spring Construction Group Co.，Ltd.	即墨区 Jimo District	A101
29	山东荣泰建筑工程集团有限公司	Shandong Rongtai Construction Engineering Group Co.，Ltd.	黄岛区 Huangdao District	A001
30	青岛第一市政工程有限公司	Qingdao First Municipal Engineering Co.，Ltd.	市北区 Shibei District	A110
31	青岛恒生源集团建设有限公司	Qingdao Hengshengyuan Group Construction Co.，Ltd.	即墨区 Jimo District	A101
32	青岛城建集团有限公司	Qingdao Urban Construction Group Co.，Ltd.	市北区 Shibei District	A110
33	青岛海德工程集团股份有限公司	Qingdao Hyde Road and Bridge Engineering Limited by Share Ltd.	市南区 Shinan District	A210

13-5 续表 1
Continued

序号 Precedence	企业单位名称	Name	所在地区 Location	资质等级 Qualification Croteria
34	青岛瑞源工程集团有限公司	Qingdao Ruiyuan Engineering Group Co., Ltd.	黄岛区 Huangdao District	A105
35	中国石油天然气第七建设有限公司	China Petroleum and Natural Gas Seventh Construction Co., Ltd.	崂山区 Laoshan District	A109
36	中石化第十建设有限公司	Sinopec tenth Construction Company Limited	黄岛区 Huangdao District	A109
37	青岛土木建工集团有限公司	Qingdao Civil Engineering Group Co., Ltd.	黄岛区 Huangdao District	A101
38	青岛建设集团有限公司	Qingdao Construction Group Co., Ltd.	市南区 Shinan District	A201
39	青岛福瀛建设集团有限公司	Qingdao Fu ying Construction Group Co., Ltd.	黄岛区 Huangdao District	A101
40	青岛颐金建筑装饰工程有限公司	Qingdao Yijin Building Decoration Engineering Co., Ltd.	市南区 Shinan District	B103
41	青岛登科市政工程有限公司	Qingdao Dengke Municipal Engineering Co., Ltd.	城阳区 Chengyang District	A110
42	青岛德泰建设工程有限公司	Qingdao de Tai Construction Engineering Co., Ltd.	黄岛区 Huangdao District	A101
43	山东国建工程集团有限公司	Shandong State Construction Engineering Group Co., Ltd.	崂山区 Laoshan District	A101
44	青岛胶城建设集团有限公司	Qingdao Rubber City Construction Group Co., Ltd.	胶州市 Jiaozhou	A101
45	青岛建工集团有限公司	Qingdao Construction Engineering Group Co., Ltd.	崂山区 Laoshan District	A102
46	青岛市政空间开发集团有限责任公司	Qingdao Municipal Space Development Group Ltd	市南区 Shinan District	A110
47	青岛营上建设集团有限公司	Qingdao Yingshang Construction Group Co., Ltd.	即墨区 Jimo District	A101
48	青岛鑫兴泰建筑工程有限公司	Qingdao xinxingtai Construction Engineering Co., Ltd.	城阳区 Chengyang District	A201
49	青岛市房屋建筑集团股份有限公司	Qingdao Housing Construction Group Limited by Share Ltd.	市北区 Shibei District	A101
50	山东天诚市政公路工程有限公司	Shandong Tiancheng Municipal Highway Engineering Co., Ltd.	崂山区 Laoshan District	A110
51	青岛安装建设股份有限公司	Qingdao Installation and Construction Limited by Share Ltd.	市北区 Shibei District	A112
52	青岛滨海建设集团有限公司	Qingdao Binhai Construction Group Co., Ltd.	黄岛区 Huangdao District	A101
53	青岛青房建安集团有限公司	Qingdao Qingfang Jianan Group Co., Ltd.	市南区 Shinan District	A101
54	青岛建祥建设集团有限公司	Qingdao Jian Xiang Construction Group Co., Ltd.	胶州市 Jiaozhou	A101
55	青岛平建建筑安装股份有限公司	Qingdao Ping Jian Building and Installation Limited by Share Ltd.	平度市 Pingdu	A101
56	青岛艺高城市建设工程有限公司	Qingdao Yi Gao City Construction Engineering Co., Ltd.	市北区 Shibei District	B237
57	青岛港务局港务工程公司	Qingdao Port Bureau Port Engineering Company	黄岛区 Huangdao District	A204
58	青岛公路建设集团有限公司	Qingdao Highway Construction Group Co., Ltd.	崂山区 Laoshan District	A102
59	青岛胶州湾建设集团有限公司	Qingdao Jiaozhou Bay Construction Group Co., Ltd.	胶州市 Jiaozhou	A101
60	青岛昶德建设集团有限公司	Qingdao Changde Construction Group Co., Ltd.	胶州市 Jiaozhou	A101
61	青岛营海建设（集团）有限公司	Qingdao Ying hai Construction Group Co., Ltd.	胶州市 Jiaozhou	A101
62	青岛新城发展建筑工程有限公司	Qingdao New Town Development Engineering Co., Ltd.	市北区 Shibei District	A101
63	青岛东捷建设工程有限公司	Qingdao Dong jie Construction Engineering Co., Ltd.	市北区 Shibei District	A101
64	青岛西海岸市政工程有限公司	Qingdao West Coast municipal Group Municipal Engineering Company	黄岛区 Huangdao District	A110
65	青岛环城建工集团有限公司	Qingdao Ring City Construction Engineering Group Co., Ltd.	黄岛区 Huangdao District	A101
66	青岛地矿岩土工程有限公司	Qingdao Geotechnical Engineering Co., Ltd.	市南区 Shinan District	B101

13-5 续表 2
Continued

序号 Precedence	企业单位名称	Name	所在地区 Location	资质等级 Qualification Croteria
67	青岛东建建设有限公司	Qingdao Dongjian Construction Co.，Ltd.	李沧区 Licang District	A201
68	青岛建设集团建兴工程有限公司	Qingdao construction group LITE-ON Engineering Co.，Ltd.	红岛经济区 Qingdao National High-tech Industrial Development Zone	A101
69	青岛市崂山区古建建筑工程有限公司	Qingdao Laoshan ancient building engineering Co.，Ltd.	崂山区 Laoshan District	A101
70	山东奥华建筑安装工程有限公司	Shandong Ao Hua Construction and Installation Engineering Co.，Ltd.	市南区 Shinan District	A101
71	青岛建设装饰集团有限公司	Qingdao Construction Decoration Group Co.,Ltd.	市南区 Shinan District	B103
72	青岛万怡东方建设集团有限公司	Qingdao Wan Yi Oriental Construction Group Co.，Ltd.	黄岛区 Huangdao District	A101
73	青岛柏高市政园林建设集团有限公司	Qingdao BOGAO Municipal Garden Construction Group Co. Ltd	市南区 Shinan District	A110
74	鲁建集团股份有限公司	Lu Jian Group Limited by Share Ltd.	崂山区 Laoshan District	A202
75	青岛通力建设集团有限公司	Qingdao Tongli Construction Engineering Co.,Ltd.	市南区 Shinan District	A101
76	山东省路通工程集团有限公司	Shandong Lutong Engineering Group Co.，Ltd.	崂山区 Laoshan District	A102
77	青岛金沙滩建设集团有限公司	Oingdao Jinshatan Construction Group Co.，Ltd.	黄岛区 Huangdao District	A101
78	青岛康太源建设集团有限公司	Qingdao Kangtine Construciton Group.Lnc.	李沧区 Licang District	A101
79	青岛九龙建设集团有限公司	Qingdao Jiulong Construction Group Co.，Ltd.	胶州市 Jiaozhou	A101
80	青岛环海工程贸易发展有限公司	Qingdao Huanhai Engineering & Trade Development Co.，Ltd.	市北区 Shibei District	A201
81	青岛三星工程有限公司	Qingdao Sanxing Engineering Co.，Ltd.	城阳区 Chengyang District	A110
82	青岛鑫隆建设集团有限公司	Qingdao Xinlong Construction Group Co.，Ltd.	黄岛区 Huangdao District	A101
83	青岛福海洋建设集团有限公司	Qingdao Fu Ocean Construction Group Co.，Ltd.	胶州市 Jiaozhou	A201
84	青岛华联装饰工程有限公司	Qingdao Hualian Decoration Engineering Co., Ltd.	市北区 Shibei District	B203
85	青岛云柱电气有限公司	Qingdao Yunzhu Electric Co., Ltd.	城阳区 Chengyang District	A206
86	青岛四机建筑安装有限公司	Qingdao Siji Construction Installation Co.,Ltd.	市北区 Shibei District	A201
87	青岛市益水工程股份有限公司	Qingdao Yishui Engineering Co.,Ltd.	市南区 Shinan District	A210
88	青岛望城三宝建设有限公司	Qingdao Wangcheng Sanbao Construction Co., Ltd.	莱西市 Laixi	A101
89	青岛恒华机房设备工程有限公司	Qingdao Heng Hua machine Room Equipment Engineering Co.，Ltd.	市南区 Shinan District	B123
90	青岛元昌盛房建集团有限公司	Qingdao Yuanchangsheng Housing Construction Group Co., Ltd.	胶州市 Jiaozhou	A201
91	青岛方圆达建设集团有限公司	Qindao Fang Yuan Da Construction Group Co.,Ltd.	李沧区 Licang District	A201
92	青岛嘉恒建设集团有限公司	Qingdao Jiaheng Construction Group Co., Ltd.	市南区 Shinan District	A101
93	青岛永鑫建筑工程有限公司	Qingdao Yongxin Construction Engineering Co., Ltd.	平度市 Pingdu	A201
94	青岛市黄岛区园林绿化工程有限公司	Huangdao of Qingdao Landscaping Engineering Co.，Ltd.	黄岛区 Huangdao District	A310
95	青岛青城建工集团有限公司	Qingdao Qingcheng Construction Group Co., Ltd.	红岛经济区 Qingdao National High-tech Industrial Development Zone	A101
96	青岛施运机械施工有限责任公司	Qingdao Construction Transportation Company	市北区 Shibei District	B101
97	青岛恒源送变电工程有限公司	Qingdao Hengyuan transmission and Transformation Engineering Co.，Ltd.	城阳区 Chengyang District	A201
98	青岛太行园林建设有限公司	Qingdao Taihang Garden Construction Co.，Ltd.	黄岛区 Huangdao District	A310
99	青岛政益达实业有限公司	Qingdao Zhengyida Industrial Co., Ltd.	市北区 Shibei District	A210
100	青岛盛泽建设工程有限公司	Qingdao Shengze Construction Engineering Co.， Ltd.	胶州市 Jiaozhou	A201

主要统计指标解释

建筑业统计单位 指从事房屋、构筑物建造和设备安装活动的法人企业。建筑业法人企业应具有建筑业资质并能够独立核算，同时其应具备以下条件：①依法成立，有自己的名称、组织机构和场所，能够承担民事责任；②独立拥有和使用资产，承担负债，有权与其他单位签订合同；③独立核算盈亏，能够编制资产负债表。

建筑业总产值 是以货币形式表现的建筑业企业在一定时期内生产的建筑业产品和提供的服务的总和。建筑业总产值包括：

（1）建筑工程产值：指列入建筑工程预算内的各种工程价值。

（2）安装工程产值：指设备安装工程价值，不包括被安装设备本身的价值。

（3）其他产值：建筑业总产值中除建筑工程、安装工程以外的产值。包括房屋构筑物修理产值、非标准设备制造产值、总包企业向分包企业收取的管理费以及不能明确划分的施工活动所完成的产值。

建筑业增加值 指建筑业企业在报告期内以货币形式表现的建筑业生产经营活动的最终成果。

建筑业现价增加值 按生产法和分配法（收入法）两种方法计算，以收入法的计算结果为准，即从收入的角度出发，根据生产要素在生产过程中应得的收入份额计算。具体计算方法：经济普查年度建筑业增加值按照《经济普查年度 GDP 核算方案》计算，非经济普查年度建筑业增加值按照《非经济普查年度 GDP 核算方案》计算。

Explanatory Notes on Main Statistical Indicators

Statistical Unit in Construction refers to corporate enterprise engaged in the construction of buildings and structures and in the installation of equipment. A corporate construction enterprise should have qualification certificates with independent accounting system, and should meet the following 3 requirements：a）being set up in line with relevant legal basis，having its full name，organization and location，and capable of taking civil liabilities；b）independently possessing and using its assets and assuming its liabilities，and entitled to sign contracts with other institutions；and c）making independent accounts of its profits and losses，and capable of compiling its own balance sheet.

Gross Output Value of Construction refers to total of construction products and services，expressed in money terms，produced or rendered by construction and installation enterprises during a given period of time. It includes：

（1）Output value of construction projects，that is the value of projects covered by the project budgets；

（2）Output value of installation projects，that is the value of the installation of equipment，（excluding the value of the equipment to be installed）；

（3）Output value of others，that is the output value of construction industry excluding that of construction projects and installation projects. It includes：output value of repair of buildings and structures；output value of non-standard equipment manufacturing；overhead expenses received by contracted enterprises to the sub-contracted enterprises and the completed output value of construction activities that have no clear definition.

运输、邮电 14

TRANSPORT，POSTAL AND TELECOMMUNICATION SERVICES

简 要 说 明

一、本篇资料的主要内容

本篇资料主要反映了全市交通运输业和邮电业通讯业发展的基本情况，主要包括客货运输及港口吞吐量、民用汽车拥有量、独立核算运输邮电单位主要财务指标、邮电通讯基本情况、邮政行业发展情况、快递企业发展情况、电信行业基本情况、城市轨道交通发展情况等方面的内容。

二、本篇资料的来源

1、本篇资料中交通运输邮电资料来源于全市交通运输邮电业统计年报。

2、交通运输资料来源于青岛市交通运输委员会、青岛市港航局、青岛市运管局、青岛港（集团）有限公司、青岛国际机场集团有限公司、青岛地铁集团有限公司。

3、邮政业资料来源于青岛市邮政管理局、中国邮政集团公司青岛市分公司。

4、电信业资料来源于青岛市通信管理局、中国电信集团公司山东省青岛市电信分公司、中国电信股份有限公司青岛分公司、中国联合网络通信有限公司青岛市分公司、中国移动通信集团山东有限公司青岛分公司、中国铁通集团有限公司青岛分公司。

5、民用汽车拥有量资料来源于青岛市公安局交警支队车辆管理所。

本篇资料由市统计局服务业统计处整理提供。

Brief Introduction

I. Main Content

Data in this chapter show the basic conditions of the development of transport，post and telecommunications in Shandong Province，mainly including the freight traffic and passenger traffic，cargo handled at port，possession of civil motor vehicles，major financial indices and basic conditions of transport，postal and telecommunication services，development of the postal services, express company development，development of urban rail transit.

II. Source of Data

（1）Data on transport are based on the annual report of transport.

（2）Data on transportation are provided by Qingdao Municipal Transportation Committee, Qingdao Port and Shipping Bureau , Qingdao Transportation Management Bureau, Qing Dao Port International Co.,Ltd, Qing Dao International Airport Group Co.,Ltd, Qingdao Metro Group Co.,Ltd.

（3）Data on postal and telecommunication are based on Qingdao Municipal Postal Administration，Qingdao Post Bureau.

（4）Data on business of telecommunication services Qingdao Communication Administration, China Telecom Group Corporation Qingdao Branch of Shandong Province, China Telecom Co., Ltd. Qingdao Branch, China United Network Communications Co., Ltd. Qingdao Branch, China Mobile Communications Group Shandong Co., Ltd. Qingdao Branch, China Tietong Group Co., Ltd. Qingdao Branch.

（5）Date on possession of civil motor vehicles are are provided by Qingdao Municipal Public Security Bureau Traffic Police Detachment Vehicle Management Office.

Data in this chapter are prepared and provide by the Division of Industry Statistics of Qingdao Municipal Bureau of Statistics.

14-1 主要年份客货运输及港口吞吐量
MAJOR YEAR'S PASSENGER & FREIGHT TRAFFIC AND HANDLING CAPACITY OF THE PORTS

年份 Year	客运量（万人） Passenger Traffic（10000 persons）				货运量（万吨） Freight Traffic（10000 tons）				港口吞吐量（万吨） Cargo Handied at Ports（10000 tons）	集装箱吞吐量（万标箱） Containers（10000 TEU）
	铁路 Railways	公路 Highways	海运 Waterways	航空 Civil Aviation	铁路 Railways	公路 Highways	海运 Waterways	航空 Civil Aviation		
1949	327	14	9		386	162	22		73	
1952	571	49	13		447	399	44		181	
1957	1054	149	13		747	548	118		269	
1962	3100	141	39		909	524	97		391	
1965	1424	230	19		1257	690	158		524	
1970	1329	373	20		1856	736	232		695	
1975	1803	159	30		2325	817	282		1542	
1978	1986	274	49		2917	1561	528		2081	
1980	1896	713	73		2596	1642	941		1779	
1985	2063	1894	86	1.0	3009	2303	919	0.03	2610	
1988	2083	3300	234	8.8	3418	6486	1160	0.04	3153	
1990	1488	3639	316	14.1	3741	6433	1556	0.20	3068	
1991	1453	4144	366	19.0	3774	6896	1789	0.31	3194	
1992	1488	4965	437	27.0	3899	6822	1981	0.45	3240	
1993	1499	6436	499	35.3	4044	7685	1910	0.64	3650	
1994	1564	6787		47.0	4112	8336	1804		4331	
1995	1480	9785		66.6	4205	11788	2185		5165	
1996	1230	10738		79.9	4415	10749	474		6056	81.1
1997	1308	11855			4466	11232	544		6944	103.3
1998	1549	11754			4028	14311	1792	1.44	7044	121.3
1999	1674	12103			3919	14550	1926	1.96	7282	154.3
2000	1780	12145		124.1	4256	17234	2998	2.30	8661	212.0
2001	1678	13686		143.0	4778	20906	3432	2.40	10423	264.0
2002	1530	14516	777	163.7	5079	24480	2530	3.00	12252	341.0
2003	1366	13132	786	174.7	4929	27241	2773	3.60	14135	424.0
2004	1496	16420	862	241.9	5119	29937	3503	5.30	16303	514.0
2005	552	17212	972	280.9	2299	31009	4471	4.60	18727	630.7
2006	810	17752	999	320.2	2790	32047	4768	5.10	22438	770.0
2007	1101	18800	1080	733.8	2781	33336	4019	5.90	26507	946.6
2008	1278	19909	1055	755.8	3225	34172	5080	7.20	30029	1037.7
2009	1425	19612	1145	881.8	3772	16297	4332	7.19	31668	1027.6
2010	1537	20460	1302	1010.9	5031	17525	4407	8.44	35012	1201.0
2011	1693	21560	791	1074.8	5697	19208	4220	8.93	37971	1302.0
2012	1881	22724	414	1173.1	6126	21312	1791	9.06	41465	1450.0
2013	2056	23588	333	1572.4	6266	23270	1313	9.31	45782	1552.0
2014	2301	5588	324	1641.2	5564	19043	1434	10.22	47701	1658.0
2015	2421	5370	288	1820.2	5486	20073	1456	20.80	49749	1743.0
2016	2701	4532	225	2050.5	5569	20701	1685	23.07	51463	1805.0
2017	3179	4534	215	2321.1	6021	24716	1800	23.20	51314	1830.9
2018	3455	4602	161	2453.6	6280	27453	2502	22.45	54250	1931.5

注：从 2014 年公路客、货运量统计口径进行调整，与以前年度不可比。
Note：In 2014，the Ministry of Transportation made an adjustment to the statistical gauge for highway passenger and freight transportation and the data are not comparable directly.

14-2 民用车辆拥有量（2018 年底）
ROSSESSION OF CIVIL MOTOR VEHICLES（END OF 2018）

单位：辆（unit）

项目	Item	合计<br>Total<br>辆数<br>Number	#个人<br>of which：Private Vehicles<br>辆数<br>Number
**一、汽车**	**Automobile**	**2690558**	**2321113**
1. 载客汽车	Passenger Vehicles	2426198	2176901
其中：轿车	of which：Sedan	1576286	1432953
2. 载货汽车	Trucks	248075	134205
3. 其他汽车	Other Automobile	16285	10007
**二、电车**	**Tram**	**125**	**0**
**三、摩托车**	**Motorcycle**	**106356**	**104836**
**四、农用运输车**	**Agricultural Camion**		
**五、挂车**	**Trailer Trucks**	**28610**	**273**
**六、其他类型车**	**Other Vehicles**	**142**	**2**

补充资料：机动车驾驶员（3350694 人）Motor drivers（person）
其中：汽车驾驶员（3304193 人）of which：Automobile drivers（person）

14-3 客货运输及港口吞吐量（2018 年）
PASSENGER & FREIGHT TRAFFIC AND HANDLING CAPACITY OF THE PORTS（2018）

项目	Item	货运量（万吨）Freight Traffic（10 000 tons）	货物周转量（亿吨公里）Freight Ton-kilometers（100million tons · km）	客运量（万人）Passenger Traffic（10 000 persons）	旅客周转量（亿人公里）Passenger-kilometers（100million persons-km）	旅客吞吐量（万人）Volume of Passenger Handled（10000 persons）	货物吞吐量（万吨）Volume of Freight Handled（10000 tons）	集装箱（万标箱）Containers（10000 TEU）
**总计**	**Total**	**36235**	**1647.12**	**8218**	**182.49**	**2473**	**54272**	**1932**
铁路	Railways	6280	190.90	3455	105.36			
公路	HiIghways	27453	532.20	161	0.16			
水运	Waterways	2502	924.02	4602	76.97			
机场	Civil Aviation					2454	22	
港口	Ports					19	54250	1932

14-4 规模以上运输邮电单位主要财务指标（2018 年）
MAJOR FINANCIAL INDICATORS OF TRANSPORTATION，POST AND TELECOMMUNICATION UNITS ABOVE DESIGNATED SCALE（2018）

单位：万元（10 000yuan）

项目	Item	营业收入 Revenue from principal Business	营业成本 Cost of principal Business	营业利润 Operating Profits	营业税金及附加 Business taxes and surtax	利润总额 Total Profits
**总计**	**Total**	**8739006**	**7740691**	**539461**	**34513**	**727935**
道路运输业	Road Transport	1739578	1833292	-70903	10541	82664
水上运输业	Water Transport	305332	227560	29216	5670	47375
航空运输业	Air Transport	385408	321742	39981	2182	42876
管道运输业	Pipeline Transport	24783	9291	13159	125	13590
多式联运和运输代理业	Multimodal Transport and Transport Agent	3824941	3574956	140759	5527	141518
装卸搬运和仓储业	loading and unloading and storage	900786	762783	45264	4628	55308
邮政业	Post	325276	283198	5870	1480	5497
电信、广播电视和卫星传输服务	Telecommunticatìons,Radia and Television and Satellite Transmission Services	1232904	727869	336115	4359	339107

14-5 邮电通讯基本情况（2018 年）

BASIC CONDITIONS OF POSTAL AND TELECOMMUNICATION SERVICES（2018）

市、区名称	Region	邮电局（处）Post Office（unit）	#提供邮政全功能服务的 of which：Providing Omni-directional Services	邮路总长度（公里）Length of Postal Routes（km）	邮运汽车（辆）Vehicles for Postal Services（unit）	信筒、信箱（个）Post Boxes（unit）
**全市**	**Whole Municipality**	**265**	**216**	**5974**	**391**	**663**
市南区	Shinan District	19	10	1505	47	79
市北区	Shibei District	22	13	1743	54	92
李沧区	Licang District	9	4	71	20	31
崂山区	Laoshan District	7	4	56	15	25
黄岛区	Huangdao District	48	38	670	69	86
城阳区	Chengyang District	12	9	178	28	29
即墨区	Jimo District	36	34	522	48	75
胶州市	Jiaozhou	31	29	295	40	63
平度市	Pingdu	54	50	636	38	127
莱西市	Laixi	27	25	298	32	56

注：黄岛区含保税港区数据；城阳区含红岛经济区数据。
Note：The Huangdao District contains the data of the bonded harbor area，Chengyang District includes Hongdao Economic Zone.

14-6 邮政行业发展情况
DEVELOPMENT OF THE POSTAL SERVICES

指标名称	Name	单位	Unit	2018 年	2017 年
**一、邮政行业业务收入**	**Postal Services Business Income**	**亿元**	**100 million yuan**	**62.81**	**53.43**
1、邮政寄递服务	Postal Delivery Service	亿元	100 million yuan	3.05	2.88
2、快递业务	Express Service	亿元	100 million yuan	50.22	41.33
**二、邮政行业业务总量**	**Total Business Volume Of Postal Industry**	**亿元**	100 million yuan	**82.87**	**72.46**
1、邮政寄递服务	Postal Delivery Service	万件	10 000 articles	17449	18243
其中：函件	Number Of Letters	万件	10 000 articles	1612	1317
包裹	Package	万件	10 000 articles	29	28
订销报纸累计数	Cumulative number of newspapers ordered and sold	万份	10 000 copies	14566	15206
订销杂志累计数	Cumulative number of magazines ordered and sold	万份	10 000 copies	629	677
汇兑	Exchange	万笔	10 000 sums	11	17
2、快递业务	Express Service	万件	10 000 articles	38323	31150
其中：同城	Same City	万件	10 000 articles	10738	7700
异地	Different Places	万件	10 000 articles	27016	22854
国际 / 港澳台	International/Hong Kong, Macao And Taiwan	万件	10 000 articles	569	596

14-7 快递企业发展情况
EXPRESS COMPANY DEVELOPMENT

指标名称	Name	单位	Unit	2018 年	2017 年
**快递业务量**	**Express Business Volume**	**万件**	**10 000 articles**	**38323**	**31150**
同城	Same City	万件	10 000 articles	10738	7700
异地	Different Places	万件	10 000 articles	27016	22854
国际及港澳台	International/ Hong Kong, Macao And Taiwan	万件	10 000 articles	569	596
**快递业务收入**	**Express Revenue**	**亿元**	**100 million yuan**	**50.22**	**41.33**
同城	Same City	亿元	100 million yuan	8.99	6.51
异地	Different Places	亿元	100 million yuan	27.02	22.07
国际及港澳台	International/ Hong Kong, Macao And Taiwan	亿元	100 million yuan	7.88	7.77
其他	Others	亿元	100 million yuan	6.33	4.97

14-8 电信行业基本情况
BASIC SITUATION OF TELECOM INDUSTRY

指标名称	Name	单位	Unit	2018 年	2017 年
**一、电信业务总量（2015 年不变价）**	**TOTAL TELECOMMUNICATIONS BUSINESS**	**亿元**	**100 million yuan**	**568.01**	**260.72**
其中：固定话音业务总量	Total Fixed Voice Service	亿元	100 million yuan	1.84	2.41
固定数据及互联网业务总量	Fixed Data And Total Internet Business	亿元	100 million yuan	16.26	14.36
移动话音业务总量	Total Mobile Voice Service	亿元	100 million yuan	22.40	23.25
移动数据及互联网业务总量	Total Mobile Data And Internet Services	亿元	100 million yuan	431.26	148.75
电信业务收入	Telecommunications Revenue	亿元	100 million yuan	113.26	108.99
**二、电信营业网点**	**Telecom Business Network**	**个**	**unit**	**482**	**495**
**三、固定电话交换机总容量（含农话）**	**Total Capacity Of Fixed Telephone Exchanges(Including Rural Fixed Telephone)**	**万门**	**10 000 line**	**339**	**299**
固定电话年末用户	Fixed Telephone Year-End User	万户	10 000 households	139	148
其中：新装电话	Newly Installed Telephone	万户	10 000 households	7	12
城市电话用户	Urban Telephone User	万户	10 000 households	105	111
乡村电话用户	Village Phone User	万户	10 000 households	30	33
住宅电话年末用户	Home Phone Year-End User	万户	10 000 households	54	58
城市电话用户	Urban Telephone User	万户	10 000 households	38	41
乡村电话用户	Village Phone User	万户	10 000 households	16	17
**四、固定电话本地通话量**	**Fixed Telephone Local Call Volume**	**万分钟**	**10 000 minutes**	**109990**	**144430**
长话电路	Long Distance Circuit	路	line	15665	15062
长途电话通话量	Long Distance Call Volume	万次	10 000 times	618219	590271
长途电话通话时长	Long-Distance Phone Call Duration	万分钟	10 000 minutes	16456	22348
**五、互联网用户数**	**Number Of Internet Users**	**万户**	**10 000 households**	**1462**	**1311**
固定宽带互联网用户数	Number Of Fixed Broadband Internet Users	万户	10 000 households	398	369
移动互联网用户数	Number Of Mobile Internet Users	万户	10 000 households	1064	941

14-8 续表
Continued

指标名称	Name	单位	Unit	2018 年	2017 年
**六、互联网通信量**	**Internet Traffic**				
固定互联网宽带使用时长	Fixed Internet Broadband Usage Time	万分钟	10 000 minutes	48342353	51871902
固定互联网宽带接入流量	Fixed Internet Broadband Access Traffic	万 G	10 000 G	517	867
移动互联网接入流量	Mobile Internet Access Traffic	万 G	10 000 G	25952	9731
**七、光缆线路长度**	**Cable Line Length**				
长途光缆线路长度	Long-Distance Cable Line Length	公里	km	8068	7193
本地网中继光缆线路长度	Line Length of Relay Optical Cable in Local Network	公里	km	91564	75822
接入网光缆线路长度	Optical Cable Line Length In Access Network	公里	km	215744	173974
**八、移动通信用户**	**Mobile Communication Users**				
移动电话交换机容量	Mobile Phone Switch Capacity	万户	10 000 households	2930	2862
期末移动电话用户数	nmber of mobile phone users at the end of the period	万户	10 000 households	1259	1223
其中：3G 移动电话用户	3G Mobile Phone Users	万户	10 000 households	132	120
其中：4G 移动电话用户	4G Mobile Phone Users	万户	10 000 households	932	821
**九、移动电话通话量**	**Mobile Phone Call Volume**				
移动电话通话时长	Mobile Phone Call Duration	万分钟	10 000 minutes	1402646	1441012
**十、通信能力**	**Communication Ability**				
移动电话交换机容量	Mobile Phone Switch Capacity	万户	10 000 households	2930	2862
移动电话基站数	Number Of Mobile Phone Base Stations	个	unit	53448	45041
其中：3G 移动电话基站数	Number Of 3G Mobile Phone Base Stations	个	unit	8295	9644
其中：4G 移动电话基站数	Number Of 4G Mobile Phone Base Stations	个	unit	30377	24005
**十一、互联网通信能力**	**Internet Communication Capability**				
互联网宽带接入端口	Internet Broadband Access Port	个	unit	7263867	5610728

14-9 城市轨道交通发展情况
DEVELOPMENT OF URBAN RAIL TRANSIT

指标名称	Name	单位	Unit	2018 年	2017 年
**客运量**	**Passenger Traffic**	**万人次**	**10 000 person-times**	**15294**	**6573**
**日均客运量**	**Average Daily Passenger**	**万人次**	10 000 person-times	**42**	**18**
**旅客周转量**	**Passenger Turnover**	**万人公里**	**10 000 passenger-km**	**124411**	**58983**
**运营车数**	**Number Of Vehicles Operated**	**辆**	**unit**	**522**	**294**
其中：地铁	Subway	辆	unit	522	294
轻轨	Subway	辆	unit		
**运营线路总数**	**Total Number Of Operating Lines**	**条**	**line**	**4**	**2**
其中：新增运营线路	New Operating Line	条	line	2	1
其中：地铁	Subway	条	line	2	1
轻轨	Light Rail	条	line		
**运营线路总长度**	**Total Length Of Operating Line**	**公里**	**km**	**169**	**45**
其中：新增运营线路长度	New Operating Line Length	公里	km	121	20
其中：地铁	Subway	公里	km	121	20
轻轨	Light Rail	公里	km		
**地铁站点总数**	**Total Number Of Subway Stations**	**个**	**unit**	**80**	**38**
其中：换乘站点个数	Number Of Transfer Stations	个	unit	3	2
其中：新增站点个数	Number Of New Sites	个	unit	42	16
其中：新增换乘站点个数	Increase The Number Of Transfer Stations	个	unit	1	2
**总开行次数**	**Total Number Of Trips**	**列次**	**train number**	**329849**	**119101**
**总运营里程**	**Total Operating Mileage**	**公里**	**km**	**9149612**	**2919273**
**列车准点率**	**Train On Time Rate**	**%**	**%**	**99.97**	**99.96**

主要统计指标解释

货（客）运量 指在一定时期内，各运输部门实际运送的货物（旅客）数量。是反映运输业为国民经济和人民生活服务的数量指标，也是制定和检查运输生产计划，研究运输发展规模和速度的重要指标。货运按吨计算，客运按人计算。货物不论运输距离长短，货物类别，均按实际重量统计；旅客不论行程远近或票价多少，均按一人一次作为客运量统计。半价票、小孩票也按一人统计。

货物（旅客）周转量 指在一定时期内，由各种运输工具运送的货物（旅客）数量与其相应运输距离的乘积之总和，是反映运输业生产总成果的重要指标，也是编制和检查运输生产计划，计算运输效益、劳动生产率以及核算运输单位成本的主要基础资料。通常以吨公里和人公里为计算单位。计算货物周转量通常按发出站与到达站之间的最短距离，也就是计费距离计算。

港口货物吞吐量 指经水运进出沿海主要港区范围，并经过装卸的货物数量，包括邮件及办理托运手续的行李、包裹以及补给运输船舶的燃、物料和淡水。货物吞吐量按货物流向分为进口、出口吞吐量，按货物交流性质分为外贸货物吞吐量和国内贸易货物吞吐量。货物吞吐量的货类构成及其流向，是衡量港口生产能力大小的重要指标。

邮政行业业务总量 指以货币形式表现的邮政企业为社会提供各类邮政通信服务或其他服务的总数量。计算方法为各类邮政通信服务业务的实物量分别乘以相应的不变单价，求出各类业务的货币量后加总求得。该指标反映了一定时期邮政通信业务发展的总成果，是观察邮政通信业务发展变化总趋势的综合性指标。

快递业务量 指企业收寄的各类快递业务总数量，由受理用户委托的企业负责统计。包括国内同城快递业务量、国内异地快递业务量、港澳台快递业务量、国际快递业务量。

电信业务总量 指以货币形式表示的电信企业为社会提供的各类电信服务的总数量。包括固定话音业务总量、固定数据及互联网业务总量、固定增值及其他业务总量、移动话音业务总量、移动数据及互联网业务总量、移动增值及其他业务总量。

长途光缆线路长度 指长途光缆线路的实际长度。架空的光缆按实际杆路长度统计，埋设地下、水底的光缆按沟长统计。

本地网中继光缆线路长度 指本地网内各业务节点之间，从起点至终点所经由的光缆线路实际长度。架空光缆按实际杆路长度统计，埋设地下、水底的光缆按埋设光缆的沟长统计。

移动电话基站数 指为小区服务的无线收发信设备，处理基站与移动台之间的无线通信，在移动交换机与移动台之间起中继作用，监视无线传输质量的全套设备数。

3G 移动电话基站数 指报告期末本电信企业 3G 移动通信网络上实际使用的 NoFe-B 数量。包括 TF-SCFMA 基站、WCFMA 基站和 CFMA2000 基站。

4G 移动电话基站数 指报告期末本电信企业 4G 移动通信网络上实际使用的 Node-B 数量。包括 TD-LTE 基站和 FDD-LTE 基站。

互联网宽带接入端口 指用于接入互联网用户的各类实际安装运行的宽带接入端口的数量。

Explanatory Notes on Main Statistical Indicators

Freight（Passenger）Traffic refers to the volume of freight（passenger）transported with various means. Freight transport is calculated in tons and passenger traffic is calculated in the number of persons. Despite the type of freight and traveling distance，the freight transport is calculated in the actual weight of the goods：and despite the traveling distance and ticket price，the passenger traffic is calculated by the principle that one person can be counted only once in one travel. The passengers who travel with a half price ticket or a child ticket is also calculated as one person. The freight（passenger）traffic provides a quantitative measure to show how the transport industry serves the national economy and people，and is also an important indicator for planning the transport industry and for studying the development scale and speed of the transport industry.

Freight Ton-kilometers（Passenger-kilometers） refer to the sum of the products of the volume of transported cargo（passengers）multiplying by the transport distance. It is an important indicator to reflect the achievement of transportation industry. Normally，the shortest distance between the departure station and the destination station （i.e.，the payable distance）is the basis to calculate the freight ton-kilometers. This is an important indicator to show the total results of the transport industry，to prepare and examine the transport plan and to measure the efficiency，the labour productivity and the unit cost of transport.

Volume of Freight Handled in Ports refers to the volume of cargo passing in and out the harbor area of the major coastal ports and having been loaded and unloaded. The volume includes that of the postal matters，registered luggage and fuels，materials and fresh water as supplies of the ships. The volume of freight handled may be classified by direction of flow as freight for import and freight for export，or by nature of cargo as freight for domestic trade and freight for foreign trade. As an important indicator，the volume of freight handled by type of cargo and by main flow direction reflects the production capacity of ports.

Business Volume of Post Services refers to the total amount of post services or other services provided by the post departments for society. The calculation method is that the physical quantity of each type of postal communication service business is multiplied by the corresponding constant unit price, and the monetary quantity of each type of service is obtained and summed up. This indicator reflects the total results of the development of postal communication services in a certain period of time, and is a comprehensive indicator for observing the general trend of development and change of postal communication services.

Business Volume of Express Services refers to the total number of various types of express delivery services received and received by the enterprise, and the enterprises entrusted by the user are responsible for statistics. Including domestic city express business volume, domestic off-site express business volume, Hong Kong, Macao and Taiwan express business volume, international express business volume.

Business Volume of Telecommunication Services refers to the total number of telecommunication services provided by telecommunication enterprises in the form of money for the society. Including the total volume of fixed voice services, fixed data and total Internet services, fixed value-added and other services, total mobile voice services, total mobile data and Internet services, mobile value-added and other business.

Long-Distance Cable Line Length refers to the actual length of the long-distance cable line. The overhead optical cable is counted according to the actual length of the pole. The underground and underwater cables are counted according to the length of the trench.

Local Network Trunk Cable Length Refers to the actual length of the cable line between the service nodes in the local network and from the starting point to the destination. The overhead optical cable is counted according to the actual length of the pole. The underground and underwater optical cables are counted according to the length of the buried optical cable.

Number of Mobile Phone Base Stations refers to the wireless transceiver device serving the cell, handles the wireless communication between the base station and the mobile station, and acts as a relay between the mobile switch and the mobile station to monitor the total number of devices for wireless transmission quality.

Number of 3G Mobile Phone Base Stations refers to the number of NoFe–B actually used on the 3G mobile communication network of the telecommunications enterprise at the end of the reporting period. It includes TF–SCFMA base station, WCFMA base station, and CFMA2000 base station.

Number of 4G Mobile Phone Base Stations refers to the number of Node–B actually used on the 4G mobile communication network of the telecommunications enterprise at the end of the reporting period. It includes TD–LTE base station and FDD–LTE base station.

Internet Broadband Access Port refers to the number of broadband access ports that are actually installed and operated for accessing Internet users.

批发和零售业 15

WHOLESALE AND RETAIL TRADES

简要说明

一、本篇资料的主要内容

本篇资料主要反映了全市批发和零售业经营情况和效益情况，主要包括批发和零售业商品流转及财务情况、亿元以上商品交易市场、社会消费品零售总额等内容。

二、本篇资料的来源

本篇资料来源于批发和零售业统计年报和定期报表统计资料。由市统计局外经贸易统计处整理提供。

Brief Introduction

I. Main Content

Data in this chapter show the development of wholesale and retail trade of the whole city, mainly including the circulation and financial indices of commodities in the wholesale and retail trade, commodity exchange markets over 100 million yuan, Total retail sales of consumer goods, etc.

II. Source of Data

Data in this chapter are based on annual report and regular reports of wholesale and retail trade.

Data in this chapter are prepared and compiled by the Division of Trade and External Economic Relations Statistics of Qingdao Municipal Bureau of Statistics.

15-1 社会消费品零售总额（1985-2018 年）
TOTAL RETAIL SALES OF CONSUMER GOODS（1985-2018）

单位：万元（10 000 yuan）

年份 Year	消费品零售总额 Total Retail Sales of Consumer Goods	批发零售业 Wholesale and Retail Trades	住宿餐饮业 Hotels and Catering Services	其他行业 Othres
1985	307222	231477	16996	18753
1986	364091	269418	21384	27118
1987	432569	310658	23319	32617
1988	590538	434572	32304	45449
1989	619941	450812	34083	56303
1990	658264	484770	35568	67863
1991	760562	566767	43320	56812
1992	888411	666911	53045	67392
1993	1384533	1031794	80077	96759
1994	1880654	1352073	127313	202937
1995	2376776	1637349	186028	305947
1996	2708527	1873008	220322	321325
1997	3008981	2105728	241928	361248
1998	3368736	2322209	277696	411210
1999	3762754	2715635	315307	391388
2000	4282871	3116990	387944	403854
2001	4911735	3561731	507012	433851
2002	5574364	4162676	638358	402654
2003	6455113	5283584	849032	322497
2004	7475022	6266750	1017193	191079
2005	8701057	7304660	1176539	219858
2006	10163457	8458226	1443120	262111
2007	12162246	9995772	1837754	328720
2008	14922153	12995842	1554086	372225
2009	17302231	14232954	2549203	520074
2010	19611331	17151504	2459827	
2011	23023703	20153079	2870624	
2012	26356180	23043005	3313175	
2013	29868133	26142405	3725728	
2014	33617217	29435856	4181361	
2015	37136940	32459539	4677401	
2016	41049345	35840499	5208846	
2017	45410086	39669946	5740140	
2018	48424565	42209564	6215001	

注：1. 自 2003 年开始消费品零售总额不再包括制造业零售额和农民对非农居民的销售额分组。
2. 自 2005 年起，住宿业从其它行业调整到住宿餐饮业中。
3. 根据第二次经济普查结果，对 2005 年以来的历史数据进行了调整。

Note：1.Since 2003，retail sales of manufacturing and retail sales of rural residents to urban residents are excluded in total retail sales of consumer goods.
2.Since 2005，hotels is adjusted to hotels and catering services from the others.
3.Historical data since 2005 have been adjusted according to results of the Second China Economic Census.

15-2 分市、区社会消费品零售总额（2018 年）
TOTAL RETAIL SALES OF CONSUMER GOODS BY REGION（2018）

单位：万元（10 000 yuan）

市、区名称	Region	消费品零售总额 Total Retail Sales of Consumer Gddos	按行业分	
			批发零售业 Wholesale and Retail Trades	住宿餐饮业 Hotels and Catering Services
**全　市**	**Whole Municipality**	**48424565**	**42209564**	**6215001**
市南区	Shinan District	6237329	5088017	1149312
市北区	Shibei District	7748227	6830252	917976
李沧区	Licang District	4629130	4172420	456710
崂山区	Laoshan District	2720213	2535450	184763
黄岛区	Huangdao District	6247883	5268752	979131
城阳区	Chengyang District	2758130	2410785	347346
即墨区	Jimo District	4981556	4504040	477516
胶州市	Jiaozhou	4877139	4232948	644191
平度市	Pingdu	4415180	3888430	526750
莱西市	Laixi	3429782	2932326	497456
红岛经济区	Qingdao National Hightech Industrial Development Zone	194745	166096	28648
保税港区	Qingdao Free Trade Port Area of China	185250	180048	5202

15-3 限额以上批发和零售业商品购销存总额（2018 年）
TOTAL RETAIL SALES OF CONSUMER GOODS BY REGION（2018）

单位：万元（10 000 yuan）

项目	Item	商品销售总额 Total Sales Value	#批发总额 Wholesale Value	零售总额 Retail Value	库存总额 Stock
**总　　计**	**Total**	**76342679.1**	**61087847.5**	**15254831.6**	**3801103.4**
国有企业	State-owned Enterprises	96408.5	73158.0	23250.5	30739.9
集体企业	Collective-owned Enterprises	82616.1	77151.5	5464.6	654.2
其它企业	Other Enterprises	76163654.5	60937538.0	15226116.5	3769709.3
农、林、牧产品批发	Wholesale of Farm Produce and Livestock Products	1084294.8	1079890.4	4404.4	93370.3
食品、饮料及烟草制品批发	Wholesale of Food，Beverages and Tobaccos	4835490.1	4670466.9	165023.2	348306.0
纺织、服装及家庭用品批发	Retail of Textiles，Garments and Daily Consumer Articles	12795566.5	12449014.2	346552.3	279673.4
文化、体育用品及器材批发	Wholesale of Culture，Sports Appliances and Equipments	866029.1	836483.4	29545.7	73050.4
医药及医疗器材批发	Wholesale of Medicines and Medical Appliances	2036980.6	2032380.7	4599.9	176027.0
矿产品、建材及化工产品批发	Wholesale of Machinery，Hardware and Electronic Equipment	33066883.7	32910530.0	156353.7	931994.6
机械设备、五金产品及电子产品批发	Wholesale of Machinery，Hardware and Electronic Equipment	5090647.4	5008510.0	82137.4	317143.7
贸易经纪与代理	Trade Broker and Agency	200838.9	200838.9	0.0	5942.3
其它批发业	Other Wholesale not Classified Elsewhere	675459.6	675442.5	17.1	12198.9
综合零售	Integrated Retail Trade	3337941.6	308.5	3337633.1	232903.3
食品、饮料及烟草制品专门零售	Retail of Food，Beverages and Tobaccos	223381.5	21041.6	202339.9	25829.4
纺织、服装及日用品专门零售	Retail of Textiles，Garments and Daily Consumer Articles	1255013.2	253795.6	1001217.6	301935.9
文化、体育用品及器材专门零售	Retail of Culture，Sports Appliances and Equipments	281654.6	68185.4	213469.2	72567.6
医药及医疗器材专门零售	Retail of Medicines and Medical Appliances	770555.7	73987.1	696568.6	85511.9
汽车、摩托车、燃料及零配件专门零售	Retail of Motor Vehicles，Motorcycles，Fuel and Parts	5603640.3	367721.1	5235919.2	503740.5
家用电器及电子产品专门零售	Retail of Household Electric Appliances and Electronic Products	793738.9	72940.1	720798.8	73984.2
五金、家具及室内装饰材料专门零售	Retail of Hardware，Furniture and Decoration Materials	230022.5	129964.5	100058.0	13781.3
货摊、无店铺及其他零售业	Non-shop and Other Retails	3194540.1	236346.6	2958193.5	253142.7

15-4 限额以上批发和零售业商品分类销售额（2018 年）

SALES VALUE OF ENTERPRISES ABOVE DESIGNATED SIZE OF WHOLESALE AND RETAIL TRADES BY CATEGORY OF COMMODITIES（2018）

单位：万元（10 000 yuan）

项目	Item	销售总额 Total Sales Value	#批发总额 Wholesale Value	零售总额 Retail Value
**销售总额**	**Total Sales Value**	**71119193.9**	**55846423.9**	**15272770**
1. 粮油、食品类	Grain & Oil and Food	3783984	2192273.7	1591710.3
其中：粮油类	Grain and Oil	669575.8	495100.1	174475.7
肉禽蛋类	Meat，Poultry and Eggs	589878.3	379430.4	210447.9
水产品类	Aquatic Products	210369.6	145401.9	64967.7
蔬菜类	Vegetable and Fruit	206814.8	133619	73195.8
干鲜果品类	Dried and Fresh Fruit	627590.3	449122.3	178468
2. 饮料类	Beverages	258957.3	111817.1	147140.2
3. 烟酒类	Tobacco and Liquor	1866552.8	1552974.6	313578.2
4. 服装、鞋帽、针纺织品类	Clothing，Shoes，Hats and Textiles	3922632.7	2239862	1682770.7
（1）服装类	Clothing	2031715.5	994685.1	1037030.4
（2）鞋帽类	Shoes and Hats	745889.4	181242.7	564646.7
（3）针纺织品类	Knitwear and Textiles	1145027.8	1063934.2	81093.6
5. 化妆品类	Cosmetics	317800.6	92467.5	225333.1
6. 金银珠宝类	Gold，Silver and Jewellery	533685.9	145144.8	388541.1
7. 日用品类	Articles for Daily Use	971334.7	314559.4	656775.3
其中：儿童玩具类	Children toys	28345.5	1514.8	26830.7
8. 五金、电料类	Hardware and Electrical Materials	351275.2	331523.8	19751.4
9. 体育、娱乐用品类	Sports and Recreation Articles	117429.9	18496.1	98933.8
其中：照相器材类	Photographic Apparatus	7961.6	0.3	7961.3
10. 书报杂志类	Newspapers and Magazines	167084	62802.9	104281.1
11. 电子出版物及音像制品类	E-journals and Video Products	6487.1	2049.7	4437.4
12. 家用电器和音像器材类	Household Appliances and Video Appilances	12481134.8	9648613.2	2832521.6
13. 中西药品类	Traditional Chinese and Western Medicines	2280243.1	1618977.2	661265.9
其中：西药类	Western Medicines	1858749.8	1379207.4	479542.4
中草药及中成药类	Traditional Chinese Medicines	204662.2	85904.2	118758
14. 文化办公用品类	Cultural and Offices Appliances	970275.5	561249.3	409026.2
其中：计算机及其配套产品	Computer and Related Products	430480.3	364463.3	66017
15. 家具类	Furniture	106811.7	10189.7	96622
16. 通讯器材类	Communication Appliances	738999.8	416209	322790.8
17. 煤炭及制品类	Coal and Related Products	3529013.2	3521048.8	7964.4
18. 木材及制品类	Wood and Wooden Products	617248.3	617248.3	0
19. 石油及制品类	Petroleum and Related Products	11343606.7	9557981.9	1785624.8
20. 化工材料及制品类	Chemical Materials and Related Products	5059435.6	5059435.6	0
其中：化肥类	Fertilizers	338366.1	338366.1	0
21. 金属材料类	Metal Materials	11886594.9	11886594.9	0
22. 建筑及装潢材料类	Building and Decoration Materials	513245.7	446892.3	66353.4
23. 机电产品及设备类	Mechanical and Electrical Products	1735137	1688427.8	46709.2
其中：农机类	Agricultural Machineries	17291	17291	0
24. 汽车类	Automobiles	4744664.2	1068713.9	3675950.3
25. 种子饲料类	Seeds and Feedstuff	625634.2	625634.2	0
26. 棉麻类	Cotton，Hemp	441130.2	440930.3	199.9
27. 其他类	Others	1748794.8	1614305.9	134488.9

注：此表为定期报表数据。
Note：The data are from regular reports.

15-5 限额以上批发业财务状况（2018 年）
FINANCIAL POSITION OF ENTERPRISES ABOVE DESIGNATED SIZE OF WHOLESALE TRADE（2018）

单位：万元（10 000 yuan）

指标	Indicator	合计 Total	国有经济 State-owned Enterprises	集体经济 Collective-owned Enterprises	外商及港澳台经济 Foreign Funded Enterprises and Enterprises with Funds from Hong Kong，Macao and Taiwan	其它 Other Enterprises
**单位数**	**Number of Emterprises**	**1338**	**7**	**2**	**55**	**1274**
**年末资产负债**	**Year-end Assets-Liability**					
资产总计	Total Assets	25029523	91205	44872	1742491	23150955
流动资产合计	Sub-total of Current Assets	22873778	68608	43511	1334069	21427590
负债合计	Total Liabilities	20993961	63876	39760	1238454	19651870
所有者权益合计	Total Owners’ Equities	4035562	27329	5112	504037	3499085
**损益及分配**	**Loss，Profits and Distributon**					
主营业务收入	Revenue from Principal Business	53664627	71530	66485	2427113	51099499
主营业务成本	Cost of Principal Business	51179104	70082	60342	2276379	48772302
主营业务税金及附加	Taxes and Extra Charges on Principal Business	191702	40	98	3877	187688
营业利润	Profits from Principal Business	517305	-1297	-1583	68095	452090
其他业务利润	Profits from other Business	81444	1149	125	2456	77714
销售费用	Operating Costs	1096177	1287	892	40184	1053814
管理费用	Management Expenses	564044	3092	6887	29129	524937
财务费用	Financial Expenses	227726	964	-4	18560	208206
利润总额	Total Profits	554044	792	-1584	71905	482931
**经济效益**	**Economic Benefit**					
商品经营费用率 %	Ratio of Operating Costs to Revenue from Principal Business	2.04	1.80	1.34	1.66	2.06
商品销售利润率 %	Ratio of Profit to Sales Revenue	1.03	1.11	-2.38	2.96	0.95

15-6 限额以上零售业财务状况（2018 年）

FINANCIAL POSITION OF ENTERPRISES ABOVE DESIGNATED SIZE OF RETAIL TRADE（2018）

单位：万元（10 000 yuan）

指标	Indicator	合计 Total	国有经济 State-owned Enterprises	集体经济 Collective-owned Enterprises	外商及港澳台经济 Foreign Funded Enterprises and Enterprises with Funds from Hong Kong，Macao and Taiwan	其它 Other Enterprises
**单位数**	**Number of Emterprises**	**725**	**5**	**2**	**40**	**678**
**年末资产负债**	**Year-end Assets-Liability**					
资产总计	Total Assets	7222060	26359	2668	908207	6284825
流动资产合计	Sub-total of Current Assets	5012891	24153	2587	716855	4269296
负债合计	Total Liabilities	5667847	18514	1809	642365	5005160
所有者权益合计	Total Owners’ Equities	1941127	7846	860	265842	1666579
**损益及分配**	**Loss，Profits and Distributon**					
主营业务收入	Revenue from Principal Business	13548768	19798	4846	1870188	11653936
主营业务成本	Cost of Principal Business	11980012	15587	4185	1535994	10424246
主营业务税金及附加	Taxes and Extra Charges on Principal Business	41045	74	14	10658	30298
营业利润	Profits from Principal Business	181965	2049	303	68763	110850
其他业务利润	Profits from other Business	152667	0	231	39228	113208
销售费用	Operating Costs	992239	167	82	192204	799786
管理费用	Management Expenses	421599	1960	259	103755	315626
财务费用	Financial Expenses	70396	98	3	2320	67974
利润总额	Total Profits	182252	2145	302	69444	110361
**经济效益**	**Economic Benefit**					
商品经营费用率 %	Ratio of Operating Costs to Revenue from Principal Business	7.32	0.84	1.69	10.28	6.86
商品销售利润率 %	Ratio of Profit to Sales Revenue	1.35	10.83	6.23	3.71	0.95

15-7 分市、区城乡亿元商品交易市场分布情况（2018年）

BASIC STATISTICS ON COMMODITY EXCHANGE MARKETS OF TRANSACTION VALUE OVER 100 NILLION YUAN BY REGION（2018）

市、区名称	Region	亿元商品交易市场数量（个）Number of Commodity Exchange Markets of Transaction Value over 100 Million Yuan（unit）			亿元商品交易市场成交额（亿元）Turnover of Commodity Exchange Markets of Transaction Value over 100 Million yuan（100 million yuan）		
		合计 Total	#综合市场 Integrated Markets	#专业市场 Special Markets	合计 Total	#综合市场 Integrated Markets	#专业市场 Special Markets
**全　市**	**Whole Municipality**	**55**	**11**	**44**	**1250.8**	**464.8**	**786.0**
市南区	Shinan District						
市北区	Shibei District	7	1	6	79.4	47.0	32.4
李沧区	Licang District	6	1	5	46.9	14.8	32.1
崂山区	Laoshan District	2		2	2.9		2.9
黄岛区	Huangdao District	7	2	5	69.2	22.0	47.2
城阳区	Chengyang District	2	1	1	219.4	218.3	1.1
即墨区	Jimo District	10	1	9	611.2	137.2	474.0
胶州市	Jiaozhou	11	3	8	119.1	8.3	110.8
平度市	Pingdu	9	2	7	90.6	17.3	73.3
莱西市	Laixi						
红岛经济区	Qingdao National Hightech Industrial Development Zone						
保税港区	Qingdao Free Trade Port Area of China	1		1	12.1		12.1

主要统计指标解释

社会消费品零售总额 指各种经济类型的批发和零售业、住宿和餐饮业以及其他行业对城乡居民和社会集团的消费品零售额总和。该指标从 2003 年开始不再包括制造业零售额和农民对非农居民的零售额。

商品购进总额 指从本企业（单位）以外的单位和个人购进（包括从国外直接进口）作为转卖或加工后转卖的商品。这个指标反映批发零售贸易业从国内、国外市场上购进商品的总量。商品购进总额包括：（1）从工农业生产者购进的商品；（2）从出版社、报社的出版发行部门购进的图书、杂志和报纸；（3）从各种类型的批发零售贸易企业（单位）购进的商品；（4）从其他单位购进的商品，如从机关、团体、企业、单位购进的剩余物资，从餐饮业、服务业购进的商品，从海关、市场管理部门购进的缉私和没收的商品，向居民收购的废旧商品等；（5）从国（境）外直接进口的商品，但不包括企业（单位）为自身经营用和未通过买卖行为而收入的商品以及销售退回、商品损益等。

商品销售总额 指对本企业（单位）以外的单位和个人出售（包括对国（境）外直接出口）的商品。这个指标反映批发零售贸易业在国内市场上销售商品以及出口商品的总量。商品销售总额包括：（1）售给城乡居民和社会集团消费用的商品；（2）售给工业、农业、建筑业、运输邮电业、批发零售贸易业、餐饮业、服务业等作为生产、经营使用的商品；（3）售给批发零售贸易业作为转卖或加工后转卖的商品；（4）对国（境）外直接出口的商品。不包括：出售本企业（单位）自用的废旧包装用品，未通过买卖行为付出的商品，经本单位介绍，由买卖双方直接结算，本单位只收取手续费的业务，购货退出的商品以及商品损耗和损失等。

Explanatory Notes on Main Statistical Indicators

Total Retail Sales of Consumer Goods refers to the sum of retail sales of consumer goods sold by all sectors of the national economy to urban and rural residents and social groups.Sectors of the national economy include wholesale and retail trade，accommodation and catering trade and others.Since 2003，retail sales of manufacture and retail sales of rural residents to urban residents are excluded in total retail sales.

Total Purchases of Commodities refer to the total value of purchases of commodities by the enterprises（establishments）from other establishments or individuals（including direct import from abroad）for the purpose of re-selling，either with or without further processing of the commodities purchased. This indicator is used to show the total value of purchases of commodities by wholesale and retail establishments from domestic and overseas markets. The total purchases include：（1）agricultural and industrial products purchased from producers；（2）books，magazines and newspapers purchased from distribution departments of the publishers；（3）commodities purchased from wholesale and retail establishments of different status of registration；（4）commodities purchased from other units，such as surplus materials purchased from government agencies，enterprises or institutions，commodities purchased from catering and service establishments，confiscated goods purchased from customs authorities or market management agencies，second-hand goods and wastes purchased from residents；and（5）commodities directly imported from abroad. Excluded are commodities purchased by enterprises（establishments）for use in their own business operation，commodities obtained without buying or selling procedures，rejected commodities，etc.

Total Sales of Commodities refer to value of commodities sold by the establishments to other establishments and individuals（including direct export）. This indicator is used to show the total value of sales of commodities at domestic markets and export. The total sales include：（1）commodities sold to urban and rural residents and social groups for their consumption；（2）commodities sold to establishments in industry，agriculture，construction，transportation，post and telecommunications，wholesale and retail trades，hotels and catering services，and public utility for their production and operation；（3）commodities sold to wholesale and retail establishments for re-selling，with or without further processing；and（4）commodities for direct export to other countries. Excluded are selling of waste packaging materials used by the establishments（units）themselves，commodities transferred without buying or selling procedures，commission income from brokerage in transactions for which settlement is directly handled by buyers and sellers，rejected commodities in the purchase，loss in commodities，etc.

住宿、餐饮业和旅游 16

HOTELS，CATERING SERVICES AND TOURISM

简 要 说 明

一、本篇资料的主要内容

本篇资料主要反映了全市住宿和餐饮业的经营情况和效益情况以及旅游的基本情况，主要包括住宿和餐饮业经营情况和财务情况、涉外以及国内旅游基本情况等方面的内容。

二、本篇资料的来源

本篇资料中住宿和餐饮业经营情况和财务情况资料来源于住宿和餐饮业统计年报和定期报表统计资料；旅游资料来源于市旅游局。由市统计局外经贸易统计处整理提供。

Brief Introduction

I. Main Content

Data in this chapter show the development of wholesale and retail trade and tourism of the whole city, mainly including the circulation and financial indices of hotels and catering services, international and domestic tourism, etc.

II. Source of Data

Data on hotels and catering services are based on annual report and regular reports of hotels and catering services. Data on international tourism are provided by Qingdao Tourism Bureau. The above data are prepared and compiled by the Division of Trade and External Economic Relations Statistics of Qingdao Municipal Bureau of Statistics.

16-1 限额以上住宿和餐饮业法人企业经营情况（2018年）

BUSINESS CONDITIONS OF ENTERPRISES OF HOTELS AND CATERING SERVICES ABOVE DESIGNATED SIZE（2018）

单位：万元（10 000 yuan）

项目	Item	营业额 Turnover	#客房收入 Income from Guest Rooms	餐费收入 Catering Income	商品销售收入 Income from Commodity Sales	其他收入 Other Income
**总　计**	**Total**	**1134264.3**	**288103.4**	**747476.5**	**22255.5**	**76428.9**
**一、住宿业**	**Hotels**	**522013.4**	**248898.4**	**194782.3**	**12528.9**	**65803.8**
国有企业	State-owned Enterprises	76932.7	31876.7	34262.8	913.0	9880.2
集体企业	Collective-owned Enterprises					
其他企业	Other Enterprises	445080.7	217021.7	160519.5	11615.9	55923.6
独立门店	Freestanding Stores	453418.4	217839.5	181900.8	12356.4	41321.7
连锁总店	General Chain Stores					
连锁直营店	Chain Store	2081.2	1974.4	46.3	22.5	38.0
连锁加盟店	Chain Store	3974.1	3679.5	27.4	45.7	221.5
其他	Others	62539.7	25405.0	12807.8	104.3	24222.6
五星	Five Star Class	132615.8	60733.7	58312.5	1583.4	11986.2
四星	Four Star class	99476.3	43454.9	47584.0	911.5	7525.9
三星	Three Star class	46530.2	22493.1	19791.3	1096.8	3149.0
二星	Two Star class	1089.8	304.6	753.9	31.3	0.0
其他	Others	242301.3	121912.1	68340.6	8905.9	43142.7
**二、餐饮业**	**Catering Services**	**612250.9**	**39205.0**	**552694.2**	**9726.6**	**10625.1**
国有企业	State-owned Enterprises	10546.4	5821.8	4101.9	400.6	222.1
集体企业	Collective-owned Enterprises	3297.3	533.1	795.8	1931.1	37.3
其他企业	Other Enterprises	598407.2	32850.1	547796.5	7394.9	10365.7
独立门店	Freestanding Stores	241633.5	34189.8	191126.3	5735.2	10582.2
连锁总店	General Chain Stores	268364.2		266313.3	2050.9	
连锁直营店	Chain Store	7714.5		7684.6	29.9	
连锁加盟店	Chain Store	41755.2		41755.2		
其他	Others	52783.5	5015.2	45814.8	1910.6	42.9

16-2 限额以上住宿业财务状况（2018 年）
FINANCIAL SITUATION OF HOTELS ABOVE DESIGNATED SIZE（2018）

单位：万元（10 000 yuan）

指标	Indicator	合计 Total	国有经济 State-owned Enterprises	集体经济 Collective-owned Enterprises	外商及港澳台经济 Foreign Funded Enterprises and Enterprises with Funds from Hong Kong，Macao and Taiwan	其它 Other Enterprises
**单位数**	**Number of Emterprises**	**181**	**15**		**11**	**155**
**年末资产负债**	**Year-end Assets-Liability**					
资产总计	Total Assets	1270184	102633		322699	844852
流动资产合计	Sub-total of Current Assets	481917	45066		68056	368795
负债合计	Total Liabilities	956162	41343		268130	646689
所有者权益合计	Total Owners' Equities	314021	61289		54569	198163
**损益及分配**	**Loss，Profits and Distributon**					
主营业务收入	Revenue from Principal Business	483401	71926		92986	318488
主营业务成本	Cost of Principal Business	171899	18147		35359	118392
主营业务税金及附加	Taxes and Extra Charges on Principal Business	9199	948		3135	5117
营业利润	Profits from Principal Business	-6896	56		607	-7559
其他业务利润	Profits from other Business	4352	2386		86	1880
销售费用	Operating Costs	144674	34287		18074	92313
管理费用	Management Expenses	151291	20328		36401	94563
财务费用	Financial Expenses	20651	95		4451	16105
利润总额	Total Profits	-5139	1064		116	-6318
**其他**	**Others**					
应付职工薪酬	Total Wages Payble in This Year	140147	30803		27342	82001
主营业务利润率	Profitability of Principal	-1.06	1.48		0.12	-1.98

16-3 限额以上餐饮业财务状况（2018 年）
FINANCIAL SITUATION OF CATERING SERVICES ABOVE DESIGNATED SIZE（2018）

单位：万元（10 000 yuan）

指标	Indicator	合计 Total	国有经济 State-owned Enterprises	集体经济 Collective-owned Enterprises	外商及港澳台经济 Foreign Funded Enterprises and Enterprises with Funds from Hong Kong，Macao and Taiwan	其它 Other Enterprises
**单位数**	**Number of Emterprises**	**187**	**6**	**2**	**18**	**161**
**年末资产负债**	**Year-end Assets-Liability**					
资产总计	Total Assets	602422	15541	1030	145903	439948
流动资产合计	Sub-total of Current Assets	358413	10675	933	49122	297683
负债合计	Total Liabilities	498347	6325	993	109521	381508
所有者权益合计	Total Owners' Equities	104075	9216	37	36383	58440
**损益及分配**	**Loss，Profits and Distributon**					
主营业务收入	Revenue from Principal Business	572329	8995	3013	249664	310657
主营业务成本	Cost of Principal Business	288859	5594	2708	124323	156235
主营业务税金及附加	Taxes and Extra Charges on Principal Business	1619	97	7	284	1232
营业利润	Profits from Principal Business	29945	-115	83	24930	5048
其他业务利润	Profits from other Business	965	5		166	795
销售费用	Operating Costs	170827	1514	200	73708	95406
管理费用	Management Expenses	78972	2737	17	26770	49447
财务费用	Financial Expenses	5049	-27	-1	1478	3599
利润总额	Total Profits	29684	-111	83	24845	4868
**其他**	**Others**					
应付职工薪酬	Total Wages Payble in This Year	128547	2824.0	362	46232	79128
主营业务利润率	Profitability of Principal	5.19	-1.23	2.74	9.95	1.57

16-4 入境旅游人数（2000-2018年）
NUMBER OF OVERSEA VISITOR ARRIVALS（2000-2018）

项目	Item	2000年	2005年	2008年	2009年
**总计**	**Total**	**260592**	**684407**	**800455**	**1000670**
**一、外国人**	**Foreigner**	**177098**	**596177**	**697391**	**801424**
# 韩国	Korea	69878	295111	340648	363181
日本	Japan	67215	177121	210090	261553
美国	United States	10384	23284	28998	28745
俄罗斯	Russia	4820	12062	3774	9525
德国	Germany	3820	10646	11261	14735
新加坡	Singapore	6321	6060	10683	17453
英国	United Kingdom	2798	5744	10657	9103
加拿大	Canada	1865	4397	5467	7741
法国	France	2491	5166	6067	7030
意大利	Italy	1555	3963	3871	3747
澳大利亚	Australia	1817	4257	7940	6641
菲律宾	Philippines	7947	3459	2075	7800
泰国	Thailand	780	3261	2236	3685
印尼	Indonesia	1241	2349	1589	4221
新西兰	New Zealand	442	710	1161	2029
**二、港澳和台湾同胞**	**Chinese Compatriots from Hong Kong，Macao and Taiwan**	**60367**	**88230**	**103064**	**199246**

16-5 入境旅游收入（2000-2018年）
EARNINGS FROM INTERNATIONAL TOURISM（2000-2018）

项目	Item	2000年	2005年	2008年	2009年
**总计**	**Total**	**118164**	**340899**	**347645**	**377000**
旅游购物	Shopping	20742	67047	66053	70763
住宿费	Accommodation	17443	37277	37198	41093
餐饮费	Dining	11223	33557	33374	36569
交通费	Transportation	35252	128112	9039	9877
邮电费	Postal and Telecommunication	5502	18394	17382	18812
文化娱乐费	Culture and Entertainment	6894	20240	20163	22394
游览	Sightseeing			18773	21037
其他	Other Services		36272	145663	29745

单位：人次（person-time）

2010 年	2011 年	2012 年	2013 年	2014 年	2015 年	2016 年	2017 年	2018 年
**1080511**	**1156391**	**1270113**	**1282814**	**1280526**	**1338098**	**1410494**	**1443715**	**1535733**
**826628**	**809043**	**877593**	**905415**	**951732**	**996191**	**1042213**	**1056800**	**1118860**
382225	298096	338383	338191	366584	389832	68010	432430	451046
242082	216755	185613	146080	135179	140762	151832	164095	174147
35539	45842	48431	54178	57907	62771	68010	73905	79392
15793	15750	26432	26519	25356	28529	30534	32540	37053
21626	26178	26311	19014	21723	23425	25456	26636	28646
13444	16626	16316	16305	19975	21310	23713	25257	26060
17639	23955	28385	19496	22817	23812	26133	28006	28942
9279	12007	11678	9727	9483	10659	11592	12544	15473
11766	12019	15595	21422	17828	19388	21836	23507	25035
3867	7028	5622	5679	7705	8747	9234	10627	11559
8223	15465	16644	11149	15339	16798	18839	20304	21491
14140	14259	7546	18630	6668	7300	7891	8489	9837
2572	3271	2369	3692	4803	5331	5784	6628	7367
7863	8128	4571	10011	6568	5692	6220	7388	9816
1597	6265	6448	1452	2549	2872	3352	4230	7367
**253883**	**347348**	**392520**	**377399**	**328794**	**342511**	**368281**	**386915**	**416873**

单位：万元（10 000 yuan）

2010 年	2011 年	2012 年	2013 年	2014 年	2015 年	2016 年	2017 年	2018 年
**399688**	**441171**	**519495**	**511158**	**503900**	**566742**	**656966**	**694103**	**798374**
74104	80823	93872	94055	109246	122473	141970	129381	102192
43933	59823	59378	58577	62232	69879	81004	77184	150893
38747	40191	51378	51065	44041	49589	57483	68091	122950
145551	134425	170238	180945	160492	180791	209573	249113	273842
19081	16720	23014	22950	19400	21593	25031	33248	16766
24032	36132	32832	32103	33409	36271	42045	42201	62273
22978	51705	32832	32152	44192	49930	57879	40674	33532
31262	21353	55951	39311	30888	36216	41982	54211	35927

16-6 国内旅游人数及收入（2018 年）
NUMBER OF DOME STTC TOURISM INCOME（2018）

指标	Indicator	单位	Unit	2018 年
国内旅游人数	Number of Domestic Toursts	万人次	10000 person-times	9848.94
1. 过夜旅游者人数	Number of Overnight Tourists	万人次	10000 person-times	4855.06
（1）旅游住宿设施国内旅游人数	Domestic Tourists Staying Overnight at Hotels	万人次	10000 person-times	2135.26
（2）住亲友家去景点的国内旅游人数	Domestic Tourists Staying Overnight at Relatives and Friends’ s Home	万人次	10000 person-times	2719.80
2. 不过夜旅游者（一日游人数）	Number of Same-day（One-day Sightseeing）Tourists	万人次	10000 person-times	4993.88
旅游景点接待一日游人数	One-day Sightseeing Tourists Received by Tour Scenes	万人次	10000 person-times	4993.88
（1）本地一日游人数	Number of Local One-day Sightseeing Tourists	万人次	10000 person-times	2353.47
（2）外地一日游人数	Number of One-day Sightseeing Tourists from Outside Areas	万人次	10000 person-times	2640.41
国内旅游人均花费	Per Capita Expenditure of Domestic Tourist	元	yuan	1676.31
国内旅游收入	Earings from Domestic Tourism	亿元	100 million yuan	1650.99
1. 接待过夜旅游者收入	Earings from Overnight Tourists	万 元	10000 yuan	14493800
2. 接待不过夜旅游者（一日游）收入	Earings from Same-day（One-day Sightseeing）Tourists	万 元	10000 yuan	2016100

主要统计指标解释

住宿餐饮业营业额 指住宿和餐饮业法人企业、产业活动单位在经营活动中因提供服务或销售商品等取得的收入，包括客房收入、餐费收入、商品销售收入和其他收入。客房收入指住宿和餐饮业法人企业、产业活动单位在经营活动中因提供住宿服务取得的客房收入。餐费收入指住宿和餐饮业法人企业、产业活动单位因为顾客提供就餐服务取得的收入，包括经烹饪、调制加工后出售的各种食品，如主食、炒菜、凉拌菜等的收入。商品销售收入指住宿和餐饮业法人企业、产业活动单位伴随服务而出售商品所取得的收入。其他收入指营业收入中除客房收入、餐费收入、商品销售收入以外的其他收入，包括娱乐、健身和商务服务等。

国内旅游者 是指不以谋求职业、获取报酬为目的，离开惯常居住环境，到国内其它地方从事参观、游览、度假等旅游活动（包括外出探亲、疗养、考察、参加会议和从事商务、科技、文化、教育、宗教活动过程中的旅游活动），出行距离超过 10 公里，出游时间超过 6 小时，但不超过 12 个月的我国大陆居民。

入境旅游者 指来我国参观、访问、旅行、探亲、访友、休养、考察、参加会议和从事经济、科技、文化、教育、体育、宗教等活动的外国人、华侨、港澳和台湾同胞的人数。不包括外国在我国的常驻机构，如领事馆、通讯社、企业办事处的工作人员；来我国常住的外国专家、留学生以及在岸逗留不过夜人员。

Explanatory Notes on Main Statistical Indicators

Business Revenue of Hotels and Catering Services refer to revenue received from providing services or selling commodities by corporate enterprises and establishments engaged in hotel and catering services，including income from hotel rooms，from catering services，from selling of commodities and from other services. Income from hotel rooms refers to income of corporate enterprises and establishments by providing lodging services. Income from catering services refers to income of corporate enterprises and establishments by providing catering services，including selling of cooked or prepared foods such as stable food，cooked dishes or cold dishes. Income from selling of commodities refers to income of corporate enterprises and establishments by selling commodities that accompany the services they provide. Income from other activities refers to income received other than income from hotel rooms，catering services or selling of commodities，such as income from providing recreation，fitness or business services.

Domestic Tourists refers to residents in mainland China who are not for the purpose of seeking employment，remuneration，and leaving the usual living environment，elsewhere to engage in domestic visitors，sightseeing，vacation travel（including to go out to visit relatives，infirmary，observing，participate in the meeting and engage in business，science and technology，culture，education，religious activities in the course of tourism activities），trip distance of more than 10 km，trips longer than six hours，but not more than 12 months.

Entrance Tourists refers to foreigners，overseas Chinese，Chinese compatriots from Hong Kong，Macao and Taiwan coming to China for sight-seeing，visits，tours，family reunions，vacations，study tours，conferences and other activities of a business，scientific and technological，cultural，educational and religious nature. It does not include representatives and employees of resident institutions of foreign countries in China such as embassies，consulates，news agencies and offices of foreign companies and organizations，nor does it include long-term foreign experts or students residing in China，or persons in transition without spending a night in China.

教育、科技和文化 17

EDUCATION SCIENCE &
TECHNOLOY AND CULTURE

简 要 说 明

一、本篇资料的主要内容

本篇资料主要反映了全市教育、科技和文化事业基本情况。教育部分主要包括高等教育、中等教育、初等教育、成人教育、职业教育、幼儿园等方面的基本情况。科技部分主要包括科研机构、科学技术奖励、大中型工业企业技术开发情况。文化部分主要包括文化、文物、广播、电视、报纸杂志出版、图书出版等方面的发展状况。

二、本篇资料的来源

1、教育部分，技工学校的资料来源于市人力资源和社会保障局，其他资料来源于市教育局。

2、科技部分，科研机构及科技奖励资料来源于市科技局，大中型工业企业科技活动资料来源于市统计局统计调查年报。

3、文化部分，图书、杂志、报纸出版有关资料来源于青岛出版集团、海大出版社其他资料来源于市文化广电新闻出版局。

本篇资料由市统计局人口和社会科技统计处整理提供。

Brief Introduction

I. Main Content

Data in this chapter show the basic conditions of education, science & technology and culture. Data on education show the development of higher education, secondary education, primary education, adult education, vocational education and kindergartens. Data on science & technology show the basic conditions of science research institutions, scientific and technological achievements and prizes, scientific & technological activities of large and medium-size industrial enterprises. Data on culture show the basic conditions of arts, cultural relics, broadcasting, television and publication of newspapers, magazines and books.

II. Source of Data

(1) In education component, data on the basic conditions of technical schools are provided by Qingdao Municipal Bureau of Human Resources and Social Security, and the other data on education are provided by Qingdao Municipal Bureau of Education.

(2) In science & technology component, data on science research institutions and scientific & technological achievements and prizes are provided by Qingdao Municipal Bureau of Science and Technology. Data on scientific and technological activities are from the annual report of scientific and technological activities, which is provided by Qingdao Municipal Bureau of Statistics.

(3) In culture component, data on publication of books, magazines and newspapers are provided by Qingdao Publishing Group and Ocean University Press. The other data are provided by Qingdao Municipal Bureau of Cluture, Broadcasting, Television, Press and Publication.

Data in this chapter are provided and compiled by the Division of Population and Science & Technology of Qingdao Municipal Bureau of Statistics.

17-1 各级各类学校基本情况（2018 年）

BASIC STATISTICS ON SCHOOLS BY LEVEL AND TYPE OF SCHOOL（2018）

项目	Item	学校数（所）Schools（unit）	毕业生数（人）Graduates（person）	招生数（人）New Students Enrollment(person)	在校学生数（人）Students Enrollment(person)	教职工数（人）Teachers and Staff（person）	#专任教师 of which：Full-time Teachers
研究生	Postgraduates	8	11044	15679	41145	21935	8346
普通高等学校	Regular Institutions of Higher Education	25	105580	118247	397982	32628	21576
中等专业学校	Specialized Secondary Schools	6	10881	10390	31879	879	670
技工学校	Technical Schools	22	7054	9201	32110	2296	1737
职业学校	Vocational Schools	42	15801	15892	51440	7251	5994
普通中学	Regular Secondary Schools	319	110168	124356	385443	67526	36007
初中	Junior Secondary Schools	247	72370	86103	268336	25848	24007
高中	Senior Secondary Schools	72	37798	38253	117107	41678	12000
小学	Primary Schools	713	86070	103988	569923	35866	36890
特殊教育学校	Special Education Schools	13	235	292	1861	644	547
幼儿园	Kindergartens	2241	90149	90986	263890	32428	20957
成人高等学校	Adult Institutions of Higher Education	1	51281	45664	82721		
成人中等学校	Adult Institutions of Higher Education	2	1021	9	974	36	28
成人初等学校	Adult Primary Education Schools						

17-2 主要年份各级各类学校在校学生数
MAJOR YEAR'S STUDENTS ENROLLMENT OF SCHOOLS BY LEVEL AND TYPE OF SCHOOL

单位：人（person）

年份 Year	普通高等学校 Pegular Institutions of Higher Education	中等学校 Secondary Schools	中等专业学校 Specialized Secondary Schools	普通中学 Regular Secondary Schools	职业中学 Vocational Schools	技工学校 Technical Schools	小学 Primary Schools
1949	1 007	13 804	1 600	12 204			213 470
1952	2 761	27 951	3 770	24 181			413 286
1957	3 133	56 689	4 985	51 604	100		482 397
1962	4 381	62 646	2 791	58 639	243	973	522 921
1965	2 987	99 605	9 405	88 187	1 490	523	788 485
1970	320	222 521	105	220 262	2 154		728 433
1975	1 151	350 309	3 144	346 403		762	928 153
1978	3 465	464 584	4 269	457 460	2 000	865	634 905
1980	6 783	325 935	5 195	318 193	1 344	1 203	816 970
1985	10 631	327 446	11 475	279 915	33 945	2 111	691 341
1988	14 408	352 576	11 819	306 943	28 933	4 881	629 212
1990	15 433	351 365	15 118	296 163	34 568	5 516	632 314
1991	15 491	353 410	14 618	298 568	34 421	5 803	612 772
1992	16 470	367 480	14 763	307 903	38 242	6 572	585 381
1993	23 858	383 043	16 877	318 179	40 743	7 244	583 585
1994	25 018	420 077	20 582	347 667	43 734	8 094	593 419
1995	24 908	450 212	23 744	372 335	46 044	8 089	595 891
1996	26 076	468 561	26 951	382 760	50 131	8 719	607 284
1997	27 434	463 619	28 543	362 305	61 640	11 131	626 749
1998	29 507	442 525	28 757	336 918	65 989	10 861	625 308
1999	33 681	459 520	26 955	352 238	70 236	10 091	583 594
2000	46 131	497 391	25 192	398 458	63 894	9 847	534 922
2001	60 728	542 400	25 358	443 693	62 003	11 346	499 147
2002	82 539	575 599	27 421	467 628	67 085	13 465	478 634
2003	168 439	586 704	28 948	461 435	75 221	21 100	467 560
2004	201 739	598 804	30 881	436 117	101 553	30 253	476 897
2005	239 761	569 230	33 522	391 148	106 400	38 160	479 781
2006	260 339	554 308	32 051	365 192	116 487	40 578	483 892
2007	264 917	558 527	28 011	360 410	130 156	39 950	484 775
2008	269 314	582 932	25 330	373 884	143 126	40 592	477 230
2009	275157	564476	22739	377427	127792	36518	465031
2010	284788	535079	24014	380025	98560	32480	462722
2011	291453	505051	25905	373226	74892	31028	479513
2012	296645	489103	27005	369580	65419	27099	484985
2013	300246	484025	28389	365110	61594	28932	496343
2014	313486	479260	28751	362599	58170	29740	516529
2015	322260	467231	33038	355401	48590	30202	536492
2016	340875	470333	32710	356107	50927	30589	547231
2017	346328	490796	32882	372196	52539	33179	550540
2018	397982	500872	31879	385443	51440	32110	569923

17-3 主要年份普通高等学校基本情况
MAJOR YEAR'S BASIC STATISTICS ON REGULAR INSTTUTIONS OF HIGHER EDUCATION

单位：人（person）

年份<br>Year	学校数（所）<br>Schools<br>（unit）	毕业生数<br>Graduates	招生数<br>New Students<br>Enrollment	在校学生数<br>Students<br>Enrollment	教职工数<br>Teachers and<br>Staff	#专任教师<br>of which：<br>Full-time Teachers
1949	1		250	1 007	779	226
1952	2	262	565	2 761	12 530	308
1957	2	356	755	3 133	1 308	622
1962	5	647	653	4 381	1 866	749
1965	3	741	529	2 987	1 539	623
1970	2	684		320	1 178	486
1975	2	460	473	1 151	1 353	582
1978	4	739	1 782	3 465	2 829	1 176
1980	6	431	1 717	6 783	4 182	1 606
1985	7	1 828	3 823	10 631	5 561	2 181
1988	7	3 496	4 687	14 408	6 875	2 499
1990	7	4 495	4 745	15 433	7 305	2 624
1991	7	4 880	4 954	15 491	7 282	2 489
1992	7	4 458	5 428	16 470	7 344	2 523
1993	7	3 140	9 449	23 858	7 366	2 520
1994	4	7 059	8 042	25 018	7 466	2 650
1995	4	7 828	7 978	24 908	7 545	2 682
1996	4	6 691	7 956	26 076	7 606	2 745
1997	4	7 091	8 503	27 434	7 790	2 813
1998	4	6 912	8 849	29 507	7 662	2 907
1999	4	7 202	11 584	33 681	7 662	2 928
2000	6	7 128	18 427	46 131	8 030	3 278
2001	6	8 991	22 787	60 728	8 706	4 058
2002	7	13 053	29 731	82 539	9 517	4 723
2003	25	25 747	61 975	168 439	18 911	10 293
2004	25	34 860	70 022	201 739	21 437	12 347
2005	25	46 114	79 725	239 761	23 078	13 894
2006	25	55 931	79 094	260 339	24 881	15 195
2007	25	68 354	78 728	264 917	25 956	16 005
2008	25	75 198	86 878	269 314	26 654	16 805
2009	25	69475	81025	275157	26983	16870
2010	25	70450	82220	284788	28145	16996
2011	22	79466	84434	291453	28151	17120
2012	22	79791	87981	296645	29445	18183
2013	22	78974	85707	300246	28958	18396
2014	22	77503	90127	313486	28944	18587
2015	24	79784	92251	322260	29620	19213
2016	26	86270	100525	340875	30390	20151
2017	25	95006	98983	346238	32013	21231
2018	25	105580	118247	397982	32628	21576

17-4　各类成人教育基本情况（2018年）
BASIC STATISTICS ON ADULT EDUCATION（2018）

单位：人（person）

各类学校	Schools by Type of School	学校数（所）Schools（unit）	毕业生数 Graduates	招生数 New Students Enrollment	在校学生数 Students Enrollment	教职工数 Teachers and Staff	#专任教师 of which：Full-time Teachers
**总　计**	**Total**						
成人高等教育	Adult Higher Education	1	51281	45664	82721		
广播电视大学	Broadcasting and TV Universities						
职工、农民大学	Universities of Vocational and Agricultural Education						
函授、夜大学	Correspondence and Evening College Education						
管理干部学校	Cadre Management Schools						
成人中等教育	Adult Secondary Education	2	1021	9	974	36	28
成人中等专业学校	Adult Specialized Secondary Schools	2	1021	9	974	36	28

17-5 分市、区普通中学情况（2018 年）
BASIC STATISTICS ON REGULAR SECONDARY SCHOOLS BY REGION (2018)

单位：人（person）

市、区名称	Region	普通高中 Regular Senior Secondary Schools				普通初中 Regular Junior Secondary Schools			
		学校数 Schools（unit）	毕业生数 Graduates	招生数 New Students Enrollment	在校生数 Students Enrollment	学校数 Schools（unit）	毕业生数 Graduates	招生数 New Students Enrollment	在校生数 Students Enrollment
**全　市**	**Whole Municipality**	**72**	**37798**	**38253**	**117107**	**247**	**72370**	**86103**	**268336**
市　直	Municipal Level	22	9153	9702	29711	3	2486	2614	7765
市南区	Shinan District	0	0			10	2315	2753	8146
市北区	Shibei District	1	0	180	331	25	5773	7109	20939
李沧区	Licang District	2	0	18	47	12	3177	4028	11640
崂山区	Laoshan District	2	0			10	1903	2319	7151
黄岛区	Huangdao District	11	6375	5745	18333	40	12149	14980	45726
城阳区	Chengyang District	6	2700	2712	8228	16	5275	7010	20643
即墨区	Jimo District	9	5382	5916	18233	30	11733	14268	41722
胶州市	Jiaozhou	7	4234	4424	13162	23	8920	10548	33610
平度市	Pingdu	7	6917	6245	18972	45	11634	12095	39115
莱西市	Laixi	4	3037	3240	10019	29	6371	7583	29539
红岛经济区	Qingdao National High-tech Industrial Development Zone	1	0	71	71	4	634	796	2340

注：黄岛区含保税港区数据。
Note：The Huangdao District contains the data of the bonded harbor area.

17-6 分市、区职业中学、小学情况（2018 年）
BASIC STATISTICS ON VOCATIONAL SECONDARY SCHOOLS AND PRIMARY SCHOOLS BY REGION（2018）

单位：人（person）

市、区名称	Region	职业中学 Vocational Secondary Schools				小学 Primany School			
		学校数 Schools（unit）	毕业生数 Graduates	招生数 New Students Enrollment	在校生数 Students Enrollment	学校数 Schools（unit）	毕业生数 Graduates	招生数 New Students Enrollment	在校生数 Students Enrollment
**全　市**	**Whole Municipality**	**50**	**27703**	**26291**	**84293**	**713**	**86070**	**103988**	**569923**
市　直	Municipal Level	30	11206	9964	32734		45	67	313
市南区	Shinan District					28	4488	5922	32529
市北区	Shibei District					68	7512	10965	59050
李沧区	Licang District					37	4329	7509	37250
崂山区	Laoshan District					27	2790	4474	24333
黄岛区	Huangdao District	10	5889	5840	18832	90	14941	19038	101670
城阳区	Chengyang District	3	2879	2736	8036	46	7320	12064	61620
即墨区	Jimo District	2	2041	1781	5944	169	14031	14032	83937
胶州市	Jiaozhou	1	2568	2153	7322	74	10151	10875	62449
平度市	Pingdu	1	1632	1228	4767	100	12151	11499	69058
莱西市	Laixi	3	1488	2589	6658	70	7547	6291	32238
红岛经济区	Qingdao National High-tech Industrial Development Zone					4	765	1252	5476

注：黄岛区含保税港区数据。
Note：The Huangdao District contains the data of the bonded harbor area.

17-7　分市、区中小学教职工情况（2018 年）
BASIC STATSTICS ON TEACHERS AND STAFF IN SECONDARY AND PRIMARY SCHOOLS BY REGION（2018）

单位：人（person）

市、区名称	Region	普通中学 Regular Secondary Schools		职业中学 Vocational Secondary Schools		小学 Primany School	
		教职工数 Teachers and Staff	# 专任教师 of which：Full-time Teachers	教职工数 Teachers and Staff	# 专任教师 of which：Full-time Teachers	教职工数 Teachers and Staff	# 专任教师 of which：Full-time Teachers
**全　市**	**Whole Municipality**	**41678**	**38321**	**8166**	**6692**	**35866**	**34576**
市　直	Municipal Level	4071	3325	3387	2549		
市南区	Shinan District	1087	963			2104	2003
市北区	Shibei District	2451	2266			3565	3409
李沧区	Licang District	1400	1252			1994	1922
崂山区	Laoshan District	998	935			1606	1547
黄岛区	Huangdao District	6887	6384	1488	1305	6166	5982
城阳区	Chengyang District	3013	2750	661	581	3036	2867
即墨区	Jimo District	6529	6193	713	623	5467	5305
胶州市	Jiaozhou	4229	3869	632	504	3963	3883
平度市	Pingdu	6289	5929	651	625	5122	4938
莱西市	Laixi	4133	3887	634	505	2526	2405
红岛经济区	Qingdao National High-tech Industrial Development Zone	591	568			317	315

注：黄岛区含保税港区数据。
Note：The Huangdao District contains the data of the bonded harbor area.

17-8 分市、区幼儿园基本情况（2018 年）
BASIC STATISTICS ON KINDERGARTENS BY REGION (2018)

单位：人（person）

市、区名称	Region	幼儿园（所）Kindergartens（unit）	幼儿数 Children	教职工数 Teachers and Staff	#专任教师 of which：Full-time Teachers
**全　市**	**Whole Municipality**	**2241**	**263890**	**32428**	**20957**
市南区	Shinan District	65	14064	2361	1132
市北区	Shibei District	115	28029	3083	1956
李沧区	Licang District	57	20969	2687	1360
崂山区	Laoshan District	114	13941	2275	1374
黄岛区	Huangdao District	443	47474	6711	4158
城阳区	Chengyang District	151	25456	3637	2398
即墨区	Jimo District	361	35556	3791	2593
胶州市	Jiaozhou	355	29507	3285	2327
平度市	Pingdu	405	30533	2562	2053
莱西市	Laixi	146	15153	1583	1237
红岛经济区	Qingdao National High-tech Industrial Development Zone	29	3208	453	369

注：黄岛区含保税港区数据。
Note: The Huangdao District contains the data of the bonded harbor area.

17-9 科研机构基本情况（1978-2018 年）
BASIC STATISTICS ON SCIENTIFIC RESEARCH INSTITUTIONS（1978-2018）

年份<br>Year	独立自然科研结构（个）<br>Independent Institutions of Natural Scientific Research（unit）	独立自然科研结构中科技人员（个）<br>Personnel of Independent Institutions of Natural Scientific Research（person）	完成科研项目（项）<br>Number of Scientific Research Projects Completed（item）	取得科技成果（项）<br>Number of Achievements in S&T（item）
1978	31	2701	306	306
1980			481	297
1982	41	2345	451	451
1983	48	3182	530	530
1984	53	3312	507	507
1985	52	3673	552	552
1986	63	3998	577	577
1988	101	5199	544	510
1990	141	6046	414	931
1991	72	5665	753	753
1992	71	5726	659	659
1993	69	5545	546	546
1994	69	5540	467	467
1995	59	5286	441	441
1996	58	4738	613	613
1997	54	4577	418	418
1998	52	4142	517	517
1999	58	3771	606	606
2000	54	3637	621	621
2001	50	3191	457	457
2002	52	3248	406	406
2003	53	3246	589	589
2004	50	3226	506	506
2005	50	3234	438	438
2006	50	3291	548	548
2007	50	3432	506	506
2008	42	3709	416	416
2009	43	3727	572	572
2010	44	4088	472	472
2011	47	4548	345	345
2012	48	4967	304	304
2013	48	6057	433	433
2014	50	5438	415	415
2015	54	6037	639	639
2016	57	5722	606	606
2017	57	6017	588	588
2018	58	6017	406	406

注：1991 年以后不包括民办科研机构。
Note：Since 1991，private institutions of scientific research are not included.

17-10 独立科学研究机构情况（2018 年）
BASIC STATISTICS ON INDEPENDENT INSHUTIONS OF SCIENTIFIC RESEARCH（2018）

项目	Item	计量单位	Unit	总 计 Total	中央属 Central	地方属 Local
机构数	Number of Institutions	个	unit	58	22	36
职工人数	Staff and Workers	人	person	8544	6470	2074
科技人员	Personnel Engaged in S&T	人	person	6107	4455	1652
经费收入	Funding for S&T	万元	10 000 yuan	406021	291193.7	114827.3
经费支出	Expenditures for S&T	万元	10 000 yuan	403589.4	305614.2	97975.2

17-11 科学技术奖励情况（2018 年）
AWARD STATISTICS ON SCIENCE AND TECHNOLOGY（2018）

项目	Item	单位	Unit	自然科学奖 Natural Science Prizes Awarded	技术发明奖 Invention Prizes Awarded	科技进步奖 Scientific and Technological Progress Prizes Awarded	科学技术功勋奖 Scientific and Technolgical Credit Prizes Awarded	国际科技合作奖 International S&T Cooperation Prizes Award	最高奖 Highest Prizes Award
国家级	National Level	项	Item	1	1	11		1	
特等	Special Grade								
#一等	First Prize	项	Item			2			
二等	Second Prize	项	Item	1	1	9			
省级	Provincial Level	项	Item	6	7	52			1
#一等	First Prize	项	Item	1	0	10			
二等	Second Prize	项	Item	5	5	27			
三等	Third Prize	项	Item		2	15			
市级	Municipal Level	项	Item	11	5	129		1	1
#一等	First Prize	项	Item	2	0	14			
二等	Second Prize	项	Item	6	3	69			
三等	Third Prize	项	Item	3	2	46			

注：国际科技合作奖、最高奖不分等级。
Note：There is no grade in international scientific and technological cooperation prizes award and highest prizes award.

17-12 文化、文物事业机构、人员数（2018 年）

NUMBER OF INSTITUTIONS AND PERSONNEL IN CUUTURE（2018）

项目	Item	文化机构数（个）Number of Cultural Institutions（unit）		文化人员数（人）Number of Cultural Personnel（Person）	
		2018 年	2017 年	2018 年	2017 年
**一、艺术事业**	**Art**				
1. 艺术表演团体	Art Peformance Troupes	9	8	830	575
2. 艺术表演场所	Art Centers	10	10	201	197
3. 艺术展览创作机构	Art Creation Institutions	5	5	28	33
4. 艺术研究机构	Art Research Institutions				
5. 艺术展览机构	Art Exhibition Institutions				
6. 其他	Others	3	3	24	27
**二、图书馆事业**	**Libraries**				
公共图书馆	Publie Libraries	12	12	279	275
#县（区）级图书馆	County Libraries	11	11	170	175
**三、群众文化事业**	**Nass Culture**				
1. 群众艺术馆	Nass Art Centers				
2. 文化馆	Cultural Palaces	12	12	214	225
3. 文化站	Cultural Centers	136	136	527	500
4. 其他	Others				

17-13 文化部门艺术剧团情况（2018 年）
STATISTICS ON ART TROUPES OF CULTURAL DEPARTMENT（2018）

项目	Item	剧团数（个）Number of Troupes（unit）	职工人数（人）Stalf and Workers（person）	演出场次（场）Number of Performances（show）	观众人数（万人次）Number of Spectators（10000 person-times）	演出收入（万元）Income from Ticket Sales（10000 yuan）	# 财政补贴（万元）Apporopriation Funds（10000 yuan）
**总　计**	**Total**	**8**	**574**	**1903**	**120.4**	**12717.0**	**10848.5**
**一、按隶属关系分**	**Grouped by Administrative Relationship**						
国营剧团	State-run Troupes	8	574	1903	120.4	12717.0	10848.5
集体经营剧团	Collective-owned Troupes						
**二、按剧种分**	**Grouped by Type of Drama**						
1. 话剧团	Drama Troupes	1	110	405	20.3	2450.9	2008.3
2. 歌舞团	Song and Dance Troupes						
3. 戏曲剧团	Local Opera Troupes	6	371	1416	93.6	6332.2	5764.4
# 京剧	of which：Beijing Opera	1	87	116	5.5	1759.3	1661.7
4. 曲艺团	Recitation and Ballad Troupes						
5. 文工团（艺术团）	Cultural and Performance Troupes	1	93	82	6.5	3933.9	3075.8

17-14 图书馆、文化馆情况
STATISTICS ON LIBRARIES AND CULTURAL CENTERS

项目	Item	单位	2018 年		2017 年	
			全市合计 Total	# 市区 Urban Area	全市合计 Total	# 市区 Urban Area
**公共图书馆**	**Pubilc Libraries**	个	**12**	**9**	**12**	**9**
工作人员	Staff and Workers	人	279	220	275	225
藏书册数	Colicetion Books	千册	7479	6540	6996	6108
阅览席位	Seating Capacity of Reading Rooms	张	5866	4609	6412	5351
读者人数	Readers	万人次	469.9	402.1	482.1	419.3
**文化馆**	**Cultural Centers**	个	**12**	**9**	**12**	**9**

主要统计指标解释

普通高等学校　指按照国家规定的设置标准和审批程序批准举办，通过国家统一招生考试，招收高中毕业生为主要培养对象，实施高等教育的全日制大学、独立设置的学院和高等专科学校、短期职业大学。

成人高等学校　指按照国家有关规定审批，招收通过全国成人高教统一招生考试的具有高中毕业或同等学历的在职从业人员，利用脱产、半脱产、业余或函授等多种形式对其实施高等学历教育，培养高等教育专科或本科毕业水平的专门人才，修业年限、课程设置和总学时数均按高等学历教育要求付诸实施的学校。包括广播电视大学、职工高等学校、农民高等学校、管理干部学院；教育学院、独立设置的函授学院等。

科技活动人员　指直接从事科技活动、以及专门从事科技活动管理和为科技活动提供直接服务，累计的实际工作时间占全年制度工作时间 10% 及以上的人员。

文化事业机构　指从事专业文化工作和为专业文化工作服务的独立建制的单独核算的单位。不包括这些单位另外举办独立核算的其他机构和各部门的业余文化组织。

Explanatory Notes on Main Statistical Indicators

Regular Institutions of Higher Education　refer to educational establishments set up according to the government evaluation and approval procedures，enrolling graduates from senior secondary schools and providing higher education courses and training for senior professionals. They include full-time universities，colleges，high professional schools，high professional vocational schools and others.

Adult Institutions of Higher Education　refer to educational establishments，set up in line with relevant rules approved by the government，enrolling staff and workers with senior secondary school or equivalent education，and providing higher education courses in many forms of correspondence，spare time，or full time for adults. Professionals thus trained receive a qualification equivalent to graduates studying regular courses at regular universities，colleges and professional colleges. Institutions of higher learning for adults include schools of high education for staff and workers，schools of high education for peasants，colleges for management cadres，pedagogical colleges，independent correspondence colleges，Radio and TV universities and other educational establishments.

Personnel Engaged in S&T Activities　refer to personnel directly engaged in S&T activities，in the management of S&T activities，and in providing direct service to S&T activities，who spend over 10% of the total working hours in a year in S&T activities.

Cultural Institutions　refer to units，which have their own organizational system and independent accounting system and specialize in or serve cultural development. They exclude other establishments run by these cultural institutions and amateur cultural groups established by various departments.

体育、卫生和民政、司法 18

SPORTS，PUBLIC HEALTH AND CIVIL AFFAIRS，JUDICIAL AFFAIRS

简要说明

一、本篇资料的主要内容

本篇资料反映了全市体育、卫生、民政、司法等社会事业的基本情况。体育部分主要包括主要年份运动员、教练员、裁判员发展人数、体育运动破纪录、获奖情况。卫生部分主要包括各类卫生机构及其人员、床位数。民政部分主要包括婚姻登记、社会救济、社会福利事业基本情况、殡葬服务情况。司法部分主要包括律师、公证、调解、社会治安基本情况。

二、本篇资料的来源

1、体育部分来源于市体育局。

2、卫生部分来源于市卫计委。

3、民政部分来源于市民政局。

4、司法部分，社会治安资料来源于市公安局，其他资料来源于市司法局。

本篇资料由市统计局人口和社会科技统计处整理提供。

Brief Introduction

I. Main Content

Data in this chapter show the basic conditions of sports，public health，civil affairs and judicial affairs. Data on sports are mainly including the number of athletes，coaches and referees，conditions of record and awards. Data on public health are mainly including the number of health institutions，personnel and beds. Data on civil affairs are mainly including the conditions of marriage registration，social relief，social welfare and funeral services. Data on judicial affairs are mainly including the conditions of lawyers，notarization，mediation and social order.

II. Source of Data

(1) Data on sports are provided by Qingdao Municipal Bureau of Physical.

(2) Data on public health are provided by Qingdao Municipal Bureau of Health.

(3) Data on civil affairs are provided by Qingdao Municipal Bureau of Civil Affairs.

(4) In judicial affairs component，data on social order are provided by Qingdao Municipal Bureau of Public Security and the other data are provided by Qingdao Municipal Bureau of Justice.

Data in this chapter are provided and compiled by the Division of Population and Science & Technology of Qingdao Municipal Bureau of Statistics.

18-1　体育事业情况 (2000-2018 年)
STATISTICS ON SPORTS（2000-2018）

单位：人 (person)

项　目	Item	2000	2005	2007	2008	2009	2010	2011	2012	2013	2014	2015	2016	2017	2018
**体育部门职工人数**	**Staff and Workers in Sports Commissions**	**849**	**834**	**772**	**1210**	**910**	**1015**				**847**	**795**	**857**	**654**	**682**
#运动员	Athletes	90	87	55	452	512	287				287	259		284	1123
教练员	Coaches	174	235	211	173	106	170	136					120	133	134
重点体校	Key Sports School	126	117	249	109	97	115	129						1061	1370
业余体校	Sparetime Sports Schools	142	223	145		160								1537	1846
体育专业队	Professional Sports Teams	135	132	121		144	192	87	84	42	38	17	17	5	5
优秀运动员	Exeellent Athletes	90	87	55	71	72	146	57							
优秀运动队专职教练员	Full-time Coaches of Excellennt Sports Teams	18	23	47	106	92	97	54				89	120		
**等级裁判员发展人数**	**Certified Referees**	**1114**	**275**	**781**	**205**	**292**	**212**	**510**	**558**	**512**	**514**	**490**	**167**		**511**
#二级	Second Grades	451	275	781	181	252	169	510	484	463	514	490	116		319
三级	Third Grades	616													
**等级运动员发展人数**	**Certified Athletes**	**343**	**260**	**108**	**310**	**512**	**229**	**436**	**362**	**362**	**283**	**259**		**176**	**327**
#二级	Second Grades	209	260	108	281	448	229	436	256	362	283	259		176	290
三级	Third Grades	46													
少年级	Juvenile	88													

18-1 续表
Contiuned

单位：人（person）

项　目	Item	单位	Unit	2000	2005	2007	2008	2009	2010	2011	2012	2013	2014	2015	2016	2017	2018
**全年获得奖牌数**	**Number of Medals Won in the Year**	枚	**unit**	**347.5**	**669**	**481**	**494**	**602**	**754**	**829**	**746**	**659**	**576.5**	**596**	**729.5**	**3032**	**849**
# 国家级金牌	Golden Medals at National Level	枚	unit	45	62	61	26	30	14	105	88	90	48	51	101	104	76
国家级银牌	Silver Medals at National Level	枚	unit	28	48	38	21	23	7	49	100	44	21	44	56	91	81
省级金牌	Golden Medals at Provincial Level	枚	unit	116.5	187	116	168	207	459	225	193	174.5	202.5	149	219.5	214.5	264
省级银牌	Silver Medals at Provincial Level	枚	unit	62	174	93	122	149	116	155	110	111.5	121.5	126	135	140	161
**体育运动破全国记录**	**Records Broken the National Records**																
项目	Number of Events	项	item		2	1		1	1	2	1						1
人数	Number of Persons	人	person		2	1		1	1	2	1						1
次数	Number of Times	人次	person-tims		4	1		1	1	2	1						1
**体育运动破全省记录**	**Records Broken the Provincial Records**																
项目	Number of Events	项	item	7	3		2		1								
人数	Number of Persons	人	person	7	1		2		1								
次数	Number of Times	人次	person-tims	7	3		2		1								15

18-2　主要年份卫生事业基本情况

MAJOR YEAR'S BASIC STATISTICS ON PUBLIC HEALTH

单位：人（person）

年　份<br>Year	卫生机构数（个）<br>Health Institutions（unit）	# 医院<br>of which：Hospitals	医疗床位数（张）<br>Beds in Health Institutions（bed）	# 医院<br>of which：Hospitals	卫生技术人员（个）<br>Medical and Technical Personnel（person）	# 医生<br>of which：Hospitals	每千人口拥有医生数（人）<br>Number of Doctors per 1000 Population（person）	每千人口拥有床位数（张）<br>Number of Hospital Beds per 1000 Population（bed）
1949	80	26	1079	970	2637	1292	0.32	0.27
1952	260	34	2943	1691	3748	1559	0.37	0.70
1957	624	45	5163	2656	6941	2578	0.53	1.07
1962	849	149	7167	4526	8279	2972	0.64	1.55
1965	912	162	8428	5003	9051	3586	0.73	1.72
1970	712	170	8613	6603	9591	3761	0.70	1.60
1975	959	181	10902	9078	13558	4566	0.80	1.90
1978	1192	212	13084	10709	16921	5893	1.01	2.24
1980	1253	212	14170	11345	19265	6781	1.14	2.38
1985	1517	219	18469	13214	23413	9694	1.55	2.95
1990	1563	235	23541	16586	27932	13761	2.08	3.56
1991	1579	235	23727	16796	27772	13227	1.98	3.54
1992	1562	236	24765	17269	28305	13157	1.95	3.67
1993	1540	237	24885	17469	28705	13312	1.97	3.68
1994	1335	242	24732	17882	28938	13535	1.99	3.64
1995	1347	228	25282	18126	29816	13840	2.02	3.69
1996	2216	228	24334	18344	30048	13790	2.00	3.53
1997	2193	239	25201	19046	31121	14400	2.07	3.62
1998	2190	237	24887	19083	31623	14327	2.05	3.56
1999	2185	235	25481	19798	31965	14453	2.06	3.62
2000	2911	231	24392	20057	32160	14860	2.10	3.45
2001	3199	217	24755	20313	32765	15772	2.22	3.48
2002	3050	213	21370	20662	31469	13371	1.87	2.99
2003	3111	213	23980	21605	32337	13452	1.87	3.33
2004	2923	219	25432	23465	32937	13953	1.91	3.48
2005	2609	235	30627	28564	33942	15008	2.03	4.13
2006	2834	232	28724	26653	34360	15451	2.06	3.83
2007	2006	251	30062	28107	33807	15018	1.98	3.97
2008	1985	249	32350	29874	36574	16260	2.14	4.25
2009	2017	252	32803	30527	40217	16734	2.19	4.30
2010	2147	254	36066	33383	43285	17696	2.32	4.72
2011	2549	280	39980	35600	47347	18310	2.39	5.22
2012	2607	288	47254	41186	54488	21593	2.81	6.14
2013	2936	285	44876	39725	59961	24113	3.12	5.80
2014	3126	297	47081	42961	62738	24946	3.20	6.03
2015	3146	308	48601	45066	65264	26270	3.35	6.20
2016	7564	322	50649	47309	87733	27675	3.49	6.39
2017	7927	410	55798	46282	76146	30867	3.84	6.94
2018	8027	421	53154	50842	83975	34578	4.23	6.82

注：自 2016 年起，卫生机构及人员包含村卫生室。
Note：Health agency and personnel includes village clinic as of 2016.

18-3 各类卫生机构、床位、人员数（2018 年底）

NUMBER OF HEALTH INSTITUTIONS，BEDS AND EMPLOYED PERSONS（END OF 2018）

项　　目	Item	机构数（个）Health Institutions（unit）	床位数（张）Beds in Health Institutions（bed）	人员数（人）Personnel（person）
**总　　计**		**8027**	**53154**	**102991**
**一、医院合计**	**Sub-total of Hospitals**	**421**	**50842**	**71213**
1. 医院	Hospitals	318	45136	63621
# 综合医院	General Hospitals	179	27718	42017
中医医院	Hospitals Specialized in Traditional Chinese Medicine	35	6028	8012
中西医结合医院	Hospitals of Combination of Chinese and Western Medicine	6	762	932
专科医院	Specialized Hospitals	95	10433	12562
# 口腔医院	Stomatological Hospitals	8	140	653
眼科医院	Ophthalmology Hospitals	8	585	903
肿瘤医院	Tumor Hospitals	3	660	592
心血管病医院	Cardiovascular Diseases Hospitals	3	870	1080
精神病医院	Mental Hospitals	14	2434	1607
传染病医院	Infectious Disease Hospitals	2	490	516
皮肤病医院	The Skin Disease Hospital	1	40	50
骨科医院	Orthopaedics Hospitals	6	404	346
2. 卫生院	Sanitation Stations	103	5706	7592
**二、社区卫生服务中心**	**Neighborhood Service Centers**	**72**	**616**	**3009**
**三、门诊部**	**Outpatient Service Stations**	**363**		**5461**
**四、诊所**	**Clinics**	**2243**		**8906**
**五、卫生所、医务室、护理站**	**Medical Houses Nursing Station**	**311**		**914**
**六、社区卫生服务站**	**Neighorhood Service Stations**	**208**	**48**	**2840**
**七、急救中心**	**First Aid Centers**	**3**		**178**
**八、采供血机构**	**Institutions of Blood Collection and Supply**	**1**		**253**
**九、妇幼保健院（所、站）**	**Maternity and Child Care Centers**	**12**	**524**	**1499**
**十、专科疾病防治院（所、站）**	**Specialized Disease Prevention & Treatment Institutions**	**6**	**224**	**253**
**十一、疾病预防控制中心（防疫站）**	**Centers for Disease Control and Prevention（Epidemic Prevention Stations）**	**26**		**966**
**十二、卫生监督所**	**Sanitation Control Stations**	**11**		**373**
**十三、卫生监督检验所（站）**	**Sanitation Control and Test Stations**			
**十四、计划生育技术服务机构**	**Family Planning Service Institutions**	**23**		**118**
**十五、临床检验中心（所、站）**	**Clinical Laboratory Center**	**3**		**202**
**十六、健康教育所（站、中心）**	**Health Education Stations**			
**十七、其它卫生机构**	**Other Health Institutions**	**37**		**304**
**十八、疗养院**	**Sanatoriums**	**4**	**900**	**432**
**十九、村卫生室**	**Village clinic**	**4283**		**6070**

# 卫生技术人员 Medical and Technical Personnel	# 医生 Doctos	注册护士 Registered Nurses	药剂人员 Pharmacists	检验人员 Laboratory Technicians
**83975**	**34578**	**38360**	**3988**	**2615**
**60286**	**21812**	**29944**	**3165**	**2050**
53583	18999	27581	2660	1745
35891	12873	18595	1666	1114
6816	2515	3285	453	202
774	268	358	77	44
10035	3318	5314	456	383
502	269	216	6	4
621	197	350	20	18
517	172	304	17	13
830	299	431	45	29
1362	337	882	64	36
442	148	230	24	27
34	15	12	5	2
266	85	134	14	8
6703	2813	2363	505	305
**2516**	**1098**	**974**	**223**	**92**
**4672**	**2317**	**1863**	**199**	**143**
**8763**	**5304**	**3324**	**102**	**9**
**892**	**536**	**332**	**11**	**3**
**2325**	**1134**	**876**	**196**	**49**
**112**	**45**	**62**	**1**	**3**
**183**	**51**	**79**	**0**	**52**
**1113**	**410**	**498**	**43**	**67**
**202**	**92**	**66**	**14**	**14**
**719**	**354**	**50**	**15**	**79**
**263**				
**36**	**21**	**6**		
**98**	**7**	**3**		**42**
**223**	**106**	**65**	**5**	**4**
**272**	**112**	**97**	**14**	**8**
**1300**	**1179**	**121**		

18-4 分市、区各类卫生机构、床位、人员数（2018年底）

NUMBER OF HEALTH INSTITUTIONS，BEDS AND EMPLOYED PERSONS BY REGION（END OF 2018）

市、区名称	Region	机构数（个）Instiutions（unit）	医院小计 Sub-total of Hospitals	#医院 Hospitals	#卫生院 Sanitation Staions
**全　市**	**Whole Municpality**	**8027**	**421**	**318**	**103**
市南区	Shinan District	393	27	27	
市北区	Shibei District	758	79	79	
李沧区	Licang District	449	22	22	
崂山区	Laoshan District	423	20	18	2
黄岛区	Huangdao District	1317	60	44	16
城阳区	Chengyang District	729	28	23	5
即墨区	Jimo District	1034	50	29	21
胶州市	Jiaozhou	975	39	25	14
平度市	Pingdu	1130	55	26	29
莱西市	Laixi	819	41	25	16

注：黄岛区含保税港区数据；城阳区含红岛经济区数据。
Note：The Huangdao District contains the data of the bonded harbor area. Chengyang District contains red island economic zone data.

18-4 续表1

Continued

市、区名称	Region	采供血机构 Institutions of Blood Collection and Supply	妇幼保健院（所、站）Maternity and Child Care Centers	专科疾病防治院（所、站）Specialized Disease Prevention & Treatment Institutions
**全　市**	**Whole Municpality**	**1**	**12**	**6**
市南区	Shinan District	1	1	1
市北区	Shibei District		2	
李沧区	Licang District		1	
崂山区	Laoshan District		1	
黄岛区	Huangdao District		2	2
城阳区	Chengyang District		1	
即墨区	Jimo District		1	1
胶州市	Jiaozhou		1	
平度市	Pingdu		1	2
莱西市	Laixi		1	

注：黄岛区含保税港区数据；城阳区含红岛经济区数据。
Note：The Huangdao District contains the data of the bonded harbor area. Chengyang District contains red island economic zone data.

社区卫生服务中心<br>Neighborhood<br>Service Centers	社区卫生服务站<br>Neighborhood<br>Service Stations	门诊部<br>Outpatient<br>Service Stations	诊所、卫生所、医务室、护理站<br>Clinics Medical Houses	急救中心<br>First Aid Centers
72	208	363	2554	3
11	30	37	275	
13	53	79	526	1
14	46	35	329	
3	30	26	196	
13	21	62	406	1
10	1	66	192	1
2	21	13	195	
4		18	189	
		26	196	
2	6	1	50	

疾病预防控制中心（防疫站）<br>Centers for Disease Control and<br>Prevention（Epidemic Prevention Stations）	卫生监督所<br>Sanitation<br>Control Stations	村卫生室<br>Village health room	计划生育技术服务机构<br>Family Planning<br>Service Institutions
**26**	**11**	**4283**	**23**
1	1		
2	2		
1	1		
1	1	142	
7	1	742	
9	1	413	7
1	1	708	16
1	1	718	
1	1	846	
2	1	714	

18-4 续表 2
Continued

市、区名称	Region	健康教育所（站、中心）Health Education Station	临床检验中心（站、所）Clinical Laboratory Center	其他卫生机构 Other Health Institutions	疗养院 Sanatoriums	床位数（张）Beds in Health Institutions（bed）
**全　市**	**Whole Municpality**		**3**	**37**	**4**	**53154**
市南区	Shinan District			4	4	6424
市北区	Shibei District		1			13932
李沧区	Licang District					3260
崂山区	Laoshan District		2	1		1374
黄岛区	Huangdao District					6898
城阳区	Chengyang District					3304
即墨区	Jimo District			25		4896
胶州市	Jiaozhou			4		5096
平度市	Pingdu			2		5003
莱西市	Laixi			1		2967

注：黄岛区含保税港区数据；城阳区含红岛经济区数据。
Note： The Huangdao District contains the data of the bonded harbor area. Chengyang District contains red island economic zone data.

18-4 续表 3
Continued

市、区名称	Region	专科疾病防治院（所、站）Specialized Disease Centers	人员数（人）Personnel（person）	卫生技术人员 Medical and Technical Personnel
**全　市**	**Whole Municpality**	**224**	**102991**	**83975**
市南区	Shinan District	10	14598	11878
市北区	Shibei District		24631	21528
李沧区	Licang District		8109	7071
崂山区	Laoshan District		4375	3320
黄岛区	Huangdao District	144	12970	10511
城阳区	Chengyang District		7639	6133
即墨区	Jimo District		8710	6584
胶州市	Jiaozhou		8220	6421
平度市	Pingdu	70	7802	6037
莱西市	Laixi		5937	4492

注：黄岛区含保税港区数据；城阳区含红岛经济区数据。
Note： The Huangdao District contains the data of the bonded harbor area. Chengyang District contains red island economic zone data.

医院小计<br>Sub-total of<br>Hospitals	# 医院<br>Hospitals	# 卫生院<br>Sanitation Stations	门诊部<br>Outpatient<br>Service Stations	妇幼保健院（所、站）<br>Maternity and<br>Child Care Centers
**50842**	**45136**	**5706**		**524**
5511	5511			
13892	13892			8
3175	3175			
1245	1175	70		
6369	5463	906		150
3277	3069	208		
4773	3624	1149		100
4915	4218	697		121
4833	2917	1916		100
2852	2092	760		45

执业（助理）医师<br>Certified（Assistant）Doctors	注册护士<br>Registered Nurses	药剂人员<br>Pharmacists	检验人员<br>Laboratory Technicians	其他人员<br>Other Personnel
**34578**	**38360**	**3988**	**2615**	**3350**
4624	5734	538	424	378
8945	9995	1021	631	748
3073	3302	327	183	103
1552	1330	140	125	136
4345	4864	484	306	358
2464	2724	314	200	355
2696	2823	373	207	371
2534	3014	291	190	293
2515	2650	280	192	302
1830	1924	220	157	306

18-5 收养性社会福利单位情况(2018年)

BASIC STATICTICS ON SOCIAL WELFARE INSTITUTIONS (2018)

市、区名称	Region	单位数(个)<br>Number of Institutions<br>(unit)	年末职工人数(人)<br>Number of Staff and Workers at Year-end (person)	年末床位数(张)<br>Number of Beds at Year-end (bed)
**全　市**	**Whole Municipality**	**228**	**4682**	**38482**
市本级	Municipal Level	8	689	2402
市南区	Shinan District	17	386	2813
市北区	Shibei District	58	1319	9784
李沧区	Licang District	34	698	3496
崂山区	Laoshan District	11	283	1782
黄岛区	Huangdao District	19	468	3118
城阳区	Chengyang District	16	178	3879
即墨区	Jimo District	20	134	3393
胶州市	Jiaozhou	8	91	2378
平度市	Pingdu	25	151	2557
莱西市	Laixi	12	185	2880

注：1. 年末在院人数仅包括老年人与残疾人服务机构。2. 黄岛区含保税港区数据；城阳区含红岛经济区数据。

Note: 1. At the end of the year, the number of people in hostitals includes the elderly and disabled service organizations only.
2. The Huangdao District contains the data of the bonded harbor area. Chengyang District contains red island economic zone data.

18-6 社会救济情况(2018年)

BASIC STATISTICS ON SOCLAL RELIEF (2018)

市、区名称	Region	城镇居民最低生活保障人数(人)<br>Number of Persons Receiving Minimun Living Allowance in Urban Area (Person)	城镇居民最低生活保障家庭数(户)<br>Number of Households Receiving Minimum Living Allowance in Urban Area (household)	城镇低保资金计划支出(万元)<br>Planned Expense of Funds for Minimum Living Allowance in Urban Area (10 000 yuan)
**全　市**	**Whole Municipality**	**20457**	**13638**	**19921.5**
市南区	Shinan District	3305	2270	3359.1
市北区	Shibei District	12125	7904	11722.2
李沧区	Licang District	1922	1242	2009.9
崂山区	Laoshan District	109	64	106.9
黄岛区	Huangdao District	721	526	671
城阳区	Chengyang District	208	138	187.5
即墨区	Jimo District	927	660	855.3
胶州市	Jiaozhou	561	412	528.2
平度市	Pingdu	414	291	336
莱西市	Laixi	165	131	145.4

注：1. 全市农村低保资金计划支出中包含市本级数据。2. 黄岛区含保税港区数据；城阳区含红岛经济区数据。

Note: 1. The municipal level data shall be included in the expenditure of the rural low-insurance fund plan of the whole city.
2. The Huangdao District contains the data of the bonded harbor area. Chengyang District contains red island economic zone data.

年末在院人数（人）<br>Residences at Year-end<br>（person）	老年人<br>Senior Citizens	青壮年<br>Young and middle-aged	少年儿童<br>Youth
**20614**	**19989**	**325**	**231**
1715	1154	280	225
1289	1289		
6620	6620		
2702	2702		
585	546	39	
1603	1597	6	
1918	1918		
896	896		
671	671		
1409	1396		
1206	1200		6

农村居民最低生活保障人数（人）<br>Number of Persons Receiving<br>Minimum living Allowance in<br>Rural Area（person）	农村居民最低生活保障家庭数（户）<br>Number of Households<br>Receiving Minimum Living Allowance<br>in Rural Area（household）	农村低保资金计划支出<br>（万元）<br>Rural low-insurance fund plan expenditure<br>（10 000 yuan）
**77064**	**52421**	**51222.7**
1648	904	1357.6
12364	8449	9364
3098	2005	2390.9
17637	12151	14558.8
12225	8070	7276.5
21861	14673	12017
8231	6169	4257.9

18-7 分市、区婚姻登记情况(2018年)

BASIC STATISTICS ON MARRIAGE REGISTRATION BY REGION (2018)

市、区名称	Region	准予登记结婚 Registered Marriages		
		合计(对) Total (couple)	初婚(人) First Marriages (person)	恢复结婚(对) Resumed Marriages (couple)
**全　市**	**Whole Municipality**	**56824**	**78993**	**120**
市本级	Municipal Level	188	245	2
市南区	Shinan District	5318	7970	6
市北区	Shibei District	6717	8710	9
李沧区	Licang District	3034	4069	3
崂山区	Laoshan District	2679	4255	0
黄岛区	Huangdao District	9998	13522	0
城阳区	Chengyang District	4045	6009	0
即墨区	Jimo District	6699	9987	14
胶州市	Jiaozhou	5493	7302	1
平度市	Pingdu	8255	11097	85
莱西市	Laixi	4398	5827	0

注：黄岛区含保税港区数据；城阳区含红岛经济区数据。
Note：The Huangdao District contains the data of the bonded harbor area. Chengyang District contains red island economic zone data.

再婚（人）Remariages（person）		准予登记离婚（对）Registered Divorces（couple）	涉外婚姻登记（对）Registered Marriages with Foreigner（couple）
男 Male	女 Female		
**17212**	**17443**	**24085**	**51**
67	64	51	51
1412	1254	2229	
2453	2271	3832	
1047	952	1572	
576	527	799	
3170	3304	4467	
1067	1014	1390	
1663	1748	2006	
1800	1884	2577	
2529	2884	3467	
1428	1541	1695	

18-8 律师、公证、调解、社会治安基本情况(2000-2018年)

BASIC STATISTICS ON LAW YERS，NOTARIZATION，MEDIATION AND SOCIAL ORDER（2000-2018）

项　目	Item	单位	Unit	2000年	2005年	2008年	2009年	2010年
**一、律师工作**	**Lawyers**							
律师事务所	Law Offices	个	Unit	76	116	159	203	222
律师工作者	Lawyers	人	person	1439	1941	2915	3144	2487
专职	Full-time Lawyers	人	person	971	1542	1855	1934	2377
兼职(含特邀)	Part-time Lawyers	人	person	150	73	90	86	94
请常年法律顾问单位	Units with Permanent Legal Advisors	个	Unit	2508	3564	4086	4457	4779
全年办理民事代理	Agent of Civil Gases	件	case	5571	11104	20547	29025	26935
刑事辩护	Defender of Criminal Cases	件	case	1755	3003	3655	3758	3964
非诉讼事件	Agent of Non-litigious Legal Affairs	件	case	5251	6501	5467	5897	4274
解答法律询问	Agent of Legal Advisory Services	件	case	58385	27880	31571	30123	21102
代写法律事务文书	Agent of Legal Documents Written on Behalf of Clients	件	case	9335	8595	9077	6015	6210
**二、公证工作**	**Notarization**							
公证处	Notary Offices	个	Unit	15	15	13	13	13
公证人员	Notarial Personnel	人	person	152	196	214	214	214
公证员	Notaries	人	person	106	112	117	118	117
办理公证文书	Notarized Documents	件	case	120849	105787	101766	97019	94077
**三、人民调解**	**People's Mediation**							
人民调解委员会	People's Mediation Committees	个	Unit	9750	11480	11223	9643	8014
调解人员	Mediators	人	person	34714	46735	33875	32675	27820
调解民间纠纷	Civil Disputes Mediated	件	case	18501	13658	12803	14660	20550
**四、社会治安**	**Social Order**							
交通事故发生次数	Traffic Accidents	次	time	15601	7051	3025	2570	2313
死亡人数	Deaths	人	person	1371	776	489	402	393
受伤人数	Injuries	人	person	8620	6639	3273	2943	2504
经济损失	Losses	万元	10000 yuan	4815.70	2208.20	991.50	854.80	811.27
火灾发生次数	Fire Accidents	次	time	3088	1948	840	773	683
死亡人数	Deaths	人	person	10	3	16	5	9
受伤人数	Injuries	人	person	43	3	13		4
经济损失	Losses	万元	10000 yuan	1531.06	550.40	979.70	865.50	5566.11

2011 年	2012 年	2013 年	2014 年	2015 年	2016 年	2017 年	2018 年
247	266	285	322	337	355	371	394
2801	3130	3143	3637	3896	4260	4839	5451
2643	2944	2980	3443	3728	4134	4578	5179
105	107	93	110	112	123	127	129
5180	5228	5374	5647	6101	6294	6952	
27702	30586	31147	36317	39640	42216	47679	63277
4535	4935	4858	5370	5107	4560	4936	7490
3576	4251	4133	4833	4900	4624	5386	8486
42428	45388	53775	33717	34003	40884	55320	
6762	6388	7376	5961	6272	7326	7112	
13	13	13	13	12	12	12	12
221	225	244	249	242	250	264	265
119	118	113	119	129	125	127	125
88506	87703	99852	100553	96085	104423	105200	88619
8486	8426	8448	8027	7708	7861	7413	7366
28094	27421	27035	25646	25222	26318	23918	24761
22882	23999	19525	23737	23552	30084		29989
2164	1951	1896	1857	1814	1759	1847	1825
374	343	333	321	313	308	324	319
2240	1913	1840	1796	1785	1770	1828	1814
861.05	652.10	609.80	561.80	509.1	506.6	528.7	573.8
369	366	742	1170	1001	1654	1458	1258
1	6	7	12	10	8	13	2
2	2	11	9	3	10	9	6
598.20	601.90	2348.10	1725.50	1491.0	1433.2	2739.7	2912

18-9　分区、市殡葬服务情况 (2018 年)

BASIC STATISTICS ON FUNERAL SERVICES（2018）

区、市名称	Region	单位数（个）<br>Number of Institutions<br>（unit）	年末职工人数（人）<br>Number of Staff and Workers at Year-end<br>（person）	火化炉数（台）<br>Number of Cremators<br>（unit）	处理遗体数（具）<br>Number of Remains Cremated<br>（body）
**全　市**	**Whole Municpality**	**7**	**176**	**51**	**64720**
市本级	Municipal Level	1	47	14	13340
市南区	Shinan District				
市北区	Shibei District				
李沧区	Licang District				
崂山区	Laoshan District				
黄岛区	Huangdao District	1	18	7	9482
城阳区	Chengyang District	1	19	5	6738
即墨区	Jimo District	1	11	7	9964
胶州市	Jiaozhou	1	9	5	7017
平度市	Pingdu	1	18	7	11659
莱西市	Laixi	1	54	6	6520

注：黄岛区含保税港区数据；城阳区含红岛经济区数据。

Note：The Huangdao District contains the data of the bonded harbor area. Chengyang District contains red island economic zone data.

18-9 续表
Continued

区、市名称	Region	穴位数（个）Number of Graves（unit）	本年销售穴位数 Number of Graves Saled in the Year	安葬数（具）Number of Remains Buried（body）	本年安葬数 Number of Remains Buried in the Year
**全　市**	**Whole Municipality**	**20118**	**125**	**4105**	**520**
市本级	Municipal Level	20118	125	4105	520
市南区	Shinan District				
市北区	Shibei District				
李沧区	Licang District				
崂山区	Laoshan District				
黄岛区	Huangdao District				
城阳区	Chengyang District				
即墨区	Jimo District				
胶州市	Jiaozhou				
平度市	Pingdu				
莱西市	Laixi				

注：黄岛区含保税港区数据；城阳区含红岛经济区数据。
Note：The Huangdao District contains the data of the bonded harbor area. Chengyang District contains red island economic zone data.

主要统计指标解释

卫生机构　包括医疗机构、疾病预防控制中心（防疫站）、采供血机构、卫生监督及监测（检验）机构、医学科研和在职培训机构、健康教育所等。

医疗机构　包括医院、社区卫生服务中心（站）、疗养院、卫生院、门诊部、诊所（卫生所、医务室）、妇幼保健院（所、站）、专科疾病防治院（所、站）、急救中心（站）和临床检验中心。医疗机构分为非赢利性医疗机构和赢利性医疗机构。

医院　包括综合医院、中医医院、中西医结合医院、民族医院、各类专科医院和护理院。

医生　指在医疗、预防保健机构工作且取得《执业医师证书》的执业医师和执业助理医师。

卫生技术人员　指卫生事业机构中现任职务为卫生技术工作的人员。包括中医师、西医师、中西医结合高级医师、护师、中药师、西药师、检验师、其他技师、中医士、西医士、护士、助产士、中药剂士、西药剂士、检验士、其他技士、其他中医、护理员、中药剂员、西药剂员、检验员、其他初级卫生技术人员。

社会福利事业单位　指集中收养社会孤老、残、幼的机构，包括由民政部门管理的社会福利院、儿童福利院、精神病人福利院和城镇集体举办的福利院及农村集体举办的敬老院以及优抚医院和具有收养能力的社区服务中心等。

Explanatory Notes on Main Statistical Indicators

Health Care Institutions　include：medical institutions，disease prevention and control centers（epidemic prevention stations），blood gathering and supplying institutions，health supervision and inspection（check up）institutions，medicinal scientific research and on-job training institutions，health education and so on.

Medical Organizations　include：hospitals，health service centers（stations）of communities，nursing homes，health centers，clinics，clinics（health stations and infirmaries），maternity and child care agencies（centers and stations），special disease prevention and curing agencies（centers and stations），first aid centers（stations）and clinical inspection centers.Medical organizations are grouped by two types：profit-making and non-profit-making medical organizations.

Hospitals　include：polyclinics，traditional Chinese medical hospitals，hospitals integrated with traditional Chinese therapeutics and western therapeutics，ethical hospitals，various specialties hospitals and nursing hospitals.

Doctors　refer to certified physicians and certified assistant physicians with certifications working in medical and health care and prevention agencies.

Medical Technical Personnel　refers to those medical workers employed institutions，including doctors of Chinese and Western medicine，senior doctors of integrated Chinese-Western medicine，head nurses，pharmacists of Chinese and Western medicine，laboratory specialists，other specialists，junior doctors of Chinese and Western medicine，nurses，midwives，druggists of Chinese and Western medicine，laboratory technicians，other technicians，other practitioners of Chinese medicine，nursing attendants，pharmacological workers of Chinese and Western medicine，laboratory workers，and other primary medical personnel.

Social Welfare Institutionsr　efer to institutions taking care of old people without children，handicapped people and orphans. They include social welfare institutions run by civil affairs departments，children welfare institutions，social welfare institutions for mental patients，collective-owned old people's homes in rural areas，convalescent homes and community service centers with the capacity of receiving those people.

附 录

APPENDIX

2018 年省内各市主要经济指标对比情况

MAJOR ECONOMIC INDICATORS ON CTTIES OF THE PROVINCE（2018）

指标<br>Indicator	单位<br>Unit	山东省<br>Shandong	青岛市<br>Qingdao	济南市<br>JIman	淄博市<br>Zibo	枣庄市<br>Zaozhuang	东营市<br>Dongying
生产总值（GDP）	亿元<br>（100 million yuan）	76469.7	12001.5	7856.6	5068.4	2402.4	4152.5
比上年增长 YOY Growth	%	6.4	7.4	7.4	6.1	4.3	4.5
规上工业增加值<br>YOY Growth of Industrial Added Value above Designated Size	%	5.2	6.8	7.1	6.9	4.0	3.8
固定资产投资比上年增长<br>YOY Growth of Investment Fixed Assets	%	4.1	7.9	9.6	6.6	−19.8	−10.0
货物进出口总额<br>Imports and Exports	亿元<br>（100 million yuan）	19302.5	5321.3	870.5	950.7	105.5	1627.2
比上年增长 YOY Growth	%	7.7	5.7	13.7	35.9	5.4	20.2
# 货物出口 Exports	亿元<br>（100 million yuan）	10569.6	3172.2	564.7	416.8	98.5	368.2
比上年增长 YOY Growth	%	6.1	4.7	11.1	12.0	11.8	10.3
货物进口 Imports	亿元<br>（100 million yuan）	8732.9	2149.0	305.7	533.9	7.0	1259.0
比上年增长 YOY Growth	%	9.7	7.3	19.0	63.0	−41.4	23.5
社会消费品零售总额<br>Total Retail Sales of Consumer Goods	亿元<br>（100 million yuan）	–	4842.5	4404.5	–	950.3	867.0
比上年增长 YOY Growth	%	8.8	10.0	10.0	7.3	8.6	8.5
一般公共预算收入<br>General Public Budget Revenue	亿元<br>（100 million yuan）	6485.4	1231.9	752.8	385.2	146.7	244.6
比上年增长 YOY Growth	%	6.3	6.5	11.2	6.5	1.0	5.0
全体居民人均可支配收入<br>Disposable income per capita of all residents	元<br>yuan	29205	42019	39944	34730	24334	37586
比上年增长 YOY Growth	%	8.4	8.4	8.3	8.4	8.5	7.9
城镇居民人均可支配收入<br>Per Capita Disposable Income of Urban Households	元<br>yuan	39549	50817	50146	42277	32001	47912
比上年增长 YOY Growth	%	7.5	7.7	7.5	7.3	6.9	7.0
农村居民人均可支配收入<br>Per Capital Net Income of Rural Households	元<br>yuan	16297	20820	17924	18273	15345	17485
比上年增长 YOY Growth	%	7.8	7.5	8.0	7.8	8.3	7.6

注：山东省、淄博、济宁、威海未公布社会消费品零售总额数据。
Note：Shandong, Zibo, Jining and Weihai did not publish the total retail sales of consumer goods.

烟台市 Yantai	潍坊市 Weifang	济宁市 Jining	泰安市 Taian	威海市 Weihai	日照市 Rizhao	莱芜市 Laiwu	临沂市 Linyi	德州市 Dezhou	聊城市 Liaocheng	滨州市 Binzhou	菏泽市 Heze
7832.6	6156.8	4930.6	3651.5	3641.5	2202.2	1005.7	4717.8	3380.3	3152.2	2640.5	3078.8
6.4	6.5	5.8	5.7	6.7	7.3	7.2	7.3	6.7	5.4	1.5	7.9
6.8	5.6	6.1	5.5	6.0	8.4	8.3	8.7	7.3	2.8	–2.3	7.2
6.0	4.4	7.1	5.8	7.5	6.3	7.2	7.8	7.3	–4.3	–16.8	8.0
3047.6	1623.4	423.8	157.5	1389.8	894.9	111.0	673.8	292.8	487.0	818.4	512.5
–1.3	11.1	3.4	4.6	–1.1	–1.5	4.2	1.2	19.3	6.2	21.5	26.5
1767.0	1036.3	218.6	124.2	911.7	405.7	72.0	532.4	187.7	243.2	303.9	151.6
1.9	9.6	–7.6	7.3	6.9	16.1	3.0	8.3	4.5	4.0	12.5	–3.2
1280.6	587.1	205.2	33.2	478.1	489.2	39.0	141.5	105.1	243.8	514.5	360.9
–5.4	13.9	18.4	–4.4	–13.5	–12.5	6.4	–18.8	59.6	8.3	27.6	45.2
3079.4	2702.7	–	1593.8	–	774.1	373.2	2482.2	1525.5	1334.4	993.7	1811.4
7.8	8.8	8.3	8.8	10.1	7.5	8.8	8.5	9.3	7.7	6.7	9.8
636.6	569.8	400.0	219.5	284.4	159.8	62.6	311.8	202.5	194.3	240.5	206.0
6.0	5.7	3.7	6.0	4.2	13.0	11.7	9.3	8.0	4.2	6.3	10.4
34901	30049	25902	27389	36626	25377	29254	25545	20761	19855	26371	18858
8.1	8.0	8.6	8.5	8.4	9.0	8.3	8.6	8.8	9.2	7.4	9.5
44875	39042	34796	35196	45896	33280	37401	35727	26562	27276	35049	26176
7.3	7.6	7.3	7.5	7.5	8.1	7.2	7.4	7.8	8.1	6.5	8.5
19425	18719	16055	16959	20423	15785	17468	13638	14564	13492	16061	12848
7.6	7.4	8.2	8.2	7.7	8.6	8.2	8.1	8.8	8.7	7.7	9.3

2018 年十五个副省级城市主要经济指标对比表

MAJOR ECONOMIC INDICATORS ON CTTIES UNDER PROVINCIAL LEVELS（2018）

指标 Indicator	单位 Unit	青岛 Qingdao	沈阳 Shenyang	大连 Dalian	长春 Changchun	哈尔滨 Harbin
全市生产总值（GDP）	亿元（100 million yuan）	12001.5	6292.4	7668.5	7175.7	6300.5
比上年增长 YOY Growth	%	7.4	5.4	6.5	7.2	5.1
# 第一产业 Primary Industry	亿元（100 million yuan）	386.9	260.1	442.7		525.5
比上年增长 YOY Growth	%	3.5	3.2	3.0	1.7	-0.1
第二产业 Secondary Industry	亿元（100 million yuan）	4850.6	2376.6	3241.6		1689.3
比上年增长 YOY Growth	%	7.3	5.7	11.9	7.3	2.7
第三产业 Tertiary Industry	亿元（100 million yuan）	6764.0	3655.7	3984.2		4085.7
比上年增长 YOY Growth	%	7.7	5.4	2.9	7.8	7.5
三产占比 Three-yield ratio	%	56.4	58.1	52.0	46.9	64.8
规上工业增加值 YOY Growth of Industrial Added Value above Designated size	%	6.8	7.6	15.9	8.5	5.8
固定资产投资比上年增长 YOY Growth of Investment in Fixed Assets	%	7.9	15.3	10.1	6.7	-7.2
社会消费品零售总额 Total Retail Sales of Consumer Goods	亿元（100 million yuan）	4842.5	4051.2	3880.1	3003.6	4125.1
比上年增长 YOY Growth	%	10.0	9.2	7.8	6.2	4.2
货物进出口总额 Imports and Exports	亿元（100 million yuan）	5321.2	984.3	4701.4	1054.6	209.7
比上年增长 YOY Growth	%	5.7	13.5	13.9	10.7	-8.0
# 货物出口 Exports	亿元（100 million yuan）	3172.2	342.1	1889.6	152.5	103.5
比上年增长 YOY Growth	%	4.7	7.7	8.5	17.5	5.2
货物进口 Imports	亿元（100 million yuan）	2149.0	642.1	2811.8	902.1	106.2
比上年增长 YOY Growth	%	7.3	16.8	17.8	9.6	-18.0
一般公共预算收入 General Public Budget Revenue	亿元（100 million yuan）	1231.9	720.6	704.0	478.0	384.4
比上年增长 YOY Growth	%	6.5	10.0	7.0	6.2	4.4
城镇居民人均可支配收入 Per Capita Disposable Income of Urban Households	元（yuan）	50817	44054	43550	35332	37828
比上年增长 YOY Growth	%	7.7	6.5	7.3	6.5	6.5
农村居民人均可支配收入 Per Capltal Net Income of Rural Households	元（yuan）	20820	16530	18103	14237	16934
比上年增长 YOY Growth	%	7.5	6.9	7.3	6.0	9.0
居民消费价格指数 Consumer Price Index	%	102.1	103.0	103.0	102.0	102.5

注：部分城市数据未公布。

Note：Some of the urban income data has not been finalized.

南京 Nanjing	杭州 Hangzhou	宁波 Mingbo	厦门 Xiamen	济南 Jinan	武汉 Wuhan	广州 Guangzhou	深圳 Shenzhen	成都 Chengdu	西安 Xian
12820.4	13509.0	10745.5	4791.4	7856.6	14847.3	22859.4	24222.0	15342.8	8349.9
8.0	6.7	7.0	7.7	7.4	8.0	6.2	7.6	8.0	8.2
273.4	306.0	306.0	24.4	272.4	362.0	223.4	22.1	522.6	258.8
0.6	1.8	2.2	2.6	2.5	2.9	2.5	3.9	3.6	3.3
4721.6	4572.0	5507.5	1980.2	2829.3	6377.8	6234.1	9962.0	6516.2	2925.6
6.5	5.8	6.2	8.1	7.8	5.7	5.4	9.3	7.0	8.5
7825.4	8632.0	4932.0	2786.9	4754.8	8107.5	16401.8	14237.9	8304.0	5165.4
9.1	7.5	8.1	7.5	7.5	10.1	6.6	6.4	9.0	8.3
61.0	63.9	45.9	58.2	60.5	54.6	71.8	58.8	54.1	61.9
7.8	6.3	6.3	8.8	7.1	5.7	5.5	9.5	8.5	9.4
9.4	10.8	3.6	10.1	9.6	10.6	8.2	20.6	10.0	8.5
5832.5	5715.0	4154.9	1542.4	4404.5	6843.9	9256.2	6168.9	6801.8	4658.7
8.4	9.0	8.1	6.6	10.0	10.5	7.6	7.6	10.0	9.6
4317.2	5245.3	8576.3	6002.1	825.0	2146.0	9810.2	29983.7	4983.2	3303.9
4.7	3.1	12.9	3.2	16.2	10.9	1.0	7.0	26.4	29.6
2500.7	3417.1	5550.6	3338.5	519.3	1272.7	5607.6	16274.7	2746.9	1957.5
7.9	−1.0	11.4	2.6	14.6	10.0	−3.2	−1.6	33.0	26.1
1816.5	1828.2	3025.6	2663.5	305.7	873.3	4202.6	13709.1	2236.6	1346.4
0.6	11.8	15.7	4.0	19.0	12.2	7.1	19.4	19.2	35.0
1470.0	1825.1	1379.7	754.5	752.8	1528.7	1632.3	3538.4	1424.2	684.7
15.6	12.5	10.8	8.3	11.2	11.0	6.5	6.2	9.4	10.8
59308	61172	60134	54401	50146	47359	59982		42128	38729
8.7	8.7	8.0	8.8	7.5	9.1	8.3		8.2	8.1
25263	33193	33633	18842	17924	22652	26020		22135	13286
9.2	9.2	8.9	7.1	8.0	8.5	10.8		9.0	9.0
102.4	102.3	102.2	101.8	102.6	101.9	102.4	102.8	101.4	101.9

2018 年副省级城市之外部分城市主要经济指标情况

THE MAIN ECONOMIC INDICATORS OF SOME OTHER CITIES THAN THE SUB-PROVINCIAL CITY FOR（2018）

指标<br>Indicator	单位<br>Unit	北京<br>Beijing	天津<br>Tianjin	上海<br>Shanghai	重庆<br>Chongqing	苏州<br>Suzhou	无锡<br>Wuxi
全市生产总值（GDP）	亿元<br>（100 million yuan）	30320.0	18809.6	32679.9	20363.2	18597.5	11438.6
同比增长 Year-on-year growth	%	6.6	6.0	6.6	6.0	7.0	7.4
#第一产业 Primary Industry	亿元<br>（100 million yuan）	118.7	172.7	104.4	1378.3	214	125.1
同比增长 Year-on-year growth	%	-2.3	0.1	-6.9	4.4	-4.6	-0.3
第二产业 Secondary Industry	亿元<br>（100 million yuan）	5647.7	7609.8	9732.5	8328.8	8933.3	5464.0
同比增长 Year-on-year growth	%	4.2	1.0	1.8	3.0	5.6	8.0
第三产业 Tertiary Industry	亿元<br>（100 million yuan）	24553.6	11027.1	22843.0	10656.1	9450.2	5849.5
同比增长 Year-on-year growth	%	7.3	5.9	8.7	6.0	7.0	7.1
第三产业增加值占 GDP 比重<br>Proportion taken by the added value in GDP for the Tertiary Industry	%	81.0	58.6	69.9	52.3	50.8	51.1
规模以上工业增加值同比增长<br>YOY Growth of Industrial Added Value above Designated Size	%	4.6	2.4	10.8	0.5	5.4	9.0
固定资产投资同比增长<br>YOY Growth of Investment in Fixed Assets	%	-9.9	-5.6	5.2	7.0	4.5	5.8
社会消费品零售总额<br>Total Retail Sales of Consumer Goods	亿元<br>（100 million yuan）	25405.9		12668.7		5746.9	3672.7
同比增长 Year-on-year growth	%	7.4	1.7	7.9	8.7	7.4	9.0
进出口总额<br>Imports and Exports	亿元<br>（100 million yuan）	27182.5	8077.0	34009.9	5222.6	23375.6	6161.8
同比增长 Year-on-year growth	%	23.9	5.6	5.5	15.9	9.3	12.0
#出口总额 Exports	亿元<br>（100 million yuan）	4878.5	3207.2	13666.9	3395.3	13656.9	3743.7
同比增长 Year-on-year growth	%	23.0	8.6	4.2	17.7	7.8	12.6
进口总额 Imports	亿元<br>（100 million yuan）	22303.9	4869.9	20343.1	1827.3	9718.7	2418.2
同比增长 Year-on-year growth	%	24.1	3.8	6.4	12.5	11.4	11.6
一般公共预算收入<br>General Public Budget Revenue	亿元<br>（100 million yuan）	5785.9	2106.2	7108.2	2265.5	2120.0	1012.3
同比增长 Year-on-year growth	%	6.5	-8.8	7.0	0.6	11.1	8.8
期末金融机构本外币存款余额<br>The foreign currency deposit balance of financial institution at year end	亿元<br>（100 million yuan）	157092.2	30983.2	121112.3	36887.3	30629.4	16056.8
同比增长 Year-on-year growth	%	9.0	0.1	7.7	5.8	7.3	6.1
期末金融机构本外币贷款余额<br>The foreign currency loan balance of financial institution at year end	亿元<br>（100 million yuan）	70483.7	34084.9	73272.4	32247.8	27308.3	12102.8
同比增长 Year-on-year growth	%	1.3	7.9	9.1	13.3	9.3	7.8
居民消费价格指数<br>Consumer Price Index	%	102.5	102.0	101.6	102.0	102.6	102.3

注：部分城市数据未公布。

Note：Some of the urban income data has not been finalized.

青岛西海岸新区统计指标表

QINGDAO WEST COAST NEW DISTRICT STATISTICAL INDICATOR TABLE

指　标	Indicator	单位	Unit	2014	2015	2016	2017	2018
年末常住人口	Resident population at the end of the year	万人	10 000 persons	148.42	149.36	151.59	153.92	157.73
国民经济各部门就业人员人数	Number of employed persons in various sectors of the national economy	万人	10 000 persons	55.6	61.2	63.2	69.3	75.8
公共财政预算收入	Public Budget Revenue	亿元	100 million yuan	175.3	197.79	223.3	243.7	262.7
公共财政预算支出	Public Budget Expenditure	亿元	100 million yuan	163.53	180.48	192.7	198.5	221.7
生产总值	Total output value	亿元	100 million yuan	2404.74	2648.79	2942.08	3212.71	3517.07
第一产业	Primary Industry	亿元	100 million yuan	60.24	61.7	63.26	67.45	73.7
第二产业	Secondary Industry	亿元	100 million yuan	1219.53	1296.64	1366.60	1474.33	1578.20
# 工业	Industy	亿元	100 million yuan	1101.49	1170.27	1234.28	1315.48	1395.45
第三产业	Tertiary Industry	亿元	100 million yuan	1124.97	1290.45	1512.22	1670.93	1865.17
生产总值增长速度	Total output value growth rate	%	%	9.8	12.2	12.3	11.0	9.8
生产总值构成	Total output value composition	%	%	100	100	100	100	100
第一产业	Primary Industry	%	%	2.5	2.3	2.2	2.1	2.1
第二产业	Secondary Industry	%	%	50.7	49.0	46.4	45.9	44.9
# 工业	Industy	%	%	45.8	44.2	42.0	41.0	39.7
第三产业	Tertiary Industry	%	%	46.8	48.7	51.4	52.0	53.0
规模以上工业营业收入	Above Designated Size	亿元	100 million yuan	4516.27	5203.69	4871.86	4452.76	4264.66
规模以上工业利润总额	Total industrial profits above Designated Size	亿元	100 million yuan	196.12	254.43	287.42	289.22	246.69
房地产开发投资	Investrment In Real Estate Development	亿元	100 million yuan	228.11	233.16	210.75	275.54	282.98
社会消费品零售总额	Total Retail Sales Of Consumer Goods	亿元	100 million yuan	418.2	476.6	533.4	596.4	643.3
农林牧渔业总产值	Gross Output Value of Farming，Forestry，Animal Husbandry and Fishery	亿元	100 million yuan	105.75	110.73	122.61	129.22	138.61
粮食产量	Grain production	吨	ton	252239	237170	249128	244014	263545
肉类产量	Meat production	吨	ton	69880	68906	67625	69928	72288
水产品总产量	Output of Aquatic Products	吨	ton	347712	349496	349496	366200	350234

注：2014 年 –2017 年农业数据为根据第三次农业普查结果核定修正后数据。

Note：Crop data from 2014 to 2017 are revised based on the results of the third agricultural census.

青岛改革开放以来主要指标数据

MAIN INDICATOR DATA SINCE QINGDAO´S REFORM AND OPENING UP

指 标	Indicator	单位	Unit	1978 年
全市生产总值	GDP	亿元	100 million yuan	38.43
第一产业	Primary Industry	亿元	100 million yuan	8.73
第二产业	Secondary Industry	亿元	100 million yuan	20.25
第三产业	Tertiary Industry	亿元	100 million yuan	9.45
人均生产总值	Per Capita GDP	元	yuan	663
一般公共预算收入	General Public Budget Revenue	亿元	100 million yuan	13.07
一般公共预算支出	General Public Budget Expenditures	亿元	100 million yuan	1.94
社会消费品零售总额	Total Retail Sales of Consumer Goods	亿元	100 million yuan	10
外贸进出口总额	Imports and Exports（excluding central and provincial companies）	亿美元	100 million USD	–
# 出口总额	Exports	亿美元	100 million USD	–
实际利用外商直接投资	Actual Use Of Foreign Direct Investment	亿美元	100 million USD	–
港口吞吐量	Volume of Freight Handled in Ports	万吨	10 000 tons	2081
集装箱吞吐量	Volume of Containers Handled	万标箱	Ten Thousand TEUs	–
金融机构人民币存款余额	Financial Institution's Rmb Deposit Balance	亿元	100 million yuan	6.65
金融机构人民币贷款余额	Financial Institution's Rmb Loan Balance	亿元	100 million yuan	29.72
城镇非私营在岗职工平均工资	Average Wage Of Non–Private Employees In Urban Areas	元	yuan	584
城镇居民人均可支配收入	Per Capita Disposable Income of Urban Households	元	yuan	336
城镇居民人均消费支出	Per Capita Consumption Expenditure Of Urban Residents	元	yuan	321
农村居民人均可支配收入	Per Capita Consumption Expenditure Of Rural Residents	元	yuan	146
农村居民人均消费支出	Per Capltal Net Income of Rural Households	元	yuan	109

注：自 2015 年开始，全市城乡住户调查统一使用一体化改革后的数据，与原数据相比，城乡居民收支指标的调查范围和口径均存较大变化。故无法计算“平均每年增长（%）”一项。

Note：Since 2015, the urban and rural households investigation unified the data after the integrated reform, compared with the original data, the investigation scope and the caliber of urban and rural residents' income and expenditure indicators have changed greatly. Therefore, it is impossible to calculate the "average annual growth (%)".

1988 年	1998 年	2008 年	2018 年	平均每年增长（%）<br>Average Increase Rate（%）
142.87	901.19	4401.56	12001.52	11.7
32.72	140.66	223.4	386.91	4.8
70.32	409.6	2234.83	4850.59	12.2
39.83	350.93	1943.33	6764.02	13.0
2199	12443	52266	128459	10.4
20.12	58.0	342.44	1231.90	12.0
9.39	67.9	369.41	1561.23	18.2
59.1	336.9	1492.2	4842.5	16.7
2.8	59.6	521.6	805	20.8
2.2	38.3	314.6	480	19.7
0.3	7.3	26.4	86.9	20.8
3153	7044	30029	54250	8.5
8.6	121.3	1037.7	1931.5	19.8
75.44	818.35	4735.38	15532.19	21.4
95.54	723.92	3748.32	15194.19	16.9
1862	7518	30233	90840	13.4
1225	6542	20034	50817	–
1278	5567	15055	32890	–
820	3144	7666	20820	–
439	2112	5083	13885	–

中国统计出版社有限公司最新图书简目

(仅供参考,以实际出版为准)

统计资料

中国统计年鉴　中国统计摘要　中国第三产业统计年鉴
中国第三次全国农业普查综合资料　国际统计年鉴　金砖国家联合统计手册
中国-东盟国家统计手册　中国农村统计年鉴　中国县域统计年鉴
中国农产品价格调查年鉴　中国城市统计年鉴　中国价格统计年鉴
中国贸易外经统计年鉴　中国零售和餐饮连锁企业统计年鉴　中国商品交易市场统计年鉴
大中型批发零售和住宿餐饮企业统计年鉴　中国住户调查年鉴　中国工业统计年鉴
中国环境统计年鉴　中国能源统计年鉴　中国建筑业统计年鉴
中国房地产统计年鉴　中国固定资产投资统计年鉴　中国对外直接投资统计公报
中国人口和就业统计年鉴　中国劳动统计年鉴　中国社会统计年鉴
中国科技统计年鉴　中国高技术产业统计年鉴　全国企业创新调查年鉴
中国文化及相关产业统计年鉴　2018年时间利用调查资料　中国妇女儿童状况统计资料
中国基本单位统计年鉴　中国教育统计年鉴　中国教育经费统计年鉴
中国民族统计年鉴　中国残疾人事业统计年鉴　长江经济带发展统计年鉴

省级综合统计年鉴系列

北京 天津 河北 山西 内蒙古 辽宁 吉林 黑龙江 上海 江苏 浙江 安徽 福建 江西 山东 河南 湖北 湖南
广东 广西 海南 重庆 四川 贵州 云南 西藏 陕西 甘肃 青海 宁夏 新疆 新疆生产建设兵团

市(县)级综合统计年鉴系列

滨海新区 石家庄 唐山 邯郸 保定 沧州 邢台 廊坊 承德 衡水 秦皇岛 张家口 太原 大同 阳泉 长治 晋城
朔州 晋中 运城 忻州 临汾 吕梁 呼和浩特 鄂尔多斯 包头 沈阳 大连 长春 延吉 四平 白山 通化 哈尔滨
齐齐哈尔 黑龙江垦区 上海浦东新区 南京 无锡 徐州 常州 苏州 南通 连云港 淮安 盐城 扬州 镇江 泰州
宿迁 江阴 丹阳 海门 张家港 杭州 宁波 温州 嘉兴 湖州 绍兴 金华 衢州 舟山 台州 丽水 合肥 安庆 福州
厦门 宁德 漳州 龙岩 莆田 泉州 三明 南平 南昌 九江 上饶 新余 抚州 赣州 景德镇 济南 青岛 潍坊 枣庄
潍坊 聊城 郑州 洛阳 平顶山 三门峡 南阳 商丘 信阳 济源 汝州 武汉 十堰 荆州 宜昌 荆门 咸宁 黄冈
长沙 鹰潭 广州 深圳 惠州 东莞 汕尾 湛江 肇庆 南宁 柳州 桂林 贵港 梧州 来宾 河池 防城港 海口 三亚
儋州 成都 内江 贵阳 黔南 毕节 昆明 文山 德宏 西安 延安 安康 铜川 汉中 商洛 银川 兰州 庆阳 乌鲁木齐
昌吉 阿勒泰 兵团一师、二师、三师、四师、六师、七师、八师、十师、十三师、十四师

调查年鉴系列

天津 内蒙古 上海 河南 湖北 湖南 广东 广西 重庆 四川 云南 甘肃 宁夏 南宁 贵港 昆明

统计方法应用/实用手册

Python数据分析基础（第二版）　医用多元统计分析（第三版）　中华生物统计用表
中国国民经济核算体系（2016）基础知识　国民经济核算初级教程　医学统计学手册
全国统计专业技术资格考试系列考试用书：统计业务知识（第四版修订版）　统计业务知识学习指导与习题
全国统计专业技术资格考试系列考试用书：统计相关知识（第四版）　统计相关知识学习指导与习题

统计通俗读物/统计科普图书

领导干部统计知识问答　《防范和惩治统计造假、弄虚作假督察工作规定》辅导读本
统计新媒体运营指南　统计公文知识问答　理解国民账户　中国古代统计史简编

重点图书

新中国70年　第三次全国农业普查农作物面积遥感测量图集　中国第四次经济普查年鉴
新编英汉汉英统计大词典　中国国民经济核算体系2016　国民经济行业分类注释
挑大学选专业2019—考研择校指南　挑大学选专业2019—高考志愿填报指南　中华医学统计百科全书

发行部电话：（010）63376907　63376908　63376909　同榻行书店电话：68783171　68783172
地址：北京市丰台区西三环南路甲6号　邮政编码：100073　网址：http://www.zgtjcbs.com
